# Computational Characterization of ncRNA Fragments in Various Tissues of the *Brassica rapa* Plant

**DOI:** 10.3390/ncrna3020017

**Published:** 2017-03-24

**Authors:** Boseon Byeon, Andriy Bilichak, Igor Kovalchuk

**Affiliations:** Department of Biological Sciences, University of Lethbridge, Lethbridge, AB T1K 3M4, Canada; byeon@uleth.ca (B.B.); a.bilichak@gmail.com (A.B.)

**Keywords:** non-coding RNA, ncRNA, ncRNA fragments, ncRFs, tRF, rRF, snRF, snoRF, *Brassica rapa*, leaves, apical meristem, pollen, unpollinated ovules, pollinated ovules, embryo, endosperm

## Abstract

Recently, a novel type of non-coding RNA (ncRNA), known as ncRNA fragments or ncRFs, has been characterised in various organisms, including plants. The biogenesis mechanism, function and abundance of ncRFs stemming from various ncRNAs are poorly understood, especially in plants. In this work, we have computationally analysed the composition of ncRNAs and the fragments that derive from them in various tissues of *Brassica rapa* plants, including leaves, meristem tissue, pollen, unfertilized and fertilized ova, embryo and endosperm. Detailed analysis of transfer RNA (tRNA) fragments (tRFs), ribosomal RNA (rRNA) fragments (rRFs), small nucleolar RNA (snoRNA) fragments (snoRFs) and small nuclear RNA (snRNA) fragments (snRFs) showed a predominance of tRFs, with the 26 nucleotides (nt) fraction being the largest. Mapping ncRF reads to full-length mature ncRNAs showed a strong bias for one or both termini. tRFs mapped predominantly to the 5′ end, whereas snRFs mapped to the 3′ end, suggesting that there may be specific biogenesis and retention mechanisms. In the case of tRFs, specific isoacceptors were enriched, including tRNA^Gly(UCC)^ and tRF^Asp(GUC)^. The analysis showed that the processing of 26-nt tRF5′ occurred by cleavage at the last unpaired nucleotide of the loop between the D arm and the anticodon arm. Further support for the functionality of ncRFs comes from the analysis of binding between ncRFs and their potential targets. A higher average percentage of binding at the first half of fragments was observed, with the highest percentage being at 2–6 nt. To summarise, our analysis showed that ncRFs in *B. rapa* are abundantly produced in a tissue-specific manner, with bias toward a terminus, the bias toward the size of generated fragments and the bias toward the targeting of specific biological processes.

## 1. Introduction

Non-coding RNAs (ncRNAs) are the most abundant cellular RNAs in all organisms [1]. Among these molecules are transfer RNA (tRNAs) and ribosomal RNA (rRNAs) that are involved in translation, and small nuclear RNA (snRNA) and small nucleolar RNA (snoRNAs) that are involved in the processing and modification of other RNAs. Small ncRNAs, including miRNA, small interfering RNA (siRNA), piwi-interacting RNA (piRNA) and others, are known to regulate gene expression at both the transcriptional and posttranscriptional level via direct or indirect interaction with DNA and RNA [2]. These ncRNAs are either transcribed from their own dedicated genes or processed from mRNAs or larger ncRNAs. Recent evidence in animal and, to a lesser extent, plant studies suggests that another class of ncRNAs, known as ncRNA fragments, are also processed from tRNA [3,4,5,6,7,8,9], rRNA [6,7,8], snoNA [6,8], snRNAs [6,7,8] and several other ncRNAs [7,10,11]. These fragments are found in all kingdoms of life [7,12,13] and are typically smaller than 30 nt; they show distinctive sizes depending on precursor molecules [14,15,16] and often demonstrate a terminal bias [15,17]. They are expressed in a tissue-specific manner [9,18,19], are frequently induced by stress [17,20,21], and their expression is often uncoupled from the expression of their precursors [3,17,22,23]. These features of ncRNA fragments (ncRFs) allow us to assume they are not the result of a simple degradation process, but are produced in a specific manner, and therefore must have defined functions.

ncRNAs stemming from tRNAs are the most abundant type of ncRNA processed from other ncRNAs. Most tRNAs are 76–90 nt in size [24], have highly conserved secondary and tertiary structures, undergo extensive post-transcriptional modifications [25], and are extremely stable and resistant to nucleases [26]. The first types of ncRNAs derived from tRNAs were originally described in *Escherichia coli* and later in several organisms; they were named tRNA halves (tiRNAs). It was further demonstrated that tiRNAs are cleaved from anticodon loops in response to stress by ribonuclease angiogenin, resulting in 5′ and 3′ tiRNAs [27]. The second types of tRNA-derived ncRNAs are called tRNA fragments, or tRFs. tRNAs can generate three types of tRFs: those processed from the 5′ end—tRF5′, from the 3′ end—tRF3′, and those processed from the 3′ trailer present in the pre-tRNA—the tRF1 series [28]. tRF5′ and tRF3′ are likely produced by cleavage at the D-loop or T-loop, respectively, either by Dicer or endonucleases of similar function. The importance of Dicer for processing of tRFs is evidenced by silencing Dicer, which greatly depletes the tRF pool [3]. It has also been suggested that Dicer may process tRFs from tiRNAs, rather than from mature tRNAs or pre-tRNAs [6]. tiRNAs and tRFs regulate gene expression primarily via translation inhibition, although the cleavage of target RNAs cannot be excluded. However, tiRNAs inhibit translation initiation by interfering with the assembly of the cap binding complex eIF4F [29], while tRFs likely affect the translation elongation process [15]. Both tiRNAs and tRFs have been linked to various pathological conditions such as cancer, infection and neurodegeneration (reviewed in [30]).

ncRFs are also produced from other ncRNAs, such as snoRNAs, snRNAs and rRNAs. snoRNAs are divided into two types, C/D box and H/ACA box, with the former being ~70–120 nucleotides (nt) and the latter ~100–200 nt in size [31]. snRNAs range from 80 to 350 nt, being on average 150 nt, whereas rRNAs are several thousand nt in length.

The analysis of ncRFs in the human breast adenocarcinoma line MDA-MB-231 showed abundant ncRFs derived from tRNAs, snoRNAs, snRNAs and rRNAs [32]. Sequencing of Argonaut (AGO)-associated ncRNAs showed a substantial enrichment of tRFs of a smaller size. Curiously, the authors analysed the translational repression activity of several ncRFs and found that only snoRFs detected in more than 1000 reads per cell had the translation repression capacity [32]. Similar data were observed in miRNAs [33], suggesting that the regulatory capacity of ncRFs deriving from different small RNAs may be similar.

Specific processing of ncRNAs into ncRFs is further supported by the fact that many ncRFs are found to be enriched from one end of ncRNA and are cleaved in a specific manner, e.g., the processing of tiRNAs by angiogenin. Li et al. found that the majority of ~20-nt long fragments deriving from mature sequences of tRNAs, rRNAs, snoRNAs and snRNAs are produced in a specific manner, favouring the 5′ or 3′ ends. A similar pattern was previously observed for snoRNAs [14] and tRNAs [3,4].

Although the presence of tRFs has been documented in many species, only a handful of papers demonstrate the existence of tRFs in plants. While we were preparing a revised version of this report, the paper by Cognat et al. appeared, characterising various tRFs produced in different tissues of *Arabidopsis* plants in normal conditions and in response to ultraviolet radiation C (UVC) stress [9]. This work demonstrated that tRFs are abundantly produced from nuclear and organellar genomes by cleavage at both the D loop (tRF5′) and T loop (tRF3′). Also, in *Arabidopsis thaliana*, several tRF5′ and tRF3′ were found to be induced in roots upon phosphate deprivation [17]. Similarly to previous work in animals [3], the enrichment of tRFs by precipitation of AGO proteins was also demonstrated in *Arabidopsis* [19]; it was found that the AGO-associated tRFs differ in size distribution and abundance, suggesting that certain tRFs are enriched by specific AGO proteins and thus are likely involved in different cellular processes [19]. The presence of tRFs was also demonstrated in crops. In rice, the tissue-specific expression of tRF5s stemming from tRNA^Ala(AGC)^ and tRNA^Pro(CGG)^ tRFs was noted [18]; in barley, tRF^His(GTG)^ was the most abundant small ncRNA [34]; in wheat, tRFs processed from the tRNAs Val, Ser, Thr and Tyr were predominant [20].

Fragments of 30–70-nt in length produced from rRNA, tRNA and snRNA were found in the phloem sap of various plants [35]. It was further demonstrated that the translational inhibition in distant tissues is caused by ncRNA fragments rather than proteins present in the phloem sap. tiRNAs were proposed as long-distance silencing signals [35]. Also, the 2D-Page analysis of the RNA degradome in plants revealed the presence of 10–90-nt ncRNA fragments, with many of them being as abundant as miRNAs [36]. Translational inhibition was demonstrated using several tRNA degradants found in *Arabidopsis* [37]. Unfortunately, these reports did not demonstrate any specificity in the processing of these fragments or in any analysis of sequence reads.

Here, we attempted to characterise in detail the ncRNA fragments produced during the development of *B. rapa*. We saw tissue-specific differences in the type of ncRFs formed, their size and potential targets.

## 2. Methods

### 2.1. Sequence Data

In this work, we used the sequencing data previously produced by our lab [38]. In brief, RNA was extracted from various tissues of *B. rapa* plants, including leaves, the apical meristem, unpollinated ovules, pollinated ovules, pollen, the embryo and endosperm tissues [38]. The original dataset contained two biological replicates for each analysed tissue type. It is important to remember that, for sequencing ncRNA, a fraction of ~145–160 nt was excised from the gel, representing the adapter sequences of 55 nt (PCR index RP1; 5′-AATGATACGGCGACCACCGAGATCTACACGTTCAGAGTTCTACAGTCCGACGAUC-3′), 63 nt (RNA PCR index RPI1-RPI48; 5′-CAAGCAGAAGACGGCATACGAGATCGTGATGTGACTGGAGTTCCTTGGCACCCGAGAATTCCA-3′), and ncRNA inserts. According to Illumina, there is a discrepancy of about 7 nt between the size of fragments on the gel and their actual size (they appear to be 7 nt larger on the gel). Therefore, the potential size of the fragments excised from the gel could be 145−118−7 = 20 nt for the lower range, and 160−118−7 = 35 nt for the upper range. Single sequence reads of 36 nt were performed using Genome Analyzer IIx (Illumina, San Diego, USA) of which 29 nt were useful sequence reads and 7 nt were adapters [38].

Raw and treated reads can be found at http://www.ncbi.nlm.nih.gov/geo/query/acc.cgi?token=etuxosqqvtktjad&acc=GSE58897.

Base calling and demultiplexing of sequencing reads generated by the Illumina GAIIx platform were performed using the CASAVA v 1.8.1. software. The sequencing reads were then processed using adapter-trimming Cutadapt v 1.1 software [39], retaining only the sequences that were longer than 17 nucleotides (a Sanger quality cutoff score of 20 was applied for quality trimming). Samples that passed quality control tests were aligned to the reference sequences using Bowtie with the options of “-v 2 --best”.

### 2.2. Mapping the Reads to Genomic Regions and Mature ncRNAs

Reads were initially mapped to nuclear and organellar genomes of *B. rapa*. Reads mapping to genomic regions encoding known ncRNAs and reads mapping to the remaining genomic regions were counted separately. Reads mapping to genomic regions were further separated into reads that mapped to genic and intergenic regions. Likewise, reads mapping to genic regions were further separated into those that mapped to genic and intergenic regions.

Next, reads shorter or equal in size to 27 nt (see below) were mapped to known mature ncRNAs.

### 2.3. ncRFs Identification and Description

Sequence reads that were shorter or equal to 27 nt in length and that mapped to any part of mature ncRNAs were defined as ncRFs. In the case of tRFs, tRF5′ and tRF3′were defined as per Lee et al. [40]. Specifically, the 5′-end of ncRFs included reads that mapped to the 5′-end of the mature ncRNA, starting from the first nt, whereas the 3′-end—starting from the last nt. Only those ncRFs that had more than five reads (unique or not) mapping to any region of mature ncRNA, and that were less than or equal to the 27 nt size, were used for the analysis.

### 2.4. Analysis of Distribution of ncRNA and ncRF Reads across the Entire Length of Mature ncRNAs

Each ncRNA was divided into 10 equal-sized bins, and reads in each bin were counted across all ncRNAs in each ncRNA type. Then, the distribution of ncRNA reads was calculated by dividing the read count in each bin by the total read count across all bins in each ncRNA type. Furthermore, the distribution of ncRF reads was obtained using the same process, using reads less than or equal to 27 nt.

### 2.5. Analysis of Enrichment of ncRFs Relative to the Number of Mature ncRNAs

ncRNA reads and ncRFs were extracted for each ncRNA type. Then, the ncRNA ratio was calculated by dividing the number of ncRF reads mapping to a specific type of ncRNA by the total number of corresponding ncRNA reads for each ncRNA type. A ratio of 1 indicated that there was an equal number of ncRF reads and corresponding ncRNA reads, further showing that 50% of those ncRNAs were processed into ncRFs.

### 2.6. ncRFs Target Prediction

To identify potential targets of ncRFs we used psRNATarget [41]. To score the complementarity between ncRFs and their potential target transcript, we calculated the maximum expectation score by applying the scoring schema of miRU [42]; the maximum expectation is the threshold of this score. A small RNA/target site pair was discarded if its score was greater than the threshold. We used the default cutoff threshold of 3.0. We also identified target accessibility as the maximum energy to unpair the target site (UPE). The psRNATarget server employs RNAup to calculate target accessibility, represented by the energy required to open (unpair) the secondary structure from around the target site. The lower the energy score, the greater the possibility that ncRF will be able to bind to (cleave or inhibit translation) its potential mRNA target. Since, in addition to promoting the cleavage of mRNA, plant miRNAs also inhibit the translation of target mRNAs [43], we included both mechanisms in our analysis of ncRFs. We also included “multiplicity”—the number of potential targets for ncRFs on a single mRNA.

### 2.7. Classification of snoRNAs

We classified snoRNAs into two groups (C/D-box snoRNA and H/ACA-box snoRNA), using the SnoSeekerNGS software [44] and the *B. rapa* genome. SnoSeekerNGS predicts the C/D-box and H/ACA-box snoRNAs using the NGS sequencing data and the reference genome. Out of our 310 snoRNA sequences, 64 C/D-box snoRNAs and 13 H/ACA-box snoRNAs were classified. The “EPlBRAT00000000785” snoRNA was included in both snoRNA groups. Because SnoSeekerNGS is very slow, we predicted the C/D-box and H/ACA-box snoRNAs by using two individual sequencing data of “leaves” and “ap_meri” tissues. Then, we classified our snoRNAs based on the predicted C/D-box and H/ACA-box snoRNAs. If our snoRNA overlapped with the predicted C/D-box snoRNAs, it was classified as C/D-box snoRNA. If our snoRNA overlapped with the predicted H/ACA-box snoRNAs, it was classified as H/ACA-box snoRNA.

### 2.8. Gene Ontology Term Analysis of Targets for Each Tissue

We performed an ontological analysis of *B. rapa* using the corresponding *Arabidopsis* genes and SuperViewer. All unique target genes of ncRF sequences for each tissue type were annotated. Classification SuperViewer generates an overview of functional classification of a list of AGI IDs based on the gene ontology (GO) database. A ranking score is calculated for each functional class. The input set is bootstrapped 100 times to provide some idea as to the over- or under-representation reliability. In the figures, the significantly enriched or underrepresented classifications have *p* < 0.05. The bars indicate the Normalized Frequency, which is calculated by (Number_in_Class_input_set_/Number_Classified_input_set_)/(Number_in_Class_reference_set_/Number_Classified_reference_set_). The black range line is the bootstrap standard deviation from 100 bootstraps.

### 2.9. The Identification of Binding Consensus between ncRFs and Their Potential Target

To identify the degree of base pairing between ncRFs and their target, we calculated the consensus ratio for each nucleotide pair. The consensus ratio was calculated by dividing the number of the same nucleotides at a position by the total number of comparisons at that position.

## 3. Results and Discussion

In our previous work we analysed the memory of heat stress in various tissues of *B. rapa* plants. The initial dataset included leaf, apical meristem, pollen, unpollinated ovules, pollinated ovules, embryo and endosperm tissues. Although we had done initial ncRNA sequence analysis in these tissues, no in-depth analysis was performed. In addition, we learned about the existence of ncRNA fragments [17] and hypothesized that such fragments can also be produced in *B. rapa* and may have some functional significance. The availability of ncRNA sequencing reads from various tissues was tempting; therefore, we performed computational analysis of mature ncRNAs and ncRNA fragments derived from them. It should be noted that in our analysis we did not remove duplicate reads, as it is not commonly done when comparing two or more datasets [45].

### 3.1. Characterization of Mapped Reads in Various Tissues

**Figure 1:**
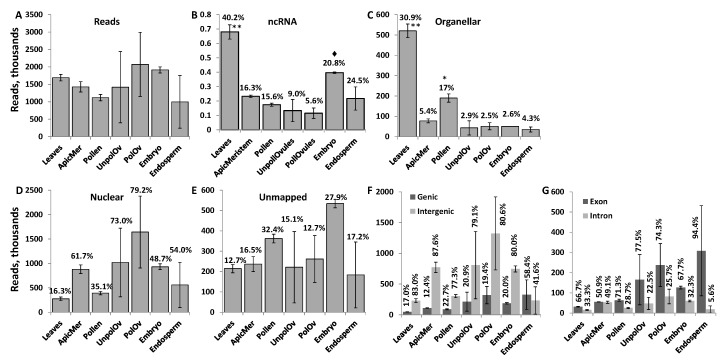
Characterisation of read number. The total number of reads (in thousands) in different tissues (**A**); and the percentage of reads mapped to genomic regions encoding ncRNAs (**B**), organellar genomes (**C**), the nuclear genome (excluding ncRNA regions) (**D**), genic versus intergenic regions (**F**), exonic versus intronic and unmapped reads (**E**). Percentages above the bars indicate the percentage of reads mapped to a specific region in a given tissue, as compared to all reads. The data shows the average calculated from two biological replicates (with standard deviation (SD)). Asterisks show significant differences in a particular tissue type as compared to other tissues (one—*p* < 0.05 and two—*p* < 0.01); the diamond symbol shows significant difference at *p* < 0.1.

The number of ncRNAs reads ranged from 2 million to almost 4 million per sample (Figure 1A). Mapping reads to the known ncRNA in the *Brassica rapa* genome revealed that most of the mapped ncRNA reads were found in leaf tissues (Figure 1B). In contrast, in other tissues, a low percentage of reads mapped to known ncRNA encoding regions. A high percentage of reads mapping to ncRNA regions in leaves, as compared to other tissues, could be due to previous reports characterizing ncRNAs in *Brassica* being based on the analysis of reads in leaf tissue [46]. A detailed RNA sequencing of different tissues of *B. rapa* did not include ncRNA sequences, therefore not allowing us to improve the annotation of genomic regions that encode ncRNA genes [47,48]. Other tissue types expressed various other, perhaps yet uncharacterised, ncRNA sequences.

Mapping the remaining reads to organellar genomes showed that leaves had the highest percentage of reads mapped (Figure 1C); this is not surprising because leaves contain a high number of chloroplasts per cell. ncRNAs and ncRNA fragments are frequently found in chloroplasts and are believed to regulate gene expression at the level of DNA and RNA [49]. Mapping the remaining reads to the nuclear genome (excluding the known ncRNA-coding regions) showed that the majority of reads in the apical meristem, unpollinated ovules, pollinated ovules and endosperm mapped to the nuclear genome (Figure 1D). In all tissues except the endosperm, the percentage of reads mapping to genic regions was around 20% or lower; in the endosperm, the majority of reads mapped to genic regions (Figure 1F). The majority of reads mapping to the genic region mapped to exons; in fact, in the endosperm tissue, 95% of all reads mapping to genic regions mapped to exons. Such a high percentage of reads mapping to exonic regions suggests that these ncRNA reads are predominantly derived from spliced mRNAs.

The largest percentage of unmapped reads was found in pollen and the embryo (Figure 1E). It was possible that a certain fraction of reads was not mapped to the genome of *B. rapa* because of insufficient annotation of the genome. We attempted to map these unmapped reads to other genomes, including *Arabidopsis* and rice, but found that only a small percentage (less than 1% on average) of these reads were mapped to these genomes (see Figure S1). Therefore, a large percentage of reads remained unmapped, likely due to the still insufficient annotation of the *B. rapa* genome.

**Figure 2:**
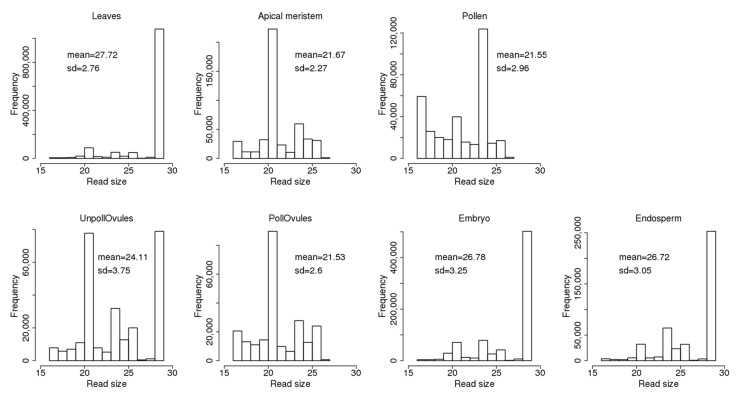
The size of non-coding RNA (ncRNA) reads mapped to known ncRNA sequences in the *Brassica rapa* genome. The *Y*-axis shows the number of reads of a certain size. The mean (with standard deviation, SD) size of reads is also shown. “UnpollOvules” = unpollinated ovules; “PollOvules” = pollinated ovules.

The read size ranged from 17 to 29 nt and was different between different tissues (Figure 2). Leaves, unpollinated ovules, the endosperm and embryo tissues had a large fraction of 29 nt reads. The fraction of 29 nt reads can include fragments of 29–35 nt, or even larger (see explanations in Section 2.1). At the same time, the apical meristem, unpollinated ovules and pollinated ovules had a large fraction of 21-nt reads that likely represent miRNAs. Meanwhile, pollen had a large fraction of 24-nt reads. Curiously, the apical meristem, pollen and pollinated ovules did not contain reads larger than 27 nt; in general, these tissues contained ncRNA reads of a shorter size. This could be due either to a more intensive degradation of ncRNAs in these tissues or the tissue-specific processing of smaller ncRNAs from larger mature ncRNAs.

### 3.2. Characterisation of Types of ncRNA Reads in Various Tissues

**Figure 3:**
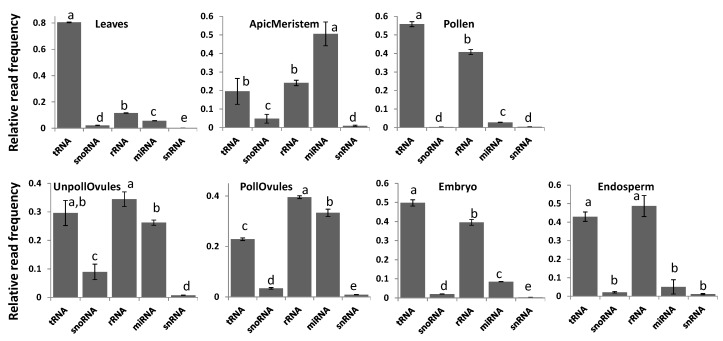
The relative read frequency of various ncRNAs in various tissues of *Brassica rapa*. The relative read frequency was calculated by dividing the read number of specific ncRNAs by the total read number. In each figure, the data points not connected by the same letters are significantly different from each other. tRNA = transfer RNA; rRNA = ribosomal RNA; snoRNA = small nucleolar RNA; snRNA = small nuclear RNA; miRNA = micro RNA; ApicMeristem = apical mersitem; UnpollOvules = unpollinated ovules; PollOvules = pollinated ovules.

Reads classified as either tRNA or rRNA were the most common among all tissue types, with the exception of the apical meristem (Figure 3). tRNA reads were predominant in leaf tissues, representing over 80% of all ncRNAs in these tissues (Figure 3; Figure S2). Since the relative number of reads was comparable in different tissues, the high percentage of tRNA reads in leaves could be due to the high translation rate in leaves. Apical meristem tissue was unusually enriched in miRNA, likely reflecting the importance of miRNAs in highly dividing cells that give rise to gametes. Reads mapping to rRNA fraction stemmed predominantly from 18S and 28S rRNAs, and there was no difference among tissues (data not shown).

Next, we analysed the size distribution of reads mapping to different types of ncRNA in different tissues. All of the analysed mature ncRNAs (tRNA, rRNA, snoRNA, snRNA), except miRNAs, were substantially larger than 29 nt. Since the libraries were enriched for sequencing with a fraction of ~20–35 nt (see Section 2.1), we were only able to sequence the passively degraded or actively processed ncRNA molecules.

**Figure 4:**
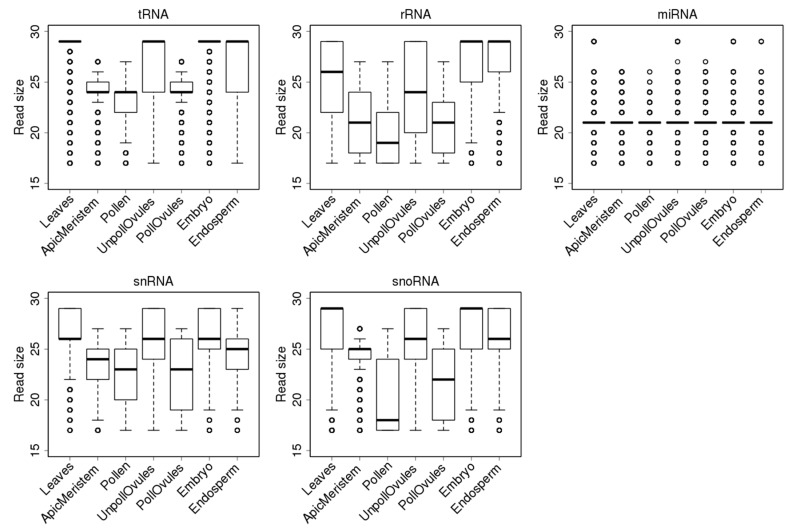
Size distribution of reads mapping to different ncRNAs in different tissues. The *y*-axis shows the size of reads. The bottom and top of the rectangle indicate the first and third quartiles, respectively. The lower and upper ends of the vertical line extending outside the rectangle represent the minimum and maximum, respectively. The thick horizontal line inside the rectangle is the median, and the circle beyond the rectangle displays an outlier.

Reads mapping to tRNAs had a median (second quartile) size of 29 nt in leaves, unpollinated ovules and the endosperm and embryo tissues. However, there was a large fraction of smaller reads in the unpollinated ovules and endosperm (Figure 4), compared to the embryo tissue. Since a 29-nt fraction includes reads of 29–35 nt, it is not surprising that it is the largest fraction and that its contribution shifts the median towards 29 nt. In contrast, in the apical meristem, pollen and pollinated ovules, most ncRNA reads were smaller than 25 nt; this similarity likely reflects a common cell lineage from the apical meristem to pollinated ovules. The fact that embryos and the endosperm do not have such a similarity may suggest a major reprogramming step is occurring in these tissues. It remains to be confirmed whether such small reads are a unique feature of gamete producing cell lineages or an artefact generated during the library preparation, cluster generation or sequencing carried out in our experiment.

Similarly to reads mapping to tRNA loci, reads mapping to rRNA loci were shorter in the apical meristem, pollen and pollinated ovules. Most reads in the embryo and endosperm were 29 nt, likely mapping to partially degraded rRNA (Figure 4). The populations of snRNAs were all smaller than 27 nt, on average. Again, in the apical meristem, pollen and pollinated ovules, reads were shorter than in other tissues (Figure 4).

**Figure 5:**
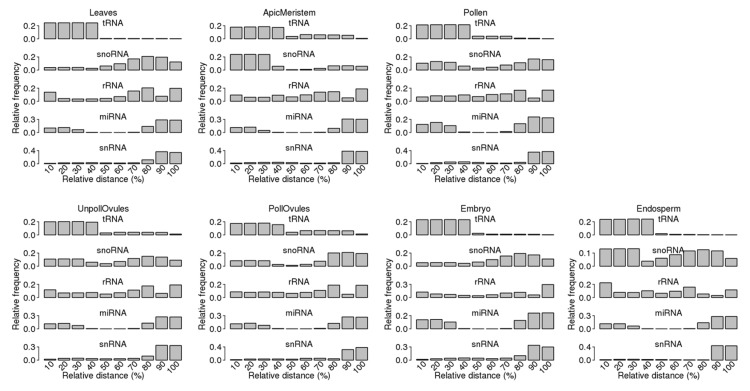
Read distribution across the whole length of the corresponding ncRNA. The entire length of each ncRNA was taken to be 100% and then divided into bins (each of 10%), with 0–10% representing the first 10% of the ncRNA length (starting from the 5′ end). The relative frequency of reads in a specific bin was calculated by dividing the frequency of reads in a specific bin by all reads mapping to the entire length of the ncRNA.

The analysis of the mapping of ncRNA reads to specific ncRNAs showed a bias in read mapping (Figure 5). Most tRNA reads mapped to the 5′ end, whereas most snRNA reads mapped to the 3′ end. Reads mapping to snoRNAs primarily mapped to either the 5′ or 3′ end, whereas reads mapping to rRNA had a more or less equal distribution across the length of loci. Finally, reads mapping to miRNA showed a clear bias towards both ends, as should be expected because miRNA precursors form hairpins as secondary structures.

**Figure 6:**
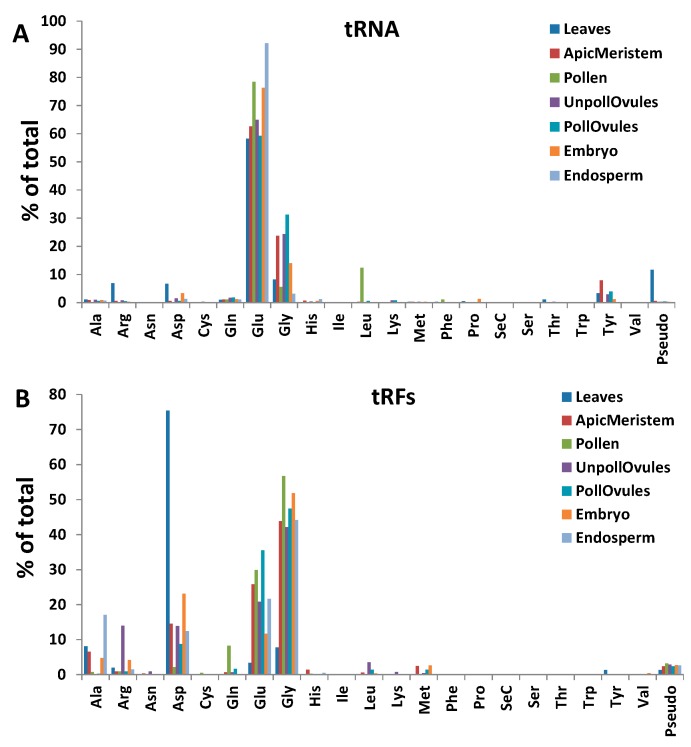
The frequency of the occurrence of reads mapping to various tRNAs (**A**); and tRFs (**B**) in different tissues of *Brassica rapa*. The *y*-axis shows the frequency (as a percentage of the total) of the occurrence of reads mapping to a specific tRNA, relative to reads mapping to all tRNAs.

The analysis of tRNA distribution in different tissues showed that tRNA^Glu^ was the most abundant tRNA in all tissues, with tRNA^Gly^ being the second most abundant (Figure 6A). Leaf tissues had an unusually high representation of tRNA^Arg^ and tRNA^Asp^, compared to other tissues (Table S1).

### 3.3. Characterisation of Size Distribution of ncRNA Fragments

While analysing ncRNAs reads, we noticed the presence of a large fraction of reads that were smaller than 29 nt. Previous reports showed that tRNAs, rRNAs, snRNAs and snoRNAs gave rise to smaller ncRNA fragments; reports in the literature also indicated that at least some of these fragments may be processed via Dicer activity [3]. The abundance of some of these fragments was altered in developmental stages and even under stress conditions [17,18,20]. Therefore, we decided to further characterise smaller reads by selecting reads that were 27 nt and shorter. This was done to contrast smaller reads from a fraction of reads of 29 nt in size (the combined fraction of ~29–35 nt reads). Since 29-nt reads were the largest reads possible in our analysis, we could not conclude whether the actual ncRNAs were 29 nt in size or larger. Libraries that were cut from the gel for sequencing were 145–160 nt in size and included the 125-nt adapter sequence, and therefore they potentially included ncRNAs of ~20–35 nt in length. By selecting reads that were 27 nt or shorter, we wanted to ensure that we were indeed dealing with ncRNAs of ≤27 nt in length that are the product of processing or degradation. We referred to these reads as tRNA fragments (tRFs), rRNA fragments (rRFs), snRNA fragments (snRFs) and snoRNA fragments (snoRFs).

ncRNA reads mapping to genomic regions that encode tRNAs were among the most common ncRNA reads in most of the analysed tissues. The analysis of the distribution of ncRNA reads by size showed that tRFs had two main fractions in most tissues, one 25–27 nt and one smaller (between 17 and 20 nt) (Figure S3A). The apical meristem and pollinated ovules tissue had much smaller fractions of 26 nt as compared to other tissues; in fact, in the apical meristem, the 25-nt fraction predominated (Figure S3A).

Information about tRFs in plants is scarce. An analysis of tRFs in *Arabidopsis* showed the presence of reads ranging from 18 to 27 nt in length, with a bias towards reads of a smaller size [19]. When the same analysis was done for a pool of reads associated with a specific Argonaut protein, it was found that AGO2 and AGO7 were enriched with shorter reads, whereas AGO1 and AGO4 had a more or less even distribution, with AGO4 having a larger fraction of longer reads associated with it, as compared to AGO1 [19]. In rice, tRFs of predominantly 21 and 22 nt in length were formed [18]. In wheat, tRFs from the 5′ and 3′ end were observed; more reads were processed from the 5′ end, and these reads were mostly 21 and 22 nt in length [20]. In contrast, reads from the 3′ end ranged from 18 to 29 nt without forming any major peak [20].

An analysis of rRFs showed two main fractions, one measuring 26 nt and the other 17–18 nt in length. The 26 nt fraction was much smaller in pollen, the pollinated ovules and the apical meristem. No 26-nt rRFs were found in pollen, which instead had a very large fraction of 17–18 nt reads (Figure S3B). snRFs had a very large fraction of 26 nt in all tissues except pollen; no fractions of 17–19 nt were found. In comparison, in wheat, rRFs ranged from 15 to 30 nt, and those stemming from 5.8 rRNA had two peaks: 22 and 24 nt [20].

snoRFs derived from the C/D-box were of a different size, but had larger fractions of 17–20 nt and 26 nt, with the 5′ nucleotide being predominantly A or U (Figure S3D). In *Arabidopsis*, sno-derived ncRNAs (sdRNAs) were associated with AGO7, C/D sdRNAs were smaller and predominantly of 21 nt, and H/ACA sdRNAs were larger and ~27–29 nt in size, in contrast to what we observed in *B. rapa*. *Arabidopsis* Argonaut proteins preferentially loaded small RNAs with the 5′ A nucleotide in H/ACA sdRNAs, and the 5′U in C/D box sdRNAs; this is similar to what we observed in *Brassica*. In wheat, 90% of reads mapped either to the 5′ or 3′ end of snoRNAs, and their size ranged from 18 to 22 nt [20].

In animals, sdRNAs from H/ACA snoRNAs were predominantly 20–24 nt in length and originated from the 3′ end. The ones derived from C/D snoRNAs showed a bimodal size distribution at ~17–19 nt and >27 nt and mainly originated from the 5′ end [14]. Therefore, the size of generated snoRFs (sdRNAs) in *Brassica*, their terminal origin, and the predominance of 5′ nt are partially similar to *Arabidopsis* and animals.

Finally, snRFs were predominantly 26 nt in size; only those snRFs in pollen had a major fraction of 21–24-nt reads and a very small fraction of 26-nt reads. The apical meristem also had a large fraction of 23-nt reads (Figure S3C). In the eukaryotic phytoplankton *Phaeodactylum tricornutum*, 21–25-nt ncRFs stemming from U2 snRNAs were found [16].

To summarise this section, the presence of specific fractions of ncRNA reads and the fact that the 27-nt fraction was either very small or not existing further suggests a specific processing of ncRNA fragments from tRNA, rRNA and snRNAs. It remains to be shown whether differences in the size of produced ncRFs are indeed due to the different mechanism of biogenesis.

### 3.4. Characterisation of tRFs, rRFs, snRFs and snoRFs

#### 3.4.1. Termini-Specific Processing of ncRNAs into Fragments

While mapping ncRNA reads to the genome, we noticed a bias towards the 5′, 3′ or both ends of ncRNA to which reads mapped (Figure 5). The phenomenon of the 5′ or 3′ end-specific processing was previously observed across all major classes of ncRNAs (except miRNAs) in normal and malignant human and murine cells [6], suggesting that it is highly likely that these ncRNA fragments are processed through an active mechanism rather than a passive degradation process. While analysing tRFs, Li et al. noticed a high degree of bias for tRFs stemming from one of the termini of a specific tRNA [6]. It was proposed that in human cells, tRFs are typically processed from both termini; however, one of the termini is retained. A similar finding was also observed in wheat seedlings, in which over two-thirds of tRFs were asymmetrically processed [20]. On the contrary, in prostate cancer cells, equal fractions of tRF-5′ and tRF-3′ were found [40].

The analysis of prostate cancer cells showed that the average size of tRF-5′ was 18–19 nt [40]. tRNA fragments in the B lymphoma BCP1 cell line were of a different size; for example, in cell lines, fragments matching the 5′ end were 14–15 nt in length, and those matching the 3′ end were 17–18 nt [6]. The discrepancy between these two reports is likely due to the latter not using reads smaller than 17 nt in the analysis [40]. A similar bias was found for snRFs in the B lymphoma BCP1 cell line; therein, snRFs were exclusively processed from the 3′ end [40]. The analysis of human lymphoma cells, mouse embryonic stem cells and HEK293 cells also showed exclusive processing of snRFs from the 3′ end. However, the authors suggested that this bias could be due to the modification of the 5′ end of snRNAs hindering the cloning of 5′ fragments, thereby leading to their absence in deep sequencing data [31].

Our analysis of the distribution of ncRNA fragments across the entire length of specific ncRNAs showed that an observed bias for 29 nt reads (Figure 5) remained nearly the same for reads of 27 nt and smaller (Figure S4). A strong bias remained toward the 5′ end for tRFs and the 3′ end for snRFs.

tRFs stemming from the 5′ end (tRF5′) were predominant in all tissues. In fact, only leaf, apical meristem and embryo tissue had a small fraction (less than 0.5%) of tRFs mapping to the 3′ end (tRF3′), whereas other tissues did not contain any tRF3′. tRF5′ arising from tRNA^Asp^, tRNA^Glu^ and tRNA^Gly^ were the most abundant. However, leaf tissues had an unusually high occurrence of tRF5′ stemming from tRNA^Asp^ and an unusually low occurrence of those from tRNA^Glu^ and tRNA^Gly^ (Figure 6B; Table S2).

Fragments stemming from snRNAs mainly came from the 3′ end of snRNAs. Fragments produced from rRNAs mapped to the entire length of rRNAs, with a bias for termini. However, when we took into consideration only rRFs that mapped exclusively to termini (starting from the first nt of the 5′ end or the last nt of the 3′ end), only rRF3′ were found. In contrast, in the case of snoRNAs, fragments were produced from both ends. Overall, snoRF3′ reads were longer than snoRF5′ reads, and they had two main fractions (17 and 26 nt) in all tissues except pollen and embryo (Figure S5).

While analysing tRFs, we noticed that some tRFs occurred more in certain tissues, as compared to tRNAs. tRFs stemming from tRNA^Gly^, tRNA^Glu^ and tRNA^Asp^ were predominant (Figure 6B). In contrast, a similar analysis performed in wheat showed that tRFs processed from the tRNAs Val, Ser, Thr and Tyr were predominant [20].

**Figure 7:**
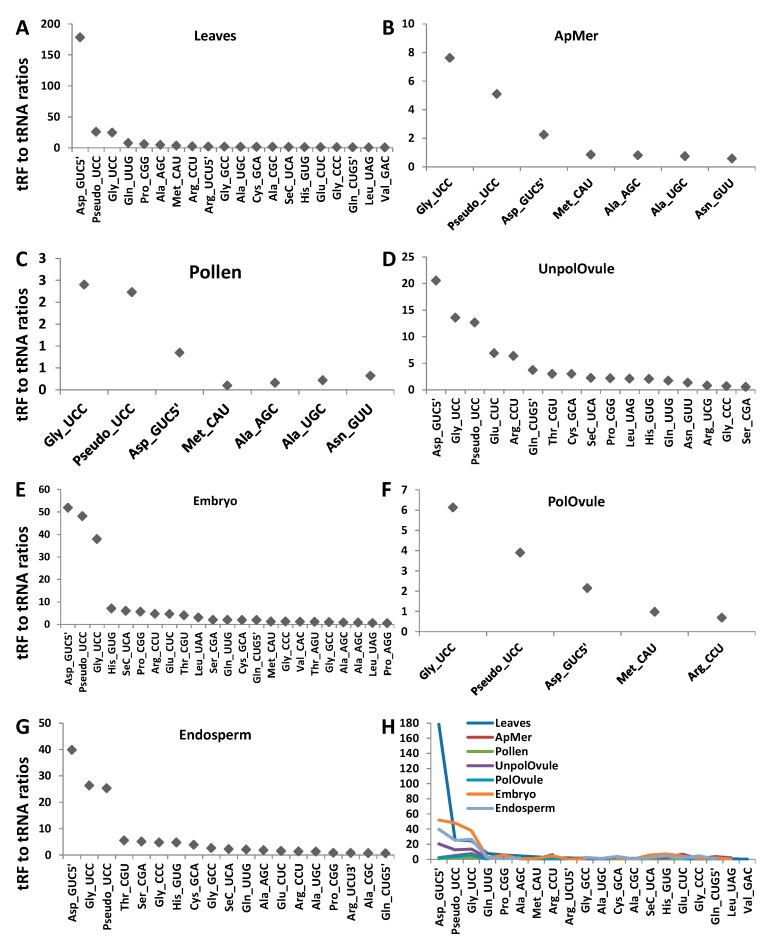
A correlation between transfer RNA (tRNA) and tRNA fragments (tRFs) in leaves (**A**), apical meristems (**B**), pollen (**C**), unpollinated ovules (**D**), pollinated ovules (**E**), embryos (**F**) and endosperms (**G**). The *y*-axis shows the tRF to tRNA ratio (tRF5′ in all cases, unless specifically labelled). The tRF to tRNA ratio was calculated by dividing the number of tRF reads in the indicated type by the total number of tRNA reads. A summary of all tissues is shown in panel H.

**Figure 8:**
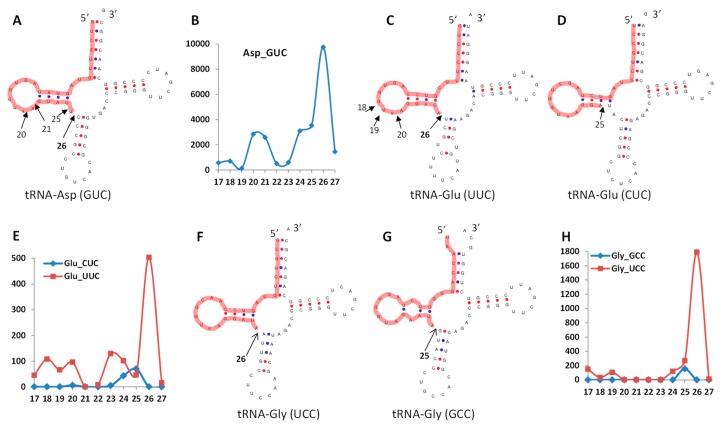
The structure of tRNAs to which the most abundant tRF reads match. (**A**,**C**,**D**,**F**,**G**) The secondary structure of tRNAs, with a thick red line indicating tRF and the arrow indicating the potential cleavage site; (**B**,**E**,**H**) the number of reads (*y*-axis) mapping to a specific tRNA, and their relative size (x-axis).

To find whether there is enrichment in any type of tRF reads, we calculated the tRF to tRNA ratio, dividing the number of tRF reads by the number of tRNA reads of the same kind in each tissue type (Figure 7). We plotted the data for those tRF:tRNA pairs whose ratio was higher than 0.5 (a ratio of 1.0, for example, means that there is an equal number of tRF and tRNA reads, indicating that one of two tRNA reads is processed to tRF (Figure 8)). The data for all tRF:tRNA pairs are shown in Table S3. The analysis showed that many tRFs were enriched in different tissues. Three isoacceptors, tRF5′^Asp(GUC)^, tRF^Gly(UCC)^ and tRF^Pseudo(UCC)^, were enriched across all tissue types, with the most substantial enrichment observed in leaves. In fact, in leaves, the tRF5′^Asp(GUC)^ to tRNA^Asp(GUC)^ ratio was over 178, indicating that over 99.5% of the tRNA^Asp(GUC)^ isoacceptor was processed to tRF5′^Asp(GUC)^. Isoacceptors of tRNA^Gly^ were enriched in all tissues, with the tRF^Gly(UCC)^ isoacceptor being enriched the most. Several isoacceptors were processed at an extremely low rate; for example, only 0.1% of all tRNA^Glu(UUC)^ was processed into tRF3' ^Glu(UUC)^ and the isoacceptor tRNA^Asp(GUC)^ was processed to tRF3′^Asp(GUC)^ at a rate of 0.2% (Table S3). The latter case is very interesting, indicating a huge bias in the processing of isoacceptor tRNA^Asp(GUC)^—the vast majority of which was processed to tRF5′^Asp(GUC)^, and very little to tRF3′^Asp(GUC)^.

Similar results were obtained in a previous analysis in *Arabidopsis*. Among all tRFs, tRF^Gly(UCC)^ was by far the most predominant [17,19], at 80%. In contrast, in rice, the most abundant tRFs came from tRNA^Ala(AGC)^ [18], and in wheat from tRNA^Val(CAC)^ [20]. It should be noted, however, that no correlation analysis between tRNA reads and tRF reads has been done in previous studies. Therefore, it is not clear whether the abundance of tRF reads is an actual enrichment, i.e., a more frequent processing of tRFs from the corresponding tRNAs. Nevertheless, these data suggest that not only specific tRNAs, but also tRNAs with specific anticodons, are predominantly processed to tRFs, and that this mechanism may be tissue-specific. We also noted that tRF enrichment was observed in all analysed tissues except leaves. It is possible that tRF enrichment is an active developmental process that occurs in generative tissues.

A similar correlation analysis between rRNA, snoRNA, and snRNAs and their respective ncRFs also showed enrichment, including 28S rRF3′, 18S rRF3′, snRF U2 and U4, snoRF-3 derived from snoR117, snoRNAZ102/R77 and snoR21 and several others (data not shown). This analysis showed that, similarly to tRFs, fragments from other ncRNAs can also be processed in an RNA-type and tissue-specific manner. We were not able to find similar data from other plants to make a meaningful comparison.

### 3.5. A Prediction of Potential Cleavage Sites in tRFs

The main tRF5′ fraction was 26 nt long. We checked the structures of tRNAs to which the most abundant tRFs were mapped, including tRNA^Glu(UUC)^, tRNA^Gly(UCC)^ and tRNA^Asp(GUC)^. We also looked at the structures of reads that mapped to the same tRNAs but different anticodons, including tRNA^Glu(CUC)^ and tRNA^Gly(GCC)^. Mapping the reads to the structure of tRNAs allowed us to hypothesise that all reads of 26 nt were likely produced through cleavage between the unpaired nucleotide connecting the D arm and the anticodon arm and the first paired nt of the anticodon arm, regardless of tRNA type (Figure 8). In the case of the less abundant 25-nt reads mapping to tRNA^Gly(GCC)^, these reads were likely produced in the same manner, whereas the less abundant 25-nt reads mapping to tRNA^Glu(CUC)^ were possibly cut from the D arm. Reads of a smaller fraction of 17–20 nt were likely processed from the D loop itself. It has to be stressed that such predictions are purely speculative and the exact mechanism of biogenesis of tRFs from the aforementioned isoacceptors remains to be validated. At the same time, a recent paper confirmed (by northern blot) the existence of ncRFs from D loop and T loop [9]; again, the exact mechanism of processing remains unknown.

### 3.6. Annotation of Potential mRNA Targets of tRFs, rRFs, snRFs and snoRFs

Previous reports suggested that ncRNA fragments can target mRNAs for degradation or for translational inhibition. Therefore, we attempted to identify potential mRNA targets for the tRFs, rRFs, snRFs and snoRFs we found, first by using the miRU–miRNA target prediction server [42] and then by classifying all these targets for a specific pathway enrichment using SuperViewer annotation. It should be emphasised that we used miRU based on the assumption that tRFs, rRFs, snRFs and snoRFs functioned similarly to miRNAs in plants. The entire list of potential targets can be found in Supplementary file 1.

There were many unique targets of tRFs in leaves, but the most common (118 tRFs) targets were *Bra010298* and *Bra010299*, both encoding hydrogen-exporting ATPase with a phosphorylative mechanism. These enzymes encode proton pumps that drive secondary transport processes. A plasma membrane proton pump is essential for the uptake of various metabolites; it is involved in various developmental processes, as well as in responses to the environment [50]. Another unique target in leaves was *Bra023165*, a gene encoding an inositol monophosphatase-like 1 (IMPL1) enzyme, which is a major enzyme required both for the de novo synthesis of inositol and the breakdown of D-inositol (1,4,5)-trisphosphate (Ins(1,4,5)P_3_), a messenger molecule involved in many plant physiological responses [51].

Among snoRF targets, there were 72 unique genes in leaves, of which all but two were targeted by ≤2 kinds of snoRFs; these two genes, *Bra038991* and *Bra011753*, were targeted by three snoRFs. The apical meristem had 52 unique targets, with 10 of them targeted by three or more snoRFs.

Among tRF targets, there were 16 gene targets common to all tissues, with most of them being targeted by more than five different tRFs (Table S4). Among them was *Bra003753*, encoding a protein similar to BEL1-like homeodomain 3. BEL1-type proteins are transcription factors that form heterodimers with KNOTTED1-like proteins, and are involved in both vegetative and floral development [52]. *Bra013336* encodes the leucine-rich repeat protein kinase, whereas *Bra037748* encodes CDKE1. CDKE1 has been proposed to be a central kinase, integrating diverse cellular signals and effectively switching between growth and stress responses [53].

*Bra000529* encodes a proteasome family protein. Bra013584 encodes WRKY DNA binding protein 31. *Bra032111* encodes LJRHL1-like 1, one of the homologs of the *Lotus japonicus ROOTHAIRLESS1* (*LjRHL1*) gene involved in root hair development [54]. *Bra023172* encodes an Argonaut family protein (AGO2). So, as it appears, tRFs likely target mRNAs encoding proteins that function in various developmental physiological processes, as well as those involved in stress responses.

### 3.7. SuperViewer Annotation of Biological Processes, Molecular Functions and Cellular Components

#### 3.7.1. The Analysis of tRF Targets

The SuperViewer analysis of the targets of tRFs showed that, among biological processes, “developmental processes” were enriched in all tissues, likely reflecting that tRFs play an important role in the regulation of developmental processes (Figure S6). The biological process “signal transduction” was enriched in all tissues except unpollinated ovules, whereas the biological process “transport” was enriched in all tissues except the endosperm. “Response to abiotic/biotic stress” and “cell organization and biogenesis” were enriched in all tissues except for unpollinated ovules and the endosperm. These results further support the idea that tRFs may have an essential role in regulating mRNAs that encode proteins involved in various biological processes across all plant tissues.

“Protein binding” was the only molecular function enriched in all tissues, likely indicating that the regulation of this process by tRFs is not tissue-specific. “The transcription factor activity” was enriched in all tissues except pollen. “Kinase activity” was enriched over two-fold in all tissues except unpollinated ovules and the endosperm. Overall, unpollinated ovules showed the lowest number of enriched biological processes, molecular functions and cellular components.

#### 3.7.2. Analysis of snRF and snoRF Targets

SuperViewer analysis of snRF targets showed that the biological process “DNA or RNA metabolism” was enriched in the apical meristem, unpollinated and pollinated ovules, and embryo tissues (data not shown). It is interesting that snRFs almost exclusively regulate one specific biological process— “DNA/RNA metabolism”. At the same time, among tRF targets, despite the abundance of tRFs, this biological process was not enriched. Thus, it appears that the processing of snRNAs likely generates a specific class of RNA fragments that might be specifically targeting “DNA or RNA metabolism”.

Among snoRF targets, biological processes such as “the developmental process”, “response to abiotic and biotic stress”, and “protein metabolism” were enriched in leaves, the apical meristem, unpollinated and pollinated ovules, and the embryo (data not shown). Our analysis showed that in a number and type of various enriched biological processes and molecular functions, tRFs and snoRFs appear to target similar processes.

### 3.8. Characterization of tRF Binding Sites

Binding of miRNAs to mRNA in plants typically results in cleavage of the substrate [55], although translational inhibition was also reported [56] and may be common in plants [43]. The cleavage typically occurs with a perfect or nearly perfect binding to the substrate, whereas translational inhibition may occur with a loose binding. One of the important parameters for translational inhibition is the binding between 2 and 8 nt of miRNA and the target [57]. The analysis of consensus binding rates between tRFs and their cleavage or translational inhibition targets showed an overall average of 77.7 ± 0.6 and 75.4 ± 1.9, respectively (Figure S7A). It was noted, however, that the consensus binding rate between 2 and 8 nt of tRFs and their targets was significantly higher (*p* < 0.05) in the case of translational inhibition, as compared to cleavage—92.0 ± 2.0 vs. 77.4 ± 2.3 (Figure S7B). Finally, the consensus binding rate was significantly higher at the first half (the first 13 nt) vs. the second half (the last 13 nt)—80.9 ± 1.3 vs. 74.6 ± 1.7 for cleavage targets, and 79.4 ± 2.6 vs. 70.2 ± 2.2 for translational inhibition (Figures S7C and S8). Similar trends were observed in rRFs, snRFs and snoRFs (data not shown).

**Figure 9:**
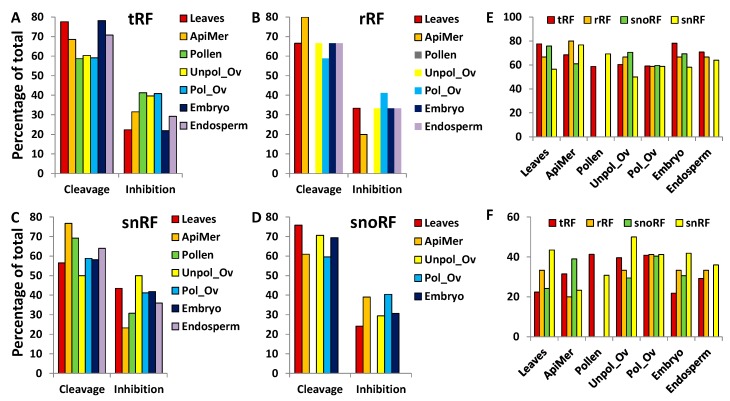
The percentage of cleavage and translational inhibition of targets by ncRFs. (**A**–**D**) data for tRF, rRF, snRF and snoRF, respectively; (**E**,**F**) summarise data for cleavage and inhibition, respectively. “ApiMer” = apical meristem; “Unpol_Ov” = unpollinated ovule; “Pol_Ov” = pollinated ovules.

Our analysis of the prediction of interaction between ncRNA fragments and their targets showed that substrate cleavage is likely to occur more often than inhibition (Figure 9). On average, cleavage took place in ~67% of cases when tRF or snoRF interacted with the target, whereas rRF and snRF caused cleavage in ~58% and 62% of cases, respectively. No significant difference between the rate of cleavage and translational inhibition was observed among the different tissues. In addition, the analysis of binding location of tRFs revealed that tRFs rarely bind to the 3′ end (~1%). tRFs that are predicted to cleave the substrate more frequently bind at the 5′ end (63% on average), whereas those that are predicted to translationally inhibit the target predominantly bind to the central portion of the target (74% on average) (Table S5). We were not able to find any extensive analysis of the position of miRNA binding to their targets in plants. At this stage it is not clear whether there is bias to binding the 5′-end, the central region or the 3′-end, as demonstrated in our analysis of tRFs. It is known, however, that in contrast to animals, where miRNAs predominantly bind 3′-untranslated region (UTR), in plants they bind the coding region of mRNA [43]. Integration of target sequences into various positions of mRNA showed that translational inhibition is much more effective when miRNAs bind to 5′-UTR and to the open reading frame, as compared to 3′-UTR [58]. Moreover, it was demonstrated that 2–4 nt mismatch only partially abolished the translation-inhibition activity.

## 4. Conclusions

It appears that the generation of ncRFs is not a random process. First, many ncRFs have a predominant size that is specific to the type of ncRNAs they derive from. Second, ncRF abundance does not fully correlate with the abundance of their respective mature ncRNAs, which has been documented in other plant species [3,17,22].

The exact role of these ncRFs is not clear, although previous reports demonstrate that many of these fragments can be enriched through the precipitation of AGO proteins. The interaction of precursors and ncRFs with AGO proteins may suggest that ncRFs function similarly to miRNAs. It can also be suggested that these ncRFs can function in association with AGO proteins by regulating the chromatin structure, alternative splicing and double-stranded break repair [59,60]. DNA and chromatin modification guided by tRFs cannot be excluded, as it was demonstrated that in these processes tRFs associate with AGO4 [61]. AGO protein-binding, RNA-binding activity and slicing activity, together with the ability of ncRFs to bind various RNA or DNA molecules, allowed a broad type of action by this duo [32]. It remains to be seen whether such ncRFs are indeed able to regulate gene expression through either cleavage and degradation or inhibition of translation.

## Supplementary Materials

The following are available online at http://www.mdpi.com/2311-553X/3/2/17/s1, File S1: tRF-Leaves, Figures S1–S5; Figure S6: SuperViewer tRF, Figure S7: Consensus binding rate; Figure S8, Table S1: Frequency of occurrence of various tRNAs in different tissues of *Brassica rapa*, Table S2: Frequency of occurrence of various tRFs in different tissues of *Brassica rapa*, Table S3: tRF to tRNA ratio for different tissues, Table S4: Common targets for various ncRFs, Table S5: Frequency of predicted binding of tRFs to various regions of their potential targets in different tissues.
