# Supplementary material for: Computational Characterization of ncRNA Fragments in Various Tissues of the Brassica rapa Plant
Source: Noncoding RNA. 2017 Mar 24;3(2):17. doi: 10.3390/ncrna3020017 (PMC5831936; doi:10.3390/ncrna3020017)
Supplement: Supplementary file 1 [file ncrna-03-00017-s001.zip › Supplementary Figures_1-5.pdf]

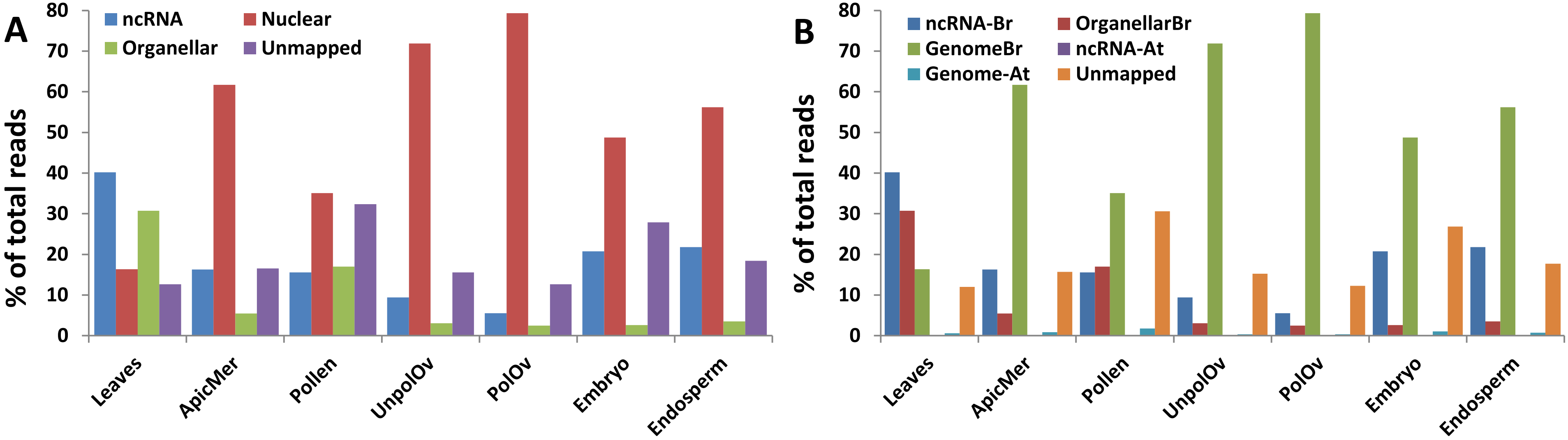

Supplementary Figure 1

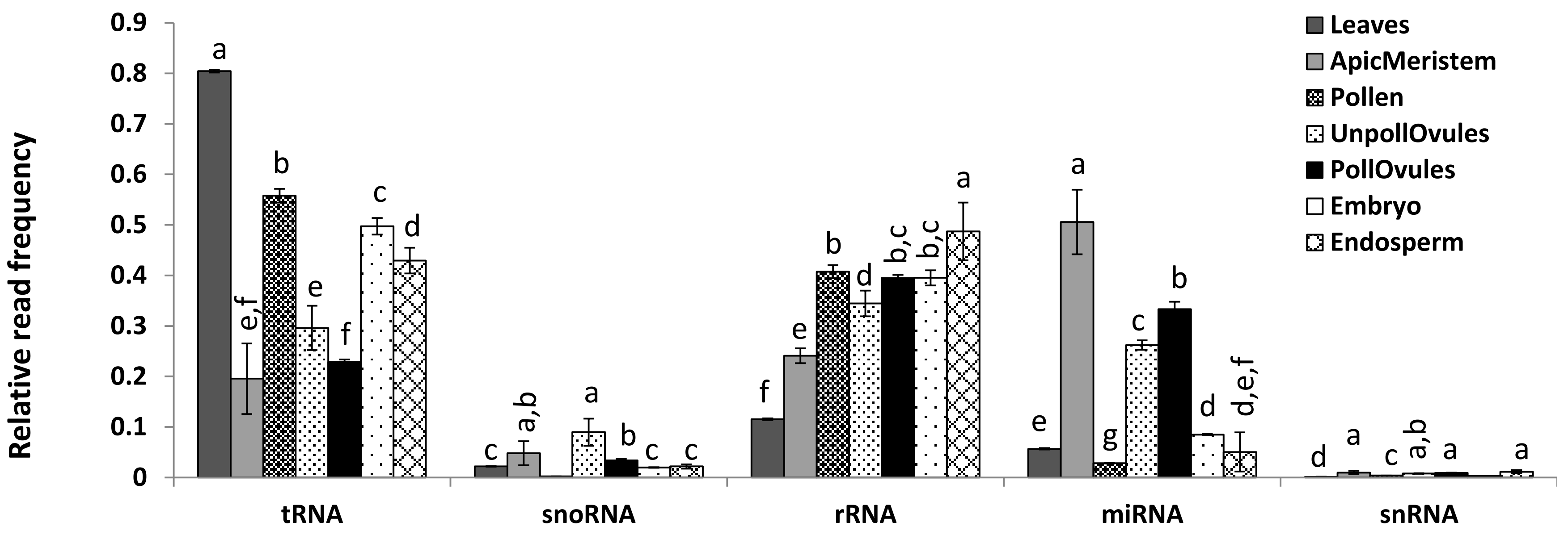

Supplementary Figure 2

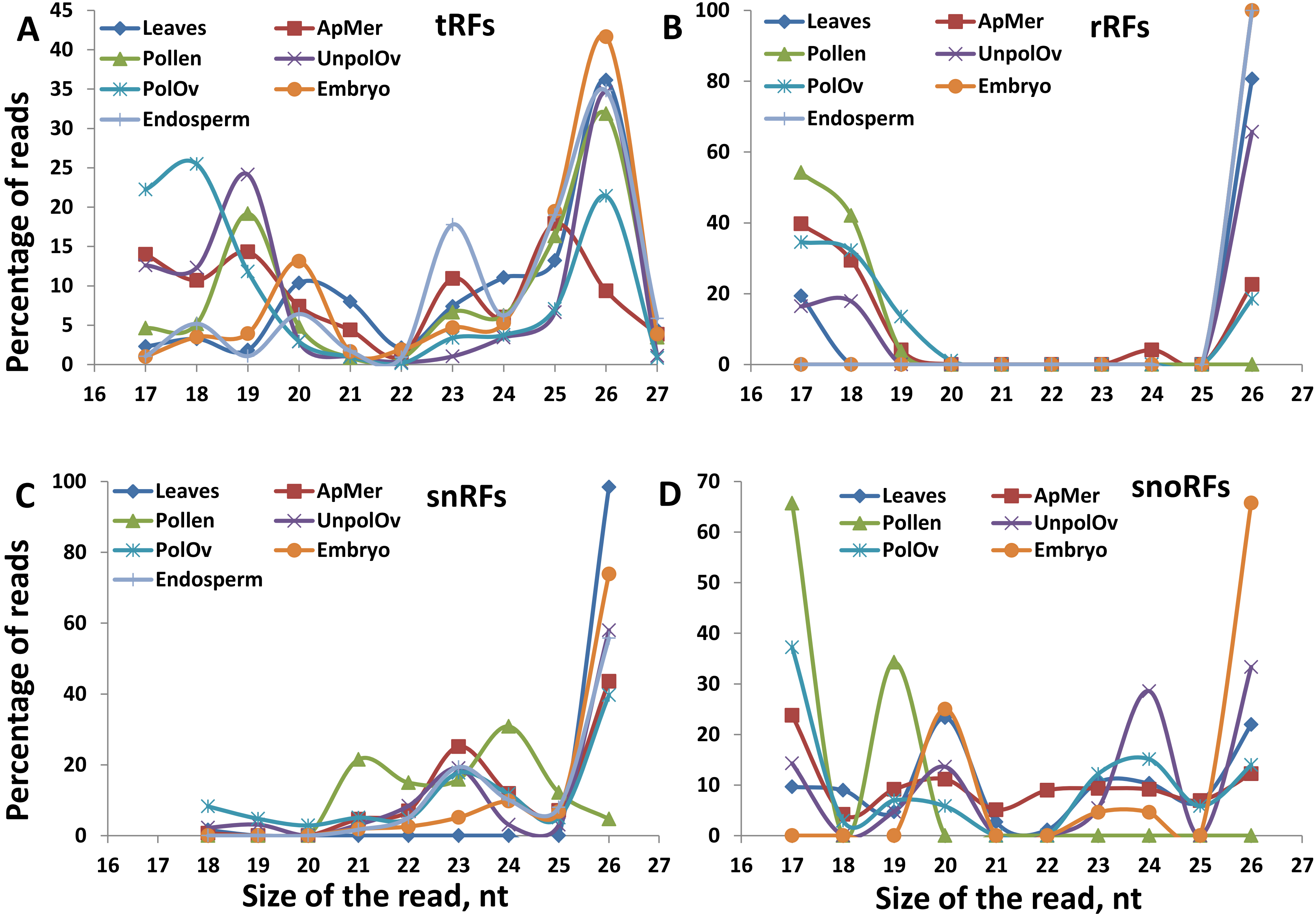

Supplementary Figure 3

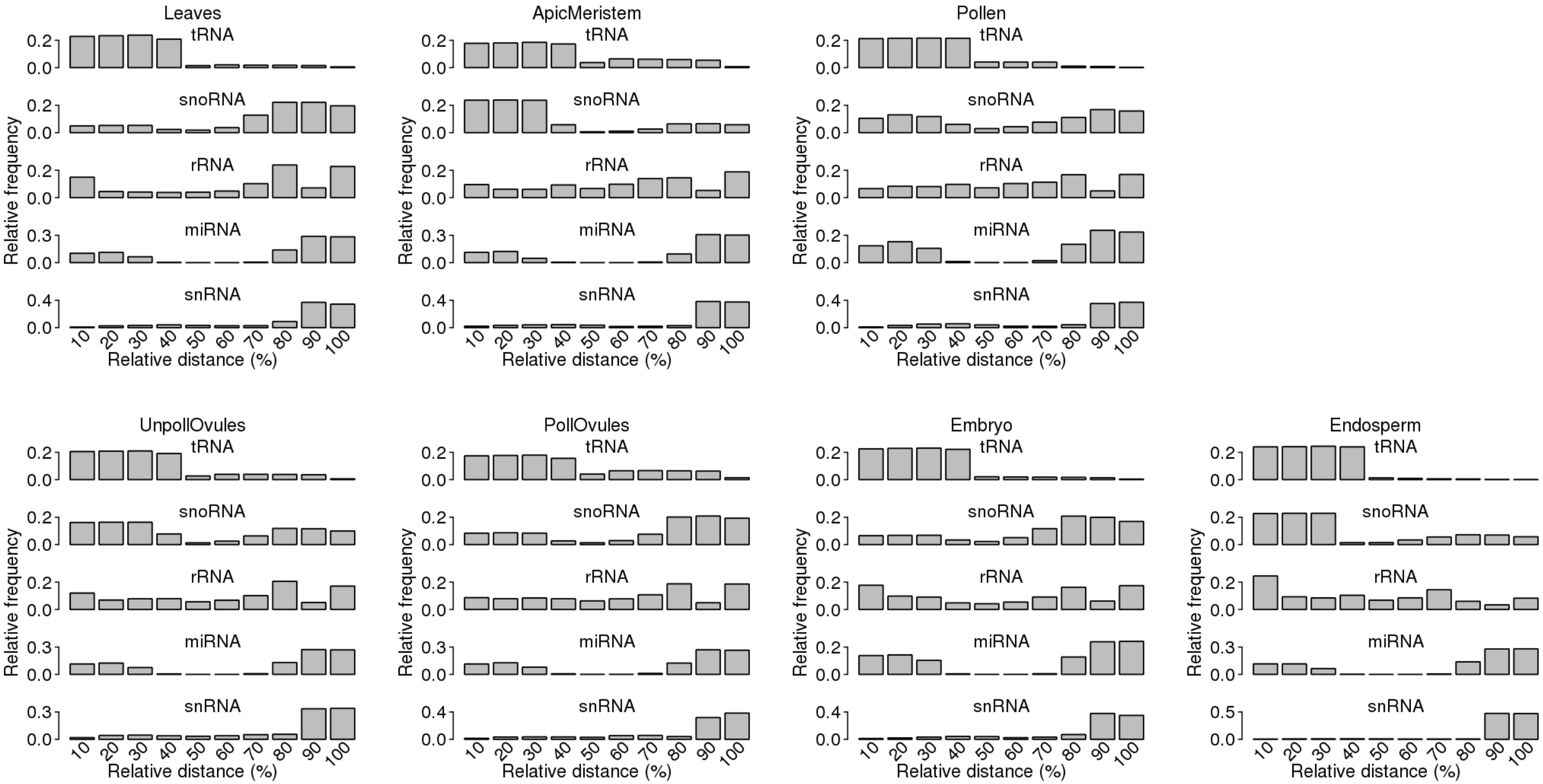

Supplementary Figure 4

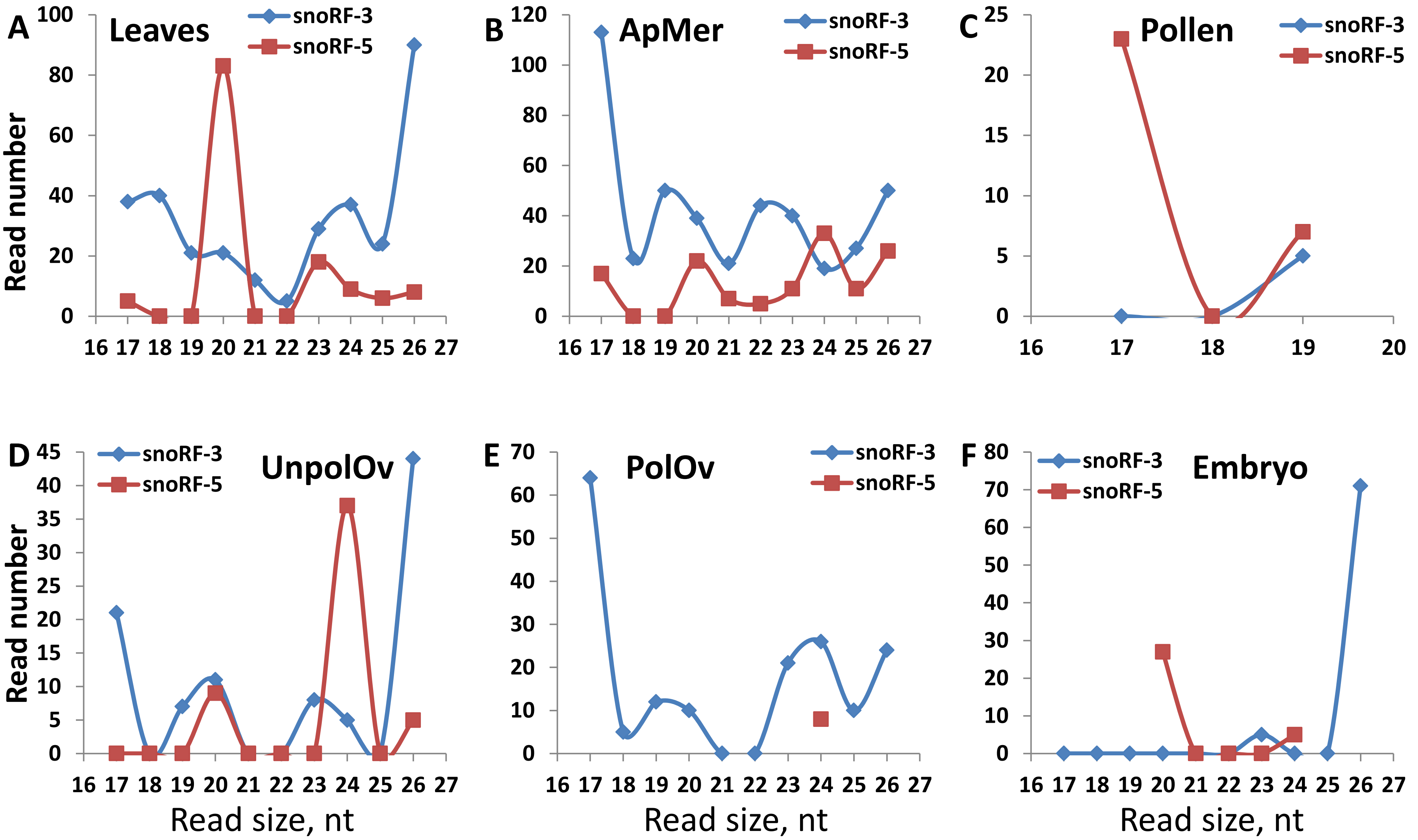

Supplementary Figure 5
