# Supplementary material for: Computational Characterization of ncRNA Fragments in Various Tissues of the Brassica rapa Plant
Source: Noncoding RNA. 2017 Mar 24;3(2):17. doi: 10.3390/ncrna3020017 (PMC5831936; doi:10.3390/ncrna3020017)
Supplement: Supplementary file 1 [file ncrna-03-00017-s001.zip › Supplementary file 1.pdf]

Supplementary file 1

tRF - Leaves

ncRNA_Acc.	Target_Acc.	Expectation	UPE	ncRNA_start	ncRNA_end	Target_start	Target_end	ncRNA_aligned_fragment	Target_aligned_fragment	Inhibition	Multiplicity
trf_1_464	Bra000111	3	14.5	1	22	635	656	GGGAUUGUAGUUAAAUUGGUCA	UGGUUCAUUUAGCUACAAUCCC	Cleavage	1
trf_1_8	Bra000529	2.5	11.2	1	21	984	1004	UCCGUUAUCGUCCAGCGGUUA	UAACCGGAGGACGAUAAUGGA	Cleavage	1
trf_1_9	Bra000529	2.5	11.2	1	21	984	1004	UCCGUUAUCGUCCAGCGGUUA	UAACCGGAGGACGAUAAUGGA	Cleavage	1
trf_1_10	Bra000529	2.5	11.2	1	21	984	1004	UCCGUUAUCGUCCAGCGGUUA	UAACCGGAGGACGAUAAUGGA	Cleavage	1
trf_1_94	Bra000529	3	11.2	1	21	984	1004	UCCGUUGUCGUCCAGCGGUUA	UAACCGGAGGACGAUAAUGGA	Cleavage	1
trf_1_95	Bra000529	3	11.2	1	21	984	1004	UCCGUUGUCGUCCAGCGGUUA	UAACCGGAGGACGAUAAUGGA	Cleavage	1
trf_1_231	Bra000529	2.5	11.2	1	21	984	1004	UCCGUUAUCGUCCAGCGGUUA	UAACCGGAGGACGAUAAUGGA	Cleavage	1
trf_1_232	Bra000529	2.5	11.2	1	21	984	1004	UCCGUUAUCGUCCAGCGGUUA	UAACCGGAGGACGAUAAUGGA	Cleavage	1
trf_1_259	Bra000529	3	11.2	1	20	985	1004	UCCGUUGUCGUCCAGCGGUU	AACCGGAGGACGAUAAUGGA	Cleavage	1
trf_1_261	Bra000529	3	11.2	1	21	984	1004	UCCGUUGUCGUCCAGCGGUUA	UAACCGGAGGACGAUAAUGGA	Cleavage	1
trf_1_287	Bra000529	3	11.2	1	21	984	1004	UCCGUUGUCGUCCAGCGGUUA	UAACCGGAGGACGAUAAUGGA	Cleavage	1
trf_1_288	Bra000529	3	11.2	1	21	984	1004	UCCGUUGUCGUCCAGCGGUUA	UAACCGGAGGACGAUAAUGGA	Cleavage	1
trf_1_510	Bra000529	3	11.2	1	21	984	1004	UCCGUUGUCGUCCAGCGGUUA	UAACCGGAGGACGAUAAUGGA	Cleavage	1
trf_1_511	Bra000529	3	11.2	1	21	984	1004	UCCGUUGUCGUCCAGCGGUUA	UAACCGGAGGACGAUAAUGGA	Cleavage	1

trf_1_512	Bra000529	3	11.2	1	21	984	1004	UCCGUUGUCGUCCAGCGGUUA	UAACCGGAGGACGAUAAUGGA	Cleavage	1
trf_1_126	Bra000788	3	16.6	1	20	52	71	GACGGUUUGGCCGAGUGGUC	AACCAUUCCGCCAAAUCGUC	Cleavage	1
trf_1_54	Bra000881	2.5	11.0	1	24	162	185	GUCUGGGUGGUGUAGUCGGUUAUC	GCUAACCAACUACACCAUCCAGAA	Cleavage	1
trf_1_188	Bra000881	2.5	11.0	1	24	162	185	GUCUGGGUGGUGUAGUCGGUUAUC	GCUAACCAACUACACCAUCCAGAA	Cleavage	1
trf_1_528	Bra000881	2.5	11.0	1	24	162	185	GUCUGGGUGGUGUAGUCGGUUAUC	GCUAACCAACUACACCAUCCAGAA	Cleavage	1
trf_1_425	Bra000885	3	17.3	1	26	398	423	UGGAUUGUAGUUCAAUUGGUCAGAGC	GCUCUUACCAGUUGAACUACAAAUCG	Cleavage	1
trf_1_462	Bra000885	3	17.3	1	25	399	423	UGGAUUGUAGUUCAAUUGGUCAGAG	CUCUUACCAGUUGAACUACAAAUCG	Cleavage	1
trf_1_466	Bra000885	3	17.3	1	26	398	423	UGGAUUGUAGUUCAAUUGGUCAGAGC	GCUCUUACCAGUUGAACUACAAAUCG	Cleavage	1
trf_1_105	Bra001550	3	24.2	1	22	1006	1027	ACCUACUUAACUCAGUGGUUAG	CUAACCAUGGCGUUAAGUGGGU	Cleavage	1
trf_1_460	Bra002337	3	25.0	1	20	863	882	GGAUUGUAGUUCAAUUGUC	GGCAGUUGAUCUACAAUUUC	Translation	1
trf_1_126	Bra002609	3	10.2	1	20	1237	1256	GACGGUUUGGCCGAGUGGUC	GACUUUCCGGCCAAACCGUC	Cleavage	1
trf_1_195	Bra002746	3	16.3	1	21	218	238	AGGGAUAUAACUCAGCGGUAG	CUGCUGCUGAGUUCUAUCCCA	Cleavage	1
trf_1_196	Bra002746	3	16.3	1	21	218	238	AGGGAUAUAACUCAGCGGUAG	CUGCUGCUGAGUUCUAUCCCA	Cleavage	1
trf_1_197	Bra002746	3	16.3	1	21	218	238	AGGGAUAUAACUCAGCGGUAG	CUGCUGCUGAGUUCUAUCCCA	Cleavage	1
trf_1_198	Bra002746	3	16.3	1	20	219	238	AGGGAUAUAACUCAGCGGUA	UGCUGCUGAGUUCUAUCCCA	Cleavage	1
trf_1_199	Bra002746	3	16.3	1	21	218	238	AGGGAUAUAACUCAGCGGUAG	CUGCUGCUGAGUUCUAUCCCA	Cleavage	1
trf_1_529	Bra003013	3	16.5	1	20	319	338	GCUGGAGUAGCUCAGUUGGU	CCUAACGGAGCUGCUCCAGC	Cleavage	1

trf_1_54	Bra003121	3	15.1	1	24	760	783	GUCUGGGUGGUGUAGUCGGUUAUC	GCUCACCAGCUACACCACACAGAC	Cleavage	1
trf_1_188	Bra003121	3	15.1	1	24	760	783	GUCUGGGUGGUGUAGUCGGUUAUC	GCUCACCAGCUACACCACACAGAC	Cleavage	1
trf_1_528	Bra003121	3	15.1	1	24	760	783	GUCUGGGUGGUGUAGUCGGUUAUC	GCUCACCAGCUACACCACACAGAC	Cleavage	1
trf_1_126	Bra003534	3	24.7	1	20	3589	3608	GACGGUUUGGCCGAGUGGUC	GGCGAUUCGGCCAAAUUGUC	Cleavage	1
trf_1_418	Bra003597	3	13.1	1	20	1508	1527	GGGAUUGUAGUUCAAUUGGA	UCCAGUUAAUCUACAAUUCC	Translation	1
trf_1_419	Bra003597	3	13.1	1	20	1508	1527	GGGAUUGUAGUUCAAUUGGA	UCCAGUUAAUCUACAAUUCC	Translation	1
trf_1_434	Bra003597	3	13.1	1	20	1508	1527	GGGAUUGUAGUUCAAUUGGA	UCCAGUUAAUCUACAAUUCC	Translation	1
trf_1_445	Bra003597	3	13.1	1	20	1508	1527	GGGAUUGUAGUUCAAUUGGA	UCCAGUUAAUCUACAAUUCC	Translation	1
trf_1_447	Bra003597	3	13.1	1	21	1507	1527	GGGAUUGUAGUUCAAUUGGAU	AUCCAGUUAAUCUACAAUUCC	Translation	1
trf_1_456	Bra003597	3	13.1	1	20	1508	1527	GGGAUUGUAGUUCAAUUGGA	UCCAGUUAAUCUACAAUUCC	Translation	1
trf_1_465	Bra003597	3	13.1	1	20	1508	1527	GGGAUUGUAGUUCAAUUGGA	UCCAGUUAAUCUACAAUUCC	Translation	1
trf_1_529	Bra003602	3	23.1	1	23	271	293	GCUGGAGUAGCUCAGUUGGUUAG	CGAACCAACCGGGCUACUGCAGC	Cleavage	1
trf_1_413	Bra003753	2.5	16.2	1	20	284	303	GGGAUUGUAGUUCAAUCGGU	ACCAGUUGAAUUACAAUCCU	Cleavage	1
trf_1_414	Bra003753	1.5	16.2	1	20	284	303	GGGAUUGUAGUUCAAUUGGU	ACCAGUUGAAUUACAAUCCU	Cleavage	1
trf_1_415	Bra003753	1.5	16.2	1	20	284	303	GGGAUUGUAGUUCAAUUGGU	ACCAGUUGAAUUACAAUCCU	Cleavage	1
trf_1_416	Bra003753	1.5	16.2	1	20	284	303	GGGAUUGUAGUUCAAUUGGU	ACCAGUUGAAUUACAAUCCU	Cleavage	1
trf_1_418	Bra003753	2.5	16.2	1	20	284	303	GGGAUUGUAGUUCAAUUGGA	ACCAGUUGAAUUACAAUCCU	Cleavage	1

trf_1_419	Bra003753	2.5	16.2	1	20	284	303	GGGAUUGUAGUUCAAUUGGA	ACCAGUUGAAUUACAAUCCU	Cleavage	1
trf_1_420	Bra003753	2.5	16.2	1	20	284	303	GGGAUUGUAGUUCAAUUGGC	ACCAGUUGAAUUACAAUCCU	Cleavage	1
trf_1_421	Bra003753	2	16.2	1	20	284	303	NGGAUUGUAGUUCAAUUGGU	ACCAGUUGAAUUACAAUCCU	Cleavage	1
trf_1_422	Bra003753	1.5	16.2	1	20	284	303	GGGAUUGUAGUUCAAUUGGU	ACCAGUUGAAUUACAAUCCU	Cleavage	1
trf_1_423	Bra003753	1.5	16.2	1	20	284	303	GGGAUUGUAGUUCAAUUGGU	ACCAGUUGAAUUACAAUCCU	Cleavage	1
trf_1_424	Bra003753	1.5	16.2	1	20	284	303	GGGAUUGUAGUUCAAUUGGU	ACCAGUUGAAUUACAAUCCU	Cleavage	1
trf_1_425	Bra003753	2	16.2	1	20	284	303	UGGAUUGUAGUUCAAUUGGU	ACCAGUUGAAUUACAAUCCU	Cleavage	1
trf_1_426	Bra003753	1.5	16.2	1	20	284	303	GGGAUUGUAGUUCAAUUGGU	ACCAGUUGAAUUACAAUCCU	Cleavage	1
trf_1_428	Bra003753	3	16.2	1	20	284	303	GGGAUCGUAGUUCAAUUGGU	ACCAGUUGAAUUACAAUCCU	Cleavage	1
trf_1_429	Bra003753	1.5	16.2	1	20	284	303	GGGAUUGUAGUUCAAUUGGU	ACCAGUUGAAUUACAAUCCU	Cleavage	1
trf_1_430	Bra003753	2.5	16.2	1	21	283	303	GGGAUUGUAGUUCAAUUGGCU	AACCAGUUGAAUUACAAUCCU	Cleavage	1
trf_1_431	Bra003753	1.5	16.2	1	20	284	303	GGGAUUGUAGUUCAAUUGGU	ACCAGUUGAAUUACAAUCCU	Cleavage	1
trf_1_433	Bra003753	2.5	16.2	1	20	284	303	GGGAUUGUAGUUCAAUUGGG	ACCAGUUGAAUUACAAUCCU	Cleavage	1
trf_1_434	Bra003753	2.5	16.2	1	20	284	303	GGGAUUGUAGUUCAAUUGGA	ACCAGUUGAAUUACAAUCCU	Cleavage	1
trf_1_435	Bra003753	2	16.2	1	20	284	303	NGGAUUGUAGUUCAAUUGGU	ACCAGUUGAAUUACAAUCCU	Cleavage	1
trf_1_436	Bra003753	2.5	16.2	1	20	284	303	GGGAUUGUAGUUCAAUCGGU	ACCAGUUGAAUUACAAUCCU	Cleavage	1
trf_1_437	Bra003753	2.5	16.2	1	20	284	303	GGGAUUGUAGUUCAAUUGGC	ACCAGUUGAAUUACAAUCCU	Cleavage	1

trf_1_438	Bra003753	1.5	16.2	1	20	284	303	GGGAUUGUAGUUCAAUUGGU	ACCAGUUGAAUUACAAUCCU	Cleavage	1
trf_1_439	Bra003753	1.5	16.2	1	20	284	303	GGGAUUGUAGUUCAAUUGGU	ACCAGUUGAAUUACAAUCCU	Cleavage	1
trf_1_440	Bra003753	1.5	16.2	1	20	284	303	GGGAUUGUAGUUCAAUUGGU	ACCAGUUGAAUUACAAUCCU	Cleavage	1
trf_1_441	Bra003753	2.5	16.2	1	20	284	303	GGGAUUGUAGUUCAAUUGGG	ACCAGUUGAAUUACAAUCCU	Cleavage	1
trf_1_442	Bra003753	2.5	16.2	1	20	284	303	GGGAUUGUAGUUCAAUUGGG	ACCAGUUGAAUUACAAUCCU	Cleavage	1
trf_1_443	Bra003753	1.5	16.2	1	20	284	303	GGGAUUGUAGUUCAAUUGGU	ACCAGUUGAAUUACAAUCCU	Cleavage	1
trf_1_444	Bra003753	1.5	16.2	1	20	284	303	GGGAUUGUAGUUCAAUUGGU	ACCAGUUGAAUUACAAUCCU	Cleavage	1
trf_1_445	Bra003753	2.5	16.2	1	20	284	303	GGGAUUGUAGUUCAAUUGGA	ACCAGUUGAAUUACAAUCCU	Cleavage	1
trf_1_447	Bra003753	2.5	16.2	1	21	283	303	GGGAUUGUAGUUCAAUUGGAU	AACCAGUUGAAUUACAAUCCU	Cleavage	1
trf_1_448	Bra003753	2.5	16.2	1	20	284	303	GGGAUUGUAGUUCAAUUGGC	ACCAGUUGAAUUACAAUCCU	Cleavage	1
trf_1_449	Bra003753	1.5	16.2	1	20	284	303	GGGAUUGUAGUUCAAUUGGU	ACCAGUUGAAUUACAAUCCU	Cleavage	1
trf_1_450	Bra003753	1.5	16.2	1	20	284	303	GGGAUUGUAGUUCAAUUGGU	ACCAGUUGAAUUACAAUCCU	Cleavage	1
trf_1_451	Bra003753	1.5	16.2	1	20	284	303	GGGAUUGUAGUUCAAUUGGU	ACCAGUUGAAUUACAAUCCU	Cleavage	1
trf_1_453	Bra003753	1.5	16.2	1	20	284	303	GGGAUUGUAGUUCAAUUGGU	ACCAGUUGAAUUACAAUCCU	Cleavage	1
trf_1_454	Bra003753	2.5	16.2	1	20	284	303	GGGAUUGUAGUUCAAUCGGU	ACCAGUUGAAUUACAAUCCU	Cleavage	1
trf_1_455	Bra003753	1.5	16.2	1	20	284	303	GGGAUUGUAGUUCAAUUGGU	ACCAGUUGAAUUACAAUCCU	Cleavage	1
trf_1_456	Bra003753	2.5	16.2	1	20	284	303	GGGAUUGUAGUUCAAUUGGA	ACCAGUUGAAUUACAAUCCU	Cleavage	1

trf_1_457	Bra003753	1.5	16.2	1	21	283	303	GGGAUUGUAGUUCAAUUGGUU	AACCAGUUGAAUUACAAUCCU	Cleavage	1
trf_1_458	Bra003753	1.5	16.2	1	20	284	303	GGGAUUGUAGUUCAAUUGGU	ACCAGUUGAAUUACAAUCCU	Cleavage	1
trf_1_459	Bra003753	2.5	16.2	1	20	284	303	GGGAUUGUAGUUCAAUCGGU	ACCAGUUGAAUUACAAUCCU	Cleavage	1
trf_1_461	Bra003753	2	16.2	1	20	284	303	NGGAUUGUAGUUCAAUUGGU	ACCAGUUGAAUUACAAUCCU	Cleavage	1
trf_1_462	Bra003753	2	16.2	1	20	284	303	UGGAUUGUAGUUCAAUUGGU	ACCAGUUGAAUUACAAUCCU	Cleavage	1
trf_1_463	Bra003753	1.5	16.2	1	20	284	303	GGGAUUGUAGUUCAAUUGGU	ACCAGUUGAAUUACAAUCCU	Cleavage	1
trf_1_464	Bra003753	2.5	16.2	1	20	284	303	GGGAUUGUAGUUAAAUUGGU	ACCAGUUGAAUUACAAUCCU	Cleavage	1
trf_1_465	Bra003753	2.5	16.2	1	20	284	303	GGGAUUGUAGUUCAAUUGGA	ACCAGUUGAAUUACAAUCCU	Cleavage	1
trf_1_466	Bra003753	2	16.2	1	20	284	303	UGGAUUGUAGUUCAAUUGGU	ACCAGUUGAAUUACAAUCCU	Cleavage	1
trf_1_467	Bra003753	1.5	16.2	1	22	282	303	GGGAUUGUAGUUCAAUUGGUCC	GAACCAGUUGAAUUACAAUCCU	Cleavage	1
trf_1_468	Bra003753	1.5	16.2	1	21	283	303	GGGAUUGUAGUUCAAUUGGUU	AACCAGUUGAAUUACAAUCCU	Cleavage	1
trf_1_469	Bra003753	2.5	16.2	1	20	284	303	GGGAUUGUAGUUCAAUUGGC	ACCAGUUGAAUUACAAUCCU	Cleavage	1
trf_1_470	Bra003753	3	16.2	1	20	284	303	NGGAUUGUAGUUCAAUUGGA	ACCAGUUGAAUUACAAUCCU	Cleavage	1
trf_1_471	Bra003753	1.5	16.2	1	22	282	303	GGGAUUGUAGUUCAAUUGGUCC	GAACCAGUUGAAUUACAAUCCU	Cleavage	1
trf_1_182	Bra004481	3	20.0	1	20	1660	1679	CCGACCUUAGCUCAGUUGGU	GUUAACUGGGCUAAGGUCGC	Cleavage	1
trf_1_476	Bra004481	3	20.0	1	20	1660	1679	CCGACCUUAGCUCAGUUGGU	GUUAACUGGGCUAAGGUCGC	Cleavage	1
trf_1_478	Bra004481	3	20.0	1	20	1660	1679	CCGACCUUAGCUCAGUUGGU	GUUAACUGGGCUAAGGUCGC	Cleavage	1

trf_1_479	Bra004481	3	20.0	1	20	1660	1679	CCGACCUUAGCUCAGUUGGU	GUUAACUGGGCUAAGGUCGC	Cleavage	1
trf_1_520	Bra004481	3	20.0	1	20	1660	1679	CCGACCUUAGCUCAGUUGGU	GUUAACUGGGCUAAGGUCGC	Cleavage	1
trf_1_529	Bra005116	3	13.5	1	21	233	254	GCUGGAGU-AGCUCAGUUGGUU	AACCAACUGAUCUCACUCCAGC	Translation	1
trf_1_460	Bra006584	3	21.1	1	20	1526	1545	GGGAUUGUAGUUCAAUUGUC	GGCAGUUGAUCUACAAUUUC	Translation	1
trf_1_4	Bra007154	2.5	14.1	1	21	510	530	GCACCAGUGGUCUAGUGGUAG	UUACUACUGGACCUUUGGUGC	Cleavage	1
trf_1_7	Bra007154	2.5	14.1	1	21	510	530	GCACCAGUGGUCUAGUGGUAG	UUACUACUGGACCUUUGGUGC	Cleavage	1
trf_1_68	Bra007154	2.5	14.1	1	21	510	530	GCACCAGUGGUCUAGUGGUAG	UUACUACUGGACCUUUGGUGC	Cleavage	1
trf_1_69	Bra007154	2.5	14.1	1	21	510	530	GCACCAGUGGUCUAGUGGUAG	UUACUACUGGACCUUUGGUGC	Cleavage	1
trf_1_86	Bra007154	2.5	14.1	1	21	510	530	GCACCAGUGGUCUAGUGGUAG	UUACUACUGGACCUUUGGUGC	Cleavage	1
trf_1_87	Bra007154	2.5	14.1	1	21	510	530	GCACCAGUGGUCUAGUGGUAG	UUACUACUGGACCUUUGGUGC	Cleavage	1
trf_1_127	Bra007154	2.5	14.1	1	21	510	530	GCACCAGUGGUCUAGUGGUAG	UUACUACUGGACCUUUGGUGC	Cleavage	1
trf_1_141	Bra007154	2.5	14.1	1	21	510	530	GCACCAGUGGUCUAGUGGUAG	UUACUACUGGACCUUUGGUGC	Cleavage	1
trf_1_150	Bra007154	2.5	14.1	1	21	510	530	GCACCAGUGGUCUAGUGGUAG	UUACUACUGGACCUUUGGUGC	Cleavage	1
trf_1_167	Bra007154	2.5	14.1	1	21	510	530	GCACCAGUGGUCUAGUGGUAG	UUACUACUGGACCUUUGGUGC	Cleavage	1
trf_1_250	Bra007154	2.5	14.1	1	21	510	530	GCACCAGUGGUCUAGUGGUAG	UUACUACUGGACCUUUGGUGC	Cleavage	1
trf_1_262	Bra007154	2.5	14.1	1	21	510	530	GCACCAGUGGUCUAGUGGUAG	UUACUACUGGACCUUUGGUGC	Cleavage	1
trf_1_280	Bra007154	2.5	14.1	1	21	510	530	GCACCAGUGGUCUAGUGGUAG	UUACUACUGGACCUUUGGUGC	Cleavage	1

trf_1_329	Bra007154	2.5	14.1	1	21	510	530	GCACCAGUGGUCUAGUGGUAG	UUACUACUGGACCUUUGGUGC	Cleavage	1
trf_1_330	Bra007154	2.5	14.1	1	21	510	530	GCACCAGUGGUCUAGUGGUAG	UUACUACUGGACCUUUGGUGC	Cleavage	1
trf_1_412	Bra007154	2.5	14.1	1	21	510	530	GCACCAGUGGUCUAGUGGUAG	UUACUACUGGACCUUUGGUGC	Cleavage	1
trf_1_480	Bra007154	2.5	14.1	1	21	510	530	GCACCAGUGGUCUAGUGGUAG	UUACUACUGGACCUUUGGUGC	Cleavage	1
trf_1_513	Bra007154	2.5	14.1	1	21	510	530	GCACCAGUGGUCUAGUGGUAG	UUACUACUGGACCUUUGGUGC	Cleavage	1
trf_1_575	Bra007154	2.5	14.1	1	21	510	530	GCACCAGUGGUCUAGUGGUAG	UUACUACUGGACCUUUGGUGC	Cleavage	1
trf_1_464	Bra007277	3	21.1	1	20	926	945	GGGAUUGUAGUUAAAUUGGU	AACAGUUUAACAACAAUCCU	Translation	1
trf_1_271	Bra007620	3	16.3	1	20	292	311	GCGUCCAUUGUCUAAUGGAU	AUCCGUCAGACAAUGGAAGC	Cleavage	1
trf_1_272	Bra007620	3	16.3	1	20	292	311	GCGUCCAUUGUCUAAUGGAU	AUCCGUCAGACAAUGGAAGC	Cleavage	1
trf_1_273	Bra007620	3	16.3	1	20	292	311	GCGUCCAUUGUCUAAUGGAU	AUCCGUCAGACAAUGGAAGC	Cleavage	1
trf_1_274	Bra007620	3	16.3	1	20	292	311	GCGUCCAUUGUCUAAUGGAU	AUCCGUCAGACAAUGGAAGC	Cleavage	1
trf_1_275	Bra007620	3	16.3	1	20	292	311	GCGUCCAUUGUCUAAUGGAU	AUCCGUCAGACAAUGGAAGC	Cleavage	1
trf_1_276	Bra007620	3	16.3	1	20	292	311	GCGUCCAUUGUCUAAUGGAU	AUCCGUCAGACAAUGGAAGC	Cleavage	1
trf_1_278	Bra007620	3	16.3	1	20	292	311	GCGUCCAUUGUCUAAUGGAU	AUCCGUCAGACAAUGGAAGC	Cleavage	1
trf_1_279	Bra007620	3	16.3	1	20	292	311	GCGUCCAUUGUCUAAUGGAU	AUCCGUCAGACAAUGGAAGC	Cleavage	1
trf_1_577	Bra008706	3	14.7	1	20	1321	1340	UUAGGUUCAAAUCCUAUUGG	UCGAUGGGGAUUGAACCUAA	Translation	1
trf_1_182	Bra009057	3	16.0	1	20	737	756	CCGACCUUAGCUCAGUUGGU	UCAAACUGAGCCAAGGUCGG	Translation	1

trf_1_476	Bra009057	3	16.0	1	20	737	756	CCGACCUUAGCUCAGUUGGU	UCAAACUGAGCCAAGGUCGG	Translation	1
trf_1_478	Bra009057	3	16.0	1	20	737	756	CCGACCUUAGCUCAGUUGGU	UCAAACUGAGCCAAGGUCGG	Translation	1
trf_1_479	Bra009057	3	16.0	1	20	737	756	CCGACCUUAGCUCAGUUGGU	UCAAACUGAGCCAAGGUCGG	Translation	1
trf_1_520	Bra009057	3	16.0	1	20	737	756	CCGACCUUAGCUCAGUUGGU	UCAAACUGAGCCAAGGUCGG	Translation	1
trf_1_425	Bra009115	3	15.2	1	20	602	621	UGGAUUGUAGUUCAAUUGGU	CUCAGUUGAACUCAAUCCA	Cleavage	1
trf_1_433	Bra009115	3	15.2	1	20	602	621	GGGAUUGUAGUUCAAUUGGG	CUCAGUUGAACUUCAAUCCA	Cleavage	1
trf_1_441	Bra009115	3	15.2	1	20	602	621	GGGAUUGUAGUUCAAUUGGG	CUCAGUUGAACUUCAAUCCA	Cleavage	1
trf_1_442	Bra009115	3	15.2	1	20	602	621	GGGAUUGUAGUUCAAUUGGG	CUCAGUUGAACUUCAAUCCA	Cleavage	1
trf_1_462	Bra009115	3	15.2	1	20	602	621	UGGAUUGUAGUUCAAUUGGU	CUCAGUUGAACUUCAAUCCA	Cleavage	1
trf_1_466	Bra009115	3	15.2	1	20	602	621	UGGAUUGUAGUUCAAUUGGU	CUCAGUUGAACUUCAAUCCA	Cleavage	1
trf_1_580	Bra009190	2.5	18.3	1	22	559	580	GGUUCAAAUCCUAUUGGACGCA	UCCGUCCAAGAGGAUUUCAACC	Cleavage	1
trf_1_577	Bra009377	3	21.2	1	25	578	602	UUAGGUUCAAAUCCUAUUGGACGCA	UGCAGUCGAUAGGAUAUGAGCCUGA	Translation	1
trf_1_31	Bra009876	3	14.9	1	22	16	37	GGGGAUGUAGCUCAGAUGGUAG	UUAUCAUCUGGGCUUCGUCCUC	Cleavage	1
trf_1_35	Bra009876	3	14.9	1	20	18	37	GGGGAUGUAGCUCAGAUGGU	AUCAUCUGGGCUUCGUCCUC	Cleavage	1
trf_1_60	Bra009876	3	14.9	1	22	16	37	GGGGAUGUAGCUCAGAUGGUAG	UUAUCAUCUGGGCUUCGUCCUC	Cleavage	1
trf_1_62	Bra009876	3	14.9	1	24	14	37	GGGGAUGUAGCUCAGAUGGUAGAG	CGUUAUCAUCUGGGCUUCGUCCUC	Cleavage	1
trf_1_90	Bra009876	3	14.9	1	22	16	37	GGGGAUGUAGCUCAGAUGGUAG	UUAUCAUCUGGGCUUCGUCCUC	Cleavage	1

trf_1_133	Bra009876	3	14.9	1	22	16	37	GGGGAUGUAGCUCAGAUGGUAG	UUAUCAUCUGGGCUUCGUCCUC	Cleavage	1
trf_1_137	Bra009876	3	14.9	1	20	18	37	GGGGAUGUAGCUCAGAUGGU	AUCAUCUGGGCUUCGUCCUC	Cleavage	1
trf_1_138	Bra009876	3	14.9	1	21	17	37	GGGGAUGUAGCUCAGAUGGUA	UAUCAUCUGGGCUUCGUCCUC	Cleavage	1
trf_1_153	Bra009876	3	14.9	1	22	16	37	GGGGAUGUAGCUCAGAUGGUAG	UUAUCAUCUGGGCUUCGUCCUC	Cleavage	1
trf_1_154	Bra009876	3	14.9	1	24	14	37	GGGGAUGUAGCUCAGAUGGUAGAG	CGUUAUCAUCUGGGCUUCGUCCUC	Cleavage	1
trf_1_160	Bra009876	3	14.9	1	22	16	37	GGGGAUGUAGCUCAGAUGGUAG	UUAUCAUCUGGGCUUCGUCCUC	Cleavage	1
trf_1_162	Bra009876	3	14.9	1	24	14	37	GGGGAUGUAGCUCAGAUGGUAGAG	CGUUAUCAUCUGGGCUUCGUCCUC	Cleavage	1
trf_1_169	Bra009876	3	14.9	1	22	16	37	GGGGAUGUAGCUCAGAUGGUAG	UUAUCAUCUGGGCUUCGUCCUC	Cleavage	1
trf_1_173	Bra009876	3	14.9	1	24	14	37	GGGGAUGUAGCUCAGAUGGUAGAG	CGUUAUCAUCUGGGCUUCGUCCUC	Cleavage	1
trf_1_183	Bra009876	3	14.9	1	22	16	37	GGGGAUGUAGCUCAGAUGGUAG	UUAUCAUCUGGGCUUCGUCCUC	Cleavage	1
trf_1_233	Bra009876	3	14.9	1	22	16	37	GGGGAUGUAGCUCAGAUGGUAG	UUAUCAUCUGGGCUUCGUCCUC	Cleavage	1
trf_1_236	Bra009876	3	14.9	1	24	14	37	GGGGAUGUAGCUCAGAUGGUAGAG	CGUUAUCAUCUGGGCUUCGUCCUC	Cleavage	1
trf_1_244	Bra009876	3	14.9	1	22	16	37	GGGGAUGUAGCUCAGAUGGUAG	UUAUCAUCUGGGCUUCGUCCUC	Cleavage	1
trf_1_247	Bra009876	3	14.9	1	24	14	37	GGGGAUGUAGCUCAGAUGGUAGAG	CGUUAUCAUCUGGGCUUCGUCCUC	Cleavage	1
trf_1_266	Bra009876	3	14.9	1	22	16	37	GGGGAUGUAGCUCAGAUGGUAG	UUAUCAUCUGGGCUUCGUCCUC	Cleavage	1
trf_1_286	Bra009876	3	14.9	1	22	16	37	GGGGAUGUAGCUCAGAUGGUAG	UUAUCAUCUGGGCUUCGUCCUC	Cleavage	1
trf_1_293	Bra009876	3	14.9	1	22	16	37	GGGGAUGUAGCUCAGAUGGUAG	UUAUCAUCUGGGCUUCGUCCUC	Cleavage	1

trf_1_297	Bra009876	3	14.9	1	24	14	37	GGGGAUGUAGCUCAGAUGGUAGAG	CGUUAUCAUCUGGGCUUCGUCCUC	Cleavage	1
trf_1_302	Bra009876	3	14.9	1	22	16	37	GGGGAUGUAGCUCAGAUGGUAG	UUAUCAUCUGGGCUUCGUCCUC	Cleavage	1
trf_1_307	Bra009876	3	14.9	1	22	16	37	GGGGAUGUAGCUCAGAUGGUAG	UUAUCAUCUGGGCUUCGUCCUC	Cleavage	1
trf_1_314	Bra009876	3	14.9	1	22	16	37	GGGGAUGUAGCUCAGAUGGUAG	UUAUCAUCUGGGCUUCGUCCUC	Cleavage	1
trf_1_322	Bra009876	3	14.9	1	22	16	37	GGGGAUGUAGCUCAGAUGGUAG	UUAUCAUCUGGGCUUCGUCCUC	Cleavage	1
trf_1_323	Bra009876	3	14.9	1	24	14	37	GGGGAUGUAGCUCAGAUGGUAGAG	CGUUAUCAUCUGGGCUUCGUCCUC	Cleavage	1
trf_1_332	Bra009876	3	14.9	1	22	16	37	GGGGAUGUAGCUCAGAUGGUAG	UUAUCAUCUGGGCUUCGUCCUC	Cleavage	1
trf_1_338	Bra009876	3	14.9	1	24	14	37	GGGGAUGUAGCUCAGAUGGUAGAG	CGUUAUCAUCUGGGCUUCGUCCUC	Cleavage	1
trf_1_341	Bra009876	3	14.9	1	22	16	37	GGGGAUGUAGCUCAGAUGGUAG	UUAUCAUCUGGGCUUCGUCCUC	Cleavage	1
trf_1_351	Bra009876	3	14.9	1	22	16	37	GGGGAUGUAGCUCAGAUGGUAG	UUAUCAUCUGGGCUUCGUCCUC	Cleavage	1
trf_1_353	Bra009876	3	14.9	1	20	18	37	GGGGAUGUAGCUCAGAUGGU	AUCAUCUGGGCUUCGUCCUC	Cleavage	1
trf_1_362	Bra009876	3	14.9	1	22	16	37	GGGGAUGUAGCUCAGAUGGUAG	UUAUCAUCUGGGCUUCGUCCUC	Cleavage	1
trf_1_366	Bra009876	3	14.9	1	24	14	37	GGGGAUGUAGCUCAGAUGGUAGAG	CGUUAUCAUCUGGGCUUCGUCCUC	Cleavage	1
trf_1_375	Bra009876	3	14.9	1	22	16	37	GGGGAUGUAGCUCAGAUGGUAG	UUAUCAUCUGGGCUUCGUCCUC	Cleavage	1
trf_1_379	Bra009876	3	14.9	1	24	14	37	GGGGAUGUAGCUCAGAUGGUAGAG	CGUUAUCAUCUGGGCUUCGUCCUC	Cleavage	1
trf_1_382	Bra009876	3	14.9	1	22	16	37	GGGGAUGUAGCUCAGAUGGUAG	UUAUCAUCUGGGCUUCGUCCUC	Cleavage	1
trf_1_383	Bra009876	3	14.9	1	24	14	37	GGGGAUGUAGCUCAGAUGGUAGAG	CGUUAUCAUCUGGGCUUCGUCCUC	Cleavage	1

trf_1_391	Bra009876	3	14.9	1	22	16	37	GGGGAUGUAGCUCAGAUGGUAG	UUAUCAUCUGGGCUUCGUCCUC	Cleavage	1
trf_1_392	Bra009876	3	14.9	1	20	18	37	GGGGAUGUAGCUCAGAUGGU	AUCAUCUGGGCUUCGUCCUC	Cleavage	1
trf_1_393	Bra009876	3	14.9	1	24	14	37	GGGGAUGUAGCUCAGAUGGUAGAG	CGUUAUCAUCUGGGCUUCGUCCUC	Cleavage	1
trf_1_400	Bra009876	3	14.9	1	22	16	37	GGGGAUGUAGCUCAGAUGGUAG	UUAUCAUCUGGGCUUCGUCCUC	Cleavage	1
trf_1_404	Bra009876	3	14.9	1	20	18	37	GGGGAUGUAGCUCAGAUGGU	AUCAUCUGGGCUUCGUCCUC	Cleavage	1
trf_1_408	Bra009876	3	14.9	1	22	16	37	GGGGAUGUAGCUCAGAUGGUAG	UUAUCAUCUGGGCUUCGUCCUC	Cleavage	1
trf_1_410	Bra009876	3	14.9	1	20	18	37	GGGGAUGUAGCUCAGAUGGU	AUCAUCUGGGCUUCGUCCUC	Cleavage	1
trf_1_482	Bra009876	3	14.9	1	22	16	37	GGGGAUGUAGCUCAGAUGGUAG	UUAUCAUCUGGGCUUCGUCCUC	Cleavage	1
trf_1_486	Bra009876	3	14.9	1	20	18	37	GGGGAUGUAGCUCAGAUGGU	AUCAUCUGGGCUUCGUCCUC	Cleavage	1
trf_1_489	Bra009876	3	14.9	1	22	16	37	GGGGAUGUAGCUCAGAUGGUAG	UUAUCAUCUGGGCUUCGUCCUC	Cleavage	1
trf_1_494	Bra009876	3	14.9	1	20	18	37	GGGGAUGUAGCUCAGAUGGU	AUCAUCUGGGCUUCGUCCUC	Cleavage	1
trf_1_498	Bra009876	3	14.9	1	20	18	37	GGGGAUGUAGCUCAGAUGGU	AUCAUCUGGGCUUCGUCCUC	Cleavage	1
trf_1_499	Bra009876	3	14.9	1	22	16	37	GGGGAUGUAGCUCAGAUGGUAG	UUAUCAUCUGGGCUUCGUCCUC	Cleavage	1
trf_1_32	Bra010298	3	19.9	1	25	1891	1916	GGGGAUAUAGCUCAGUUGGU-AGAGC	GUUCUCACCAGCAGAGCUAUAUUCCA	Cleavage	1
trf_1_33	Bra010298	3	19.9	1	20	1897	1916	GGGGAUAUAGCUCAGUUGGU	ACCAGCAGAGCUAUAUUCCA	Cleavage	1
trf_1_34	Bra010298	3	19.9	1	24	1892	1916	GGGGAUAUAGCUCAGUUGGU-AGAG	UUCUCACCAGCAGAGCUAUAUUCCA	Cleavage	1
trf_1_36	Bra010298	3	19.9	1	20	1897	1916	GGGGAUAUAGCUCAGUUGGU	ACCAGCAGAGCUAUAUUCCA	Cleavage	1

trf_1_59	Bra010298	3	19.9	1	20	1897	1916	GGGGAUAUAGCUCAGUUGGU	ACCAGCAGAGCUAUAUUCCA	Cleavage	1
trf_1_61	Bra010298	3	19.9	1	25	1891	1916	GGGGAUAUAGCUCAGUUGGU-AGAGC	GUUCUCACCAGCAGAGCUAUAUUCCA	Cleavage	1
trf_1_63	Bra010298	3	19.9	1	20	1897	1916	GGGGAUAUAGCUCAGUUGGU	ACCAGCAGAGCUAUAUUCCA	Cleavage	1
trf_1_64	Bra010298	3	19.9	1	20	1897	1916	GGGGAUAUAGCUCAGUUGGU	ACCAGCAGAGCUAUAUUCCA	Cleavage	1
trf_1_89	Bra010298	3	19.9	1	20	1897	1916	GGGGAUAUAGCUCAGUUGGU	ACCAGCAGAGCUAUAUUCCA	Cleavage	1
trf_1_91	Bra010298	3	19.9	1	25	1891	1916	GGGGAUAUAGCUCAGUUGGU-AGAGC	GUUCUCACCAGCAGAGCUAUAUUCCA	Cleavage	1
trf_1_92	Bra010298	3	19.9	1	20	1897	1916	GGGGAUAUAGCUCAGUUGGU	ACCAGCAGAGCUAUAUUCCA	Cleavage	1
trf_1_93	Bra010298	3	19.9	1	20	1897	1916	GGGGAUAUAGCUCAGUUGGU	ACCAGCAGAGCUAUAUUCCA	Cleavage	1
trf_1_134	Bra010298	3	19.9	1	20	1897	1916	GGGGAUAUAGCUCAGUUGGU	ACCAGCAGAGCUAUAUUCCA	Cleavage	1
trf_1_135	Bra010298	3	19.9	1	20	1897	1916	GGGGAUAUAGCUCAGUUGGU	ACCAGCAGAGCUAUAUUCCA	Cleavage	1
trf_1_136	Bra010298	3	19.9	1	25	1891	1916	GGGGAUAUAGCUCAGUUGGU-AGAGC	GUUCUCACCAGCAGAGCUAUAUUCCA	Cleavage	1
trf_1_139	Bra010298	3	19.9	1	20	1897	1916	GGGGAUAUAGCUCAGUUGGU	ACCAGCAGAGCUAUAUUCCA	Cleavage	1
trf_1_151	Bra010298	3	19.9	1	20	1897	1916	GGGGAUAUAGCUCAGUUGGU	ACCAGCAGAGCUAUAUUCCA	Cleavage	1
trf_1_152	Bra010298	3	19.9	1	20	1897	1916	GGGGAUAUAGCUCAGUUGGU	ACCAGCAGAGCUAUAUUCCA	Cleavage	1
trf_1_155	Bra010298	3	19.9	1	20	1897	1916	GGGGAUAUAGCUCAGUUGGU	ACCAGCAGAGCUAUAUUCCA	Cleavage	1
trf_1_156	Bra010298	3	19.9	1	25	1891	1916	GGGGAUAUAGCUCAGUUGGU-AGAGC	GUUCUCACCAGCAGAGCUAUAUUCCA	Cleavage	1
trf_1_161	Bra010298	3	19.9	1	25	1891	1916	GGGGAUAUAGCUCAGUUGGU-AGAGC	GUUCUCACCAGCAGAGCUAUAUUCCA	Cleavage	1

trf_1_1<br>63	Bra010<br>298	3	19.<br>9	1	24	1892	1916	GGGGAUAUAGCUCAGU<br>UGGU-AGAG	UUCUCACCAGCAGAGCU<br>AUAUUCCA	Cleava<br>ge	1
trf_1_1<br>64	Bra010<br>298	3	19.<br>9	1	20	1897	1916	GGGGAUAUAGCUCAGU<br>UGGU	ACCAGCAGAGCUAUAU<br>UCCA	Cleava<br>ge	1
trf_1_1<br>65	Bra010<br>298	3	19.<br>9	1	20	1897	1916	GGGGAUAUAGCUCAGU<br>UGGU	ACCAGCAGAGCUAUAU<br>UCCA	Cleava<br>ge	1
trf_1_1<br>66	Bra010<br>298	3	19.<br>9	1	20	1897	1916	GGGGAUAUAGCUCAGU<br>UGGU	ACCAGCAGAGCUAUAU<br>UCCA	Cleava<br>ge	1
trf_1_1<br>68	Bra010<br>298	3	19.<br>9	1	25	1891	1916	GGGGAUAUAGCUCAGU<br>UGGU-AGAGC	GUUCUCACCAGCAGAGC<br>UAUAUUCCA	Cleava<br>ge	1
trf_1_1<br>70	Bra010<br>298	3	19.<br>9	1	20	1897	1916	GGGGAUAUAGCUCAGU<br>UGGU	ACCAGCAGAGCUAUAU<br>UCCA	Cleava<br>ge	1
trf_1_1<br>71	Bra010<br>298	3	19.<br>9	1	20	1897	1916	GGGGAUAUAGCUCAGU<br>UGGU	ACCAGCAGAGCUAUAU<br>UCCA	Cleava<br>ge	1
trf_1_1<br>72	Bra010<br>298	3	19.<br>9	1	20	1897	1916	GGGGAUAUAGCUCAGU<br>UGGU	ACCAGCAGAGCUAUAU<br>UCCA	Cleava<br>ge	1
trf_1_1<br>84	Bra010<br>298	3	19.<br>9	1	25	1891	1916	GGGGAUAUAGCUCAGU<br>UGGU-AGAGC	GUUCUCACCAGCAGAGC<br>UAUAUUCCA	Cleava<br>ge	1
trf_1_1<br>85	Bra010<br>298	3	19.<br>9	1	20	1897	1916	GGGGAUAUAGCUCAGU<br>UGGU	ACCAGCAGAGCUAUAU<br>UCCA	Cleava<br>ge	1
trf_1_1<br>86	Bra010<br>298	3	19.<br>9	1	20	1897	1916	GGGGAUAUAGCUCAGU<br>UGGU	ACCAGCAGAGCUAUAU<br>UCCA	Cleava<br>ge	1
trf_1_1<br>87	Bra010<br>298	3	19.<br>9	1	20	1897	1916	GGGGAUAUAGCUCAGU<br>UGGU	ACCAGCAGAGCUAUAU<br>UCCA	Cleava<br>ge	1
trf_1_2<br>34	Bra010<br>298	3	19.<br>9	1	20	1897	1916	GGGGAUAUAGCUCAGU<br>UGGU	ACCAGCAGAGCUAUAU<br>UCCA	Cleava<br>ge	1
trf_1_2<br>35	Bra010<br>298	3	19.<br>9	1	20	1897	1916	GGGGAUAUAGCUCAGU<br>UGGU	ACCAGCAGAGCUAUAU<br>UCCA	Cleava<br>ge	1
trf_1_2<br>37	Bra010<br>298	3	19.<br>9	1	24	1892	1916	GGGGAUAUAGCUCAGU<br>UGGU-AGAG	UUCUCACCAGCAGAGCU<br>AUAUUCCA	Cleava<br>ge	1
trf_1_2<br>45	Bra010<br>298	3	19.<br>9	1	20	1897	1916	GGGGAUAUAGCUCAGU<br>UGGU	ACCAGCAGAGCUAUAU<br>UCCA	Cleava<br>ge	1
trf_1_2<br>46	Bra010<br>298	3	19.<br>9	1	20	1897	1916	GGGGAUAUAGCUCAGU<br>UGGU	ACCAGCAGAGCUAUAU<br>UCCA	Cleava<br>ge	1

trf_1_248	Bra010298	3	19.9	1	25	1891	1916	GGGGAUAUAGCUCAGUUGGU-AGAGC	GUUCUCACCAGCAGAGCUAUAUUCCA	Cleavage	1
trf_1_265	Bra010298	3	19.9	1	20	1897	1916	GGGGAUAUAGCUCAGUUGGU	ACCAGCAGAGCUAUAUUCCA	Cleavage	1
trf_1_267	Bra010298	3	19.9	1	25	1891	1916	GGGGAUAUAGCUCAGUUGGU-AGAGC	GUUCUCACCAGCAGAGCUAUAUUCCA	Cleavage	1
trf_1_268	Bra010298	3	19.9	1	20	1897	1916	GGGGAUAUAGCUCAGUUGGU	ACCAGCAGAGCUAUAUUCCA	Cleavage	1
trf_1_269	Bra010298	3	19.9	1	20	1897	1916	GGGGAUAUAGCUCAGUUGGU	ACCAGCAGAGCUAUAUUCCA	Cleavage	1
trf_1_270	Bra010298	3	19.9	1	24	1892	1916	GGGGAUAUAGCUCAGUUGGU-AGAG	UUCUCACCAGCAGAGCUAUAUUCCA	Cleavage	1
trf_1_283	Bra010298	3	19.9	1	20	1897	1916	GGGGAUAUAGCUCAGUUGGU	ACCAGCAGAGCUAUAUUCCA	Cleavage	1
trf_1_284	Bra010298	3	19.9	1	25	1891	1916	GGGGAUAUAGCUCAGUUGGU-AGAGC	GUUCUCACCAGCAGAGCUAUAUUCCA	Cleavage	1
trf_1_285	Bra010298	3	19.9	1	20	1897	1916	GGGGAUAUAGCUCAGUUGGU	ACCAGCAGAGCUAUAUUCCA	Cleavage	1
trf_1_294	Bra010298	3	19.9	1	20	1897	1916	GGGGAUAUAGCUCAGUUGGU	ACCAGCAGAGCUAUAUUCCA	Cleavage	1
trf_1_295	Bra010298	3	19.9	1	25	1891	1916	GGGGAUAUAGCUCAGUUGGU-AGAGC	GUUCUCACCAGCAGAGCUAUAUUCCA	Cleavage	1
trf_1_296	Bra010298	3	19.9	1	20	1897	1916	GGGGAUAUAGCUCAGUUGGU	ACCAGCAGAGCUAUAUUCCA	Cleavage	1
trf_1_298	Bra010298	3	19.9	1	24	1892	1916	GGGGAUAUAGCUCAGUUGGU-AGAG	UUCUCACCAGCAGAGCUAUAUUCCA	Cleavage	1
trf_1_301	Bra010298	3	19.9	1	20	1897	1916	GGGGAUAUAGCUCAGUUGGU	ACCAGCAGAGCUAUAUUCCA	Cleavage	1
trf_1_303	Bra010298	3	19.9	1	25	1891	1916	GGGGAUAUAGCUCAGUUGGU-AGAGC	GUUCUCACCAGCAGAGCUAUAUUCCA	Cleavage	1
trf_1_304	Bra010298	3	19.9	1	20	1897	1916	GGGGAUAUAGCUCAGUUGGU	ACCAGCAGAGCUAUAUUCCA	Cleavage	1
trf_1_305	Bra010298	3	19.9	1	20	1897	1916	GGGGAUAUAGCUCAGUUGGU	ACCAGCAGAGCUAUAUUCCA	Cleavage	1

trf_1_308	Bra010298	3	19.9	1	20	1897	1916	GGGGAUAUAGCUCAGUUGGU	ACCAGCAGAGCUAUAUUCCA	Cleavage	1
trf_1_309	Bra010298	3	19.9	1	25	1891	1916	GGGGAUAUAGCUCAGUUGGU-AGAGC	GUUCUCACCAGCAGAGCUAUAUUCCA	Cleavage	1
trf_1_310	Bra010298	3	19.9	1	20	1897	1916	GGGGAUAUAGCUCAGUUGGU	ACCAGCAGAGCUAUAUUCCA	Cleavage	1
trf_1_315	Bra010298	3	19.9	1	24	1892	1916	GGGGAUAUAGCUCAGUUGGU-AGAG	UUCUCACCAGCAGAGCUAUAUUCCA	Cleavage	1
trf_1_316	Bra010298	3	19.9	1	25	1891	1916	GGGGAUAUAGCUCAGUUGGU-AGAGC	GUUCUCACCAGCAGAGCUAUAUUCCA	Cleavage	1
trf_1_317	Bra010298	3	19.9	1	20	1897	1916	GGGGAUAUAGCUCAGUUGGU	ACCAGCAGAGCUAUAUUCCA	Cleavage	1
trf_1_318	Bra010298	3	19.9	1	20	1897	1916	GGGGAUAUAGCUCAGUUGGU	ACCAGCAGAGCUAUAUUCCA	Cleavage	1
trf_1_321	Bra010298	3	19.9	1	25	1891	1916	GGGGAUAUAGCUCAGUUGGU-AGAGC	GUUCUCACCAGCAGAGCUAUAUUCCA	Cleavage	1
trf_1_324	Bra010298	3	19.9	1	20	1897	1916	GGGGAUAUAGCUCAGUUGGU	ACCAGCAGAGCUAUAUUCCA	Cleavage	1
trf_1_325	Bra010298	3	19.9	1	20	1897	1916	GGGGAUAUAGCUCAGUUGGU	ACCAGCAGAGCUAUAUUCCA	Cleavage	1
trf_1_333	Bra010298	3	19.9	1	20	1897	1916	GGGGAUAUAGCUCAGUUGGU	ACCAGCAGAGCUAUAUUCCA	Cleavage	1
trf_1_334	Bra010298	3	19.9	1	20	1897	1916	GGGGAUAUAGCUCAGUUGGU	ACCAGCAGAGCUAUAUUCCA	Cleavage	1
trf_1_335	Bra010298	3	19.9	1	25	1891	1916	GGGGAUAUAGCUCAGUUGGU-AGAGC	GUUCUCACCAGCAGAGCUAUAUUCCA	Cleavage	1
trf_1_339	Bra010298	3	19.9	1	20	1897	1916	GGGGAUAUAGCUCAGUUGGU	ACCAGCAGAGCUAUAUUCCA	Cleavage	1
trf_1_340	Bra010298	3	19.9	1	25	1891	1916	GGGGAUAUAGCUCAGUUGGU-AGAGC	GUUCUCACCAGCAGAGCUAUAUUCCA	Cleavage	1
trf_1_342	Bra010298	3	19.9	1	20	1897	1916	GGGGAUAUAGCUCAGUUGGU	ACCAGCAGAGCUAUAUUCCA	Cleavage	1
trf_1_343	Bra010298	3	19.9	1	20	1897	1916	GGGGAUAUAGCUCAGUUGGU	ACCAGCAGAGCUAUAUUCCA	Cleavage	1

trf_1_344	Bra010298	3	19.9	1	20	1897	1916	GGGGAUAUAGCUCAGUUGGU	ACCAGCAGAGCUAUAUUCCA	Cleavage	1
trf_1_352	Bra010298	3	19.9	1	20	1897	1916	GGGGAUAUAGCUCAGUUGGU	ACCAGCAGAGCUAUAUUCCA	Cleavage	1
trf_1_354	Bra010298	3	19.9	1	20	1897	1916	GGGGAUAUAGCUCAGUUGGU	ACCAGCAGAGCUAUAUUCCA	Cleavage	1
trf_1_355	Bra010298	3	19.9	1	20	1897	1916	GGGGAUAUAGCUCAGUUGGU	ACCAGCAGAGCUAUAUUCCA	Cleavage	1
trf_1_356	Bra010298	3	19.9	1	20	1897	1916	GGGGAUAUAGCUCAGUUGGU	ACCAGCAGAGCUAUAUUCCA	Cleavage	1
trf_1_357	Bra010298	3	19.9	1	25	1891	1916	GGGGAUAUAGCUCAGUUGGU-AGAGC	GUUCUCACCAGCAGAGCUAUAUUCCA	Cleavage	1
trf_1_358	Bra010298	3	19.9	1	24	1892	1916	GGGGAUAUAGCUCAGUUGGU-AGAG	UUCUCACCAGCAGAGCUAUAUUCCA	Cleavage	1
trf_1_361	Bra010298	3	19.9	1	20	1897	1916	GGGGAUAUAGCUCAGUUGGU	ACCAGCAGAGCUAUAUUCCA	Cleavage	1
trf_1_363	Bra010298	3	19.9	1	20	1897	1916	GGGGAUAUAGCUCAGUUGGU	ACCAGCAGAGCUAUAUUCCA	Cleavage	1
trf_1_364	Bra010298	3	19.9	1	25	1891	1916	GGGGAUAUAGCUCAGUUGGU-AGAGC	GUUCUCACCAGCAGAGCUAUAUUCCA	Cleavage	1
trf_1_365	Bra010298	3	19.9	1	20	1897	1916	GGGGAUAUAGCUCAGUUGGU	ACCAGCAGAGCUAUAUUCCA	Cleavage	1
trf_1_376	Bra010298	3	19.9	1	20	1897	1916	GGGGAUAUAGCUCAGUUGGU	ACCAGCAGAGCUAUAUUCCA	Cleavage	1
trf_1_377	Bra010298	3	19.9	1	20	1897	1916	GGGGAUAUAGCUCAGUUGGU	ACCAGCAGAGCUAUAUUCCA	Cleavage	1
trf_1_378	Bra010298	3	19.9	1	25	1891	1916	GGGGAUAUAGCUCAGUUGGU-AGAGC	GUUCUCACCAGCAGAGCUAUAUUCCA	Cleavage	1
trf_1_380	Bra010298	3	19.9	1	20	1897	1916	GGGGAUAUAGCUCAGUUGGU	ACCAGCAGAGCUAUAUUCCA	Cleavage	1
trf_1_384	Bra010298	3	19.9	1	20	1897	1916	GGGGAUAUAGCUCAGUUGGU	ACCAGCAGAGCUAUAUUCCA	Cleavage	1
trf_1_385	Bra010298	3	19.9	1	20	1897	1916	GGGGAUAUAGCUCAGUUGGU	ACCAGCAGAGCUAUAUUCCA	Cleavage	1

trf_1_386	Bra010298	3	19.9	1	25	1891	1916	GGGGAUAUAGCUCAGUUGGU-AGAGC	GUUCUCACCAGCAGAGCUAUAUUCCA	Cleavage	1
trf_1_387	Bra010298	3	19.9	1	20	1897	1916	GGGGAUAUAGCUCAGUUGGU	ACCAGCAGAGCUAUAUUCCA	Cleavage	1
trf_1_394	Bra010298	3	19.9	1	20	1897	1916	GGGGAUAUAGCUCAGUUGGU	ACCAGCAGAGCUAUAUUCCA	Cleavage	1
trf_1_395	Bra010298	3	19.9	1	25	1891	1916	GGGGAUAUAGCUCAGUUGGU-AGAGC	GUUCUCACCAGCAGAGCUAUAUUCCA	Cleavage	1
trf_1_396	Bra010298	3	19.9	1	20	1897	1916	GGGGAUAUAGCUCAGUUGGU	ACCAGCAGAGCUAUAUUCCA	Cleavage	1
trf_1_397	Bra010298	3	19.9	1	20	1897	1916	GGGGAUAUAGCUCAGUUGGU	ACCAGCAGAGCUAUAUUCCA	Cleavage	1
trf_1_401	Bra010298	3	19.9	1	25	1891	1916	GGGGAUAUAGCUCAGUUGGU-AGAGC	GUUCUCACCAGCAGAGCUAUAUUCCA	Cleavage	1
trf_1_402	Bra010298	3	19.9	1	20	1897	1916	GGGGAUAUAGCUCAGUUGGU	ACCAGCAGAGCUAUAUUCCA	Cleavage	1
trf_1_403	Bra010298	3	19.9	1	20	1897	1916	GGGGAUAUAGCUCAGUUGGU	ACCAGCAGAGCUAUAUUCCA	Cleavage	1
trf_1_405	Bra010298	3	19.9	1	24	1892	1916	GGGGAUAUAGCUCAGUUGGU-AGAG	UUCUCACCAGCAGAGCUAUAUUCCA	Cleavage	1
trf_1_406	Bra010298	3	19.9	1	20	1897	1916	GGGGAUAUAGCUCAGUUGGU	ACCAGCAGAGCUAUAUUCCA	Cleavage	1
trf_1_407	Bra010298	3	19.9	1	20	1897	1916	GGGGAUAUAGCUCAGUUGGU	ACCAGCAGAGCUAUAUUCCA	Cleavage	1
trf_1_409	Bra010298	3	19.9	1	25	1891	1916	GGGGAUAUAGCUCAGUUGGU-AGAGC	GUUCUCACCAGCAGAGCUAUAUUCCA	Cleavage	1
trf_1_411	Bra010298	3	19.9	1	20	1897	1916	GGGGAUAUAGCUCAGUUGGU	ACCAGCAGAGCUAUAUUCCA	Cleavage	1
trf_1_483	Bra010298	3	19.9	1	20	1897	1916	GGGGAUAUAGCUCAGUUGGU	ACCAGCAGAGCUAUAUUCCA	Cleavage	1
trf_1_484	Bra010298	3	19.9	1	20	1897	1916	GGGGAUAUAGCUCAGUUGGU	ACCAGCAGAGCUAUAUUCCA	Cleavage	1
trf_1_485	Bra010298	3	19.9	1	20	1897	1916	GGGGAUAUAGCUCAGUUGGU	ACCAGCAGAGCUAUAUUCCA	Cleavage	1

trf_1_487	Bra010298	3	19.9	1	25	1891	1916	GGGGAUAUAGCUCAGUUGGU-AGAGC	GUUCUCACCAGCAGAGCUAUAUUCCA	Cleavage	1
trf_1_488	Bra010298	3	19.9	1	20	1897	1916	GGGGAUAUAGCUCAGUUGGU	ACCAGCAGAGCUAUAUUCCA	Cleavage	1
trf_1_490	Bra010298	3	19.9	1	20	1897	1916	GGGGAUAUAGCUCAGUUGGU	ACCAGCAGAGCUAUAUUCCA	Cleavage	1
trf_1_491	Bra010298	3	19.9	1	25	1891	1916	GGGGAUAUAGCUCAGUUGGU-AGAGC	GUUCUCACCAGCAGAGCUAUAUUCCA	Cleavage	1
trf_1_492	Bra010298	3	19.9	1	20	1897	1916	GGGGAUAUAGCUCAGUUGGU	ACCAGCAGAGCUAUAUUCCA	Cleavage	1
trf_1_493	Bra010298	3	19.9	1	20	1897	1916	GGGGAUAUAGCUCAGUUGGU	ACCAGCAGAGCUAUAUUCCA	Cleavage	1
trf_1_495	Bra010298	3	19.9	1	24	1892	1916	GGGGAUAUAGCUCAGUUGGU-AGAG	UUCUCACCAGCAGAGCUAUAUUCCA	Cleavage	1
trf_1_500	Bra010298	3	19.9	1	20	1897	1916	GGGGAUAUAGCUCAGUUGGU	ACCAGCAGAGCUAUAUUCCA	Cleavage	1
trf_1_501	Bra010298	3	19.9	1	24	1892	1916	GGGGAUAUAGCUCAGUUGGU-AGAG	UUCUCACCAGCAGAGCUAUAUUCCA	Cleavage	1
trf_1_502	Bra010298	3	19.9	1	20	1897	1916	GGGGAUAUAGCUCAGUUGGU	ACCAGCAGAGCUAUAUUCCA	Cleavage	1
trf_1_503	Bra010298	3	19.9	1	25	1891	1916	GGGGAUAUAGCUCAGUUGGU-AGAGC	GUUCUCACCAGCAGAGCUAUAUUCCA	Cleavage	1
trf_1_504	Bra010298	3	19.9	1	20	1897	1916	GGGGAUAUAGCUCAGUUGGU	ACCAGCAGAGCUAUAUUCCA	Cleavage	1
trf_1_32	Bra010299	3	19.9	1	25	1867	1892	GGGGAUAUAGCUCAGUUGGU-AGAGC	GUUCUCACCAGCAGAGCUAUAUUCCA	Cleavage	1
trf_1_33	Bra010299	3	19.9	1	20	1873	1892	GGGGAUAUAGCUCAGUUGGU	ACCAGCAGAGCUAUAUUCCA	Cleavage	1
trf_1_34	Bra010299	3	19.9	1	24	1868	1892	GGGGAUAUAGCUCAGUUGGU-AGAG	UUCUCACCAGCAGAGCUAUAUUCCA	Cleavage	1
trf_1_36	Bra010299	3	19.9	1	20	1873	1892	GGGGAUAUAGCUCAGUUGGU	ACCAGCAGAGCUAUAUUCCA	Cleavage	1
trf_1_59	Bra010299	3	19.9	1	20	1873	1892	GGGGAUAUAGCUCAGUUGGU	ACCAGCAGAGCUAUAUUCCA	Cleavage	1

trf_1_61	Bra010299	3	19.9	1	25	1867	1892	GGGGAUAUAGCUCAGUUGGU-AGAGC	GUUCUCACCAGCAGAGCUAUAUUCCA	Cleavage	1
trf_1_63	Bra010299	3	19.9	1	20	1873	1892	GGGGAUAUAGCUCAGUUGGU	ACCAGCAGAGCUAUAUUCCA	Cleavage	1
trf_1_64	Bra010299	3	19.9	1	20	1873	1892	GGGGAUAUAGCUCAGUUGGU	ACCAGCAGAGCUAUAUUCCA	Cleavage	1
trf_1_89	Bra010299	3	19.9	1	20	1873	1892	GGGGAUAUAGCUCAGUUGGU	ACCAGCAGAGCUAUAUUCCA	Cleavage	1
trf_1_91	Bra010299	3	19.9	1	25	1867	1892	GGGGAUAUAGCUCAGUUGGU-AGAGC	GUUCUCACCAGCAGAGCUAUAUUCCA	Cleavage	1
trf_1_92	Bra010299	3	19.9	1	20	1873	1892	GGGGAUAUAGCUCAGUUGGU	ACCAGCAGAGCUAUAUUCCA	Cleavage	1
trf_1_93	Bra010299	3	19.9	1	20	1873	1892	GGGGAUAUAGCUCAGUUGGU	ACCAGCAGAGCUAUAUUCCA	Cleavage	1
trf_1_134	Bra010299	3	19.9	1	20	1873	1892	GGGGAUAUAGCUCAGUUGGU	ACCAGCAGAGCUAUAUUCCA	Cleavage	1
trf_1_135	Bra010299	3	19.9	1	20	1873	1892	GGGGAUAUAGCUCAGUUGGU	ACCAGCAGAGCUAUAUUCCA	Cleavage	1
trf_1_136	Bra010299	3	19.9	1	25	1867	1892	GGGGAUAUAGCUCAGUUGGU-AGAGC	GUUCUCACCAGCAGAGCUAUAUUCCA	Cleavage	1
trf_1_139	Bra010299	3	19.9	1	20	1873	1892	GGGGAUAUAGCUCAGUUGGU	ACCAGCAGAGCUAUAUUCCA	Cleavage	1
trf_1_151	Bra010299	3	19.9	1	20	1873	1892	GGGGAUAUAGCUCAGUUGGU	ACCAGCAGAGCUAUAUUCCA	Cleavage	1
trf_1_152	Bra010299	3	19.9	1	20	1873	1892	GGGGAUAUAGCUCAGUUGGU	ACCAGCAGAGCUAUAUUCCA	Cleavage	1
trf_1_155	Bra010299	3	19.9	1	20	1873	1892	GGGGAUAUAGCUCAGUUGGU	ACCAGCAGAGCUAUAUUCCA	Cleavage	1
trf_1_156	Bra010299	3	19.9	1	25	1867	1892	GGGGAUAUAGCUCAGUUGGU-AGAGC	GUUCUCACCAGCAGAGCUAUAUUCCA	Cleavage	1
trf_1_161	Bra010299	3	19.9	1	25	1867	1892	GGGGAUAUAGCUCAGUUGGU-AGAGC	GUUCUCACCAGCAGAGCUAUAUUCCA	Cleavage	1
trf_1_163	Bra010299	3	19.9	1	24	1868	1892	GGGGAUAUAGCUCAGUUGGU-AGAG	UUCUCACCAGCAGAGCUAUAUUCCA	Cleavage	1

trf_1_164	Bra010299	3	19.9	1	20	1873	1892	GGGGAUAUAGCUCAGUUGGU	ACCAGCAGAGCUAUAUUCCA	Cleavage	1
trf_1_165	Bra010299	3	19.9	1	20	1873	1892	GGGGAUAUAGCUCAGUUGGU	ACCAGCAGAGCUAUAUUCCA	Cleavage	1
trf_1_166	Bra010299	3	19.9	1	20	1873	1892	GGGGAUAUAGCUCAGUUGGU	ACCAGCAGAGCUAUAUUCCA	Cleavage	1
trf_1_168	Bra010299	3	19.9	1	25	1867	1892	GGGGAUAUAGCUCAGUUGGU-AGAGC	GUUCUCACCAGCAGAGCUAUAUUCCA	Cleavage	1
trf_1_170	Bra010299	3	19.9	1	20	1873	1892	GGGGAUAUAGCUCAGUUGGU	ACCAGCAGAGCUAUAUUCCA	Cleavage	1
trf_1_171	Bra010299	3	19.9	1	20	1873	1892	GGGGAUAUAGCUCAGUUGGU	ACCAGCAGAGCUAUAUUCCA	Cleavage	1
trf_1_172	Bra010299	3	19.9	1	20	1873	1892	GGGGAUAUAGCUCAGUUGGU	ACCAGCAGAGCUAUAUUCCA	Cleavage	1
trf_1_184	Bra010299	3	19.9	1	25	1867	1892	GGGGAUAUAGCUCAGUUGGU-AGAGC	GUUCUCACCAGCAGAGCUAUAUUCCA	Cleavage	1
trf_1_185	Bra010299	3	19.9	1	20	1873	1892	GGGGAUAUAGCUCAGUUGGU	ACCAGCAGAGCUAUAUUCCA	Cleavage	1
trf_1_186	Bra010299	3	19.9	1	20	1873	1892	GGGGAUAUAGCUCAGUUGGU	ACCAGCAGAGCUAUAUUCCA	Cleavage	1
trf_1_187	Bra010299	3	19.9	1	20	1873	1892	GGGGAUAUAGCUCAGUUGGU	ACCAGCAGAGCUAUAUUCCA	Cleavage	1
trf_1_234	Bra010299	3	19.9	1	20	1873	1892	GGGGAUAUAGCUCAGUUGGU	ACCAGCAGAGCUAUAUUCCA	Cleavage	1
trf_1_235	Bra010299	3	19.9	1	20	1873	1892	GGGGAUAUAGCUCAGUUGGU	ACCAGCAGAGCUAUAUUCCA	Cleavage	1
trf_1_237	Bra010299	3	19.9	1	24	1868	1892	GGGGAUAUAGCUCAGUUGGU-AGAG	UUCUCACCAGCAGAGCUAUAUUCCA	Cleavage	1
trf_1_245	Bra010299	3	19.9	1	20	1873	1892	GGGGAUAUAGCUCAGUUGGU	ACCAGCAGAGCUAUAUUCCA	Cleavage	1
trf_1_246	Bra010299	3	19.9	1	20	1873	1892	GGGGAUAUAGCUCAGUUGGU	ACCAGCAGAGCUAUAUUCCA	Cleavage	1
trf_1_248	Bra010299	3	19.9	1	25	1867	1892	GGGGAUAUAGCUCAGUUGGU-AGAGC	GUUCUCACCAGCAGAGCUAUAUUCCA	Cleavage	1

trf_1_265	Bra010299	3	19.9	1	20	1873	1892	GGGGAUAUAGCUCAGUUGGU	ACCAGCAGAGCUAUAUUCCA	Cleavage	1
trf_1_267	Bra010299	3	19.9	1	25	1867	1892	GGGGAUAUAGCUCAGUUGGU-AGAGC	GUUCUCACCAGCAGAGCUAUAUUCCA	Cleavage	1
trf_1_268	Bra010299	3	19.9	1	20	1873	1892	GGGGAUAUAGCUCAGUUGGU	ACCAGCAGAGCUAUAUUCCA	Cleavage	1
trf_1_269	Bra010299	3	19.9	1	20	1873	1892	GGGGAUAUAGCUCAGUUGGU	ACCAGCAGAGCUAUAUUCCA	Cleavage	1
trf_1_270	Bra010299	3	19.9	1	24	1868	1892	GGGGAUAUAGCUCAGUUGGU-AGAG	UUCUCACCAGCAGAGCUAUAUUCCA	Cleavage	1
trf_1_283	Bra010299	3	19.9	1	20	1873	1892	GGGGAUAUAGCUCAGUUGGU	ACCAGCAGAGCUAUAUUCCA	Cleavage	1
trf_1_284	Bra010299	3	19.9	1	25	1867	1892	GGGGAUAUAGCUCAGUUGGU-AGAGC	GUUCUCACCAGCAGAGCUAUAUUCCA	Cleavage	1
trf_1_285	Bra010299	3	19.9	1	20	1873	1892	GGGGAUAUAGCUCAGUUGGU	ACCAGCAGAGCUAUAUUCCA	Cleavage	1
trf_1_294	Bra010299	3	19.9	1	20	1873	1892	GGGGAUAUAGCUCAGUUGGU	ACCAGCAGAGCUAUAUUCCA	Cleavage	1
trf_1_295	Bra010299	3	19.9	1	25	1867	1892	GGGGAUAUAGCUCAGUUGGU-AGAGC	GUUCUCACCAGCAGAGCUAUAUUCCA	Cleavage	1
trf_1_296	Bra010299	3	19.9	1	20	1873	1892	GGGGAUAUAGCUCAGUUGGU	ACCAGCAGAGCUAUAUUCCA	Cleavage	1
trf_1_298	Bra010299	3	19.9	1	24	1868	1892	GGGGAUAUAGCUCAGUUGGU-AGAG	UUCUCACCAGCAGAGCUAUAUUCCA	Cleavage	1
trf_1_301	Bra010299	3	19.9	1	20	1873	1892	GGGGAUAUAGCUCAGUUGGU	ACCAGCAGAGCUAUAUUCCA	Cleavage	1
trf_1_303	Bra010299	3	19.9	1	25	1867	1892	GGGGAUAUAGCUCAGUUGGU-AGAGC	GUUCUCACCAGCAGAGCUAUAUUCCA	Cleavage	1
trf_1_304	Bra010299	3	19.9	1	20	1873	1892	GGGGAUAUAGCUCAGUUGGU	ACCAGCAGAGCUAUAUUCCA	Cleavage	1
trf_1_305	Bra010299	3	19.9	1	20	1873	1892	GGGGAUAUAGCUCAGUUGGU	ACCAGCAGAGCUAUAUUCCA	Cleavage	1
trf_1_308	Bra010299	3	19.9	1	20	1873	1892	GGGGAUAUAGCUCAGUUGGU	ACCAGCAGAGCUAUAUUCCA	Cleavage	1

trf_1_3	Bra010		19.					GGGGAUAUAGCUCAGU	GUUCUCACCAGCAGAGC	Cleava	
09	299	3	9	1	25	1867	1892	UGGU-AGAGC	UAUAUUCCA	ge	1
trf_1_3	Bra010		19.					GGGGAUAUAGCUCAGU	ACCAGCAGAGCUAUAU	Cleava	
10	299	3	9	1	20	1873	1892	UGGU	UCCA	ge	1
trf_1_3	Bra010		19.					GGGGAUAUAGCUCAGU	UUCUCACCAGCAGAGCU	Cleava	
15	299	3	9	1	24	1868	1892	UGGU-AGAG	AUAUUCCA	ge	1
trf_1_3	Bra010		19.					GGGGAUAUAGCUCAGU	GUUCUCACCAGCAGAGC	Cleava	
16	299	3	9	1	25	1867	1892	UGGU-AGAGC	UAUAUUCCA	ge	1
trf_1_3	Bra010		19.					GGGGAUAUAGCUCAGU	ACCAGCAGAGCUAUAU	Cleava	
17	299	3	9	1	20	1873	1892	UGGU	UCCA	ge	1
trf_1_3	Bra010		19.					GGGGAUAUAGCUCAGU	ACCAGCAGAGCUAUAU	Cleava	
18	299	3	9	1	20	1873	1892	UGGU	UCCA	ge	1
trf_1_3	Bra010		19.					GGGGAUAUAGCUCAGU	GUUCUCACCAGCAGAGC	Cleava	
21	299	3	9	1	25	1867	1892	UGGU-AGAGC	UAUAUUCCA	ge	1
trf_1_3	Bra010		19.					GGGGAUAUAGCUCAGU	ACCAGCAGAGCUAUAU	Cleava	
24	299	3	9	1	20	1873	1892	UGGU	UCCA	ge	1
trf_1_3	Bra010		19.					GGGGAUAUAGCUCAGU	ACCAGCAGAGCUAUAU	Cleava	
25	299	3	9	1	20	1873	1892	UGGU	UCCA	ge	1
trf_1_3	Bra010		19.					GGGGAUAUAGCUCAGU	ACCAGCAGAGCUAUAU	Cleava	
33	299	3	9	1	20	1873	1892	UGGU	UCCA	ge	1
trf_1_3	Bra010		19.					GGGGAUAUAGCUCAGU	ACCAGCAGAGCUAUAU	Cleava	
34	299	3	9	1	20	1873	1892	UGGU	UCCA	ge	1
trf_1_3	Bra010		19.					GGGGAUAUAGCUCAGU	GUUCUCACCAGCAGAGC	Cleava	
35	299	3	9	1	25	1867	1892	UGGU-AGAGC	UAUAUUCCA	ge	1
trf_1_3	Bra010		19.					GGGGAUAUAGCUCAGU	ACCAGCAGAGCUAUAU	Cleava	
39	299	3	9	1	20	1873	1892	UGGU	UCCA	ge	1
trf_1_3	Bra010		19.					GGGGAUAUAGCUCAGU	GUUCUCACCAGCAGAGC	Cleava	
40	299	3	9	1	25	1867	1892	UGGU-AGAGC	UAUAUUCCA	ge	1
trf_1_3	Bra010		19.					GGGGAUAUAGCUCAGU	ACCAGCAGAGCUAUAU	Cleava	
42	299	3	9	1	20	1873	1892	UGGU	UCCA	ge	1
trf_1_3	Bra010		19.					GGGGAUAUAGCUCAGU	ACCAGCAGAGCUAUAU	Cleava	
43	299	3	9	1	20	1873	1892	UGGU	UCCA	ge	1
trf_1_3	Bra010		19.					GGGGAUAUAGCUCAGU	ACCAGCAGAGCUAUAU	Cleava	
44	299	3	9	1	20	1873	1892	UGGU	UCCA	ge	1

trf_1_352	Bra010299	3	19.9	1	20	1873	1892	GGGGAUAUAGCUCAGUUGGU	ACCAGCAGAGCUAUAUUCCA	Cleavage	1
trf_1_354	Bra010299	3	19.9	1	20	1873	1892	GGGGAUAUAGCUCAGUUGGU	ACCAGCAGAGCUAUAUUCCA	Cleavage	1
trf_1_355	Bra010299	3	19.9	1	20	1873	1892	GGGGAUAUAGCUCAGUUGGU	ACCAGCAGAGCUAUAUUCCA	Cleavage	1
trf_1_356	Bra010299	3	19.9	1	20	1873	1892	GGGGAUAUAGCUCAGUUGGU	ACCAGCAGAGCUAUAUUCCA	Cleavage	1
trf_1_357	Bra010299	3	19.9	1	25	1867	1892	GGGGAUAUAGCUCAGUUGGU-AGAGC	GUUCUCACCAGCAGAGCUAUAUUCCA	Cleavage	1
trf_1_358	Bra010299	3	19.9	1	24	1868	1892	GGGGAUAUAGCUCAGUUGGU-AGAG	UUCUCACCAGCAGAGCUAUAUUCCA	Cleavage	1
trf_1_361	Bra010299	3	19.9	1	20	1873	1892	GGGGAUAUAGCUCAGUUGGU	ACCAGCAGAGCUAUAUUCCA	Cleavage	1
trf_1_363	Bra010299	3	19.9	1	20	1873	1892	GGGGAUAUAGCUCAGUUGGU	ACCAGCAGAGCUAUAUUCCA	Cleavage	1
trf_1_364	Bra010299	3	19.9	1	25	1867	1892	GGGGAUAUAGCUCAGUUGGU-AGAGC	GUUCUCACCAGCAGAGCUAUAUUCCA	Cleavage	1
trf_1_365	Bra010299	3	19.9	1	20	1873	1892	GGGGAUAUAGCUCAGUUGGU	ACCAGCAGAGCUAUAUUCCA	Cleavage	1
trf_1_376	Bra010299	3	19.9	1	20	1873	1892	GGGGAUAUAGCUCAGUUGGU	ACCAGCAGAGCUAUAUUCCA	Cleavage	1
trf_1_377	Bra010299	3	19.9	1	20	1873	1892	GGGGAUAUAGCUCAGUUGGU	ACCAGCAGAGCUAUAUUCCA	Cleavage	1
trf_1_378	Bra010299	3	19.9	1	25	1867	1892	GGGGAUAUAGCUCAGUUGGU-AGAGC	GUUCUCACCAGCAGAGCUAUAUUCCA	Cleavage	1
trf_1_380	Bra010299	3	19.9	1	20	1873	1892	GGGGAUAUAGCUCAGUUGGU	ACCAGCAGAGCUAUAUUCCA	Cleavage	1
trf_1_384	Bra010299	3	19.9	1	20	1873	1892	GGGGAUAUAGCUCAGUUGGU	ACCAGCAGAGCUAUAUUCCA	Cleavage	1
trf_1_385	Bra010299	3	19.9	1	20	1873	1892	GGGGAUAUAGCUCAGUUGGU	ACCAGCAGAGCUAUAUUCCA	Cleavage	1
trf_1_386	Bra010299	3	19.9	1	25	1867	1892	GGGGAUAUAGCUCAGUUGGU-AGAGC	GUUCUCACCAGCAGAGCUAUAUUCCA	Cleavage	1

trf_1_387	Bra010299	3	19.9	1	20	1873	1892	GGGGAUAUAGCUCAGUUGGU	ACCAGCAGAGCUAUAUUCCA	Cleavage	1
trf_1_394	Bra010299	3	19.9	1	20	1873	1892	GGGGAUAUAGCUCAGUUGGU	ACCAGCAGAGCUAUAUUCCA	Cleavage	1
trf_1_395	Bra010299	3	19.9	1	25	1867	1892	GGGGAUAUAGCUCAGUUGGU-AGAGC	GUUCUCACCAGCAGAGCUAUAUUCCA	Cleavage	1
trf_1_396	Bra010299	3	19.9	1	20	1873	1892	GGGGAUAUAGCUCAGUUGGU	ACCAGCAGAGCUAUAUUCCA	Cleavage	1
trf_1_397	Bra010299	3	19.9	1	20	1873	1892	GGGGAUAUAGCUCAGUUGGU	ACCAGCAGAGCUAUAUUCCA	Cleavage	1
trf_1_401	Bra010299	3	19.9	1	25	1867	1892	GGGGAUAUAGCUCAGUUGGU-AGAGC	GUUCUCACCAGCAGAGCUAUAUUCCA	Cleavage	1
trf_1_402	Bra010299	3	19.9	1	20	1873	1892	GGGGAUAUAGCUCAGUUGGU	ACCAGCAGAGCUAUAUUCCA	Cleavage	1
trf_1_403	Bra010299	3	19.9	1	20	1873	1892	GGGGAUAUAGCUCAGUUGGU	ACCAGCAGAGCUAUAUUCCA	Cleavage	1
trf_1_405	Bra010299	3	19.9	1	24	1868	1892	GGGGAUAUAGCUCAGUUGGU-AGAG	UUCUCACCAGCAGAGCUAUAUUCCA	Cleavage	1
trf_1_406	Bra010299	3	19.9	1	20	1873	1892	GGGGAUAUAGCUCAGUUGGU	ACCAGCAGAGCUAUAUUCCA	Cleavage	1
trf_1_407	Bra010299	3	19.9	1	20	1873	1892	GGGGAUAUAGCUCAGUUGGU	ACCAGCAGAGCUAUAUUCCA	Cleavage	1
trf_1_409	Bra010299	3	19.9	1	25	1867	1892	GGGGAUAUAGCUCAGUUGGU-AGAGC	GUUCUCACCAGCAGAGCUAUAUUCCA	Cleavage	1
trf_1_411	Bra010299	3	19.9	1	20	1873	1892	GGGGAUAUAGCUCAGUUGGU	ACCAGCAGAGCUAUAUUCCA	Cleavage	1
trf_1_483	Bra010299	3	19.9	1	20	1873	1892	GGGGAUAUAGCUCAGUUGGU	ACCAGCAGAGCUAUAUUCCA	Cleavage	1
trf_1_484	Bra010299	3	19.9	1	20	1873	1892	GGGGAUAUAGCUCAGUUGGU	ACCAGCAGAGCUAUAUUCCA	Cleavage	1
trf_1_485	Bra010299	3	19.9	1	20	1873	1892	GGGGAUAUAGCUCAGUUGGU	ACCAGCAGAGCUAUAUUCCA	Cleavage	1
trf_1_487	Bra010299	3	19.9	1	25	1867	1892	GGGGAUAUAGCUCAGUUGGU-AGAGC	GUUCUCACCAGCAGAGCUAUAUUCCA	Cleavage	1

trf_1_488	Bra010299	3	19.9	1	20	1873	1892	GGGGAUAUAGCUCAGUUGGU	ACCAGCAGAGCUAUAUUCCA	Cleavage	1
trf_1_490	Bra010299	3	19.9	1	20	1873	1892	GGGGAUAUAGCUCAGUUGGU	ACCAGCAGAGCUAUAUUCCA	Cleavage	1
trf_1_491	Bra010299	3	19.9	1	25	1867	1892	GGGGAUAUAGCUCAGUUGGU-AGAGC	GUUCUCACCAGCAGAGCUAUAUUCCA	Cleavage	1
trf_1_492	Bra010299	3	19.9	1	20	1873	1892	GGGGAUAUAGCUCAGUUGGU	ACCAGCAGAGCUAUAUUCCA	Cleavage	1
trf_1_493	Bra010299	3	19.9	1	20	1873	1892	GGGGAUAUAGCUCAGUUGGU	ACCAGCAGAGCUAUAUUCCA	Cleavage	1
trf_1_495	Bra010299	3	19.9	1	24	1868	1892	GGGGAUAUAGCUCAGUUGGU-AGAG	UUCUCACCAGCAGAGCUAUAUUCCA	Cleavage	1
trf_1_500	Bra010299	3	19.9	1	20	1873	1892	GGGGAUAUAGCUCAGUUGGU	ACCAGCAGAGCUAUAUUCCA	Cleavage	1
trf_1_501	Bra010299	3	19.9	1	24	1868	1892	GGGGAUAUAGCUCAGUUGGU-AGAG	UUCUCACCAGCAGAGCUAUAUUCCA	Cleavage	1
trf_1_502	Bra010299	3	19.9	1	20	1873	1892	GGGGAUAUAGCUCAGUUGGU	ACCAGCAGAGCUAUAUUCCA	Cleavage	1
trf_1_503	Bra010299	3	19.9	1	25	1867	1892	GGGGAUAUAGCUCAGUUGGU-AGAGC	GUUCUCACCAGCAGAGCUAUAUUCCA	Cleavage	1
trf_1_504	Bra010299	3	19.9	1	20	1873	1892	GGGGAUAUAGCUCAGUUGGU	ACCAGCAGAGCUAUAUUCCA	Cleavage	1
trf_1_271	Bra010618	3	20.4	1	20	442	461	GCGUCCAUUGUCUAAUGGAU	AUGCAUCAGAUAGUGGACGC	Cleavage	1
trf_1_272	Bra010618	3	20.4	1	20	442	461	GCGUCCAUUGUCUAAUGGAU	AUGCAUCAGAUAGUGGACGC	Cleavage	1
trf_1_273	Bra010618	3	20.4	1	20	442	461	GCGUCCAUUGUCUAAUGGAU	AUGCAUCAGAUAGUGGACGC	Cleavage	1
trf_1_274	Bra010618	3	20.4	1	20	442	461	GCGUCCAUUGUCUAAUGGAU	AUGCAUCAGAUAGUGGACGC	Cleavage	1
trf_1_275	Bra010618	3	20.4	1	20	442	461	GCGUCCAUUGUCUAAUGGAU	AUGCAUCAGAUAGUGGACGC	Cleavage	1
trf_1_276	Bra010618	3	20.4	1	20	442	461	GCGUCCAUUGUCUAAUGGAU	AUGCAUCAGAUAGUGGACGC	Cleavage	1

trf_1_278	Bra010618	3	20.4	1	20	442	461	GCGUCCAUUGUCUAAUGGAU	AUGCAUCAGAUAGUGGACGC	Cleavage	1
trf_1_279	Bra010618	3	20.4	1	20	442	461	GCGUCCAUUGUCUAAUGGAU	AUGCAUCAGAUAGUGGACGC	Cleavage	1
trf_1_76	Bra011329	3	21.7	1	20	399	418	GCGCCUGUAGCUCAGUGGAA	UUCCACUGAGGUGAAGGCGC	Translation	1
trf_1_428	Bra011657	3	15.7	1	23	764	786	GGGAUCGUAGUUCAAUUGGUCAG	CAGAACAAUUUAACUACGGUUCC	Cleavage	1
trf_1_464	Bra011657	2.5	15.7	1	23	764	786	GGGAUUGUAGUUAAAUUGGUCAG	CAGAACAAUUUAACUACGGUUCC	Cleavage	1
trf_1_94	Bra012143	2.5	18.5	1	20	392	411	UCCGUUGUCGUCCAGCGGUU	AAUCGCUGAACCACAACGGA	Translation	1
trf_1_95	Bra012143	2.5	18.5	1	20	392	411	UCCGUUGUCGUCCAGCGGUU	AAUCGCUGAACCACAACGGA	Translation	1
trf_1_259	Bra012143	2.5	18.5	1	20	392	411	UCCGUUGUCGUCCAGCGGUU	AAUCGCUGAACCACAACGGA	Translation	1
trf_1_261	Bra012143	2.5	18.5	1	20	392	411	UCCGUUGUCGUCCAGCGGUU	AAUCGCUGAACCACAACGGA	Translation	1
trf_1_287	Bra012143	2.5	18.5	1	20	392	411	UCCGUUGUCGUCCAGCGGUU	AAUCGCUGAACCACAACGGA	Translation	1
trf_1_288	Bra012143	2.5	18.5	1	20	392	411	UCCGUUGUCGUCCAGCGGUU	AAUCGCUGAACCACAACGGA	Translation	1
trf_1_510	Bra012143	2.5	18.5	1	20	392	411	UCCGUUGUCGUCCAGCGGUU	AAUCGCUGAACCACAACGGA	Translation	1
trf_1_511	Bra012143	2.5	18.5	1	20	392	411	UCCGUUGUCGUCCAGCGGUU	AAUCGCUGAACCACAACGGA	Translation	1
trf_1_512	Bra012143	2.5	18.5	1	20	392	411	UCCGUUGUCGUCCAGCGGUU	AAUCGCUGAACCACAACGGA	Translation	1
trf_1_452	Bra012183	3	9.2	1	20	301	320	GGGAUUGUAGUUCAAUUGAA	UUCAAUUCAAUUACAAUUCG	Cleavage	1
trf_1_54	Bra012326	3	15.0	1	20	1326	1345	GUCUGGGUGGUGUAGUCGGU	ACCGGUUGCACCGUCCGGAC	Cleavage	1
trf_1_188	Bra012326	3	15.0	1	20	1326	1345	GUCUGGGUGGUGUAGUCGGU	ACCGGUUGCACCGUCCGGAC	Cleavage	1

trf_1_5	Bra012		15.					GUCUGGGUGGUGUAGU	ACCGGUUGCACCGUCCG	Cleava	
28	326	3	0	1	20	1326	1345	CGGU	GAC	ge	1
trf_1_1	Bra012		14.					AGGGAUAUAACUCAGCG	UACCUUUGAGUUAUGU	Cleava	
95	734	3	1	1	20	1709	1728	GUA	UUCU	ge	1
trf_1_1	Bra012		14.					AGGGAUAUAACUCAGCG	UACCUUUGAGUUAUGU	Cleava	
96	734	3	1	1	20	1709	1728	GUA	UUCU	ge	1
trf_1_1	Bra012		14.					AGGGAUAUAACUCAGCG	UACCUUUGAGUUAUGU	Cleava	
97	734	3	1	1	20	1709	1728	GUA	UUCU	ge	1
trf_1_1	Bra012		14.					AGGGAUAUAACUCAGCG	UACCUUUGAGUUAUGU	Cleava	
98	734	3	1	1	20	1709	1728	GUA	UUCU	ge	1
trf_1_1	Bra012		14.					AGGGAUAUAACUCAGCG	UACCUUUGAGUUAUGU	Cleava	
99	734	3	1	1	20	1709	1728	GUA	UUCU	ge	1
trf_1_2	Bra013		18.					GGGUCGAUGCCCGAGCG	UAACCGAUCGGGCCUCG	Cleava	
11	150	2.5	5	1	21	93	113	GUUA	AUCC	ge	1
trf_1_2	Bra013		18.					GGGUCGAUGCCCGAGCG	UAACCGAUCGGGCCUCG	Cleava	
13	150	2.5	5	1	21	93	113	GUUA	AUCC	ge	1
trf_1_2	Bra013		18.					GGGUCGAUGCCCGAGCG	UAACCGAUCGGGCCUCG	Cleava	
18	150	2.5	5	1	21	93	113	GUUA	AUCC	ge	1
trf_1_2	Bra013		18.					GGGUCGAUGCCCGAGCG	UAACCGAUCGGGCCUCG	Cleava	
19	150	2.5	5	1	21	93	113	GUUA	AUCC	ge	1
trf_1_2	Bra013		18.					GGGUCGAUGCCCGAGCG	GCUAACCGAUCGGGCCU	Cleava	
20	150	2.5	5	1	23	91	113	GUUAAC	CGAUCC	ge	1
trf_1_2	Bra013		18.					GGGUCGAUGCCCGAGCG	UAACCGAUCGGGCCUCG	Cleava	
23	150	2.5	5	1	21	93	113	GUUA	AUCC	ge	1
trf_1_2	Bra013		18.					GGGUCGAUGCCCGAGCG	UAACCGAUCGGGCCUCG	Cleava	
24	150	2.5	5	1	21	93	113	GUUA	AUCC	ge	1
trf_1_2	Bra013		18.					GGGUCGAUGCCCGAGCG	UAACCGAUCGGGCCUCG	Cleava	
25	150	2.5	5	1	21	93	113	GUUA	AUCC	ge	1
trf_1_2	Bra013		18.					GGGUCGAUGCCCGAGCG	UAACCGAUCGGGCCUCG	Cleava	
26	150	2.5	5	1	21	93	113	GUUA	AUCC	ge	1
trf_1_2	Bra013		18.					GGGUCGAUGCCCGAGCG	CUAACCGAUCGGGCCUC	Cleava	
28	150	2.5	5	1	22	92	113	GUUAG	GAUCC	ge	1
trf_1_2	Bra013		18.					GGGUCGAUGCCCGAGCG	CUAACCGAUCGGGCCUC	Cleava	
29	150	2.5	5	1	22	92	113	GUUAG	GAUCC	ge	1

trf_1_414	Bra013336	3	19.5	1	20	267	286	GGGAUUGUAGUUCAAUUGGU	ACUAAUUGAACUAAGAUCUC	Cleavage	1
trf_1_415	Bra013336	3	19.5	1	20	267	286	GGGAUUGUAGUUCAAUUGGU	ACUAAUUGAACUAAGAUCUC	Cleavage	1
trf_1_416	Bra013336	3	19.5	1	20	267	286	GGGAUUGUAGUUCAAUUGGU	ACUAAUUGAACUAAGAUCUC	Cleavage	1
trf_1_422	Bra013336	3	19.5	1	20	267	286	GGGAUUGUAGUUCAAUUGGU	ACUAAUUGAACUAAGAUCUC	Cleavage	1
trf_1_423	Bra013336	3	19.5	1	20	267	286	GGGAUUGUAGUUCAAUUGGU	ACUAAUUGAACUAAGAUCUC	Cleavage	1
trf_1_424	Bra013336	3	19.5	1	20	267	286	GGGAUUGUAGUUCAAUUGGU	ACUAAUUGAACUAAGAUCUC	Cleavage	1
trf_1_426	Bra013336	3	19.5	1	20	267	286	GGGAUUGUAGUUCAAUUGGU	ACUAAUUGAACUAAGAUCUC	Cleavage	1
trf_1_428	Bra013336	2.5	19.5	1	20	267	286	GGGAUCGUAGUUCAAUUGGU	ACUAAUUGAACUAAGAUCUC	Cleavage	1
trf_1_429	Bra013336	3	19.5	1	20	267	286	GGGAUUGUAGUUCAAUUGGU	ACUAAUUGAACUAAGAUCUC	Cleavage	1
trf_1_431	Bra013336	3	19.5	1	20	267	286	GGGAUUGUAGUUCAAUUGGU	ACUAAUUGAACUAAGAUCUC	Cleavage	1
trf_1_438	Bra013336	3	19.5	1	23	264	286	GGGAUUGUAGUUCAAUUGGUCGG	CCAACUAAUUGAACUAAGAUCUC	Cleavage	1
trf_1_439	Bra013336	3	19.5	1	23	264	286	GGGAUUGUAGUUCAAUUGGUCGG	CCAACUAAUUGAACUAAGAUCUC	Cleavage	1
trf_1_440	Bra013336	3	19.5	1	23	264	286	GGGAUUGUAGUUCAAUUGGUCGG	CCAACUAAUUGAACUAAGAUCUC	Cleavage	1
trf_1_443	Bra013336	3	19.5	1	22	265	286	GGGAUUGUAGUUCAAUUGGUCG	CAACUAAUUGAACUAAGAUCUC	Cleavage	1
trf_1_444	Bra013336	3	19.5	1	23	264	286	GGGAUUGUAGUUCAAUUGGUCGG	CCAACUAAUUGAACUAAGAUCUC	Cleavage	1
trf_1_449	Bra013336	3	19.5	1	20	267	286	GGGAUUGUAGUUCAAUUGGU	ACUAAUUGAACUAAGAUCUC	Cleavage	1
trf_1_450	Bra013336	3	19.5	1	20	267	286	GGGAUUGUAGUUCAAUUGGU	ACUAAUUGAACUAAGAUCUC	Cleavage	1

trf_1_451	Bra013336	3	19.5	1	20	267	286	GGGAUUGUAGUUCAAUUGGU	ACUAAUUGAACUAAGAUCUC	Cleavage	1
trf_1_453	Bra013336	3	19.5	1	20	267	286	GGGAUUGUAGUUCAAUUGGU	ACUAAUUGAACUAAGAUCUC	Cleavage	1
trf_1_455	Bra013336	3	19.5	1	20	267	286	GGGAUUGUAGUUCAAUUGGU	ACUAAUUGAACUAAGAUCUC	Cleavage	1
trf_1_457	Bra013336	3	19.5	1	21	266	286	GGGAUUGUAGUUCAAUUGGUU	AACUAAUUGAACUAAGAUCUC	Cleavage	1
trf_1_458	Bra013336	3	19.5	1	20	267	286	GGGAUUGUAGUUCAAUUGGU	ACUAAUUGAACUAAGAUCUC	Cleavage	1
trf_1_463	Bra013336	3	19.5	1	20	267	286	GGGAUUGUAGUUCAAUUGGU	ACUAAUUGAACUAAGAUCUC	Cleavage	1
trf_1_467	Bra013336	3	19.5	1	20	267	286	GGGAUUGUAGUUCAAUUGGU	ACUAAUUGAACUAAGAUCUC	Cleavage	1
trf_1_468	Bra013336	3	19.5	1	23	264	286	GGGAUUGUAGUUCAAUUGGUUAG	CCAACUAAUUGAACUAAGAUCUC	Cleavage	1
trf_1_471	Bra013336	3	19.5	1	20	267	286	GGGAUUGUAGUUCAAUUGGU	ACUAAUUGAACUAAGAUCUC	Cleavage	1
trf_1_14	Bra013348	3	14.5	1	21	1004	1024	GGGGAUAUAGCUCAGUUGGGA	UCCCUACAGAGUUGUAUCCCC	Cleavage	1
trf_1_11	Bra013528	3	22.0	1	24	196	219	UCCGUUGUAGUCUAGCUGGUUAGG	CAUCAUCAGCUUGGCUAUAACGGG	Cleavage	1
trf_1_12	Bra013528	3	22.0	1	24	196	219	UCCGUUGUAGUCUAGCUGGUUAGG	CAUCAUCAGCUUGGCUAUAACGGG	Cleavage	1
trf_1_42	Bra013528	3	22.0	1	24	196	219	UCCGUUGUAGUCUAGCUGGUCAGG	CAUCAUCAGCUUGGCUAUAACGGG	Cleavage	1
trf_1_88	Bra013528	3	22.0	1	24	196	219	UCCGUUGUAGUCUAGCUGGUCAGG	CAUCAUCAGCUUGGCUAUAACGGG	Cleavage	1
trf_1_128	Bra013528	3	22.0	1	24	196	219	UCCGUUGUAGUCUAGCUGGUCAGG	CAUCAUCAGCUUGGCUAUAACGGG	Cleavage	1
trf_1_140	Bra013528	3	22.0	1	24	196	219	UCCGUUGUAGUCUAGCUGGUCAGG	CAUCAUCAGCUUGGCUAUAACGGG	Cleavage	1
trf_1_159	Bra013528	3	22.0	1	24	196	219	UCCGUUGUAGUCUAGCUGGUCAGG	CAUCAUCAGCUUGGCUAUAACGGG	Cleavage	1

trf_1_263	Bra013528	3	22.0	1	24	196	219	UCCGUUGUAGUCUAGCUGGUCAGG	CAUCAUCAGCUUGGCUAUAACGGG	Cleavage	1
trf_1_326	Bra013528	3	22.0	1	24	196	219	UCCGUUGUAGUCUAGCUGGUCAGG	CAUCAUCAGCUUGGCUAUAACGGG	Cleavage	1
trf_1_570	Bra013528	3	22.0	1	24	196	219	UCCGUUGUAGUCUAGCUGGUCAGG	CAUCAUCAGCUUGGCUAUAACGGG	Cleavage	1
trf_1_54	Bra013558	3	11.8	1	21	165	185	GUCUGGGUGGUGUAGUCGGUU	AACCGACUCCACAAUUCAGAC	Translation	1
trf_1_188	Bra013558	3	11.8	1	21	165	185	GUCUGGGUGGUGUAGUCGGUU	AACCGACUCCACAAUUCAGAC	Translation	1
trf_1_528	Bra013558	3	11.8	1	21	165	185	GUCUGGGUGGUGUAGUCGGUU	AACCGACUCCACAAUUCAGAC	Translation	1
trf_1_94	Bra013584	3	16.4	1	25	886	910	UCCGUUGUCGUCCAGCGGUUAGGAU	AUGAUAACCGAUGGAUGUCAAUGGA	Cleavage	1
trf_1_95	Bra013584	3	16.4	1	26	885	910	UCCGUUGUCGUCCAGCGGUUAGGAUA	UAUGAUAACCGAUGGAUGUCAAUGGA	Cleavage	1
trf_1_259	Bra013584	3	16.4	1	20	891	910	UCCGUUGUCGUCCAGCGGUU	AACCGAUGGAUGUCAAUGGA	Cleavage	1
trf_1_261	Bra013584	3	16.4	1	26	885	910	UCCGUUGUCGUCCAGCGGUUAGGAUA	UAUGAUAACCGAUGGAUGUCAAUGGA	Cleavage	1
trf_1_287	Bra013584	3	16.4	1	25	886	910	UCCGUUGUCGUCCAGCGGUUAGGAU	AUGAUAACCGAUGGAUGUCAAUGGA	Cleavage	1
trf_1_288	Bra013584	3	16.4	1	26	885	910	UCCGUUGUCGUCCAGCGGUUAGGAUA	UAUGAUAACCGAUGGAUGUCAAUGGA	Cleavage	1
trf_1_510	Bra013584	3	16.4	1	25	886	910	UCCGUUGUCGUCCAGCGGUUAGGAU	AUGAUAACCGAUGGAUGUCAAUGGA	Cleavage	1
trf_1_511	Bra013584	3	16.4	1	26	885	910	UCCGUUGUCGUCCAGCGGUUAGGAUA	UAUGAUAACCGAUGGAUGUCAAUGGA	Cleavage	1
trf_1_512	Bra013584	3	16.4	1	21	890	910	UCCGUUGUCGUCCAGCGGUUA	UAACCGAUGGAUGUCAAUGGA	Cleavage	1
trf_1_425	Bra014182	3	17.7	1	20	689	708	UGGAUUGUAGUUCAAUUGGU	AUCAAGUGAGUUACAAUCUA	Cleavage	1
trf_1_462	Bra014182	3	17.7	1	20	689	708	UGGAUUGUAGUUCAAUUGGU	AUCAAGUGAGUUACAAUCUA	Cleavage	1

trf_1_466	Bra014182	3	17.7	1	20	689	708	UGGAUUGUAGUUCAAUUGGU	AUCAAGUGAGUUACAAUCUA	Cleavage	1
trf_1_119	Bra014507	3	17.0	1	20	544	563	GGGGAUGUAGCUCAUAUGGU	AUUAUAUUAGCUAUAUCUCC	Cleavage	1
trf_1_120	Bra014507	3	17.0	1	23	541	563	GGGGAUGUAGCUCAUAUGGUAGA	UGUAUUAUAUUAGCUAUAUCUCC	Cleavage	1
trf_1_289	Bra014507	3	17.0	1	23	541	563	GGGGAUGUAGCUCAUAUGGUAGA	UGUAUUAUAUUAGCUAUAUCUCC	Cleavage	1
trf_1_290	Bra014507	3	17.0	1	20	544	563	GGGGAUGUAGCUCAUAUGGU	AUUAUAUUAGCUAUAUCUCC	Cleavage	1
trf_1_388	Bra014507	3	17.0	1	20	544	563	GGGGAUGUAGCUCAUAUGGU	AUUAUAUUAGCUAUAUCUCC	Cleavage	1
trf_1_389	Bra014507	3	17.0	1	23	541	563	GGGGAUGUAGCUCAUAUGGUAGA	UGUAUUAUAUUAGCUAUAUCUCC	Cleavage	1
trf_1_481	Bra014507	3	17.0	1	23	541	563	GGGGAUGUAGCUCAUAUGGUAGA	UGUAUUAUAUUAGCUAUAUCUCC	Cleavage	1
trf_1_496	Bra014507	3	17.0	1	23	541	563	GGGGAUGUAGCUCAUAUGGUAGA	UGUAUUAUAUUAGCUAUAUCUCC	Cleavage	1
trf_1_497	Bra014507	3	17.0	1	20	544	563	GGGGAUGUAGCUCAUAUGGU	AUUAUAUUAGCUAUAUCUCC	Cleavage	1
trf_1_258	Bra015824	3	24.0	1	26	913	938	AUCAGAGUGGCGCAGCGGAAGCGUGG	CUCCGUUUCCACUGCACCACUCUGAC	Translation	1
trf_1_569	Bra015824	3	24.0	1	26	913	938	AUCAGAGUGGCGCAGCGGAAGCGUGG	CUCCGUUUCCACUGCACCACUCUGAC	Translation	1
trf_1_31	Bra016141	3	10.4	1	22	3031	3052	GGGGAUGUAGCUCAGAUGGUAG	UUAUCAUCUGAGCUUCGUUCUC	Cleavage	1
trf_1_35	Bra016141	3	10.4	1	20	3033	3052	GGGGAUGUAGCUCAGAUGGU	AUCAUCUGAGCUUCGUUCUC	Cleavage	1
trf_1_60	Bra016141	3	10.4	1	22	3031	3052	GGGGAUGUAGCUCAGAUGGUAG	UUAUCAUCUGAGCUUCGUUCUC	Cleavage	1
trf_1_62	Bra016141	3	10.4	1	24	3029	3052	GGGGAUGUAGCUCAGAUGGUAGAG	CAUUAUCAUCUGAGCUUCGUUCUC	Cleavage	1
trf_1_90	Bra016141	3	10.4	1	22	3031	3052	GGGGAUGUAGCUCAGAUGGUAG	UUAUCAUCUGAGCUUCGUUCUC	Cleavage	1

trf_1_133	Bra016141	3	10.4	1	22	3031	3052	GGGGAUGUAGCUCAGAUGGUAG	UUAUCAUCUGAGCUUCGUUCUC	Cleavage	1
trf_1_137	Bra016141	3	10.4	1	20	3033	3052	GGGGAUGUAGCUCAGAUGGU	AUCAUCUGAGCUUCGUUCUC	Cleavage	1
trf_1_138	Bra016141	3	10.4	1	21	3032	3052	GGGGAUGUAGCUCAGAUGGUA	UAUCAUCUGAGCUUCGUUCUC	Cleavage	1
trf_1_153	Bra016141	3	10.4	1	22	3031	3052	GGGGAUGUAGCUCAGAUGGUAG	UUAUCAUCUGAGCUUCGUUCUC	Cleavage	1
trf_1_154	Bra016141	3	10.4	1	24	3029	3052	GGGGAUGUAGCUCAGAUGGUAGAG	CAUUAUCAUCUGAGCUUCGUUCUC	Cleavage	1
trf_1_160	Bra016141	3	10.4	1	22	3031	3052	GGGGAUGUAGCUCAGAUGGUAG	UUAUCAUCUGAGCUUCGUUCUC	Cleavage	1
trf_1_162	Bra016141	3	10.4	1	24	3029	3052	GGGGAUGUAGCUCAGAUGGUAGAG	CAUUAUCAUCUGAGCUUCGUUCUC	Cleavage	1
trf_1_169	Bra016141	3	10.4	1	22	3031	3052	GGGGAUGUAGCUCAGAUGGUAG	UUAUCAUCUGAGCUUCGUUCUC	Cleavage	1
trf_1_173	Bra016141	3	10.4	1	24	3029	3052	GGGGAUGUAGCUCAGAUGGUAGAG	CAUUAUCAUCUGAGCUUCGUUCUC	Cleavage	1
trf_1_183	Bra016141	3	10.4	1	22	3031	3052	GGGGAUGUAGCUCAGAUGGUAG	UUAUCAUCUGAGCUUCGUUCUC	Cleavage	1
trf_1_233	Bra016141	3	10.4	1	22	3031	3052	GGGGAUGUAGCUCAGAUGGUAG	UUAUCAUCUGAGCUUCGUUCUC	Cleavage	1
trf_1_236	Bra016141	3	10.4	1	24	3029	3052	GGGGAUGUAGCUCAGAUGGUAGAG	CAUUAUCAUCUGAGCUUCGUUCUC	Cleavage	1
trf_1_244	Bra016141	3	10.4	1	22	3031	3052	GGGGAUGUAGCUCAGAUGGUAG	UUAUCAUCUGAGCUUCGUUCUC	Cleavage	1
trf_1_247	Bra016141	3	10.4	1	24	3029	3052	GGGGAUGUAGCUCAGAUGGUAGAG	CAUUAUCAUCUGAGCUUCGUUCUC	Cleavage	1
trf_1_266	Bra016141	3	10.4	1	22	3031	3052	GGGGAUGUAGCUCAGAUGGUAG	UUAUCAUCUGAGCUUCGUUCUC	Cleavage	1
trf_1_286	Bra016141	3	10.4	1	22	3031	3052	GGGGAUGUAGCUCAGAUGGUAG	UUAUCAUCUGAGCUUCGUUCUC	Cleavage	1
trf_1_293	Bra016141	3	10.4	1	22	3031	3052	GGGGAUGUAGCUCAGAUGGUAG	UUAUCAUCUGAGCUUCGUUCUC	Cleavage	1

trf_1_297	Bra016141	3	10.4	1	24	3029	3052	GGGGAUGUAGCUCAGAUGGUAGAG	CAUUAUCAUCUGAGCUUCGUUCUC	Cleavage	1
trf_1_302	Bra016141	3	10.4	1	22	3031	3052	GGGGAUGUAGCUCAGAUGGUAG	UUAUCAUCUGAGCUUCGUUCUC	Cleavage	1
trf_1_307	Bra016141	3	10.4	1	22	3031	3052	GGGGAUGUAGCUCAGAUGGUAG	UUAUCAUCUGAGCUUCGUUCUC	Cleavage	1
trf_1_314	Bra016141	3	10.4	1	22	3031	3052	GGGGAUGUAGCUCAGAUGGUAG	UUAUCAUCUGAGCUUCGUUCUC	Cleavage	1
trf_1_322	Bra016141	3	10.4	1	22	3031	3052	GGGGAUGUAGCUCAGAUGGUAG	UUAUCAUCUGAGCUUCGUUCUC	Cleavage	1
trf_1_323	Bra016141	3	10.4	1	24	3029	3052	GGGGAUGUAGCUCAGAUGGUAGAG	CAUUAUCAUCUGAGCUUCGUUCUC	Cleavage	1
trf_1_332	Bra016141	3	10.4	1	22	3031	3052	GGGGAUGUAGCUCAGAUGGUAG	UUAUCAUCUGAGCUUCGUUCUC	Cleavage	1
trf_1_338	Bra016141	3	10.4	1	24	3029	3052	GGGGAUGUAGCUCAGAUGGUAGAG	CAUUAUCAUCUGAGCUUCGUUCUC	Cleavage	1
trf_1_341	Bra016141	3	10.4	1	22	3031	3052	GGGGAUGUAGCUCAGAUGGUAG	UUAUCAUCUGAGCUUCGUUCUC	Cleavage	1
trf_1_351	Bra016141	3	10.4	1	22	3031	3052	GGGGAUGUAGCUCAGAUGGUAG	UUAUCAUCUGAGCUUCGUUCUC	Cleavage	1
trf_1_353	Bra016141	3	10.4	1	20	3033	3052	GGGGAUGUAGCUCAGAUGGU	AUCAUCUGAGCUUCGUUCUC	Cleavage	1
trf_1_362	Bra016141	3	10.4	1	22	3031	3052	GGGGAUGUAGCUCAGAUGGUAG	UUAUCAUCUGAGCUUCGUUCUC	Cleavage	1
trf_1_366	Bra016141	3	10.4	1	24	3029	3052	GGGGAUGUAGCUCAGAUGGUAGAG	CAUUAUCAUCUGAGCUUCGUUCUC	Cleavage	1
trf_1_375	Bra016141	3	10.4	1	22	3031	3052	GGGGAUGUAGCUCAGAUGGUAG	UUAUCAUCUGAGCUUCGUUCUC	Cleavage	1
trf_1_379	Bra016141	3	10.4	1	24	3029	3052	GGGGAUGUAGCUCAGAUGGUAGAG	CAUUAUCAUCUGAGCUUCGUUCUC	Cleavage	1
trf_1_382	Bra016141	3	10.4	1	22	3031	3052	GGGGAUGUAGCUCAGAUGGUAG	UUAUCAUCUGAGCUUCGUUCUC	Cleavage	1
trf_1_383	Bra016141	3	10.4	1	24	3029	3052	GGGGAUGUAGCUCAGAUGGUAGAG	CAUUAUCAUCUGAGCUUCGUUCUC	Cleavage	1

trf_1_391	Bra016141	3	10.4	1	22	3031	3052	GGGGAUGUAGCUCAGAUGGUAG	UUAUCAUCUGAGCUUCGUUCUC	Cleavage	1
trf_1_392	Bra016141	3	10.4	1	20	3033	3052	GGGGAUGUAGCUCAGAUGGU	AUCAUCUGAGCUUCGUUCUC	Cleavage	1
trf_1_393	Bra016141	3	10.4	1	24	3029	3052	GGGGAUGUAGCUCAGAUGGUAGAG	CAUUAUCAUCUGAGCUUCGUUCUC	Cleavage	1
trf_1_400	Bra016141	3	10.4	1	22	3031	3052	GGGGAUGUAGCUCAGAUGGUAG	UUAUCAUCUGAGCUUCGUUCUC	Cleavage	1
trf_1_404	Bra016141	3	10.4	1	20	3033	3052	GGGGAUGUAGCUCAGAUGGU	AUCAUCUGAGCUUCGUUCUC	Cleavage	1
trf_1_408	Bra016141	3	10.4	1	22	3031	3052	GGGGAUGUAGCUCAGAUGGUAG	UUAUCAUCUGAGCUUCGUUCUC	Cleavage	1
trf_1_410	Bra016141	3	10.4	1	20	3033	3052	GGGGAUGUAGCUCAGAUGGU	AUCAUCUGAGCUUCGUUCUC	Cleavage	1
trf_1_482	Bra016141	3	10.4	1	22	3031	3052	GGGGAUGUAGCUCAGAUGGUAG	UUAUCAUCUGAGCUUCGUUCUC	Cleavage	1
trf_1_486	Bra016141	3	10.4	1	20	3033	3052	GGGGAUGUAGCUCAGAUGGU	AUCAUCUGAGCUUCGUUCUC	Cleavage	1
trf_1_489	Bra016141	3	10.4	1	22	3031	3052	GGGGAUGUAGCUCAGAUGGUAG	UUAUCAUCUGAGCUUCGUUCUC	Cleavage	1
trf_1_494	Bra016141	3	10.4	1	20	3033	3052	GGGGAUGUAGCUCAGAUGGU	AUCAUCUGAGCUUCGUUCUC	Cleavage	1
trf_1_498	Bra016141	3	10.4	1	20	3033	3052	GGGGAUGUAGCUCAGAUGGU	AUCAUCUGAGCUUCGUUCUC	Cleavage	1
trf_1_499	Bra016141	3	10.4	1	22	3031	3052	GGGGAUGUAGCUCAGAUGGUAG	UUAUCAUCUGAGCUUCGUUCUC	Cleavage	1
trf_1_1	Bra016584	3	9.9	1	20	1045	1064	GUCGUUGUAGUAUAGUGGUA	UACUAUUAUACUACAAAGGC	Cleavage	1
trf_1_24	Bra016584	3	9.9	1	20	1045	1064	GUCGUUGUAGUAUAGUGGUA	UACUAUUAUACUACAAAGGC	Cleavage	1
trf_1_25	Bra016584	3	9.9	1	20	1045	1064	GUCGUUGUAGUAUAGUGGUA	UACUAUUAUACUACAAAGGC	Cleavage	1
trf_1_26	Bra016584	3	9.9	1	20	1045	1064	GUCGUUGUAGUAUAGUGGUA	UACUAUUAUACUACAAAGGC	Cleavage	1

trf_1_50	Bra016584	3	9.9	1	20	1045	1064	GUCGUUGUAGUAUAGUGGUA	UACUAUUAUACUACAAAGGC	Cleavage	1
trf_1_51	Bra016584	3	9.9	1	20	1045	1064	GUCGUUGUAGUAUAGUGGUA	UACUAUUAUACUACAAAGGC	Cleavage	1
trf_1_52	Bra016584	3	9.9	1	20	1045	1064	GUCGUUGUAGUAUAGUGGUA	UACUAUUAUACUACAAAGGC	Cleavage	1
trf_1_53	Bra016584	3	9.9	1	20	1045	1064	GUCGUUGUAGUAUAGUGGUA	UACUAUUAUACUACAAAGGC	Cleavage	1
trf_1_65	Bra016584	3	9.9	1	20	1045	1064	GUCGUUGUAGUAUAGUGGUA	UACUAUUAUACUACAAAGGC	Cleavage	1
trf_1_66	Bra016584	3	9.9	1	20	1045	1064	GUCGUUGUAGUAUAGUGGUA	UACUAUUAUACUACAAAGGC	Cleavage	1
trf_1_67	Bra016584	3	9.9	1	20	1045	1064	GUCGUUGUAGUAUAGUGGUA	UACUAUUAUACUACAAAGGC	Cleavage	1
trf_1_125	Bra016584	3	9.9	1	20	1045	1064	GUCGUUGUAGUAUAGUGGUA	UACUAUUAUACUACAAAGGC	Cleavage	1
trf_1_131	Bra016584	3	9.9	1	20	1045	1064	GUCGUUGUAGUAUAGUGGUA	UACUAUUAUACUACAAAGGC	Cleavage	1
trf_1_132	Bra016584	3	9.9	1	20	1045	1064	GUCGUUGUAGUAUAGUGGUA	UACUAUUAUACUACAAAGGC	Cleavage	1
trf_1_230	Bra016584	3	9.9	1	20	1045	1064	GUCGUUGUAGUAUAGUGGUA	UACUAUUAUACUACAAAGGC	Cleavage	1
trf_1_257	Bra016584	3	9.9	1	20	1045	1064	GUCGUUGUAGUAUAGUGGUA	UACUAUUAUACUACAAAGGC	Cleavage	1
trf_1_281	Bra016584	3	9.9	1	20	1045	1064	GUCGUUGUAGUAUAGUGGUA	UACUAUUAUACUACAAAGGC	Cleavage	1
trf_1_282	Bra016584	3	9.9	1	20	1045	1064	GUCGUUGUAGUAUAGUGGUA	UACUAUUAUACUACAAAGGC	Cleavage	1
trf_1_306	Bra016584	3	9.9	1	20	1045	1064	GUCGUUGUAGUAUAGUGGUA	UACUAUUAUACUACAAAGGC	Cleavage	1
trf_1_331	Bra016584	3	9.9	1	20	1045	1064	GUCGUUGUAGUAUAGUGGUA	UACUAUUAUACUACAAAGGC	Cleavage	1
trf_1_372	Bra016584	3	9.9	1	20	1045	1064	GUCGUUGUAGUAUAGUGGUA	UACUAUUAUACUACAAAGGC	Cleavage	1

trf_1_373	Bra016584	3	9.9	1	20	1045	1064	GUCGUUGUAGUAUAGUGGUA	UACUAUUAUACUACAAAGGC	Cleavage	1
trf_1_374	Bra016584	3	9.9	1	20	1045	1064	GUCGUUGUAGUAUAGUGGUA	UACUAUUAUACUACAAAGGC	Cleavage	1
trf_1_390	Bra016584	3	9.9	1	20	1045	1064	GUCGUUGUAGUAUAGUGGUA	UACUAUUAUACUACAAAGGC	Cleavage	1
trf_1_399	Bra016584	3	9.9	1	20	1045	1064	GUCGUUGUAGUAUAGUGGUA	UACUAUUAUACUACAAAGGC	Cleavage	1
trf_1_524	Bra016584	3	9.9	1	20	1045	1064	GUCGUUGUAGUAUAGUGGUA	UACUAUUAUACUACAAAGGC	Cleavage	1
trf_1_532	Bra016584	3	9.9	1	20	1045	1064	GUCGUUGUAGUAUAGUGGUA	UACUAUUAUACUACAAAGGC	Cleavage	1
trf_1_576	Bra016584	3	9.9	1	20	1045	1064	GUCGUUGUAGUAUAGUGGUA	UACUAUUAUACUACAAAGGC	Cleavage	1
trf_1_21	Bra016601	3	20.8	1	24	415	438	UCCGUCGUAGUCUAGCUGGUUAGG	CUGAAGAAGCUAGGCUGCGACGGA	Cleavage	1
trf_1_22	Bra016601	3	20.8	1	24	415	438	UCCGUCGUAGUCUAGCUGGUUAGG	CUGAAGAAGCUAGGCUGCGACGGA	Cleavage	1
trf_1_291	Bra016601	3	20.8	1	24	415	438	UCCGUCGUAGUCUAGCUGGUUAGG	CUGAAGAAGCUAGGCUGCGACGGA	Cleavage	1
trf_1_292	Bra016601	3	20.8	1	24	415	438	UCCGUCGUAGUCUAGCUGGUUAGG	CUGAAGAAGCUAGGCUGCGACGGA	Cleavage	1
trf_1_516	Bra016601	3	20.8	1	24	415	438	UCCGUCGUAGUCUAGCUGGUUAGG	CUGAAGAAGCUAGGCUGCGACGGA	Cleavage	1
trf_1_521	Bra016601	3	20.8	1	24	415	438	UCCGUCGUAGUCUAGCUGGUUAGG	CUGAAGAAGCUAGGCUGCGACGGA	Cleavage	1
trf_1_522	Bra016601	3	20.8	1	24	415	438	UCCGUCGUAGUCUAGCUGGUUAGG	CUGAAGAAGCUAGGCUGCGACGGA	Cleavage	1
trf_1_523	Bra016601	3	20.8	1	21	418	438	UCCGUCGUAGUCUAGCUGGUU	AAGAAGCUAGGCUGCGACGGA	Cleavage	1
trf_1_126	Bra018257	3	7.1	1	25	27	51	GACGGUUUGGCCGAGUGGUCUAAGG	CCCAAGACCACUCCUCGAAACCGUC	Translation	1
trf_1_31	Bra018620	3	8.3	1	20	7	26	GGGGAUGUAGCUCAGAUGGU	GCCACCUCAGCUACAUCCUC	Cleavage	1

trf_1_35	Bra018620	3	8.3	1	20	7	26	GGGGAUGUAGCUCAGAUGGU	GCCACCUCAGCUACAUCCUC	Cleavage	1
trf_1_60	Bra018620	3	8.3	1	20	7	26	GGGGAUGUAGCUCAGAUGGU	GCCACCUCAGCUACAUCCUC	Cleavage	1
trf_1_62	Bra018620	3	8.3	1	20	7	26	GGGGAUGUAGCUCAGAUGGU	GCCACCUCAGCUACAUCCUC	Cleavage	1
trf_1_90	Bra018620	3	8.3	1	20	7	26	GGGGAUGUAGCUCAGAUGGU	GCCACCUCAGCUACAUCCUC	Cleavage	1
trf_1_133	Bra018620	3	8.3	1	20	7	26	GGGGAUGUAGCUCAGAUGGU	GCCACCUCAGCUACAUCCUC	Cleavage	1
trf_1_137	Bra018620	3	8.3	1	20	7	26	GGGGAUGUAGCUCAGAUGGU	GCCACCUCAGCUACAUCCUC	Cleavage	1
trf_1_138	Bra018620	3	8.3	1	20	7	26	GGGGAUGUAGCUCAGAUGGU	GCCACCUCAGCUACAUCCUC	Cleavage	1
trf_1_153	Bra018620	3	8.3	1	20	7	26	GGGGAUGUAGCUCAGAUGGU	GCCACCUCAGCUACAUCCUC	Cleavage	1
trf_1_154	Bra018620	3	8.3	1	20	7	26	GGGGAUGUAGCUCAGAUGGU	GCCACCUCAGCUACAUCCUC	Cleavage	1
trf_1_160	Bra018620	3	8.3	1	20	7	26	GGGGAUGUAGCUCAGAUGGU	GCCACCUCAGCUACAUCCUC	Cleavage	1
trf_1_162	Bra018620	3	8.3	1	20	7	26	GGGGAUGUAGCUCAGAUGGU	GCCACCUCAGCUACAUCCUC	Cleavage	1
trf_1_169	Bra018620	3	8.3	1	20	7	26	GGGGAUGUAGCUCAGAUGGU	GCCACCUCAGCUACAUCCUC	Cleavage	1
trf_1_173	Bra018620	3	8.3	1	20	7	26	GGGGAUGUAGCUCAGAUGGU	GCCACCUCAGCUACAUCCUC	Cleavage	1
trf_1_183	Bra018620	3	8.3	1	20	7	26	GGGGAUGUAGCUCAGAUGGU	GCCACCUCAGCUACAUCCUC	Cleavage	1
trf_1_233	Bra018620	3	8.3	1	20	7	26	GGGGAUGUAGCUCAGAUGGU	GCCACCUCAGCUACAUCCUC	Cleavage	1
trf_1_236	Bra018620	3	8.3	1	20	7	26	GGGGAUGUAGCUCAGAUGGU	GCCACCUCAGCUACAUCCUC	Cleavage	1
trf_1_244	Bra018620	3	8.3	1	20	7	26	GGGGAUGUAGCUCAGAUGGU	GCCACCUCAGCUACAUCCUC	Cleavage	1

trf_1_2	Bra018							GGGGAUGUAGCUCAGA	GCCACCUCAGCUACAUC	Cleava	
47	620	3	8.3	1	20	7	26	UGGU	CUC	ge	1
trf_1_2	Bra018							GGGGAUGUAGCUCAGA	GCCACCUCAGCUACAUC	Cleava	
66	620	3	8.3	1	20	7	26	UGGU	CUC	ge	1
trf_1_2	Bra018							GGGGAUGUAGCUCAGA	GCCACCUCAGCUACAUC	Cleava	
86	620	3	8.3	1	20	7	26	UGGU	CUC	ge	1
trf_1_2	Bra018							GGGGAUGUAGCUCAGA	GCCACCUCAGCUACAUC	Cleava	
93	620	3	8.3	1	20	7	26	UGGU	CUC	ge	1
trf_1_2	Bra018							GGGGAUGUAGCUCAGA	GCCACCUCAGCUACAUC	Cleava	
97	620	3	8.3	1	20	7	26	UGGU	CUC	ge	1
trf_1_3	Bra018							GGGGAUGUAGCUCAGA	GCCACCUCAGCUACAUC	Cleava	
02	620	3	8.3	1	20	7	26	UGGU	CUC	ge	1
trf_1_3	Bra018							GGGGAUGUAGCUCAGA	GCCACCUCAGCUACAUC	Cleava	
07	620	3	8.3	1	20	7	26	UGGU	CUC	ge	1
trf_1_3	Bra018							GGGGAUGUAGCUCAGA	GCCACCUCAGCUACAUC	Cleava	
14	620	3	8.3	1	20	7	26	UGGU	CUC	ge	1
trf_1_3	Bra018							GGGGAUGUAGCUCAGA	GCCACCUCAGCUACAUC	Cleava	
22	620	3	8.3	1	20	7	26	UGGU	CUC	ge	1
trf_1_3	Bra018							GGGGAUGUAGCUCAGA	GCCACCUCAGCUACAUC	Cleava	
23	620	3	8.3	1	20	7	26	UGGU	CUC	ge	1
trf_1_3	Bra018							GGGGAUGUAGCUCAGA	GCCACCUCAGCUACAUC	Cleava	
32	620	3	8.3	1	20	7	26	UGGU	CUC	ge	1
trf_1_3	Bra018							GGGGAUGUAGCUCAGA	GCCACCUCAGCUACAUC	Cleava	
38	620	3	8.3	1	20	7	26	UGGU	CUC	ge	1
trf_1_3	Bra018							GGGGAUGUAGCUCAGA	GCCACCUCAGCUACAUC	Cleava	
41	620	3	8.3	1	20	7	26	UGGU	CUC	ge	1
trf_1_3	Bra018							GGGGAUGUAGCUCAGA	GCCACCUCAGCUACAUC	Cleava	
51	620	3	8.3	1	20	7	26	UGGU	CUC	ge	1
trf_1_3	Bra018							GGGGAUGUAGCUCAGA	GCCACCUCAGCUACAUC	Cleava	
53	620	3	8.3	1	20	7	26	UGGU	CUC	ge	1
trf_1_3	Bra018							GGGGAUGUAGCUCAGA	GCCACCUCAGCUACAUC	Cleava	
62	620	3	8.3	1	20	7	26	UGGU	CUC	ge	1
trf_1_3	Bra018							GGGGAUGUAGCUCAGA	GCCACCUCAGCUACAUC	Cleava	
66	620	3	8.3	1	20	7	26	UGGU	CUC	ge	1

trf_1_375	Bra018620	3	8.3	1	20	7	26	GGGGAUGUAGCUCAGAUGGU	GCCACCUCAGCUACAUCCUC	Cleavage	1
trf_1_379	Bra018620	3	8.3	1	20	7	26	GGGGAUGUAGCUCAGAUGGU	GCCACCUCAGCUACAUCCUC	Cleavage	1
trf_1_382	Bra018620	3	8.3	1	20	7	26	GGGGAUGUAGCUCAGAUGGU	GCCACCUCAGCUACAUCCUC	Cleavage	1
trf_1_383	Bra018620	3	8.3	1	20	7	26	GGGGAUGUAGCUCAGAUGGU	GCCACCUCAGCUACAUCCUC	Cleavage	1
trf_1_391	Bra018620	3	8.3	1	20	7	26	GGGGAUGUAGCUCAGAUGGU	GCCACCUCAGCUACAUCCUC	Cleavage	1
trf_1_392	Bra018620	3	8.3	1	20	7	26	GGGGAUGUAGCUCAGAUGGU	GCCACCUCAGCUACAUCCUC	Cleavage	1
trf_1_393	Bra018620	3	8.3	1	20	7	26	GGGGAUGUAGCUCAGAUGGU	GCCACCUCAGCUACAUCCUC	Cleavage	1
trf_1_400	Bra018620	3	8.3	1	20	7	26	GGGGAUGUAGCUCAGAUGGU	GCCACCUCAGCUACAUCCUC	Cleavage	1
trf_1_404	Bra018620	3	8.3	1	20	7	26	GGGGAUGUAGCUCAGAUGGU	GCCACCUCAGCUACAUCCUC	Cleavage	1
trf_1_408	Bra018620	3	8.3	1	20	7	26	GGGGAUGUAGCUCAGAUGGU	GCCACCUCAGCUACAUCCUC	Cleavage	1
trf_1_410	Bra018620	3	8.3	1	20	7	26	GGGGAUGUAGCUCAGAUGGU	GCCACCUCAGCUACAUCCUC	Cleavage	1
trf_1_482	Bra018620	3	8.3	1	20	7	26	GGGGAUGUAGCUCAGAUGGU	GCCACCUCAGCUACAUCCUC	Cleavage	1
trf_1_486	Bra018620	3	8.3	1	20	7	26	GGGGAUGUAGCUCAGAUGGU	GCCACCUCAGCUACAUCCUC	Cleavage	1
trf_1_489	Bra018620	3	8.3	1	20	7	26	GGGGAUGUAGCUCAGAUGGU	GCCACCUCAGCUACAUCCUC	Cleavage	1
trf_1_494	Bra018620	3	8.3	1	20	7	26	GGGGAUGUAGCUCAGAUGGU	GCCACCUCAGCUACAUCCUC	Cleavage	1
trf_1_498	Bra018620	3	8.3	1	20	7	26	GGGGAUGUAGCUCAGAUGGU	GCCACCUCAGCUACAUCCUC	Cleavage	1
trf_1_499	Bra018620	3	8.3	1	20	7	26	GGGGAUGUAGCUCAGAUGGU	GCCACCUCAGCUACAUCCUC	Cleavage	1

trf_1_182	Bra018971	3	15.6	1	22	132	153	CCGACCUUAGCUCAGUUGGUAG	CAACAAACUGGGUGAAGGUCGG	Translation	1
trf_1_476	Bra018971	3	15.6	1	22	132	153	CCGACCUUAGCUCAGUUGGUAG	CAACAAACUGGGUGAAGGUCGG	Translation	1
trf_1_478	Bra018971	3	15.6	1	22	132	153	CCGACCUUAGCUCAGUUGGUAG	CAACAAACUGGGUGAAGGUCGG	Translation	1
trf_1_479	Bra018971	3	15.6	1	22	132	153	CCGACCUUAGCUCAGUUGGUAG	CAACAAACUGGGUGAAGGUCGG	Translation	1
trf_1_520	Bra018971	3	15.6	1	22	132	153	CCGACCUUAGCUCAGUUGGUAG	CAACAAACUGGGUGAAGGUCGG	Translation	1
trf_1_577	Bra019452	2.5	16.5	1	20	970	989	UUAGGUUCAAAUCCUAUUGG	CCAAUUGGAUUUGAUCCUAA	Cleavage	1
trf_1_126	Bra019760	3	12.5	1	23	1328	1350	GACGGUUUGGCCGAGUGGUCUAA	UGAGACUACUUGGUCCAGCCGUC	Cleavage	1
trf_1_94	Bra020153	2.5	15.4	1	22	1953	1974	UCCGUUGUCGUCCAGCGGUUAG	CCAACUGCUGGAAGACAAUGGG	Translation	1
trf_1_95	Bra020153	2.5	15.4	1	22	1953	1974	UCCGUUGUCGUCCAGCGGUUAG	CCAACUGCUGGAAGACAAUGGG	Translation	1
trf_1_259	Bra020153	2.5	15.4	1	20	1955	1974	UCCGUUGUCGUCCAGCGGUU	AACUGCUGGAAGACAAUGGG	Translation	1
trf_1_261	Bra020153	2.5	15.4	1	22	1953	1974	UCCGUUGUCGUCCAGCGGUUAG	CCAACUGCUGGAAGACAAUGGG	Translation	1
trf_1_287	Bra020153	2.5	15.4	1	22	1953	1974	UCCGUUGUCGUCCAGCGGUUAG	CCAACUGCUGGAAGACAAUGGG	Translation	1
trf_1_288	Bra020153	2.5	15.4	1	22	1953	1974	UCCGUUGUCGUCCAGCGGUUAG	CCAACUGCUGGAAGACAAUGGG	Translation	1
trf_1_510	Bra020153	2.5	15.4	1	22	1953	1974	UCCGUUGUCGUCCAGCGGUUAG	CCAACUGCUGGAAGACAAUGGG	Translation	1
trf_1_511	Bra020153	2.5	15.4	1	22	1953	1974	UCCGUUGUCGUCCAGCGGUUAG	CCAACUGCUGGAAGACAAUGGG	Translation	1
trf_1_512	Bra020153	2.5	15.4	1	22	1953	1974	UCCGUUGUCGUCCAGCGGUUAG	CCAACUGCUGGAAGACAAUGGG	Translation	1
trf_1_5	Bra020755	3	12.6	1	20	454	473	GGGGAUGUAGCUCAAAUGGU	GCCAUUGGAGCUAUAUUCUC	Cleavage	1

trf_1_19	Bra020755	3	12.6	1	20	454	473	GGGGAUGUAGCUCAAAUGGU	GCCAUUGGAGCUAUAUUCUC	Cleavage	1
trf_1_30	Bra020755	3	12.6	1	20	454	473	GGGGAUGUAGCUCAAAUGGU	GCCAUUGGAGCUAUAUUCUC	Cleavage	1
trf_1_73	Bra020755	3	12.6	1	20	454	473	GGGGAUGUAGCUCAAAUGGU	GCCAUUGGAGCUAUAUUCUC	Cleavage	1
trf_1_116	Bra020755	3	12.6	1	20	454	473	GGGGAUGUAGCUCAAAUGGU	GCCAUUGGAGCUAUAUUCUC	Cleavage	1
trf_1_122	Bra020755	3	12.6	1	20	454	473	GGGGAUGUAGCUCAAAUGGU	GCCAUUGGAGCUAUAUUCUC	Cleavage	1
trf_1_130	Bra020755	3	12.6	1	20	454	473	GGGGAUGUAGCUCAAAUGGU	GCCAUUGGAGCUAUAUUCUC	Cleavage	1
trf_1_336	Bra020755	3	12.6	1	20	454	473	GGGGAUGUAGCUCAAAUGGU	GCCAUUGGAGCUAUAUUCUC	Cleavage	1
trf_1_519	Bra020755	3	12.6	1	20	454	473	GGGGAUGUAGCUCAAAUGGU	GCCAUUGGAGCUAUAUUCUC	Cleavage	1
trf_1_568	Bra020755	3	12.6	1	20	454	473	GGGGAUGUAGCUCAAAUGGU	GCCAUUGGAGCUAUAUUCUC	Cleavage	1
trf_1_17	Bra020835	3	9.5	1	22	5316	5337	GGGGGUGUAGCUCAUAUGGUAG	UUAUUACAUCAGCUACACCCCC	Cleavage	1
trf_1_18	Bra020835	3	9.5	1	22	5316	5337	GGGGGUGUAGCUCAUAUGGUAG	UUAUUACAUCAGCUACACCCCC	Cleavage	1
trf_1_43	Bra020835	3	9.5	1	22	5316	5337	GGGGGUGUAGCUCAUAUGGUAG	UUAUUACAUCAGCUACACCCCC	Cleavage	1
trf_1_264	Bra020835	3	9.5	1	22	5316	5337	GGGGGUGUAGCUCAUAUGGUAG	UUAUUACAUCAGCUACACCCCC	Cleavage	1
trf_1_398	Bra020835	3	9.5	1	22	5316	5337	GGGGGUGUAGCUCAUAUGGUAG	UUAUUACAUCAGCUACACCCCC	Cleavage	1
trf_1_578	Bra020978	3	12.8	1	20	173	192	AGGUUCAAAUCCUAUUGGAC	GUCCGAGAGGCUUUGGACCU	Translation	1
trf_1_414	Bra021314	3	23.2	1	20	234	253	GGAUUGUAGUUCAAUUGGU	ACCAACUGAACUGCAAUCUG	Cleavage	1
trf_1_415	Bra021314	3	23.2	1	20	234	253	GGAUUGUAGUUCAAUUGGU	ACCAACUGAACUGCAAUCUG	Cleavage	1

trf_1_416	Bra021314	3	23.2	1	20	234	253	GGGAUUGUAGUUCAAUUGGU	ACCAACUGAACUGCAAUCUG	Cleavage	1
trf_1_421	Bra021314	3	23.2	1	20	234	253	NGGAUUGUAGUUCAAUUGGU	ACCAACUGAACUGCAAUCUG	Cleavage	1
trf_1_422	Bra021314	3	23.2	1	20	234	253	GGGAUUGUAGUUCAAUUGGU	ACCAACUGAACUGCAAUCUG	Cleavage	1
trf_1_423	Bra021314	3	23.2	1	20	234	253	GGGAUUGUAGUUCAAUUGGU	ACCAACUGAACUGCAAUCUG	Cleavage	1
trf_1_424	Bra021314	3	23.2	1	20	234	253	GGGAUUGUAGUUCAAUUGGU	ACCAACUGAACUGCAAUCUG	Cleavage	1
trf_1_425	Bra021314	2.5	23.2	1	20	234	253	UGGAUUGUAGUUCAAUUGGU	ACCAACUGAACUGCAAUCUG	Cleavage	1
trf_1_426	Bra021314	3	23.2	1	20	234	253	GGGAUUGUAGUUCAAUUGGU	ACCAACUGAACUGCAAUCUG	Cleavage	1
trf_1_429	Bra021314	3	23.2	1	20	234	253	GGGAUUGUAGUUCAAUUGGU	ACCAACUGAACUGCAAUCUG	Cleavage	1
trf_1_431	Bra021314	3	23.2	1	20	234	253	GGGAUUGUAGUUCAAUUGGU	ACCAACUGAACUGCAAUCUG	Cleavage	1
trf_1_435	Bra021314	3	23.2	1	20	234	253	NGGAUUGUAGUUCAAUUGGU	ACCAACUGAACUGCAAUCUG	Cleavage	1
trf_1_438	Bra021314	3	23.2	1	20	234	253	GGGAUUGUAGUUCAAUUGGU	ACCAACUGAACUGCAAUCUG	Cleavage	1
trf_1_439	Bra021314	3	23.2	1	20	234	253	GGGAUUGUAGUUCAAUUGGU	ACCAACUGAACUGCAAUCUG	Cleavage	1
trf_1_440	Bra021314	3	23.2	1	20	234	253	GGGAUUGUAGUUCAAUUGGU	ACCAACUGAACUGCAAUCUG	Cleavage	1
trf_1_443	Bra021314	3	23.2	1	20	234	253	GGGAUUGUAGUUCAAUUGGU	ACCAACUGAACUGCAAUCUG	Cleavage	1
trf_1_444	Bra021314	3	23.2	1	20	234	253	GGGAUUGUAGUUCAAUUGGU	ACCAACUGAACUGCAAUCUG	Cleavage	1
trf_1_449	Bra021314	3	23.2	1	20	234	253	GGGAUUGUAGUUCAAUUGGU	ACCAACUGAACUGCAAUCUG	Cleavage	1
trf_1_450	Bra021314	3	23.2	1	20	234	253	GGGAUUGUAGUUCAAUUGGU	ACCAACUGAACUGCAAUCUG	Cleavage	1

trf_1_451	Bra021314	3	23.2	1	20	234	253	GGGAUUGUAGUUCAAUUGGU	ACCAACUGAACUGCAAUCUG	Cleavage	1
trf_1_453	Bra021314	3	23.2	1	20	234	253	GGGAUUGUAGUUCAAUUGGU	ACCAACUGAACUGCAAUCUG	Cleavage	1
trf_1_455	Bra021314	3	23.2	1	20	234	253	GGGAUUGUAGUUCAAUUGGU	ACCAACUGAACUGCAAUCUG	Cleavage	1
trf_1_457	Bra021314	3	23.2	1	20	234	253	GGGAUUGUAGUUCAAUUGGU	ACCAACUGAACUGCAAUCUG	Cleavage	1
trf_1_458	Bra021314	3	23.2	1	22	232	253	GGGAUUGUAGUUCAAUUGGUCU	ACACCAACUGAACUGCAAUCUG	Cleavage	1
trf_1_461	Bra021314	3	23.2	1	20	234	253	NGGAUUGUAGUUCAAUUGGU	ACCAACUGAACUGCAAUCUG	Cleavage	1
trf_1_462	Bra021314	2.5	23.2	1	20	234	253	UGGAUUGUAGUUCAAUUGGU	ACCAACUGAACUGCAAUCUG	Cleavage	1
trf_1_463	Bra021314	3	23.2	1	20	234	253	GGGAUUGUAGUUCAAUUGGU	ACCAACUGAACUGCAAUCUG	Cleavage	1
trf_1_466	Bra021314	2.5	23.2	1	20	234	253	UGGAUUGUAGUUCAAUUGGU	ACCAACUGAACUGCAAUCUG	Cleavage	1
trf_1_467	Bra021314	3	23.2	1	20	234	253	GGGAUUGUAGUUCAAUUGGU	ACCAACUGAACUGCAAUCUG	Cleavage	1
trf_1_468	Bra021314	3	23.2	1	26	227	253	GGGAUUGUAGUUCAAUUGGU-UAGAGC	GCUCAACACCAACUGAACUGCAAUCUG	Cleavage	1
trf_1_471	Bra021314	3	23.2	1	20	234	253	GGGAUUGUAGUUCAAUUGGU	ACCAACUGAACUGCAAUCUG	Cleavage	1
trf_1_577	Bra021441	3	14.8	1	21	759	779	UUAGGUUCAAAUCCUAUUGGA	UGCAAUAGGAUUUAGACCUAG	Cleavage	1
trf_1_579	Bra021441	3	14.7	1	20	759	778	UAGGUUCAAAUCCUAUUGGA	UGCAAUAGGAUUUAGACCUA	Cleavage	1
trf_1_105	Bra021555	3	19.4	1	22	946	967	ACCUACUUAACUCAGUGGUUAG	CUAACCAUGGCGUUAAGUGGGU	Cleavage	1
trf_1_529	Bra022251	3	23.9	1	20	3475	3494	GCUGGAGUAGCUCAGUUGGU	AUCAAUUGGGAUACUCUAGC	Translation	1
trf_1_32	Bra023165	3	19.6	1	20	229	248	GGGGAUAUAGCUCAGUUGGU	ACCACCGGACCUAUAUCCC	Translation	1

trf_1_33	Bra023165	3	19.6	1	20	229	248	GGGGAUAUAGCUCAGUUGGU	ACCACCGGACCUAUAUCCC	Translation	1
trf_1_34	Bra023165	3	19.6	1	20	229	248	GGGGAUAUAGCUCAGUUGGU	ACCACCGGACCUAUAUCCC	Translation	1
trf_1_36	Bra023165	3	19.6	1	20	229	248	GGGGAUAUAGCUCAGUUGGU	ACCACCGGACCUAUAUCCC	Translation	1
trf_1_59	Bra023165	3	19.6	1	20	229	248	GGGGAUAUAGCUCAGUUGGU	ACCACCGGACCUAUAUCCC	Translation	1
trf_1_61	Bra023165	3	19.6	1	20	229	248	GGGGAUAUAGCUCAGUUGGU	ACCACCGGACCUAUAUCCC	Translation	1
trf_1_63	Bra023165	3	19.6	1	20	229	248	GGGGAUAUAGCUCAGUUGGU	ACCACCGGACCUAUAUCCC	Translation	1
trf_1_64	Bra023165	3	19.6	1	20	229	248	GGGGAUAUAGCUCAGUUGGU	ACCACCGGACCUAUAUCCC	Translation	1
trf_1_89	Bra023165	3	19.6	1	20	229	248	GGGGAUAUAGCUCAGUUGGU	ACCACCGGACCUAUAUCCC	Translation	1
trf_1_91	Bra023165	3	19.6	1	20	229	248	GGGGAUAUAGCUCAGUUGGU	ACCACCGGACCUAUAUCCC	Translation	1
trf_1_92	Bra023165	3	19.6	1	20	229	248	GGGGAUAUAGCUCAGUUGGU	ACCACCGGACCUAUAUCCC	Translation	1
trf_1_93	Bra023165	3	19.6	1	20	229	248	GGGGAUAUAGCUCAGUUGGU	ACCACCGGACCUAUAUCCC	Translation	1
trf_1_134	Bra023165	3	19.6	1	20	229	248	GGGGAUAUAGCUCAGUUGGU	ACCACCGGACCUAUAUCCC	Translation	1
trf_1_135	Bra023165	3	19.6	1	20	229	248	GGGGAUAUAGCUCAGUUGGU	ACCACCGGACCUAUAUCCC	Translation	1
trf_1_136	Bra023165	3	19.6	1	20	229	248	GGGGAUAUAGCUCAGUUGGU	ACCACCGGACCUAUAUCCC	Translation	1
trf_1_139	Bra023165	3	19.6	1	20	229	248	GGGGAUAUAGCUCAGUUGGU	ACCACCGGACCUAUAUCCC	Translation	1
trf_1_151	Bra023165	3	19.6	1	20	229	248	GGGGAUAUAGCUCAGUUGGU	ACCACCGGACCUAUAUCCC	Translation	1
trf_1_152	Bra023165	3	19.6	1	20	229	248	GGGGAUAUAGCUCAGUUGGU	ACCACCGGACCUAUAUCCC	Translation	1

trf_1_155	Bra023165	3	19.6	1	20	229	248	GGGGAUAUAGCUCAGUUGGU	ACCACCGGACCUAUAUCCC	Translation	1
trf_1_156	Bra023165	3	19.6	1	20	229	248	GGGGAUAUAGCUCAGUUGGU	ACCACCGGACCUAUAUCCC	Translation	1
trf_1_161	Bra023165	3	19.6	1	20	229	248	GGGGAUAUAGCUCAGUUGGU	ACCACCGGACCUAUAUCCC	Translation	1
trf_1_163	Bra023165	3	19.6	1	20	229	248	GGGGAUAUAGCUCAGUUGGU	ACCACCGGACCUAUAUCCC	Translation	1
trf_1_164	Bra023165	3	19.6	1	20	229	248	GGGGAUAUAGCUCAGUUGGU	ACCACCGGACCUAUAUCCC	Translation	1
trf_1_165	Bra023165	3	19.6	1	20	229	248	GGGGAUAUAGCUCAGUUGGU	ACCACCGGACCUAUAUCCC	Translation	1
trf_1_166	Bra023165	3	19.6	1	20	229	248	GGGGAUAUAGCUCAGUUGGU	ACCACCGGACCUAUAUCCC	Translation	1
trf_1_168	Bra023165	3	19.6	1	20	229	248	GGGGAUAUAGCUCAGUUGGU	ACCACCGGACCUAUAUCCC	Translation	1
trf_1_170	Bra023165	3	19.6	1	20	229	248	GGGGAUAUAGCUCAGUUGGU	ACCACCGGACCUAUAUCCC	Translation	1
trf_1_171	Bra023165	3	19.6	1	20	229	248	GGGGAUAUAGCUCAGUUGGU	ACCACCGGACCUAUAUCCC	Translation	1
trf_1_172	Bra023165	3	19.6	1	20	229	248	GGGGAUAUAGCUCAGUUGGU	ACCACCGGACCUAUAUCCC	Translation	1
trf_1_184	Bra023165	3	19.6	1	20	229	248	GGGGAUAUAGCUCAGUUGGU	ACCACCGGACCUAUAUCCC	Translation	1
trf_1_185	Bra023165	3	19.6	1	20	229	248	GGGGAUAUAGCUCAGUUGGU	ACCACCGGACCUAUAUCCC	Translation	1
trf_1_186	Bra023165	3	19.6	1	20	229	248	GGGGAUAUAGCUCAGUUGGU	ACCACCGGACCUAUAUCCC	Translation	1
trf_1_187	Bra023165	3	19.6	1	20	229	248	GGGGAUAUAGCUCAGUUGGU	ACCACCGGACCUAUAUCCC	Translation	1
trf_1_234	Bra023165	3	19.6	1	20	229	248	GGGGAUAUAGCUCAGUUGGU	ACCACCGGACCUAUAUCCC	Translation	1
trf_1_235	Bra023165	3	19.6	1	20	229	248	GGGGAUAUAGCUCAGUUGGU	ACCACCGGACCUAUAUCCC	Translation	1

trf_1_237	Bra023165	3	19.6	1	20	229	248	GGGGAUAUAGCUCAGUUGGU	ACCACCGGACCUAUAUCCC	Translation	1
trf_1_245	Bra023165	3	19.6	1	20	229	248	GGGGAUAUAGCUCAGUUGGU	ACCACCGGACCUAUAUCCC	Translation	1
trf_1_246	Bra023165	3	19.6	1	20	229	248	GGGGAUAUAGCUCAGUUGGU	ACCACCGGACCUAUAUCCC	Translation	1
trf_1_248	Bra023165	3	19.6	1	20	229	248	GGGGAUAUAGCUCAGUUGGU	ACCACCGGACCUAUAUCCC	Translation	1
trf_1_265	Bra023165	3	19.6	1	20	229	248	GGGGAUAUAGCUCAGUUGGU	ACCACCGGACCUAUAUCCC	Translation	1
trf_1_267	Bra023165	3	19.6	1	20	229	248	GGGGAUAUAGCUCAGUUGGU	ACCACCGGACCUAUAUCCC	Translation	1
trf_1_268	Bra023165	3	19.6	1	20	229	248	GGGGAUAUAGCUCAGUUGGU	ACCACCGGACCUAUAUCCC	Translation	1
trf_1_269	Bra023165	3	19.6	1	20	229	248	GGGGAUAUAGCUCAGUUGGU	ACCACCGGACCUAUAUCCC	Translation	1
trf_1_270	Bra023165	3	19.6	1	20	229	248	GGGGAUAUAGCUCAGUUGGU	ACCACCGGACCUAUAUCCC	Translation	1
trf_1_283	Bra023165	3	19.6	1	20	229	248	GGGGAUAUAGCUCAGUUGGU	ACCACCGGACCUAUAUCCC	Translation	1
trf_1_284	Bra023165	3	19.6	1	20	229	248	GGGGAUAUAGCUCAGUUGGU	ACCACCGGACCUAUAUCCC	Translation	1
trf_1_285	Bra023165	3	19.6	1	20	229	248	GGGGAUAUAGCUCAGUUGGU	ACCACCGGACCUAUAUCCC	Translation	1
trf_1_294	Bra023165	3	19.6	1	20	229	248	GGGGAUAUAGCUCAGUUGGU	ACCACCGGACCUAUAUCCC	Translation	1
trf_1_295	Bra023165	3	19.6	1	20	229	248	GGGGAUAUAGCUCAGUUGGU	ACCACCGGACCUAUAUCCC	Translation	1
trf_1_296	Bra023165	3	19.6	1	20	229	248	GGGGAUAUAGCUCAGUUGGU	ACCACCGGACCUAUAUCCC	Translation	1
trf_1_298	Bra023165	3	19.6	1	20	229	248	GGGGAUAUAGCUCAGUUGGU	ACCACCGGACCUAUAUCCC	Translation	1
trf_1_301	Bra023165	3	19.6	1	20	229	248	GGGGAUAUAGCUCAGUUGGU	ACCACCGGACCUAUAUCCC	Translation	1

trf_1_303	Bra023165	3	19.6	1	20	229	248	GGGGAUAUAGCUCAGUUGGU	ACCACCGGACCUAUAUCCCC	Translation	1
trf_1_304	Bra023165	3	19.6	1	20	229	248	GGGGAUAUAGCUCAGUUGGU	ACCACCGGACCUAUAUCCCC	Translation	1
trf_1_305	Bra023165	3	19.6	1	20	229	248	GGGGAUAUAGCUCAGUUGGU	ACCACCGGACCUAUAUCCCC	Translation	1
trf_1_308	Bra023165	3	19.6	1	20	229	248	GGGGAUAUAGCUCAGUUGGU	ACCACCGGACCUAUAUCCCC	Translation	1
trf_1_309	Bra023165	3	19.6	1	20	229	248	GGGGAUAUAGCUCAGUUGGU	ACCACCGGACCUAUAUCCCC	Translation	1
trf_1_310	Bra023165	3	19.6	1	20	229	248	GGGGAUAUAGCUCAGUUGGU	ACCACCGGACCUAUAUCCCC	Translation	1
trf_1_315	Bra023165	3	19.6	1	20	229	248	GGGGAUAUAGCUCAGUUGGU	ACCACCGGACCUAUAUCCCC	Translation	1
trf_1_316	Bra023165	3	19.6	1	20	229	248	GGGGAUAUAGCUCAGUUGGU	ACCACCGGACCUAUAUCCCC	Translation	1
trf_1_317	Bra023165	3	19.6	1	20	229	248	GGGGAUAUAGCUCAGUUGGU	ACCACCGGACCUAUAUCCCC	Translation	1
trf_1_318	Bra023165	3	19.6	1	20	229	248	GGGGAUAUAGCUCAGUUGGU	ACCACCGGACCUAUAUCCCC	Translation	1
trf_1_321	Bra023165	3	19.6	1	20	229	248	GGGGAUAUAGCUCAGUUGGU	ACCACCGGACCUAUAUCCCC	Translation	1
trf_1_324	Bra023165	3	19.6	1	20	229	248	GGGGAUAUAGCUCAGUUGGU	ACCACCGGACCUAUAUCCCC	Translation	1
trf_1_325	Bra023165	3	19.6	1	20	229	248	GGGGAUAUAGCUCAGUUGGU	ACCACCGGACCUAUAUCCCC	Translation	1
trf_1_333	Bra023165	3	19.6	1	20	229	248	GGGGAUAUAGCUCAGUUGGU	ACCACCGGACCUAUAUCCCC	Translation	1
trf_1_334	Bra023165	3	19.6	1	20	229	248	GGGGAUAUAGCUCAGUUGGU	ACCACCGGACCUAUAUCCCC	Translation	1
trf_1_335	Bra023165	3	19.6	1	20	229	248	GGGGAUAUAGCUCAGUUGGU	ACCACCGGACCUAUAUCCCC	Translation	1
trf_1_339	Bra023165	3	19.6	1	20	229	248	GGGGAUAUAGCUCAGUUGGU	ACCACCGGACCUAUAUCCCC	Translation	1

trf_1_340	Bra023165	3	19.6	1	20	229	248	GGGGAUAUAGCUCAGUUGGU	ACCACCGGACCUAUAUCCC	Translation	1
trf_1_342	Bra023165	3	19.6	1	20	229	248	GGGGAUAUAGCUCAGUUGGU	ACCACCGGACCUAUAUCCC	Translation	1
trf_1_343	Bra023165	3	19.6	1	20	229	248	GGGGAUAUAGCUCAGUUGGU	ACCACCGGACCUAUAUCCC	Translation	1
trf_1_344	Bra023165	3	19.6	1	20	229	248	GGGGAUAUAGCUCAGUUGGU	ACCACCGGACCUAUAUCCC	Translation	1
trf_1_352	Bra023165	3	19.6	1	20	229	248	GGGGAUAUAGCUCAGUUGGU	ACCACCGGACCUAUAUCCC	Translation	1
trf_1_354	Bra023165	3	19.6	1	20	229	248	GGGGAUAUAGCUCAGUUGGU	ACCACCGGACCUAUAUCCC	Translation	1
trf_1_355	Bra023165	3	19.6	1	20	229	248	GGGGAUAUAGCUCAGUUGGU	ACCACCGGACCUAUAUCCC	Translation	1
trf_1_356	Bra023165	3	19.6	1	20	229	248	GGGGAUAUAGCUCAGUUGGU	ACCACCGGACCUAUAUCCC	Translation	1
trf_1_357	Bra023165	3	19.6	1	20	229	248	GGGGAUAUAGCUCAGUUGGU	ACCACCGGACCUAUAUCCC	Translation	1
trf_1_358	Bra023165	3	19.6	1	20	229	248	GGGGAUAUAGCUCAGUUGGU	ACCACCGGACCUAUAUCCC	Translation	1
trf_1_361	Bra023165	3	19.6	1	20	229	248	GGGGAUAUAGCUCAGUUGGU	ACCACCGGACCUAUAUCCC	Translation	1
trf_1_363	Bra023165	3	19.6	1	20	229	248	GGGGAUAUAGCUCAGUUGGU	ACCACCGGACCUAUAUCCC	Translation	1
trf_1_364	Bra023165	3	19.6	1	20	229	248	GGGGAUAUAGCUCAGUUGGU	ACCACCGGACCUAUAUCCC	Translation	1
trf_1_365	Bra023165	3	19.6	1	20	229	248	GGGGAUAUAGCUCAGUUGGU	ACCACCGGACCUAUAUCCC	Translation	1
trf_1_376	Bra023165	3	19.6	1	20	229	248	GGGGAUAUAGCUCAGUUGGU	ACCACCGGACCUAUAUCCC	Translation	1
trf_1_377	Bra023165	3	19.6	1	20	229	248	GGGGAUAUAGCUCAGUUGGU	ACCACCGGACCUAUAUCCC	Translation	1
trf_1_378	Bra023165	3	19.6	1	20	229	248	GGGGAUAUAGCUCAGUUGGU	ACCACCGGACCUAUAUCCC	Translation	1

trf_1_380	Bra023165	3	19.6	1	20	229	248	GGGGAUAUAGCUCAGUUGGU	ACCACCGGACCUAUAUCCCC	Translation	1
trf_1_384	Bra023165	3	19.6	1	20	229	248	GGGGAUAUAGCUCAGUUGGU	ACCACCGGACCUAUAUCCCC	Translation	1
trf_1_385	Bra023165	3	19.6	1	20	229	248	GGGGAUAUAGCUCAGUUGGU	ACCACCGGACCUAUAUCCCC	Translation	1
trf_1_386	Bra023165	3	19.6	1	20	229	248	GGGGAUAUAGCUCAGUUGGU	ACCACCGGACCUAUAUCCCC	Translation	1
trf_1_387	Bra023165	3	19.6	1	20	229	248	GGGGAUAUAGCUCAGUUGGU	ACCACCGGACCUAUAUCCCC	Translation	1
trf_1_394	Bra023165	3	19.6	1	20	229	248	GGGGAUAUAGCUCAGUUGGU	ACCACCGGACCUAUAUCCCC	Translation	1
trf_1_395	Bra023165	3	19.6	1	20	229	248	GGGGAUAUAGCUCAGUUGGU	ACCACCGGACCUAUAUCCCC	Translation	1
trf_1_396	Bra023165	3	19.6	1	20	229	248	GGGGAUAUAGCUCAGUUGGU	ACCACCGGACCUAUAUCCCC	Translation	1
trf_1_397	Bra023165	3	19.6	1	20	229	248	GGGGAUAUAGCUCAGUUGGU	ACCACCGGACCUAUAUCCCC	Translation	1
trf_1_401	Bra023165	3	19.6	1	20	229	248	GGGGAUAUAGCUCAGUUGGU	ACCACCGGACCUAUAUCCCC	Translation	1
trf_1_402	Bra023165	3	19.6	1	20	229	248	GGGGAUAUAGCUCAGUUGGU	ACCACCGGACCUAUAUCCCC	Translation	1
trf_1_403	Bra023165	3	19.6	1	20	229	248	GGGGAUAUAGCUCAGUUGGU	ACCACCGGACCUAUAUCCCC	Translation	1
trf_1_405	Bra023165	3	19.6	1	20	229	248	GGGGAUAUAGCUCAGUUGGU	ACCACCGGACCUAUAUCCCC	Translation	1
trf_1_406	Bra023165	3	19.6	1	20	229	248	GGGGAUAUAGCUCAGUUGGU	ACCACCGGACCUAUAUCCCC	Translation	1
trf_1_407	Bra023165	3	19.6	1	20	229	248	GGGGAUAUAGCUCAGUUGGU	ACCACCGGACCUAUAUCCCC	Translation	1
trf_1_409	Bra023165	3	19.6	1	20	229	248	GGGGAUAUAGCUCAGUUGGU	ACCACCGGACCUAUAUCCCC	Translation	1
trf_1_411	Bra023165	3	19.6	1	20	229	248	GGGGAUAUAGCUCAGUUGGU	ACCACCGGACCUAUAUCCCC	Translation	1

trf_1_483	Bra023165	3	19.6	1	20	229	248	GGGGAUAUAGCUCAGUUGGU	ACCACCGGACCUAUAUCCC	Translation	1
trf_1_484	Bra023165	3	19.6	1	20	229	248	GGGGAUAUAGCUCAGUUGGU	ACCACCGGACCUAUAUCCC	Translation	1
trf_1_485	Bra023165	3	19.6	1	20	229	248	GGGGAUAUAGCUCAGUUGGU	ACCACCGGACCUAUAUCCC	Translation	1
trf_1_487	Bra023165	3	19.6	1	20	229	248	GGGGAUAUAGCUCAGUUGGU	ACCACCGGACCUAUAUCCC	Translation	1
trf_1_488	Bra023165	3	19.6	1	20	229	248	GGGGAUAUAGCUCAGUUGGU	ACCACCGGACCUAUAUCCC	Translation	1
trf_1_490	Bra023165	3	19.6	1	20	229	248	GGGGAUAUAGCUCAGUUGGU	ACCACCGGACCUAUAUCCC	Translation	1
trf_1_491	Bra023165	3	19.6	1	20	229	248	GGGGAUAUAGCUCAGUUGGU	ACCACCGGACCUAUAUCCC	Translation	1
trf_1_492	Bra023165	3	19.6	1	20	229	248	GGGGAUAUAGCUCAGUUGGU	ACCACCGGACCUAUAUCCC	Translation	1
trf_1_493	Bra023165	3	19.6	1	20	229	248	GGGGAUAUAGCUCAGUUGGU	ACCACCGGACCUAUAUCCC	Translation	1
trf_1_495	Bra023165	3	19.6	1	20	229	248	GGGGAUAUAGCUCAGUUGGU	ACCACCGGACCUAUAUCCC	Translation	1
trf_1_500	Bra023165	3	19.6	1	20	229	248	GGGGAUAUAGCUCAGUUGGU	ACCACCGGACCUAUAUCCC	Translation	1
trf_1_501	Bra023165	3	19.6	1	20	229	248	GGGGAUAUAGCUCAGUUGGU	ACCACCGGACCUAUAUCCC	Translation	1
trf_1_502	Bra023165	3	19.6	1	20	229	248	GGGGAUAUAGCUCAGUUGGU	ACCACCGGACCUAUAUCCC	Translation	1
trf_1_503	Bra023165	3	19.6	1	20	229	248	GGGGAUAUAGCUCAGUUGGU	ACCACCGGACCUAUAUCCC	Translation	1
trf_1_504	Bra023165	3	19.6	1	20	229	248	GGGGAUAUAGCUCAGUUGGU	ACCACCGGACCUAUAUCCC	Translation	1
trf_1_418	Bra023172	3	12.4	1	20	614	633	GGGAUUGUAGUUCAAUUGGA	UCCAAGUGAACAUCAAUCCC	Translation	1
trf_1_419	Bra023172	3	12.4	1	21	613	633	GGGAUUGUAGUUCAAUUGGAA	UUCCAAGUGAACAUCAAUCCC	Translation	1

trf_1_434	Bra023172	3	12.4	1	20	614	633	GGGAUUGUAGUUCAAUUGGA	UCCAAGUGAACAUCAAUCCC	Translation	1
trf_1_445	Bra023172	3	12.4	1	22	612	633	GGGAUUGUAGUUCAAUUGGAAA	UUUCCAAGUGAACAUCAAUCCC	Translation	1
trf_1_447	Bra023172	3	12.4	1	20	614	633	GGGAUUGUAGUUCAAUUGGA	UCCAAGUGAACAUCAAUCCC	Translation	1
trf_1_456	Bra023172	3	12.4	1	22	612	633	GGGAUUGUAGUUCAAUUGGAGA	UUUCCAAGUGAACAUCAAUCCC	Translation	1
trf_1_465	Bra023172	3	12.4	1	21	613	633	GGGAUUGUAGUUCAAUUGGAG	UUCCAAGUGAACAUCAAUCCC	Translation	1
trf_1_477	Bra023904	3	9.9	1	20	1163	1182	GGUUCUAUGGUGUAGUGGUU	AACCAAUACAUGAUAGAAUC	Translation	1
trf_1_539	Bra023904	3	9.9	1	20	1163	1182	GGUUCUAUGGUGUAGUGGUU	AACCAAUACAUGAUAGAAUC	Translation	1
trf_1_14	Bra024084	3	22.2	1	20	1249	1268	GGGGAUAUAGCUCAGUUGGG	UCCAACAGAGUUGUAUCCUC	Cleavage	1
trf_1_271	Bra024316	3	20.4	1	25	431	455	GCGUCCAUUGUCUAAUGGAUAGGAC	GUUCUGUCCAUCAGAUACUGGACGC	Cleavage	1
trf_1_272	Bra024316	3	20.4	1	24	432	455	GCGUCCAUUGUCUAAUGGAUAGGA	UUCUGUCCAUCAGAUACUGGACGC	Cleavage	1
trf_1_273	Bra024316	3	20.4	1	25	431	455	GCGUCCAUUGUCUAAUGGAUAGGAC	GUUCUGUCCAUCAGAUACUGGACGC	Cleavage	1
trf_1_274	Bra024316	3	20.4	1	20	436	455	GCGUCCAUUGUCUAAUGGAU	GUCCAUCAGAUACUGGACGC	Cleavage	1
trf_1_275	Bra024316	3	20.4	1	23	433	455	GCGUCCAUUGUCUAAUGGAUAGG	UCUGUCCAUCAGAUACUGGACGC	Cleavage	1
trf_1_276	Bra024316	3	20.4	1	27	429	455	GCGUCCAUUGUCUAAUGGAUAGGACAU	AGGUUCUGUCCAUCAGAUACUGGACGC	Cleavage	1
trf_1_278	Bra024316	3	20.4	1	21	435	455	GCGUCCAUUGUCUAAUGGAUA	UGUCCAUCAGAUACUGGACGC	Cleavage	1
trf_1_279	Bra024316	3	20.4	1	22	434	455	GCGUCCAUUGUCUAAUGGAUAG	CUGUCCAUCAGAUACUGGACGC	Cleavage	1
trf_1_2	Bra024638	3	7.5	1	23	203	225	GGGGAUGUAGCUCAAAUGGUAGA	UCCAACAUUUCGGUUACAUCCCC	Cleavage	1

trf_1_3	Bra024638	3	7.5	1	24	202	225	GGGGAUGUAGCUCAAAUGGUAGAG	UUCCAACAUUUCGGUUACAUCCCC	Cleavage	1
trf_1_5	Bra024638	3	7.5	1	20	206	225	GGGGAUGUAGCUCAAAUGGU	AACAUUUCGGUUACAUCCCC	Cleavage	1
trf_1_6	Bra024638	3	7.5	1	23	203	225	GGGGAUGUAGCUCAAAUGGUAGA	UCCAACAUUUCGGUUACAUCCCC	Cleavage	1
trf_1_19	Bra024638	3	7.5	1	20	206	225	GGGGAUGUAGCUCAAAUGGU	AACAUUUCGGUUACAUCCCC	Cleavage	1
trf_1_20	Bra024638	3	7.5	1	23	203	225	GGGGAUGUAGCUCAAAUGGUAGA	UCCAACAUUUCGGUUACAUCCCC	Cleavage	1
trf_1_29	Bra024638	3	7.5	1	23	203	225	GGGGAUGUAGCUCAAAUGGUAGA	UCCAACAUUUCGGUUACAUCCCC	Cleavage	1
trf_1_30	Bra024638	3	7.5	1	20	206	225	GGGGAUGUAGCUCAAAUGGU	AACAUUUCGGUUACAUCCCC	Cleavage	1
trf_1_72	Bra024638	3	7.5	1	23	203	225	GGGGAUGUAGCUCAAAUGGUAGA	UCCAACAUUUCGGUUACAUCCCC	Cleavage	1
trf_1_73	Bra024638	3	7.5	1	20	206	225	GGGGAUGUAGCUCAAAUGGU	AACAUUUCGGUUACAUCCCC	Cleavage	1
trf_1_116	Bra024638	3	7.5	1	20	206	225	GGGGAUGUAGCUCAAAUGGU	AACAUUUCGGUUACAUCCCC	Cleavage	1
trf_1_117	Bra024638	3	7.5	1	23	203	225	GGGGAUGUAGCUCAAAUGGUAGA	UCCAACAUUUCGGUUACAUCCCC	Cleavage	1
trf_1_118	Bra024638	3	7.5	1	24	202	225	GGGGAUGUAGCUCAAAUGGUAGAG	UUCCAACAUUUCGGUUACAUCCCC	Cleavage	1
trf_1_121	Bra024638	3	7.5	1	23	203	225	GGGGAUGUAGCUCAAAUGGUAGA	UCCAACAUUUCGGUUACAUCCCC	Cleavage	1
trf_1_122	Bra024638	3	7.5	1	20	206	225	GGGGAUGUAGCUCAAAUGGU	AACAUUUCGGUUACAUCCCC	Cleavage	1
trf_1_129	Bra024638	3	7.5	1	23	203	225	GGGGAUGUAGCUCAAAUGGUAGA	UCCAACAUUUCGGUUACAUCCCC	Cleavage	1
trf_1_130	Bra024638	3	7.5	1	20	206	225	GGGGAUGUAGCUCAAAUGGU	AACAUUUCGGUUACAUCCCC	Cleavage	1
trf_1_336	Bra024638	3	7.5	1	20	206	225	GGGGAUGUAGCUCAAAUGGU	AACAUUUCGGUUACAUCCCC	Cleavage	1

trf_1_337	Bra024638	3	7.5	1	23	203	225	GGGGAUGUAGCUCAAAUGGUAGA	UCCAACAUUUCGGUUACAUCCCC	Cleavage	1
trf_1_367	Bra024638	3	7.5	1	23	203	225	GGGGAUGUAGCUCAAAUGGUAGA	UCCAACAUUUCGGUUACAUCCCC	Cleavage	1
trf_1_368	Bra024638	3	7.5	1	24	202	225	GGGGAUGUAGCUCAAAUGGUAGAG	UUCCAACAUUUCGGUUACAUCCCC	Cleavage	1
trf_1_517	Bra024638	3	7.5	1	23	203	225	GGGGAUGUAGCUCAAAUGGUAGA	UCCAACAUUUCGGUUACAUCCCC	Cleavage	1
trf_1_518	Bra024638	3	7.5	1	24	202	225	GGGGAUGUAGCUCAAAUGGUAGAG	UUCCAACAUUUCGGUUACAUCCCC	Cleavage	1
trf_1_519	Bra024638	3	7.5	1	20	206	225	GGGGAUGUAGCUCAAAUGGU	AACAUUUCGGUUACAUCCCC	Cleavage	1
trf_1_566	Bra024638	3	7.5	1	23	203	225	GGGGAUGUAGCUCAAAUGGUAGA	UCCAACAUUUCGGUUACAUCCCC	Cleavage	1
trf_1_567	Bra024638	3	7.5	1	24	202	225	GGGGAUGUAGCUCAAAUGGUAGAG	UUCCAACAUUUCGGUUACAUCCCC	Cleavage	1
trf_1_568	Bra024638	3	7.5	1	20	206	225	GGGGAUGUAGCUCAAAUGGU	AACAUUUCGGUUACAUCCCC	Cleavage	1
trf_1_2	Bra025904	3	17.2	1	20	344	363	GGGGAUGUAGCUCAAAUGGU	ACAAUUUGAGCUUCAUCUUC	Cleavage	1
trf_1_3	Bra025904	3	17.2	1	20	344	363	GGGGAUGUAGCUCAAAUGGU	ACAAUUUGAGCUUCAUCUUC	Cleavage	1
trf_1_5	Bra025904	3	17.2	1	20	344	363	GGGGAUGUAGCUCAAAUGGU	ACAAUUUGAGCUUCAUCUUC	Cleavage	1
trf_1_6	Bra025904	3	17.2	1	20	344	363	GGGGAUGUAGCUCAAAUGGU	ACAAUUUGAGCUUCAUCUUC	Cleavage	1
trf_1_19	Bra025904	3	17.2	1	20	344	363	GGGGAUGUAGCUCAAAUGGU	ACAAUUUGAGCUUCAUCUUC	Cleavage	1
trf_1_20	Bra025904	3	17.2	1	20	344	363	GGGGAUGUAGCUCAAAUGGU	ACAAUUUGAGCUUCAUCUUC	Cleavage	1
trf_1_29	Bra025904	3	17.2	1	20	344	363	GGGGAUGUAGCUCAAAUGGU	ACAAUUUGAGCUUCAUCUUC	Cleavage	1
trf_1_30	Bra025904	3	17.2	1	20	344	363	GGGGAUGUAGCUCAAAUGGU	ACAAUUUGAGCUUCAUCUUC	Cleavage	1

trf_1_72	Bra025904	3	17.2	1	20	344	363	GGGGAUGUAGCUCAAAUGGU	ACAAUUUGAGCUUCAUCUUC	Cleavage	1
trf_1_73	Bra025904	3	17.2	1	20	344	363	GGGGAUGUAGCUCAAAUGGU	ACAAUUUGAGCUUCAUCUUC	Cleavage	1
trf_1_116	Bra025904	3	17.2	1	20	344	363	GGGGAUGUAGCUCAAAUGGU	ACAAUUUGAGCUUCAUCUUC	Cleavage	1
trf_1_117	Bra025904	3	17.2	1	20	344	363	GGGGAUGUAGCUCAAAUGGU	ACAAUUUGAGCUUCAUCUUC	Cleavage	1
trf_1_118	Bra025904	3	17.2	1	20	344	363	GGGGAUGUAGCUCAAAUGGU	ACAAUUUGAGCUUCAUCUUC	Cleavage	1
trf_1_121	Bra025904	3	17.2	1	20	344	363	GGGGAUGUAGCUCAAAUGGU	ACAAUUUGAGCUUCAUCUUC	Cleavage	1
trf_1_122	Bra025904	3	17.2	1	20	344	363	GGGGAUGUAGCUCAAAUGGU	ACAAUUUGAGCUUCAUCUUC	Cleavage	1
trf_1_129	Bra025904	3	17.2	1	20	344	363	GGGGAUGUAGCUCAAAUGGU	ACAAUUUGAGCUUCAUCUUC	Cleavage	1
trf_1_130	Bra025904	3	17.2	1	20	344	363	GGGGAUGUAGCUCAAAUGGU	ACAAUUUGAGCUUCAUCUUC	Cleavage	1
trf_1_336	Bra025904	3	17.2	1	20	344	363	GGGGAUGUAGCUCAAAUGGU	ACAAUUUGAGCUUCAUCUUC	Cleavage	1
trf_1_337	Bra025904	3	17.2	1	20	344	363	GGGGAUGUAGCUCAAAUGGU	ACAAUUUGAGCUUCAUCUUC	Cleavage	1
trf_1_367	Bra025904	3	17.2	1	20	344	363	GGGGAUGUAGCUCAAAUGGU	ACAAUUUGAGCUUCAUCUUC	Cleavage	1
trf_1_368	Bra025904	3	17.2	1	20	344	363	GGGGAUGUAGCUCAAAUGGU	ACAAUUUGAGCUUCAUCUUC	Cleavage	1
trf_1_517	Bra025904	3	17.2	1	20	344	363	GGGGAUGUAGCUCAAAUGGU	ACAAUUUGAGCUUCAUCUUC	Cleavage	1
trf_1_518	Bra025904	3	17.2	1	20	344	363	GGGGAUGUAGCUCAAAUGGU	ACAAUUUGAGCUUCAUCUUC	Cleavage	1
trf_1_519	Bra025904	3	17.2	1	20	344	363	GGGGAUGUAGCUCAAAUGGU	ACAAUUUGAGCUUCAUCUUC	Cleavage	1
trf_1_566	Bra025904	3	17.2	1	20	344	363	GGGGAUGUAGCUCAAAUGGU	ACAAUUUGAGCUUCAUCUUC	Cleavage	1

trf_1_567	Bra025904	3	17.2	1	20	344	363	GGGGAUGUAGCUCAAAUGGU	ACAAUUUGAGCUUCAUCUUC	Cleavage	1
trf_1_568	Bra025904	3	17.2	1	20	344	363	GGGGAUGUAGCUCAAAUGGU	ACAAUUUGAGCUUCAUCUUC	Cleavage	1
trf_1_208	Bra026093	3	24.7	1	20	454	473	GGUCGAUGCCCGAGCGGAG	CGCCGCUCGGGAUCGACUU	Translation	1
trf_1_212	Bra026093	3	24.7	1	20	454	473	GGUCGAUGCCCGAGCGGCU	CGCCGCUCGGGAUCGACUU	Translation	1
trf_1_158	Bra027297	3	22.9	1	20	178	197	GCCGACUUAGCUCAGUGGUA	UACCACAGGGCGAAGUUGGC	Translation	1
trf_1_350	Bra027297	3	22.9	1	20	178	197	GCCGACUUAGCUCAGUGGUA	UACCACAGGGCGAAGUUGGC	Translation	1
trf_1_208	Bra027479	2.5	22.7	1	20	193	212	GGUCGAUGCCCGAGCGGAG	UUCUGCUCCGGCAUCGACCU	Cleavage	1
trf_1_214	Bra027479	3	22.7	1	20	193	212	GGUCGAUGCCCGAGCGGAU	UUCUGCUCCGGCAUCGACCU	Cleavage	1
trf_1_216	Bra027479	2.5	22.7	1	20	193	212	GGUCGAUGCCCGAGCGGGA	UUCUGCUCCGGCAUCGACCU	Cleavage	1
trf_1_217	Bra027479	2	22.7	1	20	193	212	GGUCGAUGCCCGAGCGGAA	UUCUGCUCCGGCAUCGACCU	Cleavage	1
trf_1_221	Bra027479	2	22.7	1	20	193	212	GGUCGAUGCCCGAGCGGAA	UUCUGCUCCGGCAUCGACCU	Cleavage	1
trf_1_428	Bra028504	3	17.0	1	20	1007	1026	GGAUCGUAGUUCAAUUGGU	ACCCUUUGAACUAUGAUUCC	Cleavage	1
trf_1_217	Bra029587	3	16.1	1	21	1011	1031	GGUCGAUGCCCGAGCGGAAA	UUUUCGCUCGGGCCUUGACCG	Cleavage	1
trf_1_221	Bra029587	3	16.1	1	20	1012	1031	GGUCGAUGCCCGAGCGGAA	UUUCGCUCGGGCCUUGACCG	Cleavage	1
trf_1_529	Bra029975	3	20.8	1	21	1244	1264	GCUGGAGUAGCUCAGUUGGUU	GGUCAGCUGAGCUACUCAAGC	Cleavage	1
trf_1_208	Bra030030	3	6.8	1	20	340	359	GGUCGAUGCCCGAGCGGAG	CUCCGCUCCGCCGUCGACUC	Translation	1
trf_1_14	Bra030524	3	14.1	1	20	2271	2290	GGGGAUAUAGCUCAGUUGGG	UCCAAUUGAUCUUUAUCCCC	Translation	1

trf_1_8	Bra032111	3	22.4	1	20	727	746	UCCGUUAUCGUCCAGCGGUU	GACGGCUUGACGAUGACGGA	Cleavage	1
trf_1_9	Bra032111	3	22.4	1	20	727	746	UCCGUUAUCGUCCAGCGGUU	GACGGCUUGACGAUGACGGA	Cleavage	1
trf_1_10	Bra032111	3	22.4	1	20	727	746	UCCGUUAUCGUCCAGCGGUU	GACGGCUUGACGAUGACGGA	Cleavage	1
trf_1_231	Bra032111	3	22.4	1	20	727	746	UCCGUUAUCGUCCAGCGGUU	GACGGCUUGACGAUGACGGA	Cleavage	1
trf_1_232	Bra032111	3	22.4	1	20	727	746	UCCGUUAUCGUCCAGCGGUU	GACGGCUUGACGAUGACGGA	Cleavage	1
trf_1_529	Bra032168	2.5	22.3	1	20	150	169	GCUGGAGUAGCUCAGUUGGU	AUCGGCUGAGCCACUCCAGC	Translation	1
trf_1_580	Bra033006	3	19.3	1	20	787	805	GGUUCAAAUCCUAUUGGACG	UGUCCAA-AGGGUUUGAACC	Cleavage	1
trf_1_258	Bra033352	3	18.3	1	25	586	609	AUCAGAGUGGCGCAGCGGAAGCGUG	CACG-UUACGUUGCACCACUUUGAU	Translation	1
trf_1_569	Bra033352	3	18.3	1	25	586	609	AUCAGAGUGGCGCAGCGGAAGCGUG	CACG-UUACGUUGCACCACUUUGAU	Translation	1
trf_1_2	Bra033370	3	21.6	1	20	4750	4769	GGGGAUGUAGCUCAAAUGGU	ACCCUUUGGGUAACAUCCCC	Translation	1
trf_1_3	Bra033370	3	21.6	1	24	4746	4769	GGGGAUGUAGCUCAAAUGGUAGAG	CUGGACCCUUUGGGUAACAUCCCC	Translation	1
trf_1_5	Bra033370	3	21.6	1	20	4750	4769	GGGGAUGUAGCUCAAAUGGU	ACCCUUUGGGUAACAUCCCC	Translation	1
trf_1_6	Bra033370	3	21.6	1	20	4750	4769	GGGGAUGUAGCUCAAAUGGU	ACCCUUUGGGUAACAUCCCC	Translation	1
trf_1_19	Bra033370	3	21.6	1	20	4750	4769	GGGGAUGUAGCUCAAAUGGU	ACCCUUUGGGUAACAUCCCC	Translation	1
trf_1_20	Bra033370	3	21.6	1	20	4750	4769	GGGGAUGUAGCUCAAAUGGU	ACCCUUUGGGUAACAUCCCC	Translation	1
trf_1_29	Bra033370	3	21.6	1	20	4750	4769	GGGGAUGUAGCUCAAAUGGU	ACCCUUUGGGUAACAUCCCC	Translation	1

trf_1_30	Bra033370	3	21.6	1	20	4750	4769	GGGGAUGUAGCUCAAAUGGU	ACCCUUUGGGUAACAUCCCC	Translation	1
trf_1_72	Bra033370	3	21.6	1	20	4750	4769	GGGGAUGUAGCUCAAAUGGU	ACCCUUUGGGUAACAUCCCC	Translation	1
trf_1_73	Bra033370	3	21.6	1	20	4750	4769	GGGGAUGUAGCUCAAAUGGU	ACCCUUUGGGUAACAUCCCC	Translation	1
trf_1_116	Bra033370	3	21.6	1	20	4750	4769	GGGGAUGUAGCUCAAAUGGU	ACCCUUUGGGUAACAUCCCC	Translation	1
trf_1_117	Bra033370	3	21.6	1	20	4750	4769	GGGGAUGUAGCUCAAAUGGU	ACCCUUUGGGUAACAUCCCC	Translation	1
trf_1_118	Bra033370	3	21.6	1	24	4746	4769	GGGGAUGUAGCUCAAAUGGUAGAG	CUGGACCCUUUGGGUAACAUCCCC	Translation	1
trf_1_121	Bra033370	3	21.6	1	20	4750	4769	GGGGAUGUAGCUCAAAUGGU	ACCCUUUGGGUAACAUCCCC	Translation	1
trf_1_122	Bra033370	3	21.6	1	20	4750	4769	GGGGAUGUAGCUCAAAUGGU	ACCCUUUGGGUAACAUCCCC	Translation	1
trf_1_129	Bra033370	3	21.6	1	20	4750	4769	GGGGAUGUAGCUCAAAUGGU	ACCCUUUGGGUAACAUCCCC	Translation	1
trf_1_130	Bra033370	3	21.6	1	20	4750	4769	GGGGAUGUAGCUCAAAUGGU	ACCCUUUGGGUAACAUCCCC	Translation	1
trf_1_336	Bra033370	3	21.6	1	20	4750	4769	GGGGAUGUAGCUCAAAUGGU	ACCCUUUGGGUAACAUCCCC	Translation	1
trf_1_337	Bra033370	3	21.6	1	20	4750	4769	GGGGAUGUAGCUCAAAUGGU	ACCCUUUGGGUAACAUCCCC	Translation	1
trf_1_367	Bra033370	3	21.6	1	20	4750	4769	GGGGAUGUAGCUCAAAUGGU	ACCCUUUGGGUAACAUCCCC	Translation	1
trf_1_368	Bra033370	3	21.6	1	24	4746	4769	GGGGAUGUAGCUCAAAUGGUAGAG	CUGGACCCUUUGGGUAACAUCCCC	Translation	1
trf_1_517	Bra033370	3	21.6	1	20	4750	4769	GGGGAUGUAGCUCAAAUGGU	ACCCUUUGGGUAACAUCCCC	Translation	1
trf_1_518	Bra033370	3	21.6	1	24	4746	4769	GGGGAUGUAGCUCAAAUGGUAGAG	CUGGACCCUUUGGGUAACAUCCCC	Translation	1
trf_1_519	Bra033370	3	21.6	1	20	4750	4769	GGGGAUGUAGCUCAAAUGGU	ACCCUUUGGGUAACAUCCCC	Translation	1

trf_1_566	Bra033370	3	21.6	1	20	4750	4769	GGGGAUGUAGCUCAAAUGGU	ACCCUUUGGGUAACAUCCCC	Translation	1
trf_1_567	Bra033370	3	21.6	1	24	4746	4769	GGGGAUGUAGCUCAAAUGGUAGAG	CUGGACCCUUUGGGUAACAUCCCC	Translation	1
trf_1_568	Bra033370	3	21.6	1	20	4750	4769	GGGGAUGUAGCUCAAAUGGU	ACCCUUUGGGUAACAUCCCC	Translation	1
trf_1_182	Bra033405	3	24.4	1	23	30	52	CCGACCUUAGCUCAGUUGGUAGA	UCAACCAACGGAGUUGGGGUUGG	Cleavage	1
trf_1_476	Bra033405	3	24.4	1	23	30	52	CCGACCUUAGCUCAGUUGGUAGA	UCAACCAACGGAGUUGGGGUUGG	Cleavage	1
trf_1_478	Bra033405	3	24.4	1	23	30	52	CCGACCUUAGCUCAGUUGGUAGA	UCAACCAACGGAGUUGGGGUUGG	Cleavage	1
trf_1_479	Bra033405	3	24.4	1	23	30	52	CCGACCUUAGCUCAGUUGGUAGA	UCAACCAACGGAGUUGGGGUUGG	Cleavage	1
trf_1_520	Bra033405	3	24.4	1	23	30	52	CCGACCUUAGCUCAGUUGGUAGA	UCAACCAACGGAGUUGGGGUUGG	Cleavage	1
trf_1_182	Bra033406	3	24.4	1	23	30	52	CCGACCUUAGCUCAGUUGGUAGA	UCAACCAACGGAGUUGGGGUUGG	Cleavage	1
trf_1_476	Bra033406	3	24.4	1	23	30	52	CCGACCUUAGCUCAGUUGGUAGA	UCAACCAACGGAGUUGGGGUUGG	Cleavage	1
trf_1_478	Bra033406	3	24.4	1	23	30	52	CCGACCUUAGCUCAGUUGGUAGA	UCAACCAACGGAGUUGGGGUUGG	Cleavage	1
trf_1_479	Bra033406	3	24.4	1	23	30	52	CCGACCUUAGCUCAGUUGGUAGA	UCAACCAACGGAGUUGGGGUUGG	Cleavage	1
trf_1_520	Bra033406	3	24.4	1	23	30	52	CCGACCUUAGCUCAGUUGGUAGA	UCAACCAACGGAGUUGGGGUUGG	Cleavage	1
trf_1_413	Bra033536	3	10.5	1	20	426	445	GGGAUUGUAGUUCAAUCGGU	ACCGAUUGAUCAGCAAUUCC	Translation	1
trf_1_436	Bra033536	3	10.5	1	20	426	445	GGGAUUGUAGUUCAAUCGGU	ACCGAUUGAUCAGCAAUUCC	Translation	1
trf_1_454	Bra033536	3	10.5	1	20	426	445	GGGAUUGUAGUUCAAUCGGU	ACCGAUUGAUCAGCAAUUCC	Translation	1
trf_1_459	Bra033536	3	10.5	1	20	426	445	GGGAUUGUAGUUCAAUCGGU	ACCGAUUGAUCAGCAAUUCC	Translation	1

trf_1_577	Bra034278	3	21.9	1	21	2430	2450	UUAGGUUCAAAUCCUAUUGGA	UGCGAUAGGAUUUGGACCUGG	Cleavage	1
trf_1_578	Bra034278	3	22.4	1	20	2429	2448	AGGUUCAAAUCCUAUUGGAC	CUGCGAUAGGAUUUGGACCU	Cleavage	1
trf_1_579	Bra034278	2.5	22.3	1	20	2430	2449	UAGGUUCAAAUCCUAUUGGA	UGCGAUAGGAUUUGGACCUG	Cleavage	1
trf_1_433	Bra034571	3	9.0	1	20	52	71	GGGAUUGUAGUUCAAUUGGG	CCUAAUUCAACCACAAUCUC	Translation	1
trf_1_441	Bra034571	3	9.0	1	20	52	71	GGGAUUGUAGUUCAAUUGGG	CCUAAUUCAACCACAAUCUC	Translation	1
trf_1_442	Bra034571	3	9.0	1	20	52	71	GGGAUUGUAGUUCAAUUGGG	CCUAAUUCAACCACAAUCUC	Translation	1
trf_1_418	Bra034753	3	11.6	1	20	443	462	GGGAUUGUAGUUCAAUUGGA	UCCAAAUGAGCAGCAAUCCC	Translation	1
trf_1_419	Bra034753	3	11.6	1	20	443	462	GGGAUUGUAGUUCAAUUGGA	UCCAAAUGAGCAGCAAUCCC	Translation	1
trf_1_434	Bra034753	3	11.6	1	20	443	462	GGGAUUGUAGUUCAAUUGGA	UCCAAAUGAGCAGCAAUCCC	Translation	1
trf_1_445	Bra034753	3	11.6	1	22	441	462	GGGAUUGUAGUUCAAUUGGAAA	UAUCCAAAUGAGCAGCAAUCCC	Translation	1
trf_1_447	Bra034753	3	11.6	1	21	442	462	GGGAUUGUAGUUCAAUUGGAU	AUCCAAAUGAGCAGCAAUCCC	Translation	1
trf_1_456	Bra034753	3	11.6	1	22	441	462	GGGAUUGUAGUUCAAUUGGAGA	UAUCCAAAUGAGCAGCAAUCCC	Translation	1
trf_1_465	Bra034753	3	11.6	1	20	443	462	GGGAUUGUAGUUCAAUUGGA	UCCAAAUGAGCAGCAAUCCC	Translation	1
trf_1_1	Bra035409	3	10.4	1	20	1328	1347	GUCGUUGUAGUAUAGUGGUA	UACCACAAUACUUCAACGAG	Cleavage	1
trf_1_24	Bra035409	3	10.4	1	20	1328	1347	GUCGUUGUAGUAUAGUGGUA	UACCACAAUACUUCAACGAG	Cleavage	1
trf_1_25	Bra035409	3	10.4	1	20	1328	1347	GUCGUUGUAGUAUAGUGGUA	UACCACAAUACUUCAACGAG	Cleavage	1
trf_1_26	Bra035409	3	10.4	1	20	1328	1347	GUCGUUGUAGUAUAGUGGUA	UACCACAAUACUUCAACGAG	Cleavage	1

trf_1_50	Bra035409	3	10.4	1	20	1328	1347	GUCGUUGUAGUAUAGUGGUA	UACCACAAUACUUCAACGAG	Cleavage	1
trf_1_52	Bra035409	3	10.4	1	20	1328	1347	GUCGUUGUAGUAUAGUGGUA	UACCACAAUACUUCAACGAG	Cleavage	1
trf_1_53	Bra035409	3	10.4	1	20	1328	1347	GUCGUUGUAGUAUAGUGGUA	UACCACAAUACUUCAACGAG	Cleavage	1
trf_1_65	Bra035409	3	10.4	1	20	1328	1347	GUCGUUGUAGUAUAGUGGUA	UACCACAAUACUUCAACGAG	Cleavage	1
trf_1_66	Bra035409	3	10.4	1	20	1328	1347	GUCGUUGUAGUAUAGUGGUA	UACCACAAUACUUCAACGAG	Cleavage	1
trf_1_67	Bra035409	3	10.4	1	20	1328	1347	GUCGUUGUAGUAUAGUGGUA	UACCACAAUACUUCAACGAG	Cleavage	1
trf_1_125	Bra035409	3	10.4	1	20	1328	1347	GUCGUUGUAGUAUAGUGGUA	UACCACAAUACUUCAACGAG	Cleavage	1
trf_1_131	Bra035409	3	10.4	1	20	1328	1347	GUCGUUGUAGUAUAGUGGUA	UACCACAAUACUUCAACGAG	Cleavage	1
trf_1_132	Bra035409	3	10.4	1	20	1328	1347	GUCGUUGUAGUAUAGUGGUA	UACCACAAUACUUCAACGAG	Cleavage	1
trf_1_230	Bra035409	3	10.4	1	20	1328	1347	GUCGUUGUAGUAUAGUGGUA	UACCACAAUACUUCAACGAG	Cleavage	1
trf_1_257	Bra035409	3	10.4	1	20	1328	1347	GUCGUUGUAGUAUAGUGGUA	UACCACAAUACUUCAACGAG	Cleavage	1
trf_1_281	Bra035409	3	10.4	1	20	1328	1347	GUCGUUGUAGUAUAGUGGUA	UACCACAAUACUUCAACGAG	Cleavage	1
trf_1_282	Bra035409	3	10.4	1	20	1328	1347	GUCGUUGUAGUAUAGUGGUA	UACCACAAUACUUCAACGAG	Cleavage	1
trf_1_306	Bra035409	3	10.4	1	20	1328	1347	GUCGUUGUAGUAUAGUGGUA	UACCACAAUACUUCAACGAG	Cleavage	1
trf_1_331	Bra035409	3	10.4	1	20	1328	1347	GUCGUUGUAGUAUAGUGGUA	UACCACAAUACUUCAACGAG	Cleavage	1
trf_1_372	Bra035409	3	10.4	1	20	1328	1347	GUCGUUGUAGUAUAGUGGUA	UACCACAAUACUUCAACGAG	Cleavage	1
trf_1_373	Bra035409	3	10.4	1	20	1328	1347	GUCGUUGUAGUAUAGUGGUA	UACCACAAUACUUCAACGAG	Cleavage	1

trf_1_374	Bra035409	3	10.4	1	20	1328	1347	GUCGUUGUAGUAUAGUGGUA	UACCACAAUACUUCAACGAG	Cleavage	1
trf_1_390	Bra035409	3	10.4	1	20	1328	1347	GUCGUUGUAGUAUAGUGGUA	UACCACAAUACUUCAACGAG	Cleavage	1
trf_1_399	Bra035409	3	10.4	1	20	1328	1347	GUCGUUGUAGUAUAGUGGUA	UACCACAAUACUUCAACGAG	Cleavage	1
trf_1_524	Bra035409	3	10.4	1	20	1328	1347	GUCGUUGUAGUAUAGUGGUA	UACCACAAUACUUCAACGAG	Cleavage	1
trf_1_532	Bra035409	3	10.4	1	20	1328	1347	GUCGUUGUAGUAUAGUGGUA	UACCACAAUACUUCAACGAG	Cleavage	1
trf_1_576	Bra035409	3	10.4	1	20	1328	1347	GUCGUUGUAGUAUAGUGGUA	UACCACAAUACUUCAACGAG	Cleavage	1
trf_1_578	Bra036447	2.5	17.4	1	22	804	825	AGGUUCAAAUCCUAUUGGACGC	GAGUCCAAUGUGGUUUGAGCCU	Cleavage	1
trf_1_31	Bra036771	3	20.2	1	23	5375	5397	GGGGAUGUAGCUCAGAUGGUAGA	UCGACUGGCUGAGCUACAUUCCU	Cleavage	1
trf_1_35	Bra036771	3	20.2	1	20	5378	5397	GGGGAUGUAGCUCAGAUGGU	ACUGGCUGAGCUACAUUCCU	Cleavage	1
trf_1_60	Bra036771	3	20.2	1	23	5375	5397	GGGGAUGUAGCUCAGAUGGUAGA	UCGACUGGCUGAGCUACAUUCCU	Cleavage	1
trf_1_62	Bra036771	3	20.2	1	24	5374	5397	GGGGAUGUAGCUCAGAUGGUAGAG	CUCGACUGGCUGAGCUACAUUCCU	Cleavage	1
trf_1_90	Bra036771	3	20.2	1	23	5375	5397	GGGGAUGUAGCUCAGAUGGUAGA	UCGACUGGCUGAGCUACAUUCCU	Cleavage	1
trf_1_133	Bra036771	3	20.2	1	23	5375	5397	GGGGAUGUAGCUCAGAUGGUAGA	UCGACUGGCUGAGCUACAUUCCU	Cleavage	1
trf_1_137	Bra036771	3	20.2	1	20	5378	5397	GGGGAUGUAGCUCAGAUGGU	ACUGGCUGAGCUACAUUCCU	Cleavage	1
trf_1_138	Bra036771	3	20.2	1	20	5378	5397	GGGGAUGUAGCUCAGAUGGU	ACUGGCUGAGCUACAUUCCU	Cleavage	1
trf_1_153	Bra036771	3	20.2	1	23	5375	5397	GGGGAUGUAGCUCAGAUGGUAGA	UCGACUGGCUGAGCUACAUUCCU	Cleavage	1
trf_1_154	Bra036771	3	20.2	1	24	5374	5397	GGGGAUGUAGCUCAGAUGGUAGAG	CUCGACUGGCUGAGCUACAUUCCU	Cleavage	1

trf_1_160	Bra036771	3	20.2	1	23	5375	5397	GGGGAUGUAGCUCAGAUGGUAGA	UCGACUGGCUGAGCUACAUUCCU	Cleavage	1
trf_1_162	Bra036771	3	20.2	1	24	5374	5397	GGGGAUGUAGCUCAGAUGGUAGAG	CUCGACUGGCUGAGCUACAUUCCU	Cleavage	1
trf_1_169	Bra036771	3	20.2	1	23	5375	5397	GGGGAUGUAGCUCAGAUGGUAGA	UCGACUGGCUGAGCUACAUUCCU	Cleavage	1
trf_1_173	Bra036771	3	20.2	1	24	5374	5397	GGGGAUGUAGCUCAGAUGGUAGAG	CUCGACUGGCUGAGCUACAUUCCU	Cleavage	1
trf_1_183	Bra036771	3	20.2	1	23	5375	5397	GGGGAUGUAGCUCAGAUGGUAGA	UCGACUGGCUGAGCUACAUUCCU	Cleavage	1
trf_1_233	Bra036771	3	20.2	1	23	5375	5397	GGGGAUGUAGCUCAGAUGGUAGA	UCGACUGGCUGAGCUACAUUCCU	Cleavage	1
trf_1_236	Bra036771	3	20.2	1	24	5374	5397	GGGGAUGUAGCUCAGAUGGUAGAG	CUCGACUGGCUGAGCUACAUUCCU	Cleavage	1
trf_1_244	Bra036771	3	20.2	1	23	5375	5397	GGGGAUGUAGCUCAGAUGGUAGA	UCGACUGGCUGAGCUACAUUCCU	Cleavage	1
trf_1_247	Bra036771	3	20.2	1	24	5374	5397	GGGGAUGUAGCUCAGAUGGUAGAG	CUCGACUGGCUGAGCUACAUUCCU	Cleavage	1
trf_1_266	Bra036771	3	20.2	1	23	5375	5397	GGGGAUGUAGCUCAGAUGGUAGA	UCGACUGGCUGAGCUACAUUCCU	Cleavage	1
trf_1_286	Bra036771	3	20.2	1	23	5375	5397	GGGGAUGUAGCUCAGAUGGUAGA	UCGACUGGCUGAGCUACAUUCCU	Cleavage	1
trf_1_293	Bra036771	3	20.2	1	23	5375	5397	GGGGAUGUAGCUCAGAUGGUAGA	UCGACUGGCUGAGCUACAUUCCU	Cleavage	1
trf_1_297	Bra036771	3	20.2	1	24	5374	5397	GGGGAUGUAGCUCAGAUGGUAGAG	CUCGACUGGCUGAGCUACAUUCCU	Cleavage	1
trf_1_302	Bra036771	3	20.2	1	23	5375	5397	GGGGAUGUAGCUCAGAUGGUAGA	UCGACUGGCUGAGCUACAUUCCU	Cleavage	1
trf_1_307	Bra036771	3	20.2	1	23	5375	5397	GGGGAUGUAGCUCAGAUGGUAGA	UCGACUGGCUGAGCUACAUUCCU	Cleavage	1
trf_1_314	Bra036771	3	20.2	1	23	5375	5397	GGGGAUGUAGCUCAGAUGGUAGA	UCGACUGGCUGAGCUACAUUCCU	Cleavage	1
trf_1_322	Bra036771	3	20.2	1	23	5375	5397	GGGGAUGUAGCUCAGAUGGUAGA	UCGACUGGCUGAGCUACAUUCCU	Cleavage	1

trf_1_323	Bra036771	3	20.2	1	24	5374	5397	GGGGAUGUAGCUCAGAUGGUAGAG	CUCGACUGGCUGAGCUACAUUCCU	Cleavage	1
trf_1_332	Bra036771	3	20.2	1	23	5375	5397	GGGGAUGUAGCUCAGAUGGUAGA	UCGACUGGCUGAGCUACAUUCCU	Cleavage	1
trf_1_338	Bra036771	3	20.2	1	24	5374	5397	GGGGAUGUAGCUCAGAUGGUAGAG	CUCGACUGGCUGAGCUACAUUCCU	Cleavage	1
trf_1_341	Bra036771	3	20.2	1	23	5375	5397	GGGGAUGUAGCUCAGAUGGUAGA	UCGACUGGCUGAGCUACAUUCCU	Cleavage	1
trf_1_351	Bra036771	3	20.2	1	23	5375	5397	GGGGAUGUAGCUCAGAUGGUAGA	UCGACUGGCUGAGCUACAUUCCU	Cleavage	1
trf_1_353	Bra036771	3	20.2	1	20	5378	5397	GGGGAUGUAGCUCAGAUGGU	ACUGGCUGAGCUACAUUCCU	Cleavage	1
trf_1_362	Bra036771	3	20.2	1	23	5375	5397	GGGGAUGUAGCUCAGAUGGUAGA	UCGACUGGCUGAGCUACAUUCCU	Cleavage	1
trf_1_366	Bra036771	3	20.2	1	24	5374	5397	GGGGAUGUAGCUCAGAUGGUAGAG	CUCGACUGGCUGAGCUACAUUCCU	Cleavage	1
trf_1_375	Bra036771	3	20.2	1	23	5375	5397	GGGGAUGUAGCUCAGAUGGUAGA	UCGACUGGCUGAGCUACAUUCCU	Cleavage	1
trf_1_379	Bra036771	3	20.2	1	24	5374	5397	GGGGAUGUAGCUCAGAUGGUAGAG	CUCGACUGGCUGAGCUACAUUCCU	Cleavage	1
trf_1_382	Bra036771	3	20.2	1	23	5375	5397	GGGGAUGUAGCUCAGAUGGUAGA	UCGACUGGCUGAGCUACAUUCCU	Cleavage	1
trf_1_383	Bra036771	3	20.2	1	24	5374	5397	GGGGAUGUAGCUCAGAUGGUAGAG	CUCGACUGGCUGAGCUACAUUCCU	Cleavage	1
trf_1_391	Bra036771	3	20.2	1	23	5375	5397	GGGGAUGUAGCUCAGAUGGUAGA	UCGACUGGCUGAGCUACAUUCCU	Cleavage	1
trf_1_392	Bra036771	3	20.2	1	20	5378	5397	GGGGAUGUAGCUCAGAUGGU	ACUGGCUGAGCUACAUUCCU	Cleavage	1
trf_1_393	Bra036771	3	20.2	1	24	5374	5397	GGGGAUGUAGCUCAGAUGGUAGAG	CUCGACUGGCUGAGCUACAUUCCU	Cleavage	1
trf_1_400	Bra036771	3	20.2	1	23	5375	5397	GGGGAUGUAGCUCAGAUGGUAGA	UCGACUGGCUGAGCUACAUUCCU	Cleavage	1
trf_1_404	Bra036771	3	20.2	1	20	5378	5397	GGGGAUGUAGCUCAGAUGGU	ACUGGCUGAGCUACAUUCCU	Cleavage	1

trf_1_408	Bra036771	3	20.2	1	23	5375	5397	GGGGAUGUAGCUCAGAUGGUAGA	UCGACUGGCUGAGCUACAUUCCU	Cleavage	1
trf_1_410	Bra036771	3	20.2	1	20	5378	5397	GGGGAUGUAGCUCAGAUGGU	ACUGGCUGAGCUACAUUCCU	Cleavage	1
trf_1_482	Bra036771	3	20.2	1	23	5375	5397	GGGGAUGUAGCUCAGAUGGUAGA	UCGACUGGCUGAGCUACAUUCCU	Cleavage	1
trf_1_486	Bra036771	3	20.2	1	20	5378	5397	GGGGAUGUAGCUCAGAUGGU	ACUGGCUGAGCUACAUUCCU	Cleavage	1
trf_1_489	Bra036771	3	20.2	1	23	5375	5397	GGGGAUGUAGCUCAGAUGGUAGA	UCGACUGGCUGAGCUACAUUCCU	Cleavage	1
trf_1_494	Bra036771	3	20.2	1	20	5378	5397	GGGGAUGUAGCUCAGAUGGU	ACUGGCUGAGCUACAUUCCU	Cleavage	1
trf_1_498	Bra036771	3	20.2	1	20	5378	5397	GGGGAUGUAGCUCAGAUGGU	ACUGGCUGAGCUACAUUCCU	Cleavage	1
trf_1_499	Bra036771	3	20.2	1	23	5375	5397	GGGGAUGUAGCUCAGAUGGUAGA	UCGACUGGCUGAGCUACAUUCCU	Cleavage	1
trf_1_126	Bra037152	3	12.2	1	22	478	499	GACGGUUUGGCCGAGUGGUCUA	UUGACUACUCGUCCAAACCCUC	Translation	1
trf_1_11	Bra037499	3	14.7	1	22	174	195	UCCGUUGUAGUCUAGCUGGUUA	UGACUGGUUAGGCCACAACGGA	Translation	1
trf_1_12	Bra037499	3	14.7	1	22	174	195	UCCGUUGUAGUCUAGCUGGUUA	UGACUGGUUAGGCCACAACGGA	Translation	1
trf_1_42	Bra037499	3	14.7	1	22	174	195	UCCGUUGUAGUCUAGCUGGUCA	UGACUGGUUAGGCCACAACGGA	Translation	1
trf_1_88	Bra037499	3	14.7	1	22	174	195	UCCGUUGUAGUCUAGCUGGUCA	UGACUGGUUAGGCCACAACGGA	Translation	1
trf_1_128	Bra037499	3	14.7	1	22	174	195	UCCGUUGUAGUCUAGCUGGUCA	UGACUGGUUAGGCCACAACGGA	Translation	1
trf_1_140	Bra037499	3	14.7	1	22	174	195	UCCGUUGUAGUCUAGCUGGUCA	UGACUGGUUAGGCCACAACGGA	Translation	1
trf_1_159	Bra037499	3	14.7	1	22	174	195	UCCGUUGUAGUCUAGCUGGUCA	UGACUGGUUAGGCCACAACGGA	Translation	1
trf_1_263	Bra037499	3	14.7	1	22	174	195	UCCGUUGUAGUCUAGCUGGUCA	UGACUGGUUAGGCCACAACGGA	Translation	1

trf_1_326	Bra037499	3	14.7	1	22	174	195	UCCGUUGUAGUCUAGCUGGUCA	UGACUGGUUAGGCCACAACGGA	Translation	1
trf_1_570	Bra037499	3	14.7	1	22	174	195	UCCGUUGUAGUCUAGCUGGUCA	UGACUGGUUAGGCCACAACGGA	Translation	1
trf_1_8	Bra037748	2.5	22.8	1	25	561	585	UCCGUUAUCGUCCAGCGGUUAGGAU	GUUGAAACCGCUGUCUGAUAACGGA	Translation	1
trf_1_9	Bra037748	2.5	22.8	1	25	561	585	UCCGUUAUCGUCCAGCGGUUAGGAU	GUUGAAACCGCUGUCUGAUAACGGA	Translation	1
trf_1_10	Bra037748	2.5	22.8	1	25	561	585	UCCGUUAUCGUCCAGCGGUUAGGAU	GUUGAAACCGCUGUCUGAUAACGGA	Translation	1
trf_1_94	Bra037748	3	22.8	1	25	561	585	UCCGUUGUCGUCCAGCGGUUAGGAU	GUUGAAACCGCUGUCUGAUAACGGA	Translation	1
trf_1_95	Bra037748	3	22.8	1	25	561	585	UCCGUUGUCGUCCAGCGGUUAGGAU	GUUGAAACCGCUGUCUGAUAACGGA	Translation	1
trf_1_231	Bra037748	2.5	22.8	1	25	561	585	UCCGUUAUCGUCCAGCGGUUAGGAU	GUUGAAACCGCUGUCUGAUAACGGA	Translation	1
trf_1_232	Bra037748	2.5	22.8	1	25	561	585	UCCGUUAUCGUCCAGCGGUUAGGAU	GUUGAAACCGCUGUCUGAUAACGGA	Translation	1
trf_1_259	Bra037748	3	22.8	1	20	566	585	UCCGUUGUCGUCCAGCGGUU	AACCGCUGUCUGAUAACGGA	Translation	1
trf_1_261	Bra037748	3	22.8	1	25	561	585	UCCGUUGUCGUCCAGCGGUUAGGAU	GUUGAAACCGCUGUCUGAUAACGGA	Translation	1
trf_1_287	Bra037748	3	22.8	1	25	561	585	UCCGUUGUCGUCCAGCGGUUAGGAU	GUUGAAACCGCUGUCUGAUAACGGA	Translation	1
trf_1_288	Bra037748	3	22.8	1	25	561	585	UCCGUUGUCGUCCAGCGGUUAGGAU	GUUGAAACCGCUGUCUGAUAACGGA	Translation	1
trf_1_510	Bra037748	3	22.8	1	25	561	585	UCCGUUGUCGUCCAGCGGUUAGGAU	GUUGAAACCGCUGUCUGAUAACGGA	Translation	1
trf_1_511	Bra037748	3	22.8	1	25	561	585	UCCGUUGUCGUCCAGCGGUUAGGAU	GUUGAAACCGCUGUCUGAUAACGGA	Translation	1
trf_1_512	Bra037748	3	22.8	1	20	566	585	UCCGUUGUCGUCCAGCGGUU	AACCGCUGUCUGAUAACGGA	Translation	1
trf_1_27	Bra038442	3	9.2	1	21	202	222	GUCGAUAUGUCCGAGUGGUUA	UAUCUAUUCGGACGUAUCGAU	Cleavage	1

trf_1_345	Bra038442	3	9.2	1	21	202	222	GUCGAUAUGUCCGAGUGGUUA	UAUCUAUUCGGACGUAUCGAU	Cleavage	1
trf_1_54	Bra040072	3	13.8	1	21	504	524	GUCUGGGUGGUGUAGUCGGUU	AGCUGACUCCAUCACUCAGAC	Cleavage	1
trf_1_188	Bra040072	3	13.8	1	21	504	524	GUCUGGGUGGUGUAGUCGGUU	AGCUGACUCCAUCACUCAGAC	Cleavage	1
trf_1_528	Bra040072	3	13.8	1	21	504	524	GUCUGGGUGGUGUAGUCGGUU	AGCUGACUCCAUCACUCAGAC	Cleavage	1
trf_1_217	Bra040276	3	16.3	1	21	987	1007	GGGUCGAUGCCCGAGCGGAAA	UUUCCGCUCGGGCCUUGAUCG	Cleavage	1
trf_1_221	Bra040276	3	16.3	1	20	988	1007	GGGUCGAUGCCCGAGCGGAA	UUCCGCUCGGGCCUUGAUCG	Cleavage	1
trf_1_76	Bra040563	3	21.7	1	20	1048	1067	GCGCCUGUAGCUCAGUGGAA	UUCCACUCAGCUACAGUUGC	Cleavage	1

tRF - Apical Meristem

ncRNA_Acc.	Target_Acc.	Expectation	UPE	ncRNA_start	ncRNA_end	Target_start	Target_end	ncRNA_aligned_fragment	Target_aligned_fragment	Inhibition	Multiplicity
trf_2_1	Bra024638	3	7.5	1	24	202	225	GGGGAUGUAGCUCAAAUGGUAGAG	UUCCAACAUUUCGGUUACAUCCCC	Cleavage	1
trf_2_1	Bra025904	3	17.2	1	20	344	363	GGGGAUGUAGCUCAAAUGGU	ACAAUUUGAGCUUCAUCUUC	Cleavage	1
trf_2_2	Bra024638	3	7.5	1	23	203	225	GGGGAUGUAGCUCAAAUGGUAGA	UCCAACAUUUCGGUUACAUCCCC	Cleavage	1
trf_2_2	Bra025904	3	17.2	1	20	344	363	GGGGAUGUAGCUCAAAUGGU	ACAAUUUGAGCUUCAUCUUC	Cleavage	1
trf_2_3	Bra000529	2.5	11.2	1	20	985	1004	UCCGUUAUCGUCCAGCGGUU	AACCGGAGGACGAUAAUGGA	Cleavage	1
trf_2_3	Bra032111	3	22.4	1	20	727	746	UCCGUUAUCGUCCAGCGGUU	GACGGCUUGACGAUGACGGA	Cleavage	1
trf_2_4	Bra000529	2.5	11.2	1	21	984	1004	UCCGUUAUCGUCCAGCGGUUA	UAACCGGAGGACGAUAAUGGA	Cleavage	1
trf_2_4	Bra032111	3	22.4	1	20	727	746	UCCGUUAUCGUCCAGCGGUU	GACGGCUUGACGAUGACGGA	Cleavage	1
trf_2_5	Bra000529	2.5	11.2	1	21	984	1004	UCCGUUAUCGUCCAGCGGUUA	UAACCGGAGGACGAUAAUGGA	Cleavage	1
trf_2_5	Bra032111	3	22.4	1	20	727	746	UCCGUUAUCGUCCAGCGGUU	GACGGCUUGACGAUGACGGA	Cleavage	1
trf_2_7	Bra000529	2.5	11.2	1	21	984	1004	UCCGUUAUCGUCCAGCGGUUA	UAACCGGAGGACGAUAAUGGA	Cleavage	1
trf_2_7	Bra032111	3	22.4	1	20	727	746	UCCGUUAUCGUCCAGCGGUU	GACGGCUUGACGAUGACGGA	Cleavage	1
trf_2_11	Bra000529	2.5	11.2	1	21	984	1004	UCCGUUAUCGUCCAGCGGUUA	UAACCGGAGGACGAUAAUGGA	Cleavage	1
trf_2_11	Bra032111	3	22.4	1	20	727	746	UCCGUUAUCGUCCAGCGGUU	GACGGCUUGACGAUGACGGA	Cleavage	1
trf_2_12	Bra000529	2.5	11.2	1	21	984	1004	UCCGUUAUCGUCCAGCGGUUA	UAACCGGAGGACGAUAAUGGA	Cleavage	1

trf_2_12	Bra032111	3	22.4	1	20	727	746	UCCGUUAUCGUCCAGCGGUU	GACGGCUUGACGAUGACGGA	Cleavage	1
trf_2_13	Bra000529	2.5	11.2	1	21	984	1004	UCCGUUAUCGUCCAGCGGUUA	UAACCGGAGGACGAUAAUGGA	Cleavage	1
trf_2_13	Bra032111	3	22.4	1	20	727	746	UCCGUUAUCGUCCAGCGGUU	GACGGCUUGACGAUGACGGA	Cleavage	1
trf_2_14	Bra013528	3	22.0	1	24	196	219	UCCGUUGUAGUCUAGCUGGUUAGG	CAUCAUCAGCUUGGCUAUAACGGG	Cleavage	1
trf_2_15	Bra013528	3	22.0	1	24	196	219	UCCGUUGUAGUCUAGCUGGUUAGG	CAUCAUCAGCUUGGCUAUAACGGG	Cleavage	1
trf_2_16	Bra024638	3	7.5	1	23	203	225	GGGGAUGUAGCUCAAAUGGUAGA	UCCAACAUUUCGGUUACAUCCCC	Cleavage	1
trf_2_16	Bra025904	3	17.2	1	20	344	363	GGGGAUGUAGCUCAAAUGGU	ACAAUUUGAGCUUCAUCUUC	Cleavage	1
trf_2_17	Bra016601	3	20.8	1	24	415	438	UCCGUCGUAGUCUAGCUGGUUAGG	CUGAAGAAGCUAGGCUGCGACGGA	Cleavage	1
trf_2_18	Bra016601	3	20.8	1	21	418	438	UCCGUCGUAGUCUAGCUGGUU	AAGAAGCUAGGCUGCGACGGA	Cleavage	1
trf_2_19	Bra016601	3	20.8	1	24	415	438	UCCGUCGUAGUCUAGCUGGUUAGG	CUGAAGAAGCUAGGCUGCGACGGA	Cleavage	1
trf_2_21	Bra016601	3	20.8	1	21	418	438	UCCGUCGUAGUCUAGCUGGUU	AAGAAGCUAGGCUGCGACGGA	Cleavage	1
trf_2_22	Bra016601	3	20.8	1	24	415	438	UCCGUCGUAGUCUAGCUGGUUAGG	CUGAAGAAGCUAGGCUGCGACGGA	Cleavage	1
trf_2_26	Bra020755	3	12.6	1	20	454	473	GGGGAUGUAGCUCAAAUGGU	GCCAUUGGAGCUAUAUUCUC	Cleavage	1
trf_2_26	Bra025904	3	17.2	1	20	344	363	GGGGAUGUAGCUCAAAUGGU	ACAAUUUGAGCUUCAUCUUC	Cleavage	1
trf_2_26	Bra024638	3	7.5	1	20	206	225	GGGGAUGUAGCUCAAAUGGU	AACAUUUCGGUUACAUCCCC	Cleavage	1
trf_2_27	Bra009876	3	14.9	1	22	16	37	GGGGAUGUAGCUCAGAUGGUAG	UUAUCAUCUGGGCUUCGUCCUC	Cleavage	1
trf_2_27	Bra016141	3	10.4	1	22	3031	3052	GGGGAUGUAGCUCAGAUGGUAG	UUAUCAUCUGAGCUUCGUUCUC	Cleavage	1

trf_2_27	Bra036771	3	20.2	1	23	5375	5397	GGGGAUGUAGCUCAGAUGGUAGA	UCGACUGGCUGAGCUACAUUCCU	Cleavage	1
trf_2_27	Bra018620	3	8.3	1	20	7	26	GGGGAUGUAGCUCAGAUGGU	GCCACCUCAGCUACAUCCUC	Cleavage	1
trf_2_37	Bra013528	3	22.0	1	24	196	219	UCCGUUGUAGUCUAGCUGGUCAGG	CAUCAUCAGCUUGGCUAUAACGGG	Cleavage	1
trf_2_38	Bra005511	2.5	12.8	1	22	1113	1134	GUGGCUGUAGUUUAGUGGUGAG	CUCACCGCUGAACUAUGGCUAC	Cleavage	1
trf_2_38	Bra035454	3	17.9	1	24	1241	1264	GUGGCUGUAGUUUAGUGGUGAGAA	UAUUCAUCUCUAGGCUACAGCCAU	Cleavage	1
trf_2_38	Bra009047	3	12.1	1	20	243	262	GUGGCUGUAGUUUAGUGGUG	CAACAUUGAGCUACAGCCAU	Cleavage	1
trf_2_49	Bra007154	2.5	14.1	1	21	510	530	GCACCAGUGGUCUAGUGGUAG	UUACUACUGGACCUUUGGUGC	Cleavage	1
trf_2_55	Bra009876	3	14.9	1	22	16	37	GGGGAUGUAGCUCAGAUGGUAG	UUAUCAUCUGGGCUUCGUCCUC	Cleavage	1
trf_2_55	Bra016141	3	10.4	1	22	3031	3052	GGGGAUGUAGCUCAGAUGGUAG	UUAUCAUCUGAGCUUCGUUCUC	Cleavage	1
trf_2_55	Bra036771	3	20.2	1	23	5375	5397	GGGGAUGUAGCUCAGAUGGUAGA	UCGACUGGCUGAGCUACAUUCCU	Cleavage	1
trf_2_55	Bra018620	3	8.3	1	20	7	26	GGGGAUGUAGCUCAGAUGGU	GCCACCUCAGCUACAUCCUC	Cleavage	1
trf_2_56	Bra007154	2.5	14.1	1	21	510	530	GCACCAGUGGUCUAGUGGUAG	UUACUACUGGACCUUUGGUGC	Cleavage	1
trf_2_57	Bra007154	2.5	14.1	1	21	510	530	GCACCAGUGGUCUAGUGGUAG	UUACUACUGGACCUUUGGUGC	Cleavage	1
trf_2_59	Bra020835	3	9.5	1	22	5316	5337	GGGGGUGUAGCUCAUAUGGUAG	UUAUUACAUCAGCUACACCCCC	Cleavage	1
trf_2_72	Bra014489	2	21.1	1	20	168	187	GGUCCCAUGGUCUAGCGGUU	AGCUGCUAGACCUUGGGACC	Cleavage	1
trf_2_72	Bra007535	3	20.1	1	20	51	70	GGUCCCAUGGUCUAGCGGUU	GGCUGCUAGACCUUGGGAUC	Cleavage	1
trf_2_73	Bra013528	3	22.0	1	20	200	219	UCCGUUGUAGUCUAGCUGGU	AUCAGCUUGGCUAUAACGGG	Cleavage	1

trf_2_74	Bra007154	2.5	14.1	1	21	510	530	GCACCAGUGGUCUAGUGGUAG	UUACUACUGGACCUUUGGUGC	Cleavage	1
trf_2_75	Bra007154	2.5	14.1	1	21	510	530	GCACCAGUGGUCUAGUGGUAG	UUACUACUGGACCUUUGGUGC	Cleavage	1
trf_2_76	Bra009876	3	14.9	1	22	16	37	GGGGAUGUAGCUCAGAUGGUAG	UUAUCAUCUGGGCUUCGUCCUC	Cleavage	1
trf_2_76	Bra016141	3	10.4	1	22	3031	3052	GGGGAUGUAGCUCAGAUGGUAG	UUAUCAUCUGAGCUUCGUUCUC	Cleavage	1
trf_2_76	Bra036771	3	20.2	1	23	5375	5397	GGGGAUGUAGCUCAGAUGGUAGA	UCGACUGGCUGAGCUACAUUCCU	Cleavage	1
trf_2_76	Bra018620	3	8.3	1	20	7	26	GGGGAUGUAGCUCAGAUGGU	GCCACCUCAGCUACAUCCUC	Cleavage	1
trf_2_77	Bra013584	3	16.4	1	25	886	910	UCCGUUGUCGUCCAGCGGUUAGGAU	AUGAUAACCGAUGGAUGUCAAUGGA	Cleavage	1
trf_2_77	Bra000529	3	11.2	1	21	984	1004	UCCGUUGUCGUCCAGCGGUUA	UAACCGGAGGACGAUAAUGGA	Cleavage	1
trf_2_78	Bra013584	3	16.4	1	21	890	910	UCCGUUGUCGUCCAGCGGUUA	UAACCGAUGGAUGUCAAUGGA	Cleavage	1
trf_2_78	Bra000529	3	11.2	1	21	984	1004	UCCGUUGUCGUCCAGCGGUUA	UAACCGGAGGACGAUAAUGGA	Cleavage	1
trf_2_80	Bra013584	3	16.4	1	21	890	910	UCCGUUGUCGUCCAGCGGUUA	UAACCGAUGGAUGUCAAUGGA	Cleavage	1
trf_2_80	Bra000529	3	11.2	1	21	984	1004	UCCGUUGUCGUCCAGCGGUUA	UAACCGGAGGACGAUAAUGGA	Cleavage	1
trf_2_81	Bra013584	3	16.4	1	20	891	910	UCCGUUGUCGUCCAGCGGUU	AACCGAUGGAUGUCAAUGGA	Cleavage	1
trf_2_81	Bra000529	3	11.2	1	20	985	1004	UCCGUUGUCGUCCAGCGGUU	AACCGGAGGACGAUAAUGGA	Cleavage	1
trf_2_83	Bra013584	3	16.4	1	26	885	910	UCCGUUGUCGUCCAGCGGUUAGGAUA	UAUGAUAACCGAUGGAUGUCAAUGGA	Cleavage	1
trf_2_83	Bra000529	3	11.2	1	21	984	1004	UCCGUUGUCGUCCAGCGGUUA	UAACCGGAGGACGAUAAUGGA	Cleavage	1
trf_2_85	Bra013584	3	16.4	1	21	890	910	UCCGUUGUCGUCCAGCGGUUA	UAACCGAUGGAUGUCAAUGGA	Cleavage	1

trf_2_85	Bra000529	3	11.2	1	21	984	1004	UCCGUUGUCGUCCAGCGGUUA	UAACCGGAGGACGAUAAUGGA	Cleavage	1
trf_2_86	Bra013584	3	16.4	1	26	885	910	UCCGUUGUCGUCCAGCGGUUAGGAUA	UAUGAUAACCGAUGGAUGUCAAUGGA	Cleavage	1
trf_2_86	Bra000529	3	11.2	1	21	984	1004	UCCGUUGUCGUCCAGCGGUUA	UAACCGGAGGACGAUAAUGGA	Cleavage	1
trf_2_97	Bra005511	2.5	12.8	1	22	1113	1134	GUGGCUGUAGUUUAGUGGUGAG	CUCACCGCUGAACUAUGGCUAC	Cleavage	1
trf_2_97	Bra035454	3	17.9	1	22	1243	1264	GUGGCUGUAGUUUAGUGGUGAG	UUCAUCUCUAGGCUACAGCCAU	Cleavage	1
trf_2_97	Bra009047	3	12.1	1	20	243	262	GUGGCUGUAGUUUAGUGGUG	CAACAUUGAGCUACAGCCAU	Cleavage	1
trf_2_98	Bra005511	2.5	12.8	1	22	1113	1134	GUGGCUGUAGUUUAGUGGUGAG	CUCACCGCUGAACUAUGGCUAC	Cleavage	1
trf_2_98	Bra035454	3	17.9	1	24	1241	1264	GUGGCUGUAGUUUAGUGGUGAGAA	UAUUCAUCUCUAGGCUACAGCCAU	Cleavage	1
trf_2_98	Bra009047	3	12.1	1	20	243	262	GUGGCUGUAGUUUAGUGGUG	CAACAUUGAGCUACAGCCAU	Cleavage	1
trf_2_100	Bra000881	2.5	11.0	1	24	162	185	GUCUGGGUGGUGUAGUCGGUUAUC	GCUAACCAACUACACCAUCCAGAA	Cleavage	1
trf_2_100	Bra040072	3	13.8	1	21	504	524	GUCUGGGUGGUGUAGUCGGUU	AGCUGACUCCAUCACUCAGAC	Cleavage	1
trf_2_100	Bra012326	3	15.0	1	20	1326	1345	GUCUGGGUGGUGUAGUCGGU	ACCGGUUGCACCGUCCGGAC	Cleavage	1
trf_2_100	Bra003121	3	15.1	1	24	760	783	GUCUGGGUGGUGUAGUCGGUUAUC	GCUCACCAGCUACACCACACAGAC	Cleavage	1
trf_2_116	Bra024638	3	7.5	1	23	203	225	GGGGAUGUAGCUCAAAUGGUAGA	UCCAACAUUUCGGUUACAUCCCC	Cleavage	1
trf_2_116	Bra025904	3	17.2	1	20	344	363	GGGGAUGUAGCUCAAAUGGU	ACAAUUUGAGCUUCAUCUUC	Cleavage	1
trf_2_117	Bra014507	3	17.0	1	20	544	563	GGGGAUGUAGCUCAUAUGGU	AUUAUAUUAGCUAUAUCUCC	Cleavage	1
trf_2_118	Bra014507	3	17.0	1	23	541	563	GGGGAUGUAGCUCAUAUGGUAGA	UGUAUUAUAUUAGCUAUAUCUCC	Cleavage	1

trf_2_119	Bra014507	3	17.0	1	24	540	563	GGGGAUGUAGCUCAUAUGGUAGAG	UUGUAUUAUAUUAGCUAUAUCUCC	Cleavage	1
trf_2_120	Bra024638	3	7.5	1	23	203	225	GGGGAUGUAGCUCAAAUGGUAGA	UCCAACAUUUCGGUUACAUCCCC	Cleavage	1
trf_2_120	Bra025904	3	17.2	1	20	344	363	GGGGAUGUAGCUCAAAUGGU	ACAAUUUGAGCUUCAUCUUC	Cleavage	1
trf_2_121	Bra024638	3	7.5	1	24	202	225	GGGGAUGUAGCUCAAAUGGUAGAG	UUCCAACAUUUCGGUUACAUCCCC	Cleavage	1
trf_2_121	Bra025904	3	17.2	1	20	344	363	GGGGAUGUAGCUCAAAUGGU	ACAAUUUGAGCUUCAUCUUC	Cleavage	1
trf_2_122	Bra019760	3	12.5	1	23	1328	1350	GACGGUUUGGCCGAGUGGUCUAA	UGAGACUACUUGGUCCAGCCGUC	Cleavage	1
trf_2_122	Bra000788	3	16.6	1	20	52	71	GACGGUUUGGCCGAGUGGUC	AACCAUUCCGCCAAAUCGUC	Cleavage	1
trf_2_122	Bra003534	3	24.7	1	20	3589	3608	GACGGUUUGGCCGAGUGGUC	GGCGAUUCGGCCAAAUUGUC	Cleavage	1
trf_2_122	Bra002609	3	10.2	1	20	1237	1256	GACGGUUUGGCCGAGUGGUC	GACUUUCCGGCCAAACCGUC	Cleavage	1
trf_2_123	Bra014489	2	21.1	1	20	168	187	GGUCCCAUGGUCUAGCGGUU	AGCUGCUAGACCUUGGGACC	Cleavage	1
trf_2_123	Bra007535	3	20.1	1	20	51	70	GGUCCCAUGGUCUAGCGGUU	GGCUGCUAGACCUUGGGAUC	Cleavage	1
trf_2_124	Bra014489	2	21.1	1	20	168	187	GGUCCCAUGGUCUAGCGGUU	AGCUGCUAGACCUUGGGACC	Cleavage	1
trf_2_124	Bra007535	3	20.1	1	20	51	70	GGUCCCAUGGUCUAGCGGUU	GGCUGCUAGACCUUGGGAUC	Cleavage	1
trf_2_126	Bra024638	3	7.5	1	24	202	225	GGGGAUGUAGCUCAAAUGGUAGAG	UUCCAACAUUUCGGUUACAUCCCC	Cleavage	1
trf_2_126	Bra025904	3	17.2	1	20	344	363	GGGGAUGUAGCUCAAAUGGU	ACAAUUUGAGCUUCAUCUUC	Cleavage	1
trf_2_127	Bra024638	3	7.5	1	23	203	225	GGGGAUGUAGCUCAAAUGGUAGA	UCCAACAUUUCGGUUACAUCCCC	Cleavage	1
trf_2_127	Bra025904	3	17.2	1	20	344	363	GGGGAUGUAGCUCAAAUGGU	ACAAUUUGAGCUUCAUCUUC	Cleavage	1

trf_2_128	Bra009876	3	14.9	1	22	16	37	GGGGAUGUAGCUCAGAUGGUAG	UUAUCAUCUGGGCUUCGUCCUC	Cleavage	1
trf_2_128	Bra016141	3	10.4	1	22	3031	3052	GGGGAUGUAGCUCAGAUGGUAG	UUAUCAUCUGAGCUUCGUUCUC	Cleavage	1
trf_2_128	Bra036771	3	20.2	1	23	5375	5397	GGGGAUGUAGCUCAGAUGGUAGA	UCGACUGGCUGAGCUACAUUCCU	Cleavage	1
trf_2_128	Bra018620	3	8.3	1	20	7	26	GGGGAUGUAGCUCAGAUGGU	GCCACCUCAGCUACAUCCUC	Cleavage	1
trf_2_129	Bra009876	3	14.9	1	25	13	37	GGGGAUGUAGCUCAGAUGGUAGAGC	GCGUUAUCAUCUGGGCUUCGUCCUC	Cleavage	1
trf_2_129	Bra036771	3	20.2	1	24	5374	5397	GGGGAUGUAGCUCAGAUGGUAGAG	CUCGACUGGCUGAGCUACAUUCCU	Cleavage	1
trf_2_129	Bra016141	3	10.4	1	24	3029	3052	GGGGAUGUAGCUCAGAUGGUAGAG	CAUUAUCAUCUGAGCUUCGUUCUC	Cleavage	1
trf_2_129	Bra018620	3	8.3	1	20	7	26	GGGGAUGUAGCUCAGAUGGU	GCCACCUCAGCUACAUCCUC	Cleavage	1
trf_2_130	Bra007154	2.5	14.1	1	21	510	530	GCACCAGUGGUCUAGUGGUAG	UUACUACUGGACCUUUGGUGC	Cleavage	1
trf_2_131	Bra007154	2.5	14.1	1	21	510	530	GCACCAGUGGUCUAGUGGUAG	UUACUACUGGACCUUUGGUGC	Cleavage	1
trf_2_133	Bra000719	3	15.3	1	20	1680	1699	GGUUCUAUGGUCUAGCGGUU	GACCGCUGGACCAGAGAACU	Cleavage	1
trf_2_133	Bra014489	3	21.1	1	20	168	187	GGUUCUAUGGUCUAGCGGUU	AGCUGCUAGACCUUGGGACC	Cleavage	1
trf_2_142	Bra005511	2.5	12.8	1	22	1113	1134	GUGGCUGUAGUUUAGUGGUGAG	CUCACCGCUGAACUAUGGCUAC	Cleavage	1
trf_2_142	Bra035454	3	17.9	1	24	1241	1264	GUGGCUGUAGUUUAGUGGUGAGAA	UAUUCAUCUCUAGGCUACAGCCAU	Cleavage	1
trf_2_142	Bra009047	3	12.1	1	20	243	262	GUGGCUGUAGUUUAGUGGUG	CAACAUUGAGCUACAGCCAU	Cleavage	1
trf_2_147	Bra007154	2.5	14.1	1	21	510	530	GCACCAGUGGUCUAGUGGUAG	UUACUACUGGACCUUUGGUGC	Cleavage	1
trf_2_149	Bra009876	3	14.9	1	22	16	37	GGGGAUGUAGCUCAGAUGGUAG	UUAUCAUCUGGGCUUCGUCCUC	Cleavage	1

trf_2_149	Bra016141	3	10.4	1	22	3031	3052	GGGGAUGUAGCUCAGAUGGUAG	UUAUCAUCUGAGCUUCGUUCUC	Cleavage	1
trf_2_149	Bra036771	3	20.2	1	23	5375	5397	GGGGAUGUAGCUCAGAUGGUAGA	UCGACUGGCUGAGCUACAUUCCU	Cleavage	1
trf_2_149	Bra018620	3	8.3	1	20	7	26	GGGGAUGUAGCUCAGAUGGU	GCCACCUCAGCUACAUCCUC	Cleavage	1
trf_2_150	Bra007154	2.5	14.1	1	21	510	530	GCACCAGUGGUCUAGUGGUAG	UUACUACUGGACCUUUGGUGC	Cleavage	1
trf_2_152	Bra013528	3	22.0	1	24	196	219	UCCGUUGUAGUCUAGCUGGUCAGG	CAUCAUCAGCUUGGCUAUAACGGG	Cleavage	1
trf_2_153	Bra009876	3	14.9	1	22	16	37	GGGGAUGUAGCUCAGAUGGUAG	UUAUCAUCUGGGCUUCGUCCUC	Cleavage	1
trf_2_153	Bra016141	3	10.4	1	22	3031	3052	GGGGAUGUAGCUCAGAUGGUAG	UUAUCAUCUGAGCUUCGUUCUC	Cleavage	1
trf_2_153	Bra036771	3	20.2	1	23	5375	5397	GGGGAUGUAGCUCAGAUGGUAGA	UCGACUGGCUGAGCUACAUUCCU	Cleavage	1
trf_2_153	Bra018620	3	8.3	1	20	7	26	GGGGAUGUAGCUCAGAUGGU	GCCACCUCAGCUACAUCCUC	Cleavage	1
trf_2_154	Bra007154	2.5	14.1	1	21	510	530	GCACCAGUGGUCUAGUGGUAG	UUACUACUGGACCUUUGGUGC	Cleavage	1
trf_2_155	Bra009876	3	14.9	1	22	16	37	GGGGAUGUAGCUCAGAUGGUAG	UUAUCAUCUGGGCUUCGUCCUC	Cleavage	1
trf_2_155	Bra016141	3	10.4	1	22	3031	3052	GGGGAUGUAGCUCAGAUGGUAG	UUAUCAUCUGAGCUUCGUUCUC	Cleavage	1
trf_2_155	Bra036771	3	20.2	1	23	5375	5397	GGGGAUGUAGCUCAGAUGGUAGA	UCGACUGGCUGAGCUACAUUCCU	Cleavage	1
trf_2_155	Bra018620	3	8.3	1	20	7	26	GGGGAUGUAGCUCAGAUGGU	GCCACCUCAGCUACAUCCUC	Cleavage	1
trf_2_164	Bra009876	3	14.9	1	22	16	37	GGGGAUGUAGCUCAGAUGGUAG	UUAUCAUCUGGGCUUCGUCCUC	Cleavage	1
trf_2_164	Bra016141	3	10.4	1	22	3031	3052	GGGGAUGUAGCUCAGAUGGUAG	UUAUCAUCUGAGCUUCGUUCUC	Cleavage	1
trf_2_164	Bra036771	3	20.2	1	23	5375	5397	GGGGAUGUAGCUCAGAUGGUAGA	UCGACUGGCUGAGCUACAUUCCU	Cleavage	1

trf_2_164	Bra018620	3	8.3	1	20	7	26	GGGGAUGUAGCUCAGAUGGU	GCCACCUCAGCUACAUCCUC	Cleavage	1
trf_2_173	Bra013528	3	22.0	1	24	196	219	UCCGUUGUAGUCUAGCUGGUCAGG	CAUCAUCAGCUUGGCUAUAACGGG	Cleavage	1
trf_2_182	Bra000719	3	15.3	1	20	1680	1699	GGUUCUAUGGUCUAGCGGUU	GACCGCUGGACCAGAGAACU	Cleavage	1
trf_2_182	Bra014489	3	21.1	1	20	168	187	GGUUCUAUGGUCUAGCGGUU	AGCUGCUAGACCUUGGGACC	Cleavage	1
trf_2_183	Bra000719	3	15.3	1	20	1680	1699	GGUUCUAUGGUCUAGCGGUU	GACCGCUGGACCAGAGAACU	Cleavage	1
trf_2_183	Bra014489	3	21.1	1	20	168	187	GGUUCUAUGGUCUAGCGGUU	AGCUGCUAGACCUUGGGACC	Cleavage	1
trf_2_186	Bra000529	2.5	11.2	1	21	984	1004	UCCGUUAUCGUCCAGCGGUUA	UAACCGGAGGACGAUAAUGGA	Cleavage	1
trf_2_186	Bra032111	3	22.4	1	20	727	746	UCCGUUAUCGUCCAGCGGUU	GACGGCUUGACGAUGACGGA	Cleavage	1
trf_2_187	Bra000529	2.5	11.2	1	21	984	1004	UCCGUUAUCGUCCAGCGGUUA	UAACCGGAGGACGAUAAUGGA	Cleavage	1
trf_2_187	Bra032111	3	22.4	1	20	727	746	UCCGUUAUCGUCCAGCGGUU	GACGGCUUGACGAUGACGGA	Cleavage	1
trf_2_188	Bra000529	2.5	11.2	1	21	984	1004	UCCGUUAUCGUCCAGCGGUUA	UAACCGGAGGACGAUAAUGGA	Cleavage	1
trf_2_188	Bra032111	3	22.4	1	20	727	746	UCCGUUAUCGUCCAGCGGUU	GACGGCUUGACGAUGACGGA	Cleavage	1
trf_2_190	Bra000529	2.5	11.2	1	20	985	1004	UCCGUUAUCGUCCAGCGGUU	AACCGGAGGACGAUAAUGGA	Cleavage	1
trf_2_190	Bra032111	3	22.4	1	20	727	746	UCCGUUAUCGUCCAGCGGUU	GACGGCUUGACGAUGACGGA	Cleavage	1
trf_2_192	Bra000529	2.5	11.2	1	21	984	1004	UCCGUUAUCGUCCAGCGGUUA	UAACCGGAGGACGAUAAUGGA	Cleavage	1
trf_2_192	Bra032111	3	22.4	1	20	727	746	UCCGUUAUCGUCCAGCGGUU	GACGGCUUGACGAUGACGGA	Cleavage	1
trf_2_193	Bra000529	2.5	11.2	1	21	984	1004	UCCGUUAUCGUCCAGCGGUUA	UAACCGGAGGACGAUAAUGGA	Cleavage	1

trf_2_193	Bra032111	3	22.4	1	20	727	746	UCCGUUAUCGUCCAGCGGUU	GACGGCUUGACGAUGACGGA	Cleavage	1
trf_2_194	Bra000529	2.5	11.2	1	21	984	1004	UCCGUUAUCGUCCAGCGGUUA	UAACCGGAGGACGAUAAUGGA	Cleavage	1
trf_2_194	Bra032111	3	22.4	1	20	727	746	UCCGUUAUCGUCCAGCGGUU	GACGGCUUGACGAUGACGGA	Cleavage	1
trf_2_195	Bra009876	3	14.9	1	22	16	37	GGGGAUGUAGCUCAGAUGGUAG	UUAUCAUCUGGGCUUCGUCCUC	Cleavage	1
trf_2_195	Bra016141	3	10.4	1	22	3031	3052	GGGGAUGUAGCUCAGAUGGUAG	UUAUCAUCUGAGCUUCGUUCUC	Cleavage	1
trf_2_195	Bra036771	3	20.2	1	23	5375	5397	GGGGAUGUAGCUCAGAUGGUAGA	UCGACUGGCUGAGCUACAUUCCU	Cleavage	1
trf_2_195	Bra018620	3	8.3	1	20	7	26	GGGGAUGUAGCUCAGAUGGU	GCCACCUCAGCUACAUCCUC	Cleavage	1
trf_2_208	Bra009876	3	14.9	1	22	16	37	GGGGAUGUAGCUCAGAUGGUAG	UUAUCAUCUGGGCUUCGUCCUC	Cleavage	1
trf_2_208	Bra016141	3	10.4	1	22	3031	3052	GGGGAUGUAGCUCAGAUGGUAG	UUAUCAUCUGAGCUUCGUUCUC	Cleavage	1
trf_2_208	Bra036771	3	20.2	1	23	5375	5397	GGGGAUGUAGCUCAGAUGGUAGA	UCGACUGGCUGAGCUACAUUCCU	Cleavage	1
trf_2_208	Bra018620	3	8.3	1	20	7	26	GGGGAUGUAGCUCAGAUGGU	GCCACCUCAGCUACAUCCUC	Cleavage	1
trf_2_210	Bra007154	2.5	14.1	1	21	510	530	GCACCAGUGGUCUAGUGGUAG	UUACUACUGGACCUUUGGUGC	Cleavage	1
trf_2_224	Bra013584	3	16.4	1	21	890	910	UCCGUUGUCGUCCAGCGGUUA	UAACCGAUGGAUGUCAAUGGA	Cleavage	1
trf_2_224	Bra000529	3	11.2	1	21	984	1004	UCCGUUGUCGUCCAGCGGUUA	UAACCGGAGGACGAUAAUGGA	Cleavage	1
trf_2_225	Bra013584	3	16.4	1	25	886	910	UCCGUUGUCGUCCAGCGGUUAGGAU	AUGAUAACCGAUGGAUGUCAAUGGA	Cleavage	1
trf_2_225	Bra000529	3	11.2	1	21	984	1004	UCCGUUGUCGUCCAGCGGUUA	UAACCGGAGGACGAUAAUGGA	Cleavage	1
trf_2_226	Bra013584	3	16.4	1	26	885	910	UCCGUUGUCGUCCAGCGGUUAGGAUA	UAUGAUAACCGAUGGAUGUCAAUGGA	Cleavage	1

trf_2_226	Bra000529	3	11.2	1	21	984	1004	UCCGUUGUCGUCCAGCGGUUA	UAACCGGAGGACGAUAAUGGA	Cleavage	1
trf_2_227	Bra013584	3	16.4	1	21	890	910	UCCGUUGUCGUCCAGCGGUUA	UAACCGAUGGAUGUCAAUGGA	Cleavage	1
trf_2_227	Bra000529	3	11.2	1	21	984	1004	UCCGUUGUCGUCCAGCGGUUA	UAACCGGAGGACGAUAAUGGA	Cleavage	1
trf_2_228	Bra013584	3	16.4	1	20	891	910	UCCGUUGUCGUCCAGCGGUU	AACCGAUGGAUGUCAAUGGA	Cleavage	1
trf_2_228	Bra000529	3	11.2	1	20	985	1004	UCCGUUGUCGUCCAGCGGUU	AACCGGAGGACGAUAAUGGA	Cleavage	1
trf_2_229	Bra013584	3	16.4	1	26	885	910	UCCGUUGUCGUCCAGCGGUUAGGAUA	UAUGAUAACCGAUGGAUGUCAAUGGA	Cleavage	1
trf_2_229	Bra000529	3	11.2	1	21	984	1004	UCCGUUGUCGUCCAGCGGUUA	UAACCGGAGGACGAUAAUGGA	Cleavage	1
trf_2_230	Bra013584	3	16.4	1	21	890	910	UCCGUUGUCGUCCAGCGGUUA	UAACCGAUGGAUGUCAAUGGA	Cleavage	1
trf_2_230	Bra000529	3	11.2	1	21	984	1004	UCCGUUGUCGUCCAGCGGUUA	UAACCGGAGGACGAUAAUGGA	Cleavage	1
trf_2_235	Bra007154	2.5	14.1	1	21	510	530	GCACCAGUGGUCUAGUGGUAG	UUACUACUGGACCUUUGGUGC	Cleavage	1
trf_2_236	Bra013528	3	22.0	1	24	196	219	UCCGUUGUAGUCUAGCUGGUCAGG	CAUCAUCAGCUUGGCUAUAACGGG	Cleavage	1
trf_2_237	Bra009876	3	14.9	1	22	16	37	GGGGAUGUAGCUCAGAUGGUAG	UUAUCAUCUGGGCUUCGUCCUC	Cleavage	1
trf_2_237	Bra016141	3	10.4	1	22	3031	3052	GGGGAUGUAGCUCAGAUGGUAG	UUAUCAUCUGAGCUUCGUUCUC	Cleavage	1
trf_2_237	Bra036771	3	20.2	1	23	5375	5397	GGGGAUGUAGCUCAGAUGGUAGA	UCGACUGGCUGAGCUACAUUCCU	Cleavage	1
trf_2_237	Bra018620	3	8.3	1	20	7	26	GGGGAUGUAGCUCAGAUGGU	GCCACCUCAGCUACAUCCUC	Cleavage	1
trf_2_238	Bra024316	3	20.4	1	25	431	455	GCGUCCAUUGUCUAAUGGAUAGGAC	GUUCUGUCCAUCAGAUACUGGACGC	Cleavage	1
trf_2_238	Bra007620	3	16.3	1	20	292	311	GCGUCCAUUGUCUAAUGGAU	AUCCGUCAGACAAUGGAAGC	Cleavage	1

trf_2_238	Bra010618	3	20.4	1	20	442	461	GCGUCCAUUGUCUAAUGGAU	AUGCAUCAGAUAGUGGACGC	Cleavage	1
trf_2_240	Bra024316	3	20.4	1	25	431	455	GCGUCCAUUGUCUAAUGGAUAGGAC	GUUCUGUCCAUCAGAUACUGGACGC	Cleavage	1
trf_2_240	Bra007620	3	16.3	1	20	292	311	GCGUCCAUUGUCUAAUGGAU	AUCCGUCAGACAAUGGAAGC	Cleavage	1
trf_2_240	Bra010618	3	20.4	1	20	442	461	GCGUCCAUUGUCUAAUGGAU	AUGCAUCAGAUAGUGGACGC	Cleavage	1
trf_2_241	Bra024316	3	20.4	1	24	432	455	GCGUCCAUUGUCUAAUGGAUAGGA	UUCUGUCCAUCAGAUACUGGACGC	Cleavage	1
trf_2_241	Bra007620	3	16.3	1	20	292	311	GCGUCCAUUGUCUAAUGGAU	AUCCGUCAGACAAUGGAAGC	Cleavage	1
trf_2_241	Bra010618	3	20.4	1	20	442	461	GCGUCCAUUGUCUAAUGGAU	AUGCAUCAGAUAGUGGACGC	Cleavage	1
trf_2_244	Bra007154	2.5	14.1	1	21	510	530	GCACCAGUGGUCUAGUGGUAG	UUACUACUGGACCUUUGGUGC	Cleavage	1
trf_2_245	Bra000881	2.5	11.0	1	24	162	185	GUCUGGGUGGUGUAGUCGGUUAUC	GCUAACCAACUACACCAUCCAGAA	Cleavage	1
trf_2_245	Bra040072	3	13.8	1	21	504	524	GUCUGGGUGGUGUAGUCGGUU	AGCUGACUCCAUCACUCAGAC	Cleavage	1
trf_2_245	Bra012326	3	15.0	1	20	1326	1345	GUCUGGGUGGUGUAGUCGGU	ACCGGUUGCACCGUCCGGAC	Cleavage	1
trf_2_245	Bra003121	3	15.1	1	24	760	783	GUCUGGGUGGUGUAGUCGGUUAUC	GCUCACCAGCUACACCACACAGAC	Cleavage	1
trf_2_246	Bra007154	2.5	14.1	1	21	510	530	GCACCAGUGGUCUAGUGGUAG	UUACUACUGGACCUUUGGUGC	Cleavage	1
trf_2_247	Bra009876	3	14.9	1	22	16	37	GGGGAUGUAGCUCAGAUGGUAG	UUAUCAUCUGGGCUUCGUCCUC	Cleavage	1
trf_2_247	Bra016141	3	10.4	1	22	3031	3052	GGGGAUGUAGCUCAGAUGGUAG	UUAUCAUCUGAGCUUCGUUCUC	Cleavage	1
trf_2_247	Bra036771	3	20.2	1	23	5375	5397	GGGGAUGUAGCUCAGAUGGUAGA	UCGACUGGCUGAGCUACAUUCCU	Cleavage	1
trf_2_247	Bra018620	3	8.3	1	20	7	26	GGGGAUGUAGCUCAGAUGGU	GCCACCUCAGCUACAUCCUC	Cleavage	1

trf_2_248	Bra013584	3	16.4	1	26	885	910	UCCGUUGUCGUCCAGCGGUUAGGAUA	UAUGAUAACCGAUGGAUGUCAAUGGA	Cleavage	1
trf_2_248	Bra000529	3	11.2	1	21	984	1004	UCCGUUGUCGUCCAGCGGUUA	UAACCGGAGGACGAUAAUGGA	Cleavage	1
trf_2_250	Bra013584	3	16.4	1	21	890	910	UCCGUUGUCGUCCAGCGGUUA	UAACCGAUGGAUGUCAAUGGA	Cleavage	1
trf_2_250	Bra000529	3	11.2	1	21	984	1004	UCCGUUGUCGUCCAGCGGUUA	UAACCGGAGGACGAUAAUGGA	Cleavage	1
trf_2_251	Bra013584	3	16.4	1	25	886	910	UCCGUUGUCGUCCAGCGGUUAGGAU	AUGAUAACCGAUGGAUGUCAAUGGA	Cleavage	1
trf_2_251	Bra000529	3	11.2	1	21	984	1004	UCCGUUGUCGUCCAGCGGUUA	UAACCGGAGGACGAUAAUGGA	Cleavage	1
trf_2_253	Bra013584	3	16.4	1	21	890	910	UCCGUUGUCGUCCAGCGGUUA	UAACCGAUGGAUGUCAAUGGA	Cleavage	1
trf_2_253	Bra000529	3	11.2	1	21	984	1004	UCCGUUGUCGUCCAGCGGUUA	UAACCGGAGGACGAUAAUGGA	Cleavage	1
trf_2_254	Bra013584	3	16.4	1	20	891	910	UCCGUUGUCGUCCAGCGGUU	AACCGAUGGAUGUCAAUGGA	Cleavage	1
trf_2_254	Bra000529	3	11.2	1	20	985	1004	UCCGUUGUCGUCCAGCGGUU	AACCGGAGGACGAUAAUGGA	Cleavage	1
trf_2_256	Bra013584	3	16.4	1	21	890	910	UCCGUUGUCGUCCAGCGGUUA	UAACCGAUGGAUGUCAAUGGA	Cleavage	1
trf_2_256	Bra000529	3	11.2	1	21	984	1004	UCCGUUGUCGUCCAGCGGUUA	UAACCGGAGGACGAUAAUGGA	Cleavage	1
trf_2_257	Bra013584	3	16.4	1	26	885	910	UCCGUUGUCGUCCAGCGGUUAGGAUA	UAUGAUAACCGAUGGAUGUCAAUGGA	Cleavage	1
trf_2_257	Bra000529	3	11.2	1	21	984	1004	UCCGUUGUCGUCCAGCGGUUA	UAACCGGAGGACGAUAAUGGA	Cleavage	1
trf_2_258	Bra014507	3	17.0	1	23	541	563	GGGGAUGUAGCUCAUAUGGUAGA	UGUAUUAUAUUAGCUAUAUCUCC	Cleavage	1
trf_2_259	Bra014507	3	17.0	1	20	544	563	GGGGAUGUAGCUCAUAUGGU	AUUAUAUUAGCUAUAUCUCC	Cleavage	1
trf_2_260	Bra016601	3	20.8	1	24	415	438	UCCGUCGUAGUCUAGCUGGUUAGG	CUGAAGAAGCUAGGCUGCGACGGA	Cleavage	1

trf_2_262	Bra016601	3	20.8	1	24	415	438	UCCGUCGUAGUCUAGCUGGUUAGG	CUGAAGAAGCUAGGCUGCGACGGA	Cleavage	1
trf_2_263	Bra016601	3	20.8	1	21	418	438	UCCGUCGUAGUCUAGCUGGUU	AAGAAGCUAGGCUGCGACGGA	Cleavage	1
trf_2_265	Bra016601	3	20.8	1	24	415	438	UCCGUCGUAGUCUAGCUGGUUAGG	CUGAAGAAGCUAGGCUGCGACGGA	Cleavage	1
trf_2_266	Bra016601	3	20.8	1	21	418	438	UCCGUCGUAGUCUAGCUGGUU	AAGAAGCUAGGCUGCGACGGA	Cleavage	1
trf_2_267	Bra016601	3	20.8	1	21	418	438	UCCGUCGUAGUCUAGCUGGUU	AAGAAGCUAGGCUGCGACGGA	Cleavage	1
trf_2_268	Bra005511	2.5	12.8	1	22	1113	1134	GUGGCUGUAGUUUAGUGGUGAG	CUCACCGCUGAACUAUGGCUAC	Cleavage	1
trf_2_268	Bra035454	3	17.9	1	24	1241	1264	GUGGCUGUAGUUUAGUGGUGAGAA	UAUUCAUCUCUAGGCUACAGCCAU	Cleavage	1
trf_2_268	Bra009047	3	12.1	1	20	243	262	GUGGCUGUAGUUUAGUGGUG	CAACAUUGAGCUACAGCCAU	Cleavage	1
trf_2_269	Bra009876	3	14.9	1	22	16	37	GGGGAUGUAGCUCAGAUGGUAG	UUAUCAUCUGGGCUUCGUCCUC	Cleavage	1
trf_2_269	Bra016141	3	10.4	1	22	3031	3052	GGGGAUGUAGCUCAGAUGGUAG	UUAUCAUCUGAGCUUCGUUCUC	Cleavage	1
trf_2_269	Bra036771	3	20.2	1	23	5375	5397	GGGGAUGUAGCUCAGAUGGUAGA	UCGACUGGCUGAGCUACAUUCCU	Cleavage	1
trf_2_269	Bra018620	3	8.3	1	20	7	26	GGGGAUGUAGCUCAGAUGGU	GCCACCUCAGCUACAUCCUC	Cleavage	1
trf_2_273	Bra009876	3	14.9	1	22	16	37	GGGGAUGUAGCUCAGAUGGUAG	UUAUCAUCUGGGCUUCGUCCUC	Cleavage	1
trf_2_273	Bra016141	3	10.4	1	22	3031	3052	GGGGAUGUAGCUCAGAUGGUAG	UUAUCAUCUGAGCUUCGUUCUC	Cleavage	1
trf_2_273	Bra036771	3	20.2	1	23	5375	5397	GGGGAUGUAGCUCAGAUGGUAGA	UCGACUGGCUGAGCUACAUUCCU	Cleavage	1
trf_2_273	Bra018620	3	8.3	1	20	7	26	GGGGAUGUAGCUCAGAUGGU	GCCACCUCAGCUACAUCCUC	Cleavage	1
trf_2_275	Bra020835	3	9.5	1	22	5316	5337	GGGGGUGUAGCUCAUAUGGUAG	UUAUUACAUCAGCUACACCCCC	Cleavage	1

trf_2_2	Bra009		14					GGGGAUGUAGCUCAGA	UUAUCAUCUGGGCUUC	Cleava	
77	876	3	.9	1	22	16	37	UGGUAG	GUCCUC	ge	1
trf_2_2	Bra016		10					GGGGAUGUAGCUCAGA	UUAUCAUCUGAGCUUC	Cleava	
77	141	3	.4	1	22	3031	3052	UGGUAG	GUUCUC	ge	1
trf_2_2	Bra036		20					GGGGAUGUAGCUCAGA	UCGACUGGCUGAGCUA	Cleava	
77	771	3	.2	1	23	5375	5397	UGGUAGA	CAUUCCU	ge	1
trf_2_2	Bra018		8.					GGGGAUGUAGCUCAGA	GCCACCUCAGCUACAUC	Cleava	
77	620	3	3	1	20	7	26	UGGU	CUC	ge	1
trf_2_2	Bra009		14					GGGGAUGUAGCUCAGA	UUAUCAUCUGGGCUUC	Cleava	
79	876	3	.9	1	22	16	37	UGGUAG	GUCCUC	ge	1
trf_2_2	Bra016		10					GGGGAUGUAGCUCAGA	UUAUCAUCUGAGCUUC	Cleava	
79	141	3	.4	1	22	3031	3052	UGGUAG	GUUCUC	ge	1
trf_2_2	Bra036		20					GGGGAUGUAGCUCAGA	UCGACUGGCUGAGCUA	Cleava	
79	771	3	.2	1	23	5375	5397	UGGUAGA	CAUUCCU	ge	1
trf_2_2	Bra018		8.					GGGGAUGUAGCUCAGA	GCCACCUCAGCUACAUC	Cleava	
79	620	3	3	1	20	7	26	UGGU	CUC	ge	1
trf_2_2	Bra009		14					GGGGAUGUAGCUCAGA	UUAUCAUCUGGGCUUC	Cleava	
83	876	3	.9	1	22	16	37	UGGUAG	GUCCUC	ge	1
trf_2_2	Bra016		10					GGGGAUGUAGCUCAGA	UUAUCAUCUGAGCUUC	Cleava	
83	141	3	.4	1	22	3031	3052	UGGUAG	GUUCUC	ge	1
trf_2_2	Bra036		20					GGGGAUGUAGCUCAGA	UCGACUGGCUGAGCUA	Cleava	
83	771	3	.2	1	23	5375	5397	UGGUAGA	CAUUCCU	ge	1
trf_2_2	Bra018		8.					GGGGAUGUAGCUCAGA	GCCACCUCAGCUACAUC	Cleava	
83	620	3	3	1	20	7	26	UGGU	CUC	ge	1
trf_2_2	Bra007		14					GCACCAGUGGUCUAGU	UUACUACUGGACCUUU	Cleava	
84	154	2.5	.1	1	21	510	530	GGUAG	GGUGC	ge	1
trf_2_2	Bra009		14					GGGGAUGUAGCUCAGA	UUAUCAUCUGGGCUUC	Cleava	
87	876	3	.9	1	22	16	37	UGGUAG	GUCCUC	ge	1
trf_2_2	Bra016		10					GGGGAUGUAGCUCAGA	UUAUCAUCUGAGCUUC	Cleava	
87	141	3	.4	1	22	3031	3052	UGGUAG	GUUCUC	ge	1
trf_2_2	Bra036		20					GGGGAUGUAGCUCAGA	UCGACUGGCUGAGCUA	Cleava	
87	771	3	.2	1	23	5375	5397	UGGUAGA	CAUUCCU	ge	1
trf_2_2	Bra018		8.					GGGGAUGUAGCUCAGA	GCCACCUCAGCUACAUC	Cleava	
87	620	3	3	1	20	7	26	UGGU	CUC	ge	1

trf_2_288	Bra024638	3	7.5	1	24	202	225	GGGGAUGUAGCUCAAAUGGUAGAG	UUCCAACAUUUCGGUUACAUCCCC	Cleavage	1
trf_2_288	Bra025904	3	17.2	1	20	344	363	GGGGAUGUAGCUCAAAUGGU	ACAAUUUGAGCUUCAUCUUC	Cleavage	1
trf_2_289	Bra024638	3	7.5	1	23	203	225	GGGGAUGUAGCUCAAAUGGUAGA	UCCAACAUUUCGGUUACAUCCCC	Cleavage	1
trf_2_289	Bra025904	3	17.2	1	20	344	363	GGGGAUGUAGCUCAAAUGGU	ACAAUUUGAGCUUCAUCUUC	Cleavage	1
trf_2_290	Bra009876	3	14.9	1	22	16	37	GGGGAUGUAGCUCAGAUGGUAG	UUAUCAUCUGGGCUUCGUCCUC	Cleavage	1
trf_2_290	Bra016141	3	10.4	1	22	3031	3052	GGGGAUGUAGCUCAGAUGGUAG	UUAUCAUCUGAGCUUCGUUCUC	Cleavage	1
trf_2_290	Bra036771	3	20.2	1	23	5375	5397	GGGGAUGUAGCUCAGAUGGUAGA	UCGACUGGCUGAGCUACAUUCCU	Cleavage	1
trf_2_290	Bra018620	3	8.3	1	20	7	26	GGGGAUGUAGCUCAGAUGGU	GCCACCUCAGCUACAUCCUC	Cleavage	1
trf_2_301	Bra009876	3	14.9	1	22	16	37	GGGGAUGUAGCUCAGAUGGUAG	UUAUCAUCUGGGCUUCGUCCUC	Cleavage	1
trf_2_301	Bra016141	3	10.4	1	22	3031	3052	GGGGAUGUAGCUCAGAUGGUAG	UUAUCAUCUGAGCUUCGUUCUC	Cleavage	1
trf_2_301	Bra036771	3	20.2	1	23	5375	5397	GGGGAUGUAGCUCAGAUGGUAGA	UCGACUGGCUGAGCUACAUUCCU	Cleavage	1
trf_2_301	Bra018620	3	8.3	1	20	7	26	GGGGAUGUAGCUCAGAUGGU	GCCACCUCAGCUACAUCCUC	Cleavage	1
trf_2_302	Bra036771	3	20.2	1	24	5374	5397	GGGGAUGUAGCUCAGAUGGUAGAG	CUCGACUGGCUGAGCUACAUUCCU	Cleavage	1
trf_2_302	Bra009876	3	14.9	1	24	14	37	GGGGAUGUAGCUCAGAUGGUAGAG	CGUUAUCAUCUGGGCUUCGUCCUC	Cleavage	1
trf_2_302	Bra016141	3	10.4	1	24	3029	3052	GGGGAUGUAGCUCAGAUGGUAGAG	CAUUAUCAUCUGAGCUUCGUUCUC	Cleavage	1
trf_2_302	Bra018620	3	8.3	1	20	7	26	GGGGAUGUAGCUCAGAUGGU	GCCACCUCAGCUACAUCCUC	Cleavage	1
trf_2_306	Bra009876	3	14.9	1	22	16	37	GGGGAUGUAGCUCAGAUGGUAG	UUAUCAUCUGGGCUUCGUCCUC	Cleavage	1

trf_2_306	Bra016141	3	10.4	1	22	3031	3052	GGGGAUGUAGCUCAGAUGGUAG	UUAUCAUCUGAGCUUCGUUCUC	Cleavage	1
trf_2_306	Bra036771	3	20.2	1	23	5375	5397	GGGGAUGUAGCUCAGAUGGUAGA	UCGACUGGCUGAGCUACAUUCCU	Cleavage	1
trf_2_306	Bra018620	3	8.3	1	20	7	26	GGGGAUGUAGCUCAGAUGGU	GCCACCUCAGCUACAUCCUC	Cleavage	1
trf_2_307	Bra009876	3	14.9	1	25	13	37	GGGGAUGUAGCUCAGAUGGUAGAGC	GCGUUAUCAUCUGGGCUUCGUCCUC	Cleavage	1
trf_2_307	Bra036771	3	20.2	1	24	5374	5397	GGGGAUGUAGCUCAGAUGGUAGAG	CUCGACUGGCUGAGCUACAUUCCU	Cleavage	1
trf_2_307	Bra016141	3	10.4	1	24	3029	3052	GGGGAUGUAGCUCAGAUGGUAGAG	CAUUAUCAUCUGAGCUUCGUUCUC	Cleavage	1
trf_2_307	Bra018620	3	8.3	1	20	7	26	GGGGAUGUAGCUCAGAUGGU	GCCACCUCAGCUACAUCCUC	Cleavage	1
trf_2_308	Bra024638	3	7.5	1	23	203	225	GGGGAUGUAGCUCAAAUGGUAGA	UCCAACAUUUCGGUUACAUCCCC	Cleavage	1
trf_2_308	Bra025904	3	17.2	1	20	344	363	GGGGAUGUAGCUCAAAUGGU	ACAAUUUGAGCUUCAUCUUC	Cleavage	1
trf_2_310	Bra009876	3	14.9	1	22	16	37	GGGGAUGUAGCUCAGAUGGUAG	UUAUCAUCUGGGCUUCGUCCUC	Cleavage	1
trf_2_310	Bra016141	3	10.4	1	22	3031	3052	GGGGAUGUAGCUCAGAUGGUAG	UUAUCAUCUGAGCUUCGUUCUC	Cleavage	1
trf_2_310	Bra036771	3	20.2	1	23	5375	5397	GGGGAUGUAGCUCAGAUGGUAGA	UCGACUGGCUGAGCUACAUUCCU	Cleavage	1
trf_2_310	Bra018620	3	8.3	1	20	7	26	GGGGAUGUAGCUCAGAUGGU	GCCACCUCAGCUACAUCCUC	Cleavage	1
trf_2_311	Bra009876	3	14.9	1	25	13	37	GGGGAUGUAGCUCAGAUGGUAGAGC	GCGUUAUCAUCUGGGCUUCGUCCUC	Cleavage	1
trf_2_311	Bra036771	3	20.2	1	24	5374	5397	GGGGAUGUAGCUCAGAUGGUAGAG	CUCGACUGGCUGAGCUACAUUCCU	Cleavage	1
trf_2_311	Bra016141	3	10.4	1	24	3029	3052	GGGGAUGUAGCUCAGAUGGUAGAG	CAUUAUCAUCUGAGCUUCGUUCUC	Cleavage	1
trf_2_311	Bra018620	3	8.3	1	20	7	26	GGGGAUGUAGCUCAGAUGGU	GCCACCUCAGCUACAUCCUC	Cleavage	1

trf_2_312	Bra009876	3	14.9	1	22	16	37	GGGGAUGUAGCUCAGAUGGUAG	UUAUCAUCUGGGCUUCGUCCUC	Cleavage	1
trf_2_312	Bra016141	3	10.4	1	22	3031	3052	GGGGAUGUAGCUCAGAUGGUAG	UUAUCAUCUGAGCUUCGUUCUC	Cleavage	1
trf_2_312	Bra036771	3	20.2	1	23	5375	5397	GGGGAUGUAGCUCAGAUGGUAGA	UCGACUGGCUGAGCUACAUUCCU	Cleavage	1
trf_2_312	Bra018620	3	8.3	1	20	7	26	GGGGAUGUAGCUCAGAUGGU	GCCACCUCAGCUACAUCCUC	Cleavage	1
trf_2_314	Bra014507	3	17.0	1	23	541	563	GGGGAUGUAGCUCAUAUGGUAGA	UGUAUUAUAUUAGCUAUAUCUCC	Cleavage	1
trf_2_315	Bra014507	3	17.0	1	20	544	563	GGGGAUGUAGCUCAUAUGGU	AUUAUAUUAGCUAUAUCUCC	Cleavage	1
trf_2_316	Bra009876	3	14.9	1	22	16	37	GGGGAUGUAGCUCAGAUGGUAG	UUAUCAUCUGGGCUUCGUCCUC	Cleavage	1
trf_2_316	Bra016141	3	10.4	1	22	3031	3052	GGGGAUGUAGCUCAGAUGGUAG	UUAUCAUCUGAGCUUCGUUCUC	Cleavage	1
trf_2_316	Bra036771	3	20.2	1	23	5375	5397	GGGGAUGUAGCUCAGAUGGUAGA	UCGACUGGCUGAGCUACAUUCCU	Cleavage	1
trf_2_316	Bra018620	3	8.3	1	20	7	26	GGGGAUGUAGCUCAGAUGGU	GCCACCUCAGCUACAUCCUC	Cleavage	1
trf_2_317	Bra009876	3	14.9	1	22	16	37	GGGGAUGUAGCUCAGAUGGUAG	UUAUCAUCUGGGCUUCGUCCUC	Cleavage	1
trf_2_317	Bra016141	3	10.4	1	22	3031	3052	GGGGAUGUAGCUCAGAUGGUAG	UUAUCAUCUGAGCUUCGUUCUC	Cleavage	1
trf_2_317	Bra036771	3	20.2	1	23	5375	5397	GGGGAUGUAGCUCAGAUGGUAGA	UCGACUGGCUGAGCUACAUUCCU	Cleavage	1
trf_2_317	Bra018620	3	8.3	1	20	7	26	GGGGAUGUAGCUCAGAUGGU	GCCACCUCAGCUACAUCCUC	Cleavage	1
trf_2_318	Bra009876	3	14.9	1	22	16	37	GGGGAUGUAGCUCAGAUGGUAG	UUAUCAUCUGGGCUUCGUCCUC	Cleavage	1
trf_2_318	Bra016141	3	10.4	1	22	3031	3052	GGGGAUGUAGCUCAGAUGGUAG	UUAUCAUCUGAGCUUCGUUCUC	Cleavage	1
trf_2_318	Bra036771	3	20.2	1	23	5375	5397	GGGGAUGUAGCUCAGAUGGUAGA	UCGACUGGCUGAGCUACAUUCCU	Cleavage	1

trf_2_318	Bra018620	3	8.3	1	20	7	26	GGGGAUGUAGCUCAGAUGGU	GCCACCUCAGCUACAUCCUC	Cleavage	1
trf_2_319	Bra007154	2.5	14.1	1	21	510	530	GCACCAGUGGUCUAGUGGUAG	UUACUACUGGACCUUUGGUGC	Cleavage	1
trf_2_320	Bra003753	2.5	16.2	1	21	283	303	GGGAUUGUAGUUCAAUUGGCU	AACCAGUUGAAUUACAAUCCU	Cleavage	1
trf_2_321	Bra003753	1.5	16.2	1	20	284	303	GGGAUUGUAGUUCAAUUGGU	ACCAGUUGAAUUACAAUCCU	Cleavage	1
trf_2_321	Bra013336	3	19.5	1	20	267	286	GGGAUUGUAGUUCAAUUGGU	ACUAAUUGAACUAAGAUCUC	Cleavage	1
trf_2_321	Bra021314	3	23.2	1	20	234	253	GGGAUUGUAGUUCAAUUGGU	ACCAACUGAACUGCAAUCUG	Cleavage	1
trf_2_322	Bra003753	1.5	16.2	1	20	284	303	GGGAUUGUAGUUCAAUUGGU	ACCAGUUGAAUUACAAUCCU	Cleavage	1
trf_2_322	Bra013336	3	19.5	1	20	267	286	GGGAUUGUAGUUCAAUUGGU	ACUAAUUGAACUAAGAUCUC	Cleavage	1
trf_2_322	Bra021314	3	23.2	1	20	234	253	GGGAUUGUAGUUCAAUUGGU	ACCAACUGAACUGCAAUCUG	Cleavage	1
trf_2_323	Bra003753	1.5	16.2	1	20	284	303	GGGAUUGUAGUUCAAUUGGU	ACCAGUUGAAUUACAAUCCU	Cleavage	1
trf_2_323	Bra013336	3	19.5	1	20	267	286	GGGAUUGUAGUUCAAUUGGU	ACUAAUUGAACUAAGAUCUC	Cleavage	1
trf_2_323	Bra021314	3	23.2	1	20	234	253	GGGAUUGUAGUUCAAUUGGU	ACCAACUGAACUGCAAUCUG	Cleavage	1
trf_2_325	Bra003753	1.5	16.2	1	20	284	303	GGGAUUGUAGUUCAAUUGGU	ACCAGUUGAAUUACAAUCCU	Cleavage	1
trf_2_325	Bra013336	3	19.5	1	20	267	286	GGGAUUGUAGUUCAAUUGGU	ACUAAUUGAACUAAGAUCUC	Cleavage	1
trf_2_325	Bra021314	3	23.2	1	20	234	253	GGGAUUGUAGUUCAAUUGGU	ACCAACUGAACUGCAAUCUG	Cleavage	1
trf_2_326	Bra003753	2.5	16.2	1	20	284	303	GGGAUUGUAGUUCAAUCGGU	ACCAGUUGAAUUACAAUCCU	Cleavage	1
trf_2_327	Bra003753	1.5	16.2	1	20	284	303	GGGAUUGUAGUUCAAUUGGU	ACCAGUUGAAUUACAAUCCU	Cleavage	1

trf_2_327	Bra013336	3	19.5	1	20	267	286	GGGAUUGUAGUUCAAUUGGU	ACUAAUUGAACUAAGAUCUC	Cleavage	1
trf_2_327	Bra021314	3	23.2	1	20	234	253	GGGAUUGUAGUUCAAUUGGU	ACCAACUGAACUGCAAUCUG	Cleavage	1
trf_2_328	Bra003753	2.5	16.2	1	20	284	303	GGGAUUGUAGUUCAAUUGGC	ACCAGUUGAAUUACAAUCCU	Cleavage	1
trf_2_329	Bra003753	2.5	16.2	1	20	284	303	GGGAUUGUAGUUCAAUCGGU	ACCAGUUGAAUUACAAUCCU	Cleavage	1
trf_2_330	Bra003753	2.5	16.2	1	20	284	303	GGGAUUGUAGUUCAAUUGGA	ACCAGUUGAAUUACAAUCCU	Cleavage	1
trf_2_331	Bra003753	1.5	16.2	1	20	284	303	GGGAUUGUAGUUCAAUUGGU	ACCAGUUGAAUUACAAUCCU	Cleavage	1
trf_2_331	Bra013336	3	19.5	1	20	267	286	GGGAUUGUAGUUCAAUUGGU	ACUAAUUGAACUAAGAUCUC	Cleavage	1
trf_2_331	Bra021314	3	23.2	1	20	234	253	GGGAUUGUAGUUCAAUUGGU	ACCAACUGAACUGCAAUCUG	Cleavage	1
trf_2_333	Bra003753	1.5	16.2	1	21	283	303	GGGAUUGUAGUUCAAUUGGUU	AACCAGUUGAAUUACAAUCCU	Cleavage	1
trf_2_333	Bra013336	3	19.5	1	21	266	286	GGGAUUGUAGUUCAAUUGGUU	AACUAAUUGAACUAAGAUCUC	Cleavage	1
trf_2_333	Bra021314	3	23.2	1	20	234	253	GGGAUUGUAGUUCAAUUGGU	ACCAACUGAACUGCAAUCUG	Cleavage	1
trf_2_334	Bra003753	1.5	16.2	1	20	284	303	GGGAUUGUAGUUCAAUUGGU	ACCAGUUGAAUUACAAUCCU	Cleavage	1
trf_2_334	Bra013336	3	19.5	1	20	267	286	GGGAUUGUAGUUCAAUUGGU	ACUAAUUGAACUAAGAUCUC	Cleavage	1
trf_2_334	Bra021314	3	23.2	1	20	234	253	GGGAUUGUAGUUCAAUUGGU	ACCAACUGAACUGCAAUCUG	Cleavage	1
trf_2_336	Bra003753	2.5	16.2	1	20	284	303	GGGAUUGUAGUUCAAUUGGC	ACCAGUUGAAUUACAAUCCU	Cleavage	1
trf_2_337	Bra003753	2.5	16.2	1	20	284	303	GGGAUUGUAGUUCAAUUGGC	ACCAGUUGAAUUACAAUCCU	Cleavage	1
trf_2_338	Bra003753	2.5	16.2	1	21	283	303	GGGAUUGUAGUUCAAUUGGAU	AACCAGUUGAAUUACAAUCCU	Cleavage	1

trf_2_339	Bra003753	2.5	16.2	1	20	284	303	GGGAUUGUAGUUCAAUCGGU	ACCAGUUGAAUUACAAUCCU	Cleavage	1
trf_2_340	Bra003753	1.5	16.2	1	20	284	303	GGGAUUGUAGUUCAAUUGGU	ACCAGUUGAAUUACAAUCCU	Cleavage	1
trf_2_340	Bra013336	3	19.5	1	20	267	286	GGGAUUGUAGUUCAAUUGGU	ACUAAUUGAACUAAGAUCUC	Cleavage	1
trf_2_340	Bra021314	3	23.2	1	20	234	253	GGGAUUGUAGUUCAAUUGGU	ACCAACUGAACUGCAAUCUG	Cleavage	1
trf_2_341	Bra003753	2.5	16.2	1	20	284	303	GGGAUUGUAGUUCAAUCGGU	ACCAGUUGAAUUACAAUCCU	Cleavage	1
trf_2_342	Bra003753	2.5	16.2	1	20	284	303	GGGAUUGUAGUUCAAUCGGU	ACCAGUUGAAUUACAAUCCU	Cleavage	1
trf_2_343	Bra003753	1.5	16.2	1	20	284	303	GGGAUUGUAGUUCAAUUGGU	ACCAGUUGAAUUACAAUCCU	Cleavage	1
trf_2_343	Bra013336	3	19.5	1	20	267	286	GGGAUUGUAGUUCAAUUGGU	ACUAAUUGAACUAAGAUCUC	Cleavage	1
trf_2_343	Bra021314	3	23.2	1	20	234	253	GGGAUUGUAGUUCAAUUGGU	ACCAACUGAACUGCAAUCUG	Cleavage	1
trf_2_345	Bra003753	2.5	16.2	1	20	284	303	GGGAUUGUAGUUCAAUUGGG	ACCAGUUGAAUUACAAUCCU	Cleavage	1
trf_2_345	Bra009115	3	15.2	1	20	602	621	GGGAUUGUAGUUCAAUUGGG	CUCAGUUGAACUUCAAUCCA	Cleavage	1
trf_2_346	Bra003753	2.5	16.2	1	20	284	303	GGGAUUGUAGUUCAAUUGGG	ACCAGUUGAAUUACAAUCCU	Cleavage	1
trf_2_346	Bra009115	3	15.2	1	20	602	621	GGGAUUGUAGUUCAAUUGGG	CUCAGUUGAACUUCAAUCCA	Cleavage	1
trf_2_352	Bra010153	2.5	20.4	1	20	850	869	GCUUCAGUAGCUCGGAUGGC	GUCAUCCGAGCUGAUGAAGC	Cleavage	1
trf_2_352	Bra035649	3	17.6	1	20	423	442	GCUUCAGUAGCUCGGAUGGC	GCUGUCUGAGUUACUGAAGA	Cleavage	1
trf_2_352	Bra016307	2.5	16.1	1	20	1153	1172	GCUUCAGUAGCUCGGAUGGC	GCCAUCCAGGUUGCUGAAGC	Cleavage	1
trf_2_352	Bra029313	3	21.3	1	20	262	281	GCUUCAGUAGCUCGGAUGGC	ACUAACCGGGCUACUGAAGC	Cleavage	1

trf_2_352	Bra018588	3	12.1	1	20	897	916	GCUUCAGUAGCUCGGAUGGC	GGCAUCUGAGCUACUAAAGC	Cleavage	1
trf_2_352	Bra032316	3	23.9	1	20	880	899	GCUUCAGUAGCUCGGAUGGC	GAUGUUCGAGCUGCUGAAGC	Cleavage	1
trf_2_352	Bra008784	3	20.8	1	20	1057	1076	GCUUCAGUAGCUCGGAUGGC	GUCAUCGCAGCUGCUGAAGC	Cleavage	1
trf_2_355	Bra014507	3	17.0	1	23	541	563	GGGGAUGUAGCUCAUAUGGUAGA	UGUAUUAUAUUAGCUAUAUCUCC	Cleavage	1
trf_2_356	Bra009876	3	14.9	1	22	16	37	GGGGAUGUAGCUCAGAUGGUAG	UUAUCAUCUGGGCUUCGUCCUC	Cleavage	1
trf_2_356	Bra016141	3	10.4	1	22	3031	3052	GGGGAUGUAGCUCAGAUGGUAG	UUAUCAUCUGAGCUUCGUUCUC	Cleavage	1
trf_2_356	Bra036771	3	20.2	1	23	5375	5397	GGGGAUGUAGCUCAGAUGGUAGA	UCGACUGGCUGAGCUACAUUCCU	Cleavage	1
trf_2_356	Bra018620	3	8.3	1	20	7	26	GGGGAUGUAGCUCAGAUGGU	GCCACCUCAGCUACAUCCUC	Cleavage	1
trf_2_357	Bra009876	3	14.9	1	22	16	37	GGGGAUGUAGCUCAGAUGGUAG	UUAUCAUCUGGGCUUCGUCCUC	Cleavage	1
trf_2_357	Bra016141	3	10.4	1	22	3031	3052	GGGGAUGUAGCUCAGAUGGUAG	UUAUCAUCUGAGCUUCGUUCUC	Cleavage	1
trf_2_357	Bra036771	3	20.2	1	23	5375	5397	GGGGAUGUAGCUCAGAUGGUAGA	UCGACUGGCUGAGCUACAUUCCU	Cleavage	1
trf_2_357	Bra018620	3	8.3	1	20	7	26	GGGGAUGUAGCUCAGAUGGU	GCCACCUCAGCUACAUCCUC	Cleavage	1
trf_2_358	Bra014507	3	17.0	1	23	541	563	GGGGAUGUAGCUCAUAUGGUAGA	UGUAUUAUAUUAGCUAUAUCUCC	Cleavage	1
trf_2_359	Bra014507	3	17.0	1	20	544	563	GGGGAUGUAGCUCAUAUGGU	AUUAUAUUAGCUAUAUCUCC	Cleavage	1
trf_2_360	Bra009876	3	14.9	1	22	16	37	GGGGAUGUAGCUCAGAUGGUAG	UUAUCAUCUGGGCUUCGUCCUC	Cleavage	1
trf_2_360	Bra016141	3	10.4	1	22	3031	3052	GGGGAUGUAGCUCAGAUGGUAG	UUAUCAUCUGAGCUUCGUUCUC	Cleavage	1
trf_2_360	Bra036771	3	20.2	1	23	5375	5397	GGGGAUGUAGCUCAGAUGGUAGA	UCGACUGGCUGAGCUACAUUCCU	Cleavage	1

trf_2_3	Bra018		8.					GGGGAUGUAGCUCAGA	GCCACCUCAGCUACAUC	Cleava	
60	620	3	3	1	20	7	26	UGGU	CUC	ge	1
trf_2_3	Bra013		16					UCCGUUGUCGUCCAGC	UAACCGAUGGAUGUCA	Cleava	
70	584	3	.4	1	21	890	910	GGUUA	AUGGA	ge	1
trf_2_3	Bra000		11					UCCGUUGUCGUCCAGC	UAACCGGAGGACGAUA	Cleava	
70	529	3	.2	1	21	984	1004	GGUUA	AUGGA	ge	1
trf_2_3	Bra013		16					UCCGUUGUCGUCCAGC	AUGAUAACCGAUGGAU	Cleava	
71	584	3	.4	1	25	886	910	GGUUAGGAU	GUCAAUGGA	ge	1
trf_2_3	Bra000		11					UCCGUUGUCGUCCAGC	UAACCGGAGGACGAUA	Cleava	
71	529	3	.2	1	21	984	1004	GGUUA	AUGGA	ge	1
trf_2_3	Bra013		16					UCCGUUGUCGUCCAGC	UAACCGAUGGAUGUCA	Cleava	
73	584	3	.4	1	21	890	910	GGUUA	AUGGA	ge	1
trf_2_3	Bra000		11					UCCGUUGUCGUCCAGC	UAACCGGAGGACGAUA	Cleava	
73	529	3	.2	1	21	984	1004	GGUUA	AUGGA	ge	1
trf_2_3	Bra013		16					UCCGUUGUCGUCCAGC	UAUGAUAACCGAUGGA	Cleava	
74	584	3	.4	1	26	885	910	GGUUAGGAUA	UGUCAAUGGA	ge	1
trf_2_3	Bra000		11					UCCGUUGUCGUCCAGC	UAACCGGAGGACGAUA	Cleava	
74	529	3	.2	1	21	984	1004	GGUUA	AUGGA	ge	1
trf_2_3	Bra013		16					UCCGUUGUCGUCCAGC	AACCGAUGGAUGUCAA	Cleava	
77	584	3	.4	1	20	891	910	GGUU	UGGA	ge	1
trf_2_3	Bra000		11					UCCGUUGUCGUCCAGC	AACCGGAGGACGAUAA	Cleava	
77	529	3	.2	1	20	985	1004	GGUU	UGGA	ge	1
trf_2_3	Bra013		16					UCCGUUGUCGUCCAGC	UAACCGAUGGAUGUCA	Cleava	
78	584	3	.4	1	21	890	910	GGUUA	AUGGA	ge	1
trf_2_3	Bra000		11					UCCGUUGUCGUCCAGC	UAACCGGAGGACGAUA	Cleava	
78	529	3	.2	1	21	984	1004	GGUUA	AUGGA	ge	1
trf_2_3	Bra013		16					UCCGUUGUCGUCCAGC	UAUGAUAACCGAUGGA	Cleava	
79	584	3	.4	1	26	885	910	GGUUAGGAUA	UGUCAAUGGA	ge	1
trf_2_3	Bra000		11					UCCGUUGUCGUCCAGC	UAACCGGAGGACGAUA	Cleava	
79	529	3	.2	1	21	984	1004	GGUUA	AUGGA	ge	1
trf_2_3	Bra007		14					GCACCAGUGGUCUAGU	UUACUACUGGACCUUU	Cleava	
80	154	2.5	.1	1	21	510	530	GGUAG	GGUGC	ge	1
trf_2_3	Bra016		20					UCCGUCGUAGUCUAGC	AAGAAGCUAGGCUGCG	Cleava	
81	601	3	.8	1	21	418	438	UGGUU	ACGGA	ge	1

trf_2_3	Bra016		20					UCCGUCGUAGUCUAGC	CUGAAGAAGCUAGGCU	Cleava	
82	601	3	.8	1	24	415	438	UGGUUAGG	GCGACGGA	ge	1
trf_2_3	Bra016		20					UCCGUCGUAGUCUAGC	AAGAAGCUAGGCUGCG	Cleava	
84	601	3	.8	1	21	418	438	UGGUU	ACGGA	ge	1
trf_2_3	Bra016		20					UCCGUCGUAGUCUAGC	CUGAAGAAGCUAGGCU	Cleava	
86	601	3	.8	1	24	415	438	UGGUUAGG	GCGACGGA	ge	1
trf_2_3	Bra016		20					UCCGUCGUAGUCUAGC	CUGAAGAAGCUAGGCU	Cleava	
87	601	3	.8	1	24	415	438	UGGUUAGG	GCGACGGA	ge	1
trf_2_3	Bra024		7.					GGGGAUGUAGCUCAAA	UUCCAACAUUUCGGUU	Cleava	
88	638	3	5	1	24	202	225	UGGUAGAG	ACAUCCCC	ge	1
trf_2_3	Bra025		17					GGGGAUGUAGCUCAAA	ACAAUUUGAGCUUCAU	Cleava	
88	904	3	.2	1	20	344	363	UGGU	CUUC	ge	1
trf_2_3	Bra024		7.					GGGGAUGUAGCUCAAA	UCCAACAUUUCGGUUA	Cleava	
89	638	3	5	1	23	203	225	UGGUAGA	CAUCCCC	ge	1
trf_2_3	Bra025		17					GGGGAUGUAGCUCAAA	ACAAUUUGAGCUUCAU	Cleava	
89	904	3	.2	1	20	344	363	UGGU	CUUC	ge	1
trf_2_3	Bra016		20					UCCGUCGUAGUCUAGC	CUGAAGAAGCUAGGCU	Cleava	
90	601	3	.8	1	24	415	438	UGGUUAGG	GCGACGGA	ge	1
trf_2_3	Bra016		20					UCCGUCGUAGUCUAGC	CUGAAGAAGCUAGGCU	Cleava	
91	601	3	.8	1	24	415	438	UGGUUAGG	GCGACGGA	ge	1
trf_2_3	Bra016		20					UCCGUCGUAGUCUAGC	AAGAAGCUAGGCUGCG	Cleava	
92	601	3	.8	1	21	418	438	UGGUU	ACGGA	ge	1
trf_2_3	Bra016		20					UCCGUCGUAGUCUAGC	AAGAAGCUAGGCUGCG	Cleava	
94	601	3	.8	1	21	418	438	UGGUU	ACGGA	ge	1
trf_2_3	Bra016		20					UCCGUCGUAGUCUAGC	CUGAAGAAGCUAGGCU	Cleava	
95	601	3	.8	1	24	415	438	UGGUUAGG	GCGACGGA	ge	1
trf_2_4	Bra013		22					UCCGUUGUAGUCUAGC	AUCAGCUUGGCUAUAA	Cleava	
06	528	3	.0	1	20	200	219	UGGU	CGGG	ge	1
trf_2_4	Bra029		20					GCUGGAGUAGCUCAGU	GUCAGCUGAGCUACUC	Cleava	
07	975	3	.8	1	20	1245	1264	UGGU	AAGC	ge	1
trf_2_4	Bra003		16					GCUGGAGUAGCUCAGU	CCUAACGGAGCUGCUC	Cleava	
07	013	3	.5	1	20	319	338	UGGU	CAGC	ge	1
trf_2_4	Bra003		23					GCUGGAGUAGCUCAGU	ACCAACCGGGCUACUG	Cleava	
07	602	3	.1	1	20	274	293	UGGU	CAGC	ge	1

trf_2_408	Bra003602	3	23.1	1	21	273	293	GCUGGAGUAGCUCAGUUGGUU	AACCAACCGGGCUACUGCAGC	Cleavage	1
trf_2_408	Bra029975	3	20.8	1	21	1244	1264	GCUGGAGUAGCUCAGUUGGUU	GGUCAGCUGAGCUACUCAAGC	Cleavage	1
trf_2_408	Bra003013	3	16.5	1	20	319	338	GCUGGAGUAGCUCAGUUGGU	CCUAACGGAGCUGCUCCAGC	Cleavage	1
trf_2_409	Bra005511	2.5	12.8	1	22	1113	1134	GUGGCUGUAGUUUAGUGGUGAG	CUCACCGCUGAACUAUGGCUAC	Cleavage	1
trf_2_409	Bra035454	3	17.9	1	24	1241	1264	GUGGCUGUAGUUUAGUGGUGAGAA	UAUUCAUCUCUAGGCUACAGCCAU	Cleavage	1
trf_2_409	Bra009047	3	12.1	1	20	243	262	GUGGCUGUAGUUUAGUGGUG	CAACAUUGAGCUACAGCCAU	Cleavage	1
trf_2_411	Bra007154	2.5	14.1	1	21	510	530	GCACCAGUGGUCUAGUGGUAG	UUACUACUGGACCUUUGGUGC	Cleavage	1
trf_2_420	Bra013528	3	22.0	1	24	196	219	UCCGUUGUAGUCUAGCUGGUCAGG	CAUCAUCAGCUUGGCUAUAACGGG	Cleavage	1
trf_2_433	Bra024638	3	7.5	1	23	203	225	GGGGAUGUAGCUCAAAUGGUAGA	UCCAACAUUUCGGUUACAUCCCC	Cleavage	1
trf_2_433	Bra025904	3	17.2	1	20	344	363	GGGGAUGUAGCUCAAAUGGU	ACAAUUUGAGCUUCAUCUUC	Cleavage	1
trf_2_435	Bra013528	3	22.0	1	24	196	219	UCCGUUGUAGUCUAGCUGGUCAGG	CAUCAUCAGCUUGGCUAUAACGGG	Cleavage	1
trf_2_445	Bra034278	3	21.9	1	21	2430	2450	UUAGGUUCAAAUCCUAUUGGA	UGCGAUAGGAUUUGGACCUGG	Cleavage	1
trf_2_445	Bra019452	2.5	16.5	1	20	970	989	UUAGGUUCAAAUCCUAUUGG	CCAAUUGGAUUUGAUCCUAA	Cleavage	1
trf_2_445	Bra021441	3	14.8	1	21	759	779	UUAGGUUCAAAUCCUAUUGGA	UGCAAUAGGAUUUAGACCUAG	Cleavage	1
trf_2_446	Bra036447	2.5	17.4	1	22	804	825	AGGUUCAAAUCCUAUUGGACGC	GAGUCCAAUGUGGUUUGAGCCU	Cleavage	1
trf_2_446	Bra034278	3	22.4	1	20	2429	2448	AGGUUCAAAUCCUAUUGGAC	CUGCGAUAGGAUUUGGACCU	Cleavage	1
trf_2_1	Bra033370	3	21.6	1	24	4746	4769	GGGGAUGUAGCUCAAAUGGUAGAG	CUGGACCCUUUGGUAACAUCCCC	Translation	1

trf_2_2	Bra033370	3	21.6	1	20	4750	4769	GGGGAUGUAGCUCAAAUGGU	ACCCUUUGGGUAACAUCCCC	Translation	1
trf_2_3	Bra037748	2.5	22.8	1	20	566	585	UCCGUUAUCGUCCAGCGGUU	AACCGCUGUCUGAUAACGGA	Translation	1
trf_2_4	Bra037748	2.5	22.8	1	25	561	585	UCCGUUAUCGUCCAGCGGUUAGGAU	GUUGAAACCGCUGUCUGAUAACGGA	Translation	1
trf_2_5	Bra037748	2.5	22.8	1	20	566	585	UCCGUUAUCGUCCAGCGGUU	AACCGCUGUCUGAUAACGGA	Translation	1
trf_2_7	Bra037748	2.5	22.8	1	25	561	585	UCCGUUAUCGUCCAGCGGUUAGGAU	GUUGAAACCGCUGUCUGAUAACGGA	Translation	1
trf_2_11	Bra037748	2.5	22.8	1	20	566	585	UCCGUUAUCGUCCAGCGGUU	AACCGCUGUCUGAUAACGGA	Translation	1
trf_2_12	Bra037748	2.5	22.8	1	20	566	585	UCCGUUAUCGUCCAGCGGUU	AACCGCUGUCUGAUAACGGA	Translation	1
trf_2_13	Bra037748	2.5	22.8	1	25	561	585	UCCGUUAUCGUCCAGCGGUUAGGAU	GUUGAAACCGCUGUCUGAUAACGGA	Translation	1
trf_2_14	Bra037499	3	14.7	1	22	174	195	UCCGUUGUAGUCUAGCUGGUUA	UGACUGGUUAGGCCACAACGGA	Translation	1
trf_2_15	Bra037499	3	14.7	1	22	174	195	UCCGUUGUAGUCUAGCUGGUUA	UGACUGGUUAGGCCACAACGGA	Translation	1
trf_2_16	Bra033370	3	21.6	1	20	4750	4769	GGGGAUGUAGCUCAAAUGGU	ACCCUUUGGGUAACAUCCCC	Translation	1
trf_2_26	Bra033370	3	21.6	1	20	4750	4769	GGGGAUGUAGCUCAAAUGGU	ACCCUUUGGGUAACAUCCCC	Translation	1
trf_2_37	Bra037499	3	14.7	1	22	174	195	UCCGUUGUAGUCUAGCUGGUCA	UGACUGGUUAGGCCACAACGGA	Translation	1
trf_2_38	Bra036328	3	13.8	1	20	708	727	GUGGCUGUAGUUUAGUGGUG	CAACACUAAAGUGCAGCUAC	Translation	1
trf_2_54	Bra015824	3	24.0	1	23	916	938	AUCAGAGUGGCGCAGCGGAAGCG	CGUUUCCACUGCACCACUCUGAC	Translation	1
trf_2_54	Bra033352	3	18.3	1	20	590	609	AUCAGAGUGGCGCAGCGGAA	UUACGUUGCACCACUUUGAU	Translation	1
trf_2_72	Bra012946	3	16.6	1	20	957	976	GGUCCCAUGGUCUAGCGGUU	AACUGCUACUCCAUGGGACU	Translation	1

trf_2_73	Bra037499	3	14.7	1	20	176	195	UCCGUUGUAGUCUAGCUGGU	ACUGGUUAGGCCACAACGGA	Translation	1
trf_2_77	Bra020153	2.5	15.4	1	22	1953	1974	UCCGUUGUCGUCCAGCGGUUAG	CCAACUGCUGGAAGACAAUGGG	Translation	1
trf_2_77	Bra012143	2.5	18.5	1	20	392	411	UCCGUUGUCGUCCAGCGGUU	AAUCGCUGAACCACAACGGA	Translation	1
trf_2_77	Bra037748	3	22.8	1	25	561	585	UCCGUUGUCGUCCAGCGGUUAGGAU	GUUGAAACCGCUGUCUGAUAACGGA	Translation	1
trf_2_78	Bra020153	2.5	15.4	1	22	1953	1974	UCCGUUGUCGUCCAGCGGUUAG	CCAACUGCUGGAAGACAAUGGG	Translation	1
trf_2_78	Bra012143	2.5	18.5	1	20	392	411	UCCGUUGUCGUCCAGCGGUU	AAUCGCUGAACCACAACGGA	Translation	1
trf_2_78	Bra037748	3	22.8	1	20	566	585	UCCGUUGUCGUCCAGCGGUU	AACCGCUGUCUGAUAACGGA	Translation	1
trf_2_80	Bra020153	2.5	15.4	1	22	1953	1974	UCCGUUGUCGUCCAGCGGUUAG	CCAACUGCUGGAAGACAAUGGG	Translation	1
trf_2_80	Bra012143	2.5	18.5	1	20	392	411	UCCGUUGUCGUCCAGCGGUU	AAUCGCUGAACCACAACGGA	Translation	1
trf_2_80	Bra037748	3	22.8	1	20	566	585	UCCGUUGUCGUCCAGCGGUU	AACCGCUGUCUGAUAACGGA	Translation	1
trf_2_81	Bra020153	2.5	15.4	1	20	1955	1974	UCCGUUGUCGUCCAGCGGUU	AACUGCUGGAAGACAAUGGG	Translation	1
trf_2_81	Bra012143	2.5	18.5	1	20	392	411	UCCGUUGUCGUCCAGCGGUU	AAUCGCUGAACCACAACGGA	Translation	1
trf_2_81	Bra037748	3	22.8	1	20	566	585	UCCGUUGUCGUCCAGCGGUU	AACCGCUGUCUGAUAACGGA	Translation	1
trf_2_83	Bra020153	2.5	15.4	1	22	1953	1974	UCCGUUGUCGUCCAGCGGUUAG	CCAACUGCUGGAAGACAAUGGG	Translation	1
trf_2_83	Bra012143	2.5	18.5	1	20	392	411	UCCGUUGUCGUCCAGCGGUU	AAUCGCUGAACCACAACGGA	Translation	1
trf_2_83	Bra037748	3	22.8	1	25	561	585	UCCGUUGUCGUCCAGCGGUUAGGAU	GUUGAAACCGCUGUCUGAUAACGGA	Translation	1
trf_2_85	Bra020153	2.5	15.4	1	22	1953	1974	UCCGUUGUCGUCCAGCGGUUAG	CCAACUGCUGGAAGACAAUGGG	Translation	1

trf_2_85	Bra012143	2.5	18.5	1	20	392	411	UCCGUUGUCGUCCAGCGGUU	AAUCGCUGAACCACAACGGA	Translation	1
trf_2_85	Bra037748	3	22.8	1	20	566	585	UCCGUUGUCGUCCAGCGGUU	AACCGCUGUCUGAUAACGGA	Translation	1
trf_2_86	Bra020153	2.5	15.4	1	22	1953	1974	UCCGUUGUCGUCCAGCGGUUAG	CCAACUGCUGGAAGACAAUGGG	Translation	1
trf_2_86	Bra012143	2.5	18.5	1	20	392	411	UCCGUUGUCGUCCAGCGGUU	AAUCGCUGAACCACAACGGA	Translation	1
trf_2_86	Bra037748	3	22.8	1	25	561	585	UCCGUUGUCGUCCAGCGGUUAGGAU	GUUGAAACCGCUGUCUGAUAACGGA	Translation	1
trf_2_97	Bra036328	3	13.8	1	20	708	727	GUGGCUGUAGUUUAGUGGUG	CAACACUAAAGUGCAGCUAC	Translation	1
trf_2_97	Bra003191	3	7.6	1	20	27	45	GUGGCUGUAGUUUAGUGGUG	UACCAUUAA-CUACAGCCAC	Translation	1
trf_2_98	Bra036328	3	13.8	1	20	708	727	GUGGCUGUAGUUUAGUGGUG	CAACACUAAAGUGCAGCUAC	Translation	1
trf_2_100	Bra013558	3	11.8	1	21	165	185	GUCUGGGUGGUGUAGUCGGUU	AACCGACUCCACAAUUCAGAC	Translation	1
trf_2_116	Bra033370	3	21.6	1	20	4750	4769	GGGGAUGUAGCUCAAAUGGU	ACCCUUUGGGUAACAUCCCC	Translation	1
trf_2_120	Bra033370	3	21.6	1	20	4750	4769	GGGGAUGUAGCUCAAAUGGU	ACCCUUUGGGUAACAUCCCC	Translation	1
trf_2_121	Bra033370	3	21.6	1	24	4746	4769	GGGGAUGUAGCUCAAAUGGUAGAG	CUGGACCCUUUGGGUAACAUCCCC	Translation	1
trf_2_122	Bra037152	3	12.2	1	22	478	499	GACGGUUUGGCCGAGUGGUCUA	UUGACUACUCGUCCAAACCCUC	Translation	1
trf_2_122	Bra018257	3	7.1	1	25	27	51	GACGGUUUGGCCGAGUGGUCUAAGG	CCCAAGACCACUCCUCGAAACCGUC	Translation	1
trf_2_123	Bra012946	3	16.6	1	20	957	976	GGUCCCAUGGUCUAGCGGUU	AACUGCUACUCCAUGGGACU	Translation	1
trf_2_124	Bra012946	3	16.6	1	20	957	976	GGUCCCAUGGUCUAGCGGUU	AACUGCUACUCCAUGGGACU	Translation	1
trf_2_125	Bra015824	3	24.0	1	23	916	938	AUCAGAGUGGCGCAGCGGAAGCG	CGUUUCCACUGCACCACUCUGAC	Translation	1

trf_2_125	Bra033352	3	18.3	1	20	590	609	AUCAGAGUGGCGCAGCGGAA	UUACGUUGCACCACUUUGAU	Translation	1
trf_2_126	Bra033370	3	21.6	1	24	4746	4769	GGGGAUGUAGCUCAAAUGGUAGAG	CUGGACCCUUUGGGUAACAUCCCC	Translation	1
trf_2_127	Bra033370	3	21.6	1	20	4750	4769	GGGGAUGUAGCUCAAAUGGU	ACCCUUUGGGUAACAUCCCC	Translation	1
trf_2_132	Bra015824	3	24.0	1	23	916	938	AUCAGAGUGGCGCAGCGGAAGCG	CGUUUCCACUGCACCACUCUGAC	Translation	1
trf_2_132	Bra033352	3	18.3	1	20	590	609	AUCAGAGUGGCGCAGCGGAA	UUACGUUGCACCACUUUGAU	Translation	1
trf_2_142	Bra036328	3	13.8	1	20	708	727	GUGGCUGUAGUUUAGUGGUG	CAACACUAAAGUGCAGCUAC	Translation	1
trf_2_151	Bra027297	3	22.9	1	20	178	197	GCCGACUUAGCUCAGUGGUA	UACCACAGGGCGAAGUUGGC	Translation	1
trf_2_152	Bra037499	3	14.7	1	22	174	195	UCCGUUGUAGUCUAGCUGGUCA	UGACUGGUUAGGCCACAACGGA	Translation	1
trf_2_173	Bra037499	3	14.7	1	22	174	195	UCCGUUGUAGUCUAGCUGGUCA	UGACUGGUUAGGCCACAACGGA	Translation	1
trf_2_186	Bra037748	2.5	22.8	1	25	561	585	UCCGUUAUCGUCCAGCGGUUAGGAU	GUUGAAACCGCUGUCUGAUAACGGA	Translation	1
trf_2_187	Bra037748	2.5	22.8	1	25	561	585	UCCGUUAUCGUCCAGCGGUUAGGAU	GUUGAAACCGCUGUCUGAUAACGGA	Translation	1
trf_2_188	Bra037748	2.5	22.8	1	20	566	585	UCCGUUAUCGUCCAGCGGUU	AACCGCUGUCUGAUAACGGA	Translation	1
trf_2_190	Bra037748	2.5	22.8	1	20	566	585	UCCGUUAUCGUCCAGCGGUU	AACCGCUGUCUGAUAACGGA	Translation	1
trf_2_192	Bra037748	2.5	22.8	1	20	566	585	UCCGUUAUCGUCCAGCGGUU	AACCGCUGUCUGAUAACGGA	Translation	1
trf_2_193	Bra037748	2.5	22.8	1	20	566	585	UCCGUUAUCGUCCAGCGGUU	AACCGCUGUCUGAUAACGGA	Translation	1
trf_2_194	Bra037748	2.5	22.8	1	25	561	585	UCCGUUAUCGUCCAGCGGUUAGGAU	GUUGAAACCGCUGUCUGAUAACGGA	Translation	1
trf_2_212	Bra015824	3	24.0	1	23	916	938	AUCAGAGUGGCGCAGCGGAAGCG	CGUUUCCACUGCACCACUCUGAC	Translation	1

trf_2_212	Bra033352	3	18.3	1	20	590	609	AUCAGAGUGGCGCAGCGGAA	UUACGUUGCACCACUUUGAU	Translation	1
trf_2_222	Bra015824	3	24.0	1	23	916	938	AUCAGAGUGGCGCAGCGGAAGCG	CGUUUCCACUGCACCACUCUGAC	Translation	1
trf_2_222	Bra033352	3	18.3	1	20	590	609	AUCAGAGUGGCGCAGCGGAA	UUACGUUGCACCACUUUGAU	Translation	1
trf_2_224	Bra020153	2.5	15.4	1	22	1953	1974	UCCGUUGUCGUCCAGCGGUUAG	CCAACUGCUGGAAGACAAUGGG	Translation	1
trf_2_224	Bra012143	2.5	18.5	1	20	392	411	UCCGUUGUCGUCCAGCGGUU	AAUCGCUGAACCACAACGGA	Translation	1
trf_2_224	Bra037748	3	22.8	1	20	566	585	UCCGUUGUCGUCCAGCGGUU	AACCGCUGUCUGAUAACGGA	Translation	1
trf_2_225	Bra020153	2.5	15.4	1	22	1953	1974	UCCGUUGUCGUCCAGCGGUUAG	CCAACUGCUGGAAGACAAUGGG	Translation	1
trf_2_225	Bra012143	2.5	18.5	1	20	392	411	UCCGUUGUCGUCCAGCGGUU	AAUCGCUGAACCACAACGGA	Translation	1
trf_2_225	Bra037748	3	22.8	1	25	561	585	UCCGUUGUCGUCCAGCGGUUAGGAU	GUUGAAACCGCUGUCUGAUAACGGA	Translation	1
trf_2_226	Bra020153	2.5	15.4	1	22	1953	1974	UCCGUUGUCGUCCAGCGGUUAG	CCAACUGCUGGAAGACAAUGGG	Translation	1
trf_2_226	Bra012143	2.5	18.5	1	20	392	411	UCCGUUGUCGUCCAGCGGUU	AAUCGCUGAACCACAACGGA	Translation	1
trf_2_226	Bra037748	3	22.8	1	25	561	585	UCCGUUGUCGUCCAGCGGUUAGGAU	GUUGAAACCGCUGUCUGAUAACGGA	Translation	1
trf_2_227	Bra020153	2.5	15.4	1	22	1953	1974	UCCGUUGUCGUCCAGCGGUUAG	CCAACUGCUGGAAGACAAUGGG	Translation	1
trf_2_227	Bra012143	2.5	18.5	1	20	392	411	UCCGUUGUCGUCCAGCGGUU	AAUCGCUGAACCACAACGGA	Translation	1
trf_2_227	Bra037748	3	22.8	1	20	566	585	UCCGUUGUCGUCCAGCGGUU	AACCGCUGUCUGAUAACGGA	Translation	1
trf_2_228	Bra020153	2.5	15.4	1	20	1955	1974	UCCGUUGUCGUCCAGCGGUU	AACUGCUGGAAGACAAUGGG	Translation	1
trf_2_228	Bra012143	2.5	18.5	1	20	392	411	UCCGUUGUCGUCCAGCGGUU	AAUCGCUGAACCACAACGGA	Translation	1

trf_2_2	Bra037		22					UCCGUUGUCGUCCAGC	AACCGCUGUCUGAUAA	Transl	
28	748	3	.8	1	20	566	585	GGUU	CGGA	ation	1
trf_2_2	Bra020		15					UCCGUUGUCGUCCAGC	CCAACUGCUGGAAGAC	Transl	
29	153	2.5	.4	1	22	1953	1974	GGUUAG	AAUGGG	ation	1
trf_2_2	Bra012		18					UCCGUUGUCGUCCAGC	AAUCGCUGAACCACAAC	Transl	
29	143	2.5	.5	1	20	392	411	GGUU	GGA	ation	1
trf_2_2	Bra037		22					UCCGUUGUCGUCCAGC	GUUGAAACCGCUGUCU	Transl	
29	748	3	.8	1	25	561	585	GGUUAGGAU	GAUAACGGA	ation	1
trf_2_2	Bra020		15					UCCGUUGUCGUCCAGC	CCAACUGCUGGAAGAC	Transl	
30	153	2.5	.4	1	22	1953	1974	GGUUAG	AAUGGG	ation	1
trf_2_2	Bra012		18					UCCGUUGUCGUCCAGC	AAUCGCUGAACCACAAC	Transl	
30	143	2.5	.5	1	20	392	411	GGUU	GGA	ation	1
trf_2_2	Bra037		22					UCCGUUGUCGUCCAGC	AACCGCUGUCUGAUAA	Transl	
30	748	3	.8	1	20	566	585	GGUU	CGGA	ation	1
trf_2_2	Bra037		14					UCCGUUGUAGUCUAGC	UGACUGGUUAGGCCAC	Transl	
36	499	3	.7	1	22	174	195	UGGUCA	AACGGA	ation	1
trf_2_2	Bra015		24					AUCAGAGUGGCGCAGC	CGUUUCCACUGCACCA	Transl	
43	824	3	.0	1	23	916	938	GGAAGCG	CUCUGAC	ation	1
trf_2_2	Bra033		18					AUCAGAGUGGCGCAGC	UUACGUUGCACCACUU	Transl	
43	352	3	.3	1	20	590	609	GGAA	UGAU	ation	1
trf_2_2	Bra013		11					GUCUGGGUGGUGUAG	AACCGACUCCACAAUUC	Transl	
45	558	3	.8	1	21	165	185	UCGGUU	AGAC	ation	1
trf_2_2	Bra020		15					UCCGUUGUCGUCCAGC	CCAACUGCUGGAAGAC	Transl	
48	153	2.5	.4	1	22	1953	1974	GGUUAG	AAUGGG	ation	1
trf_2_2	Bra012		18					UCCGUUGUCGUCCAGC	AAUCGCUGAACCACAAC	Transl	
48	143	2.5	.5	1	20	392	411	GGUU	GGA	ation	1
trf_2_2	Bra037		22					UCCGUUGUCGUCCAGC	GUUGAAACCGCUGUCU	Transl	
48	748	3	.8	1	25	561	585	GGUUAGGAU	GAUAACGGA	ation	1
trf_2_2	Bra020		15					UCCGUUGUCGUCCAGC	CCAACUGCUGGAAGAC	Transl	
50	153	2.5	.4	1	22	1953	1974	GGUUAG	AAUGGG	ation	1
trf_2_2	Bra012		18					UCCGUUGUCGUCCAGC	AAUCGCUGAACCACAAC	Transl	
50	143	2.5	.5	1	20	392	411	GGUU	GGA	ation	1
trf_2_2	Bra037		22					UCCGUUGUCGUCCAGC	AACCGCUGUCUGAUAA	Transl	
50	748	3	.8	1	20	566	585	GGUU	CGGA	ation	1

trf_2_2	Bra020		15					UCCGUUGUCGUCCAGC	CCAACUGCUGGAAGAC	Transl	
51	153	2.5	.4	1	22	1953	1974	GGUUAG	AAUGGG	ation	1
trf_2_2	Bra012		18					UCCGUUGUCGUCCAGC	AAUCGCUGAACCACAAC	Transl	
51	143	2.5	.5	1	20	392	411	GGUU	GGA	ation	1
trf_2_2	Bra037		22					UCCGUUGUCGUCCAGC	GUUGAAACCGCUGUCU	Transl	
51	748	3	.8	1	25	561	585	GGUUAGGAU	GAUAACGGA	ation	1
trf_2_2	Bra020		15					UCCGUUGUCGUCCAGC	CCAACUGCUGGAAGAC	Transl	
53	153	2.5	.4	1	22	1953	1974	GGUUAG	AAUGGG	ation	1
trf_2_2	Bra012		18					UCCGUUGUCGUCCAGC	AAUCGCUGAACCACAAC	Transl	
53	143	2.5	.5	1	20	392	411	GGUU	GGA	ation	1
trf_2_2	Bra037		22					UCCGUUGUCGUCCAGC	AACCGCUGUCUGAUAA	Transl	
53	748	3	.8	1	20	566	585	GGUU	CGGA	ation	1
trf_2_2	Bra020		15					UCCGUUGUCGUCCAGC	AACUGCUGGAAGACAA	Transl	
54	153	2.5	.4	1	20	1955	1974	GGUU	UGGG	ation	1
trf_2_2	Bra012		18					UCCGUUGUCGUCCAGC	AAUCGCUGAACCACAAC	Transl	
54	143	2.5	.5	1	20	392	411	GGUU	GGA	ation	1
trf_2_2	Bra037		22					UCCGUUGUCGUCCAGC	AACCGCUGUCUGAUAA	Transl	
54	748	3	.8	1	20	566	585	GGUU	CGGA	ation	1
trf_2_2	Bra020		15					UCCGUUGUCGUCCAGC	CCAACUGCUGGAAGAC	Transl	
56	153	2.5	.4	1	22	1953	1974	GGUUAG	AAUGGG	ation	1
trf_2_2	Bra012		18					UCCGUUGUCGUCCAGC	AAUCGCUGAACCACAAC	Transl	
56	143	2.5	.5	1	20	392	411	GGUU	GGA	ation	1
trf_2_2	Bra037		22					UCCGUUGUCGUCCAGC	AACCGCUGUCUGAUAA	Transl	
56	748	3	.8	1	20	566	585	GGUU	CGGA	ation	1
trf_2_2	Bra020		15					UCCGUUGUCGUCCAGC	CCAACUGCUGGAAGAC	Transl	
57	153	2.5	.4	1	22	1953	1974	GGUUAG	AAUGGG	ation	1
trf_2_2	Bra012		18					UCCGUUGUCGUCCAGC	AAUCGCUGAACCACAAC	Transl	
57	143	2.5	.5	1	20	392	411	GGUU	GGA	ation	1
trf_2_2	Bra037		22					UCCGUUGUCGUCCAGC	GUUGAAACCGCUGUCU	Transl	
57	748	3	.8	1	25	561	585	GGUUAGGAU	GAUAACGGA	ation	1
trf_2_2	Bra036		13					GUGGCUGUAGUUUAG	CAACACUAAAGUGCAG	Transl	
68	328	3	.8	1	20	708	727	UGGUG	CUAC	ation	1
trf_2_2	Bra015		24					AUCAGAGUGGCGCAGC	CGUUUCCACUGCACCA	Transl	
74	824	3	.0	1	23	916	938	GGAAGCG	CUCUGAC	ation	1

trf_2_274	Bra033352	3	18.3	1	20	590	609	AUCAGAGUGGCGCAGCGGAA	UUACGUUGCACCACUUUGAU	Translation	1
trf_2_276	Bra015824	3	24.0	1	23	916	938	AUCAGAGUGGCGCAGCGGAAGCG	CGUUUCCACUGCACCACUCUGAC	Translation	1
trf_2_276	Bra033352	3	18.3	1	20	590	609	AUCAGAGUGGCGCAGCGGAA	UUACGUUGCACCACUUUGAU	Translation	1
trf_2_282	Bra015824	3	24.0	1	23	916	938	AUCAGAGUGGCGCAGCGGAAGCG	CGUUUCCACUGCACCACUCUGAC	Translation	1
trf_2_282	Bra033352	3	18.3	1	20	590	609	AUCAGAGUGGCGCAGCGGAA	UUACGUUGCACCACUUUGAU	Translation	1
trf_2_286	Bra038641	3	17.8	1	25	1139	1163	GGGUCCAUAGCUCAGUGGUAGAGCA	UGUUUUAUCACUCAGAUAUGGACUC	Translation	1
trf_2_286	Bra003794	3	22.2	1	21	404	424	GGGUCCAUAGCUCAGUGGUAG	CUUCCACUGAUCUGUGGAUCC	Translation	1
trf_2_288	Bra033370	3	21.6	1	24	4746	4769	GGGGAUGUAGCUCAAAUGGUAGAG	CUGGACCCUUUGGGUAACAUCCCC	Translation	1
trf_2_289	Bra033370	3	21.6	1	20	4750	4769	GGGGAUGUAGCUCAAAUGGU	ACCCUUUGGGUAACAUCCCC	Translation	1
trf_2_299	Bra015824	3	24.0	1	23	916	938	AUCAGAGUGGCGCAGCGGAAGCG	CGUUUCCACUGCACCACUCUGAC	Translation	1
trf_2_299	Bra033352	3	18.3	1	20	590	609	AUCAGAGUGGCGCAGCGGAA	UUACGUUGCACCACUUUGAU	Translation	1
trf_2_300	Bra027297	3	22.9	1	20	178	197	GCCGACUUAGCUCAGUGGUA	UACCACAGGGCGAAGUUGGC	Translation	1
trf_2_304	Bra015824	3	24.0	1	23	916	938	AUCAGAGUGGCGCAGCGGAAGCG	CGUUUCCACUGCACCACUCUGAC	Translation	1
trf_2_304	Bra033352	3	18.3	1	20	590	609	AUCAGAGUGGCGCAGCGGAA	UUACGUUGCACCACUUUGAU	Translation	1
trf_2_305	Bra015824	3	24.0	1	23	916	938	AUCAGAGUGGCGCAGCGGAAGCG	CGUUUCCACUGCACCACUCUGAC	Translation	1
trf_2_305	Bra033352	3	18.3	1	20	590	609	AUCAGAGUGGCGCAGCGGAA	UUACGUUGCACCACUUUGAU	Translation	1
trf_2_308	Bra033370	3	21.6	1	20	4750	4769	GGGGAUGUAGCUCAAAUGGU	ACCCUUUGGGUAACAUCCCC	Translation	1

trf_2_3	Bra033		10					GGGAUUGUAGUUCAA	ACCGAUUGAUCAGCAA	Transl	
26	536	3	.5	1	20	426	445	UCGGU	UUCC	ation	1
trf_2_3	Bra033		10					GGGAUUGUAGUUCAA	ACCGAUUGAUCAGCAA	Transl	
29	536	3	.5	1	20	426	445	UCGGU	UUCC	ation	1
trf_2_3	Bra034		11					GGGAUUGUAGUUCAA	UCCAAAUGAGCAGCAA	Transl	
30	753	3	.6	1	20	443	462	UUGGA	UCCC	ation	1
trf_2_3	Bra003		13					GGGAUUGUAGUUCAA	UCCAGUUAAUCUACAA	Transl	
30	597	3	.1	1	20	1508	1527	UUGGA	UUCC	ation	1
trf_2_3	Bra023		12					GGGAUUGUAGUUCAA	UCCAAGUGAACAUCAA	Transl	
30	172	3	.4	1	20	614	633	UUGGA	UCCC	ation	1
trf_2_3	Bra034		11					GGGAUUGUAGUUCAA	AUCCAAAUGAGCAGCA	Transl	
38	753	3	.6	1	21	442	462	UUGGAU	AUCCC	ation	1
trf_2_3	Bra003		13					GGGAUUGUAGUUCAA	AUCCAGUUAAUCUACA	Transl	
38	597	3	.1	1	21	1507	1527	UUGGAU	AUUCC	ation	1
trf_2_3	Bra023		12					GGGAUUGUAGUUCAA	UCCAAGUGAACAUCAA	Transl	
38	172	3	.4	1	20	614	633	UUGGA	UCCC	ation	1
trf_2_3	Bra033		10					GGGAUUGUAGUUCAA	ACCGAUUGAUCAGCAA	Transl	
39	536	3	.5	1	20	426	445	UCGGU	UUCC	ation	1
trf_2_3	Bra033		10					GGGAUUGUAGUUCAA	ACCGAUUGAUCAGCAA	Transl	
41	536	3	.5	1	20	426	445	UCGGU	UUCC	ation	1
trf_2_3	Bra033		10					GGGAUUGUAGUUCAA	ACCGAUUGAUCAGCAA	Transl	
42	536	3	.5	1	20	426	445	UCGGU	UUCC	ation	1
trf_2_3	Bra034		9.					GGGAUUGUAGUUCAA	CCUAAUUCAACCACAA	Transl	
45	571	3	0	1	20	52	71	UUGGG	UCUC	ation	1
trf_2_3	Bra034		9.					GGGAUUGUAGUUCAA	CCUAAUUCAACCACAA	Transl	
46	571	3	0	1	20	52	71	UUGGG	UCUC	ation	1
trf_2_3	Bra023		16					GCUUCAGUAGCUCGGA	ACCAACCGAGCAACUGA	Transl	
52	806	3	.3	1	20	781	800	UGGC	AGC	ation	1
trf_2_3	Bra023		9.					GGUUCUAUGGUGUAG	AACCAAUACAUGAUAG	Transl	
54	904	3	9	1	20	1163	1182	UGGUU	AAUC	ation	1
trf_2_3	Bra020		15					UCCGUUGUCGUCCAGC	CCAACUGCUGGAAGAC	Transl	
70	153	2.5	.4	1	22	1953	1974	GGUUAG	AAUGGG	ation	1
trf_2_3	Bra012		18					UCCGUUGUCGUCCAGC	AAUCGCUGAACCACAAC	Transl	
70	143	2.5	.5	1	20	392	411	GGUU	GGA	ation	1

trf_2_3	Bra037		22					UCCGUUGUCGUCCAGC	AACCGCUGUCUGAUAA	Transl	
70	748	3	.8	1	20	566	585	GGUU	CGGA	ation	1
trf_2_3	Bra020		15					UCCGUUGUCGUCCAGC	CCAACUGCUGGAAGAC	Transl	
71	153	2.5	.4	1	22	1953	1974	GGUUAG	AAUGGG	ation	1
trf_2_3	Bra012		18					UCCGUUGUCGUCCAGC	AAUCGCUGAACCACAAC	Transl	
71	143	2.5	.5	1	20	392	411	GGUU	GGA	ation	1
trf_2_3	Bra037		22					UCCGUUGUCGUCCAGC	GUUGAAACCGCUGUCU	Transl	
71	748	3	.8	1	25	561	585	GGUUAGGAU	GAUAACGGA	ation	1
trf_2_3	Bra020		15					UCCGUUGUCGUCCAGC	CCAACUGCUGGAAGAC	Transl	
73	153	2.5	.4	1	22	1953	1974	GGUUAG	AAUGGG	ation	1
trf_2_3	Bra012		18					UCCGUUGUCGUCCAGC	AAUCGCUGAACCACAAC	Transl	
73	143	2.5	.5	1	20	392	411	GGUU	GGA	ation	1
trf_2_3	Bra037		22					UCCGUUGUCGUCCAGC	AACCGCUGUCUGAUAA	Transl	
73	748	3	.8	1	20	566	585	GGUU	CGGA	ation	1
trf_2_3	Bra020		15					UCCGUUGUCGUCCAGC	CCAACUGCUGGAAGAC	Transl	
74	153	2.5	.4	1	22	1953	1974	GGUUAG	AAUGGG	ation	1
trf_2_3	Bra012		18					UCCGUUGUCGUCCAGC	AAUCGCUGAACCACAAC	Transl	
74	143	2.5	.5	1	20	392	411	GGUU	GGA	ation	1
trf_2_3	Bra037		22					UCCGUUGUCGUCCAGC	GUUGAAACCGCUGUCU	Transl	
74	748	3	.8	1	25	561	585	GGUUAGGAU	GAUAACGGA	ation	1
trf_2_3	Bra020		15					UCCGUUGUCGUCCAGC	AACUGCUGGAAGACAA	Transl	
77	153	2.5	.4	1	20	1955	1974	GGUU	UGGG	ation	1
trf_2_3	Bra012		18					UCCGUUGUCGUCCAGC	AAUCGCUGAACCACAAC	Transl	
77	143	2.5	.5	1	20	392	411	GGUU	GGA	ation	1
trf_2_3	Bra037		22					UCCGUUGUCGUCCAGC	AACCGCUGUCUGAUAA	Transl	
77	748	3	.8	1	20	566	585	GGUU	CGGA	ation	1
trf_2_3	Bra020		15					UCCGUUGUCGUCCAGC	CCAACUGCUGGAAGAC	Transl	
78	153	2.5	.4	1	22	1953	1974	GGUUAG	AAUGGG	ation	1
trf_2_3	Bra012		18					UCCGUUGUCGUCCAGC	AAUCGCUGAACCACAAC	Transl	
78	143	2.5	.5	1	20	392	411	GGUU	GGA	ation	1
trf_2_3	Bra037		22					UCCGUUGUCGUCCAGC	AACCGCUGUCUGAUAA	Transl	
78	748	3	.8	1	20	566	585	GGUU	CGGA	ation	1
trf_2_3	Bra020		15					UCCGUUGUCGUCCAGC	CCAACUGCUGGAAGAC	Transl	
79	153	2.5	.4	1	22	1953	1974	GGUUAG	AAUGGG	ation	1

trf_2_379	Bra012143	2.5	18.5	1	20	392	411	UCCGUUGUCGUCCAGCGGUU	AAUCGCUGAACCACAACGGA	Translation	1
trf_2_379	Bra037748	3	22.8	1	25	561	585	UCCGUUGUCGUCCAGCGGUUAGGAU	GUUGAAACCGCUGUCUGAUAACGGA	Translation	1
trf_2_388	Bra033370	3	21.6	1	24	4746	4769	GGGGAUGUAGCUCAAAUGGUAGAG	CUGGACCCUUUGGGUAACAUCCCC	Translation	1
trf_2_389	Bra033370	3	21.6	1	20	4750	4769	GGGGAUGUAGCUCAAAUGGU	ACCCUUUGGGUAACAUCCCC	Translation	1
trf_2_406	Bra037499	3	14.7	1	20	176	195	UCCGUUGUAGUCUAGCUGGU	ACUGGUUAGGCCACAACGGA	Translation	1
trf_2_407	Bra032168	2.5	22.3	1	20	150	169	GCUGGAGUAGCUCAGUUGGU	AUCGGCUGAGCCACUCCAGC	Translation	1
trf_2_407	Bra022251	3	23.9	1	20	3475	3494	GCUGGAGUAGCUCAGUUGGU	AUCAAUUGGGAUACUCUAGC	Translation	1
trf_2_407	Bra005116	3	13.5	1	20	234	254	GCUGGAGU-AGCUCAGUUGGU	ACCAACUGAUCUCACUCCAGC	Translation	1
trf_2_408	Bra032168	2.5	22.3	1	20	150	169	GCUGGAGUAGCUCAGUUGGU	AUCGGCUGAGCCACUCCAGC	Translation	1
trf_2_408	Bra005116	3	13.5	1	21	233	254	GCUGGAGU-AGCUCAGUUGGUU	AACCAACUGAUCUCACUCCAGC	Translation	1
trf_2_408	Bra022251	3	23.9	1	20	3475	3494	GCUGGAGUAGCUCAGUUGGU	AUCAAUUGGGAUACUCUAGC	Translation	1
trf_2_409	Bra036328	3	13.8	1	20	708	727	GUGGCUGUAGUUUAGUGGUG	CAACACUAAAGUGCAGCUAC	Translation	1
trf_2_420	Bra037499	3	14.7	1	22	174	195	UCCGUUGUAGUCUAGCUGGUCA	UGACUGGUUAGGCCACAACGGA	Translation	1
trf_2_421	Bra015824	3	24.0	1	23	916	938	AUCAGAGUGGCGCAGCGGAAGCG	CGUUUCCACUGCACCACUCUGAC	Translation	1
trf_2_421	Bra033352	3	18.3	1	20	590	609	AUCAGAGUGGCGCAGCGGAA	UUACGUUGCACCACUUUGAU	Translation	1
trf_2_433	Bra033370	3	21.6	1	20	4750	4769	GGGGAUGUAGCUCAAAUGGU	ACCCUUUGGGUAACAUCCCC	Translation	1
trf_2_434	Bra015824	3	24.0	1	23	916	938	AUCAGAGUGGCGCAGCGGAAGCG	CGUUUCCACUGCACCACUCUGAC	Translation	1

trf_2_434	Bra033352	3	18.3	1	20	590	609	AUCAGAGUGGCGCAGCGGAA	UUACGUUGCACCACUUUGAU	Translation	1
trf_2_435	Bra037499	3	14.7	1	22	174	195	UCCGUUGUAGUCUAGCUGGUCA	UGACUGGUUAGGCCACAACGGA	Translation	1
trf_2_445	Bra009377	3	21.2	1	25	578	602	UUAGGUUCAAAUCCUAUUGGACGCA	UGCAGUCGAUAGGAUAUGAGCCUGA	Translation	1
trf_2_445	Bra008706	3	14.7	1	20	1321	1340	UUAGGUUCAAAUCCUAUUGG	UCGAUGGGGAUUGAACCUAA	Translation	1
trf_2_446	Bra020978	3	12.8	1	20	173	192	AGGUUCAAAUCCUAUUGGAC	GUCCGAGAGGCUUUGGACCU	Translation	1

tRF pollen

ncRNA_Acc.	Target_Acc.	Expectation	UPE	ncRNA_start	ncRNA_end	Target_start	Target_end	ncRNA_aligned_fragment	Target_aligned_fragment	Inhibition	Multiplicity
trf_3_1	Bra020755	3	12.6	1	20	454	473	GGGGAUGUAGCUCAAAUGGU	GCCAUUGGAGCUAUAUUCUC	Cleavage	1
trf_3_1	Bra025904	3	17.2	1	20	344	363	GGGGAUGUAGCUCAAAUGGU	ACAAUUUGAGCUUCAUCUUC	Cleavage	1
trf_3_1	Bra024638	3	7.5	1	20	206	225	GGGGAUGUAGCUCAAAUGGU	AACAUUUCGGUUACAUCCCC	Cleavage	1
trf_3_3	Bra000529	2.5	11.2	1	21	984	1004	UCCGUUAUCGUCCAGCGGUUA	UAACCGGAGGACGAUAAUGGA	Cleavage	1
trf_3_3	Bra032111	3	22.4	1	20	727	746	UCCGUUAUCGUCCAGCGGUU	GACGGCUUGACGAUGACGGA	Cleavage	1
trf_3_4	Bra000529	2.5	11.2	1	21	984	1004	UCCGUUAUCGUCCAGCGGUUA	UAACCGGAGGACGAUAAUGGA	Cleavage	1
trf_3_4	Bra032111	3	22.4	1	20	727	746	UCCGUUAUCGUCCAGCGGUU	GACGGCUUGACGAUGACGGA	Cleavage	1
trf_3_5	Bra000529	2.5	11.2	1	20	985	1004	UCCGUUAUCGUCCAGCGGUU	AACCGGAGGACGAUAAUGGA	Cleavage	1
trf_3_5	Bra032111	3	22.4	1	20	727	746	UCCGUUAUCGUCCAGCGGUU	GACGGCUUGACGAUGACGGA	Cleavage	1
trf_3_6	Bra000529	2.5	11.2	1	21	984	1004	UCCGUUAUCGUCCAGCGGUUA	UAACCGGAGGACGAUAAUGGA	Cleavage	1
trf_3_6	Bra032111	3	22.4	1	20	727	746	UCCGUUAUCGUCCAGCGGUU	GACGGCUUGACGAUGACGGA	Cleavage	1
trf_3_7	Bra000529	2.5	11.2	1	21	984	1004	UCCGUUAUCGUCCAGCGGUUA	UAACCGGAGGACGAUAAUGGA	Cleavage	1
trf_3_7	Bra032111	3	22.4	1	20	727	746	UCCGUUAUCGUCCAGCGGUU	GACGGCUUGACGAUGACGGA	Cleavage	1
trf_3_10	Bra000529	2.5	11.2	1	21	984	1004	UCCGUUAUCGUCCANCGGUUA	UAACCGGAGGACGAUAAUGGA	Cleavage	1
trf_3_11	Bra000529	2.5	11.2	1	21	984	1004	UCCGUUAUCGUCCAGCGGUUA	UAACCGGAGGACGAUAAUGGA	Cleavage	1

trf_3_11	Bra032111	3	22.4	1	20	727	746	UCCGUUAUCGUCCAGCGGUU	GACGGCUUGACGAUGACGGA	Cleavage	1
trf_3_13	Bra000529	2.5	11.2	1	21	984	1004	UCCGUUAUCGUCCAGCGGUUA	UAACCGGAGGACGAUAAUGGA	Cleavage	1
trf_3_13	Bra032111	3	22.4	1	20	727	746	UCCGUUAUCGUCCAGCGGUU	GACGGCUUGACGAUGACGGA	Cleavage	1
trf_3_15	Bra013528	3	22.0	1	24	196	219	UCCGUUGUAGUCUAGCUGGUUAGG	CAUCAUCAGCUUGGCUAUAACGGG	Cleavage	1
trf_3_16	Bra034638	2.5	20.0	1	20	109	128	GCGGACAUAGCUCAGUUGGU	ACCAGCAGAGUUAUGUCCGU	Cleavage	1
trf_3_18	Bra016601	3	20.8	1	24	415	438	UCCGUCGUAGUCUAGCUGGUUAGG	CUGAAGAAGCUAGGCUGCGACGGA	Cleavage	1
trf_3_19	Bra016601	3	20.8	1	24	415	438	UCCGUCGUAGUCUAGCUGGUUAGG	CUGAAGAAGCUAGGCUGCGACGGA	Cleavage	1
trf_3_20	Bra016601	3	20.8	1	24	415	438	UCCGUCGUAGUCUAGCUGGUUAGG	CUGAAGAAGCUAGGCUGCGACGGA	Cleavage	1
trf_3_22	Bra016601	3	20.8	1	21	418	438	UCCGUCGUAGUCUAGCUGGUU	AAGAAGCUAGGCUGCGACGGA	Cleavage	1
trf_3_23	Bra016601	3	20.8	1	21	418	438	UCCGUCGUAGUCUAGCUGGUU	AAGAAGCUAGGCUGCGACGGA	Cleavage	1
trf_3_27	Bra009876	3	14.9	1	20	18	37	GGGGAUGUAGCUCAGAUGGU	AUCAUCUGGGCUUCGUCCUC	Cleavage	1
trf_3_27	Bra016141	3	10.4	1	20	3033	3052	GGGGAUGUAGCUCAGAUGGU	AUCAUCUGAGCUUCGUUCUC	Cleavage	1
trf_3_27	Bra036771	3	20.2	1	20	5378	5397	GGGGAUGUAGCUCAGAUGGU	ACUGGCUGAGCUACAUUCCU	Cleavage	1
trf_3_27	Bra018620	3	8.3	1	20	7	26	GGGGAUGUAGCUCAGAUGGU	GCCACCUCAGCUACAUCCUC	Cleavage	1
trf_3_40	Bra000719	3	15.3	1	20	1680	1699	GGUUCUAUGGUCUAGCGGUU	GACCGCUGGACCAGAGAACU	Cleavage	1
trf_3_40	Bra014489	3	21.1	1	20	168	187	GGUUCUAUGGUCUAGCGGUU	AGCUGCUAGACCUUGGGACC	Cleavage	1
trf_3_41	Bra000719	3	15.3	1	20	1680	1699	GGUUCUAUGGUCUAGCGGUU	GACCGCUGGACCAGAGAACU	Cleavage	1

trf_3_41	Bra014489	3	21.1	1	20	168	187	GGUUCUAUGGUCUAGCGGUU	AGCUGCUAGACCUUGGGACC	Cleavage	1
trf_3_54	Bra007154	2.5	14.1	1	21	510	530	GCACCAGUGGUCUAGUGGUAG	UUACUACUGGACCUUUGGUGC	Cleavage	1
trf_3_81	Bra007154	2.5	14.1	1	21	510	530	GCACCAGUGGUCUAGUGGUAG	UUACUACUGGACCUUUGGUGC	Cleavage	1
trf_3_82	Bra007154	2.5	14.1	1	21	510	530	GCACCAGUGGUCUAGUGGUAG	UUACUACUGGACCUUUGGUGC	Cleavage	1
trf_3_83	Bra013584	3	16.4	1	25	886	910	UCCGUUGUCGUCCAGCGGUUAGGAU	AUGAUAACCGAUGGAUGUCAAUGGA	Cleavage	1
trf_3_83	Bra000529	3	11.2	1	21	984	1004	UCCGUUGUCGUCCAGCGGUUA	UAACCGGAGGACGAUAAUGGA	Cleavage	1
trf_3_84	Bra013584	3	16.4	1	26	885	910	UCCGUUGUCGUCCAGCGGUUAGGAUA	UAUGAUAACCGAUGGAUGUCAAUGGA	Cleavage	1
trf_3_84	Bra000529	3	11.2	1	21	984	1004	UCCGUUGUCGUCCAGCGGUUA	UAACCGGAGGACGAUAAUGGA	Cleavage	1
trf_3_85	Bra013584	3	16.4	1	21	890	910	UCCGUUGUCGUCCAGCGGUUA	UAACCGAUGGAUGUCAAUGGA	Cleavage	1
trf_3_85	Bra000529	3	11.2	1	21	984	1004	UCCGUUGUCGUCCAGCGGUUA	UAACCGGAGGACGAUAAUGGA	Cleavage	1
trf_3_86	Bra013584	3	16.4	1	26	885	910	UCCGUUGUCGUCCAGCGGUUAGGAUA	UAUGAUAACCGAUGGAUGUCAAUGGA	Cleavage	1
trf_3_86	Bra000529	3	11.2	1	21	984	1004	UCCGUUGUCGUCCAGCGGUUA	UAACCGGAGGACGAUAAUGGA	Cleavage	1
trf_3_87	Bra013584	3	16.4	1	21	890	910	UCCGUUGUCGUCCAGCGGUUA	UAACCGAUGGAUGUCAAUGGA	Cleavage	1
trf_3_87	Bra000529	3	11.2	1	21	984	1004	UCCGUUGUCGUCCAGCGGUUA	UAACCGGAGGACGAUAAUGGA	Cleavage	1
trf_3_89	Bra013584	3	16.4	1	20	891	910	UCCGUUGUCGUCCAGCGGUU	AACCGAUGGAUGUCAAUGGA	Cleavage	1
trf_3_89	Bra000529	3	11.2	1	20	985	1004	UCCGUUGUCGUCCAGCGGUU	AACCGGAGGACGAUAAUGGA	Cleavage	1
trf_3_127	Bra020755	3	12.6	1	20	454	473	GGGGAUGUAGCUCAAAUGGU	GCCAUUGGAGCUAUAUUCUC	Cleavage	1

trf_3_1<br>27	Bra025<br>904	3	17<br>.2	1	20	344	363	GGGGAUGUAGCUCAAA<br>UGGU	ACAAUUUGAGCUUCAU<br>CUUC	Cleava<br>ge	1
trf_3_1<br>27	Bra024<br>638	3	7.<br>5	1	20	206	225	GGGGAUGUAGCUCAAA<br>UGGU	AACAUUUCGGUUACAU<br>CCCC	Cleava<br>ge	1
trf_3_1<br>28	Bra014<br>507	3	17<br>.0	1	20	544	563	GGGGAUGUAGCUCAUA<br>UGGU	AUUAUAUUAGCUAUAU<br>CUCC	Cleava<br>ge	1
trf_3_1<br>29	Bra002<br>818	3	10<br>.7	1	22	2389	2410	UGGAGUAUAGCCAAGU<br>GGUAAG	CUUAUCACAUCGCUAU<br>ACUUCA	Cleava<br>ge	1
trf_3_1<br>29	Bra030<br>402	3	17<br>.0	1	22	1519	1540	UGGAGUAUAGCCAAGU<br>GGUAAG	CUUAUCACAUCGCUAU<br>ACUUCA	Cleava<br>ge	2
trf_3_1<br>29	Bra030<br>402	3	17<br>.2	1	22	1993	2014	UGGAGUAUAGCCAAGU<br>GGUAAG	CUUAUCACAUCGCUAU<br>ACUUCA	Cleava<br>ge	2
trf_3_1<br>29	Bra036<br>721	3	15<br>.4	1	20	590	609	UGGAGUAUAGCCAAGU<br>GGUA	AAUCACUUGGUUAUAU<br>UCUA	Cleava<br>ge	1
trf_3_1<br>29	Bra034<br>185	3	18<br>.7	1	23	644	666	UGGAGUAUAGCCAAGU<br>GGUAAGG	CAUUACCAAUGAGCUA<br>UACUCCA	Cleava<br>ge	1
trf_3_1<br>30	Bra020<br>755	3	12<br>.6	1	20	454	473	GGGGAUGUAGCUCAAA<br>UGGU	GCCAUUGGAGCUAUAU<br>UCUC	Cleava<br>ge	1
trf_3_1<br>30	Bra025<br>904	3	17<br>.2	1	20	344	363	GGGGAUGUAGCUCAAA<br>UGGU	ACAAUUUGAGCUUCAU<br>CUUC	Cleava<br>ge	1
trf_3_1<br>30	Bra024<br>638	3	7.<br>5	1	20	206	225	GGGGAUGUAGCUCAAA<br>UGGU	AACAUUUCGGUUACAU<br>CCCC	Cleava<br>ge	1
trf_3_1<br>31	Bra024<br>638	3	7.<br>5	1	24	202	225	GGGGAUGUAGCUCAAA<br>UGGUAGAG	UUCCAACAUUUCGGUU<br>ACAUCCCC	Cleava<br>ge	1
trf_3_1<br>31	Bra025<br>904	3	17<br>.2	1	20	344	363	GGGGAUGUAGCUCAAA<br>UGGU	ACAAUUUGAGCUUCAU<br>CUUC	Cleava<br>ge	1
trf_3_1<br>34	Bra019<br>760	3	12<br>.5	1	23	1328	1350	GACGGUUUGGCCGAGU<br>GGUCUAA	UGAGACUACUUGGUCC<br>AGCCGUC	Cleava<br>ge	1
trf_3_1<br>34	Bra000<br>788	3	16<br>.6	1	20	52	71	GACGGUUUGGCCGAGU<br>GGUC	AACCAUUCCGCCAAAUC<br>GUC	Cleava<br>ge	1
trf_3_1<br>34	Bra003<br>534	3	24<br>.7	1	20	3589	3608	GACGGUUUGGCCGAGU<br>GGUC	GGCGAUUCGGCCAAAU<br>UGUC	Cleava<br>ge	1
trf_3_1<br>34	Bra002<br>609	3	10<br>.2	1	20	1237	1256	GACGGUUUGGCCGAGU<br>GGUC	GACUUUCCGGCCAAAC<br>CGUC	Cleava<br>ge	1

trf_3_136	Bra007154	2.5	14.1	1	21	510	530	GCACCAGUGGUCUAGUGGUAG	UUACUACUGGACCUUUGGUGC	Cleavage	1
trf_3_139	Bra007154	2.5	14.1	1	21	510	530	GCACCAGUGGUCUAGUGGUAG	UUACUACUGGACCUUUGGUGC	Cleavage	1
trf_3_140	Bra000719	3	15.3	1	20	1680	1699	GGUUCUAUGGUCUAGCGGUU	GACCGCUGGACCAGAGAACU	Cleavage	1
trf_3_140	Bra014489	3	21.1	1	20	168	187	GGUUCUAUGGUCUAGCGGUU	AGCUGCUAGACCUUGGGACC	Cleavage	1
trf_3_152	Bra005511	2.5	12.8	1	22	1113	1134	GUGGCUGUAGUUUAGUGGUGAG	CUCACCGCUGAACUAUGGCUAC	Cleavage	1
trf_3_152	Bra035454	3	17.9	1	22	1243	1264	GUGGCUGUAGUUUAGUGGUGAG	UUCAUCUCUAGGCUACAGCCAU	Cleavage	1
trf_3_152	Bra009047	3	12.1	1	20	243	262	GUGGCUGUAGUUUAGUGGUG	CAACAUUGAGCUACAGCCAU	Cleavage	1
trf_3_153	Bra005511	2.5	12.8	1	22	1113	1134	GUGGCUGUAGUUUAGUGGUGAG	CUCACCGCUGAACUAUGGCUAC	Cleavage	1
trf_3_153	Bra035454	3	17.9	1	24	1241	1264	GUGGCUGUAGUUUAGUGGUGAGAA	UAUUCAUCUCUAGGCUACAGCCAU	Cleavage	1
trf_3_153	Bra009047	3	12.1	1	20	243	262	GUGGCUGUAGUUUAGUGGUG	CAACAUUGAGCUACAGCCAU	Cleavage	1
trf_3_159	Bra009876	3	14.9	1	20	18	37	GGGGAUGUAGCUCAGAUGGU	AUCAUCUGGGCUUCGUCCUC	Cleavage	1
trf_3_159	Bra016141	3	10.4	1	20	3033	3052	GGGGAUGUAGCUCAGAUGGU	AUCAUCUGAGCUUCGUUCUC	Cleavage	1
trf_3_159	Bra036771	3	20.2	1	20	5378	5397	GGGGAUGUAGCUCAGAUGGU	ACUGGCUGAGCUACAUUCCU	Cleavage	1
trf_3_159	Bra018620	3	8.3	1	20	7	26	GGGGAUGUAGCUCAGAUGGU	GCCACCUCAGCUACAUCCUC	Cleavage	1
trf_3_161	Bra007154	2.5	14.1	1	21	510	530	GCACCAGUGGUCUAGUGGUAG	UUACUACUGGACCUUUGGUGC	Cleavage	1
trf_3_165	Bra013528	3	22.0	1	24	196	219	UCCGUUGUAGUCUAGCUGGUCAGG	CAUCAUCAGCUUGGCUAUAACGGG	Cleavage	1
trf_3_166	Bra007154	2.5	14.1	1	21	510	530	GCACCAGUGGUCUAGUGGUAG	UUACUACUGGACCUUUGGUGC	Cleavage	1

trf_3_1	Bra009		14					GGGGAUGUAGCUCAGA	UUAUCAUCUGGGCUUC	Cleava	
67	876	3	.9	1	22	16	37	UGGUAG	GUCCUC	ge	1
trf_3_1	Bra016		10					GGGGAUGUAGCUCAGA	UUAUCAUCUGAGCUUC	Cleava	
67	141	3	.4	1	22	3031	3052	UGGUAG	GUUCUC	ge	1
trf_3_1	Bra036		20					GGGGAUGUAGCUCAGA	UCGACUGGCUGAGCUA	Cleava	
67	771	3	.2	1	23	5375	5397	UGGUAGA	CAUUCCU	ge	1
trf_3_1	Bra018		8.					GGGGAUGUAGCUCAGA	GCCACCUCAGCUACAUC	Cleava	
67	620	3	3	1	20	7	26	UGGU	CUC	ge	1
trf_3_1	Bra002		16					AGGGAUAUAACUCAGC	CUGCUGCUGAGUUCUA	Cleava	
95	746	3	.3	1	21	218	238	GGUAG	UCCCA	ge	1
trf_3_1	Bra012		14					AGGGAUAUAACUCAGC	UACCUUUGAGUUAUG	Cleava	
95	734	3	.1	1	20	1709	1728	GGUA	UUUCU	ge	1
trf_3_2	Bra000		15					GGUUCUAUGGUCUAGC	GACCGCUGGACCAGAG	Cleava	
09	719	3	.3	1	20	1680	1699	GGUU	AACU	ge	1
trf_3_2	Bra014		21					GGUUCUAUGGUCUAGC	AGCUGCUAGACCUUGG	Cleava	
09	489	3	.1	1	20	168	187	GGUU	GACC	ge	1
trf_3_2	Bra000		11					UCCGUUAUCGUCCAGC	UAACCGGAGGACGAUA	Cleava	
11	529	2.5	.2	1	21	984	1004	GGUUA	AUGGA	ge	1
trf_3_2	Bra032		22					UCCGUUAUCGUCCAGC	GACGGCUUGACGAUGA	Cleava	
11	111	3	.4	1	20	727	746	GGUU	CGGA	ge	1
trf_3_2	Bra000		11					UCCGUUAUCGUCCAGC	UAACCGGAGGACGAUA	Cleava	
12	529	2.5	.2	1	21	984	1004	GGUUA	AUGGA	ge	1
trf_3_2	Bra032		22					UCCGUUAUCGUCCAGC	GACGGCUUGACGAUGA	Cleava	
12	111	3	.4	1	20	727	746	GGUU	CGGA	ge	1
trf_3_2	Bra000		11					UCCGUUAUCGUCCAGC	UAACCGGAGGACGAUA	Cleava	
14	529	2.5	.2	1	21	984	1004	GGUUA	AUGGA	ge	1
trf_3_2	Bra032		22					UCCGUUAUCGUCCAGC	GACGGCUUGACGAUGA	Cleava	
14	111	3	.4	1	20	727	746	GGUU	CGGA	ge	1
trf_3_2	Bra000		11					UCCGUUAUCGUCCAGC	UAACCGGAGGACGAUA	Cleava	
15	529	2.5	.2	1	21	984	1004	GGUUA	AUGGA	ge	1
trf_3_2	Bra032		22					UCCGUUAUCGUCCAGC	GACGGCUUGACGAUGA	Cleava	
15	111	3	.4	1	20	727	746	GGUU	CGGA	ge	1
trf_3_2	Bra000		11					UCCGUUAUCGUCCAGC	UAACCGGAGGACGAUA	Cleava	
16	529	2.5	.2	1	21	984	1004	GGUUA	AUGGA	ge	1

trf_3_216	Bra032111	3	22.4	1	20	727	746	UCCGUUAUCGUCCAGCGGUU	GACGGCUUGACGAUGACGGA	Cleavage	1
trf_3_217	Bra000529	2.5	11.2	1	21	984	1004	UCCGUUAUCGUCCANCGGUUA	UAACCGGAGGACGAUAAUGGA	Cleavage	1
trf_3_218	Bra000529	2.5	11.2	1	21	984	1004	UCCGUUAUCGUCCAGCGGUUA	UAACCGGAGGACGAUAAUGGA	Cleavage	1
trf_3_218	Bra032111	3	22.4	1	20	727	746	UCCGUUAUCGUCCAGCGGUU	GACGGCUUGACGAUGACGGA	Cleavage	1
trf_3_220	Bra000529	2.5	11.2	1	20	985	1004	UCCGUUAUCGUCCAGCGGUU	AACCGGAGGACGAUAAUGGA	Cleavage	1
trf_3_220	Bra032111	3	22.4	1	20	727	746	UCCGUUAUCGUCCAGCGGUU	GACGGCUUGACGAUGACGGA	Cleavage	1
trf_3_221	Bra000529	2.5	11.2	1	21	984	1004	UCCGUUAUCGUCCAGCGGUUA	UAACCGGAGGACGAUAAUGGA	Cleavage	1
trf_3_221	Bra032111	3	22.4	1	20	727	746	UCCGUUAUCGUCCAGCGGUU	GACGGCUUGACGAUGACGGA	Cleavage	1
trf_3_238	Bra009876	3	14.9	1	20	18	37	GGGGAUGUAGCUCAGAUGGU	AUCAUCUGGGCUUCGUCCUC	Cleavage	1
trf_3_238	Bra016141	3	10.4	1	20	3033	3052	GGGGAUGUAGCUCAGAUGGU	AUCAUCUGAGCUUCGUUCUC	Cleavage	1
trf_3_238	Bra036771	3	20.2	1	20	5378	5397	GGGGAUGUAGCUCAGAUGGU	ACUGGCUGAGCUACAUUCCU	Cleavage	1
trf_3_238	Bra018620	3	8.3	1	20	7	26	GGGGAUGUAGCUCAGAUGGU	GCCACCUCAGCUACAUCCUC	Cleavage	1
trf_3_240	Bra007154	2.5	14.1	1	21	510	530	GCACCAGUGGUCUAGUGGUAG	UUACUACUGGACCUUUGGUGC	Cleavage	1
trf_3_258	Bra013584	3	16.4	1	26	885	910	UCCGUUGUCGUCCAGCGGUUAGGAUA	UAUGAUAACCGAUGGAUGUCAAUGGA	Cleavage	1
trf_3_258	Bra000529	3	11.2	1	21	984	1004	UCCGUUGUCGUCCAGCGGUUA	UAACCGGAGGACGAUAAUGGA	Cleavage	1
trf_3_259	Bra013584	3	16.4	1	20	891	910	UCCGUUGUCGUCCAGCGGUU	AACCGAUGGAUGUCAAUGGA	Cleavage	1
trf_3_259	Bra000529	3	11.2	1	20	985	1004	UCCGUUGUCGUCCAGCGGUU	AACCGGAGGACGAUAAUGGA	Cleavage	1

trf_3_260	Bra013584	3	16.4	1	21	890	910	UCCGUUGUCGUCCAGCGGUUA	UAACCGAUGGAUGUCAAUGGA	Cleavage	1
trf_3_260	Bra000529	3	11.2	1	21	984	1004	UCCGUUGUCGUCCAGCGGUUA	UAACCGGAGGACGAUAAUGGA	Cleavage	1
trf_3_261	Bra013584	3	16.4	1	25	886	910	UCCGUUGUCGUCCAGCGGUUAGGAU	AUGAUAACCGAUGGAUGUCAAUGGA	Cleavage	1
trf_3_261	Bra000529	3	11.2	1	21	984	1004	UCCGUUGUCGUCCAGCGGUUA	UAACCGGAGGACGAUAAUGGA	Cleavage	1
trf_3_263	Bra013584	3	16.4	1	26	885	910	UCCGUUGUCGUCCAGCGGUUAGGAUA	UAUGAUAACCGAUGGAUGUCAAUGGA	Cleavage	1
trf_3_263	Bra000529	3	11.2	1	21	984	1004	UCCGUUGUCGUCCAGCGGUUA	UAACCGGAGGACGAUAAUGGA	Cleavage	1
trf_3_264	Bra013584	3	16.4	1	21	890	910	UCCGUUGUCGUCCAGCGGUUA	UAACCGAUGGAUGUCAAUGGA	Cleavage	1
trf_3_264	Bra000529	3	11.2	1	21	984	1004	UCCGUUGUCGUCCAGCGGUUA	UAACCGGAGGACGAUAAUGGA	Cleavage	1
trf_3_267	Bra013584	3	16.4	1	21	890	910	UCCGUUGUCGUCCAGCGGUUA	UAACCGAUGGAUGUCAAUGGA	Cleavage	1
trf_3_267	Bra000529	3	11.2	1	21	984	1004	UCCGUUGUCGUCCAGCGGUUA	UAACCGGAGGACGAUAAUGGA	Cleavage	1
trf_3_268	Bra007154	2.5	14.1	1	21	510	530	GCACCAGUGGUCUAGUGGUAG	UUACUACUGGACCUUUGGUGC	Cleavage	1
trf_3_275	Bra007154	2.5	14.1	1	21	510	530	GCACCAGUGGUCUAGUGGUAG	UUACUACUGGACCUUUGGUGC	Cleavage	1
trf_3_279	Bra007154	2.5	14.1	1	21	510	530	GCACCAGUGGUCUAGUGGUAG	UUACUACUGGACCUUUGGUGC	Cleavage	1
trf_3_280	Bra013528	3	22.0	1	20	200	219	UCCGUUGUAGUCUAGCUGGU	AUCAGCUUGGCUAUAACGGG	Cleavage	1
trf_3_281	Bra007154	2.5	14.1	1	21	510	530	GCACCAGUGGUCUAGUGGUAG	UUACUACUGGACCUUUGGUGC	Cleavage	1
trf_3_288	Bra013584	3	16.4	1	20	891	910	UCCGUUGUCGUCCAGCGGUU	AACCGAUGGAUGUCAAUGGA	Cleavage	1
trf_3_288	Bra000529	3	11.2	1	20	985	1004	UCCGUUGUCGUCCAGCGGUU	AACCGGAGGACGAUAAUGGA	Cleavage	1

trf_3_2	Bra013		16					UCCGUUGUCGUCCAGC	AUGAUAACCGAUGGAU	Cleava	
89	584	3	.4	1	25	886	910	GGUUAGGAU	GUCAAUGGA	ge	1
trf_3_2	Bra000		11					UCCGUUGUCGUCCAGC	UAACCGGAGGACGAUA	Cleava	
89	529	3	.2	1	21	984	1004	GGUUA	AUGGA	ge	1
trf_3_2	Bra013		16					UCCGUUGUCGUCCAGC	UAACCGAUGGAUGUCA	Cleava	
90	584	3	.4	1	21	890	910	GGUUA	AUGGA	ge	1
trf_3_2	Bra000		11					UCCGUUGUCGUCCAGC	UAACCGGAGGACGAUA	Cleava	
90	529	3	.2	1	21	984	1004	GGUUA	AUGGA	ge	1
trf_3_2	Bra013		16					UCCGUUGUCGUCCAGC	UAUGAUAACCGAUGGA	Cleava	
91	584	3	.4	1	26	885	910	GGUUAGGAUA	UGUCAAUGGA	ge	1
trf_3_2	Bra000		11					UCCGUUGUCGUCCAGC	UAACCGGAGGACGAUA	Cleava	
91	529	3	.2	1	21	984	1004	GGUUA	AUGGA	ge	1
trf_3_2	Bra013		16					UCCGUUGUCGUCCAGC	UAACCGAUGGAUGUCA	Cleava	
92	584	3	.4	1	21	890	910	GGUUA	AUGGA	ge	1
trf_3_2	Bra000		11					UCCGUUGUCGUCCAGC	UAACCGGAGGACGAUA	Cleava	
92	529	3	.2	1	21	984	1004	GGUUA	AUGGA	ge	1
trf_3_2	Bra013		16					UCCGUUGUCGUCCAGC	UAACCGAUGGAUGUCA	Cleava	
94	584	3	.4	1	21	890	910	GGUUA	AUGGA	ge	1
trf_3_2	Bra000		11					UCCGUUGUCGUCCAGC	UAACCGGAGGACGAUA	Cleava	
94	529	3	.2	1	21	984	1004	GGUUA	AUGGA	ge	1
trf_3_2	Bra014		17					GGGGAUGUAGCUCAUA	AUUAUAUUAGCUAUAU	Cleava	
97	507	3	.0	1	20	544	563	UGGU	CUCC	ge	1
trf_3_2	Bra016		20					UCCGUCGUAGUCUAGC	CUGAAGAAGCUAGGCU	Cleava	
98	601	3	.8	1	24	415	438	UGGUUAGG	GCGACGGA	ge	1
trf_3_3	Bra016		20					UCCGUCGUAGUCUAGC	CUGAAGAAGCUAGGCU	Cleava	
00	601	3	.8	1	24	415	438	UGGUUAGG	GCGACGGA	ge	1
trf_3_3	Bra016		20					UCCGUCGUAGUCUAGC	CUGAAGAAGCUAGGCU	Cleava	
01	601	3	.8	1	24	415	438	UGGUUAGG	GCGACGGA	ge	1
trf_3_3	Bra016		20					UCCGUCGUAGUCUAGC	AAGAAGCUAGGCUGCG	Cleava	
02	601	3	.8	1	21	418	438	UGGUU	ACGGA	ge	1
trf_3_3	Bra005		12					GUGGCUGUAGUUUAG	CUCACCGCUGAACUAU	Cleava	
03	511	2.5	.8	1	22	1113	1134	UGGUGAG	GGCUAC	ge	1
trf_3_3	Bra035		17					GUGGCUGUAGUUUAG	UUCAUCUCUAGGCUAC	Cleava	
03	454	3	.9	1	22	1243	1264	UGGUGAG	AGCCAU	ge	1

trf_3_3	Bra009		12					GUGGCUGUAGUUUAG	CAACAUUGAGCUACAG	Cleava	
03	047	3	.1	1	20	243	262	UGGUG	CCAU	ge	1
trf_3_3	Bra013		22					UCCGUUGUAGUCUAGC	CAUCAUCAGCUUGGCU	Cleava	
08	528	3	.0	1	24	196	219	UGGUCAGG	AUAACGGG	ge	1
trf_3_3	Bra009		14					GGGGAUGUAGCUCAGA	AUCAUCUGGGCUUCGU	Cleava	
10	876	3	.9	1	20	18	37	UGGU	CCUC	ge	1
trf_3_3	Bra016		10					GGGGAUGUAGCUCAGA	AUCAUCUGAGCUUCGU	Cleava	
10	141	3	.4	1	20	3033	3052	UGGU	UCUC	ge	1
trf_3_3	Bra036		20					GGGGAUGUAGCUCAGA	ACUGGCUGAGCUACAU	Cleava	
10	771	3	.2	1	20	5378	5397	UGGU	UCCU	ge	1
trf_3_3	Bra018		8.					GGGGAUGUAGCUCAGA	GCCACCUCAGCUACAUC	Cleava	
10	620	3	3	1	20	7	26	UGGU	CUC	ge	1
trf_3_3	Bra009		14					GGGGAUGUAGCUCAGA	UUAUCAUCUGGGCUUC	Cleava	
13	876	3	.9	1	22	16	37	UGGUAG	GUCCUC	ge	1
trf_3_3	Bra016		10					GGGGAUGUAGCUCAGA	UUAUCAUCUGAGCUUC	Cleava	
13	141	3	.4	1	22	3031	3052	UGGUAG	GUUCUC	ge	1
trf_3_3	Bra036		20					GGGGAUGUAGCUCAGA	UCGACUGGCUGAGCUA	Cleava	
13	771	3	.2	1	23	5375	5397	UGGUAGA	CAUUCCU	ge	1
trf_3_3	Bra018		8.					GGGGAUGUAGCUCAGA	GCCACCUCAGCUACAUC	Cleava	
13	620	3	3	1	20	7	26	UGGU	CUC	ge	1
trf_3_3	Bra007		20					GAUAGUUUGGCCGAGU	GGACCAUUCUGUCAAG	Cleava	
14	942	2.5	.9	1	21	1425	1445	GGUCU	CUAUC	ge	1
trf_3_3	Bra028		14					GAUAGUUUGGCCGAGU	GGACCACGCGGUUAGA	Cleava	
14	573	3	.6	1	21	270	290	GGUCU	CUAUU	ge	1
trf_3_3	Bra003		24					GAUAGUUUGGCCGAGU	GGCGAUUCGGCCAAAU	Cleava	
14	534	3	.7	1	20	3589	3608	GGUC	UGUC	ge	1
trf_3_3	Bra015		16					GAUAGUUUGGCCGAGU	GAUAACUUUGCCAAAC	Cleava	
14	687	3	.3	1	20	880	899	GGUC	UAUC	ge	1
trf_3_3	Bra007		20					GAUAGUUUGGCCGAGU	GGACCAUUCUGUCAAG	Cleava	
15	942	2.5	.9	1	21	1425	1445	GGUCU	CUAUC	ge	1
trf_3_3	Bra028		14					GAUAGUUUGGCCGAGU	GGACCACGCGGUUAGA	Cleava	
15	573	3	.6	1	21	270	290	GGUCU	CUAUU	ge	1
trf_3_3	Bra003		24					GAUAGUUUGGCCGAGU	GGCGAUUCGGCCAAAU	Cleava	
15	534	3	.7	1	20	3589	3608	GGUC	UGUC	ge	1

trf_3_3	Bra015		16					GAUAGUUUGGCCGAGU	GAUAACUUUGCCAAAC	Cleava	
15	687	3	.3	1	20	880	899	GGUC	UAUC	ge	1
trf_3_3	Bra009		14					GGGGAUGUAGCUCAGA	AUCAUCUGGGCUUCGU	Cleava	
16	876	3	.9	1	20	18	37	UGGU	CCUC	ge	1
trf_3_3	Bra016		10					GGGGAUGUAGCUCAGA	AUCAUCUGAGCUUCGU	Cleava	
16	141	3	.4	1	20	3033	3052	UGGU	UCUC	ge	1
trf_3_3	Bra036		20					GGGGAUGUAGCUCAGA	ACUGGCUGAGCUACAU	Cleava	
16	771	3	.2	1	20	5378	5397	UGGU	UCCU	ge	1
trf_3_3	Bra018		8.					GGGGAUGUAGCUCAGA	GCCACCUCAGCUACAUC	Cleava	
16	620	3	3	1	20	7	26	UGGU	CUC	ge	1
trf_3_3	Bra007		14					GCACCAGUGGUCUAGU	UUACUACUGGACCUUU	Cleava	
20	154	2.5	.1	1	21	510	530	GGUAG	GGUGC	ge	1
trf_3_3	Bra023		16					GUCUGGUUGGUGUAG	UCCGACAGUACCAAUC	Cleava	
22	121	2.5	.9	1	20	661	680	UCGGA	AGAC	ge	1
trf_3_3	Bra039		13					GUCUGGUUGGUGUAG	UCCGACCGUACCGAUC	Cleava	
22	594	3	.8	1	20	280	299	UCGGA	AGAC	ge	1
trf_3_3	Bra023		15					GUCUGGUUGGUGUAG	UCCGACAACACCAACCA	Cleava	
22	936	3	.5	1	20	505	524	UCGGA	CAU	ge	1
trf_3_3	Bra012		12					GUCUGGUUGGUGUAG	UCCGACAAUACCAACCA	Cleava	
22	539	3	.0	1	20	3001	3020	UCGGA	GUC	ge	1
trf_3_3	Bra025		18					GUCUGGUUGGUGUAG	UCCGCCUCCACCAGCUA	Cleava	
22	413	3	.8	1	20	1299	1318	UCGGA	GAC	ge	1
trf_3_3	Bra007		14					GCACCAGUGGUCUAGU	UUACUACUGGACCUUU	Cleava	
40	154	2.5	.1	1	21	510	530	GGUAG	GGUGC	ge	1
trf_3_3	Bra009		14					GGGGAUGUAGCUCAGA	AUCAUCUGGGCUUCGU	Cleava	
46	876	3	.9	1	20	18	37	UGGU	CCUC	ge	1
trf_3_3	Bra016		10					GGGGAUGUAGCUCAGA	AUCAUCUGAGCUUCGU	Cleava	
46	141	3	.4	1	20	3033	3052	UGGU	UCUC	ge	1
trf_3_3	Bra036		20					GGGGAUGUAGCUCAGA	ACUGGCUGAGCUACAU	Cleava	
46	771	3	.2	1	20	5378	5397	UGGU	UCCU	ge	1
trf_3_3	Bra018		8.					GGGGAUGUAGCUCAGA	GCCACCUCAGCUACAUC	Cleava	
46	620	3	3	1	20	7	26	UGGU	CUC	ge	1
trf_3_3	Bra020		12					GGGGAUGUAGCUCAAA	GCCAUUGGAGCUAUAU	Cleava	
47	755	3	.6	1	20	454	473	UGGU	UCUC	ge	1

trf_3_3	Bra025		17					GGGGAUGUAGCUCAAA	ACAAUUUGAGCUUCAU	Cleava	
47	904	3	.2	1	20	344	363	UGGU	CUUC	ge	1
trf_3_3	Bra024		7.					GGGGAUGUAGCUCAAA	AACAUUUCGGUUACAU	Cleava	
47	638	3	5	1	20	206	225	UGGU	CCCC	ge	1
trf_3_3	Bra009		14					GGGGAUGUAGCUCAGA	AUCAUCUGGGCUUCGU	Cleava	
48	876	3	.9	1	20	18	37	UGGU	CCUC	ge	1
trf_3_3	Bra016		10					GGGGAUGUAGCUCAGA	AUCAUCUGAGCUUCGU	Cleava	
48	141	3	.4	1	20	3033	3052	UGGU	UCUC	ge	1
trf_3_3	Bra036		20					GGGGAUGUAGCUCAGA	ACUGGCUGAGCUACAU	Cleava	
48	771	3	.2	1	20	5378	5397	UGGU	UCCU	ge	1
trf_3_3	Bra018		8.					GGGGAUGUAGCUCAGA	GCCACCUCAGCUACAUC	Cleava	
48	620	3	3	1	20	7	26	UGGU	CUC	ge	1
trf_3_3	Bra000		15					GGUUCUAUGGUCUAGC	GACCGCUGGACCAGAG	Cleava	
49	719	3	.3	1	20	1680	1699	GGUU	AACU	ge	1
trf_3_3	Bra014		21					GGUUCUAUGGUCUAGC	AGCUGCUAGACCUUGG	Cleava	
49	489	3	.1	1	20	168	187	GGUU	GACC	ge	1
trf_3_3	Bra000		15					GGUUCUAUGGUCUAGC	GACCGCUGGACCAGAG	Cleava	
50	719	3	.3	1	20	1680	1699	GGUU	AACU	ge	1
trf_3_3	Bra014		21					GGUUCUAUGGUCUAGC	AGCUGCUAGACCUUGG	Cleava	
50	489	3	.1	1	20	168	187	GGUU	GACC	ge	1
trf_3_3	Bra036		20					GGGGAUGUAGCUCAGA	CUCGACUGGCUGAGCU	Cleava	
51	771	3	.2	1	24	5374	5397	UGGUAGAG	ACAUUCCU	ge	1
trf_3_3	Bra009		14					GGGGAUGUAGCUCAGA	CGUUAUCAUCUGGGCU	Cleava	
51	876	3	.9	1	24	14	37	UGGUAGAG	UCGUCCUC	ge	1
trf_3_3	Bra016		10					GGGGAUGUAGCUCAGA	CAUUAUCAUCUGAGCU	Cleava	
51	141	3	.4	1	24	3029	3052	UGGUAGAG	UCGUUCUC	ge	1
trf_3_3	Bra018		8.					GGGGAUGUAGCUCAGA	GCCACCUCAGCUACAUC	Cleava	
51	620	3	3	1	20	7	26	UGGU	CUC	ge	1
trf_3_3	Bra007		14					GCACCAGUGGUCUAGU	UUACUACUGGACCUUU	Cleava	
52	154	2.5	.1	1	21	510	530	GGUAG	GGUGC	ge	1
trf_3_3	Bra003		16					GGGAUUGUAGUUCAA	ACCAGUUGAAUUACAA	Cleava	
53	753	2.5	.2	1	20	284	303	UCGGU	UCCU	ge	1
trf_3_3	Bra003		16					GGGAUUGUAGUUCAA	AACCAGUUGAAUUACA	Cleava	
54	753	2.5	.2	1	21	283	303	UUGGAU	AUCCU	ge	1

trf_3_355	Bra003753	1.5	16.2	1	20	284	303	GGGAUUGUAGUUCAAUUGGU	ACCAGUUGAAUUACAAUCCU	Cleavage	1
trf_3_355	Bra013336	3	19.5	1	20	267	286	GGGAUUGUAGUUCAAUUGGU	ACUAAUUGAACUAAGAUCUC	Cleavage	1
trf_3_355	Bra021314	3	23.2	1	20	234	253	GGGAUUGUAGUUCAAUUGGU	ACCAACUGAACUGCAAUCUG	Cleavage	1
trf_3_357	Bra003753	2.5	16.2	1	20	284	303	GGGAUUGUAGUUCAAUCGGU	ACCAGUUGAAUUACAAUCCU	Cleavage	1
trf_3_358	Bra003753	2.5	16.2	1	20	284	303	GGGAUUGUAGUUCAAUUGGA	ACCAGUUGAAUUACAAUCCU	Cleavage	1
trf_3_359	Bra003753	1.5	16.2	1	20	284	303	GGGAUUGUAGUUCAAUUGGU	ACCAGUUGAAUUACAAUCCU	Cleavage	1
trf_3_359	Bra013336	3	19.5	1	20	267	286	GGGAUUGUAGUUCAAUUGGU	ACUAAUUGAACUAAGAUCUC	Cleavage	1
trf_3_359	Bra021314	3	23.2	1	20	234	253	GGGAUUGUAGUUCAAUUGGU	ACCAACUGAACUGCAAUCUG	Cleavage	1
trf_3_362	Bra003753	2.5	16.2	1	20	284	303	GGGAUUGUAGUUCAAUCGGU	ACCAGUUGAAUUACAAUCCU	Cleavage	1
trf_3_363	Bra003753	1.5	16.2	1	20	284	303	GGGAUUGUAGUUCAAUUGGU	ACCAGUUGAAUUACAAUCCU	Cleavage	1
trf_3_363	Bra013336	3	19.5	1	20	267	286	GGGAUUGUAGUUCAAUUGGU	ACUAAUUGAACUAAGAUCUC	Cleavage	1
trf_3_363	Bra021314	3	23.2	1	20	234	253	GGGAUUGUAGUUCAAUUGGU	ACCAACUGAACUGCAAUCUG	Cleavage	1
trf_3_364	Bra003753	2.5	16.2	1	20	284	303	GGGAUUGUAGUUCAAUCGGU	ACCAGUUGAAUUACAAUCCU	Cleavage	1
trf_3_365	Bra003753	1.5	16.2	1	20	284	303	GGGAUUGUAGUUCAAUUGGU	ACCAGUUGAAUUACAAUCCU	Cleavage	1
trf_3_365	Bra013336	3	19.5	1	20	267	286	GGGAUUGUAGUUCAAUUGGU	ACUAAUUGAACUAAGAUCUC	Cleavage	1
trf_3_365	Bra021314	3	23.2	1	20	234	253	GGGAUUGUAGUUCAAUUGGU	ACCAACUGAACUGCAAUCUG	Cleavage	1
trf_3_366	Bra003753	1.5	16.2	1	20	284	303	GGGAUUGUAGUUCAAUUGGU	ACCAGUUGAAUUACAAUCCU	Cleavage	1

trf_3_3	Bra013		19					GGGAUUGUAGUUCAA	ACUAAUUGAACUAAGA	Cleava	
66	336	3	.5	1	20	267	286	UUGGU	UCUC	ge	1
trf_3_3	Bra021		23					GGGAUUGUAGUUCAA	ACCAACUGAACUGCAA	Cleava	
66	314	3	.2	1	20	234	253	UUGGU	UCUG	ge	1
trf_3_3	Bra003		16					GGGAUUGUAGUUCAA	GAACCAGUUGAAUUAC	Cleava	
67	753	2.5	.2	1	22	282	303	UCGGUCC	AAUCCU	ge	1
trf_3_3	Bra003		16					GGGAUUGUAGUUCAA	ACCAGUUGAAUUACAA	Cleava	
68	753	2.5	.2	1	20	284	303	UCGGU	UCCU	ge	1
trf_3_3	Bra003		16					GGGAUUGUAGUUCAA	ACCAGUUGAAUUACAA	Cleava	
69	753	2.5	.2	1	20	284	303	UCGGU	UCCU	ge	1
trf_3_3	Bra003		16					GGGAUUGUAGUUCAA	ACCAGUUGAAUUACAA	Cleava	
73	753	2.5	.2	1	20	284	303	UCGGU	UCCU	ge	1
trf_3_3	Bra010		20					GCUUCAGUAGCUCGGA	GUCAUCCGAGCUGAUG	Cleava	
78	153	2.5	.4	1	20	850	869	UGGC	AAGC	ge	1
trf_3_3	Bra035		17					GCUUCAGUAGCUCGGA	GCUGUCUGAGUUACUG	Cleava	
78	649	3	.6	1	20	423	442	UGGC	AAGA	ge	1
trf_3_3	Bra016		16					GCUUCAGUAGCUCGGA	GCCAUCCAGGUUGCUG	Cleava	
78	307	2.5	.1	1	20	1153	1172	UGGC	AAGC	ge	1
trf_3_3	Bra029		21					GCUUCAGUAGCUCGGA	ACUAACCGGGCUACUG	Cleava	
78	313	3	.3	1	20	262	281	UGGC	AAGC	ge	1
trf_3_3	Bra018		12					GCUUCAGUAGCUCGGA	GGCAUCUGAGCUACUA	Cleava	
78	588	3	.1	1	20	897	916	UGGC	AAGC	ge	1
trf_3_3	Bra032		23					GCUUCAGUAGCUCGGA	GAUGUUCGAGCUGCUG	Cleava	
78	316	3	.9	1	20	880	899	UGGC	AAGC	ge	1
trf_3_3	Bra008		20					GCUUCAGUAGCUCGGA	GUCAUCGCAGCUGCUG	Cleava	
78	784	3	.8	1	20	1057	1076	UGGC	AAGC	ge	1
trf_3_3	Bra013		22					UCCGUUGUAGUCUAGC	CAUCAUCAGCUUGGCU	Cleava	
86	528	3	.0	1	24	196	219	UGGUCAGG	AUAACGGG	ge	1
trf_3_3	Bra013		22					UCCGUUGUAGUCUAGC	AUCAGCUUGGCUAUAA	Cleava	
87	528	3	.0	1	20	200	219	UGGU	CGGG	ge	1
trf_3_3	Bra000		15					GGUUCUAUGGUCUAGC	GACCGCUGGACCAGAG	Cleava	
88	719	3	.3	1	20	1680	1699	GGUU	AACU	ge	1
trf_3_3	Bra014		21					GGUUCUAUGGUCUAGC	AGCUGCUAGACCUUGG	Cleava	
88	489	3	.1	1	20	168	187	GGUU	GACC	ge	1

trf_3_408	Bra013584	3	16.4	1	26	885	910	UCCGUUGUCGUCCAGCGGUUAGGAUA	UAUGAUAACCGAUGGAUGUCAAUGGA	Cleavage	1
trf_3_408	Bra000529	3	11.2	1	21	984	1004	UCCGUUGUCGUCCAGCGGUUA	UAACCGGAGGACGAUAAUGGA	Cleavage	1
trf_3_409	Bra013584	3	16.4	1	25	886	910	UCCGUUGUCGUCCAGCGGUUAGGAU	AUGAUAACCGAUGGAUGUCAAUGGA	Cleavage	1
trf_3_409	Bra000529	3	11.2	1	21	984	1004	UCCGUUGUCGUCCAGCGGUUA	UAACCGGAGGACGAUAAUGGA	Cleavage	1
trf_3_412	Bra013584	3	16.4	1	21	890	910	UCCGUUGUCGUCCAGCGGUUA	UAACCGAUGGAUGUCAAUGGA	Cleavage	1
trf_3_412	Bra000529	3	11.2	1	21	984	1004	UCCGUUGUCGUCCAGCGGUUA	UAACCGGAGGACGAUAAUGGA	Cleavage	1
trf_3_413	Bra013584	3	16.4	1	26	885	910	UCCGUUGUCGUCCAGCGGUUAGGAUA	UAUGAUAACCGAUGGAUGUCAAUGGA	Cleavage	1
trf_3_413	Bra000529	3	11.2	1	21	984	1004	UCCGUUGUCGUCCAGCGGUUA	UAACCGGAGGACGAUAAUGGA	Cleavage	1
trf_3_414	Bra013584	3	16.4	1	21	890	910	UCCGUUGUCGUCCAGCGGUUA	UAACCGAUGGAUGUCAAUGGA	Cleavage	1
trf_3_414	Bra000529	3	11.2	1	21	984	1004	UCCGUUGUCGUCCAGCGGUUA	UAACCGGAGGACGAUAAUGGA	Cleavage	1
trf_3_415	Bra013584	3	16.4	1	20	891	910	UCCGUUGUCGUCCAGCGGUU	AACCGAUGGAUGUCAAUGGA	Cleavage	1
trf_3_415	Bra000529	3	11.2	1	20	985	1004	UCCGUUGUCGUCCAGCGGUU	AACCGGAGGACGAUAAUGGA	Cleavage	1
trf_3_420	Bra016601	3	20.8	1	24	415	438	UCCGUCGUAGUCUAGCUGGUUAGG	CUGAAGAAGCUAGGCUGCGACGGA	Cleavage	1
trf_3_421	Bra016601	3	20.8	1	24	415	438	UCCGUCGUAGUCUAGCUGGUUAGG	CUGAAGAAGCUAGGCUGCGACGGA	Cleavage	1
trf_3_422	Bra016601	3	20.8	1	21	418	438	UCCGUCGUAGUCUAGCUGGUU	AAGAAGCUAGGCUGCGACGGA	Cleavage	1
trf_3_423	Bra016601	3	20.8	1	24	415	438	UCCGUCGUAGUCUAGCUGGUUAGG	CUGAAGAAGCUAGGCUGCGACGGA	Cleavage	1
trf_3_425	Bra016601	3	20.8	1	24	415	438	UCCGUCGUAGUCUAGCUGGUUAGG	CUGAAGAAGCUAGGCUGCGACGGA	Cleavage	1

trf_3_426	Bra020755	3	12.6	1	20	454	473	GGGGAUGUAGCUCAAAUGGU	GCCAUUGGAGCUAUAUUCUC	Cleavage	1
trf_3_426	Bra025904	3	17.2	1	20	344	363	GGGGAUGUAGCUCAAAUGGU	ACAAUUUGAGCUUCAUCUUC	Cleavage	1
trf_3_426	Bra024638	3	7.5	1	20	206	225	GGGGAUGUAGCUCAAAUGGU	AACAUUUCGGUUACAUCCCC	Cleavage	1
trf_3_427	Bra016601	3	20.8	1	24	415	438	UCCGUCGUAGUCUAGCUGGUUAGG	CUGAAGAAGCUAGGCUGCGACGGA	Cleavage	1
trf_3_429	Bra016601	3	20.8	1	21	418	438	UCCGUCGUAGUCUAGCUGGUU	AAGAAGCUAGGCUGCGACGGA	Cleavage	1
trf_3_430	Bra016601	3	20.8	1	24	415	438	UCCGUCGUAGUCUAGCUGGUUAGG	CUGAAGAAGCUAGGCUGCGACGGA	Cleavage	1
trf_3_431	Bra016601	3	20.8	1	24	415	438	UCCGUCGUAGUCUAGCUGGUUAGG	CUGAAGAAGCUAGGCUGCGACGGA	Cleavage	1
trf_3_433	Bra016601	3	20.8	1	21	418	438	UCCGUCGUAGUCUAGCUGGUU	AAGAAGCUAGGCUGCGACGGA	Cleavage	1
trf_3_448	Bra007154	2.5	14.1	1	21	510	530	GCACCAGUGGUCUAGUGGUAG	UUACUACUGGACCUUUGGUGC	Cleavage	1
trf_3_464	Bra013528	3	22.0	1	24	196	219	UCCGUUGUAGUCUAGCUGGUCAGG	CAUCAUCAGCUUGGCUAUAACGGG	Cleavage	1
trf_3_481	Bra020755	3	12.6	1	20	454	473	GGGGAUGUAGCUCAAAUGGU	GCCAUUGGAGCUAUAUUCUC	Cleavage	1
trf_3_481	Bra025904	3	17.2	1	20	344	363	GGGGAUGUAGCUCAAAUGGU	ACAAUUUGAGCUUCAUCUUC	Cleavage	1
trf_3_481	Bra024638	3	7.5	1	20	206	225	GGGGAUGUAGCUCAAAUGGU	AACAUUUCGGUUACAUCCCC	Cleavage	1
trf_3_1	Bra033370	3	21.6	1	20	4750	4769	GGGGAUGUAGCUCAAAUGGU	ACCCUUUGGGUAACAUCCCC	Translation	1
trf_3_3	Bra037748	2.5	22.8	1	25	561	585	UCCGUUAUCGUCCAGCGGUUAGGAU	GUUGAAACCGCUGUCUGAUAACGGA	Translation	1
trf_3_4	Bra037748	2.5	22.8	1	25	561	585	UCCGUUAUCGUCCAGCGGUUAGGAU	GUUGAAACCGCUGUCUGAUAACGGA	Translation	1
trf_3_5	Bra037748	2.5	22.8	1	20	566	585	UCCGUUAUCGUCCAGCGGUU	AACCGCUGUCUGAUAACGGA	Translation	1

trf_3_6	Bra037748	2.5	22.8	1	20	566	585	UCCGUUAUCGUCCAGCGGUU	AACCGCUGUCUGAUAACGGA	Translation	1
trf_3_7	Bra037748	2.5	22.8	1	20	566	585	UCCGUUAUCGUCCAGCGGUU	AACCGCUGUCUGAUAACGGA	Translation	1
trf_3_11	Bra037748	2.5	22.8	1	25	561	585	UCCGUUAUCGUCCAGCGGUUAGGAU	GUUGAAACCGCUGUCUGAUAACGGA	Translation	1
trf_3_13	Bra037748	2.5	22.8	1	20	566	585	UCCGUUAUCGUCCAGCGGUU	AACCGCUGUCUGAUAACGGA	Translation	1
trf_3_15	Bra037499	3	14.7	1	22	174	195	UCCGUUGUAGUCUAGCUGGUUA	UGACUGGUUAGGCCACAACGGA	Translation	1
trf_3_24	Bra038641	3	17.8	1	25	1139	1163	GGGUCCAUAGCUCAGUGGUAGAGCA	UGUUUUAUCACUCAGAUAUGGACUC	Translation	1
trf_3_24	Bra003794	3	22.2	1	21	404	424	GGGUCCAUAGCUCAGUGGUAG	CUUCCACUGAUCUGUGGAUCC	Translation	1
trf_3_56	Bra038641	3	17.8	1	25	1139	1163	GGGUCCAUAGCUCAGUGGUAGAGCA	UGUUUUAUCACUCAGAUAUGGACUC	Translation	1
trf_3_56	Bra003794	3	22.2	1	21	404	424	GGGUCCAUAGCUCAGUGGUAG	CUUCCACUGAUCUGUGGAUCC	Translation	1
trf_3_83	Bra020153	2.5	15.4	1	22	1953	1974	UCCGUUGUCGUCCAGCGGUUAG	CCAACUGCUGGAAGACAAUGGG	Translation	1
trf_3_83	Bra012143	2.5	18.5	1	20	392	411	UCCGUUGUCGUCCAGCGGUU	AAUCGCUGAACCACAACGGA	Translation	1
trf_3_83	Bra037748	3	22.8	1	25	561	585	UCCGUUGUCGUCCAGCGGUUAGGAU	GUUGAAACCGCUGUCUGAUAACGGA	Translation	1
trf_3_84	Bra020153	2.5	15.4	1	22	1953	1974	UCCGUUGUCGUCCAGCGGUUAG	CCAACUGCUGGAAGACAAUGGG	Translation	1
trf_3_84	Bra012143	2.5	18.5	1	20	392	411	UCCGUUGUCGUCCAGCGGUU	AAUCGCUGAACCACAACGGA	Translation	1
trf_3_84	Bra037748	3	22.8	1	25	561	585	UCCGUUGUCGUCCAGCGGUUAGGAU	GUUGAAACCGCUGUCUGAUAACGGA	Translation	1
trf_3_85	Bra020153	2.5	15.4	1	22	1953	1974	UCCGUUGUCGUCCAGCGGUUAG	CCAACUGCUGGAAGACAAUGGG	Translation	1
trf_3_85	Bra012143	2.5	18.5	1	20	392	411	UCCGUUGUCGUCCAGCGGUU	AAUCGCUGAACCACAACGGA	Translation	1

trf_3_85	Bra037748	3	22.8	1	20	566	585	UCCGUUGUCGUCCAGCGGUU	AACCGCUGUCUGAUAACGGA	Translation	1
trf_3_86	Bra020153	2.5	15.4	1	22	1953	1974	UCCGUUGUCGUCCAGCGGUUAG	CCAACUGCUGGAAGACAAUGGG	Translation	1
trf_3_86	Bra012143	2.5	18.5	1	20	392	411	UCCGUUGUCGUCCAGCGGUU	AAUCGCUGAACCACAACGGA	Translation	1
trf_3_86	Bra037748	3	22.8	1	25	561	585	UCCGUUGUCGUCCAGCGGUUAGGAU	GUUGAAACCGCUGUCUGAUAACGGA	Translation	1
trf_3_87	Bra020153	2.5	15.4	1	22	1953	1974	UCCGUUGUCGUCCAGCGGUUAG	CCAACUGCUGGAAGACAAUGGG	Translation	1
trf_3_87	Bra012143	2.5	18.5	1	20	392	411	UCCGUUGUCGUCCAGCGGUU	AAUCGCUGAACCACAACGGA	Translation	1
trf_3_87	Bra037748	3	22.8	1	20	566	585	UCCGUUGUCGUCCAGCGGUU	AACCGCUGUCUGAUAACGGA	Translation	1
trf_3_89	Bra020153	2.5	15.4	1	20	1955	1974	UCCGUUGUCGUCCAGCGGUU	AACUGCUGGAAGACAAUGGG	Translation	1
trf_3_89	Bra012143	2.5	18.5	1	20	392	411	UCCGUUGUCGUCCAGCGGUU	AAUCGCUGAACCACAACGGA	Translation	1
trf_3_89	Bra037748	3	22.8	1	20	566	585	UCCGUUGUCGUCCAGCGGUU	AACCGCUGUCUGAUAACGGA	Translation	1
trf_3_127	Bra033370	3	21.6	1	20	4750	4769	GGGGAUGUAGCUCAAAUGGU	ACCCUUUGGGUAACAUCCCC	Translation	1
trf_3_129	Bra009917	2.5	17.7	1	21	865	885	UGGAGUAUAGCCAAGUGGUAA	UUACCACUUGGCCGUGUUCCA	Translation	1
trf_3_129	Bra019342	3	20.5	1	22	1829	1850	UGGAGUAUAGCCAAGUGGUAAG	CAUUCUACUUGGAUGUACUCCA	Translation	1
trf_3_129	Bra014319	3	15.0	1	20	867	886	UGGAGUAUAGCCAAGUGGUA	UACCGUUUGGGUUUACUCCA	Translation	1
trf_3_130	Bra033370	3	21.6	1	20	4750	4769	GGGGAUGUAGCUCAAAUGGU	ACCCUUUGGGUAACAUCCCC	Translation	1
trf_3_131	Bra033370	3	21.6	1	24	4746	4769	GGGGAUGUAGCUCAAAUGGUAGAG	CUGGACCCUUUGGGUAACAUCCCC	Translation	1
trf_3_134	Bra037152	3	12.2	1	22	478	499	GACGGUUUGGCCGAGUGGUCUA	UUGACUACUCGUCCAAACCCUC	Translation	1

trf_3_1	Bra018		7.					GACGGUUUGGCCGAGU	CCCAAGACCACUCCUCG	Transl	
34	257	3	1	1	25	27	51	GGUCUAAGG	AAACCGUC	ation	1
trf_3_1	Bra038		17					GGGUCCAUAGCUCAGU	UGUUUUAUCACUCAGA	Transl	
37	641	3	.8	1	25	1139	1163	GGUAGAGCA	UAUGGACUC	ation	1
trf_3_1	Bra003		22					GGGUCCAUAGCUCAGU	CUUCCACUGAUCUGUG	Transl	
37	794	3	.2	1	21	404	424	GGUAG	GAUCC	ation	1
trf_3_1	Bra036		13					GUGGCUGUAGUUUAG	CAACACUAAAGUGCAG	Transl	
52	328	3	.8	1	20	708	727	UGGUG	CUAC	ation	1
trf_3_1	Bra003		7.					GUGGCUGUAGUUUAG	UACCAUUAA-	Transl	
52	191	3	6	1	20	27	45	UGGUG	CUACAGCCAC	ation	1
trf_3_1	Bra036		13					GUGGCUGUAGUUUAG	CAACACUAAAGUGCAG	Transl	
53	328	3	.8	1	20	708	727	UGGUG	CUAC	ation	1
trf_3_1	Bra027		22					GCCGACUUAGCUCAGU	UACCACAGGGCGAAGU	Transl	
62	297	3	.9	1	20	178	197	GGUA	UGGC	ation	1
trf_3_1	Bra027		22					GCCGACUUAGCUCAGU	UACCACAGGGCGAAGU	Transl	
64	297	3	.9	1	20	178	197	GGUA	UGGC	ation	1
trf_3_1	Bra037		14					UCCGUUGUAGUCUAGC	UGACUGGUUAGGCCAC	Transl	
65	499	3	.7	1	22	174	195	UGGUCA	AACGGA	ation	1
trf_3_2	Bra037		22					UCCGUUAUCGUCCAGC	GUUGAAACCGCUGUCU	Transl	
11	748	2.5	.8	1	25	561	585	GGUUAGGAU	GAUAACGGA	ation	1
trf_3_2	Bra037		22					UCCGUUAUCGUCCAGC	GUUGAAACCGCUGUCU	Transl	
12	748	2.5	.8	1	25	561	585	GGUUAGGAU	GAUAACGGA	ation	1
trf_3_2	Bra037		22					UCCGUUAUCGUCCAGC	AACCGCUGUCUGAUAA	Transl	
14	748	2.5	.8	1	20	566	585	GGUU	CGGA	ation	1
trf_3_2	Bra037		22					UCCGUUAUCGUCCAGC	AACCGCUGUCUGAUAA	Transl	
15	748	2.5	.8	1	20	566	585	GGUU	CGGA	ation	1
trf_3_2	Bra037		22					UCCGUUAUCGUCCAGC	AACCGCUGUCUGAUAA	Transl	
16	748	2.5	.8	1	20	566	585	GGUU	CGGA	ation	1
trf_3_2	Bra037		22					UCCGUUAUCGUCCAGC	AACCGCUGUCUGAUAA	Transl	
18	748	2.5	.8	1	20	566	585	GGUU	CGGA	ation	1
trf_3_2	Bra037		22					UCCGUUAUCGUCCAGC	AACCGCUGUCUGAUAA	Transl	
20	748	2.5	.8	1	20	566	585	GGUU	CGGA	ation	1
trf_3_2	Bra037		22					UCCGUUAUCGUCCAGC	GUUGAAACCGCUGUCU	Transl	
21	748	2.5	.8	1	25	561	585	GGUUAGGAU	GAUAACGGA	ation	1

trf_3_2	Bra038		17					GGGUCCAUAGCUCAGU	UGUUUUAUCACUCAGA	Transl	
42	641	3	.8	1	25	1139	1163	GGUAGAGCA	UAUGGACUC	ation	1
trf_3_2	Bra003		22					GGGUCCAUAGCUCAGU	CUUCCACUGAUCUGUG	Transl	
42	794	3	.2	1	21	404	424	GGUAG	GAUCC	ation	1
trf_3_2	Bra020		15					UCCGUUGUCGUCCAGC	CCAACUGCUGGAAGAC	Transl	
58	153	2.5	.4	1	22	1953	1974	GGUUAG	AAUGGG	ation	1
trf_3_2	Bra012		18					UCCGUUGUCGUCCAGC	AAUCGCUGAACCACAAC	Transl	
58	143	2.5	.5	1	20	392	411	GGUU	GGA	ation	1
trf_3_2	Bra037		22					UCCGUUGUCGUCCAGC	GUUGAAACCGCUGUCU	Transl	
58	748	3	.8	1	25	561	585	GGUUAGGAU	GAUAACGGA	ation	1
trf_3_2	Bra020		15					UCCGUUGUCGUCCAGC	AACUGCUGGAAGACAA	Transl	
59	153	2.5	.4	1	20	1955	1974	GGUU	UGGG	ation	1
trf_3_2	Bra012		18					UCCGUUGUCGUCCAGC	AAUCGCUGAACCACAAC	Transl	
59	143	2.5	.5	1	20	392	411	GGUU	GGA	ation	1
trf_3_2	Bra037		22					UCCGUUGUCGUCCAGC	AACCGCUGUCUGAUAA	Transl	
59	748	3	.8	1	20	566	585	GGUU	CGGA	ation	1
trf_3_2	Bra020		15					UCCGUUGUCGUCCAGC	CCAACUGCUGGAAGAC	Transl	
60	153	2.5	.4	1	22	1953	1974	GGUUAG	AAUGGG	ation	1
trf_3_2	Bra012		18					UCCGUUGUCGUCCAGC	AAUCGCUGAACCACAAC	Transl	
60	143	2.5	.5	1	20	392	411	GGUU	GGA	ation	1
trf_3_2	Bra037		22					UCCGUUGUCGUCCAGC	AACCGCUGUCUGAUAA	Transl	
60	748	3	.8	1	20	566	585	GGUU	CGGA	ation	1
trf_3_2	Bra020		15					UCCGUUGUCGUCCAGC	CCAACUGCUGGAAGAC	Transl	
61	153	2.5	.4	1	22	1953	1974	GGUUAG	AAUGGG	ation	1
trf_3_2	Bra012		18					UCCGUUGUCGUCCAGC	AAUCGCUGAACCACAAC	Transl	
61	143	2.5	.5	1	20	392	411	GGUU	GGA	ation	1
trf_3_2	Bra037		22					UCCGUUGUCGUCCAGC	GUUGAAACCGCUGUCU	Transl	
61	748	3	.8	1	25	561	585	GGUUAGGAU	GAUAACGGA	ation	1
trf_3_2	Bra020		15					UCCGUUGUCGUCCAGC	CCAACUGCUGGAAGAC	Transl	
63	153	2.5	.4	1	22	1953	1974	GGUUAG	AAUGGG	ation	1
trf_3_2	Bra012		18					UCCGUUGUCGUCCAGC	AAUCGCUGAACCACAAC	Transl	
63	143	2.5	.5	1	20	392	411	GGUU	GGA	ation	1
trf_3_2	Bra037		22					UCCGUUGUCGUCCAGC	GUUGAAACCGCUGUCU	Transl	
63	748	3	.8	1	25	561	585	GGUUAGGAU	GAUAACGGA	ation	1

trf_3_264	Bra020153	2.5	15.4	1	22	1953	1974	UCCGUUGUCGUCCAGCGGUUAG	CCAACUGCUGGAAGACAAUGGG	Translation	1
trf_3_264	Bra012143	2.5	18.5	1	20	392	411	UCCGUUGUCGUCCAGCGGUU	AAUCGCUGAACCACAACGGA	Translation	1
trf_3_264	Bra037748	3	22.8	1	20	566	585	UCCGUUGUCGUCCAGCGGUU	AACCGCUGUCUGAUAACGGA	Translation	1
trf_3_267	Bra020153	2.5	15.4	1	22	1953	1974	UCCGUUGUCGUCCAGCGGUUAG	CCAACUGCUGGAAGACAAUGGG	Translation	1
trf_3_267	Bra012143	2.5	18.5	1	20	392	411	UCCGUUGUCGUCCAGCGGUU	AAUCGCUGAACCACAACGGA	Translation	1
trf_3_267	Bra037748	3	22.8	1	20	566	585	UCCGUUGUCGUCCAGCGGUU	AACCGCUGUCUGAUAACGGA	Translation	1
trf_3_269	Bra023904	3	9.9	1	20	1163	1182	GGUUCUAUGGUGUAGUGGUU	AACCAAUACAUGAUAGAAUC	Translation	1
trf_3_270	Bra023904	3	9.9	1	20	1163	1182	GGUUCUAUGGUGUAGUGGUU	AACCAAUACAUGAUAGAAUC	Translation	1
trf_3_271	Bra023904	3	9.9	1	20	1163	1182	GGUUCUAUGGUGUAGUGGUU	AACCAAUACAUGAUAGAAUC	Translation	1
trf_3_272	Bra023904	3	9.9	1	20	1163	1182	GGUUCUAUGGUGUAGUGGUU	AACCAAUACAUGAUAGAAUC	Translation	1
trf_3_274	Bra023904	3	9.9	1	20	1163	1182	GGUUCUAUGGUGUAGUGGUU	AACCAAUACAUGAUAGAAUC	Translation	1
trf_3_276	Bra038641	3	17.8	1	25	1139	1163	GGGUCCAUAGCUCAGUGGUAGAGCA	UGUUUUAUCACUCAGAUAUGGACUC	Translation	1
trf_3_276	Bra003794	3	22.2	1	21	404	424	GGGUCCAUAGCUCAGUGGUAG	CUUCCACUGAUCUGUGGAUCC	Translation	1
trf_3_277	Bra038641	3	17.8	1	25	1139	1163	GGGUCCAUAGCUCAGUGGUAGAGCA	UGUUUUAUCACUCAGAUAUGGACUC	Translation	1
trf_3_277	Bra003794	3	22.2	1	21	404	424	GGGUCCAUAGCUCAGUGGUAG	CUUCCACUGAUCUGUGGAUCC	Translation	1
trf_3_278	Bra038641	3	17.8	1	25	1139	1163	GGGUCCAUAGCUCAGUGGUAGAGCA	UGUUUUAUCACUCAGAUAUGGACUC	Translation	1
trf_3_278	Bra003794	3	22.2	1	21	404	424	GGGUCCAUAGCUCAGUGGUAG	CUUCCACUGAUCUGUGGAUCC	Translation	1

trf_3_2	Bra037		14					UCCGUUGUAGUCUAGC	GACUGGUUAGGCCACA	Transl	
80	499	3	.7	1	21	175	195	UGGUC	ACGGA	ation	1
trf_3_2	Bra023		9.					GGUUCUAUGGUGUAG	AACCAAUACAUGAUAG	Transl	
82	904	3	9	1	20	1163	1182	UGGUU	AAUC	ation	1
trf_3_2	Bra023		9.					GGUUCUAUGGUGUAG	AACCAAUACAUGAUAG	Transl	
83	904	3	9	1	20	1163	1182	UGGUU	AAUC	ation	1
trf_3_2	Bra023		9.					GGUUCUAUGGUGUAG	AACCAAUACAUGAUAG	Transl	
84	904	3	9	1	20	1163	1182	UGGUU	AAUC	ation	1
trf_3_2	Bra023		9.					GGUUCUAUGGUGUAG	AACCAAUACAUGAUAG	Transl	
86	904	3	9	1	20	1163	1182	UGGUU	AAUC	ation	1
trf_3_2	Bra023		9.					GGUUCUAUGGUGUAG	AACCAAUACAUGAUAG	Transl	
87	904	3	9	1	20	1163	1182	UGGUU	AAUC	ation	1
trf_3_2	Bra020		15					UCCGUUGUCGUCCAGC	AACUGCUGGAAGACAA	Transl	
88	153	2.5	.4	1	20	1955	1974	GGUU	UGGG	ation	1
trf_3_2	Bra012		18					UCCGUUGUCGUCCAGC	AAUCGCUGAACCACAAC	Transl	
88	143	2.5	.5	1	20	392	411	GGUU	GGA	ation	1
trf_3_2	Bra037		22					UCCGUUGUCGUCCAGC	AACCGCUGUCUGAUAA	Transl	
88	748	3	.8	1	20	566	585	GGUU	CGGA	ation	1
trf_3_2	Bra020		15					UCCGUUGUCGUCCAGC	CCAACUGCUGGAAGAC	Transl	
89	153	2.5	.4	1	22	1953	1974	GGUUAG	AAUGGG	ation	1
trf_3_2	Bra012		18					UCCGUUGUCGUCCAGC	AAUCGCUGAACCACAAC	Transl	
89	143	2.5	.5	1	20	392	411	GGUU	GGA	ation	1
trf_3_2	Bra037		22					UCCGUUGUCGUCCAGC	GUUGAAACCGCUGUCU	Transl	
89	748	3	.8	1	25	561	585	GGUUAGGAU	GAUAACGGA	ation	1
trf_3_2	Bra020		15					UCCGUUGUCGUCCAGC	CCAACUGCUGGAAGAC	Transl	
90	153	2.5	.4	1	22	1953	1974	GGUUAG	AAUGGG	ation	1
trf_3_2	Bra012		18					UCCGUUGUCGUCCAGC	AAUCGCUGAACCACAAC	Transl	
90	143	2.5	.5	1	20	392	411	GGUU	GGA	ation	1
trf_3_2	Bra037		22					UCCGUUGUCGUCCAGC	AACCGCUGUCUGAUAA	Transl	
90	748	3	.8	1	20	566	585	GGUU	CGGA	ation	1
trf_3_2	Bra020		15					UCCGUUGUCGUCCAGC	CCAACUGCUGGAAGAC	Transl	
91	153	2.5	.4	1	22	1953	1974	GGUUAG	AAUGGG	ation	1
trf_3_2	Bra012		18					UCCGUUGUCGUCCAGC	AAUCGCUGAACCACAAC	Transl	
91	143	2.5	.5	1	20	392	411	GGUU	GGA	ation	1

trf_3_2	Bra037		22					UCCGUUGUCGUCCAGC	GUUGAAACCGCUGUCU	Transl	
91	748	3	.8	1	25	561	585	GGUUAGGAU	GAUAACGGA	ation	1
trf_3_2	Bra020		15					UCCGUUGUCGUCCAGC	CCAACUGCUGGAAGAC	Transl	
92	153	2.5	.4	1	22	1953	1974	GGUUAG	AAUGGG	ation	1
trf_3_2	Bra012		18					UCCGUUGUCGUCCAGC	AAUCGCUGAACCACAAC	Transl	
92	143	2.5	.5	1	20	392	411	GGUU	GGA	ation	1
trf_3_2	Bra037		22					UCCGUUGUCGUCCAGC	AACCGCUGUCUGAUAA	Transl	
92	748	3	.8	1	20	566	585	GGUU	CGGA	ation	1
trf_3_2	Bra020		15					UCCGUUGUCGUCCAGC	CCAACUGCUGGAAGAC	Transl	
94	153	2.5	.4	1	22	1953	1974	GGUUAG	AAUGGG	ation	1
trf_3_2	Bra012		18					UCCGUUGUCGUCCAGC	AAUCGCUGAACCACAAC	Transl	
94	143	2.5	.5	1	20	392	411	GGUU	GGA	ation	1
trf_3_2	Bra037		22					UCCGUUGUCGUCCAGC	AACCGCUGUCUGAUAA	Transl	
94	748	3	.8	1	20	566	585	GGUU	CGGA	ation	1
trf_3_3	Bra036		13					GUGGCUGUAGUUUAG	CAACACUAAAGUGCAG	Transl	
03	328	3	.8	1	20	708	727	UGGUG	CUAC	ation	1
trf_3_3	Bra003		7.					GUGGCUGUAGUUUAG	UACCAUUAA-	Transl	
03	191	3	6	1	20	27	45	UGGUG	CUACAGCCAC	ation	1
trf_3_3	Bra037		14					UCCGUUGUAGUCUAGC	UGACUGGUUAGGCCAC	Transl	
08	499	3	.7	1	22	174	195	UGGUCA	AACGGA	ation	1
trf_3_3	Bra015		24					AUCAGAGUGGCGCAGC	CGUUUCCACUGCACCA	Transl	
11	824	3	.0	1	23	916	938	GGAAGCG	CUCUGAC	ation	1
trf_3_3	Bra033		18					AUCAGAGUGGCGCAGC	UUACGUUGCACCACUU	Transl	
11	352	3	.3	1	20	590	609	GGAA	UGAU	ation	1
trf_3_3	Bra015		24					AUCAGAGUGGCGCAGC	CGUUUCCACUGCACCA	Transl	
12	824	3	.0	1	23	916	938	GGAAGCG	CUCUGAC	ation	1
trf_3_3	Bra033		18					AUCAGAGUGGCGCAGC	UUACGUUGCACCACUU	Transl	
12	352	3	.3	1	20	590	609	GGAA	UGAU	ation	1
trf_3_3	Bra026		22					GAUAGUUUGGCCGAGU	CACUACUCGGCGAGAC	Transl	
14	956	3	.2	1	20	116	135	GGUC	UAUC	ation	1
trf_3_3	Bra026		22					GAUAGUUUGGCCGAGU	CACUACUCGGCGAGAC	Transl	
15	956	3	.2	1	20	116	135	GGUC	UAUC	ation	1
trf_3_3	Bra038		17					GGGUCCAUAGCUCAGU	UGUUUUAUCACUCAGA	Transl	
21	641	3	.8	1	25	1139	1163	GGUAGAGCA	UAUGGACUC	ation	1

trf_3_321	Bra003794	3	22.2	1	21	404	424	GGGUCCAUAGCUCAGUGGUAG	CUUCCACUGAUCUGUGGAUCC	Translation	1
trf_3_322	Bra029882	3	18.0	1	20	290	309	GUCUGGUUGGUGUAGUCGGA	UCCGACUGAUCCAACCGGAC	Translation	1
trf_3_335	Bra027297	3	22.9	1	20	178	197	GCCGACUUAGCUCAGUGGUA	UACCACAGGGCGAAGUUGGC	Translation	1
trf_3_336	Bra038641	3	17.8	1	25	1139	1163	GGGUCCAUAGCUCAGUGGUAGAGCA	UGUUUUAUCACUCAGAUAUGGACUC	Translation	1
trf_3_336	Bra003794	3	22.2	1	21	404	424	GGGUCCAUAGCUCAGUGGUAG	CUUCCACUGAUCUGUGGAUCC	Translation	1
trf_3_341	Bra023904	3	9.9	1	20	1163	1182	GGUUCUAUGGUGUAGUGGUU	AACCAAUACAUGAUAGAAUC	Translation	1
trf_3_342	Bra023904	3	9.9	1	20	1163	1182	GGUUCUAUGGUGUAGUGGUU	AACCAAUACAUGAUAGAAUC	Translation	1
trf_3_344	Bra023904	3	9.9	1	20	1163	1182	GGUUCUAUGGUGUAGUGGUU	AACCAAUACAUGAUAGAAUC	Translation	1
trf_3_345	Bra023904	3	9.9	1	20	1163	1182	GGUUCUAUGGUGUAGUGGUU	AACCAAUACAUGAUAGAAUC	Translation	1
trf_3_347	Bra033370	3	21.6	1	20	4750	4769	GGGGAUGUAGCUCAAAUGGU	ACCCUUUGGGUAACAUCCCC	Translation	1
trf_3_353	Bra033536	3	10.5	1	20	426	445	GGGAUUGUAGUUCAAUCGGU	ACCGAUUGAUCAGCAAUUCC	Translation	1
trf_3_354	Bra034753	3	11.6	1	21	442	462	GGGAUUGUAGUUCAAUUGGAU	AUCCAAAUGAGCAGCAAUCCC	Translation	1
trf_3_354	Bra003597	3	13.1	1	21	1507	1527	GGGAUUGUAGUUCAAUUGGAU	AUCCAGUUAAUCUACAAUUCC	Translation	1
trf_3_354	Bra023172	3	12.4	1	20	614	633	GGGAUUGUAGUUCAAUUGGA	UCCAAGUGAACAUCAAUCCC	Translation	1
trf_3_357	Bra033536	3	10.5	1	20	426	445	GGGAUUGUAGUUCAAUCGGU	ACCGAUUGAUCAGCAAUUCC	Translation	1
trf_3_358	Bra034753	3	11.6	1	20	443	462	GGGAUUGUAGUUCAAUUGGA	UCCAAAUGAGCAGCAAUCCC	Translation	1
trf_3_358	Bra003597	3	13.1	1	20	1508	1527	GGGAUUGUAGUUCAAUUGGA	UCCAGUUAAUCUACAAUUCC	Translation	1

trf_3_358	Bra023172	3	12.4	1	20	614	633	GGGAUUGUAGUUCAAUUGGA	UCCAAGUGAACAUCAAUCCC	Translation	1
trf_3_362	Bra033536	3	10.5	1	20	426	445	GGGAUUGUAGUUCAAUCGGU	ACCGAUUGAUCAGCAAUUCC	Translation	1
trf_3_364	Bra033536	3	10.5	1	20	426	445	GGGAUUGUAGUUCAAUCGGU	ACCGAUUGAUCAGCAAUUCC	Translation	1
trf_3_367	Bra033536	3	10.5	1	20	426	445	GGGAUUGUAGUUCAAUCGGU	ACCGAUUGAUCAGCAAUUCC	Translation	1
trf_3_368	Bra033536	3	10.5	1	20	426	445	GGGAUUGUAGUUCAAUCGGU	ACCGAUUGAUCAGCAAUUCC	Translation	1
trf_3_369	Bra033536	3	10.5	1	20	426	445	GGGAUUGUAGUUCAAUCGGU	ACCGAUUGAUCAGCAAUUCC	Translation	1
trf_3_373	Bra033536	3	10.5	1	20	426	445	GGGAUUGUAGUUCAAUCGGU	ACCGAUUGAUCAGCAAUUCC	Translation	1
trf_3_378	Bra023806	3	16.3	1	20	781	800	GCUUCAGUAGCUCGGAUGGC	ACCAACCGAGCAACUGAAGC	Translation	1
trf_3_380	Bra023904	3	9.9	1	20	1163	1182	GGUUCUAUGGUGUAGUGGUU	AACCAAUACAUGAUAGAAUC	Translation	1
trf_3_381	Bra023904	3	9.9	1	20	1163	1182	GGUUCUAUGGUGUAGUGGUU	AACCAAUACAUGAUAGAAUC	Translation	1
trf_3_382	Bra023904	3	9.9	1	20	1163	1182	GGUUCUAUGGUGUAGUGGUU	AACCAAUACAUGAUAGAAUC	Translation	1
trf_3_383	Bra023904	3	9.9	1	20	1163	1182	GGUUCUAUGGUGUAGUGGUU	AACCAAUACAUGAUAGAAUC	Translation	1
trf_3_384	Bra023904	3	9.9	1	20	1163	1182	GGUUCUAUGGUGUAGUGGUU	AACCAAUACAUGAUAGAAUC	Translation	1
trf_3_386	Bra037499	3	14.7	1	22	174	195	UCCGUUGUAGUCUAGCUGGUCA	UGACUGGUUAGGCCACAACGGA	Translation	1
trf_3_387	Bra037499	3	14.7	1	21	175	195	UCCGUUGUAGUCUAGCUGGUC	GACUGGUUAGGCCACAACGGA	Translation	1
trf_3_389	Bra023904	3	9.9	1	20	1163	1182	GGUUCUAUGGUGUAGUGGUU	AACCAAUACAUGAUAGAAUC	Translation	1
trf_3_391	Bra023904	3	9.9	1	20	1163	1182	GGUUCUAUGGUGUAGUGGUU	AACCAAUACAUGAUAGAAUC	Translation	1

trf_3_392	Bra023904	3	9.9	1	20	1163	1182	GGUUCUAUGGUGUAGUGGUU	AACCAAUACAUGAUAGAAUC	Translation	1
trf_3_393	Bra023904	3	9.9	1	20	1163	1182	GGUUCUAUGGUGUAGUGGUU	AACCAAUACAUGAUAGAAUC	Translation	1
trf_3_395	Bra023904	3	9.9	1	20	1163	1182	GGUUCUAUGGUGUAGUGGUU	AACCAAUACAUGAUAGAAUC	Translation	1
trf_3_396	Bra023904	3	9.9	1	20	1163	1182	GGUUCUAUGGUGUAGUGGUU	AACCAAUACAUGAUAGAAUC	Translation	1
trf_3_408	Bra020153	2.5	15.4	1	22	1953	1974	UCCGUUGUCGUCCAGCGGUUAG	CCAACUGCUGGAAGACAAUGGG	Translation	1
trf_3_408	Bra012143	2.5	18.5	1	20	392	411	UCCGUUGUCGUCCAGCGGUU	AAUCGCUGAACCACAACGGA	Translation	1
trf_3_408	Bra037748	3	22.8	1	25	561	585	UCCGUUGUCGUCCAGCGGUUAGGAU	GUUGAAACCGCUGUCUGAUAACGGA	Translation	1
trf_3_409	Bra020153	2.5	15.4	1	22	1953	1974	UCCGUUGUCGUCCAGCGGUUAG	CCAACUGCUGGAAGACAAUGGG	Translation	1
trf_3_409	Bra012143	2.5	18.5	1	20	392	411	UCCGUUGUCGUCCAGCGGUU	AAUCGCUGAACCACAACGGA	Translation	1
trf_3_409	Bra037748	3	22.8	1	25	561	585	UCCGUUGUCGUCCAGCGGUUAGGAU	GUUGAAACCGCUGUCUGAUAACGGA	Translation	1
trf_3_412	Bra020153	2.5	15.4	1	22	1953	1974	UCCGUUGUCGUCCAGCGGUUAG	CCAACUGCUGGAAGACAAUGGG	Translation	1
trf_3_412	Bra012143	2.5	18.5	1	20	392	411	UCCGUUGUCGUCCAGCGGUU	AAUCGCUGAACCACAACGGA	Translation	1
trf_3_412	Bra037748	3	22.8	1	20	566	585	UCCGUUGUCGUCCAGCGGUU	AACCGCUGUCUGAUAACGGA	Translation	1
trf_3_413	Bra020153	2.5	15.4	1	22	1953	1974	UCCGUUGUCGUCCAGCGGUUAG	CCAACUGCUGGAAGACAAUGGG	Translation	1
trf_3_413	Bra012143	2.5	18.5	1	20	392	411	UCCGUUGUCGUCCAGCGGUU	AAUCGCUGAACCACAACGGA	Translation	1
trf_3_413	Bra037748	3	22.8	1	25	561	585	UCCGUUGUCGUCCAGCGGUUAGGAU	GUUGAAACCGCUGUCUGAUAACGGA	Translation	1
trf_3_414	Bra020153	2.5	15.4	1	22	1953	1974	UCCGUUGUCGUCCAGCGGUUAG	CCAACUGCUGGAAGACAAUGGG	Translation	1

trf_3_414	Bra012143	2.5	18.5	1	20	392	411	UCCGUUGUCGUCCAGCGGUU	AAUCGCUGAACCACAACGGA	Translation	1
trf_3_414	Bra037748	3	22.8	1	20	566	585	UCCGUUGUCGUCCAGCGGUU	AACCGCUGUCUGAUAACGGA	Translation	1
trf_3_415	Bra020153	2.5	15.4	1	20	1955	1974	UCCGUUGUCGUCCAGCGGUU	AACUGCUGGAAGACAAUGGG	Translation	1
trf_3_415	Bra012143	2.5	18.5	1	20	392	411	UCCGUUGUCGUCCAGCGGUU	AAUCGCUGAACCACAACGGA	Translation	1
trf_3_415	Bra037748	3	22.8	1	20	566	585	UCCGUUGUCGUCCAGCGGUU	AACCGCUGUCUGAUAACGGA	Translation	1
trf_3_426	Bra033370	3	21.6	1	20	4750	4769	GGGGAUGUAGCUCAAAUGGU	ACCCUUUGGGUAACAUCCCC	Translation	1
trf_3_464	Bra037499	3	14.7	1	22	174	195	UCCGUUGUAGUCUAGCUGGUCA	UGACUGGUUAGGCCACAACGGA	Translation	1
trf_3_466	Bra023904	3	9.9	1	20	1163	1182	GGUUCUAUGGUGUAGUGGUU	AACCAAUACAUGAUAGAAUC	Translation	1
trf_3_467	Bra023904	3	9.9	1	20	1163	1182	GGUUCUAUGGUGUAGUGGUU	AACCAAUACAUGAUAGAAUC	Translation	1
trf_3_468	Bra023904	3	9.9	1	20	1163	1182	GGUUCUAUGGUGUAGUGGUU	AACCAAUACAUGAUAGAAUC	Translation	1
trf_3_469	Bra023904	3	9.9	1	20	1163	1182	GGUUCUAUGGUGUAGUGGUU	AACCAAUACAUGAUAGAAUC	Translation	1
trf_3_470	Bra023904	3	9.9	1	20	1163	1182	GGUUCUAUGGUGUAGUGGUU	AACCAAUACAUGAUAGAAUC	Translation	1
trf_3_481	Bra033370	3	21.6	1	20	4750	4769	GGGGAUGUAGCUCAAAUGGU	ACCCUUUGGGUAACAUCCCC	Translation	1

tRF Unpollinated Ovules

ncRNA_Acc.	Target_Acc.	Expectation	UPE	ncRNA_start	ncRNA_end	Target_start	Target_end	ncRNA_aligned_fragment	Target_aligned_fragment	Inhibition	Multiplicity
trf_4_1	Bra000529	2.5	11.2	1	21	984	1004	UCCGUUAUCGUCCAGCGGUUA	UAACCGGAGGACGAUAAUGGA	Cleavage	1
trf_4_2	Bra000529	2.5	11.2	1	21	984	1004	UCCGUUAUCGUCCAGCGGUUA	UAACCGGAGGACGAUAAUGGA	Cleavage	1
trf_4_4	Bra000529	2.5	11.2	1	21	984	1004	UCCGUUAUCGUCCAGCGGUUA	UAACCGGAGGACGAUAAUGGA	Cleavage	1
trf_4_5	Bra000529	2.5	11.2	1	20	985	1004	UCCGUUAUCGUCCAGCGGUU	AACCGGAGGACGAUAAUGGA	Cleavage	1
trf_4_41	Bra000529	3	11.2	1	21	984	1004	UCCGUUGUCGUCCAGCGGUUA	UAACCGGAGGACGAUAAUGGA	Cleavage	1
trf_4_42	Bra000529	3	11.2	1	20	985	1004	UCCGUUGUCGUCCAGCGGUU	AACCGGAGGACGAUAAUGGA	Cleavage	1
trf_4_82	Bra000529	2.5	11.2	1	21	984	1004	UCCGUUAUCGUCCAGCGGUUA	UAACCGGAGGACGAUAAUGGA	Cleavage	1
trf_4_83	Bra000529	2.5	11.2	1	21	984	1004	UCCGUUAUCGUCCAGCGGUUA	UAACCGGAGGACGAUAAUGGA	Cleavage	1
trf_4_84	Bra000529	2.5	11.2	1	21	984	1004	UCCGUUAUCGUCCAGCGGUUA	UAACCGGAGGACGAUAAUGGA	Cleavage	1
trf_4_86	Bra000529	2.5	11.2	1	20	985	1004	UCCGUUAUCGUCCAGCGGUU	AACCGGAGGACGAUAAUGGA	Cleavage	1
trf_4_87	Bra000529	2.5	11.2	1	21	984	1004	UCCGUUAUCGUCCAGCGGUUA	UAACCGGAGGACGAUAAUGGA	Cleavage	1
trf_4_102	Bra000529	3	11.2	1	21	984	1004	UCCGUUGUCGUCCAGCGGUUA	UAACCGGAGGACGAUAAUGGA	Cleavage	1
trf_4_103	Bra000529	3	11.2	1	21	984	1004	UCCGUUGUCGUCCAGCGGUUA	UAACCGGAGGACGAUAAUGGA	Cleavage	1
trf_4_104	Bra000529	3	11.2	1	21	984	1004	UCCGUUGUCGUCCAGCGGUUA	UAACCGGAGGACGAUAAUGGA	Cleavage	1
trf_4_110	Bra000529	3	11.2	1	21	984	1004	UCCGUUGUCGUCCAGCGGUUA	UAACCGGAGGACGAUAAUGGA	Cleavage	1

trf_4_111	Bra000529	3	11.2	1	21	984	1004	UCCGUUGUCGUCCAGCGGUUA	UAACCGGAGGACGAUAAUGGA	Cleavage	1
trf_4_169	Bra000529	3	11.2	1	21	984	1004	UCCGUUGUCGUCCAGCGGUUA	UAACCGGAGGACGAUAAUGGA	Cleavage	1
trf_4_170	Bra000529	3	11.2	1	21	984	1004	UCCGUUGUCGUCCAGCGGUUA	UAACCGGAGGACGAUAAUGGA	Cleavage	1
trf_4_172	Bra000529	3	11.2	1	21	984	1004	UCCGUUGUCGUCCAGCGGUUA	UAACCGGAGGACGAUAAUGGA	Cleavage	1
trf_4_60	Bra000719	3	15.3	1	20	1680	1699	GGUUCUAUGGUCUAGCGGUU	GACCGCUGGACCAGAGAACU	Cleavage	1
trf_4_58	Bra000788	3	16.6	1	20	52	71	GACGGUUUGGCCGAGUGGUC	AACCAUUCCGCCAAAUCGUC	Cleavage	1
trf_4_58	Bra002609	3	10.2	1	20	1237	1256	GACGGUUUGGCCGAGUGGUC	GACUUUCCGGCCAAACCGUC	Cleavage	1
trf_4_76	Bra002746	3	16.3	1	21	218	238	AGGGAUAUAACUCAGCGGUAG	CUGCUGCUGAGUUCUAUCCCA	Cleavage	1
trf_4_188	Bra003013	3	16.5	1	20	319	338	GCUGGAGUAGCUCAGUUGGU	CCUAACGGAGCUGCUCCAGC	Cleavage	1
trf_4_58	Bra003534	3	24.7	1	20	3589	3608	GACGGUUUGGCCGAGUGGUC	GGCGAUUCGGCCAAAUUGUC	Cleavage	1
trf_4_144	Bra003597	3	13.1	1	20	1508	1527	GGGAUUGUAGUUCAAUUGGA	UCCAGUUAAUCUACAAUUCC	Translation	1
trf_4_152	Bra003597	3	13.1	1	20	1508	1527	GGGAUUGUAGUUCAAUUGGA	UCCAGUUAAUCUACAAUUCC	Translation	1
trf_4_153	Bra003597	3	13.1	1	20	1508	1527	GGGAUUGUAGUUCAAUUGGA	UCCAGUUAAUCUACAAUUCC	Translation	1
trf_4_188	Bra003602	3	23.1	1	23	271	293	GCUGGAGUAGCUCAGUUGGUUAG	CGAACCAACCGGGCUACUGCAGC	Cleavage	1
trf_4_139	Bra003753	1.5	16.2	1	20	284	303	GGGAUUGUAGUUCAAUUGGU	ACCAGUUGAAUUACAAUCCU	Cleavage	1
trf_4_141	Bra003753	1.5	16.2	1	20	284	303	GGGAUUGUAGUUCAAUUGGU	ACCAGUUGAAUUACAAUCCU	Cleavage	1
trf_4_142	Bra003753	1.5	16.2	1	20	284	303	GGGAUUGUAGUUCAAUUGGU	ACCAGUUGAAUUACAAUCCU	Cleavage	1

trf_4_143	Bra003753	2.5	16.2	1	20	284	303	GGGAUUGUAGUUCAAUCGGU	ACCAGUUGAAUUACAAUCCU	Cleavage	1
trf_4_144	Bra003753	2.5	16.2	1	20	284	303	GGGAUUGUAGUUCAAUUGGA	ACCAGUUGAAUUACAAUCCU	Cleavage	1
trf_4_146	Bra003753	2.5	16.2	1	21	283	303	GGGAUUGUAGUUCAAUUGGCU	AACCAGUUGAAUUACAAUCCU	Cleavage	1
trf_4_149	Bra003753	1.5	16.2	1	20	284	303	GGGAUUGUAGUUCAAUUGGU	ACCAGUUGAAUUACAAUCCU	Cleavage	1
trf_4_150	Bra003753	2.5	16.2	1	20	284	303	GGGAUUGUAGUUCAAUUGGC	ACCAGUUGAAUUACAAUCCU	Cleavage	1
trf_4_151	Bra003753	1.5	16.2	1	20	284	303	GGGAUUGUAGUUCAAUUGGU	ACCAGUUGAAUUACAAUCCU	Cleavage	1
trf_4_152	Bra003753	2.5	16.2	1	20	284	303	GGGAUUGUAGUUCAAUUGGA	ACCAGUUGAAUUACAAUCCU	Cleavage	1
trf_4_153	Bra003753	2.5	16.2	1	20	284	303	GGGAUUGUAGUUCAAUUGGA	ACCAGUUGAAUUACAAUCCU	Cleavage	1
trf_4_154	Bra003753	2.5	16.2	1	20	284	303	GGGAUUGUAGUUCAAUCGGU	ACCAGUUGAAUUACAAUCCU	Cleavage	1
trf_4_155	Bra003753	1.5	16.2	1	20	284	303	GGGAUUGUAGUUCAAUUGGU	ACCAGUUGAAUUACAAUCCU	Cleavage	1
trf_4_156	Bra003753	2.5	16.2	1	20	284	303	GGGAUUGUAGUUCAAUUGGC	ACCAGUUGAAUUACAAUCCU	Cleavage	1
trf_4_157	Bra003753	1.5	16.2	1	20	284	303	GGGAUUGUAGUUCAAUUGGU	ACCAGUUGAAUUACAAUCCU	Cleavage	1
trf_4_158	Bra003753	2.5	16.2	1	20	284	303	GGGAUUGUAGUUCAAUUGGC	ACCAGUUGAAUUACAAUCCU	Cleavage	1
trf_4_160	Bra003753	1.5	16.2	1	20	284	303	GGGAUUGUAGUUCAAUUGGU	ACCAGUUGAAUUACAAUCCU	Cleavage	1
trf_4_188	Bra005116	3	13.5	1	21	233	254	GCUGGAGU-AGCUCAGUUGGUU	AACCAACUGAUCUCACUCCAGC	Translation	1
trf_4_108	Bra007154	2.5	14.1	1	21	510	530	GCACCAGUGGUCUAGUGGUAG	UUACUACUGGACCUUUGGUGC	Cleavage	1
trf_4_40	Bra007535	3	20.1	1	20	51	70	GGUCCCAUGGUCUAGCGGUU	GGCUGCUAGACCUUGGGAUC	Cleavage	1

trf_4_59	Bra007535	3	20.1	1	20	51	70	GGUCCCAUGGUCUAGCGGUU	GGCUGCUAGACCUUGGGAUC	Cleavage	1
trf_4_41	Bra012143	2.5	18.5	1	20	392	411	UCCGUUGUCGUCCAGCGGUU	AAUCGCUGAACCACAACGGA	Translation	1
trf_4_42	Bra012143	2.5	18.5	1	20	392	411	UCCGUUGUCGUCCAGCGGUU	AAUCGCUGAACCACAACGGA	Translation	1
trf_4_102	Bra012143	2.5	18.5	1	20	392	411	UCCGUUGUCGUCCAGCGGUU	AAUCGCUGAACCACAACGGA	Translation	1
trf_4_103	Bra012143	2.5	18.5	1	20	392	411	UCCGUUGUCGUCCAGCGGUU	AAUCGCUGAACCACAACGGA	Translation	1
trf_4_104	Bra012143	2.5	18.5	1	20	392	411	UCCGUUGUCGUCCAGCGGUU	AAUCGCUGAACCACAACGGA	Translation	1
trf_4_110	Bra012143	2.5	18.5	1	20	392	411	UCCGUUGUCGUCCAGCGGUU	AAUCGCUGAACCACAACGGA	Translation	1
trf_4_111	Bra012143	2.5	18.5	1	20	392	411	UCCGUUGUCGUCCAGCGGUU	AAUCGCUGAACCACAACGGA	Translation	1
trf_4_169	Bra012143	2.5	18.5	1	20	392	411	UCCGUUGUCGUCCAGCGGUU	AAUCGCUGAACCACAACGGA	Translation	1
trf_4_170	Bra012143	2.5	18.5	1	20	392	411	UCCGUUGUCGUCCAGCGGUU	AAUCGCUGAACCACAACGGA	Translation	1
trf_4_172	Bra012143	2.5	18.5	1	20	392	411	UCCGUUGUCGUCCAGCGGUU	AAUCGCUGAACCACAACGGA	Translation	1
trf_4_76	Bra012734	3	14.1	1	20	1709	1728	AGGGAUAUAACUCAGCGGUA	UACCUUUGAGUUAUGUUUCU	Cleavage	1
trf_4_40	Bra012946	3	16.6	1	20	957	976	GGUCCCAUGGUCUAGCGGUU	AACUGCUACUCCAUGGGACU	Translation	1
trf_4_59	Bra012946	3	16.6	1	20	957	976	GGUCCCAUGGUCUAGCGGUU	AACUGCUACUCCAUGGGACU	Translation	1
trf_4_139	Bra013336	3	19.5	1	20	267	286	GGGAUUGUAGUUCAAUUGGU	ACUAAUUGAACUAAGAUCUC	Cleavage	1
trf_4_141	Bra013336	3	19.5	1	20	267	286	GGGAUUGUAGUUCAAUUGGU	ACUAAUUGAACUAAGAUCUC	Cleavage	1
trf_4_142	Bra013336	3	19.5	1	20	267	286	GGGAUUGUAGUUCAAUUGGU	ACUAAUUGAACUAAGAUCUC	Cleavage	1

trf_4_149	Bra013336	3	19.5	1	20	267	286	GGGAUUGUAGUUCAAUUGGU	ACUAAUUGAACUAAGAUCUC	Cleavage	1
trf_4_151	Bra013336	3	19.5	1	20	267	286	GGGAUUGUAGUUCAAUUGGU	ACUAAUUGAACUAAGAUCUC	Cleavage	1
trf_4_155	Bra013336	3	19.5	1	20	267	286	GGGAUUGUAGUUCAAUUGGU	ACUAAUUGAACUAAGAUCUC	Cleavage	1
trf_4_157	Bra013336	3	19.5	1	20	267	286	GGGAUUGUAGUUCAAUUGGU	ACUAAUUGAACUAAGAUCUC	Cleavage	1
trf_4_160	Bra013336	3	19.5	1	20	267	286	GGGAUUGUAGUUCAAUUGGU	ACUAAUUGAACUAAGAUCUC	Cleavage	1
trf_4_6	Bra013528	3	22.0	1	24	196	219	UCCGUUGUAGUCUAGCUGGUUAGG	CAUCAUCAGCUUGGCUAUAACGGG	Cleavage	1
trf_4_109	Bra013528	3	22.0	1	24	196	219	UCCGUUGUAGUCUAGCUGGUCAGG	CAUCAUCAGCUUGGCUAUAACGGG	Cleavage	1
trf_4_186	Bra013528	3	22.0	1	24	196	219	UCCGUUGUAGUCUAGCUGGUCAGG	CAUCAUCAGCUUGGCUAUAACGGG	Cleavage	1
trf_4_41	Bra013584	3	16.4	1	25	886	910	UCCGUUGUCGUCCAGCGGUUAGGAU	AUGAUAACCGAUGGAUGUCAAUGGA	Cleavage	1
trf_4_42	Bra013584	3	16.4	1	20	891	910	UCCGUUGUCGUCCAGCGGUU	AACCGAUGGAUGUCAAUGGA	Cleavage	1
trf_4_102	Bra013584	3	16.4	1	26	885	910	UCCGUUGUCGUCCAGCGGUUAGGAUA	UAUGAUAACCGAUGGAUGUCAAUGGA	Cleavage	1
trf_4_103	Bra013584	3	16.4	1	25	886	910	UCCGUUGUCGUCCAGCGGUUAGGAU	AUGAUAACCGAUGGAUGUCAAUGGA	Cleavage	1
trf_4_104	Bra013584	3	16.4	1	21	890	910	UCCGUUGUCGUCCAGCGGUUA	UAACCGAUGGAUGUCAAUGGA	Cleavage	1
trf_4_110	Bra013584	3	16.4	1	21	890	910	UCCGUUGUCGUCCAGCGGUUA	UAACCGAUGGAUGUCAAUGGA	Cleavage	1
trf_4_111	Bra013584	3	16.4	1	25	886	910	UCCGUUGUCGUCCAGCGGUUAGGAU	AUGAUAACCGAUGGAUGUCAAUGGA	Cleavage	1
trf_4_169	Bra013584	3	16.4	1	25	886	910	UCCGUUGUCGUCCAGCGGUUAGGAU	AUGAUAACCGAUGGAUGUCAAUGGA	Cleavage	1
trf_4_170	Bra013584	3	16.4	1	26	885	910	UCCGUUGUCGUCCAGCGGUUAGGAUA	UAUGAUAACCGAUGGAUGUCAAUGGA	Cleavage	1

trf_4_172	Bra013584	3	16.4	1	26	885	910	UCCGUUGUCGUCCAGCGGUUAGGAUA	UAUGAUAACCGAUGGAUGUCAAUGGA	Cleavage	1
trf_4_40	Bra014489	2	21.1	1	20	168	187	GGUCCCAUGGUCUAGCGGUU	AGCUGCUAGACCUUGGGACC	Cleavage	1
trf_4_59	Bra014489	2	21.1	1	20	168	187	GGUCCCAUGGUCUAGCGGUU	AGCUGCUAGACCUUGGGACC	Cleavage	1
trf_4_60	Bra014489	3	21.1	1	20	168	187	GGUUCUAUGGUCUAGCGGUU	AGCUGCUAGACCUUGGGACC	Cleavage	1
trf_4_113	Bra014507	3	17.0	1	23	541	563	GGGGAUGUAGCUCAUAUGGUAGA	UGUAUUAUAUUAGCUAUAUCUCC	Cleavage	1
trf_4_121	Bra015824	3	24.0	1	26	913	938	AUCAGAGUGGCGCAGCGGAAGCGUGG	CUCCGUUUCCACUGCACCACUCUGAC	Translation	1
trf_4_136	Bra015824	3	24.0	1	26	913	938	AUCAGAGUGGCGCAGCGGAAGCGUGG	CUCCGUUUCCACUGCACCACUCUGAC	Translation	1
trf_4_195	Bra015824	3	24.0	1	26	913	938	AUCAGAGUGGCGCAGCGGAAGCGUGG	CUCCGUUUCCACUGCACCACUCUGAC	Translation	1
trf_4_7	Bra016601	3	20.8	1	24	415	438	UCCGUCGUAGUCUAGCUGGUUAGG	CUGAAGAAGCUAGGCUGCGACGGA	Cleavage	1
trf_4_8	Bra016601	3	20.8	1	24	415	438	UCCGUCGUAGUCUAGCUGGUUAGG	CUGAAGAAGCUAGGCUGCGACGGA	Cleavage	1
trf_4_9	Bra016601	3	20.8	1	21	418	438	UCCGUCGUAGUCUAGCUGGUU	AAGAAGCUAGGCUGCGACGGA	Cleavage	1
trf_4_11	Bra016601	3	20.8	1	24	415	438	UCCGUCGUAGUCUAGCUGGUUAGG	CUGAAGAAGCUAGGCUGCGACGGA	Cleavage	1
trf_4_114	Bra016601	3	20.8	1	24	415	438	UCCGUCGUAGUCUAGCUGGUUAGG	CUGAAGAAGCUAGGCUGCGACGGA	Cleavage	1
trf_4_115	Bra016601	3	20.8	1	24	415	438	UCCGUCGUAGUCUAGCUGGUUAGG	CUGAAGAAGCUAGGCUGCGACGGA	Cleavage	1
trf_4_116	Bra016601	3	20.8	1	21	418	438	UCCGUCGUAGUCUAGCUGGUU	AAGAAGCUAGGCUGCGACGGA	Cleavage	1
trf_4_118	Bra016601	3	20.8	1	24	415	438	UCCGUCGUAGUCUAGCUGGUUAGG	CUGAAGAAGCUAGGCUGCGACGGA	Cleavage	1
trf_4_176	Bra016601	3	20.8	1	24	415	438	UCCGUCGUAGUCUAGCUGGUUAGG	CUGAAGAAGCUAGGCUGCGACGGA	Cleavage	1

trf_4_177	Bra016601	3	20.8	1	21	418	438	UCCGUCGUAGUCUAGCUGGUU	AAGAAGCUAGGCUGCGACGGA	Cleavage	1
trf_4_178	Bra016601	3	20.8	1	24	415	438	UCCGUCGUAGUCUAGCUGGUUAGG	CUGAAGAAGCUAGGCUGCGACGGA	Cleavage	1
trf_4_179	Bra016601	3	20.8	1	24	415	438	UCCGUCGUAGUCUAGCUGGUUAGG	CUGAAGAAGCUAGGCUGCGACGGA	Cleavage	1
trf_4_180	Bra016601	3	20.8	1	24	415	438	UCCGUCGUAGUCUAGCUGGUUAGG	CUGAAGAAGCUAGGCUGCGACGGA	Cleavage	1
trf_4_181	Bra016601	3	20.8	1	24	415	438	UCCGUCGUAGUCUAGCUGGUUAGG	CUGAAGAAGCUAGGCUGCGACGGA	Cleavage	1
trf_4_58	Bra018257	3	7.1	1	25	27	51	GACGGUUUGGCCGAGUGGUCUAAGG	CCCAAGACCACUCCUCGAAACCGUC	Translation	1
trf_4_58	Bra019760	3	12.5	1	23	1328	1350	GACGGUUUGGCCGAGUGGUCUAA	UGAGACUACUUGGUCCAGCCGUC	Cleavage	1
trf_4_41	Bra020153	2.5	15.4	1	22	1953	1974	UCCGUUGUCGUCCAGCGGUUAG	CCAACUGCUGGAAGACAAUGGG	Translation	1
trf_4_42	Bra020153	2.5	15.4	1	20	1955	1974	UCCGUUGUCGUCCAGCGGUU	AACUGCUGGAAGACAAUGGG	Translation	1
trf_4_102	Bra020153	2.5	15.4	1	22	1953	1974	UCCGUUGUCGUCCAGCGGUUAG	CCAACUGCUGGAAGACAAUGGG	Translation	1
trf_4_103	Bra020153	2.5	15.4	1	22	1953	1974	UCCGUUGUCGUCCAGCGGUUAG	CCAACUGCUGGAAGACAAUGGG	Translation	1
trf_4_104	Bra020153	2.5	15.4	1	22	1953	1974	UCCGUUGUCGUCCAGCGGUUAG	CCAACUGCUGGAAGACAAUGGG	Translation	1
trf_4_110	Bra020153	2.5	15.4	1	22	1953	1974	UCCGUUGUCGUCCAGCGGUUAG	CCAACUGCUGGAAGACAAUGGG	Translation	1
trf_4_111	Bra020153	2.5	15.4	1	22	1953	1974	UCCGUUGUCGUCCAGCGGUUAG	CCAACUGCUGGAAGACAAUGGG	Translation	1
trf_4_169	Bra020153	2.5	15.4	1	22	1953	1974	UCCGUUGUCGUCCAGCGGUUAG	CCAACUGCUGGAAGACAAUGGG	Translation	1
trf_4_170	Bra020153	2.5	15.4	1	22	1953	1974	UCCGUUGUCGUCCAGCGGUUAG	CCAACUGCUGGAAGACAAUGGG	Translation	1
trf_4_172	Bra020153	2.5	15.4	1	22	1953	1974	UCCGUUGUCGUCCAGCGGUUAG	CCAACUGCUGGAAGACAAUGGG	Translation	1

trf_4_139	Bra021314	3	23.2	1	20	234	253	GGGAUUGUAGUUCAAUUGGU	ACCAACUGAACUGCAAUCUG	Cleavage	1
trf_4_141	Bra021314	3	23.2	1	20	234	253	GGGAUUGUAGUUCAAUUGGU	ACCAACUGAACUGCAAUCUG	Cleavage	1
trf_4_142	Bra021314	3	23.2	1	20	234	253	GGGAUUGUAGUUCAAUUGGU	ACCAACUGAACUGCAAUCUG	Cleavage	1
trf_4_149	Bra021314	3	23.2	1	20	234	253	GGGAUUGUAGUUCAAUUGGU	ACCAACUGAACUGCAAUCUG	Cleavage	1
trf_4_151	Bra021314	3	23.2	1	20	234	253	GGGAUUGUAGUUCAAUUGGU	ACCAACUGAACUGCAAUCUG	Cleavage	1
trf_4_155	Bra021314	3	23.2	1	20	234	253	GGGAUUGUAGUUCAAUUGGU	ACCAACUGAACUGCAAUCUG	Cleavage	1
trf_4_157	Bra021314	3	23.2	1	20	234	253	GGGAUUGUAGUUCAAUUGGU	ACCAACUGAACUGCAAUCUG	Cleavage	1
trf_4_160	Bra021314	3	23.2	1	20	234	253	GGGAUUGUAGUUCAAUUGGU	ACCAACUGAACUGCAAUCUG	Cleavage	1
trf_4_188	Bra022251	3	23.9	1	20	3475	3494	GCUGGAGUAGCUCAGUUGGU	AUCAAUUGGGAUACUCUAGC	Translation	1
trf_4_144	Bra023172	3	12.4	1	20	614	633	GGGAUUGUAGUUCAAUUGGA	UCCAAGUGAACAUCAAUCCC	Translation	1
trf_4_152	Bra023172	3	12.4	1	21	613	633	GGGAUUGUAGUUCAAUUGGAA	UUCCAAGUGAACAUCAAUCCC	Translation	1
trf_4_153	Bra023172	3	12.4	1	20	614	633	GGGAUUGUAGUUCAAUUGGA	UCCAAGUGAACAUCAAUCCC	Translation	1
trf_4_67	Bra027297	3	22.9	1	20	178	197	GCCGACUUAGCUCAGUGGUA	UACCACAGGGCGAAGUUGGC	Translation	1
trf_4_132	Bra027297	3	22.9	1	20	178	197	GCCGACUUAGCUCAGUGGUA	UACCACAGGGCGAAGUUGGC	Translation	1
trf_4_133	Bra027297	3	22.9	1	20	178	197	GCCGACUUAGCUCAGUGGUA	UACCACAGGGCGAAGUUGGC	Translation	1
trf_4_188	Bra029975	3	20.8	1	21	1244	1264	GCUGGAGUAGCUCAGUUGGUU	GGUCAGCUGAGCUACUCAAGC	Cleavage	1
trf_4_1	Bra032111	3	22.4	1	20	727	746	UCCGUUAUCGUCCAGCGGUU	GACGGCUUGACGAUGACGGA	Cleavage	1

trf_4_2	Bra032111	3	22.4	1	20	727	746	UCCGUUAUCGUCCAGCGGUU	GACGGCUUGACGAUGACGGA	Cleavage	1
trf_4_4	Bra032111	3	22.4	1	20	727	746	UCCGUUAUCGUCCAGCGGUU	GACGGCUUGACGAUGACGGA	Cleavage	1
trf_4_5	Bra032111	3	22.4	1	20	727	746	UCCGUUAUCGUCCAGCGGUU	GACGGCUUGACGAUGACGGA	Cleavage	1
trf_4_82	Bra032111	3	22.4	1	20	727	746	UCCGUUAUCGUCCAGCGGUU	GACGGCUUGACGAUGACGGA	Cleavage	1
trf_4_83	Bra032111	3	22.4	1	20	727	746	UCCGUUAUCGUCCAGCGGUU	GACGGCUUGACGAUGACGGA	Cleavage	1
trf_4_84	Bra032111	3	22.4	1	20	727	746	UCCGUUAUCGUCCAGCGGUU	GACGGCUUGACGAUGACGGA	Cleavage	1
trf_4_86	Bra032111	3	22.4	1	20	727	746	UCCGUUAUCGUCCAGCGGUU	GACGGCUUGACGAUGACGGA	Cleavage	1
trf_4_87	Bra032111	3	22.4	1	20	727	746	UCCGUUAUCGUCCAGCGGUU	GACGGCUUGACGAUGACGGA	Cleavage	1
trf_4_188	Bra032168	2.5	22.3	1	20	150	169	GCUGGAGUAGCUCAGUUGGU	AUCGGCUGAGCCACUCCAGC	Translation	1
trf_4_121	Bra033352	3	18.3	1	25	586	609	AUCAGAGUGGCGCAGCGGAAGCGUG	CACG-UUACGUUGCACCACUUUGAU	Translation	1
trf_4_136	Bra033352	3	18.3	1	25	586	609	AUCAGAGUGGCGCAGCGGAAGCGUG	CACG-UUACGUUGCACCACUUUGAU	Translation	1
trf_4_195	Bra033352	3	18.3	1	25	586	609	AUCAGAGUGGCGCAGCGGAAGCGUG	CACG-UUACGUUGCACCACUUUGAU	Translation	1
trf_4_143	Bra033536	3	10.5	1	20	426	445	GGGAUUGUAGUUCAAUCGGU	ACCGAUUGAUCAGCAAUUCC	Translation	1
trf_4_154	Bra033536	3	10.5	1	20	426	445	GGGAUUGUAGUUCAAUCGGU	ACCGAUUGAUCAGCAAUUCC	Translation	1
trf_4_144	Bra034753	3	11.6	1	20	443	462	GGGAUUGUAGUUCAAUUGGA	UCCAAAUGAGCAGCAAUCCC	Translation	1
trf_4_1	Bra034	3	11	1	20	443	462	GGGAUUGUAGUUCAA	UCCAAAUGAGCAGCAA	Transl	1

52	753		.6					UUGGA	UCCC	ation	
trf_4_1	Bra034		11					GGGAUUGUAGUUCAA	UCCAAAUGAGCAGCAA	Transl	
53	753	3	.6	1	20	443	462	UUGGA	UCCC	ation	1
trf_4_5	Bra037		12					GACGGUUUGGCCGAGU	UUGACUACUCGUCCAA	Transl	
8	152	3	.2	1	22	478	499	GGUCUA	ACCCUC	ation	1
	Bra037		14					UCCGUUGUAGUCUAGC	UGACUGGUUAGGCCAC	Transl	
trf_4_6	499	3	.7	1	22	174	195	UGGUUA	AACGGA	ation	1
trf_4_1	Bra037		14					UCCGUUGUAGUCUAGC	UGACUGGUUAGGCCAC	Transl	
09	499	3	.7	1	22	174	195	UGGUCA	AACGGA	ation	1
trf_4_1	Bra037		14					UCCGUUGUAGUCUAGC	UGACUGGUUAGGCCAC	Transl	
86	499	3	.7	1	22	174	195	UGGUCA	AACGGA	ation	1
	Bra037		22					UCCGUUAUCGUCCAGC	GUUGAAACCGCUGUCU	Transl	
trf_4_1	748	2.5	.8	1	25	561	585	GGUUAGGAU	GAUAACGGA	ation	1
	Bra037		22					UCCGUUAUCGUCCAGC	GUUGAAACCGCUGUCU	Transl	
trf_4_2	748	2.5	.8	1	25	561	585	GGUUAGGAU	GAUAACGGA	ation	1
	Bra037		22					UCCGUUAUCGUCCAGC	GUUGAAACCGCUGUCU	Transl	
trf_4_4	748	2.5	.8	1	25	561	585	GGUUAGGAU	GAUAACGGA	ation	1
	Bra037		22					UCCGUUAUCGUCCAGC	AACCGCUGUCUGAUAA	Transl	
trf_4_5	748	2.5	.8	1	20	566	585	GGUU	CGGA	ation	1
trf_4_4	Bra037		22					UCCGUUGUCGUCCAGC	GUUGAAACCGCUGUCU	Transl	
1	748	3	.8	1	25	561	585	GGUUAGGAU	GAUAACGGA	ation	1
trf_4_4	Bra037		22					UCCGUUGUCGUCCAGC	AACCGCUGUCUGAUAA	Transl	
2	748	3	.8	1	20	566	585	GGUU	CGGA	ation	1
trf_4_8	Bra037		22					UCCGUUAUCGUCCAGC	GUUGAAACCGCUGUCU	Transl	
2	748	2.5	.8	1	25	561	585	GGUUAGGAU	GAUAACGGA	ation	1
trf_4_8	Bra037		22					UCCGUUAUCGUCCAGC	GUUGAAACCGCUGUCU	Transl	
3	748	2.5	.8	1	25	561	585	GGUUAGGAU	GAUAACGGA	ation	1
trf_4_8	Bra037		22					UCCGUUAUCGUCCAGC	AACCGCUGUCUGAUAA	Transl	
4	748	2.5	.8	1	20	566	585	GGUU	CGGA	ation	1
trf_4_8	Bra037		22					UCCGUUAUCGUCCAGC	AACCGCUGUCUGAUAA	Transl	
6	748	2.5	.8	1	20	566	585	GGUU	CGGA	ation	1
trf_4_8	Bra037		22					UCCGUUAUCGUCCAGC	GUUGAAACCGCUGUCU	Transl	
7	748	2.5	.8	1	25	561	585	GGUUAGGAU	GAUAACGGA	ation	1
trf_4_1	Bra037	3	22	1	25	561	585	UCCGUUGUCGUCCAGC	GUUGAAACCGCUGUCU	Transl	1

02	748		.8					GGUUAGGAU	GAUAACGGA	ation	
trf_4_1	Bra037		22					UCCGUUGUCGUCCAGC	GUUGAAACCGCUGUCU	Transl	
03	748	3	.8	1	25	561	585	GGUUAGGAU	GAUAACGGA	ation	1
trf_4_1	Bra037		22					UCCGUUGUCGUCCAGC	AACCGCUGUCUGAUAA	Transl	
04	748	3	.8	1	20	566	585	GGUU	CGGA	ation	1
trf_4_1	Bra037		22					UCCGUUGUCGUCCAGC	AACCGCUGUCUGAUAA	Transl	
10	748	3	.8	1	20	566	585	GGUU	CGGA	ation	1
trf_4_1	Bra037		22					UCCGUUGUCGUCCAGC	GUUGAAACCGCUGUCU	Transl	
11	748	3	.8	1	25	561	585	GGUUAGGAU	GAUAACGGA	ation	1
trf_4_1	Bra037		22					UCCGUUGUCGUCCAGC	GUUGAAACCGCUGUCU	Transl	
69	748	3	.8	1	25	561	585	GGUUAGGAU	GAUAACGGA	ation	1
trf_4_1	Bra037		22					UCCGUUGUCGUCCAGC	GUUGAAACCGCUGUCU	Transl	
70	748	3	.8	1	25	561	585	GGUUAGGAU	GAUAACGGA	ation	1
trf_4_1	Bra037		22					UCCGUUGUCGUCCAGC	GUUGAAACCGCUGUCU	Transl	
72	748	3	.8	1	25	561	585	GGUUAGGAU	GAUAACGGA	ation	1

tRF Pollinated Ovules

ncRNA_Acc.	Target_Acc.	Expectation	UPE	ncRNA_start	ncRNA_end	Target_start	Target_end	ncRNA_aligned_fragment	Target_aligned_fragment	Inhibition	Multiplicity
trf_5_1	Bra014489	2	21.1	1	20	168	187	GGUCCCAUGGUCUAGCGGUU	AGCUGCUAGACCUUGGGACC	Cleavage	1
trf_5_1	Bra007535	3	20.1	1	20	51	70	GGUCCCAUGGUCUAGCGGUU	GGCUGCUAGACCUUGGGAUC	Cleavage	1
trf_5_2	Bra014489	2	21.1	1	20	168	187	GGUCCCAUGGUCUAGCGGUU	AGCUGCUAGACCUUGGGACC	Cleavage	1
trf_5_2	Bra007535	3	20.1	1	20	51	70	GGUCCCAUGGUCUAGCGGUU	GGCUGCUAGACCUUGGGAUC	Cleavage	1
trf_5_3	Bra000529	2.5	11.2	1	21	984	1004	UCCGUUAUCGUCCAGCGGUUA	UAACCGGAGGACGAUAAUGGA	Cleavage	1
trf_5_3	Bra032111	3	22.4	1	20	727	746	UCCGUUAUCGUCCAGCGGUU	GACGGCUUGACGAUGACGGA	Cleavage	1
trf_5_6	Bra000529	2.5	11.2	1	21	984	1004	UCCGUUAUCGUCCAGCGGUUA	UAACCGGAGGACGAUAAUGGA	Cleavage	1
trf_5_6	Bra032111	3	22.4	1	20	727	746	UCCGUUAUCGUCCAGCGGUU	GACGGCUUGACGAUGACGGA	Cleavage	1
trf_5_7	Bra000529	2.5	11.2	1	21	984	1004	UCCGUUAUCGUCCAGCGGUUA	UAACCGGAGGACGAUAAUGGA	Cleavage	1
trf_5_7	Bra032111	3	22.4	1	20	727	746	UCCGUUAUCGUCCAGCGGUU	GACGGCUUGACGAUGACGGA	Cleavage	1
trf_5_8	Bra000529	2.5	11.2	1	21	984	1004	UCCGUUAUCGUCCAGCGGUUA	UAACCGGAGGACGAUAAUGGA	Cleavage	1
trf_5_8	Bra032111	3	22.4	1	20	727	746	UCCGUUAUCGUCCAGCGGUU	GACGGCUUGACGAUGACGGA	Cleavage	1
trf_5_9	Bra000529	2.5	11.2	1	20	985	1004	UCCGUUAUCGUCCAGCGGUU	AACCGGAGGACGAUAAUGGA	Cleavage	1
trf_5_9	Bra032111	3	22.4	1	20	727	746	UCCGUUAUCGUCCAGCGGUU	GACGGCUUGACGAUGACGGA	Cleavage	1
trf_5_10	Bra000529	2.5	11.2	1	21	984	1004	UCCGUUAUCGUCCAGCGGUUA	UAACCGGAGGACGAUAAUGGA	Cleavage	1

trf_5_10	Bra032111	3	22.4	1	20	727	746	UCCGUUAUCGUCCAGCGGUU	GACGGCUUGACGAUGACGGA	Cleavage	1
trf_5_11	Bra016601	3	20.8	1	24	415	438	UCCGUCGUAGUCUAGCUGGUUAGG	CUGAAGAAGCUAGGCUGCGACGGA	Cleavage	1
trf_5_13	Bra016601	3	20.8	1	24	415	438	UCCGUCGUAGUCUAGCUGGUUAGG	CUGAAGAAGCUAGGCUGCGACGGA	Cleavage	1
trf_5_14	Bra016601	3	20.8	1	24	415	438	UCCGUCGUAGUCUAGCUGGUUAGG	CUGAAGAAGCUAGGCUGCGACGGA	Cleavage	1
trf_5_15	Bra016601	3	20.8	1	24	415	438	UCCGUCGUAGUCUAGCUGGUUAGG	CUGAAGAAGCUAGGCUGCGACGGA	Cleavage	1
trf_5_16	Bra016601	3	20.8	1	21	418	438	UCCGUCGUAGUCUAGCUGGUU	AAGAAGCUAGGCUGCGACGGA	Cleavage	1
trf_5_17	Bra016601	3	20.8	1	21	418	438	UCCGUCGUAGUCUAGCUGGUU	AAGAAGCUAGGCUGCGACGGA	Cleavage	1
trf_5_26	Bra000719	3	15.3	1	20	1680	1699	GGUUCUAUGGUCUAGCGGUU	GACCGCUGGACCAGAGAACU	Cleavage	1
trf_5_26	Bra014489	3	21.1	1	20	168	187	GGUUCUAUGGUCUAGCGGUU	AGCUGCUAGACCUUGGGACC	Cleavage	1
trf_5_27	Bra013528	3	22.0	1	24	196	219	UCCGUUGUAGUCUAGCUGGUCAGG	CAUCAUCAGCUUGGCUAUAACGGG	Cleavage	1
trf_5_40	Bra007154	2.5	14.1	1	21	510	530	GCACCAGUGGUCUAGUGGUAG	UUACUACUGGACCUUUGGUGC	Cleavage	1
trf_5_41	Bra024638	3	7.5	1	23	203	225	GGGGAUGUAGCUCAAAUGGUAGA	UCCAACAUUUCGGUUACAUCCCC	Cleavage	1
trf_5_41	Bra025904	3	17.2	1	20	344	363	GGGGAUGUAGCUCAAAUGGU	ACAAUUUGAGCUUCAUCUUC	Cleavage	1
trf_5_52	Bra014489	2	21.1	1	20	168	187	GGUCCCAUGGUCUAGCGGUU	AGCUGCUAGACCUUGGGACC	Cleavage	1
trf_5_52	Bra007535	3	20.1	1	20	51	70	GGUCCCAUGGUCUAGCGGUU	GGCUGCUAGACCUUGGGAUC	Cleavage	1
trf_5_53	Bra014489	2	21.1	1	20	168	187	GGUCCCAUGGUCUAGCGGUU	AGCUGCUAGACCUUGGGACC	Cleavage	1
trf_5_53	Bra007535	3	20.1	1	20	51	70	GGUCCCAUGGUCUAGCGGUU	GGCUGCUAGACCUUGGGAUC	Cleavage	1

trf_5_54	Bra013528	3	22.0	1	24	196	219	UCCGUUGUAGUCUAGCUGGUCAGG	CAUCAUCAGCUUGGCUAUAACGGG	Cleavage	1
trf_5_55	Bra013528	3	22.0	1	24	196	219	UCCGUUGUAGUCUAGCUGGUCAGG	CAUCAUCAGCUUGGCUAUAACGGG	Cleavage	1
trf_5_57	Bra013584	3	16.4	1	20	891	910	UCCGUUGUCGUCCAGCGGUU	AACCGAUGGAUGUCAAUGGA	Cleavage	1
trf_5_57	Bra000529	3	11.2	1	20	985	1004	UCCGUUGUCGUCCAGCGGUU	AACCGGAGGACGAUAAUGGA	Cleavage	1
trf_5_58	Bra013584	3	16.4	1	26	885	910	UCCGUUGUCGUCCAGCGGUUAGGAUA	UAUGAUAACCGAUGGAUGUCAAUGGA	Cleavage	1
trf_5_58	Bra000529	3	11.2	1	21	984	1004	UCCGUUGUCGUCCAGCGGUUA	UAACCGGAGGACGAUAAUGGA	Cleavage	1
trf_5_60	Bra013584	3	16.4	1	25	886	910	UCCGUUGUCGUCCAGCGGUUAGGAU	AUGAUAACCGAUGGAUGUCAAUGGA	Cleavage	1
trf_5_60	Bra000529	3	11.2	1	21	984	1004	UCCGUUGUCGUCCAGCGGUUA	UAACCGGAGGACGAUAAUGGA	Cleavage	1
trf_5_78	Bra019760	3	12.5	1	23	1328	1350	GACGGUUUGGCCGAGUGGUCUAA	UGAGACUACUUGGUCCAGCCGUC	Cleavage	1
trf_5_78	Bra000788	3	16.6	1	20	52	71	GACGGUUUGGCCGAGUGGUC	AACCAUUCCGCCAAAUCGUC	Cleavage	1
trf_5_78	Bra003534	3	24.7	1	20	3589	3608	GACGGUUUGGCCGAGUGGUC	GGCGAUUCGGCCAAAUUGUC	Cleavage	1
trf_5_78	Bra002609	3	10.2	1	20	1237	1256	GACGGUUUGGCCGAGUGGUC	GACUUUCCGGCCAAACCGUC	Cleavage	1
trf_5_79	Bra014489	2	21.1	1	20	168	187	GGUCCCAUGGUCUAGCGGUU	AGCUGCUAGACCUUGGGACC	Cleavage	1
trf_5_79	Bra007535	3	20.1	1	20	51	70	GGUCCCAUGGUCUAGCGGUU	GGCUGCUAGACCUUGGGAUC	Cleavage	1
trf_5_80	Bra014489	2	21.1	1	20	168	187	GGUCCCAUGGUCUAGCGGUU	AGCUGCUAGACCUUGGGACC	Cleavage	1
trf_5_80	Bra007535	3	20.1	1	20	51	70	GGUCCCAUGGUCUAGCGGUU	GGCUGCUAGACCUUGGGAUC	Cleavage	1
trf_5_82	Bra013528	3	22.0	1	24	196	219	UCCGUUGUAGUCUAGCUGGUCAGG	CAUCAUCAGCUUGGCUAUAACGGG	Cleavage	1

trf_5_8	Bra000		15					GGUUCUAUGGUCUAGC	GACCGCUGGACCAGAG	Cleava	
4	719	3	.3	1	20	1680	1699	GGUU	AACU	ge	1
trf_5_8	Bra014		21					GGUUCUAUGGUCUAGC	AGCUGCUAGACCUUGG	Cleava	
4	489	3	.1	1	20	168	187	GGUU	GACC	ge	1
trf_5_8	Bra000		15					GGUUCUAUGGUCUAGC	GACCGCUGGACCAGAG	Cleava	
5	719	3	.3	1	20	1680	1699	GGUU	AACU	ge	1
trf_5_8	Bra014		21					GGUUCUAUGGUCUAGC	AGCUGCUAGACCUUGG	Cleava	
5	489	3	.1	1	20	168	187	GGUU	GACC	ge	1
trf_5_9	Bra013		22					UCCGUUGUAGUCUAGC	CAUCAUCAGCUUGGCU	Cleava	
7	528	3	.0	1	24	196	219	UGGUCAGG	AUAACGGG	ge	1
trf_5_1	Bra002		16					AGGGAUAUAACUCAGC	CUGCUGCUGAGUUCUA	Cleava	
10	746	3	.3	1	21	218	238	GGUAG	UCCCA	ge	1
trf_5_1	Bra012		14					AGGGAUAUAACUCAGC	UACCUUUGAGUUAUG	Cleava	
10	734	3	.1	1	20	1709	1728	GGUA	UUUCU	ge	1
trf_5_1	Bra000		15					GGUUCUAUGGUCUAGC	GACCGCUGGACCAGAG	Cleava	
16	719	3	.3	1	20	1680	1699	GGUU	AACU	ge	1
trf_5_1	Bra014		21					GGUUCUAUGGUCUAGC	AGCUGCUAGACCUUGG	Cleava	
16	489	3	.1	1	20	168	187	GGUU	GACC	ge	1
trf_5_1	Bra013		18					GGGUCGAUGCCCGAGC	UAACCGAUCGGGCCUC	Cleava	
17	150	2.5	.5	1	21	93	113	GGUUA	GAUCC	ge	1
trf_5_1	Bra000		11					UCCGUUAUCGUCCAGC	UAACCGGAGGACGAUA	Cleava	
19	529	2.5	.2	1	21	984	1004	GGUUA	AUGGA	ge	1
trf_5_1	Bra032		22					UCCGUUAUCGUCCAGC	GACGGCUUGACGAUGA	Cleava	
19	111	3	.4	1	20	727	746	GGUU	CGGA	ge	1
trf_5_1	Bra000		11					UCCGUUAUCGUCCAGC	UAACCGGAGGACGAUA	Cleava	
21	529	2.5	.2	1	21	984	1004	GGUUA	AUGGA	ge	1
trf_5_1	Bra032		22					UCCGUUAUCGUCCAGC	GACGGCUUGACGAUGA	Cleava	
21	111	3	.4	1	20	727	746	GGUU	CGGA	ge	1
trf_5_1	Bra000		11					UCCGUUAUCGUCCAGC	AACCGGAGGACGAUAA	Cleava	
22	529	2.5	.2	1	20	985	1004	GGUU	UGGA	ge	1
trf_5_1	Bra032		22					UCCGUUAUCGUCCAGC	GACGGCUUGACGAUGA	Cleava	
22	111	3	.4	1	20	727	746	GGUU	CGGA	ge	1
trf_5_1	Bra000		11					UCCGUUAUCGUCCAGC	UAACCGGAGGACGAUA	Cleava	
24	529	2.5	.2	1	21	984	1004	GGUUA	AUGGA	ge	1

trf_5_1	Bra032		22					UCCGUUAUCGUCCAGC	GACGGCUUGACGAUGA	Cleava	
24	111	3	.4	1	20	727	746	GGUU	CGGA	ge	1
trf_5_1	Bra000		11					UCCGUUAUCGUCCAGC	UAACCGGAGGACGAUA	Cleava	
25	529	2.5	.2	1	21	984	1004	GGUUA	AUGGA	ge	1
trf_5_1	Bra032		22					UCCGUUAUCGUCCAGC	GACGGCUUGACGAUGA	Cleava	
25	111	3	.4	1	20	727	746	GGUU	CGGA	ge	1
trf_5_1	Bra000		11					UCCGUUAUCGUCCAGC	UAACCGGAGGACGAUA	Cleava	
26	529	2.5	.2	1	21	984	1004	GGUUA	AUGGA	ge	1
trf_5_1	Bra032		22					UCCGUUAUCGUCCAGC	GACGGCUUGACGAUGA	Cleava	
26	111	3	.4	1	20	727	746	GGUU	CGGA	ge	1
trf_5_1	Bra013		16					UCCGUUGUCGUCCAGC	UAACCGAUGGAUGUCA	Cleava	
46	584	3	.4	1	21	890	910	GGUUA	AUGGA	ge	1
trf_5_1	Bra000		11					UCCGUUGUCGUCCAGC	UAACCGGAGGACGAUA	Cleava	
46	529	3	.2	1	21	984	1004	GGUUA	AUGGA	ge	1
trf_5_1	Bra013		16					UCCGUUGUCGUCCAGC	UAUGAUAACCGAUGGA	Cleava	
49	584	3	.4	1	26	885	910	GGUUAGGAUA	UGUCAAUGGA	ge	1
trf_5_1	Bra000		11					UCCGUUGUCGUCCAGC	UAACCGGAGGACGAUA	Cleava	
49	529	3	.2	1	21	984	1004	GGUUA	AUGGA	ge	1
trf_5_1	Bra013		16					UCCGUUGUCGUCCAGC	AUGAUAACCGAUGGAU	Cleava	
50	584	3	.4	1	25	886	910	GGUUAGGAU	GUCAAUGGA	ge	1
trf_5_1	Bra000		11					UCCGUUGUCGUCCAGC	UAACCGGAGGACGAUA	Cleava	
50	529	3	.2	1	21	984	1004	GGUUA	AUGGA	ge	1
trf_5_1	Bra013		16					UCCGUUGUCGUCCAGC	UAACCGAUGGAUGUCA	Cleava	
54	584	3	.4	1	21	890	910	GGUUA	AUGGA	ge	1
trf_5_1	Bra000		11					UCCGUUGUCGUCCAGC	UAACCGGAGGACGAUA	Cleava	
54	529	3	.2	1	21	984	1004	GGUUA	AUGGA	ge	1
trf_5_1	Bra013		16					UCCGUUGUCGUCCAGC	UAACCGAUGGAUGUCA	Cleava	
55	584	3	.4	1	21	890	910	GGUUA	AUGGA	ge	1
trf_5_1	Bra000		11					UCCGUUGUCGUCCAGC	UAACCGGAGGACGAUA	Cleava	
55	529	3	.2	1	21	984	1004	GGUUA	AUGGA	ge	1
trf_5_1	Bra013		16					UCCGUUGUCGUCCAGC	AUGAUAACCGAUGGAU	Cleava	
58	584	3	.4	1	25	886	910	GGUUAGGAU	GUCAAUGGA	ge	1
trf_5_1	Bra000		11					UCCGUUGUCGUCCAGC	UAACCGGAGGACGAUA	Cleava	
58	529	3	.2	1	21	984	1004	GGUUA	AUGGA	ge	1

trf_5_159	Bra013584	3	16.4	1	20	891	910	UCCGUUGUCGUCCAGCGGUU	AACCGAUGGAUGUCAAUGGA	Cleavage	1
trf_5_159	Bra000529	3	11.2	1	20	985	1004	UCCGUUGUCGUCCAGCGGUU	AACCGGAGGACGAUAAUGGA	Cleavage	1
trf_5_160	Bra016601	3	20.8	1	24	415	438	UCCGUCGUAGUCUAGCUGGUUAGG	CUGAAGAAGCUAGGCUGCGACGGA	Cleavage	1
trf_5_161	Bra016601	3	20.8	1	24	415	438	UCCGUCGUAGUCUAGCUGGUUAGG	CUGAAGAAGCUAGGCUGCGACGGA	Cleavage	1
trf_5_162	Bra016601	3	20.8	1	21	418	438	UCCGUCGUAGUCUAGCUGGUU	AAGAAGCUAGGCUGCGACGGA	Cleavage	1
trf_5_169	Bra009876	3	14.9	1	22	16	37	GGGGAUGUAGCUCAGAUGGUAG	UUAUCAUCUGGGCUUCGUCCUC	Cleavage	1
trf_5_169	Bra016141	3	10.4	1	22	3031	3052	GGGGAUGUAGCUCAGAUGGUAG	UUAUCAUCUGAGCUUCGUUCUC	Cleavage	1
trf_5_169	Bra036771	3	20.2	1	23	5375	5397	GGGGAUGUAGCUCAGAUGGUAGA	UCGACUGGCUGAGCUACAUUCCU	Cleavage	1
trf_5_169	Bra018620	3	8.3	1	20	7	26	GGGGAUGUAGCUCAGAUGGU	GCCACCUCAGCUACAUCCUC	Cleavage	1
trf_5_182	Bra000719	3	15.3	1	20	1680	1699	GGUUCUAUGGUCUAGCGGUU	GACCGCUGGACCAGAGAACU	Cleavage	1
trf_5_182	Bra014489	3	21.1	1	20	168	187	GGUUCUAUGGUCUAGCGGUU	AGCUGCUAGACCUUGGGACC	Cleavage	1
trf_5_183	Bra007154	2.5	14.1	1	21	510	530	GCACCAGUGGUCUAGUGGUAG	UUACUACUGGACCUUUGGUGC	Cleavage	1
trf_5_185	Bra003753	1.5	16.2	1	20	284	303	GGGAUUGUAGUUCAAUUGGU	ACCAGUUGAAUUACAAUCCU	Cleavage	1
trf_5_185	Bra013336	3	19.5	1	20	267	286	GGGAUUGUAGUUCAAUUGGU	ACUAAUUGAACUAAGAUCUC	Cleavage	1
trf_5_185	Bra021314	3	23.2	1	20	234	253	GGGAUUGUAGUUCAAUUGGU	ACCAACUGAACUGCAAUCUG	Cleavage	1
trf_5_186	Bra003753	1.5	16.2	1	20	284	303	GGGAUUGUAGUUCAAUUGGU	ACCAGUUGAAUUACAAUCCU	Cleavage	1
trf_5_186	Bra013336	3	19.5	1	20	267	286	GGGAUUGUAGUUCAAUUGGU	ACUAAUUGAACUAAGAUCUC	Cleavage	1

trf_5_186	Bra021314	3	23.2	1	20	234	253	GGGAUUGUAGUUCAAUUGGU	ACCAACUGAACUGCAAUCUG	Cleavage	1
trf_5_187	Bra003753	1.5	16.2	1	20	284	303	GGGAUUGUAGUUCAAUUGGU	ACCAGUUGAAUUACAAUCCU	Cleavage	1
trf_5_187	Bra013336	3	19.5	1	20	267	286	GGGAUUGUAGUUCAAUUGGU	ACUAAUUGAACUAAGAUCUC	Cleavage	1
trf_5_187	Bra021314	3	23.2	1	20	234	253	GGGAUUGUAGUUCAAUUGGU	ACCAACUGAACUGCAAUCUG	Cleavage	1
trf_5_189	Bra003753	2.5	16.2	1	20	284	303	GGGAUUGUAGUUCAAUCGGU	ACCAGUUGAAUUACAAUCCU	Cleavage	1
trf_5_190	Bra003753	1.5	16.2	1	20	284	303	GGGAUUGUAGUUCAAUUGGU	ACCAGUUGAAUUACAAUCCU	Cleavage	1
trf_5_190	Bra013336	3	19.5	1	20	267	286	GGGAUUGUAGUUCAAUUGGU	ACUAAUUGAACUAAGAUCUC	Cleavage	1
trf_5_190	Bra021314	3	23.2	1	20	234	253	GGGAUUGUAGUUCAAUUGGU	ACCAACUGAACUGCAAUCUG	Cleavage	1
trf_5_192	Bra003753	1.5	16.2	1	20	284	303	GGGAUUGUAGUUCAAUUGGU	ACCAGUUGAAUUACAAUCCU	Cleavage	1
trf_5_192	Bra013336	3	19.5	1	20	267	286	GGGAUUGUAGUUCAAUUGGU	ACUAAUUGAACUAAGAUCUC	Cleavage	1
trf_5_192	Bra021314	3	23.2	1	20	234	253	GGGAUUGUAGUUCAAUUGGU	ACCAACUGAACUGCAAUCUG	Cleavage	1
trf_5_193	Bra003753	2.5	16.2	1	20	284	303	GGGAUUGUAGUUCAAUCGGU	ACCAGUUGAAUUACAAUCCU	Cleavage	1
trf_5_195	Bra003753	1.5	16.2	1	20	284	303	GGGAUUGUAGUUCAAUUGGU	ACCAGUUGAAUUACAAUCCU	Cleavage	1
trf_5_195	Bra013336	3	19.5	1	20	267	286	GGGAUUGUAGUUCAAUUGGU	ACUAAUUGAACUAAGAUCUC	Cleavage	1
trf_5_195	Bra021314	3	23.2	1	20	234	253	GGGAUUGUAGUUCAAUUGGU	ACCAACUGAACUGCAAUCUG	Cleavage	1
trf_5_196	Bra003753	1.5	16.2	1	20	284	303	GGGAUUGUAGUUCAAUUGGU	ACCAGUUGAAUUACAAUCCU	Cleavage	1
trf_5_196	Bra013336	3	19.5	1	20	267	286	GGGAUUGUAGUUCAAUUGGU	ACUAAUUGAACUAAGAUCUC	Cleavage	1

trf_5_196	Bra021314	3	23.2	1	20	234	253	GGGAUUGUAGUUCAAUUGGU	ACCAACUGAACUGCAAUCUG	Cleavage	1
trf_5_197	Bra003753	1.5	16.2	1	20	284	303	GGGAUUGUAGUUCAAUUGGU	ACCAGUUGAAUUACAAUCCU	Cleavage	1
trf_5_197	Bra013336	3	19.5	1	20	267	286	GGGAUUGUAGUUCAAUUGGU	ACUAAUUGAACUAAGAUCUC	Cleavage	1
trf_5_197	Bra021314	3	23.2	1	20	234	253	GGGAUUGUAGUUCAAUUGGU	ACCAACUGAACUGCAAUCUG	Cleavage	1
trf_5_198	Bra003753	2.5	16.2	1	20	284	303	GGGAUUGUAGUUCAAUUGGC	ACCAGUUGAAUUACAAUCCU	Cleavage	1
trf_5_199	Bra003753	2.5	16.2	1	20	284	303	GGGAUUGUAGUUCAAUUGGC	ACCAGUUGAAUUACAAUCCU	Cleavage	1
trf_5_200	Bra003753	2.5	16.2	1	21	283	303	GGGAUUGUAGUUCAAUUGGCU	AACCAGUUGAAUUACAAUCCU	Cleavage	1
trf_5_201	Bra003753	2.5	16.2	1	20	284	303	GGGAUUGUAGUUCAAUUGGC	ACCAGUUGAAUUACAAUCCU	Cleavage	1
trf_5_202	Bra003753	2.5	16.2	1	20	284	303	GGGAUUGUAGUUCAAUUGGA	ACCAGUUGAAUUACAAUCCU	Cleavage	1
trf_5_203	Bra003753	2.5	16.2	1	20	284	303	GGGAUUGUAGUUCAAUUGGG	ACCAGUUGAAUUACAAUCCU	Cleavage	1
trf_5_203	Bra009115	3	15.2	1	20	602	621	GGGAUUGUAGUUCAAUUGGG	CUCAGUUGAACUUCAAUCCA	Cleavage	1
trf_5_205	Bra003753	2.5	16.2	1	20	284	303	GGGAUUGUAGUUCAAUUGGG	ACCAGUUGAAUUACAAUCCU	Cleavage	1
trf_5_205	Bra009115	3	15.2	1	20	602	621	GGGAUUGUAGUUCAAUUGGG	CUCAGUUGAACUUCAAUCCA	Cleavage	1
trf_5_210	Bra000719	3	15.3	1	20	1680	1699	GGUUCUAUGGUCUAGCGGUU	GACCGCUGGACCAGAGAACU	Cleavage	1
trf_5_210	Bra014489	3	21.1	1	20	168	187	GGUUCUAUGGUCUAGCGGUU	AGCUGCUAGACCUUGGGACC	Cleavage	1
trf_5_211	Bra000719	3	15.3	1	20	1680	1699	GGUUCUAUGGUCUAGCGGUU	GACCGCUGGACCAGAGAACU	Cleavage	1
trf_5_211	Bra014489	3	21.1	1	20	168	187	GGUUCUAUGGUCUAGCGGUU	AGCUGCUAGACCUUGGGACC	Cleavage	1

trf_5_2	Bra013		16					UCCGUUGUCGUCCAGC	AUGAUAACCGAUGGAU	Cleava	
17	584	3	.4	1	25	886	910	GGUUAGGAU	GUCAAUGGA	ge	1
trf_5_2	Bra000		11					UCCGUUGUCGUCCAGC	UAACCGGAGGACGAUA	Cleava	
17	529	3	.2	1	21	984	1004	GGUUA	AUGGA	ge	1
trf_5_2	Bra013		16					UCCGUUGUCGUCCAGC	UAACCGAUGGAUGUCA	Cleava	
20	584	3	.4	1	21	890	910	GGUUA	AUGGA	ge	1
trf_5_2	Bra000		11					UCCGUUGUCGUCCAGC	UAACCGGAGGACGAUA	Cleava	
20	529	3	.2	1	21	984	1004	GGUUA	AUGGA	ge	1
trf_5_2	Bra013		16					UCCGUUGUCGUCCAGC	UAUGAUAACCGAUGGA	Cleava	
21	584	3	.4	1	26	885	910	GGUUAGGAUA	UGUCAAUGGA	ge	1
trf_5_2	Bra000		11					UCCGUUGUCGUCCAGC	UAACCGGAGGACGAUA	Cleava	
21	529	3	.2	1	21	984	1004	GGUUA	AUGGA	ge	1
trf_5_2	Bra016		20					UCCGUCGUAGUCUAGC	CUGAAGAAGCUAGGCU	Cleava	
23	601	3	.8	1	24	415	438	UGGUUAGG	GCGACGGA	ge	1
trf_5_2	Bra016		20					UCCGUCGUAGUCUAGC	CUGAAGAAGCUAGGCU	Cleava	
24	601	3	.8	1	24	415	438	UGGUUAGG	GCGACGGA	ge	1
trf_5_2	Bra016		20					UCCGUCGUAGUCUAGC	AAGAAGCUAGGCUGCG	Cleava	
25	601	3	.8	1	21	418	438	UGGUU	ACGGA	ge	1
trf_5_2	Bra016		20					UCCGUCGUAGUCUAGC	CUGAAGAAGCUAGGCU	Cleava	
27	601	3	.8	1	24	415	438	UGGUUAGG	GCGACGGA	ge	1
trf_5_2	Bra024		7.					GGGGAUGUAGCUCAAA	UCCAACAUUUCGGUUA	Cleava	
28	638	3	5	1	23	203	225	UGGUAGA	CAUCCCC	ge	1
trf_5_2	Bra025		17					GGGGAUGUAGCUCAAA	ACAAUUUGAGCUUCAU	Cleava	
28	904	3	.2	1	20	344	363	UGGU	CUUC	ge	1
trf_5_2	Bra016		20					UCCGUCGUAGUCUAGC	CUGAAGAAGCUAGGCU	Cleava	
29	601	3	.8	1	24	415	438	UGGUUAGG	GCGACGGA	ge	1
trf_5_2	Bra016		20					UCCGUCGUAGUCUAGC	AAGAAGCUAGGCUGCG	Cleava	
30	601	3	.8	1	21	418	438	UGGUU	ACGGA	ge	1
trf_5_2	Bra016		20					UCCGUCGUAGUCUAGC	CUGAAGAAGCUAGGCU	Cleava	
31	601	3	.8	1	24	415	438	UGGUUAGG	GCGACGGA	ge	1
trf_5_2	Bra029		20					GCUGGAGUAGCUCAGU	GUCAGCUGAGCUACUC	Cleava	
40	975	3	.8	1	20	1245	1264	UGGU	AAGC	ge	1
trf_5_2	Bra003		16					GCUGGAGUAGCUCAGU	CCUAACGGAGCUGCUC	Cleava	
40	013	3	.5	1	20	319	338	UGGU	CAGC	ge	1

trf_5_240	Bra003602	3	23.1	1	20	274	293	GCUGGAGUAGCUCAGUUGGU	ACCAACCGGGCUACUGCAGC	Cleavage	1
trf_5_1	Bra012946	3	16.6	1	20	957	976	GGUCCCAUGGUCUAGCGGUU	AACUGCUACUCCAUGGGACU	Translation	1
trf_5_2	Bra012946	3	16.6	1	20	957	976	GGUCCCAUGGUCUAGCGGUU	AACUGCUACUCCAUGGGACU	Translation	1
trf_5_3	Bra037748	2.5	22.8	1	25	561	585	UCCGUUAUCGUCCAGCGGUUAGGAU	GUUGAAACCGCUGUCUGAUAACGGA	Translation	1
trf_5_6	Bra037748	2.5	22.8	1	25	561	585	UCCGUUAUCGUCCAGCGGUUAGGAU	GUUGAAACCGCUGUCUGAUAACGGA	Translation	1
trf_5_7	Bra037748	2.5	22.8	1	20	566	585	UCCGUUAUCGUCCAGCGGUU	AACCGCUGUCUGAUAACGGA	Translation	1
trf_5_8	Bra037748	2.5	22.8	1	20	566	585	UCCGUUAUCGUCCAGCGGUU	AACCGCUGUCUGAUAACGGA	Translation	1
trf_5_9	Bra037748	2.5	22.8	1	20	566	585	UCCGUUAUCGUCCAGCGGUU	AACCGCUGUCUGAUAACGGA	Translation	1
trf_5_10	Bra037748	2.5	22.8	1	25	561	585	UCCGUUAUCGUCCAGCGGUUAGGAU	GUUGAAACCGCUGUCUGAUAACGGA	Translation	1
trf_5_27	Bra037499	3	14.7	1	22	174	195	UCCGUUGUAGUCUAGCUGGUCA	UGACUGGUUAGGCCACAACGGA	Translation	1
trf_5_39	Bra015824	3	24.0	1	23	916	938	AUCAGAGUGGCGCAGCGGAAGCG	CGUUUCCACUGCACCACUCUGAC	Translation	1
trf_5_39	Bra033352	3	18.3	1	20	590	609	AUCAGAGUGGCGCAGCGGAA	UUACGUUGCACCACUUUGAU	Translation	1
trf_5_41	Bra033370	3	21.6	1	20	4750	4769	GGGGAUGUAGCUCAAAUGGU	ACCCUUUGGGUAACAUCCCC	Translation	1
trf_5_52	Bra012946	3	16.6	1	20	957	976	GGUCCCAUGGUCUAGCGGUU	AACUGCUACUCCAUGGGACU	Translation	1
trf_5_53	Bra012946	3	16.6	1	20	957	976	GGUCCCAUGGUCUAGCGGUU	AACUGCUACUCCAUGGGACU	Translation	1
trf_5_54	Bra037499	3	14.7	1	22	174	195	UCCGUUGUAGUCUAGCUGGUCA	UGACUGGUUAGGCCACAACGGA	Translation	1
trf_5_55	Bra037499	3	14.7	1	22	174	195	UCCGUUGUAGUCUAGCUGGUCA	UGACUGGUUAGGCCACAACGGA	Translation	1

trf_5_5	Bra020		15					UCCGUUGUCGUCCAGC	AACUGCUGGAAGACAA	Transl	
7	153	2.5	.4	1	20	1955	1974	GGUU	UGGG	ation	1
trf_5_5	Bra012		18					UCCGUUGUCGUCCAGC	AAUCGCUGAACCACAAC	Transl	
7	143	2.5	.5	1	20	392	411	GGUU	GGA	ation	1
trf_5_5	Bra037		22					UCCGUUGUCGUCCAGC	AACCGCUGUCUGAUAA	Transl	
7	748	3	.8	1	20	566	585	GGUU	CGGA	ation	1
trf_5_5	Bra020		15					UCCGUUGUCGUCCAGC	CCAACUGCUGGAAGAC	Transl	
8	153	2.5	.4	1	22	1953	1974	GGUUAG	AAUGGG	ation	1
trf_5_5	Bra012		18					UCCGUUGUCGUCCAGC	AAUCGCUGAACCACAAC	Transl	
8	143	2.5	.5	1	20	392	411	GGUU	GGA	ation	1
trf_5_5	Bra037		22					UCCGUUGUCGUCCAGC	GUUGAAACCGCUGUCU	Transl	
8	748	3	.8	1	25	561	585	GGUUAGGAU	GAUAACGGA	ation	1
trf_5_6	Bra020		15					UCCGUUGUCGUCCAGC	CCAACUGCUGGAAGAC	Transl	
0	153	2.5	.4	1	22	1953	1974	GGUUAG	AAUGGG	ation	1
trf_5_6	Bra012		18					UCCGUUGUCGUCCAGC	AAUCGCUGAACCACAAC	Transl	
0	143	2.5	.5	1	20	392	411	GGUU	GGA	ation	1
trf_5_6	Bra037		22					UCCGUUGUCGUCCAGC	GUUGAAACCGCUGUCU	Transl	
0	748	3	.8	1	25	561	585	GGUUAGGAU	GAUAACGGA	ation	1
trf_5_7	Bra037		12					GACGGUUUGGCCGAGU	UUGACUACUCGUCCAA	Transl	
8	152	3	.2	1	22	478	499	GGUCUA	ACCCUC	ation	1
trf_5_7	Bra018		7.					GACGGUUUGGCCGAGU	CCCAAGACCACUCCUCG	Transl	
8	257	3	1	1	25	27	51	GGUCUAAGG	AAACCGUC	ation	1
trf_5_7	Bra012		16					GGUCCCAUGGUCUAGC	AACUGCUACUCCAUGG	Transl	
9	946	3	.6	1	20	957	976	GGUU	GACU	ation	1
trf_5_8	Bra012		16					GGUCCCAUGGUCUAGC	AACUGCUACUCCAUGG	Transl	
0	946	3	.6	1	20	957	976	GGUU	GACU	ation	1
trf_5_8	Bra015		24					AUCAGAGUGGCGCAGC	CGUUUCCACUGCACCA	Transl	
1	824	3	.0	1	23	916	938	GGAAGCG	CUCUGAC	ation	1
trf_5_8	Bra033		18					AUCAGAGUGGCGCAGC	UUACGUUGCACCACUU	Transl	
1	352	3	.3	1	20	590	609	GGAA	UGAU	ation	1
trf_5_8	Bra037		14					UCCGUUGUAGUCUAGC	UGACUGGUUAGGCCAC	Transl	
2	499	3	.7	1	22	174	195	UGGUCA	AACGGA	ation	1
trf_5_8	Bra015		24					AUCAGAGUGGCGCAGC	CGUUUCCACUGCACCA	Transl	
3	824	3	.0	1	23	916	938	GGAAGCG	CUCUGAC	ation	1

trf_5_83	Bra033352	3	18.3	1	20	590	609	AUCAGAGUGGCGCAGCGGAA	UUACGUUGCACCACUUUGAU	Translation	1
trf_5_96	Bra027297	3	22.9	1	20	178	197	GCCGACUUAGCUCAGUGGUA	UACCACAGGGCGAAGUUGGC	Translation	1
trf_5_97	Bra037499	3	14.7	1	22	174	195	UCCGUUGUAGUCUAGCUGGUCA	UGACUGGUUAGGCCACAACGGA	Translation	1
trf_5_119	Bra037748	2.5	22.8	1	25	561	585	UCCGUUAUCGUCCAGCGGUUAGGAU	GUUGAAACCGCUGUCUGAUAACGGA	Translation	1
trf_5_121	Bra037748	2.5	22.8	1	20	566	585	UCCGUUAUCGUCCAGCGGUU	AACCGCUGUCUGAUAACGGA	Translation	1
trf_5_122	Bra037748	2.5	22.8	1	20	566	585	UCCGUUAUCGUCCAGCGGUU	AACCGCUGUCUGAUAACGGA	Translation	1
trf_5_124	Bra037748	2.5	22.8	1	20	566	585	UCCGUUAUCGUCCAGCGGUU	AACCGCUGUCUGAUAACGGA	Translation	1
trf_5_125	Bra037748	2.5	22.8	1	25	561	585	UCCGUUAUCGUCCAGCGGUUAGGAU	GUUGAAACCGCUGUCUGAUAACGGA	Translation	1
trf_5_126	Bra037748	2.5	22.8	1	25	561	585	UCCGUUAUCGUCCAGCGGUUAGGAU	GUUGAAACCGCUGUCUGAUAACGGA	Translation	1
trf_5_139	Bra015824	3	24.0	1	23	916	938	AUCAGAGUGGCGCAGCGGAAGCG	CGUUUCCACUGCACCACUCUGAC	Translation	1
trf_5_139	Bra033352	3	18.3	1	20	590	609	AUCAGAGUGGCGCAGCGGAA	UUACGUUGCACCACUUUGAU	Translation	1
trf_5_145	Bra015824	3	24.0	1	23	916	938	AUCAGAGUGGCGCAGCGGAAGCG	CGUUUCCACUGCACCACUCUGAC	Translation	1
trf_5_145	Bra033352	3	18.3	1	20	590	609	AUCAGAGUGGCGCAGCGGAA	UUACGUUGCACCACUUUGAU	Translation	1
trf_5_146	Bra020153	2.5	15.4	1	22	1953	1974	UCCGUUGUCGUCCAGCGGUUAG	CCAACUGCUGGAAGACAAUGGG	Translation	1
trf_5_146	Bra012143	2.5	18.5	1	20	392	411	UCCGUUGUCGUCCAGCGGUU	AAUCGCUGAACCACAACGGA	Translation	1
trf_5_146	Bra037748	3	22.8	1	20	566	585	UCCGUUGUCGUCCAGCGGUU	AACCGCUGUCUGAUAACGGA	Translation	1
trf_5_149	Bra020153	2.5	15.4	1	22	1953	1974	UCCGUUGUCGUCCAGCGGUUAG	CCAACUGCUGGAAGACAAUGGG	Translation	1

trf_5_1	Bra012		18					UCCGUUGUCGUCCAGC	AAUCGCUGAACCACAAC	Transl	
49	143	2.5	.5	1	20	392	411	GGUU	GGA	ation	1
trf_5_1	Bra037		22					UCCGUUGUCGUCCAGC	GUUGAAACCGCUGUCU	Transl	
49	748	3	.8	1	25	561	585	GGUUAGGAU	GAUAACGGA	ation	1
trf_5_1	Bra020		15					UCCGUUGUCGUCCAGC	CCAACUGCUGGAAGAC	Transl	
50	153	2.5	.4	1	22	1953	1974	GGUUAG	AAUGGG	ation	1
trf_5_1	Bra012		18					UCCGUUGUCGUCCAGC	AAUCGCUGAACCACAAC	Transl	
50	143	2.5	.5	1	20	392	411	GGUU	GGA	ation	1
trf_5_1	Bra037		22					UCCGUUGUCGUCCAGC	GUUGAAACCGCUGUCU	Transl	
50	748	3	.8	1	25	561	585	GGUUAGGAU	GAUAACGGA	ation	1
trf_5_1	Bra023		9.					GGUUCUAUGGUGUAG	AACCAAUACAUGAUAG	Transl	
51	904	3	9	1	20	1163	1182	UGGUU	AAUC	ation	1
trf_5_1	Bra015		24					AUCAGAGUGGCGCAGC	CGUUUCCACUGCACCA	Transl	
53	824	3	.0	1	23	916	938	GGAAGCG	CUCUGAC	ation	1
trf_5_1	Bra033		18					AUCAGAGUGGCGCAGC	UUACGUUGCACCACUU	Transl	
53	352	3	.3	1	20	590	609	GGAA	UGAU	ation	1
trf_5_1	Bra020		15					UCCGUUGUCGUCCAGC	CCAACUGCUGGAAGAC	Transl	
54	153	2.5	.4	1	22	1953	1974	GGUUAG	AAUGGG	ation	1
trf_5_1	Bra012		18					UCCGUUGUCGUCCAGC	AAUCGCUGAACCACAAC	Transl	
54	143	2.5	.5	1	20	392	411	GGUU	GGA	ation	1
trf_5_1	Bra037		22					UCCGUUGUCGUCCAGC	AACCGCUGUCUGAUAA	Transl	
54	748	3	.8	1	20	566	585	GGUU	CGGA	ation	1
trf_5_1	Bra020		15					UCCGUUGUCGUCCAGC	CCAACUGCUGGAAGAC	Transl	
55	153	2.5	.4	1	22	1953	1974	GGUUAG	AAUGGG	ation	1
trf_5_1	Bra012		18					UCCGUUGUCGUCCAGC	AAUCGCUGAACCACAAC	Transl	
55	143	2.5	.5	1	20	392	411	GGUU	GGA	ation	1
trf_5_1	Bra037		22					UCCGUUGUCGUCCAGC	AACCGCUGUCUGAUAA	Transl	
55	748	3	.8	1	20	566	585	GGUU	CGGA	ation	1
trf_5_1	Bra020		15					UCCGUUGUCGUCCAGC	CCAACUGCUGGAAGAC	Transl	
58	153	2.5	.4	1	22	1953	1974	GGUUAG	AAUGGG	ation	1
trf_5_1	Bra012		18					UCCGUUGUCGUCCAGC	AAUCGCUGAACCACAAC	Transl	
58	143	2.5	.5	1	20	392	411	GGUU	GGA	ation	1
trf_5_1	Bra037		22					UCCGUUGUCGUCCAGC	GUUGAAACCGCUGUCU	Transl	
58	748	3	.8	1	25	561	585	GGUUAGGAU	GAUAACGGA	ation	1

trf_5_159	Bra020153	2.5	15.4	1	20	1955	1974	UCCGUUGUCGUCCAGCGGUU	AACUGCUGGAAGACAAUGGG	Translation	1
trf_5_159	Bra012143	2.5	18.5	1	20	392	411	UCCGUUGUCGUCCAGCGGUU	AAUCGCUGAACCACAACGGA	Translation	1
trf_5_159	Bra037748	3	22.8	1	20	566	585	UCCGUUGUCGUCCAGCGGUU	AACCGCUGUCUGAUAACGGA	Translation	1
trf_5_166	Bra015824	3	24.0	1	23	916	938	AUCAGAGUGGCGCAGCGGAAGCG	CGUUUCCACUGCACCACUCUGAC	Translation	1
trf_5_166	Bra033352	3	18.3	1	20	590	609	AUCAGAGUGGCGCAGCGGAA	UUACGUUGCACCACUUUGAU	Translation	1
trf_5_167	Bra015824	3	24.0	1	23	916	938	AUCAGAGUGGCGCAGCGGAAGCG	CGUUUCCACUGCACCACUCUGAC	Translation	1
trf_5_167	Bra033352	3	18.3	1	20	590	609	AUCAGAGUGGCGCAGCGGAA	UUACGUUGCACCACUUUGAU	Translation	1
trf_5_168	Bra015824	3	24.0	1	23	916	938	AUCAGAGUGGCGCAGCGGAAGCG	CGUUUCCACUGCACCACUCUGAC	Translation	1
trf_5_168	Bra033352	3	18.3	1	20	590	609	AUCAGAGUGGCGCAGCGGAA	UUACGUUGCACCACUUUGAU	Translation	1
trf_5_178	Bra027297	3	22.9	1	20	178	197	GCCGACUUAGCUCAGUGGUA	UACCACAGGGCGAAGUUGGC	Translation	1
trf_5_180	Bra015824	3	24.0	1	23	916	938	AUCAGAGUGGCGCAGCGGAAGCG	CGUUUCCACUGCACCACUCUGAC	Translation	1
trf_5_180	Bra033352	3	18.3	1	20	590	609	AUCAGAGUGGCGCAGCGGAA	UUACGUUGCACCACUUUGAU	Translation	1
trf_5_181	Bra015824	3	24.0	1	23	916	938	AUCAGAGUGGCGCAGCGGAAGCG	CGUUUCCACUGCACCACUCUGAC	Translation	1
trf_5_181	Bra033352	3	18.3	1	20	590	609	AUCAGAGUGGCGCAGCGGAA	UUACGUUGCACCACUUUGAU	Translation	1
trf_5_189	Bra033536	3	10.5	1	20	426	445	GGGAUUGUAGUUCAAUCGGU	ACCGAUUGAUCAGCAAUUCC	Translation	1
trf_5_193	Bra033536	3	10.5	1	20	426	445	GGGAUUGUAGUUCAAUCGGU	ACCGAUUGAUCAGCAAUUCC	Translation	1
trf_5_202	Bra034753	3	11.6	1	20	443	462	GGGAUUGUAGUUCAAUUGGA	UCCAAAUGAGCAGCAAUCCC	Translation	1

trf_5_202	Bra003597	3	13.1	1	20	1508	1527	GGGAUUGUAGUUCAAUUGGA	UCCAGUUAAUCUACAAUUCC	Translation	1
trf_5_202	Bra023172	3	12.4	1	20	614	633	GGGAUUGUAGUUCAAUUGGA	UCCAAGUGAACAUCAAUCCC	Translation	1
trf_5_203	Bra034571	3	9.0	1	20	52	71	GGGAUUGUAGUUCAAUUGGG	CCUAAUUCAACCACAAUCUC	Translation	1
trf_5_205	Bra034571	3	9.0	1	20	52	71	GGGAUUGUAGUUCAAUUGGG	CCUAAUUCAACCACAAUCUC	Translation	1
trf_5_217	Bra020153	2.5	15.4	1	22	1953	1974	UCCGUUGUCGUCCAGCGGUUAG	CCAACUGCUGGAAGACAAUGGG	Translation	1
trf_5_217	Bra012143	2.5	18.5	1	20	392	411	UCCGUUGUCGUCCAGCGGUU	AAUCGCUGAACCACAACGGA	Translation	1
trf_5_217	Bra037748	3	22.8	1	25	561	585	UCCGUUGUCGUCCAGCGGUUAGGAU	GUUGAAACCGCUGUCUGAUAACGGA	Translation	1
trf_5_220	Bra020153	2.5	15.4	1	22	1953	1974	UCCGUUGUCGUCCAGCGGUUAG	CCAACUGCUGGAAGACAAUGGG	Translation	1
trf_5_220	Bra012143	2.5	18.5	1	20	392	411	UCCGUUGUCGUCCAGCGGUU	AAUCGCUGAACCACAACGGA	Translation	1
trf_5_220	Bra037748	3	22.8	1	20	566	585	UCCGUUGUCGUCCAGCGGUU	AACCGCUGUCUGAUAACGGA	Translation	1
trf_5_221	Bra020153	2.5	15.4	1	22	1953	1974	UCCGUUGUCGUCCAGCGGUUAG	CCAACUGCUGGAAGACAAUGGG	Translation	1
trf_5_221	Bra012143	2.5	18.5	1	20	392	411	UCCGUUGUCGUCCAGCGGUU	AAUCGCUGAACCACAACGGA	Translation	1
trf_5_221	Bra037748	3	22.8	1	25	561	585	UCCGUUGUCGUCCAGCGGUUAGGAU	GUUGAAACCGCUGUCUGAUAACGGA	Translation	1
trf_5_228	Bra033370	3	21.6	1	20	4750	4769	GGGGAUGUAGCUCAAAUGGU	ACCCUUUGGGUAACAUCCCC	Translation	1
trf_5_240	Bra032168	2.5	22.3	1	20	150	169	GCUGGAGUAGCUCAGUUGGU	AUCGGCUGAGCCACUCCAGC	Translation	1
trf_5_240	Bra022251	3	23.9	1	20	3475	3494	GCUGGAGUAGCUCAGUUGGU	AUCAAUUGGGAUACUCUAGC	Translation	1
trf_5_240	Bra005116	3	13.5	1	20	234	254	GCUGGAGU-AGCUCAGUUGGU	ACCAACUGAUCUCACUCCAGC	Translation	1

trf_5_2	Bra015		24					AUCAGAGUGGCGCAGC	CGUUUCCACUGCACCA	Transl	
48	824	3	.0	1	23	916	938	GGAAGCG	CUCUGAC	ation	1
trf_5_2	Bra033		18					AUCAGAGUGGCGCAGC	UUACGUUGCACCACUU	Transl	
48	352	3	.3	1	20	590	609	GGAA	UGAU	ation	1
trf_5_2	Bra015		24					AUCAGAGUGGCGCAGC	CGUUUCCACUGCACCA	Transl	
54	824	3	.0	1	23	916	938	GGAAGCG	CUCUGAC	ation	1
trf_5_2	Bra033		18					AUCAGAGUGGCGCAGC	UUACGUUGCACCACUU	Transl	
54	352	3	.3	1	20	590	609	GGAA	UGAU	ation	1

tRF Embryo

ncRNA_Acc.	Target_Acc.	Expectation	UPE	ncRNA_start	ncRNA_end	Target_start	Target_end	ncRNA_aligned_fragment	Target_aligned_fragment	Inhibition	Multiplicity
trf_6_1	Bra016584	3	9.9	1	20	1045	1064	GUCGUUGUAGUAUAGUGGUA	UACUAUUAUACUACAAAGGC	Cleavage	1
trf_6_1	Bra035409	3	10.4	1	20	1328	1347	GUCGUUGUAGUAUAGUGGUA	UACCACAAUACUUCAACGAG	Cleavage	1
trf_6_2	Bra016584	3	9.9	1	20	1045	1064	GUCGUUGUAGUAUAGUGGUA	UACUAUUAUACUACAAAGGC	Cleavage	1
trf_6_2	Bra035409	3	10.4	1	20	1328	1347	GUCGUUGUAGUAUAGUGGUA	UACCACAAUACUUCAACGAG	Cleavage	1
trf_6_3	Bra016584	3	9.9	1	20	1045	1064	GUCGUUGUAGUAUAGUGGUA	UACUAUUAUACUACAAAGGC	Cleavage	1
trf_6_3	Bra035409	3	10.4	1	20	1328	1347	GUCGUUGUAGUAUAGUGGUA	UACCACAAUACUUCAACGAG	Cleavage	1
trf_6_4	Bra024638	3	7.5	1	23	203	225	GGGGAUGUAGCUCAAAUGGUAGA	UCCAACAUUUCGGUUACAUCCCC	Cleavage	1
trf_6_4	Bra025904	3	17.2	1	20	344	363	GGGGAUGUAGCUCAAAUGGU	ACAAUUUGAGCUUCAUCUUC	Cleavage	1
trf_6_5	Bra024638	3	7.5	1	24	202	225	GGGGAUGUAGCUCAAAUGGUAGAG	UUCCAACAUUUCGGUUACAUCCCC	Cleavage	1
trf_6_5	Bra025904	3	17.2	1	20	344	363	GGGGAUGUAGCUCAAAUGGU	ACAAUUUGAGCUUCAUCUUC	Cleavage	1
trf_6_6	Bra007154	2.5	14.1	1	20	511	530	GCACCAGUGGUCUAGUGGUA	UACUACUGGACCUUUGGUGC	Cleavage	1
trf_6_7	Bra024638	3	7.5	1	23	203	225	GGGGAUGUAGCUCAAAUGGUAGA	UCCAACAUUUCGGUUACAUCCCC	Cleavage	1
trf_6_7	Bra025904	3	17.2	1	20	344	363	GGGGAUGUAGCUCAAAUGGU	ACAAUUUGAGCUUCAUCUUC	Cleavage	1
trf_6_10	Bra007154	2.5	14.1	1	20	511	530	GCACCAGUGGUCUAGUGGUA	UACUACUGGACCUUUGGUGC	Cleavage	1
trf_6_11	Bra000529	2.5	11.2	1	21	984	1004	UCCGUUAUCGUCCAGCGGUUA	UAACCGGAGGACGAUAAUGGA	Cleavage	1

trf_6_11	Bra032111	3	22.4	1	20	727	746	UCCGUUAUCGUCCAGCGGUU	GACGGCUUGACGAUGACGGA	Cleavage	1
trf_6_12	Bra000529	2.5	11.2	1	21	984	1004	UCCGUUAUCGUCCAGCGGUUA	UAACCGGAGGACGAUAAUGGA	Cleavage	1
trf_6_12	Bra032111	3	22.4	1	20	727	746	UCCGUUAUCGUCCAGCGGUU	GACGGCUUGACGAUGACGGA	Cleavage	1
trf_6_13	Bra000529	2.5	11.2	1	20	985	1004	UCCGUUAUCGUCCAGCGGUU	AACCGGAGGACGAUAAUGGA	Cleavage	1
trf_6_13	Bra032111	3	22.4	1	20	727	746	UCCGUUAUCGUCCAGCGGUU	GACGGCUUGACGAUGACGGA	Cleavage	1
trf_6_14	Bra000529	2.5	11.2	1	21	984	1004	UCCGUUAUCGUCCAGCGGUUA	UAACCGGAGGACGAUAAUGGA	Cleavage	1
trf_6_14	Bra032111	3	22.4	1	20	727	746	UCCGUUAUCGUCCAGCGGUU	GACGGCUUGACGAUGACGGA	Cleavage	1
trf_6_15	Bra013528	3	22.0	1	24	196	219	UCCGUUGUAGUCUAGCUGGUUAGG	CAUCAUCAGCUUGGCUAUAACGGG	Cleavage	1
trf_6_16	Bra024638	3	7.5	1	23	203	225	GGGGAUGUAGCUCAAAUGGUAGA	UCCAACAUUUCGGUUACAUCCCC	Cleavage	1
trf_6_16	Bra025904	3	17.2	1	20	344	363	GGGGAUGUAGCUCAAAUGGU	ACAAUUUGAGCUUCAUCUUC	Cleavage	1
trf_6_17	Bra020755	3	12.6	1	20	454	473	GGGGAUGUAGCUCAAAUGGU	GCCAUUGGAGCUAUAUUCUC	Cleavage	1
trf_6_17	Bra025904	3	17.2	1	20	344	363	GGGGAUGUAGCUCAAAUGGU	ACAAUUUGAGCUUCAUCUUC	Cleavage	1
trf_6_17	Bra024638	3	7.5	1	20	206	225	GGGGAUGUAGCUCAAAUGGU	AACAUUUCGGUUACAUCCCC	Cleavage	1
trf_6_18	Bra016601	3	20.8	1	24	415	438	UCCGUCGUAGUCUAGCUGGUUAGG	CUGAAGAAGCUAGGCUGCGACGGA	Cleavage	1
trf_6_19	Bra016601	3	20.8	1	24	415	438	UCCGUCGUAGUCUAGCUGGUUAGG	CUGAAGAAGCUAGGCUGCGACGGA	Cleavage	1
trf_6_20	Bra016601	3	20.8	1	24	415	438	UCCGUCGUAGUCUAGCUGGUUAGG	CUGAAGAAGCUAGGCUGCGACGGA	Cleavage	1
trf_6_22	Bra016601	3	20.8	1	24	415	438	UCCGUCGUAGUCUAGCUGGUUAGG	CUGAAGAAGCUAGGCUGCGACGGA	Cleavage	1

trf_6_23	Bra016601	3	20.8	1	21	418	438	UCCGUCGUAGUCUAGCUGGUU	AAGAAGCUAGGCUGCGACGGA	Cleavage	1
trf_6_24	Bra016584	3	9.9	1	20	1045	1064	GUCGUUGUAGUAUAGUGGUA	UACUAUUAUACUACAAAGGC	Cleavage	1
trf_6_24	Bra035409	3	10.4	1	20	1328	1347	GUCGUUGUAGUAUAGUGGUA	UACCACAAUACUUCAACGAG	Cleavage	1
trf_6_25	Bra016584	3	9.9	1	20	1045	1064	GUCGUUGUAGUAUAGUGGUA	UACUAUUAUACUACAAAGGC	Cleavage	1
trf_6_25	Bra035409	3	10.4	1	20	1328	1347	GUCGUUGUAGUAUAGUGGUA	UACCACAAUACUUCAACGAG	Cleavage	1
trf_6_26	Bra016584	3	9.9	1	20	1045	1064	GUCGUUGUAGUAUAGUGGUA	UACUAUUAUACUACAAAGGC	Cleavage	1
trf_6_26	Bra035409	3	10.4	1	20	1328	1347	GUCGUUGUAGUAUAGUGGUA	UACCACAAUACUUCAACGAG	Cleavage	1
trf_6_27	Bra016584	3	9.9	1	20	1045	1064	GUCGUUGUAGUAUAGUGGUA	UACUAUUAUACUACAAAGGC	Cleavage	1
trf_6_27	Bra035409	3	10.4	1	20	1328	1347	GUCGUUGUAGUAUAGUGGUA	UACCACAAUACUUCAACGAG	Cleavage	1
trf_6_28	Bra016584	3	9.9	1	20	1045	1064	GUCGUUGUAGUAUAGUGGUA	UACUAUUAUACUACAAAGGC	Cleavage	1
trf_6_28	Bra035409	3	10.4	1	20	1328	1347	GUCGUUGUAGUAUAGUGGUA	UACCACAAUACUUCAACGAG	Cleavage	1
trf_6_29	Bra016584	3	9.9	1	20	1045	1064	GUCGUUGUAGUAUAGUGGUA	UACUAUUAUACUACAAAGGC	Cleavage	1
trf_6_29	Bra035409	3	10.4	1	20	1328	1347	GUCGUUGUAGUAUAGUGGUA	UACCACAAUACUUCAACGAG	Cleavage	1
trf_6_30	Bra016584	3	9.9	1	20	1045	1064	GUCGUUGUAGUAUAGUGGUA	UACUAUUAUACUACAAAGGC	Cleavage	1
trf_6_30	Bra035409	3	10.4	1	20	1328	1347	GUCGUUGUAGUAUAGUGGUA	UACCACAAUACUUCAACGAG	Cleavage	1
trf_6_31	Bra016584	3	9.9	1	20	1045	1064	GUCGUUGUAGUAUAGUGGUA	UACUAUUAUACUACAAAGGC	Cleavage	1
trf_6_31	Bra035409	3	10.4	1	20	1328	1347	GUCGUUGUAGUAUAGUGGUA	UACCACAAUACUUCAACGAG	Cleavage	1

trf_6_34	Bra024638	3	7.5	1	23	203	225	GGGGAUGUAGCUCAAAUGGUAGA	UCCAACAUUUCGGUUACAUCCCC	Cleavage	1
trf_6_34	Bra025904	3	17.2	1	20	344	363	GGGGAUGUAGCUCAAAUGGU	ACAAUUUGAGCUUCAUCUUC	Cleavage	1
trf_6_35	Bra036771	3	20.2	1	24	5374	5397	GGGGAUGUAGCUCAGAUGGUAGAG	CUCGACUGGCUGAGCUACAUUCCU	Cleavage	1
trf_6_35	Bra009876	3	14.9	1	24	14	37	GGGGAUGUAGCUCAGAUGGUAGAG	CGUUAUCAUCUGGGCUUCGUCCUC	Cleavage	1
trf_6_35	Bra016141	3	10.4	1	24	3029	3052	GGGGAUGUAGCUCAGAUGGUAGAG	CAUUAUCAUCUGAGCUUCGUUCUC	Cleavage	1
trf_6_35	Bra018620	3	8.3	1	20	7	26	GGGGAUGUAGCUCAGAUGGU	GCCACCUCAGCUACAUCCUC	Cleavage	1
trf_6_36	Bra009876	3	14.9	1	22	16	37	GGGGAUGUAGCUCAGAUGGUAG	UUAUCAUCUGGGCUUCGUCCUC	Cleavage	1
trf_6_36	Bra016141	3	10.4	1	22	3031	3052	GGGGAUGUAGCUCAGAUGGUAG	UUAUCAUCUGAGCUUCGUUCUC	Cleavage	1
trf_6_36	Bra036771	3	20.2	1	23	5375	5397	GGGGAUGUAGCUCAGAUGGUAGA	UCGACUGGCUGAGCUACAUUCCU	Cleavage	1
trf_6_36	Bra018620	3	8.3	1	20	7	26	GGGGAUGUAGCUCAGAUGGU	GCCACCUCAGCUACAUCCUC	Cleavage	1
trf_6_44	Bra013528	3	22.0	1	24	196	219	UCCGUUGUAGUCUAGCUGGUCAGG	CAUCAUCAGCUUGGCUAUAACGGG	Cleavage	1
trf_6_53	Bra016584	3	9.9	1	20	1045	1064	GUCGUUGUAGUAUAGUGGUA	UACUAUUAUACUACAAAGGC	Cleavage	1
trf_6_53	Bra035409	3	10.4	1	20	1328	1347	GUCGUUGUAGUAUAGUGGUA	UACCACAAUACUUCAACGAG	Cleavage	1
trf_6_54	Bra016584	3	9.9	1	20	1045	1064	GUCGUUGUAGUAUAGUGGUA	UACUAUUAUACUACAAAGGC	Cleavage	1
trf_6_54	Bra035409	3	10.4	1	20	1328	1347	GUCGUUGUAGUAUAGUGGUA	UACCACAAUACUUCAACGAG	Cleavage	1
trf_6_55	Bra016584	3	9.9	1	20	1045	1064	GUCGUUGUAGUAUAGUGGUA	UACUAUUAUACUACAAAGGC	Cleavage	1
trf_6_55	Bra035409	3	10.4	1	20	1328	1347	GUCGUUGUAGUAUAGUGGUA	UACCACAAUACUUCAACGAG	Cleavage	1

trf_6_56	Bra016584	3	9.9	1	20	1045	1064	GUCGUUGUAGUAUAGUGGUA	UACUAUUAUACUACAAAGGC	Cleavage	1
trf_6_56	Bra035409	3	10.4	1	20	1328	1347	GUCGUUGUAGUAUAGUGGUA	UACCACAAUACUUCAACGAG	Cleavage	1
trf_6_57	Bra016584	3	9.9	1	20	1045	1064	GUCGUUGUAGUAUAGUGGUA	UACUAUUAUACUACAAAGGC	Cleavage	1
trf_6_57	Bra035409	3	10.4	1	20	1328	1347	GUCGUUGUAGUAUAGUGGUA	UACCACAAUACUUCAACGAG	Cleavage	1
trf_6_58	Bra016584	3	9.9	1	20	1045	1064	GUCGUUGUAGUAUAGUGGUA	UACUAUUAUACUACAAAGGC	Cleavage	1
trf_6_58	Bra035409	3	10.4	1	20	1328	1347	GUCGUUGUAGUAUAGUGGUA	UACCACAAUACUUCAACGAG	Cleavage	1
trf_6_59	Bra016584	3	9.9	1	20	1045	1064	GUCGUUGUAGUAUAGUGGUA	UACUAUUAUACUACAAAGGC	Cleavage	1
trf_6_59	Bra035409	3	10.4	1	20	1328	1347	GUCGUUGUAGUAUAGUGGUA	UACCACAAUACUUCAACGAG	Cleavage	1
trf_6_60	Bra016584	3	9.9	1	20	1045	1064	GUCGUUGUAGUAUAGUGGUA	UACUAUUAUACUACAAAGGC	Cleavage	1
trf_6_60	Bra035409	3	10.4	1	20	1328	1347	GUCGUUGUAGUAUAGUGGUA	UACCACAAUACUUCAACGAG	Cleavage	1
trf_6_61	Bra016584	3	9.9	1	20	1045	1064	GUCGUUGUAGUAUAGUGGUA	UACUAUUAUACUACAAAGGC	Cleavage	1
trf_6_61	Bra035409	3	10.4	1	20	1328	1347	GUCGUUGUAGUAUAGUGGUA	UACCACAAUACUUCAACGAG	Cleavage	1
trf_6_62	Bra016584	3	9.9	1	20	1045	1064	GUCGUUGUAGUAUAGUGGUA	UACUAUUAUACUACAAAGGC	Cleavage	1
trf_6_63	Bra016584	3	9.9	1	20	1045	1064	GUCGUUGUAGUAUAGUGGUA	UACUAUUAUACUACAAAGGC	Cleavage	1
trf_6_63	Bra035409	3	10.4	1	20	1328	1347	GUCGUUGUAGUAUAGUGGUA	UACCACAAUACUUCAACGAG	Cleavage	1
trf_6_64	Bra016584	3	9.9	1	20	1045	1064	GUCGUUGUAGUAUAGUGGUA	UACUAUUAUACUACAAAGGC	Cleavage	1
trf_6_64	Bra035409	3	10.4	1	20	1328	1347	GUCGUUGUAGUAUAGUGGUA	UACCACAAUACUUCAACGAG	Cleavage	1

trf_6_65	Bra016584	3	9.9	1	20	1045	1064	GUCGUUGUAGUAUAGUGGUA	UACUAUUAUACUACAAAGGC	Cleavage	1
trf_6_65	Bra035409	3	10.4	1	20	1328	1347	GUCGUUGUAGUAUAGUGGUA	UACCACAAUACUUCAACGAG	Cleavage	1
trf_6_66	Bra016584	3	9.9	1	20	1045	1064	GUCGUUGUAGUAUAGUGGUA	UACUAUUAUACUACAAAGGC	Cleavage	1
trf_6_66	Bra035409	3	10.4	1	20	1328	1347	GUCGUUGUAGUAUAGUGGUA	UACCACAAUACUUCAACGAG	Cleavage	1
trf_6_68	Bra007154	2.5	14.1	1	20	511	530	GCACCAGUGGUCUAGUGGUA	UACUACUGGACCUUUGGUGC	Cleavage	1
trf_6_72	Bra009876	3	14.9	1	22	16	37	GGGGAUGUAGCUCAGAUGGUAG	UUAUCAUCUGGGCUUCGUCCUC	Cleavage	1
trf_6_72	Bra016141	3	10.4	1	22	3031	3052	GGGGAUGUAGCUCAGAUGGUAG	UUAUCAUCUGAGCUUCGUUCUC	Cleavage	1
trf_6_72	Bra036771	3	20.2	1	23	5375	5397	GGGGAUGUAGCUCAGAUGGUAGA	UCGACUGGCUGAGCUACAUUCCU	Cleavage	1
trf_6_72	Bra018620	3	8.3	1	20	7	26	GGGGAUGUAGCUCAGAUGGU	GCCACCUCAGCUACAUCCUC	Cleavage	1
trf_6_73	Bra016584	3	9.9	1	20	1045	1064	GUCGUUGUAGUAUAGUGGUA	UACUAUUAUACUACAAAGGC	Cleavage	1
trf_6_73	Bra035409	3	10.4	1	20	1328	1347	GUCGUUGUAGUAUAGUGGUA	UACCACAAUACUUCAACGAG	Cleavage	1
trf_6_74	Bra016584	3	9.9	1	20	1045	1064	GUCGUUGUAGUAUAGUGGUA	UACUAUUAUACUACAAAGGC	Cleavage	1
trf_6_74	Bra035409	3	10.4	1	20	1328	1347	GUCGUUGUAGUAUAGUGGUA	UACCACAAUACUUCAACGAG	Cleavage	1
trf_6_75	Bra016584	3	9.9	1	20	1045	1064	GUCGUUGUAGUAUAGUGGUA	UACUAUUAUACUACAAAGGC	Cleavage	1
trf_6_75	Bra035409	3	10.4	1	20	1328	1347	GUCGUUGUAGUAUAGUGGUA	UACCACAAUACUUCAACGAG	Cleavage	1
trf_6_76	Bra016584	3	9.9	1	20	1045	1064	GUCGUUGUAGUAUAGUGGUA	UACUAUUAUACUACAAAGGC	Cleavage	1
trf_6_76	Bra035409	3	10.4	1	20	1328	1347	GUCGUUGUAGUAUAGUGGUA	UACCACAAUACUUCAACGAG	Cleavage	1

trf_6_78	Bra007154	2.5	14.1	1	20	511	530	GCACCAGUGGUCUAGUGGUA	UACUACUGGACCUUUGGUGC	Cleavage	1
trf_6_79	Bra007154	2.5	14.1	1	20	511	530	GCACCAGUGGUCUAGUGGUA	UACUACUGGACCUUUGGUGC	Cleavage	1
trf_6_81	Bra007154	2.5	14.1	1	21	510	530	GCACCAGUGGUCUAGUGGUAG	UUACUACUGGACCUUUGGUGC	Cleavage	1
trf_6_85	Bra024638	3	7.5	1	23	203	225	GGGGAUGUAGCUCAAAUGGUAGA	UCCAACAUUUCGGUUACAUCCCC	Cleavage	1
trf_6_85	Bra025904	3	17.2	1	20	344	363	GGGGAUGUAGCUCAAAUGGU	ACAAUUUGAGCUUCAUCUUC	Cleavage	1
trf_6_86	Bra020755	3	12.6	1	20	454	473	GGGGAUGUAGCUCAAAUGGU	GCCAUUGGAGCUAUAUUCUC	Cleavage	1
trf_6_86	Bra025904	3	17.2	1	20	344	363	GGGGAUGUAGCUCAAAUGGU	ACAAUUUGAGCUUCAUCUUC	Cleavage	1
trf_6_86	Bra024638	3	7.5	1	20	206	225	GGGGAUGUAGCUCAAAUGGU	AACAUUUCGGUUACAUCCCC	Cleavage	1
trf_6_99	Bra013528	3	22.0	1	24	196	219	UCCGUUGUAGUCUAGCUGGUCAGG	CAUCAUCAGCUUGGCUAUAACGGG	Cleavage	1
trf_6_100	Bra013528	3	22.0	1	24	196	219	UCCGUUGUAGUCUAGCUGGUCAGG	CAUCAUCAGCUUGGCUAUAACGGG	Cleavage	1
trf_6_101	Bra007154	2.5	14.1	1	21	510	530	GCACCAGUGGUCUAGUGGUAG	UUACUACUGGACCUUUGGUGC	Cleavage	1
trf_6_102	Bra007154	2.5	14.1	1	20	511	530	GCACCAGUGGUCUAGUGGUA	UACUACUGGACCUUUGGUGC	Cleavage	1
trf_6_104	Bra013528	3	22.0	1	24	196	219	UCCGUUGUAGUCUAGCUGGUCAGG	CAUCAUCAGCUUGGCUAUAACGGG	Cleavage	1
trf_6_106	Bra007154	2.5	14.1	1	20	511	530	GCACCAGUGGUCUAGUGGUA	UACUACUGGACCUUUGGUGC	Cleavage	1
trf_6_107	Bra000791	2.5	15.9	1	23	954	976	GGGUGUUUGGUCUAGUGGUAUGA	UUACACCACCAGACCAGACACCC	Cleavage	1
trf_6_107	Bra001870	2.5	19.8	1	25	814	838	GGGUGUUUGGUCUAGUGGUAUGAUU	GAGCCUAUCACUGGACCAAACACUA	Cleavage	1
trf_6_107	Bra003793	3	12.4	1	25	516	540	GGGUGUUUGGUCUAGUGGUAUGAUU	AGCCUUAUCACUUGACCAAGCAUCU	Cleavage	1

trf_6_1	Bra007		14					GCACCAGUGGUCUAGU	UACUACUGGACCUUUG	Cleava	
08	154	2.5	.1	1	20	511	530	GGUA	GUGC	ge	1
trf_6_1	Bra009		14					GGGGAUGUAGCUCAGA	UUAUCAUCUGGGCUUC	Cleava	
10	876	3	.9	1	22	16	37	UGGUAG	GUCCUC	ge	1
trf_6_1	Bra016		10					GGGGAUGUAGCUCAGA	UUAUCAUCUGAGCUUC	Cleava	
10	141	3	.4	1	22	3031	3052	UGGUAG	GUUCUC	ge	1
trf_6_1	Bra036		20					GGGGAUGUAGCUCAGA	UCGACUGGCUGAGCUA	Cleava	
10	771	3	.2	1	23	5375	5397	UGGUAGA	CAUUCCU	ge	1
trf_6_1	Bra018		8.					GGGGAUGUAGCUCAGA	GCCACCUCAGCUACAUC	Cleava	
10	620	3	3	1	20	7	26	UGGU	CUC	ge	1
trf_6_1	Bra013		16					UCCGUUGUCGUCCAGC	AUGAUAACCGAUGGAU	Cleava	
11	584	3	.4	1	25	886	910	GGUUAGGAU	GUCAAUGGA	ge	1
trf_6_1	Bra000		11					UCCGUUGUCGUCCAGC	UAACCGGAGGACGAUA	Cleava	
11	529	3	.2	1	21	984	1004	GGUUA	AUGGA	ge	1
trf_6_1	Bra013		16					UCCGUUGUCGUCCAGC	AACCGAUGGAUGUCAA	Cleava	
12	584	3	.4	1	20	891	910	GGUU	UGGA	ge	1
trf_6_1	Bra000		11					UCCGUUGUCGUCCAGC	AACCGGAGGACGAUAA	Cleava	
12	529	3	.2	1	20	985	1004	GGUU	UGGA	ge	1
trf_6_1	Bra013		16					UCCGUUGUCGUCCAGC	UAACCGAUGGAUGUCA	Cleava	
13	584	3	.4	1	21	890	910	GGUUA	AUGGA	ge	1
trf_6_1	Bra000		11					UCCGUUGUCGUCCAGC	UAACCGGAGGACGAUA	Cleava	
13	529	3	.2	1	21	984	1004	GGUUA	AUGGA	ge	1
trf_6_1	Bra013		16					UCCGUUGUCGUCCAGC	UAACCGAUGGAUGUCA	Cleava	
16	584	3	.4	1	21	890	910	GGUUA	AUGGA	ge	1
trf_6_1	Bra000		11					UCCGUUGUCGUCCAGC	UAACCGGAGGACGAUA	Cleava	
16	529	3	.2	1	21	984	1004	GGUUA	AUGGA	ge	1
trf_6_1	Bra013		16					UCCGUUGUCGUCCAGC	UAUGAUAACCGAUGGA	Cleava	
17	584	3	.4	1	26	885	910	GGUUAGGAUA	UGUCAAUGGA	ge	1
trf_6_1	Bra000		11					UCCGUUGUCGUCCAGC	UAACCGGAGGACGAUA	Cleava	
17	529	3	.2	1	21	984	1004	GGUUA	AUGGA	ge	1
trf_6_1	Bra013		16					UCCGUUGUCGUCCAGC	UAUGAUAACCGAUGGA	Cleava	
18	584	3	.4	1	26	885	910	GGUUAGGAUA	UGUCAAUGGA	ge	1
trf_6_1	Bra000		11					UCCGUUGUCGUCCAGC	UAACCGGAGGACGAUA	Cleava	
18	529	3	.2	1	21	984	1004	GGUUA	AUGGA	ge	1

trf_6_1	Bra024		7.					GGGGAUGUAGCUCAAA	UCCAACAUUUCGGUUA	Cleava	
43	638	3	5	1	23	203	225	UGGUAGA	CAUCCCC	ge	1
trf_6_1	Bra025		17					GGGGAUGUAGCUCAAA	ACAAUUUGAGCUUCAU	Cleava	
43	904	3	.2	1	20	344	363	UGGU	CUUC	ge	1
trf_6_1	Bra014		17					GGGGAUGUAGCUCAUA	UGUAUUAUAUUAGCU	Cleava	
44	507	3	.0	1	23	541	563	UGGUAGA	AUAUCUCC	ge	1
trf_6_1	Bra024		7.					GGGGAUGUAGCUCAAA	UCCAACAUUUCGGUUA	Cleava	
45	638	3	5	1	23	203	225	UGGUAGA	CAUCCCC	ge	1
trf_6_1	Bra025		17					GGGGAUGUAGCUCAAA	ACAAUUUGAGCUUCAU	Cleava	
45	904	3	.2	1	20	344	363	UGGU	CUUC	ge	1
trf_6_1	Bra016		9.					GUCGUUGUAGUAUAG	UACUAUUAUACUACAA	Cleava	
48	584	3	9	1	20	1045	1064	UGGUA	AGGC	ge	1
trf_6_1	Bra035		10					GUCGUUGUAGUAUAG	UACCACAAUACUUCAA	Cleava	
48	409	3	.4	1	20	1328	1347	UGGUA	CGAG	ge	1
trf_6_1	Bra016		9.					GUCGUUGUAGUAUAG	UACUAUUAUACUACAA	Cleava	
49	584	3	9	1	20	1045	1064	UGGUA	AGGC	ge	1
trf_6_1	Bra035		10					GUCGUUGUAGUAUAG	UACCACAAUACUUCAA	Cleava	
49	409	3	.4	1	20	1328	1347	UGGUA	CGAG	ge	1
trf_6_1	Bra016		9.					GUCGUUGUAGUAUAG	UACUAUUAUACUACAA	Cleava	
50	584	3	9	1	20	1045	1064	UGGUA	AGGC	ge	1
trf_6_1	Bra035		10					GUCGUUGUAGUAUAG	UACCACAAUACUUCAA	Cleava	
50	409	3	.4	1	20	1328	1347	UGGUA	CGAG	ge	1
trf_6_1	Bra016		9.					GUCGUUGUAGUAUAG	UACUAUUAUACUACAA	Cleava	
51	584	3	9	1	20	1045	1064	UGGUA	AGGC	ge	1
trf_6_1	Bra035		10					GUCGUUGUAGUAUAG	UACCACAAUACUUCAA	Cleava	
51	409	3	.4	1	20	1328	1347	UGGUA	CGAG	ge	1
trf_6_1	Bra019		12					GACGGUUUGGCCGAGU	UGAGACUACUUGGUCC	Cleava	
52	760	3	.5	1	23	1328	1350	GGUCUAA	AGCCGUC	ge	1
trf_6_1	Bra000		16					GACGGUUUGGCCGAGU	AACCAUUCCGCCAAAUC	Cleava	
52	788	3	.6	1	20	52	71	GGUC	GUC	ge	1
trf_6_1	Bra003		24					GACGGUUUGGCCGAGU	GGCGAUUCGGCCAAAU	Cleava	
52	534	3	.7	1	20	3589	3608	GGUC	UGUC	ge	1
trf_6_1	Bra002		10					GACGGUUUGGCCGAGU	GACUUUCCGGCCAAAC	Cleava	
52	609	3	.2	1	20	1237	1256	GGUC	CGUC	ge	1

trf_6_155	Bra007154	2.5	14.1	1	20	511	530	GCACCAGUGGUCUAGUGGUA	UACUACUGGACCUUUGGUGC	Cleavage	1
trf_6_157	Bra013528	3	22.0	1	24	196	219	UCCGUUGUAGUCUAGCUGGUCAGG	CAUCAUCAGCUUGGCUAUAACGGG	Cleavage	1
trf_6_159	Bra024638	3	7.5	1	23	203	225	GGGGAUGUAGCUCAAAUGGUAGA	UCCAACAUUUCGGUUACAUCCCC	Cleavage	1
trf_6_159	Bra025904	3	17.2	1	20	344	363	GGGGAUGUAGCUCAAAUGGU	ACAAUUUGAGCUUCAUCUUC	Cleavage	1
trf_6_160	Bra020755	3	12.6	1	20	454	473	GGGGAUGUAGCUCAAAUGGU	GCCAUUGGAGCUAUAUUCUC	Cleavage	1
trf_6_160	Bra025904	3	17.2	1	20	344	363	GGGGAUGUAGCUCAAAUGGU	ACAAUUUGAGCUUCAUCUUC	Cleavage	1
trf_6_160	Bra024638	3	7.5	1	20	206	225	GGGGAUGUAGCUCAAAUGGU	AACAUUUCGGUUACAUCCCC	Cleavage	1
trf_6_161	Bra016584	3	9.9	1	20	1045	1064	GUCGUUGUAGUAUAGUGGUA	UACUAUUAUACUACAAAGGC	Cleavage	1
trf_6_161	Bra035409	3	10.4	1	20	1328	1347	GUCGUUGUAGUAUAGUGGUA	UACCACAAUACUUCAACGAG	Cleavage	1
trf_6_162	Bra016584	3	9.9	1	20	1045	1064	GUCGUUGUAGUAUAGUGGUA	UACUAUUAUACUACAAAGGC	Cleavage	1
trf_6_162	Bra035409	3	10.4	1	20	1328	1347	GUCGUUGUAGUAUAGUGGUA	UACCACAAUACUUCAACGAG	Cleavage	1
trf_6_163	Bra016584	3	9.9	1	20	1045	1064	GUCGUUGUAGUAUAGUGGUA	UACUAUUAUACUACAAAGGC	Cleavage	1
trf_6_163	Bra035409	3	10.4	1	20	1328	1347	GUCGUUGUAGUAUAGUGGUA	UACCACAAUACUUCAACGAG	Cleavage	1
trf_6_164	Bra016584	3	9.9	1	20	1045	1064	GUCGUUGUAGUAUAGUGGUA	UACUAUUAUACUACAAAGGC	Cleavage	1
trf_6_164	Bra035409	3	10.4	1	20	1328	1347	GUCGUUGUAGUAUAGUGGUA	UACCACAAUACUUCAACGAG	Cleavage	1
trf_6_165	Bra016584	3	9.9	1	20	1045	1064	GUCGUUGUAGUAUAGUGGUA	UACUAUUAUACUACAAAGGC	Cleavage	1
trf_6_165	Bra035409	3	10.4	1	20	1328	1347	GUCGUUGUAGUAUAGUGGUA	UACCACAAUACUUCAACGAG	Cleavage	1

trf_6_1	Bra016		9.					GUCGUUGUAGUAUAG	UACUAUUAUACUACAA	Cleava	
66	584	3	9	1	20	1045	1064	UGGUA	AGGC	ge	1
trf_6_1	Bra035		10					GUCGUUGUAGUAUAG	UACCACAAUACUUCAA	Cleava	
66	409	3	.4	1	20	1328	1347	UGGUA	CGAG	ge	1
trf_6_1	Bra016		9.					GUCGUUGUAGUAUAG	UACUAUUAUACUACAA	Cleava	
67	584	3	9	1	20	1045	1064	UGGUA	AGGC	ge	1
trf_6_1	Bra035		10					GUCGUUGUAGUAUAG	UACCACAAUACUUCAA	Cleava	
67	409	3	.4	1	20	1328	1347	UGGUA	CGAG	ge	1
trf_6_1	Bra016		9.					GUCGUUGUAGUAUAG	UACUAUUAUACUACAA	Cleava	
68	584	3	9	1	20	1045	1064	UGGUA	AGGC	ge	1
trf_6_1	Bra035		10					GUCGUUGUAGUAUAG	UACCACAAUACUUCAA	Cleava	
68	409	3	.4	1	20	1328	1347	UGGUA	CGAG	ge	1
trf_6_1	Bra009		14					GGGGAUGUAGCUCAGA	UUAUCAUCUGGGCUUC	Cleava	
69	876	3	.9	1	22	16	37	UGGUAG	GUCCUC	ge	1
trf_6_1	Bra016		10					GGGGAUGUAGCUCAGA	UUAUCAUCUGAGCUUC	Cleava	
69	141	3	.4	1	22	3031	3052	UGGUAG	GUUCUC	ge	1
trf_6_1	Bra036		20					GGGGAUGUAGCUCAGA	UCGACUGGCUGAGCUA	Cleava	
69	771	3	.2	1	23	5375	5397	UGGUAGA	CAUUCCU	ge	1
trf_6_1	Bra018		8.					GGGGAUGUAGCUCAGA	GCCACCUCAGCUACAUC	Cleava	
69	620	3	3	1	20	7	26	UGGU	CUC	ge	1
trf_6_1	Bra013		22					UCCGUUGUAGUCUAGC	CAUCAUCAGCUUGGCU	Cleava	
70	528	3	.0	1	24	196	219	UGGUCAGG	AUAACGGG	ge	1
trf_6_1	Bra007		14					GCACCAGUGGUCUAGU	UACUACUGGACCUUUG	Cleava	
71	154	2.5	.1	1	20	511	530	GGUA	GUGC	ge	1
trf_6_1	Bra007		14					GCACCAGUGGUCUAGU	UACUACUGGACCUUUG	Cleava	
75	154	2.5	.1	1	20	511	530	GGUA	GUGC	ge	1
trf_6_1	Bra005		12					GUGGCUGUAGUUUAG	CUCACCGCUGAACUAU	Cleava	
85	511	2.5	.8	1	22	1113	1134	UGGUGAG	GGCUAC	ge	1
trf_6_1	Bra035		17					GUGGCUGUAGUUUAG	UAUUCAUCUCUAGGCU	Cleava	
85	454	3	.9	1	24	1241	1264	UGGUGAGAA	ACAGCCAU	ge	1
trf_6_1	Bra009		12					GUGGCUGUAGUUUAG	CAACAUUGAGCUACAG	Cleava	
85	047	3	.1	1	20	243	262	UGGUG	CCAU	ge	1
trf_6_1	Bra026		21					GCCGAUGUCGUCCAGC	GUCGAAACCGUGGGGU	Cleava	
89	941	3	.9	1	25	3	27	GGUUAGGAU	GAUAUCGGC	ge	1

trf_6_189	Bra014566	2.5	21.7	1	22	538	558	GCCGAUGUCGUCCAGCGGUUAG	CCAACCGCUG-ACGACAUUGGC	Cleavage	1
trf_6_190	Bra016584	3	9.9	1	20	1045	1064	GUCGUUGUAGUAUAGUGGUA	UACUAUUAUACUACAAAGGC	Cleavage	1
trf_6_190	Bra035409	3	10.4	1	20	1328	1347	GUCGUUGUAGUAUAGUGGUA	UACCACAAUACUUCAACGAG	Cleavage	1
trf_6_191	Bra016584	3	9.9	1	20	1045	1064	GUCGUUGUAGUAUAGUGGUA	UACUAUUAUACUACAAAGGC	Cleavage	1
trf_6_191	Bra035409	3	10.4	1	20	1328	1347	GUCGUUGUAGUAUAGUGGUA	UACCACAAUACUUCAACGAG	Cleavage	1
trf_6_192	Bra016584	3	9.9	1	20	1045	1064	GUCGUUGUAGUAUAGUGGUA	UACUAUUAUACUACAAAGGC	Cleavage	1
trf_6_192	Bra035409	3	10.4	1	20	1328	1347	GUCGUUGUAGUAUAGUGGUA	UACCACAAUACUUCAACGAG	Cleavage	1
trf_6_193	Bra016584	3	9.9	1	20	1045	1064	GUCGUUGUAGUAUAGUGGUA	UACUAUUAUACUACAAAGGC	Cleavage	1
trf_6_193	Bra035409	3	10.4	1	20	1328	1347	GUCGUUGUAGUAUAGUGGUA	UACCACAAUACUUCAACGAG	Cleavage	1
trf_6_195	Bra007154	2.5	14.1	1	20	511	530	GCACCAGUGGUCUAGUGGUA	UACUACUGGACCUUUGGUGC	Cleavage	1
trf_6_196	Bra009876	3	14.9	1	22	16	37	GGGGAUGUAGCUCAGAUGGUAG	UUAUCAUCUGGGCUUCGUCCUC	Cleavage	1
trf_6_196	Bra016141	3	10.4	1	22	3031	3052	GGGGAUGUAGCUCAGAUGGUAG	UUAUCAUCUGAGCUUCGUUCUC	Cleavage	1
trf_6_196	Bra036771	3	20.2	1	23	5375	5397	GGGGAUGUAGCUCAGAUGGUAGA	UCGACUGGCUGAGCUACAUUCCU	Cleavage	1
trf_6_196	Bra018620	3	8.3	1	20	7	26	GGGGAUGUAGCUCAGAUGGU	GCCACCUCAGCUACAUCCUC	Cleavage	1
trf_6_197	Bra007154	2.5	14.1	1	20	511	530	GCACCAGUGGUCUAGUGGUA	UACUACUGGACCUUUGGUGC	Cleavage	1
trf_6_199	Bra013528	3	22.0	1	24	196	219	UCCGUUGUAGUCUAGCUGGUCAGG	CAUCAUCAGCUUGGCUAUAACGGG	Cleavage	1
trf_6_200	Bra007154	2.5	14.1	1	20	511	530	GCACCAGUGGUCUAGUGGUA	UACUACUGGACCUUUGGUGC	Cleavage	1

trf_6_2	Bra007		14					GCACCAGUGGUCUAGU	UUACUACUGGACCUUU	Cleava	
02	154	2.5	.1	1	21	510	530	GGUAG	GGUGC	ge	1
trf_6_2	Bra036		20					GGGGAUGUAGCUCAGA	CUCGACUGGCUGAGCU	Cleava	
03	771	3	.2	1	24	5374	5397	UGGUAGAG	ACAUUCCU	ge	1
trf_6_2	Bra009		14					GGGGAUGUAGCUCAGA	CGUUAUCAUCUGGGCU	Cleava	
03	876	3	.9	1	24	14	37	UGGUAGAG	UCGUCCUC	ge	1
trf_6_2	Bra016		10					GGGGAUGUAGCUCAGA	CAUUAUCAUCUGAGCU	Cleava	
03	141	3	.4	1	24	3029	3052	UGGUAGAG	UCGUUCUC	ge	1
trf_6_2	Bra018		8.					GGGGAUGUAGCUCAGA	GCCACCUCAGCUACAUC	Cleava	
03	620	3	3	1	20	7	26	UGGU	CUC	ge	1
trf_6_2	Bra009		14					GGGGAUGUAGCUCAGA	UUAUCAUCUGGGCUUC	Cleava	
04	876	3	.9	1	22	16	37	UGGUAG	GUCCUC	ge	1
trf_6_2	Bra016		10					GGGGAUGUAGCUCAGA	UUAUCAUCUGAGCUUC	Cleava	
04	141	3	.4	1	22	3031	3052	UGGUAG	GUUCUC	ge	1
trf_6_2	Bra036		20					GGGGAUGUAGCUCAGA	UCGACUGGCUGAGCUA	Cleava	
04	771	3	.2	1	23	5375	5397	UGGUAGA	CAUUCCU	ge	1
trf_6_2	Bra018		8.					GGGGAUGUAGCUCAGA	GCCACCUCAGCUACAUC	Cleava	
04	620	3	3	1	20	7	26	UGGU	CUC	ge	1
trf_6_2	Bra009		14					GGGGAUGUAGCUCAGA	UUAUCAUCUGGGCUUC	Cleava	
12	876	3	.9	1	22	16	37	UGGUAG	GUCCUC	ge	1
trf_6_2	Bra016		10					GGGGAUGUAGCUCAGA	UUAUCAUCUGAGCUUC	Cleava	
12	141	3	.4	1	22	3031	3052	UGGUAG	GUUCUC	ge	1
trf_6_2	Bra036		20					GGGGAUGUAGCUCAGA	UCGACUGGCUGAGCUA	Cleava	
12	771	3	.2	1	23	5375	5397	UGGUAGA	CAUUCCU	ge	1
trf_6_2	Bra018		8.					GGGGAUGUAGCUCAGA	GCCACCUCAGCUACAUC	Cleava	
12	620	3	3	1	20	7	26	UGGU	CUC	ge	1
trf_6_2	Bra016		9.					GUCGUUGUAGUAUAG	UACUAUUAUACUACAA	Cleava	
13	584	3	9	1	20	1045	1064	UGGUA	AGGC	ge	1
trf_6_2	Bra035		10					GUCGUUGUAGUAUAG	UACCACAAUACUUCAA	Cleava	
13	409	3	.4	1	20	1328	1347	UGGUA	CGAG	ge	1
trf_6_2	Bra016		9.					GUCGUUGUAGUAUAG	UACUAUUAUACUACAA	Cleava	
14	584	3	9	1	20	1045	1064	UGGUA	AGGC	ge	1
trf_6_2	Bra035		10					GUCGUUGUAGUAUAG	UACCACAAUACUUCAA	Cleava	
14	409	3	.4	1	20	1328	1347	UGGUA	CGAG	ge	1

trf_6_2	Bra016		9.					GUCGUUGUAGUAUAG	UACUAUUAUACUACAA	Cleava	
15	584	3	9	1	20	1045	1064	UGGUA	AGGC	ge	1
trf_6_2	Bra035		10					GUCGUUGUAGUAUAG	UACCACAAUACUUCAA	Cleava	
15	409	3	.4	1	20	1328	1347	UGGUA	CGAG	ge	1
trf_6_2	Bra002		16					AGGGAUAUAACUCAGC	CUGCUGCUGAGUUCUA	Cleava	
23	746	3	.3	1	21	218	238	GGUAG	UCCCA	ge	1
trf_6_2	Bra012		14					AGGGAUAUAACUCAGC	UACCUUUGAGUUAUG	Cleava	
23	734	3	.1	1	20	1709	1728	GGUA	UUUCU	ge	1
trf_6_2	Bra016		9.					GUCGUUGUAGUAUAG	UACUAUUAUACUACAA	Cleava	
24	584	3	9	1	20	1045	1064	UGGUA	AGGC	ge	1
trf_6_2	Bra035		10					GUCGUUGUAGUAUAG	UACCACAAUACUUCAA	Cleava	
24	409	3	.4	1	20	1328	1347	UGGUA	CGAG	ge	1
trf_6_2	Bra016		9.					GUCGUUGUAGUAUAG	UACUAUUAUACUACAA	Cleava	
25	584	3	9	1	20	1045	1064	UGGUA	AGGC	ge	1
trf_6_2	Bra035		10					GUCGUUGUAGUAUAG	UACCACAAUACUUCAA	Cleava	
25	409	3	.4	1	20	1328	1347	UGGUA	CGAG	ge	1
trf_6_2	Bra016		9.					GUCGUUGUAGUAUAG	UACUAUUAUACUACAA	Cleava	
26	584	3	9	1	20	1045	1064	UGGUA	AGGC	ge	1
trf_6_2	Bra035		10					GUCGUUGUAGUAUAG	UACCACAAUACUUCAA	Cleava	
26	409	3	.4	1	20	1328	1347	UGGUA	CGAG	ge	1
trf_6_2	Bra016		9.					GUCGUUGUAGUAUAG	UACUAUUAUACUACAA	Cleava	
27	584	3	9	1	20	1045	1064	UGGUA	AGGC	ge	1
trf_6_2	Bra035		10					GUCGUUGUAGUAUAG	UACCACAAUACUUCAA	Cleava	
27	409	3	.4	1	20	1328	1347	UGGUA	CGAG	ge	1
trf_6_2	Bra007		14					GCACCAGUGGUCUAGU	UACUACUGGACCUUUG	Cleava	
28	154	2.5	.1	1	20	511	530	GGUA	GUGC	ge	1
trf_6_2	Bra013		22					UCCGUUGUAGUCUAGC	CAUCAUCAGCUUGGCU	Cleava	
30	528	3	.0	1	24	196	219	UGGUCAGG	AUAACGGG	ge	1
trf_6_2	Bra016		9.					GUCGUUGUAGUAUAG	UACUAUUAUACUACAA	Cleava	
39	584	3	9	1	20	1045	1064	UGGUA	AGGC	ge	1
trf_6_2	Bra035		10					GUCGUUGUAGUAUAG	UACCACAAUACUUCAA	Cleava	
39	409	3	.4	1	20	1328	1347	UGGUA	CGAG	ge	1
trf_6_2	Bra016		9.					GUCGUUGUAGUAUAG	UACUAUUAUACUACAA	Cleava	
40	584	3	9	1	20	1045	1064	UGGUA	AGGC	ge	1

trf_6_2	Bra035		10					GUCGUUGUAGUAUAG	UACCACAAUACUUCAA	Cleava	
40	409	3	.4	1	20	1328	1347	UGGUA	CGAG	ge	1
trf_6_2	Bra016		9.					GUCGUUGUAGUAUAG	UACUAUUAUACUACAA	Cleava	
41	584	3	9	1	20	1045	1064	UGGUA	AGGC	ge	1
trf_6_2	Bra035		10					GUCGUUGUAGUAUAG	UACCACAAUACUUCAA	Cleava	
41	409	3	.4	1	20	1328	1347	UGGUA	CGAG	ge	1
trf_6_2	Bra016		9.					GUCGUUGUAGUAUAG	UACUAUUAUACUACAA	Cleava	
42	584	3	9	1	20	1045	1064	UGGUA	AGGC	ge	1
trf_6_2	Bra035		10					GUCGUUGUAGUAUAG	UACCACAAUACUUCAA	Cleava	
42	409	3	.4	1	20	1328	1347	UGGUA	CGAG	ge	1
trf_6_2	Bra016		9.					GUCGUUGUAGUAUAG	UACUAUUAUACUACAA	Cleava	
43	584	3	9	1	20	1045	1064	UGGUA	AGGC	ge	1
trf_6_2	Bra035		10					GUCGUUGUAGUAUAG	UACCACAAUACUUCAA	Cleava	
43	409	3	.4	1	20	1328	1347	UGGUA	CGAG	ge	1
trf_6_2	Bra000		11					UCCGUUAUCGUCCAGC	UAACCGGAGGACGAUA	Cleava	
45	529	2.5	.2	1	21	984	1004	GGUUA	AUGGA	ge	1
trf_6_2	Bra032		22					UCCGUUAUCGUCCAGC	GACGGCUUGACGAUGA	Cleava	
45	111	3	.4	1	20	727	746	GGUU	CGGA	ge	1
trf_6_2	Bra000		11					UCCGUUAUCGUCCAGC	AACCGGAGGACGAUAA	Cleava	
46	529	2.5	.2	1	20	985	1004	GGUU	UGGA	ge	1
trf_6_2	Bra032		22					UCCGUUAUCGUCCAGC	GACGGCUUGACGAUGA	Cleava	
46	111	3	.4	1	20	727	746	GGUU	CGGA	ge	1
trf_6_2	Bra000		11					UCCGUUAUCGUCCAGC	UAACCGGAGGACGAUA	Cleava	
47	529	2.5	.2	1	21	984	1004	GGUUA	AUGGA	ge	1
trf_6_2	Bra032		22					UCCGUUAUCGUCCAGC	GACGGCUUGACGAUGA	Cleava	
47	111	3	.4	1	20	727	746	GGUU	CGGA	ge	1
trf_6_2	Bra000		11					UCCGUUAUCGUCCAGC	UAACCGGAGGACGAUA	Cleava	
48	529	2.5	.2	1	21	984	1004	GGUUA	AUGGA	ge	1
trf_6_2	Bra032		22					UCCGUUAUCGUCCAGC	GACGGCUUGACGAUGA	Cleava	
48	111	3	.4	1	20	727	746	GGUU	CGGA	ge	1
trf_6_2	Bra016		9.					GUCGUUGUAGUAUAG	UACUAUUAUACUACAA	Cleava	
49	584	3	9	1	20	1045	1064	UGGUA	AGGC	ge	1
trf_6_2	Bra035		10					GUCGUUGUAGUAUAG	UACCACAAUACUUCAA	Cleava	
49	409	3	.4	1	20	1328	1347	UGGUA	CGAG	ge	1

trf_6_250	Bra016584	3	9.9	1	20	1045	1064	GUCGUUGUAGUAUAGUGGUA	UACUAUUAUACUACAAAGGC	Cleavage	1
trf_6_250	Bra035409	3	10.4	1	20	1328	1347	GUCGUUGUAGUAUAGUGGUA	UACCACAAUACUUCAACGAG	Cleavage	1
trf_6_251	Bra016584	3	9.9	1	20	1045	1064	GUCGUUGUAGUAUAGUGGUA	UACUAUUAUACUACAAAGGC	Cleavage	1
trf_6_251	Bra035409	3	10.4	1	20	1328	1347	GUCGUUGUAGUAUAGUGGUA	UACCACAAUACUUCAACGAG	Cleavage	1
trf_6_252	Bra016584	3	9.9	1	20	1045	1064	GUCGUUGUAGUAUAGUGGUA	UACUAUUAUACUACAAAGGC	Cleavage	1
trf_6_252	Bra035409	3	10.4	1	20	1328	1347	GUCGUUGUAGUAUAGUGGUA	UACCACAAUACUUCAACGAG	Cleavage	1
trf_6_253	Bra009876	3	14.9	1	22	16	37	GGGGAUGUAGCUCAGAUGGUAG	UUAUCAUCUGGGCUUCGUCCUC	Cleavage	1
trf_6_253	Bra016141	3	10.4	1	22	3031	3052	GGGGAUGUAGCUCAGAUGGUAG	UUAUCAUCUGAGCUUCGUUCUC	Cleavage	1
trf_6_253	Bra036771	3	20.2	1	23	5375	5397	GGGGAUGUAGCUCAGAUGGUAGA	UCGACUGGCUGAGCUACAUUCCU	Cleavage	1
trf_6_253	Bra018620	3	8.3	1	20	7	26	GGGGAUGUAGCUCAGAUGGU	GCCACCUCAGCUACAUCCUC	Cleavage	1
trf_6_262	Bra009876	3	14.9	1	22	16	37	GGGGAUGUAGCUCAGAUGGUAG	UUAUCAUCUGGGCUUCGUCCUC	Cleavage	1
trf_6_262	Bra016141	3	10.4	1	22	3031	3052	GGGGAUGUAGCUCAGAUGGUAG	UUAUCAUCUGAGCUUCGUUCUC	Cleavage	1
trf_6_262	Bra036771	3	20.2	1	23	5375	5397	GGGGAUGUAGCUCAGAUGGUAGA	UCGACUGGCUGAGCUACAUUCCU	Cleavage	1
trf_6_262	Bra018620	3	8.3	1	20	7	26	GGGGAUGUAGCUCAGAUGGU	GCCACCUCAGCUACAUCCUC	Cleavage	1
trf_6_265	Bra007154	2.5	14.1	1	20	511	530	GCACCAGUGGUCUAGUGGUA	UACUACUGGACCUUUGGUGC	Cleavage	1
trf_6_266	Bra007154	2.5	14.1	1	20	511	530	GCACCAGUGGUCUAGUGGUA	UACUACUGGACCUUUGGUGC	Cleavage	1
trf_6_268	Bra007154	2.5	14.1	1	21	510	530	GCACCAGUGGUCUAGUGGUAG	UUACUACUGGACCUUUGGUGC	Cleavage	1

trf_6_2	Bra016		9.					GUCGUUGUAGUAUAG	UACUAUUAUACUACAA	Cleava	
78	584	3	9	1	20	1045	1064	UGGUA	AGGC	ge	1
trf_6_2	Bra035		10					GUCGUUGUAGUAUAG	UACCACAAUACUUCAA	Cleava	
78	409	3	.4	1	20	1328	1347	UGGUA	CGAG	ge	1
trf_6_2	Bra016		9.					GUCGUUGUAGUAUAG	UACUAUUAUACUACAA	Cleava	
79	584	3	9	1	20	1045	1064	UGGUA	AGGC	ge	1
trf_6_2	Bra035		10					GUCGUUGUAGUAUAG	UACCACAAUACUUCAA	Cleava	
79	409	3	.4	1	20	1328	1347	UGGUA	CGAG	ge	1
trf_6_2	Bra016		9.					GUCGUUGUAGUAUAG	UACUAUUAUACUACAA	Cleava	
80	584	3	9	1	20	1045	1064	UGGUA	AGGC	ge	1
trf_6_2	Bra035		10					GUCGUUGUAGUAUAG	UACCACAAUACUUCAA	Cleava	
80	409	3	.4	1	20	1328	1347	UGGUA	CGAG	ge	1
trf_6_2	Bra016		9.					GUCGUUGUAGUAUAG	UACUAUUAUACUACAA	Cleava	
81	584	3	9	1	20	1045	1064	UGGUA	AGGC	ge	1
trf_6_2	Bra035		10					GUCGUUGUAGUAUAG	UACCACAAUACUUCAA	Cleava	
81	409	3	.4	1	20	1328	1347	UGGUA	CGAG	ge	1
trf_6_2	Bra016		9.					GUCGUUGUAGUAUAG	UACUAUUAUACUACAA	Cleava	
82	584	3	9	1	20	1045	1064	UGGUA	AGGC	ge	1
trf_6_2	Bra035		10					GUCGUUGUAGUAUAG	UACCACAAUACUUCAA	Cleava	
82	409	3	.4	1	20	1328	1347	UGGUA	CGAG	ge	1
trf_6_2	Bra013		16					UCCGUUGUCGUCCAGC	UAUGAUAACCGAUGGA	Cleava	
84	584	3	.4	1	26	885	910	GGUUAGGAUA	UGUCAAUGGA	ge	1
trf_6_2	Bra000		11					UCCGUUGUCGUCCAGC	UAACCGGAGGACGAUA	Cleava	
84	529	3	.2	1	21	984	1004	GGUUA	AUGGA	ge	1
trf_6_2	Bra013		16					UCCGUUGUCGUCCAGC	AACCGAUGGAUGUCAA	Cleava	
85	584	3	.4	1	20	891	910	GGUU	UGGA	ge	1
trf_6_2	Bra000		11					UCCGUUGUCGUCCAGC	AACCGGAGGACGAUAA	Cleava	
85	529	3	.2	1	20	985	1004	GGUU	UGGA	ge	1
trf_6_2	Bra013		16					UCCGUUGUCGUCCAGC	UAACCGAUGGAUGUCA	Cleava	
86	584	3	.4	1	21	890	910	GGUUA	AUGGA	ge	1
trf_6_2	Bra000		11					UCCGUUGUCGUCCAGC	UAACCGGAGGACGAUA	Cleava	
86	529	3	.2	1	21	984	1004	GGUUA	AUGGA	ge	1
trf_6_2	Bra013		16					UCCGUUGUCGUCCAGC	AUGAUAACCGAUGGAU	Cleava	
87	584	3	.4	1	25	886	910	GGUUAGGAU	GUCAAUGGA	ge	1

trf_6_287	Bra000529	3	11.2	1	21	984	1004	UCCGUUGUCGUCCAGCGGUUA	UAACCGGAGGACGAUAAUGGA	Cleavage	1
trf_6_288	Bra013584	3	16.4	1	26	885	910	UCCGUUGUCGUCCAGCGGUUAGGAUA	UAUGAUAACCGAUGGAUGUCAAUGGA	Cleavage	1
trf_6_288	Bra000529	3	11.2	1	21	984	1004	UCCGUUGUCGUCCAGCGGUUA	UAACCGGAGGACGAUAAUGGA	Cleavage	1
trf_6_289	Bra007154	2.5	14.1	1	20	511	530	GCACCAGUGGUCUAGUGGUA	UACUACUGGACCUUUGGUGC	Cleavage	1
trf_6_291	Bra007154	2.5	14.1	1	20	511	530	GCACCAGUGGUCUAGUGGUA	UACUACUGGACCUUUGGUGC	Cleavage	1
trf_6_293	Bra009876	3	14.9	1	22	16	37	GGGGAUGUAGCUCAGAUGGUAG	UUAUCAUCUGGGCUUCGUCCUC	Cleavage	1
trf_6_293	Bra016141	3	10.4	1	22	3031	3052	GGGGAUGUAGCUCAGAUGGUAG	UUAUCAUCUGAGCUUCGUUCUC	Cleavage	1
trf_6_293	Bra036771	3	20.2	1	23	5375	5397	GGGGAUGUAGCUCAGAUGGUAGA	UCGACUGGCUGAGCUACAUUCCU	Cleavage	1
trf_6_293	Bra018620	3	8.3	1	20	7	26	GGGGAUGUAGCUCAGAUGGU	GCCACCUCAGCUACAUCCUC	Cleavage	1
trf_6_294	Bra016584	3	9.9	1	20	1045	1064	GUCGUUGUAGUAUAGUGGUA	UACUAUUAUACUACAAAGGC	Cleavage	1
trf_6_294	Bra035409	3	10.4	1	20	1328	1347	GUCGUUGUAGUAUAGUGGUA	UACCACAAUACUUCAACGAG	Cleavage	1
trf_6_295	Bra016584	3	9.9	1	20	1045	1064	GUCGUUGUAGUAUAGUGGUA	UACUAUUAUACUACAAAGGC	Cleavage	1
trf_6_295	Bra035409	3	10.4	1	20	1328	1347	GUCGUUGUAGUAUAGUGGUA	UACCACAAUACUUCAACGAG	Cleavage	1
trf_6_296	Bra016584	3	9.9	1	20	1045	1064	GUCGUUGUAGUAUAGUGGUA	UACUAUUAUACUACAAAGGC	Cleavage	1
trf_6_296	Bra035409	3	10.4	1	20	1328	1347	GUCGUUGUAGUAUAGUGGUA	UACCACAAUACUUCAACGAG	Cleavage	1
trf_6_299	Bra007154	2.5	14.1	1	20	511	530	GCACCAGUGGUCUAGUGGUA	UACUACUGGACCUUUGGUGC	Cleavage	1
trf_6_301	Bra016584	3	9.9	1	20	1045	1064	GUCGUUGUAGUAUAGUGGUA	UACUAUUAUACUACAAAGGC	Cleavage	1

trf_6_301	Bra035409	3	10.4	1	20	1328	1347	GUCGUUGUAGUAUAGUGGUA	UACCACAAUACUUCAACGAG	Cleavage	1
trf_6_302	Bra016584	3	9.9	1	20	1045	1064	GUCGUUGUAGUAUAGUGGUA	UACUAUUAUACUACAAAGGC	Cleavage	1
trf_6_302	Bra035409	3	10.4	1	20	1328	1347	GUCGUUGUAGUAUAGUGGUA	UACCACAAUACUUCAACGAG	Cleavage	1
trf_6_303	Bra016584	3	9.9	1	20	1045	1064	GUCGUUGUAGUAUAGUGGUA	UACUAUUAUACUACAAAGGC	Cleavage	1
trf_6_303	Bra035409	3	10.4	1	20	1328	1347	GUCGUUGUAGUAUAGUGGUA	UACCACAAUACUUCAACGAG	Cleavage	1
trf_6_304	Bra016584	3	9.9	1	20	1045	1064	GUCGUUGUAGUAUAGUGGUA	UACUAUUAUACUACAAAGGC	Cleavage	1
trf_6_304	Bra035409	3	10.4	1	20	1328	1347	GUCGUUGUAGUAUAGUGGUA	UACCACAAUACUUCAACGAG	Cleavage	1
trf_6_305	Bra013528	3	22.0	1	24	196	219	UCCGUUGUAGUCUAGCUGGUCAGG	CAUCAUCAGCUUGGCUAUAACGGG	Cleavage	1
trf_6_306	Bra016584	3	9.9	1	20	1045	1064	GUCGUUGUAGUAUAGUGGUA	UACUAUUAUACUACAAAGGC	Cleavage	1
trf_6_306	Bra035409	3	10.4	1	20	1328	1347	GUCGUUGUAGUAUAGUGGUA	UACCACAAUACUUCAACGAG	Cleavage	1
trf_6_307	Bra016584	3	9.9	1	20	1045	1064	GUCGUUGUAGUAUAGUGGUA	UACUAUUAUACUACAAAGGC	Cleavage	1
trf_6_307	Bra035409	3	10.4	1	20	1328	1347	GUCGUUGUAGUAUAGUGGUA	UACCACAAUACUUCAACGAG	Cleavage	1
trf_6_308	Bra016584	3	9.9	1	20	1045	1064	GUCGUUGUAGUAUAGUGGUA	UACUAUUAUACUACAAAGGC	Cleavage	1
trf_6_308	Bra035409	3	10.4	1	20	1328	1347	GUCGUUGUAGUAUAGUGGUA	UACCACAAUACUUCAACGAG	Cleavage	1
trf_6_309	Bra016584	3	9.9	1	20	1045	1064	GUCGUUGUAGUAUAGUGGUA	UACUAUUAUACUACAAAGGC	Cleavage	1
trf_6_309	Bra035409	3	10.4	1	20	1328	1347	GUCGUUGUAGUAUAGUGGUA	UACCACAAUACUUCAACGAG	Cleavage	1
trf_6_311	Bra007154	2.5	14.1	1	20	511	530	GCACCAGUGGUCUAGUGGUA	UACUACUGGACCUUUGGUGC	Cleavage	1

trf_6_312	Bra009876	3	14.9	1	22	16	37	GGGGAUGUAGCUCAGAUGGUAG	UUAUCAUCUGGGCUUCGUCCUC	Cleavage	1
trf_6_312	Bra016141	3	10.4	1	22	3031	3052	GGGGAUGUAGCUCAGAUGGUAG	UUAUCAUCUGAGCUUCGUUCUC	Cleavage	1
trf_6_312	Bra036771	3	20.2	1	23	5375	5397	GGGGAUGUAGCUCAGAUGGUAGA	UCGACUGGCUGAGCUACAUUCCU	Cleavage	1
trf_6_312	Bra018620	3	8.3	1	20	7	26	GGGGAUGUAGCUCAGAUGGU	GCCACCUCAGCUACAUCCUC	Cleavage	1
trf_6_313	Bra013584	3	16.4	1	21	890	910	UCCGUUGUCGUCCAGCGGUUA	UAACCGAUGGAUGUCAAUGGA	Cleavage	1
trf_6_313	Bra000529	3	11.2	1	21	984	1004	UCCGUUGUCGUCCAGCGGUUA	UAACCGGAGGACGAUAAUGGA	Cleavage	1
trf_6_314	Bra013584	3	16.4	1	25	886	910	UCCGUUGUCGUCCAGCGGUUAGGAU	AUGAUAACCGAUGGAUGUCAAUGGA	Cleavage	1
trf_6_314	Bra000529	3	11.2	1	21	984	1004	UCCGUUGUCGUCCAGCGGUUA	UAACCGGAGGACGAUAAUGGA	Cleavage	1
trf_6_315	Bra013584	3	16.4	1	21	890	910	UCCGUUGUCGUCCAGCGGUUA	UAACCGAUGGAUGUCAAUGGA	Cleavage	1
trf_6_315	Bra000529	3	11.2	1	21	984	1004	UCCGUUGUCGUCCAGCGGUUA	UAACCGGAGGACGAUAAUGGA	Cleavage	1
trf_6_316	Bra013584	3	16.4	1	26	885	910	UCCGUUGUCGUCCAGCGGUUAGGAUA	UAUGAUAACCGAUGGAUGUCAAUGGA	Cleavage	1
trf_6_316	Bra000529	3	11.2	1	21	984	1004	UCCGUUGUCGUCCAGCGGUUA	UAACCGGAGGACGAUAAUGGA	Cleavage	1
trf_6_317	Bra013584	3	16.4	1	21	890	910	UCCGUUGUCGUCCAGCGGUUA	UAACCGAUGGAUGUCAAUGGA	Cleavage	1
trf_6_317	Bra000529	3	11.2	1	21	984	1004	UCCGUUGUCGUCCAGCGGUUA	UAACCGGAGGACGAUAAUGGA	Cleavage	1
trf_6_318	Bra013584	3	16.4	1	26	885	910	UCCGUUGUCGUCCAGCGGUUAGGAUA	UAUGAUAACCGAUGGAUGUCAAUGGA	Cleavage	1
trf_6_318	Bra000529	3	11.2	1	21	984	1004	UCCGUUGUCGUCCAGCGGUUA	UAACCGGAGGACGAUAAUGGA	Cleavage	1
trf_6_320	Bra013584	3	16.4	1	20	891	910	UCCGUUGUCGUCCAGCGGUU	AACCGAUGGAUGUCAAUGGA	Cleavage	1

trf_6_3	Bra000		11					UCCGUUGUCGUCCAGC	AACCGGAGGACGAUAA	Cleava	
20	529	3	.2	1	20	985	1004	GGUU	UGGA	ge	1
trf_6_3	Bra014		17					GGGGAUGUAGCUCAUA	UGUAUUAUAUUAGCU	Cleava	
21	507	3	.0	1	23	541	563	UGGUAGA	AUAUCUCC	ge	1
trf_6_3	Bra016		20					UCCGUCGUAGUCUAGC	CUGAAGAAGCUAGGCU	Cleava	
22	601	3	.8	1	24	415	438	UGGUUAGG	GCGACGGA	ge	1
trf_6_3	Bra016		20					UCCGUCGUAGUCUAGC	CUGAAGAAGCUAGGCU	Cleava	
23	601	3	.8	1	24	415	438	UGGUUAGG	GCGACGGA	ge	1
trf_6_3	Bra016		20					UCCGUCGUAGUCUAGC	AAGAAGCUAGGCUGCG	Cleava	
24	601	3	.8	1	21	418	438	UGGUU	ACGGA	ge	1
trf_6_3	Bra016		20					UCCGUCGUAGUCUAGC	CUGAAGAAGCUAGGCU	Cleava	
25	601	3	.8	1	24	415	438	UGGUUAGG	GCGACGGA	ge	1
trf_6_3	Bra009		14					GGGGAUGUAGCUCAGA	UUAUCAUCUGGGCUUC	Cleava	
28	876	3	.9	1	22	16	37	UGGUAG	GUCCUC	ge	1
trf_6_3	Bra016		10					GGGGAUGUAGCUCAGA	UUAUCAUCUGAGCUUC	Cleava	
28	141	3	.4	1	22	3031	3052	UGGUAG	GUUCUC	ge	1
trf_6_3	Bra036		20					GGGGAUGUAGCUCAGA	UCGACUGGCUGAGCUA	Cleava	
28	771	3	.2	1	23	5375	5397	UGGUAGA	CAUUCCU	ge	1
trf_6_3	Bra018		8.					GGGGAUGUAGCUCAGA	GCCACCUCAGCUACAUC	Cleava	
28	620	3	3	1	20	7	26	UGGU	CUC	ge	1
trf_6_3	Bra036		20					GGGGAUGUAGCUCAGA	CUCGACUGGCUGAGCU	Cleava	
29	771	3	.2	1	24	5374	5397	UGGUAGAG	ACAUUCCU	ge	1
trf_6_3	Bra009		14					GGGGAUGUAGCUCAGA	CGUUAUCAUCUGGGCU	Cleava	
29	876	3	.9	1	24	14	37	UGGUAGAG	UCGUCCUC	ge	1
trf_6_3	Bra016		10					GGGGAUGUAGCUCAGA	CAUUAUCAUCUGAGCU	Cleava	
29	141	3	.4	1	24	3029	3052	UGGUAGAG	UCGUUCUC	ge	1
trf_6_3	Bra018		8.					GGGGAUGUAGCUCAGA	GCCACCUCAGCUACAUC	Cleava	
29	620	3	3	1	20	7	26	UGGU	CUC	ge	1
trf_6_3	Bra016		9.					GUCGUUGUAGUAUAG	UACUAUUAUACUACAA	Cleava	
31	584	3	9	1	20	1045	1064	UGGUA	AGGC	ge	1
trf_6_3	Bra035		10					GUCGUUGUAGUAUAG	UACCACAAUACUUCAA	Cleava	
31	409	3	.4	1	20	1328	1347	UGGUA	CGAG	ge	1
trf_6_3	Bra016		9.					GUCGUUGUAGUAUAG	UACUAUUAUACUACAA	Cleava	
32	584	3	9	1	20	1045	1064	UGGUA	AGGC	ge	1

trf_6_3	Bra035		10					GUCGUUGUAGUAUAG	UACCACAAUACUUCAA	Cleava	
32	409	3	.4	1	20	1328	1347	UGGUA	CGAG	ge	1
trf_6_3	Bra016		9.					GUCGUUGUAGUAUAG	UACUAUUAUACUACAA	Cleava	
33	584	3	9	1	20	1045	1064	UGGUA	AGGC	ge	1
trf_6_3	Bra035		10					GUCGUUGUAGUAUAG	UACCACAAUACUUCAA	Cleava	
33	409	3	.4	1	20	1328	1347	UGGUA	CGAG	ge	1
trf_6_3	Bra009		14					GGGGAUGUAGCUCAGA	UUAUCAUCUGGGCUUC	Cleava	
36	876	3	.9	1	22	16	37	UGGUAG	GUCCUC	ge	1
trf_6_3	Bra016		10					GGGGAUGUAGCUCAGA	UUAUCAUCUGAGCUUC	Cleava	
36	141	3	.4	1	22	3031	3052	UGGUAG	GUUCUC	ge	1
trf_6_3	Bra036		20					GGGGAUGUAGCUCAGA	UCGACUGGCUGAGCUA	Cleava	
36	771	3	.2	1	23	5375	5397	UGGUAGA	CAUUCCU	ge	1
trf_6_3	Bra018		8.					GGGGAUGUAGCUCAGA	GCCACCUCAGCUACAUC	Cleava	
36	620	3	3	1	20	7	26	UGGU	CUC	ge	1
trf_6_3	Bra016		9.					GUCGUUGUAGUAUAG	UACUAUUAUACUACAA	Cleava	
40	584	3	9	1	20	1045	1064	UGGUA	AGGC	ge	1
trf_6_3	Bra035		10					GUCGUUGUAGUAUAG	UACCACAAUACUUCAA	Cleava	
40	409	3	.4	1	20	1328	1347	UGGUA	CGAG	ge	1
trf_6_3	Bra016		9.					GUCGUUGUAGUAUAG	UACUAUUAUACUACAA	Cleava	
41	584	3	9	1	20	1045	1064	UGGUA	AGGC	ge	1
trf_6_3	Bra035		10					GUCGUUGUAGUAUAG	UACCACAAUACUUCAA	Cleava	
41	409	3	.4	1	20	1328	1347	UGGUA	CGAG	ge	1
trf_6_3	Bra016		9.					GUCGUUGUAGUAUAG	UACUAUUAUACUACAA	Cleava	
42	584	3	9	1	20	1045	1064	UGGUA	AGGC	ge	1
trf_6_3	Bra035		10					GUCGUUGUAGUAUAG	UACCACAAUACUUCAA	Cleava	
42	409	3	.4	1	20	1328	1347	UGGUA	CGAG	ge	1
trf_6_3	Bra016		9.					GUCGUUGUAGUAUAG	UACUAUUAUACUACAA	Cleava	
43	584	3	9	1	20	1045	1064	UGGUA	AGGC	ge	1
trf_6_3	Bra035		10					GUCGUUGUAGUAUAG	UACCACAAUACUUCAA	Cleava	
43	409	3	.4	1	20	1328	1347	UGGUA	CGAG	ge	1
trf_6_3	Bra009		14					GGGGAUGUAGCUCAGA	UUAUCAUCUGGGCUUC	Cleava	
44	876	3	.9	1	22	16	37	UGGUAG	GUCCUC	ge	1
trf_6_3	Bra016		10					GGGGAUGUAGCUCAGA	UUAUCAUCUGAGCUUC	Cleava	
44	141	3	.4	1	22	3031	3052	UGGUAG	GUUCUC	ge	1

trf_6_3	Bra036		20					GGGGAUGUAGCUCAGA	UCGACUGGCUGAGCUA	Cleava	
44	771	3	.2	1	23	5375	5397	UGGUAGA	CAUUCCU	ge	1
trf_6_3	Bra018		8.					GGGGAUGUAGCUCAGA	GCCACCUCAGCUACAUC	Cleava	
44	620	3	3	1	20	7	26	UGGU	CUC	ge	1
trf_6_3	Bra009		14					GGGGAUGUAGCUCAGA	UUAUCAUCUGGGCUUC	Cleava	
45	876	3	.9	1	22	16	37	UGGUAG	GUCCUC	ge	1
trf_6_3	Bra016		10					GGGGAUGUAGCUCAGA	UUAUCAUCUGAGCUUC	Cleava	
45	141	3	.4	1	22	3031	3052	UGGUAG	GUUCUC	ge	1
trf_6_3	Bra036		20					GGGGAUGUAGCUCAGA	UCGACUGGCUGAGCUA	Cleava	
45	771	3	.2	1	23	5375	5397	UGGUAGA	CAUUCCU	ge	1
trf_6_3	Bra018		8.					GGGGAUGUAGCUCAGA	GCCACCUCAGCUACAUC	Cleava	
45	620	3	3	1	20	7	26	UGGU	CUC	ge	1
trf_6_3	Bra026		23					AUCAGAGUCGCGCAGC	CUUCCUCUUCGCGCCU	Cleava	
50	662	3	.3	1	21	201	221	GGAAG	CUGAU	ge	1
trf_6_3	Bra009		14					GGGGAUGUAGCUCAGA	UUAUCAUCUGGGCUUC	Cleava	
51	876	3	.9	1	22	16	37	UGGUAG	GUCCUC	ge	1
trf_6_3	Bra016		10					GGGGAUGUAGCUCAGA	UUAUCAUCUGAGCUUC	Cleava	
51	141	3	.4	1	22	3031	3052	UGGUAG	GUUCUC	ge	1
trf_6_3	Bra036		20					GGGGAUGUAGCUCAGA	UCGACUGGCUGAGCUA	Cleava	
51	771	3	.2	1	23	5375	5397	UGGUAGA	CAUUCCU	ge	1
trf_6_3	Bra018		8.					GGGGAUGUAGCUCAGA	GCCACCUCAGCUACAUC	Cleava	
51	620	3	3	1	20	7	26	UGGU	CUC	ge	1
trf_6_3	Bra007		14					GCACCAGUGGUCUAGU	UACUACUGGACCUUUG	Cleava	
54	154	2.5	.1	1	20	511	530	GGUA	GUGC	ge	1
trf_6_3	Bra007		14					GCACCAGUGGUCUAGU	UACUACUGGACCUUUG	Cleava	
57	154	2.5	.1	1	20	511	530	GGUA	GUGC	ge	1
trf_6_3	Bra016		9.					GUCGUUGUAGUAUAG	UACUAUUAUACUACAA	Cleava	
58	584	3	9	1	20	1045	1064	UGGUA	AGGC	ge	1
trf_6_3	Bra035		10					GUCGUUGUAGUAUAG	UACCACAAUACUUCAA	Cleava	
58	409	3	.4	1	20	1328	1347	UGGUA	CGAG	ge	1
trf_6_3	Bra016		9.					GUCGUUGUAGUAUAG	UACUAUUAUACUACAA	Cleava	
59	584	3	9	1	20	1045	1064	UGGUA	AGGC	ge	1
trf_6_3	Bra035		10					GUCGUUGUAGUAUAG	UACCACAAUACUUCAA	Cleava	
59	409	3	.4	1	20	1328	1347	UGGUA	CGAG	ge	1

trf_6_360	Bra016584	3	9.9	1	20	1045	1064	GUCGUUGUAGUAUAGUGGUA	UACUAUUAUACUACAAAGGC	Cleavage	1
trf_6_360	Bra035409	3	10.4	1	20	1328	1347	GUCGUUGUAGUAUAGUGGUA	UACCACAAUACUUCAACGAG	Cleavage	1
trf_6_361	Bra016584	3	9.9	1	20	1045	1064	GUCGUUGUAGUAUAGUGGUA	UACUAUUAUACUACAAAGGC	Cleavage	1
trf_6_361	Bra035409	3	10.4	1	20	1328	1347	GUCGUUGUAGUAUAGUGGUA	UACCACAAUACUUCAACGAG	Cleavage	1
trf_6_362	Bra016584	3	9.9	1	20	1045	1064	GUCGUUGUAGUAUAGUGGUA	UACUAUUAUACUACAAAGGC	Cleavage	1
trf_6_362	Bra035409	3	10.4	1	20	1328	1347	GUCGUUGUAGUAUAGUGGUA	UACCACAAUACUUCAACGAG	Cleavage	1
trf_6_363	Bra009876	3	14.9	1	22	16	37	GGGGAUGUAGCUCAGAUGGUAG	UUAUCAUCUGGGCUUCGUCCUC	Cleavage	1
trf_6_363	Bra016141	3	10.4	1	22	3031	3052	GGGGAUGUAGCUCAGAUGGUAG	UUAUCAUCUGAGCUUCGUUCUC	Cleavage	1
trf_6_363	Bra036771	3	20.2	1	23	5375	5397	GGGGAUGUAGCUCAGAUGGUAGA	UCGACUGGCUGAGCUACAUUCCU	Cleavage	1
trf_6_363	Bra018620	3	8.3	1	20	7	26	GGGGAUGUAGCUCAGAUGGU	GCCACCUCAGCUACAUCCUC	Cleavage	1
trf_6_364	Bra024638	3	7.5	1	23	203	225	GGGGAUGUAGCUCAAAUGGUAGA	UCCAACAUUUCGGUUACAUCCCC	Cleavage	1
trf_6_364	Bra025904	3	17.2	1	20	344	363	GGGGAUGUAGCUCAAAUGGU	ACAAUUUGAGCUUCAUCUUC	Cleavage	1
trf_6_366	Bra009876	3	14.9	1	22	16	37	GGGGAUGUAGCUCAGAUGGUAG	UUAUCAUCUGGGCUUCGUCCUC	Cleavage	1
trf_6_366	Bra016141	3	10.4	1	22	3031	3052	GGGGAUGUAGCUCAGAUGGUAG	UUAUCAUCUGAGCUUCGUUCUC	Cleavage	1
trf_6_366	Bra036771	3	20.2	1	23	5375	5397	GGGGAUGUAGCUCAGAUGGUAGA	UCGACUGGCUGAGCUACAUUCCU	Cleavage	1
trf_6_366	Bra018620	3	8.3	1	20	7	26	GGGGAUGUAGCUCAGAUGGU	GCCACCUCAGCUACAUCCUC	Cleavage	1
trf_6_379	Bra009876	3	14.9	1	22	16	37	GGGGAUGUAGCUCAGAUGGUAG	UUAUCAUCUGGGCUUCGUCCUC	Cleavage	1

trf_6_3	Bra016		10					GGGGAUGUAGCUCAGA	UUAUCAUCUGAGCUUC	Cleava	
79	141	3	.4	1	22	3031	3052	UGGUAG	GUUCUC	ge	1
trf_6_3	Bra036		20					GGGGAUGUAGCUCAGA	UCGACUGGCUGAGCUA	Cleava	
79	771	3	.2	1	23	5375	5397	UGGUAGA	CAUUCCU	ge	1
trf_6_3	Bra018		8.					GGGGAUGUAGCUCAGA	GCCACCUCAGCUACAUC	Cleava	
79	620	3	3	1	20	7	26	UGGU	CUC	ge	1
trf_6_3	Bra007		14					GCACCAGUGGUCUAGU	UACUACUGGACCUUUG	Cleava	
83	154	2.5	.1	1	20	511	530	GGUA	GUGC	ge	1
trf_6_3	Bra009		14					GGGGAUGUAGCUCAGA	UUAUCAUCUGGGCUUC	Cleava	
86	876	3	.9	1	22	16	37	UGGUAG	GUCCUC	ge	1
trf_6_3	Bra016		10					GGGGAUGUAGCUCAGA	UUAUCAUCUGAGCUUC	Cleava	
86	141	3	.4	1	22	3031	3052	UGGUAG	GUUCUC	ge	1
trf_6_3	Bra036		20					GGGGAUGUAGCUCAGA	UCGACUGGCUGAGCUA	Cleava	
86	771	3	.2	1	23	5375	5397	UGGUAGA	CAUUCCU	ge	1
trf_6_3	Bra018		8.					GGGGAUGUAGCUCAGA	GCCACCUCAGCUACAUC	Cleava	
86	620	3	3	1	20	7	26	UGGU	CUC	ge	1
trf_6_3	Bra024		7.					GGGGAUGUAGCUCAAA	UCCAACAUUUCGGUUA	Cleava	
87	638	3	5	1	23	203	225	UGGUAGA	CAUCCCC	ge	1
trf_6_3	Bra025		17					GGGGAUGUAGCUCAAA	ACAAUUUGAGCUUCAU	Cleava	
87	904	3	.2	1	20	344	363	UGGU	CUUC	ge	1
trf_6_3	Bra016		9.					GUCGUUGUAGUAUAG	UACUAUUAUACUACAA	Cleava	
89	584	3	9	1	20	1045	1064	UGGUA	AGGC	ge	1
trf_6_3	Bra035		10					GUCGUUGUAGUAUAG	UACCACAAUACUUCAA	Cleava	
89	409	3	.4	1	20	1328	1347	UGGUA	CGAG	ge	1
trf_6_3	Bra016		9.					GUCGUUGUAGUAUAG	UACUAUUAUACUACAA	Cleava	
90	584	3	9	1	20	1045	1064	UGGUA	AGGC	ge	1
trf_6_3	Bra035		10					GUCGUUGUAGUAUAG	UACCACAAUACUUCAA	Cleava	
90	409	3	.4	1	20	1328	1347	UGGUA	CGAG	ge	1
trf_6_3	Bra016		9.					GUCGUUGUAGUAUAG	UACUAUUAUACUACAA	Cleava	
91	584	3	9	1	20	1045	1064	UGGUA	AGGC	ge	1
trf_6_3	Bra035		10					GUCGUUGUAGUAUAG	UACCACAAUACUUCAA	Cleava	
91	409	3	.4	1	20	1328	1347	UGGUA	CGAG	ge	1
trf_6_3	Bra016		9.					GUCGUUGUAGUAUAG	UACUAUUAUACUACAA	Cleava	
92	584	3	9	1	20	1045	1064	UGGUA	AGGC	ge	1

trf_6_392	Bra035409	3	10.4	1	20	1328	1347	GUCGUUGUAGUAUAGUGGUA	UACCACAAUACUUCAACGAG	Cleavage	1
trf_6_393	Bra016584	3	9.9	1	20	1045	1064	GUCGUUGUAGUAUAGUGGUA	UACUAUUAUACUACAAAGGC	Cleavage	1
trf_6_393	Bra035409	3	10.4	1	20	1328	1347	GUCGUUGUAGUAUAGUGGUA	UACCACAAUACUUCAACGAG	Cleavage	1
trf_6_394	Bra016584	3	9.9	1	20	1045	1064	GUCGUUGUAGUAUAGUGGUA	UACUAUUAUACUACAAAGGC	Cleavage	1
trf_6_394	Bra035409	3	10.4	1	20	1328	1347	GUCGUUGUAGUAUAGUGGUA	UACCACAAUACUUCAACGAG	Cleavage	1
trf_6_395	Bra016584	3	9.9	1	20	1045	1064	GUCGUUGUAGUAUAGUGGUA	UACUAUUAUACUACAAAGGC	Cleavage	1
trf_6_395	Bra035409	3	10.4	1	20	1328	1347	GUCGUUGUAGUAUAGUGGUA	UACCACAAUACUUCAACGAG	Cleavage	1
trf_6_396	Bra016584	3	9.9	1	20	1045	1064	GUCGUUGUAGUAUAGUGGUA	UACUAUUAUACUACAAAGGC	Cleavage	1
trf_6_396	Bra035409	3	10.4	1	20	1328	1347	GUCGUUGUAGUAUAGUGGUA	UACCACAAUACUUCAACGAG	Cleavage	1
trf_6_397	Bra016584	3	9.9	1	20	1045	1064	GUCGUUGUAGUAUAGUGGUA	UACUAUUAUACUACAAAGGC	Cleavage	1
trf_6_397	Bra035409	3	10.4	1	20	1328	1347	GUCGUUGUAGUAUAGUGGUA	UACCACAAUACUUCAACGAG	Cleavage	1
trf_6_398	Bra016584	3	9.9	1	20	1045	1064	GUCGUUGUAGUAUAGUGGUA	UACUAUUAUACUACAAAGGC	Cleavage	1
trf_6_398	Bra035409	3	10.4	1	20	1328	1347	GUCGUUGUAGUAUAGUGGUA	UACCACAAUACUUCAACGAG	Cleavage	1
trf_6_399	Bra016584	3	9.9	1	20	1045	1064	GUCGUUGUAGUAUAGUGGUA	UACUAUUAUACUACAAAGGC	Cleavage	1
trf_6_399	Bra035409	3	10.4	1	20	1328	1347	GUCGUUGUAGUAUAGUGGUA	UACCACAAUACUUCAACGAG	Cleavage	1
trf_6_400	Bra016584	3	9.9	1	20	1045	1064	GUCGUUGUAGUAUAGUGGUA	UACUAUUAUACUACAAAGGC	Cleavage	1
trf_6_400	Bra035409	3	10.4	1	20	1328	1347	GUCGUUGUAGUAUAGUGGUA	UACCACAAUACUUCAACGAG	Cleavage	1

trf_6_401	Bra016584	3	9.9	1	20	1045	1064	GUCGUUGUAGUAUAGUGGUA	UACUAUUAUACUACAAAGGC	Cleavage	1
trf_6_401	Bra035409	3	10.4	1	20	1328	1347	GUCGUUGUAGUAUAGUGGUA	UACCACAAUACUUCAACGAG	Cleavage	1
trf_6_402	Bra009876	3	14.9	1	22	16	37	GGGGAUGUAGCUCAGAUGGUAG	UUAUCAUCUGGGCUUCGUCCUC	Cleavage	1
trf_6_402	Bra016141	3	10.4	1	22	3031	3052	GGGGAUGUAGCUCAGAUGGUAG	UUAUCAUCUGAGCUUCGUUCUC	Cleavage	1
trf_6_402	Bra036771	3	20.2	1	23	5375	5397	GGGGAUGUAGCUCAGAUGGUAGA	UCGACUGGCUGAGCUACAUUCCU	Cleavage	1
trf_6_402	Bra018620	3	8.3	1	20	7	26	GGGGAUGUAGCUCAGAUGGU	GCCACCUCAGCUACAUCCUC	Cleavage	1
trf_6_403	Bra009876	3	14.9	1	22	16	37	GGGGAUGUAGCUCAGAUGGUAG	UUAUCAUCUGGGCUUCGUCCUC	Cleavage	1
trf_6_403	Bra016141	3	10.4	1	22	3031	3052	GGGGAUGUAGCUCAGAUGGUAG	UUAUCAUCUGAGCUUCGUUCUC	Cleavage	1
trf_6_403	Bra036771	3	20.2	1	23	5375	5397	GGGGAUGUAGCUCAGAUGGUAGA	UCGACUGGCUGAGCUACAUUCCU	Cleavage	1
trf_6_403	Bra018620	3	8.3	1	20	7	26	GGGGAUGUAGCUCAGAUGGU	GCCACCUCAGCUACAUCCUC	Cleavage	1
trf_6_404	Bra014507	3	17.0	1	23	541	563	GGGGAUGUAGCUCAUAUGGUAGA	UGUAUUAUAUUAGCUAUAUCUCC	Cleavage	1
trf_6_405	Bra016584	3	9.9	1	20	1045	1064	GUCGUUGUAGUAUAGUGGUA	UACUAUUAUACUACAAAGGC	Cleavage	1
trf_6_405	Bra035409	3	10.4	1	20	1328	1347	GUCGUUGUAGUAUAGUGGUA	UACCACAAUACUUCAACGAG	Cleavage	1
trf_6_406	Bra016584	3	9.9	1	20	1045	1064	GUCGUUGUAGUAUAGUGGUA	UACUAUUAUACUACAAAGGC	Cleavage	1
trf_6_406	Bra035409	3	10.4	1	20	1328	1347	GUCGUUGUAGUAUAGUGGUA	UACCACAAUACUUCAACGAG	Cleavage	1
trf_6_407	Bra016584	3	9.9	1	20	1045	1064	GUCGUUGUAGUAUAGUGGUA	UACUAUUAUACUACAAAGGC	Cleavage	1
trf_6_407	Bra035409	3	10.4	1	20	1328	1347	GUCGUUGUAGUAUAGUGGUA	UACCACAAUACUUCAACGAG	Cleavage	1

trf_6_408	Bra016584	3	9.9	1	20	1045	1064	GUCGUUGUAGUAUAGUGGUA	UACUAUUAUACUACAAAGGC	Cleavage	1
trf_6_408	Bra035409	3	10.4	1	20	1328	1347	GUCGUUGUAGUAUAGUGGUA	UACCACAAUACUUCAACGAG	Cleavage	1
trf_6_409	Bra009876	3	14.9	1	22	16	37	GGGGAUGUAGCUCAGAUGGUAG	UUAUCAUCUGGGCUUCGUCCUC	Cleavage	1
trf_6_409	Bra016141	3	10.4	1	22	3031	3052	GGGGAUGUAGCUCAGAUGGUAG	UUAUCAUCUGAGCUUCGUUCUC	Cleavage	1
trf_6_409	Bra036771	3	20.2	1	23	5375	5397	GGGGAUGUAGCUCAGAUGGUAGA	UCGACUGGCUGAGCUACAUUCCU	Cleavage	1
trf_6_409	Bra018620	3	8.3	1	20	7	26	GGGGAUGUAGCUCAGAUGGU	GCCACCUCAGCUACAUCCUC	Cleavage	1
trf_6_410	Bra016584	3	9.9	1	20	1045	1064	GUCGUUGUAGUAUAGUGGUA	UACUAUUAUACUACAAAGGC	Cleavage	1
trf_6_410	Bra035409	3	10.4	1	20	1328	1347	GUCGUUGUAGUAUAGUGGUA	UACCACAAUACUUCAACGAG	Cleavage	1
trf_6_411	Bra016584	3	9.9	1	20	1045	1064	GUCGUUGUAGUAUAGUGGUA	UACUAUUAUACUACAAAGGC	Cleavage	1
trf_6_411	Bra035409	3	10.4	1	20	1328	1347	GUCGUUGUAGUAUAGUGGUA	UACCACAAUACUUCAACGAG	Cleavage	1
trf_6_412	Bra016584	3	9.9	1	20	1045	1064	GUCGUUGUAGUAUAGUGGUA	UACUAUUAUACUACAAAGGC	Cleavage	1
trf_6_412	Bra035409	3	10.4	1	20	1328	1347	GUCGUUGUAGUAUAGUGGUA	UACCACAAUACUUCAACGAG	Cleavage	1
trf_6_413	Bra016584	3	9.9	1	20	1045	1064	GUCGUUGUAGUAUAGUGGUA	UACUAUUAUACUACAAAGGC	Cleavage	1
trf_6_413	Bra035409	3	10.4	1	20	1328	1347	GUCGUUGUAGUAUAGUGGUA	UACCACAAUACUUCAACGAG	Cleavage	1
trf_6_414	Bra016584	3	9.9	1	20	1045	1064	GUCGUUGUAGUAUAGUGGUA	UACUAUUAUACUACAAAGGC	Cleavage	1
trf_6_414	Bra035409	3	10.4	1	20	1328	1347	GUCGUUGUAGUAUAGUGGUA	UACCACAAUACUUCAACGAG	Cleavage	1
trf_6_415	Bra009876	3	14.9	1	22	16	37	GGGGAUGUAGCUCAGAUGGUAG	UUAUCAUCUGGGCUUCGUCCUC	Cleavage	1

trf_6_415	Bra016141	3	10.4	1	22	3031	3052	GGGGAUGUAGCUCAGAUGGUAG	UUAUCAUCUGAGCUUCGUUCUC	Cleavage	1
trf_6_415	Bra036771	3	20.2	1	23	5375	5397	GGGGAUGUAGCUCAGAUGGUAGA	UCGACUGGCUGAGCUACAUUCCU	Cleavage	1
trf_6_415	Bra018620	3	8.3	1	20	7	26	GGGGAUGUAGCUCAGAUGGU	GCCACCUCAGCUACAUCCUC	Cleavage	1
trf_6_416	Bra009876	3	14.9	1	22	16	37	GGGGAUGUAGCUCAGAUGGUAG	UUAUCAUCUGGGCUUCGUCCUC	Cleavage	1
trf_6_416	Bra016141	3	10.4	1	22	3031	3052	GGGGAUGUAGCUCAGAUGGUAG	UUAUCAUCUGAGCUUCGUUCUC	Cleavage	1
trf_6_416	Bra036771	3	20.2	1	23	5375	5397	GGGGAUGUAGCUCAGAUGGUAGA	UCGACUGGCUGAGCUACAUUCCU	Cleavage	1
trf_6_416	Bra018620	3	8.3	1	20	7	26	GGGGAUGUAGCUCAGAUGGU	GCCACCUCAGCUACAUCCUC	Cleavage	1
trf_6_418	Bra007154	2.5	14.1	1	21	510	530	GCACCAGUGGUCUAGUGGUAG	UUACUACUGGACCUUUGGUGC	Cleavage	1
trf_6_419	Bra007154	2.5	14.1	1	20	511	530	GCACCAGUGGUCUAGUGGUA	UACUACUGGACCUUUGGUGC	Cleavage	1
trf_6_420	Bra007154	2.5	14.1	1	20	511	530	GCACCAGUGGUCUAGUGGUA	UACUACUGGACCUUUGGUGC	Cleavage	1
trf_6_422	Bra003753	2.5	16.2	1	20	284	303	GGGAUUGUAGUUCAAUCGGU	ACCAGUUGAAUUACAAUCCU	Cleavage	1
trf_6_423	Bra003753	1.5	16.2	1	20	284	303	GGGAUUGUAGUUCAAUUGGU	ACCAGUUGAAUUACAAUCCU	Cleavage	1
trf_6_423	Bra013336	3	19.5	1	20	267	286	GGGAUUGUAGUUCAAUUGGU	ACUAAUUGAACUAAGAUCUC	Cleavage	1
trf_6_423	Bra021314	3	23.2	1	20	234	253	GGGAUUGUAGUUCAAUUGGU	ACCAACUGAACUGCAAUCUG	Cleavage	1
trf_6_424	Bra003753	1.5	16.2	1	20	284	303	GGGAUUGUAGUUCAAUUGGU	ACCAGUUGAAUUACAAUCCU	Cleavage	1
trf_6_424	Bra013336	3	19.5	1	20	267	286	GGGAUUGUAGUUCAAUUGGU	ACUAAUUGAACUAAGAUCUC	Cleavage	1
trf_6_424	Bra021314	3	23.2	1	20	234	253	GGGAUUGUAGUUCAAUUGGU	ACCAACUGAACUGCAAUCUG	Cleavage	1

trf_6_425	Bra003753	1.5	16.2	1	20	284	303	GGGAUUGUAGUUCAAUUGGU	ACCAGUUGAAUUACAAUCCU	Cleavage	1
trf_6_425	Bra013336	3	19.5	1	20	267	286	GGGAUUGUAGUUCAAUUGGU	ACUAAUUGAACUAAGAUCUC	Cleavage	1
trf_6_425	Bra021314	3	23.2	1	20	234	253	GGGAUUGUAGUUCAAUUGGU	ACCAACUGAACUGCAAUCUG	Cleavage	1
trf_6_427	Bra003753	1.5	16.2	1	20	284	303	GGGAUUGUAGUUCAAUUGGU	ACCAGUUGAAUUACAAUCCU	Cleavage	1
trf_6_427	Bra013336	3	19.5	1	20	267	286	GGGAUUGUAGUUCAAUUGGU	ACUAAUUGAACUAAGAUCUC	Cleavage	1
trf_6_427	Bra021314	3	23.2	1	20	234	253	GGGAUUGUAGUUCAAUUGGU	ACCAACUGAACUGCAAUCUG	Cleavage	1
trf_6_428	Bra003753	2.5	16.2	1	20	284	303	GGGAUUGUAGUUCAAUUGGA	ACCAGUUGAAUUACAAUCCU	Cleavage	1
trf_6_429	Bra003753	2.5	16.2	1	20	284	303	GGGAUUGUAGUUCAAUCGGU	ACCAGUUGAAUUACAAUCCU	Cleavage	1
trf_6_430	Bra003753	1.5	16.2	1	20	284	303	GGGAUUGUAGUUCAAUUGGU	ACCAGUUGAAUUACAAUCCU	Cleavage	1
trf_6_430	Bra013336	3	19.5	1	20	267	286	GGGAUUGUAGUUCAAUUGGU	ACUAAUUGAACUAAGAUCUC	Cleavage	1
trf_6_430	Bra021314	3	23.2	1	20	234	253	GGGAUUGUAGUUCAAUUGGU	ACCAACUGAACUGCAAUCUG	Cleavage	1
trf_6_431	Bra003753	2.5	16.2	1	20	284	303	GGGAUUGUAGUUCAAUCGGU	ACCAGUUGAAUUACAAUCCU	Cleavage	1
trf_6_432	Bra003753	1.5	16.2	1	20	284	303	GGGAUUGUAGUUCAAUUGGU	ACCAGUUGAAUUACAAUCCU	Cleavage	1
trf_6_432	Bra013336	3	19.5	1	20	267	286	GGGAUUGUAGUUCAAUUGGU	ACUAAUUGAACUAAGAUCUC	Cleavage	1
trf_6_432	Bra021314	3	23.2	1	20	234	253	GGGAUUGUAGUUCAAUUGGU	ACCAACUGAACUGCAAUCUG	Cleavage	1
trf_6_433	Bra003753	1.5	16.2	1	20	284	303	GGGAUUGUAGUUCAAUUGGU	ACCAGUUGAAUUACAAUCCU	Cleavage	1
trf_6_433	Bra013336	3	19.5	1	20	267	286	GGGAUUGUAGUUCAAUUGGU	ACUAAUUGAACUAAGAUCUC	Cleavage	1

trf_6_4	Bra021		23					GGGAUUGUAGUUCAA	ACCAACUGAACUGCAA	Cleava	
33	314	3	.2	1	20	234	253	UUGGU	UCUG	ge	1
trf_6_4	Bra003		16					GGGAUUGUAGUUCAA	ACCAGUUGAAUUACAA	Cleava	
34	753	1.5	.2	1	20	284	303	UUGGU	UCCU	ge	1
trf_6_4	Bra013		19					GGGAUUGUAGUUCAA	ACUAAUUGAACUAAGA	Cleava	
34	336	3	.5	1	20	267	286	UUGGU	UCUC	ge	1
trf_6_4	Bra021		23					GGGAUUGUAGUUCAA	ACCAACUGAACUGCAA	Cleava	
34	314	3	.2	1	20	234	253	UUGGU	UCUG	ge	1
trf_6_4	Bra003		16					GGGAUUGUAGUUCAA	ACCAGUUGAAUUACAA	Cleava	
37	753	2.5	.2	1	20	284	303	UUGGA	UCCU	ge	1
trf_6_4	Bra013		22					UCCGUUGUAGUCUAGC	CAUCAUCAGCUUGGCU	Cleava	
41	528	3	.0	1	24	196	219	UGGUCAGG	AUAACGGG	ge	1
trf_6_4	Bra033		24					CCGACCUUAGCUCAGU	UCAACCAACGGAGUUG	Cleava	
42	406	3	.4	1	23	30	52	UGGUAGA	GGGUUGG	ge	1
trf_6_4	Bra033		24					CCGACCUUAGCUCAGU	UCAACCAACGGAGUUG	Cleava	
42	405	3	.4	1	23	30	52	UGGUAGA	GGGUUGG	ge	1
trf_6_4	Bra004		20					CCGACCUUAGCUCAGU	GUUAACUGGGCUAAGG	Cleava	
42	481	3	.0	1	20	1660	1679	UGGU	UCGC	ge	1
trf_6_4	Bra033		24					CCGACCUUAGCUCAGU	UCAACCAACGGAGUUG	Cleava	
43	406	3	.4	1	23	30	52	UGGUAGA	GGGUUGG	ge	1
trf_6_4	Bra033		24					CCGACCUUAGCUCAGU	UCAACCAACGGAGUUG	Cleava	
43	405	3	.4	1	23	30	52	UGGUAGA	GGGUUGG	ge	1
trf_6_4	Bra004		20					CCGACCUUAGCUCAGU	GUUAACUGGGCUAAGG	Cleava	
43	481	3	.0	1	20	1660	1679	UGGU	UCGC	ge	1
trf_6_4	Bra007		14					GCACCAGUGGUCUAGU	UACUACUGGACCUUUG	Cleava	
45	154	2.5	.1	1	20	511	530	GGUA	GUGC	ge	1
trf_6_4	Bra007		14					GCACCAGUGGUCUAGU	UUACUACUGGACCUUU	Cleava	
46	154	2.5	.1	1	21	510	530	GGUAG	GGUGC	ge	1
trf_6_4	Bra014		17					GGGGAUGUAGCUCAUA	UGUAUUAUAUUAGCU	Cleava	
47	507	3	.0	1	23	541	563	UGGUAGA	AUAUCUCC	ge	1
trf_6_4	Bra009		14					GGGGAUGUAGCUCAGA	UUAUCAUCUGGGCUUC	Cleava	
48	876	3	.9	1	22	16	37	UGGUAG	GUCCUC	ge	1
trf_6_4	Bra016		10					GGGGAUGUAGCUCAGA	UUAUCAUCUGAGCUUC	Cleava	
48	141	3	.4	1	22	3031	3052	UGGUAG	GUUCUC	ge	1

trf_6_4	Bra036		20					GGGGAUGUAGCUCAGA	UCGACUGGCUGAGCUA	Cleava	
48	771	3	.2	1	23	5375	5397	UGGUAGA	CAUUCCU	ge	1
trf_6_4	Bra018		8.					GGGGAUGUAGCUCAGA	GCCACCUCAGCUACAUC	Cleava	
48	620	3	3	1	20	7	26	UGGU	CUC	ge	1
trf_6_4	Bra009		14					GGGGAUGUAGCUCAGA	AUCAUCUGGGCUUCGU	Cleava	
49	876	3	.9	1	20	18	37	UGGU	CCUC	ge	1
trf_6_4	Bra016		10					GGGGAUGUAGCUCAGA	AUCAUCUGAGCUUCGU	Cleava	
49	141	3	.4	1	20	3033	3052	UGGU	UCUC	ge	1
trf_6_4	Bra036		20					GGGGAUGUAGCUCAGA	ACUGGCUGAGCUACAU	Cleava	
49	771	3	.2	1	20	5378	5397	UGGU	UCCU	ge	1
trf_6_4	Bra018		8.					GGGGAUGUAGCUCAGA	GCCACCUCAGCUACAUC	Cleava	
49	620	3	3	1	20	7	26	UGGU	CUC	ge	1
trf_6_4	Bra014		17					GGGGAUGUAGCUCAUA	UGUAUUAUAUUAGCU	Cleava	
50	507	3	.0	1	23	541	563	UGGUAGA	AUAUCUCC	ge	1
trf_6_4	Bra014		17					GGGGAUGUAGCUCAUA	UUGUAUUAUAUUAGC	Cleava	
51	507	3	.0	1	24	540	563	UGGUAGAG	UAUAUCUCC	ge	1
trf_6_4	Bra009		14					GGGGAUGUAGCUCAGA	UUAUCAUCUGGGCUUC	Cleava	
52	876	3	.9	1	22	16	37	UGGUAG	GUCCUC	ge	1
trf_6_4	Bra016		10					GGGGAUGUAGCUCAGA	UUAUCAUCUGAGCUUC	Cleava	
52	141	3	.4	1	22	3031	3052	UGGUAG	GUUCUC	ge	1
trf_6_4	Bra036		20					GGGGAUGUAGCUCAGA	UCGACUGGCUGAGCUA	Cleava	
52	771	3	.2	1	23	5375	5397	UGGUAGA	CAUUCCU	ge	1
trf_6_4	Bra018		8.					GGGGAUGUAGCUCAGA	GCCACCUCAGCUACAUC	Cleava	
52	620	3	3	1	20	7	26	UGGU	CUC	ge	1
trf_6_4	Bra013		16					UCCGUUGUCGUCCAGC	UAACCGAUGGAUGUCA	Cleava	
61	584	3	.4	1	21	890	910	GGUUA	AUGGA	ge	1
trf_6_4	Bra000		11					UCCGUUGUCGUCCAGC	UAACCGGAGGACGAUA	Cleava	
61	529	3	.2	1	21	984	1004	GGUUA	AUGGA	ge	1
trf_6_4	Bra013		16					UCCGUUGUCGUCCAGC	AUGAUAACCGAUGGAU	Cleava	
62	584	3	.4	1	25	886	910	GGUUAGGAU	GUCAAUGGA	ge	1
trf_6_4	Bra000		11					UCCGUUGUCGUCCAGC	UAACCGGAGGACGAUA	Cleava	
62	529	3	.2	1	21	984	1004	GGUUA	AUGGA	ge	1
trf_6_4	Bra013		16					UCCGUUGUCGUCCAGC	UAUGAUAACCGAUGGA	Cleava	
63	584	3	.4	1	26	885	910	GGUUAGGAUA	UGUCAAUGGA	ge	1

trf_6_4	Bra000		11					UCCGUUGUCGUCCAGC	UAACCGGAGGACGAUA	Cleava	
63	529	3	.2	1	21	984	1004	GGUUA	AUGGA	ge	1
trf_6_4	Bra013		16					UCCGUUGUCGUCCAGC	UAUGAUAACCGAUGGA	Cleava	
64	584	3	.4	1	26	885	910	GGUUAGGAUA	UGUCAAUGGA	ge	1
trf_6_4	Bra000		11					UCCGUUGUCGUCCAGC	UAACCGGAGGACGAUA	Cleava	
64	529	3	.2	1	21	984	1004	GGUUA	AUGGA	ge	1
trf_6_4	Bra013		16					UCCGUUGUCGUCCAGC	AACCGAUGGAUGUCAA	Cleava	
65	584	3	.4	1	20	891	910	GGUU	UGGA	ge	1
trf_6_4	Bra000		11					UCCGUUGUCGUCCAGC	AACCGGAGGACGAUAA	Cleava	
65	529	3	.2	1	20	985	1004	GGUU	UGGA	ge	1
trf_6_4	Bra013		16					UCCGUUGUCGUCCAGC	UAACCGAUGGAUGUCA	Cleava	
66	584	3	.4	1	21	890	910	GGUUA	AUGGA	ge	1
trf_6_4	Bra000		11					UCCGUUGUCGUCCAGC	UAACCGGAGGACGAUA	Cleava	
66	529	3	.2	1	21	984	1004	GGUUA	AUGGA	ge	1
trf_6_4	Bra013		16					UCCGUUGUCGUCCAGC	UAACCGAUGGAUGUCA	Cleava	
67	584	3	.4	1	21	890	910	GGUUA	AUGGA	ge	1
trf_6_4	Bra000		11					UCCGUUGUCGUCCAGC	UAACCGGAGGACGAUA	Cleava	
67	529	3	.2	1	21	984	1004	GGUUA	AUGGA	ge	1
trf_6_4	Bra007		14					GCACCAGUGGUCUAGU	UACUACUGGACCUUUG	Cleava	
69	154	2.5	.1	1	20	511	530	GGUA	GUGC	ge	1
trf_6_4	Bra016		20					UCCGUCGUAGUCUAGC	CUGAAGAAGCUAGGCU	Cleava	
73	601	3	.8	1	24	415	438	UGGUUAGG	GCGACGGA	ge	1
trf_6_4	Bra016		20					UCCGUCGUAGUCUAGC	AAGAAGCUAGGCUGCG	Cleava	
74	601	3	.8	1	21	418	438	UGGUU	ACGGA	ge	1
trf_6_4	Bra016		20					UCCGUCGUAGUCUAGC	CUGAAGAAGCUAGGCU	Cleava	
75	601	3	.8	1	24	415	438	UGGUUAGG	GCGACGGA	ge	1
trf_6_4	Bra016		20					UCCGUCGUAGUCUAGC	CUGAAGAAGCUAGGCU	Cleava	
76	601	3	.8	1	24	415	438	UGGUUAGG	GCGACGGA	ge	1
trf_6_4	Bra024		7.					GGGGAUGUAGCUCAAA	UCCAACAUUUCGGUUA	Cleava	
77	638	3	5	1	23	203	225	UGGUAGA	CAUCCCC	ge	1
trf_6_4	Bra025		17					GGGGAUGUAGCUCAAA	ACAAUUUGAGCUUCAU	Cleava	
77	904	3	.2	1	20	344	363	UGGU	CUUC	ge	1
trf_6_4	Bra033		24					CCGACCUUAGCUCAGU	UCAACCAACGGAGUUG	Cleava	
78	406	3	.4	1	23	30	52	UGGUAGA	GGGUUGG	ge	1

trf_6_4	Bra033		24					CCGACCUUAGCUCAGU	UCAACCAACGGAGUUG	Cleava	
78	405	3	.4	1	23	30	52	UGGUAGA	GGGUUGG	ge	1
trf_6_4	Bra004		20					CCGACCUUAGCUCAGU	GUUAACUGGGCUAAGG	Cleava	
78	481	3	.0	1	20	1660	1679	UGGU	UCGC	ge	1
trf_6_4	Bra016		20					UCCGUCGUAGUCUAGC	CUGAAGAAGCUAGGCU	Cleava	
79	601	3	.8	1	24	415	438	UGGUUAGG	GCGACGGA	ge	1
trf_6_4	Bra016		20					UCCGUCGUAGUCUAGC	CUGAAGAAGCUAGGCU	Cleava	
80	601	3	.8	1	24	415	438	UGGUUAGG	GCGACGGA	ge	1
trf_6_4	Bra016		20					UCCGUCGUAGUCUAGC	AAGAAGCUAGGCUGCG	Cleava	
82	601	3	.8	1	21	418	438	UGGUU	ACGGA	ge	1
trf_6_4	Bra016		20					UCCGUCGUAGUCUAGC	CUGAAGAAGCUAGGCU	Cleava	
83	601	3	.8	1	24	415	438	UGGUUAGG	GCGACGGA	ge	1
trf_6_4	Bra016		9.					GUCGUUGUAGUAUAG	UACUAUUAUACUACAA	Cleava	
84	584	3	9	1	20	1045	1064	UGGUA	AGGC	ge	1
trf_6_4	Bra035		10					GUCGUUGUAGUAUAG	UACCACAAUACUUCAA	Cleava	
84	409	3	.4	1	20	1328	1347	UGGUA	CGAG	ge	1
trf_6_4	Bra016		9.					GUCGUUGUAGUAUAG	UACUAUUAUACUACAA	Cleava	
85	584	3	9	1	20	1045	1064	UGGUA	AGGC	ge	1
trf_6_4	Bra035		10					GUCGUUGUAGUAUAG	UACCACAAUACUUCAA	Cleava	
85	409	3	.4	1	20	1328	1347	UGGUA	CGAG	ge	1
trf_6_4	Bra016		9.					GUCGUUGUAGUAUAG	UACUAUUAUACUACAA	Cleava	
86	584	3	9	1	20	1045	1064	UGGUA	AGGC	ge	1
trf_6_4	Bra035		10					GUCGUUGUAGUAUAG	UACCACAAUACUUCAA	Cleava	
86	409	3	.4	1	20	1328	1347	UGGUA	CGAG	ge	1
trf_6_4	Bra016		9.					GUCGUUGUAGUAUAG	UACUAUUAUACUACAA	Cleava	
87	584	3	9	1	20	1045	1064	UGGUA	AGGC	ge	1
trf_6_4	Bra035		10					GUCGUUGUAGUAUAG	UACCACAAUACUUCAA	Cleava	
87	409	3	.4	1	20	1328	1347	UGGUA	CGAG	ge	1
trf_6_4	Bra003		23					GCUGGAGUAGCUCAGU	CGAACCAACCGGGCUAC	Cleava	
96	602	3	.1	1	23	271	293	UGGUUAG	UGCAGC	ge	1
trf_6_4	Bra029		20					GCUGGAGUAGCUCAGU	GGUCAGCUGAGCUACU	Cleava	
96	975	3	.8	1	21	1244	1264	UGGUU	CAAGC	ge	1
trf_6_4	Bra003		16					GCUGGAGUAGCUCAGU	CCUAACGGAGCUGCUC	Cleava	
96	013	3	.5	1	20	319	338	UGGU	CAGC	ge	1

trf_6_500	Bra007154	2.5	14.1	1	20	511	530	GCACCAGUGGUCUAGUGGUA	UACUACUGGACCUUUGGUGC	Cleavage	1
trf_6_501	Bra016584	3	9.9	1	20	1045	1064	GUCGUUGUAGUAUAGUGGUA	UACUAUUAUACUACAAAGGC	Cleavage	1
trf_6_502	Bra016584	3	9.9	1	20	1045	1064	GUCGUUGUAGUAUAGUGGUA	UACUAUUAUACUACAAAGGC	Cleavage	1
trf_6_502	Bra035409	3	10.4	1	20	1328	1347	GUCGUUGUAGUAUAGUGGUA	UACCACAAUACUUCAACGAG	Cleavage	1
trf_6_503	Bra016584	3	9.9	1	20	1045	1064	GUCGUUGUAGUAUAGUGGUA	UACUAUUAUACUACAAAGGC	Cleavage	1
trf_6_503	Bra035409	3	10.4	1	20	1328	1347	GUCGUUGUAGUAUAGUGGUA	UACCACAAUACUUCAACGAG	Cleavage	1
trf_6_504	Bra016584	3	9.9	1	20	1045	1064	GUCGUUGUAGUAUAGUGGUA	UACUAUUAUACUACAAAGGC	Cleavage	1
trf_6_504	Bra035409	3	10.4	1	20	1328	1347	GUCGUUGUAGUAUAGUGGUA	UACCACAAUACUUCAACGAG	Cleavage	1
trf_6_505	Bra016584	3	9.9	1	20	1045	1064	GUCGUUGUAGUAUAGUGGUA	UACUAUUAUACUACAAAGGC	Cleavage	1
trf_6_505	Bra035409	3	10.4	1	20	1328	1347	GUCGUUGUAGUAUAGUGGUA	UACCACAAUACUUCAACGAG	Cleavage	1
trf_6_512	Bra013528	3	22.0	1	24	196	219	UCCGUUGUAGUCUAGCUGGUCAGG	CAUCAUCAGCUUGGCUAUAACGGG	Cleavage	1
trf_6_523	Bra000791	2.5	15.9	1	23	954	976	GGGUGUUUGGUCUAGUGGUAUGA	UUACACCACCAGACCAGACACCC	Cleavage	1
trf_6_523	Bra001870	2.5	19.8	1	25	814	838	GGGUGUUUGGUCUAGUGGUAUGAUU	GAGCCUAUCACUGGACCAAACACUA	Cleavage	1
trf_6_523	Bra003793	3	12.4	1	25	516	540	GGGUGUUUGGUCUAGUGGUAUGAUU	AGCCUUAUCACUUGACCAAGCAUCU	Cleavage	1
trf_6_524	Bra024638	3	7.5	1	23	203	225	GGGGAUGUAGCUCAAAUGGUAGA	UCCAACAUUUCGGUUACAUCCCC	Cleavage	1
trf_6_524	Bra025904	3	17.2	1	20	344	363	GGGGAUGUAGCUCAAAUGGU	ACAAUUUGAGCUUCAUCUUC	Cleavage	1
trf_6_527	Bra013528	3	22.0	1	24	196	219	UCCGUUGUAGUCUAGCUGGUCAGG	CAUCAUCAGCUUGGCUAUAACGGG	Cleavage	1

trf_6_534	Bra007154	2.5	14.1	1	20	511	530	GCACCAGUGGUCUAGUGGUA	UACUACUGGACCUUUGGUGC	Cleavage	1
trf_6_536	Bra016584	3	9.9	1	20	1045	1064	GUCGUUGUAGUAUAGUGGUA	UACUAUUAUACUACAAAGGC	Cleavage	1
trf_6_536	Bra035409	3	10.4	1	20	1328	1347	GUCGUUGUAGUAUAGUGGUA	UACCACAAUACUUCAACGAG	Cleavage	1
trf_6_537	Bra016584	3	9.9	1	20	1045	1064	GUCGUUGUAGUAUAGUGGUA	UACUAUUAUACUACAAAGGC	Cleavage	1
trf_6_537	Bra035409	3	10.4	1	20	1328	1347	GUCGUUGUAGUAUAGUGGUA	UACCACAAUACUUCAACGAG	Cleavage	1
trf_6_538	Bra016584	3	9.9	1	20	1045	1064	GUCGUUGUAGUAUAGUGGUA	UACUAUUAUACUACAAAGGC	Cleavage	1
trf_6_538	Bra035409	3	10.4	1	20	1328	1347	GUCGUUGUAGUAUAGUGGUA	UACCACAAUACUUCAACGAG	Cleavage	1
trf_6_539	Bra002904	3	15.6	1	22	1319	1340	GGGGUUCGAAUCUCAGACUAUG	CAUGGUCUGAGAUUCAAACCUU	Cleavage	1
trf_6_4	Bra033370	3	21.6	1	20	4750	4769	GGGGAUGUAGCUCAAAUGGU	ACCCUUUGGGUAACAUCCCC	Translation	1
trf_6_5	Bra033370	3	21.6	1	24	4746	4769	GGGGAUGUAGCUCAAAUGGUAGAG	CUGGACCCUUUGGGUAACAUCCCC	Translation	1
trf_6_7	Bra033370	3	21.6	1	20	4750	4769	GGGGAUGUAGCUCAAAUGGU	ACCCUUUGGGUAACAUCCCC	Translation	1
trf_6_11	Bra037748	2.5	22.8	1	25	561	585	UCCGUUAUCGUCCAGCGGUUAGGAU	GUUGAAACCGCUGUCUGAUAACGGA	Translation	1
trf_6_12	Bra037748	2.5	22.8	1	25	561	585	UCCGUUAUCGUCCAGCGGUUAGGAU	GUUGAAACCGCUGUCUGAUAACGGA	Translation	1
trf_6_13	Bra037748	2.5	22.8	1	20	566	585	UCCGUUAUCGUCCAGCGGUU	AACCGCUGUCUGAUAACGGA	Translation	1
trf_6_14	Bra037748	2.5	22.8	1	25	561	585	UCCGUUAUCGUCCAGCGGUUAGGAU	GUUGAAACCGCUGUCUGAUAACGGA	Translation	1
trf_6_15	Bra037499	3	14.7	1	22	174	195	UCCGUUGUAGUCUAGCUGGUUA	UGACUGGUUAGGCCACAACGGA	Translation	1
trf_6_16	Bra033370	3	21.6	1	20	4750	4769	GGGGAUGUAGCUCAAAUGGU	ACCCUUUGGGUAACAUCCCC	Translation	1

trf_6_17	Bra033370	3	21.6	1	20	4750	4769	GGGGAUGUAGCUCAAAUGGU	ACCCUUUGGGUAACAUCCCC	Translation	1
trf_6_34	Bra033370	3	21.6	1	20	4750	4769	GGGGAUGUAGCUCAAAUGGU	ACCCUUUGGGUAACAUCCCC	Translation	1
trf_6_44	Bra037499	3	14.7	1	22	174	195	UCCGUUGUAGUCUAGCUGGUCA	UGACUGGUUAGGCCACAACGGA	Translation	1
trf_6_71	Bra015824	3	24.0	1	26	913	938	AUCAGAGUGGCGCAGCGGAAGCGUGG	CUCCGUUUCCACUGCACCACUCUGAC	Translation	1
trf_6_71	Bra033352	3	18.3	1	25	586	609	AUCAGAGUGGCGCAGCGGAAGCGUG	CACG-UUACGUUGCACCACUUUGAU	Translation	1
trf_6_82	Bra003191	2.5	7.6	1	20	27	45	GUGGCUGUAGUUUAGUGGUA	UACCAUUAA-CUACAGCCAC	Translation	1
trf_6_85	Bra033370	3	21.6	1	20	4750	4769	GGGGAUGUAGCUCAAAUGGU	ACCCUUUGGGUAACAUCCCC	Translation	1
trf_6_86	Bra033370	3	21.6	1	20	4750	4769	GGGGAUGUAGCUCAAAUGGU	ACCCUUUGGGUAACAUCCCC	Translation	1
trf_6_99	Bra037499	3	14.7	1	22	174	195	UCCGUUGUAGUCUAGCUGGUCA	UGACUGGUUAGGCCACAACGGA	Translation	1
trf_6_100	Bra037499	3	14.7	1	22	174	195	UCCGUUGUAGUCUAGCUGGUCA	UGACUGGUUAGGCCACAACGGA	Translation	1
trf_6_104	Bra037499	3	14.7	1	22	174	195	UCCGUUGUAGUCUAGCUGGUCA	UGACUGGUUAGGCCACAACGGA	Translation	1
trf_6_107	Bra001210	3	15.7	1	21	2173	2193	GGGUGUUUGGUCUAGUGGUAU	GUACCACUAGACGAAUCACCU	Translation	1
trf_6_111	Bra020153	2.5	15.4	1	22	1953	1974	UCCGUUGUCGUCCAGCGGUUAG	CCAACUGCUGGAAGACAAUGGG	Translation	1
trf_6_111	Bra012143	2.5	18.5	1	20	392	411	UCCGUUGUCGUCCAGCGGUU	AAUCGCUGAACCACAACGGA	Translation	1
trf_6_111	Bra037748	3	22.8	1	25	561	585	UCCGUUGUCGUCCAGCGGUUAGGAU	GUUGAAACCGCUGUCUGAUAACGGA	Translation	1
trf_6_112	Bra020153	2.5	15.4	1	20	1955	1974	UCCGUUGUCGUCCAGCGGUU	AACUGCUGGAAGACAAUGGG	Translation	1
trf_6_1	Bra012	2.5	18	1	20	392	411	UCCGUUGUCGUCCAGC	AAUCGCUGAACCACAAC	Transl	1

12	143		.5					GGUU	GGA	ation		
trf_6_1	Bra037		22					UCCGUUGUCGUCCAGC	AACCGCUGUCUGAUAA	Transl		
12	748	3	.8	1	20	566	585	GGUU	CGGA	ation	1	
trf_6_1	Bra020		15					UCCGUUGUCGUCCAGC	CCAACUGCUGGAAGAC	Transl		
13	153	2.5	.4	1	22	1953	1974	GGUUAG	AAUGGG	ation	1	
trf_6_1	Bra012		18					UCCGUUGUCGUCCAGC	AAUCGCUGAACCACAAC	Transl		
13	143	2.5	.5	1	20	392	411	GGUU	GGA	ation	1	
trf_6_1	Bra037		22					UCCGUUGUCGUCCAGC	AACCGCUGUCUGAUAA	Transl		
13	748	3	.8	1	20	566	585	GGUU	CGGA	ation	1	
trf_6_1	Bra020		15					NCCGUUGUCGUCCAGC	CCAACUGCUGGAAGAC	Transl		
14	153	3	.4	1	22	1953	1974	GGUUAG	AAUGGG	ation	1	
trf_6_1	Bra020		15					UCCGUUGUCGUCCAGC	CCAACUGCUGGAAGAC	Transl		
16	153	2.5	.4	1	22	1953	1974	GGUUAG	AAUGGG	ation	1	
trf_6_1	Bra012		18					UCCGUUGUCGUCCAGC	AAUCGCUGAACCACAAC	Transl		
16	143	2.5	.5	1	20	392	411	GGUU	GGA	ation	1	
trf_6_1	Bra037		22					UCCGUUGUCGUCCAGC	AACCGCUGUCUGAUAA	Transl		
16	748	3	.8	1	20	566	585	GGUU	CGGA	ation	1	
trf_6_1	Bra020		15					UCCGUUGUCGUCCAGC	CCAACUGCUGGAAGAC	Transl		
17	153	2.5	.4	1	22	1953	1974	GGUUAG	AAUGGG	ation	1	
trf_6_1	Bra012		18					UCCGUUGUCGUCCAGC	AAUCGCUGAACCACAAC	Transl		
17	143	2.5	.5	1	20	392	411	GGUU	GGA	ation	1	
trf_6_1	Bra037		22					UCCGUUGUCGUCCAGC	GUUGAAACCGCUGUCU	Transl		
17	748	3	.8	1	25	561	585	GGUUAGGAU	GAUAACGGA	ation	1	
trf_6_1	Bra020		15					UCCGUUGUCGUCCAGC	CCAACUGCUGGAAGAC	Transl		
18	153	2.5	.4	1	22	1953	1974	GGUUAG	AAUGGG	ation	1	
trf_6_1	Bra012		18					UCCGUUGUCGUCCAGC	AAUCGCUGAACCACAAC	Transl		
18	143	2.5	.5	1	20	392	411	GGUU	GGA	ation	1	
trf_6_1	Bra037		22					UCCGUUGUCGUCCAGC	GUUGAAACCGCUGUCU	Transl		
18	748	3	.8	1	25	561	585	GGUUAGGAU	GAUAACGGA	ation	1	
trf_6_1	Bra033		21					GGGGAUGUAGCUCAAA	ACCCUUUGGGUAACAU	Transl		
43	370	3	.6	1	20	4750	4769	UGGU	CCCC	ation	1	
trf_6_1	Bra033		21					GGGGAUGUAGCUCAAA	ACCCUUUGGGUAACAU	Transl		
45	370	3	.6	1	20	4750	4769	UGGU	CCCC	ation	1	
trf_6_1	Bra037	3	12	1	22	478	499	GACGGUUUGGCCGAGU	UUGACUACUCGUCCAA	Transl	1	

52	152		.2					GGUCUA	ACCCUC	ation	
trf_6_1	Bra018		7.					GACGGUUUGGCCGAGU	CCCAAGACCACUCCUCG	Transl	
52	257	3	1	1	25	27	51	GGUCUAAGG	AAACCGUC	ation	1
trf_6_1	Bra015		24					AUCAGAGUGGCGCAGC	CUCCGUUUCCACUGCA	Transl	
53	824	3	.0	1	26	913	938	GGAAGCGUGG	CCACUCUGAC	ation	1
									CACG-		
trf_6_1	Bra033		18					AUCAGAGUGGCGCAGC	UUACGUUGCACCACUU	Transl	
53	352	3	.3	1	25	586	609	GGAAGCGUG	UGAU	ation	1
trf_6_1	Bra037		14					UCCGUUGUAGUCUAGC	UGACUGGUUAGGCCAC	Transl	
57	499	3	.7	1	22	174	195	UGGUCA	AACGGA	ation	1
trf_6_1	Bra033		21					GGGGAUGUAGCUCAAA	ACCCUUUGGGUAACAU	Transl	
59	370	3	.6	1	20	4750	4769	UGGU	CCCC	ation	1
trf_6_1	Bra033		21					GGGGAUGUAGCUCAAA	ACCCUUUGGGUAACAU	Transl	
60	370	3	.6	1	20	4750	4769	UGGU	CCCC	ation	1
trf_6_1	Bra037		14					UCCGUUGUAGUCUAGC	UGACUGGUUAGGCCAC	Transl	
70	499	3	.7	1	22	174	195	UGGUCA	AACGGA	ation	1
trf_6_1	Bra015		24					AUCAGAGUGGCGCAGC	CUCCGUUUCCACUGCA	Transl	
76	824	3	.0	1	26	913	938	GGAAGCGUGG	CCACUCUGAC	ation	1
									CACG-		
trf_6_1	Bra033		18					AUCAGAGUGGCGCAGC	UUACGUUGCACCACUU	Transl	
76	352	3	.3	1	25	586	609	GGAAGCGUG	UGAU	ation	1
trf_6_1	Bra036		13					GUGGCUGUAGUUUAG	CAACACUAAAGUGCAG	Transl	
85	328	3	.8	1	20	708	727	UGGUG	CUAC	ation	1
trf_6_1	Bra027		22					GCCGACUUAGCUCAGU	UACCACAGGGCGAAGU	Transl	
98	297	3	.9	1	20	178	197	GGUA	UGGC	ation	1
trf_6_1	Bra037		14					UCCGUUGUAGUCUAGC	UGACUGGUUAGGCCAC	Transl	
99	499	3	.7	1	22	174	195	UGGUCA	AACGGA	ation	1
trf_6_2	Bra037		14					UCCGUUGUAGUCUAGC	UGACUGGUUAGGCCAC	Transl	
30	499	3	.7	1	22	174	195	UGGUCA	AACGGA	ation	1
trf_6_2	Bra037		22					UCCGUUAUCGUCCAGC	GUUGAAACCGCUGUCU	Transl	
45	748	2.5	.8	1	25	561	585	GGUUAGGAU	GAUAACGGA	ation	1
trf_6_2	Bra037		22					UCCGUUAUCGUCCAGC	AACCGCUGUCUGAUAA	Transl	
46	748	2.5	.8	1	20	566	585	GGUU	CGGA	ation	1
trf_6_2	Bra037	2.5	22	1	25	561	585	UCCGUUAUCGUCCAGC	GUUGAAACCGCUGUCU	Transl	1

47	748		.8					GGUUAGGAU	GAUAACGGA	ation	
trf_6_2	Bra037		22					UCCGUUAUCGUCCAGC	GUUGAAACCGCUGUCU	Transl	
48	748	2.5	.8	1	25	561	585	GGUUAGGAU	GAUAACGGA	ation	1
trf_6_2	Bra015		24					AUCAGAGUGGCGCAGC	CUCCGUUUCCACUGCA	Transl	
69	824	3	.0	1	26	913	938	GGAAGCGUGG	CCACUCUGAC	ation	1
									CACG-		
trf_6_2	Bra033		18					AUCAGAGUGGCGCAGC	UUACGUUGCACCACUU	Transl	
69	352	3	.3	1	25	586	609	GGAAGCGUG	UGAU	ation	1
trf_6_2	Bra015		24					AUCAGAGUGGCGCAGC	CUCCGUUUCCACUGCA	Transl	
83	824	3	.0	1	26	913	938	GGAAGCGUGG	CCACUCUGAC	ation	1
									CACG-		
trf_6_2	Bra033		18					AUCAGAGUGGCGCAGC	UUACGUUGCACCACUU	Transl	
83	352	3	.3	1	25	586	609	GGAAGCGUG	UGAU	ation	1
trf_6_2	Bra020		15					UCCGUUGUCGUCCAGC	CCAACUGCUGGAAGAC	Transl	
84	153	2.5	.4	1	22	1953	1974	GGUUAG	AAUGGG	ation	1
trf_6_2	Bra012		18					UCCGUUGUCGUCCAGC	AAUCGCUGAACCACAAC	Transl	
84	143	2.5	.5	1	20	392	411	GGUU	GGA	ation	1
trf_6_2	Bra037		22					UCCGUUGUCGUCCAGC	GUUGAAACCGCUGUCU	Transl	
84	748	3	.8	1	25	561	585	GGUUAGGAU	GAUAACGGA	ation	1
trf_6_2	Bra020		15					UCCGUUGUCGUCCAGC	AACUGCUGGAAGACAA	Transl	
85	153	2.5	.4	1	20	1955	1974	GGUU	UGGG	ation	1
trf_6_2	Bra012		18					UCCGUUGUCGUCCAGC	AAUCGCUGAACCACAAC	Transl	
85	143	2.5	.5	1	20	392	411	GGUU	GGA	ation	1
trf_6_2	Bra037		22					UCCGUUGUCGUCCAGC	AACCGCUGUCUGAUAA	Transl	
85	748	3	.8	1	20	566	585	GGUU	CGGA	ation	1
trf_6_2	Bra020		15					UCCGUUGUCGUCCAGC	CCAACUGCUGGAAGAC	Transl	
86	153	2.5	.4	1	22	1953	1974	GGUUAG	AAUGGG	ation	1
trf_6_2	Bra012		18					UCCGUUGUCGUCCAGC	AAUCGCUGAACCACAAC	Transl	
86	143	2.5	.5	1	20	392	411	GGUU	GGA	ation	1
trf_6_2	Bra037		22					UCCGUUGUCGUCCAGC	AACCGCUGUCUGAUAA	Transl	
86	748	3	.8	1	20	566	585	GGUU	CGGA	ation	1
trf_6_2	Bra020		15					UCCGUUGUCGUCCAGC	CCAACUGCUGGAAGAC	Transl	
87	153	2.5	.4	1	22	1953	1974	GGUUAG	AAUGGG	ation	1
trf_6_2	Bra012	2.5	18	1	20	392	411	UCCGUUGUCGUCCAGC	AAUCGCUGAACCACAAC	Transl	1

87	143		.5					GGUU	GGA	ation	
trf_6_2	Bra037		22					UCCGUUGUCGUCCAGC	GUUGAAACCGCUGUCU	Transl	
87	748	3	.8	1	25	561	585	GGUUAGGAU	GAUAACGGA	ation	1
trf_6_2	Bra020		15					UCCGUUGUCGUCCAGC	CCAACUGCUGGAAGAC	Transl	
88	153	2.5	.4	1	22	1953	1974	GGUUAG	AAUGGG	ation	1
trf_6_2	Bra012		18					UCCGUUGUCGUCCAGC	AAUCGCUGAACCACAAC	Transl	
88	143	2.5	.5	1	20	392	411	GGUU	GGA	ation	1
trf_6_2	Bra037		22					UCCGUUGUCGUCCAGC	GUUGAAACCGCUGUCU	Transl	
88	748	3	.8	1	25	561	585	GGUUAGGAU	GAUAACGGA	ation	1
trf_6_2	Bra015		24					AUCAGAGUGGCGCAGC	CUCCGUUUCCACUGCA	Transl	
98	824	3	.0	1	26	913	938	GGAAGCGUGG	CCACUCUGAC	ation	1
									CACG-		
trf_6_2	Bra033		18					AUCAGAGUGGCGCAGC	UUACGUUGCACCACUU	Transl	
98	352	3	.3	1	25	586	609	GGAAGCGUG	UGAU	ation	1
trf_6_3	Bra037		14					UCCGUUGUAGUCUAGC	UGACUGGUUAGGCCAC	Transl	
05	499	3	.7	1	22	174	195	UGGUCA	AACGGA	ation	1
trf_6_3	Bra020		15					UCCGUUGUCGUCCAGC	CCAACUGCUGGAAGAC	Transl	
13	153	2.5	.4	1	22	1953	1974	GGUUAG	AAUGGG	ation	1
trf_6_3	Bra012		18					UCCGUUGUCGUCCAGC	AAUCGCUGAACCACAAC	Transl	
13	143	2.5	.5	1	20	392	411	GGUU	GGA	ation	1
trf_6_3	Bra037		22					UCCGUUGUCGUCCAGC	AACCGCUGUCUGAUAA	Transl	
13	748	3	.8	1	20	566	585	GGUU	CGGA	ation	1
trf_6_3	Bra020		15					UCCGUUGUCGUCCAGC	CCAACUGCUGGAAGAC	Transl	
14	153	2.5	.4	1	22	1953	1974	GGUUAG	AAUGGG	ation	1
trf_6_3	Bra012		18					UCCGUUGUCGUCCAGC	AAUCGCUGAACCACAAC	Transl	
14	143	2.5	.5	1	20	392	411	GGUU	GGA	ation	1
trf_6_3	Bra037		22					UCCGUUGUCGUCCAGC	GUUGAAACCGCUGUCU	Transl	
14	748	3	.8	1	25	561	585	GGUUAGGAU	GAUAACGGA	ation	1
trf_6_3	Bra020		15					UCCGUUGUCGUCCAGC	CCAACUGCUGGAAGAC	Transl	
15	153	2.5	.4	1	22	1953	1974	GGUUAG	AAUGGG	ation	1
trf_6_3	Bra012		18					UCCGUUGUCGUCCAGC	AAUCGCUGAACCACAAC	Transl	
15	143	2.5	.5	1	20	392	411	GGUU	GGA	ation	1
trf_6_3	Bra037		22					UCCGUUGUCGUCCAGC	AACCGCUGUCUGAUAA	Transl	
15	748	3	.8	1	20	566	585	GGUU	CGGA	ation	1

trf_6_3<br>16	Bra020<br>153	2.5	15<br>.4	1	22	1953	1974	UCCGUUGUCGUCCAGC<br>GGUUAG	CCAACUGCUGGAAGAC<br>AAUGGG	Transl<br>ation	1
trf_6_3<br>16	Bra012<br>143	2.5	18<br>.5	1	20	392	411	UCCGUUGUCGUCCAGC<br>GGUU	AAUCGCUGAACCACAAC<br>GGA	Transl<br>ation	1
trf_6_3<br>16	Bra037<br>748	3	22<br>.8	1	25	561	585	UCCGUUGUCGUCCAGC<br>GGUUAGGAU	GUUGAAACCGCUGUCU<br>GAUAACGGA	Transl<br>ation	1
trf_6_3<br>17	Bra020<br>153	2.5	15<br>.4	1	22	1953	1974	UCCGUUGUCGUCCAGC<br>GGUUAG	CCAACUGCUGGAAGAC<br>AAUGGG	Transl<br>ation	1
trf_6_3<br>17	Bra012<br>143	2.5	18<br>.5	1	20	392	411	UCCGUUGUCGUCCAGC<br>GGUU	AAUCGCUGAACCACAAC<br>GGA	Transl<br>ation	1
trf_6_3<br>17	Bra037<br>748	3	22<br>.8	1	20	566	585	UCCGUUGUCGUCCAGC<br>GGUU	AACCGCUGUCUGAUAA<br>CGGA	Transl<br>ation	1
trf_6_3<br>18	Bra020<br>153	2.5	15<br>.4	1	22	1953	1974	UCCGUUGUCGUCCAGC<br>GGUUAG	CCAACUGCUGGAAGAC<br>AAUGGG	Transl<br>ation	1
trf_6_3<br>18	Bra012<br>143	2.5	18<br>.5	1	20	392	411	UCCGUUGUCGUCCAGC<br>GGUU	AAUCGCUGAACCACAAC<br>GGA	Transl<br>ation	1
trf_6_3<br>18	Bra037<br>748	3	22<br>.8	1	25	561	585	UCCGUUGUCGUCCAGC<br>GGUUAGGAU	GUUGAAACCGCUGUCU<br>GAUAACGGA	Transl<br>ation	1
trf_6_3<br>19	Bra020<br>153	3	15<br>.4	1	22	1953	1974	NCCGUUGUCGUCCAGC<br>GGUUAG	CCAACUGCUGGAAGAC<br>AAUGGG	Transl<br>ation	1
trf_6_3<br>20	Bra020<br>153	2.5	15<br>.4	1	20	1955	1974	UCCGUUGUCGUCCAGC<br>GGUU	AACUGCUGGAAGACAA<br>UGGG	Transl<br>ation	1
trf_6_3<br>20	Bra012<br>143	2.5	18<br>.5	1	20	392	411	UCCGUUGUCGUCCAGC<br>GGUU	AAUCGCUGAACCACAAC<br>GGA	Transl<br>ation	1
trf_6_3<br>20	Bra037<br>748	3	22<br>.8	1	20	566	585	UCCGUUGUCGUCCAGC<br>GGUU	AACCGCUGUCUGAUAA<br>CGGA	Transl<br>ation	1
trf_6_3<br>38	Bra015<br>824	3	24<br>.0	1	26	913	938	AUCAGAGUGGCGCAGC<br>GGAAGCGUGG	CUCCGUUUCCACUGCA<br>CCACUCUGAC	Transl<br>ation	1
trf_6_3<br>38	Bra033<br>352	3	18<br>.3	1	25	586	609	AUCAGAGUGGCGCAGC<br>GGAAGCGUG	CACG-<br>UUACGUUGCACCACUU<br>UGAU	Transl<br>ation	1
trf_6_3<br>39	Bra015<br>824	3	24<br>.0	1	26	913	938	AUCAGAGUGGCGCAGC<br>GGAAGCGUGG	CUCCGUUUCCACUGCA<br>CCACUCUGAC	Transl<br>ation	1
trf_6_3	Bra033	3	18	1	25	586	609	AUCAGAGUGGCGCAGC	CACG-	Transl	1

39	352		.3					GGAAGCGUG	UUACGUUGCACCACUU	ation	
									UGAU		
trf_6_3	Bra015		24					AUCAGAGUGGCGCAGC	CUCCGUUUCCACUGCA	Transl	
49	824	3	.0	1	26	913	938	GGAAGCGUGG	CCACUCUGAC	ation	1
									CACG-		
trf_6_3	Bra033		18					AUCAGAGUGGCGCAGC	UUACGUUGCACCACUU	Transl	
49	352	3	.3	1	25	586	609	GGAAGCGUG	UGAU	ation	1
trf_6_3	Bra033		21					GGGGAUGUAGCUCAAA	ACCCUUUGGGUAACAU	Transl	
64	370	3	.6	1	20	4750	4769	UGGU	CCCC	ation	1
trf_6_3	Bra015		24					AUCAGAGUGGCGCAGC	CUCCGUUUCCACUGCA	Transl	
76	824	3	.0	1	26	913	938	GGAAGCGUGG	CCACUCUGAC	ation	1
									CACG-		
trf_6_3	Bra033		18					AUCAGAGUGGCGCAGC	UUACGUUGCACCACUU	Transl	
76	352	3	.3	1	25	586	609	GGAAGCGUG	UGAU	ation	1
trf_6_3	Bra027		22					GCCGACUUAGCUCAGU	UACCACAGGGCGAAGU	Transl	
78	297	3	.9	1	20	178	197	GGUA	UGGC	ation	1
trf_6_3	Bra015		24					AUCAGAGUGGCGCAGC	CUCCGUUUCCACUGCA	Transl	
84	824	3	.0	1	26	913	938	GGAAGCGUGG	CCACUCUGAC	ation	1
									CACG-		
trf_6_3	Bra033		18					AUCAGAGUGGCGCAGC	UUACGUUGCACCACUU	Transl	
84	352	3	.3	1	25	586	609	GGAAGCGUG	UGAU	ation	1
trf_6_3	Bra015		24					AUCAGAGUGGCGCAGC	CUCCGUUUCCACUGCA	Transl	
85	824	3	.0	1	26	913	938	GGAAGCGUGG	CCACUCUGAC	ation	1
									CACG-		
trf_6_3	Bra033		18					AUCAGAGUGGCGCAGC	UUACGUUGCACCACUU	Transl	
85	352	3	.3	1	25	586	609	GGAAGCGUG	UGAU	ation	1
trf_6_3	Bra033		21					GGGGAUGUAGCUCAAA	ACCCUUUGGGUAACAU	Transl	
87	370	3	.6	1	20	4750	4769	UGGU	CCCC	ation	1
trf_6_4	Bra033		10					GGGAUUGUAGUUCAA	ACCGAUUGAUCAGCAA	Transl	
22	536	3	.5	1	20	426	445	UCGGU	UUCC	ation	1
trf_6_4	Bra023		12					GGGAUUGUAGUUCAA	UUCCAAGUGAACAUCA	Transl	
28	172	3	.4	1	21	613	633	UUGGAA	AUCCC	ation	1
trf_6_4	Bra034		11					GGGAUUGUAGUUCAA	UCCAAAUGAGCAGCAA	Transl	
28	753	3	.6	1	20	443	462	UUGGA	UCCC	ation	1

trf_6_428	Bra003597	3	13.1	1	20	1508	1527	GGGAUUGUAGUUCAAUUGGA	UCCAGUUAAUCUACAAUUCC	Translation	1
trf_6_429	Bra033536	3	10.5	1	20	426	445	GGGAUUGUAGUUCAAUCGGU	ACCGAUUGAUCAGCAAUUCC	Translation	1
trf_6_431	Bra033536	3	10.5	1	20	426	445	GGGAUUGUAGUUCAAUCGGU	ACCGAUUGAUCAGCAAUUCC	Translation	1
trf_6_437	Bra034753	3	11.6	1	20	443	462	GGGAUUGUAGUUCAAUUGGA	UCCAAAUGAGCAGCAAUCCC	Translation	1
trf_6_437	Bra003597	3	13.1	1	20	1508	1527	GGGAUUGUAGUUCAAUUGGA	UCCAGUUAAUCUACAAUUCC	Translation	1
trf_6_437	Bra023172	3	12.4	1	20	614	633	GGGAUUGUAGUUCAAUUGGA	UCCAAGUGAACAUCAAUCCC	Translation	1
trf_6_441	Bra037499	3	14.7	1	22	174	195	UCCGUUGUAGUCUAGCUGGUCA	UGACUGGUUAGGCCACAACGGA	Translation	1
trf_6_442	Bra018971	3	15.6	1	22	132	153	CCGACCUUAGCUCAGUUGGUAG	CAACAAACUGGGUGAAGGUCGG	Translation	1
trf_6_442	Bra009057	3	16.0	1	20	737	756	CCGACCUUAGCUCAGUUGGU	UCAAACUGAGCCAAGGUCGG	Translation	1
trf_6_443	Bra018971	3	15.6	1	22	132	153	CCGACCUUAGCUCAGUUGGUAG	CAACAAACUGGGUGAAGGUCGG	Translation	1
trf_6_443	Bra009057	3	16.0	1	20	737	756	CCGACCUUAGCUCAGUUGGU	UCAAACUGAGCCAAGGUCGG	Translation	1
trf_6_461	Bra020153	2.5	15.4	1	22	1953	1974	UCCGUUGUCGUCCAGCGGUUAG	CCAACUGCUGGAAGACAAUGGG	Translation	1
trf_6_461	Bra012143	2.5	18.5	1	20	392	411	UCCGUUGUCGUCCAGCGGUU	AAUCGCUGAACCACAACGGA	Translation	1
trf_6_461	Bra037748	3	22.8	1	20	566	585	UCCGUUGUCGUCCAGCGGUU	AACCGCUGUCUGAUAACGGA	Translation	1
trf_6_462	Bra020153	2.5	15.4	1	22	1953	1974	UCCGUUGUCGUCCAGCGGUUAG	CCAACUGCUGGAAGACAAUGGG	Translation	1
trf_6_462	Bra012143	2.5	18.5	1	20	392	411	UCCGUUGUCGUCCAGCGGUU	AAUCGCUGAACCACAACGGA	Translation	1
trf_6_462	Bra037748	3	22.8	1	25	561	585	UCCGUUGUCGUCCAGCGGUUAGGAU	GUUGAAACCGCUGUCUGAUAACGGA	Translation	1

trf_6_4	Bra020		15					UCCGUUGUCGUCCAGC	CCAACUGCUGGAAGAC	Transl	
63	153	2.5	.4	1	22	1953	1974	GGUUAG	AAUGGG	ation	1
trf_6_4	Bra012		18					UCCGUUGUCGUCCAGC	AAUCGCUGAACCACAAC	Transl	
63	143	2.5	.5	1	20	392	411	GGUU	GGA	ation	1
trf_6_4	Bra037		22					UCCGUUGUCGUCCAGC	GUUGAAACCGCUGUCU	Transl	
63	748	3	.8	1	25	561	585	GGUUAGGAU	GAUAACGGA	ation	1
trf_6_4	Bra020		15					UCCGUUGUCGUCCAGC	CCAACUGCUGGAAGAC	Transl	
64	153	2.5	.4	1	22	1953	1974	GGUUAG	AAUGGG	ation	1
trf_6_4	Bra012		18					UCCGUUGUCGUCCAGC	AAUCGCUGAACCACAAC	Transl	
64	143	2.5	.5	1	20	392	411	GGUU	GGA	ation	1
trf_6_4	Bra037		22					UCCGUUGUCGUCCAGC	GUUGAAACCGCUGUCU	Transl	
64	748	3	.8	1	25	561	585	GGUUAGGAU	GAUAACGGA	ation	1
trf_6_4	Bra020		15					UCCGUUGUCGUCCAGC	AACUGCUGGAAGACAA	Transl	
65	153	2.5	.4	1	20	1955	1974	GGUU	UGGG	ation	1
trf_6_4	Bra012		18					UCCGUUGUCGUCCAGC	AAUCGCUGAACCACAAC	Transl	
65	143	2.5	.5	1	20	392	411	GGUU	GGA	ation	1
trf_6_4	Bra037		22					UCCGUUGUCGUCCAGC	AACCGCUGUCUGAUAA	Transl	
65	748	3	.8	1	20	566	585	GGUU	CGGA	ation	1
trf_6_4	Bra020		15					UCCGUUGUCGUCCAGC	AACUGCUGGAAGACAA	Transl	
66	153	2.5	.4	1	20	1955	1974	GGUU	UGGG	ation	1
trf_6_4	Bra012		18					UCCGUUGUCGUCCAGC	AAUCGCUGAACCACAAC	Transl	
66	143	2.5	.5	1	20	392	411	GGUU	GGA	ation	1
trf_6_4	Bra037		22					UCCGUUGUCGUCCAGC	AACCGCUGUCUGAUAA	Transl	
66	748	3	.8	1	20	566	585	GGUU	CGGA	ation	1
trf_6_4	Bra020		15					UCCGUUGUCGUCCAGC	CCAACUGCUGGAAGAC	Transl	
67	153	2.5	.4	1	22	1953	1974	GGUUAG	AAUGGG	ation	1
trf_6_4	Bra012		18					UCCGUUGUCGUCCAGC	AAUCGCUGAACCACAAC	Transl	
67	143	2.5	.5	1	20	392	411	GGUU	GGA	ation	1
trf_6_4	Bra037		22					UCCGUUGUCGUCCAGC	AACCGCUGUCUGAUAA	Transl	
67	748	3	.8	1	20	566	585	GGUU	CGGA	ation	1
trf_6_4	Bra033		21					GGGGAUGUAGCUCAAA	ACCCUUUGGGUAACAU	Transl	
77	370	3	.6	1	20	4750	4769	UGGU	CCCC	ation	1
trf_6_4	Bra018		15					CCGACCUUAGCUCAGU	CAACAAACUGGGUGAA	Transl	
78	971	3	.6	1	22	132	153	UGGUAG	GGUCGG	ation	1

trf_6_478	Bra009057	3	16.0	1	20	737	756	CCGACCUUAGCUCAGUUGGU	UCAAACUGAGCCAAGGUCGG	Translation	1
trf_6_496	Bra032168	2.5	22.3	1	20	150	169	GCUGGAGUAGCUCAGUUGGU	AUCGGCUGAGCCACUCCAGC	Translation	1
trf_6_496	Bra005116	3	13.5	1	21	233	254	GCUGGAGU-AGCUCAGUUGGUU	AACCAACUGAUCUCACUCCAGC	Translation	1
trf_6_496	Bra022251	3	23.9	1	20	3475	3494	GCUGGAGUAGCUCAGUUGGU	AUCAAUUGGGAUACUCUAGC	Translation	1
trf_6_512	Bra037499	3	14.7	1	22	174	195	UCCGUUGUAGUCUAGCUGGUCA	UGACUGGUUAGGCCACAACGGA	Translation	1
trf_6_513	Bra015824	3	24.0	1	26	913	938	AUCAGAGUGGCGCAGCGGAAGCGUGG	CUCCGUUUCCACUGCACCACUCUGAC	Translation	1
trf_6_513	Bra033352	3	18.3	1	25	586	609	AUCAGAGUGGCGCAGCGGAAGCGUG	CACG-UUACGUUGCACCACUUUGAU	Translation	1
trf_6_523	Bra001210	3	15.7	1	21	2173	2193	GGGUGUUUGGUCUAGUGGUAU	GUACCACUAGACGAAUCACCU	Translation	1
trf_6_524	Bra033370	3	21.6	1	20	4750	4769	GGGGAUGUAGCUCAAAUGGU	ACCCUUUGGGUAACAUCCCC	Translation	1
trf_6_525	Bra015824	3	24.0	1	26	913	938	AUCAGAGUGGCGCAGCGGAAGCGUGG	CUCCGUUUCCACUGCACCACUCUGAC	Translation	1
trf_6_525	Bra033352	3	18.3	1	25	586	609	AUCAGAGUGGCGCAGCGGAAGCGUG	CACG-UUACGUUGCACCACUUUGAU	Translation	1
trf_6_527	Bra037499	3	14.7	1	22	174	195	UCCGUUGUAGUCUAGCUGGUCA	UGACUGGUUAGGCCACAACGGA	Translation	1

tRF Endosperm

ncRNA_Acc.	Target_Acc.	Expectation	UPE	ncRNA_start	ncRNA_end	Target_start	Target_end	ncRNA_aligned_fragment	Target_aligned_fragment	Inhibition	Multiplicity
trf_7_1	Bra024638	3	7.5	1	23	203	225	GGGGAUGUAGCUCAAAUGGUAGA	UCCAACAUUUCGGUUACAUCCCC	Cleavage	1
trf_7_1	Bra025904	3	17.2	1	20	344	363	GGGGAUGUAGCUCAAAUGGU	ACAAUUUGAGCUUCAUCUUC	Cleavage	1
trf_7_2	Bra024638	3	7.5	1	23	203	225	GGGGAUGUAGCUCAAAUGGUAGA	UCCAACAUUUCGGUUACAUCCCC	Cleavage	1
trf_7_2	Bra025904	3	17.2	1	20	344	363	GGGGAUGUAGCUCAAAUGGU	ACAAUUUGAGCUUCAUCUUC	Cleavage	1
trf_7_3	Bra000529	2.5	11.2	1	21	984	1004	UCCGUUAUCGUCCAGCGGUUA	UAACCGGAGGACGAUAAUGGA	Cleavage	1
trf_7_3	Bra032111	3	22.4	1	20	727	746	UCCGUUAUCGUCCAGCGGUU	GACGGCUUGACGAUGACGGA	Cleavage	1
trf_7_4	Bra000529	2.5	11.2	1	21	984	1004	UCCGUUAUCGUCCAGCGGUUA	UAACCGGAGGACGAUAAUGGA	Cleavage	1
trf_7_4	Bra032111	3	22.4	1	20	727	746	UCCGUUAUCGUCCAGCGGUU	GACGGCUUGACGAUGACGGA	Cleavage	1
trf_7_5	Bra000529	2.5	11.2	1	21	984	1004	UCCGUUAUCGUCCAGCGGUUA	UAACCGGAGGACGAUAAUGGA	Cleavage	1
trf_7_5	Bra032111	3	22.4	1	20	727	746	UCCGUUAUCGUCCAGCGGUU	GACGGCUUGACGAUGACGGA	Cleavage	1
trf_7_6	Bra000529	2.5	11.2	1	21	984	1004	UCCGUUAUCGUCCAGCGGUUA	UAACCGGAGGACGAUAAUGGA	Cleavage	1
trf_7_6	Bra032111	3	22.4	1	20	727	746	UCCGUUAUCGUCCAGCGGUU	GACGGCUUGACGAUGACGGA	Cleavage	1
trf_7_7	Bra000529	2.5	11.2	1	21	984	1004	UCCGUUAUCGUCCAGCGGUUA	UAACCGGAGGACGAUAAUGGA	Cleavage	1
trf_7_7	Bra032111	3	22.4	1	20	727	746	UCCGUUAUCGUCCAGCGGUU	GACGGCUUGACGAUGACGGA	Cleavage	1
trf_7_8	Bra000529	2.5	11.2	1	20	985	1004	UCCGUUAUCGUCCAGCGGUU	AACCGGAGGACGAUAAUGGA	Cleavage	1

trf_7_8	Bra032111	3	22.4	1	20	727	746	UCCGUUAUCGUCCAGCGGUU	GACGGCUUGACGAUGACGGA	Cleavage	1
trf_7_9	Bra000529	2.5	11.2	1	21	984	1004	UCCGUUAUCGUCCAGCGGUUA	UAACCGGAGGACGAUAAUGGA	Cleavage	1
trf_7_9	Bra032111	3	22.4	1	20	727	746	UCCGUUAUCGUCCAGCGGUU	GACGGCUUGACGAUGACGGA	Cleavage	1
trf_7_12	Bra013528	3	22.0	1	24	196	219	UCCGUUGUAGUCUAGCUGGUUAGG	CAUCAUCAGCUUGGCUAUAACGGG	Cleavage	1
trf_7_13	Bra024638	3	7.5	1	23	203	225	GGGGAUGUAGCUCAAAUGGUAGA	UCCAACAUUUCGGUUACAUCCCC	Cleavage	1
trf_7_13	Bra025904	3	17.2	1	20	344	363	GGGGAUGUAGCUCAAAUGGU	ACAAUUUGAGCUUCAUCUUC	Cleavage	1
trf_7_15	Bra016601	3	20.8	1	24	415	438	UCCGUCGUAGUCUAGCUGGUUAGG	CUGAAGAAGCUAGGCUGCGACGGA	Cleavage	1
trf_7_16	Bra016601	3	20.8	1	24	415	438	UCCGUCGUAGUCUAGCUGGUUAGG	CUGAAGAAGCUAGGCUGCGACGGA	Cleavage	1
trf_7_18	Bra024638	3	7.5	1	23	203	225	GGGGAUGUAGCUCAAAUGGUAGA	UCCAACAUUUCGGUUACAUCCCC	Cleavage	1
trf_7_18	Bra025904	3	17.2	1	20	344	363	GGGGAUGUAGCUCAAAUGGU	ACAAUUUGAGCUUCAUCUUC	Cleavage	1
trf_7_19	Bra009876	3	14.9	1	22	16	37	GGGGAUGUAGCUCAGAUGGUAG	UUAUCAUCUGGGCUUCGUCCUC	Cleavage	1
trf_7_19	Bra016141	3	10.4	1	22	3031	3052	GGGGAUGUAGCUCAGAUGGUAG	UUAUCAUCUGAGCUUCGUUCUC	Cleavage	1
trf_7_19	Bra036771	3	20.2	1	23	5375	5397	GGGGAUGUAGCUCAGAUGGUAGA	UCGACUGGCUGAGCUACAUUCCU	Cleavage	1
trf_7_19	Bra018620	3	8.3	1	20	7	26	GGGGAUGUAGCUCAGAUGGU	GCCACCUCAGCUACAUCCUC	Cleavage	1
trf_7_25	Bra005511	2.5	12.8	1	20	1115	1134	GUGGCUGUAGUUUAGUGGUG	CACCGCUGAACUAUGGCUAC	Cleavage	1
trf_7_25	Bra009047	3	12.1	1	20	243	262	GUGGCUGUAGUUUAGUGGUG	CAACAUUGAGCUACAGCCAU	Cleavage	1
trf_7_25	Bra035454	3	17.9	1	20	1245	1264	GUGGCUGUAGUUUAGUGGUG	CAUCUCUAGGCUACAGCCAU	Cleavage	1

trf_7_34	Bra009876	3	14.9	1	22	16	37	GGGGAUGUAGCUCAGAUGGUAG	UUAUCAUCUGGGCUUCGUCCUC	Cleavage	1
trf_7_34	Bra016141	3	10.4	1	22	3031	3052	GGGGAUGUAGCUCAGAUGGUAG	UUAUCAUCUGAGCUUCGUUCUC	Cleavage	1
trf_7_34	Bra036771	3	20.2	1	23	5375	5397	GGGGAUGUAGCUCAGAUGGUAGA	UCGACUGGCUGAGCUACAUUCCU	Cleavage	1
trf_7_34	Bra018620	3	8.3	1	20	7	26	GGGGAUGUAGCUCAGAUGGU	GCCACCUCAGCUACAUCCUC	Cleavage	1
trf_7_37	Bra024638	3	7.5	1	23	203	225	GGGGAUGUAGCUCAAAUGGUAGA	UCCAACAUUUCGGUUACAUCCCC	Cleavage	1
trf_7_37	Bra025904	3	17.2	1	20	344	363	GGGGAUGUAGCUCAAAUGGU	ACAAUUUGAGCUUCAUCUUC	Cleavage	1
trf_7_47	Bra009876	3	14.9	1	22	16	37	GGGGAUGUAGCUCAGAUGGUAG	UUAUCAUCUGGGCUUCGUCCUC	Cleavage	1
trf_7_47	Bra016141	3	10.4	1	22	3031	3052	GGGGAUGUAGCUCAGAUGGUAG	UUAUCAUCUGAGCUUCGUUCUC	Cleavage	1
trf_7_47	Bra036771	3	20.2	1	23	5375	5397	GGGGAUGUAGCUCAGAUGGUAGA	UCGACUGGCUGAGCUACAUUCCU	Cleavage	1
trf_7_47	Bra018620	3	8.3	1	20	7	26	GGGGAUGUAGCUCAGAUGGU	GCCACCUCAGCUACAUCCUC	Cleavage	1
trf_7_48	Bra013584	3	16.4	1	21	890	910	UCCGUUGUCGUCCAGCGGUUA	UAACCGAUGGAUGUCAAUGGA	Cleavage	1
trf_7_48	Bra000529	3	11.2	1	21	984	1004	UCCGUUGUCGUCCAGCGGUUA	UAACCGGAGGACGAUAAUGGA	Cleavage	1
trf_7_49	Bra013584	3	16.4	1	26	885	910	UCCGUUGUCGUCCAGCGGUUAGGAUA	UAUGAUAACCGAUGGAUGUCAAUGGA	Cleavage	1
trf_7_49	Bra000529	3	11.2	1	21	984	1004	UCCGUUGUCGUCCAGCGGUUA	UAACCGGAGGACGAUAAUGGA	Cleavage	1
trf_7_50	Bra013584	3	16.4	1	25	886	910	UCCGUUGUCGUCCAGCGGUUAGGAU	AUGAUAACCGAUGGAUGUCAAUGGA	Cleavage	1
trf_7_50	Bra000529	3	11.2	1	21	984	1004	UCCGUUGUCGUCCAGCGGUUA	UAACCGGAGGACGAUAAUGGA	Cleavage	1
trf_7_51	Bra013584	3	16.4	1	26	885	910	UCCGUUGUCGUCCAGCGGUUAGGAUA	UAUGAUAACCGAUGGAUGUCAAUGGA	Cleavage	1

trf_7_51	Bra000529	3	11.2	1	21	984	1004	UCCGUUGUCGUCCAGCGGUUA	UAACCGGAGGACGAUAAUGGA	Cleavage	1
trf_7_53	Bra013584	3	16.4	1	20	891	910	UCCGUUGUCGUCCAGCGGUU	AACCGAUGGAUGUCAAUGGA	Cleavage	1
trf_7_53	Bra000529	3	11.2	1	20	985	1004	UCCGUUGUCGUCCAGCGGUU	AACCGGAGGACGAUAAUGGA	Cleavage	1
trf_7_58	Bra005511	2.5	12.8	1	20	1115	1134	GUGGCUGUAGUUUAGUGGUG	CACCGCUGAACUAUGGCUAC	Cleavage	1
trf_7_58	Bra009047	3	12.1	1	20	243	262	GUGGCUGUAGUUUAGUGGUG	CAACAUUGAGCUACAGCCAU	Cleavage	1
trf_7_58	Bra035454	3	17.9	1	20	1245	1264	GUGGCUGUAGUUUAGUGGUG	CAUCUCUAGGCUACAGCCAU	Cleavage	1
trf_7_69	Bra024638	3	7.5	1	23	203	225	GGGGAUGUAGCUCAAAUGGUAGA	UCCAACAUUUCGGUUACAUCCCC	Cleavage	1
trf_7_69	Bra025904	3	17.2	1	20	344	363	GGGGAUGUAGCUCAAAUGGU	ACAAUUUGAGCUUCAUCUUC	Cleavage	1
trf_7_70	Bra014507	3	17.0	1	23	541	563	GGGGAUGUAGCUCAUAUGGUAGA	UGUAUUAUAUUAGCUAUAUCUCC	Cleavage	1
trf_7_71	Bra024638	3	7.5	1	24	202	225	GGGGAUGUAGCUCAAAUGGUAGAG	UUCCAACAUUUCGGUUACAUCCCC	Cleavage	1
trf_7_71	Bra025904	3	17.2	1	20	344	363	GGGGAUGUAGCUCAAAUGGU	ACAAUUUGAGCUUCAUCUUC	Cleavage	1
trf_7_72	Bra024638	3	7.5	1	23	203	225	GGGGAUGUAGCUCAAAUGGUAGA	UCCAACAUUUCGGUUACAUCCCC	Cleavage	1
trf_7_72	Bra025904	3	17.2	1	20	344	363	GGGGAUGUAGCUCAAAUGGU	ACAAUUUGAGCUUCAUCUUC	Cleavage	1
trf_7_75	Bra024638	3	7.5	1	23	203	225	GGGGAUGUAGCUCAAAUGGUAGA	UCCAACAUUUCGGUUACAUCCCC	Cleavage	1
trf_7_75	Bra025904	3	17.2	1	20	344	363	GGGGAUGUAGCUCAAAUGGU	ACAAUUUGAGCUUCAUCUUC	Cleavage	1
trf_7_76	Bra009876	3	14.9	1	22	16	37	GGGGAUGUAGCUCAGAUGGUAG	UUAUCAUCUGGGCUUCGUCCUC	Cleavage	1
trf_7_76	Bra016141	3	10.4	1	22	3031	3052	GGGGAUGUAGCUCAGAUGGUAG	UUAUCAUCUGAGCUUCGUUCUC	Cleavage	1

trf_7_76	Bra036771	3	20.2	1	23	5375	5397	GGGGAUGUAGCUCAGAUGGUAGA	UCGACUGGCUGAGCUACAUUCCU	Cleavage	1
trf_7_76	Bra018620	3	8.3	1	20	7	26	GGGGAUGUAGCUCAGAUGGU	GCCACCUCAGCUACAUCCUC	Cleavage	1
trf_7_82	Bra005511	2.5	12.8	1	22	1113	1134	GUGGCUGUAGUUUAGUGGUGAG	CUCACCGCUGAACUAUGGCUAC	Cleavage	1
trf_7_82	Bra035454	3	17.9	1	22	1243	1264	GUGGCUGUAGUUUAGUGGUGAG	UUCAUCUCUAGGCUACAGCCAU	Cleavage	1
trf_7_82	Bra009047	3	12.1	1	20	243	262	GUGGCUGUAGUUUAGUGGUG	CAACAUUGAGCUACAGCCAU	Cleavage	1
trf_7_86	Bra009876	3	14.9	1	22	16	37	GGGGAUGUAGCUCAGAUGGUAG	UUAUCAUCUGGGCUUCGUCCUC	Cleavage	1
trf_7_86	Bra016141	3	10.4	1	22	3031	3052	GGGGAUGUAGCUCAGAUGGUAG	UUAUCAUCUGAGCUUCGUUCUC	Cleavage	1
trf_7_86	Bra036771	3	20.2	1	23	5375	5397	GGGGAUGUAGCUCAGAUGGUAGA	UCGACUGGCUGAGCUACAUUCCU	Cleavage	1
trf_7_86	Bra018620	3	8.3	1	20	7	26	GGGGAUGUAGCUCAGAUGGU	GCCACCUCAGCUACAUCCUC	Cleavage	1
trf_7_87	Bra009876	3	14.9	1	22	16	37	GGGGAUGUAGCUCAGAUGGUAG	UUAUCAUCUGGGCUUCGUCCUC	Cleavage	1
trf_7_87	Bra016141	3	10.4	1	22	3031	3052	GGGGAUGUAGCUCAGAUGGUAG	UUAUCAUCUGAGCUUCGUUCUC	Cleavage	1
trf_7_87	Bra036771	3	20.2	1	23	5375	5397	GGGGAUGUAGCUCAGAUGGUAGA	UCGACUGGCUGAGCUACAUUCCU	Cleavage	1
trf_7_87	Bra018620	3	8.3	1	20	7	26	GGGGAUGUAGCUCAGAUGGU	GCCACCUCAGCUACAUCCUC	Cleavage	1
trf_7_88	Bra009876	3	14.9	1	22	16	37	GGGGAUGUAGCUCAGAUGGUAG	UUAUCAUCUGGGCUUCGUCCUC	Cleavage	1
trf_7_88	Bra016141	3	10.4	1	22	3031	3052	GGGGAUGUAGCUCAGAUGGUAG	UUAUCAUCUGAGCUUCGUUCUC	Cleavage	1
trf_7_88	Bra036771	3	20.2	1	23	5375	5397	GGGGAUGUAGCUCAGAUGGUAGA	UCGACUGGCUGAGCUACAUUCCU	Cleavage	1
trf_7_88	Bra018620	3	8.3	1	20	7	26	GGGGAUGUAGCUCAGAUGGU	GCCACCUCAGCUACAUCCUC	Cleavage	1

trf_7_9	Bra009		14					GGGGAUGUAGCUCAGA	UUAUCAUCUGGGCUUC	Cleava	
5	876	3	.9	1	22	16	37	UGGUAG	GUCCUC	ge	1
trf_7_9	Bra016		10					GGGGAUGUAGCUCAGA	UUAUCAUCUGAGCUUC	Cleava	
5	141	3	.4	1	22	3031	3052	UGGUAG	GUUCUC	ge	1
trf_7_9	Bra036		20					GGGGAUGUAGCUCAGA	UCGACUGGCUGAGCUA	Cleava	
5	771	3	.2	1	23	5375	5397	UGGUAGA	CAUUCCU	ge	1
trf_7_9	Bra018		8.					GGGGAUGUAGCUCAGA	GCCACCUCAGCUACAUC	Cleava	
5	620	3	3	1	20	7	26	UGGU	CUC	ge	1
trf_7_1	Bra000		11					UCCGUUAUCGUCCAGC	UAACCGGAGGACGAUA	Cleava	
07	529	2.5	.2	1	21	984	1004	GGUUA	AUGGA	ge	1
trf_7_1	Bra032		22					UCCGUUAUCGUCCAGC	GACGGCUUGACGAUGA	Cleava	
07	111	3	.4	1	20	727	746	GGUU	CGGA	ge	1
trf_7_1	Bra000		11					UCCGUUAUCGUCCAGC	UAACCGGAGGACGAUA	Cleava	
08	529	2.5	.2	1	21	984	1004	GGUUA	AUGGA	ge	1
trf_7_1	Bra032		22					UCCGUUAUCGUCCAGC	GACGGCUUGACGAUGA	Cleava	
08	111	3	.4	1	20	727	746	GGUU	CGGA	ge	1
trf_7_1	Bra000		11					UCCGUUAUCGUCCAGC	UAACCGGAGGACGAUA	Cleava	
09	529	2.5	.2	1	21	984	1004	GGUUA	AUGGA	ge	1
trf_7_1	Bra032		22					UCCGUUAUCGUCCAGC	GACGGCUUGACGAUGA	Cleava	
09	111	3	.4	1	20	727	746	GGUU	CGGA	ge	1
trf_7_1	Bra000		11					UCCGUUAUCGUCCAGC	AACCGGAGGACGAUAA	Cleava	
10	529	2.5	.2	1	20	985	1004	GGUU	UGGA	ge	1
trf_7_1	Bra032		22					UCCGUUAUCGUCCAGC	GACGGCUUGACGAUGA	Cleava	
10	111	3	.4	1	20	727	746	GGUU	CGGA	ge	1
trf_7_1	Bra000		11					UCCGUUAUCGUCCAGC	UAACCGGAGGACGAUA	Cleava	
11	529	2.5	.2	1	21	984	1004	GGUUA	AUGGA	ge	1
trf_7_1	Bra032		22					UCCGUUAUCGUCCAGC	GACGGCUUGACGAUGA	Cleava	
11	111	3	.4	1	20	727	746	GGUU	CGGA	ge	1
trf_7_1	Bra000		11					UCCGUUAUCGUCCAGC	UAACCGGAGGACGAUA	Cleava	
13	529	2.5	.2	1	21	984	1004	GGUUA	AUGGA	ge	1
trf_7_1	Bra032		22					UCCGUUAUCGUCCAGC	GACGGCUUGACGAUGA	Cleava	
13	111	3	.4	1	20	727	746	GGUU	CGGA	ge	1
trf_7_1	Bra000		11					UCCGUUAUCGUCCAGC	UAACCGGAGGACGAUA	Cleava	
15	529	2.5	.2	1	21	984	1004	GGUUA	AUGGA	ge	1

trf_7_1	Bra032		22					UCCGUUAUCGUCCAGC	GACGGCUUGACGAUGA	Cleava	
15	111	3	.4	1	20	727	746	GGUU	CGGA	ge	1
trf_7_1	Bra009		14					GGGGAUGUAGCUCAGA	UUAUCAUCUGGGCUUC	Cleava	
16	876	3	.9	1	22	16	37	UGGUAG	GUCCUC	ge	1
trf_7_1	Bra016		10					GGGGAUGUAGCUCAGA	UUAUCAUCUGAGCUUC	Cleava	
16	141	3	.4	1	22	3031	3052	UGGUAG	GUUCUC	ge	1
trf_7_1	Bra036		20					GGGGAUGUAGCUCAGA	UCGACUGGCUGAGCUA	Cleava	
16	771	3	.2	1	23	5375	5397	UGGUAGA	CAUUCCU	ge	1
trf_7_1	Bra018		8.					GGGGAUGUAGCUCAGA	GCCACCUCAGCUACAUC	Cleava	
16	620	3	3	1	20	7	26	UGGU	CUC	ge	1
trf_7_1	Bra036		20					GGGGAUGUAGCUCAGA	CUCGACUGGCUGAGCU	Cleava	
24	771	3	.2	1	24	5374	5397	UGGUAGAG	ACAUUCCU	ge	1
trf_7_1	Bra009		14					GGGGAUGUAGCUCAGA	CGUUAUCAUCUGGGCU	Cleava	
24	876	3	.9	1	24	14	37	UGGUAGAG	UCGUCCUC	ge	1
trf_7_1	Bra016		10					GGGGAUGUAGCUCAGA	CAUUAUCAUCUGAGCU	Cleava	
24	141	3	.4	1	24	3029	3052	UGGUAGAG	UCGUUCUC	ge	1
trf_7_1	Bra018		8.					GGGGAUGUAGCUCAGA	GCCACCUCAGCUACAUC	Cleava	
24	620	3	3	1	20	7	26	UGGU	CUC	ge	1
trf_7_1	Bra009		14					GGGGAUGUAGCUCAGA	UUAUCAUCUGGGCUUC	Cleava	
25	876	3	.9	1	22	16	37	UGGUAG	GUCCUC	ge	1
trf_7_1	Bra016		10					GGGGAUGUAGCUCAGA	UUAUCAUCUGAGCUUC	Cleava	
25	141	3	.4	1	22	3031	3052	UGGUAG	GUUCUC	ge	1
trf_7_1	Bra036		20					GGGGAUGUAGCUCAGA	UCGACUGGCUGAGCUA	Cleava	
25	771	3	.2	1	23	5375	5397	UGGUAGA	CAUUCCU	ge	1
trf_7_1	Bra018		8.					GGGGAUGUAGCUCAGA	GCCACCUCAGCUACAUC	Cleava	
25	620	3	3	1	20	7	26	UGGU	CUC	ge	1
trf_7_1	Bra013		16					UCCGUUGUCGUCCAGC	UAACCGAUGGAUGUCA	Cleava	
31	584	3	.4	1	21	890	910	GGUUA	AUGGA	ge	1
trf_7_1	Bra000		11					UCCGUUGUCGUCCAGC	UAACCGGAGGACGAUA	Cleava	
31	529	3	.2	1	21	984	1004	GGUUA	AUGGA	ge	1
trf_7_1	Bra013		16					UCCGUUGUCGUCCAGC	UAUGAUAACCGAUGGA	Cleava	
32	584	3	.4	1	26	885	910	GGUUAGGAUA	UGUCAAUGGA	ge	1
trf_7_1	Bra000		11					UCCGUUGUCGUCCAGC	UAACCGGAGGACGAUA	Cleava	
32	529	3	.2	1	21	984	1004	GGUUA	AUGGA	ge	1

trf_7_1	Bra013		16					UCCGUUGUCGUCCAGC	AUGAUAACCGAUGGAU	Cleava	
33	584	3	.4	1	25	886	910	GGUUAGGAU	GUCAAUGGA	ge	1
trf_7_1	Bra000		11					UCCGUUGUCGUCCAGC	UAACCGGAGGACGAUA	Cleava	
33	529	3	.2	1	21	984	1004	GGUUA	AUGGA	ge	1
trf_7_1	Bra013		16					UCCGUUGUCGUCCAGC	AACCGAUGGAUGUCAA	Cleava	
34	584	3	.4	1	20	891	910	GGUU	UGGA	ge	1
trf_7_1	Bra000		11					UCCGUUGUCGUCCAGC	AACCGGAGGACGAUAA	Cleava	
34	529	3	.2	1	20	985	1004	GGUU	UGGA	ge	1
trf_7_1	Bra013		16					UCCGUUGUCGUCCAGC	UAUGAUAACCGAUGGA	Cleava	
35	584	3	.4	1	26	885	910	GGUUAGGAUA	UGUCAAUGGA	ge	1
trf_7_1	Bra000		11					UCCGUUGUCGUCCAGC	UAACCGGAGGACGAUA	Cleava	
35	529	3	.2	1	21	984	1004	GGUUA	AUGGA	ge	1
trf_7_1	Bra013		16					UCCGUUGUCGUCCAGC	UAACCGAUGGAUGUCA	Cleava	
37	584	3	.4	1	21	890	910	GGUUA	AUGGA	ge	1
trf_7_1	Bra000		11					UCCGUUGUCGUCCAGC	UAACCGGAGGACGAUA	Cleava	
37	529	3	.2	1	21	984	1004	GGUUA	AUGGA	ge	1
trf_7_1	Bra009		14					GGGGAUGUAGCUCAGA	UUAUCAUCUGGGCUUC	Cleava	
38	876	3	.9	1	22	16	37	UGGUAG	GUCCUC	ge	1
trf_7_1	Bra016		10					GGGGAUGUAGCUCAGA	UUAUCAUCUGAGCUUC	Cleava	
38	141	3	.4	1	22	3031	3052	UGGUAG	GUUCUC	ge	1
trf_7_1	Bra036		20					GGGGAUGUAGCUCAGA	UCGACUGGCUGAGCUA	Cleava	
38	771	3	.2	1	23	5375	5397	UGGUAGA	CAUUCCU	ge	1
trf_7_1	Bra018		8.					GGGGAUGUAGCUCAGA	GCCACCUCAGCUACAUC	Cleava	
38	620	3	3	1	20	7	26	UGGU	CUC	ge	1
trf_7_1	Bra009		14					GGGGAUGUAGCUCAGA	UUAUCAUCUGGGCUUC	Cleava	
39	876	3	.9	1	22	16	37	UGGUAG	GUCCUC	ge	1
trf_7_1	Bra016		10					GGGGAUGUAGCUCAGA	UUAUCAUCUGAGCUUC	Cleava	
39	141	3	.4	1	22	3031	3052	UGGUAG	GUUCUC	ge	1
trf_7_1	Bra036		20					GGGGAUGUAGCUCAGA	UCGACUGGCUGAGCUA	Cleava	
39	771	3	.2	1	23	5375	5397	UGGUAGA	CAUUCCU	ge	1
trf_7_1	Bra018		8.					GGGGAUGUAGCUCAGA	GCCACCUCAGCUACAUC	Cleava	
39	620	3	3	1	20	7	26	UGGU	CUC	ge	1
trf_7_1	Bra013		16					UCCGUUGUCGUCCAGC	UAACCGAUGGAUGUCA	Cleava	
40	584	3	.4	1	21	890	910	GGUUA	AUGGA	ge	1

trf_7_1 40	Bra000 529	3	11 .2	1	21	984	1004	UCCGUUGUCGUCCAGC GGUUA	UAACCGGAGGACGAUA AUGGA	Cleava ge	1
trf_7_1 41	Bra013 584	3	16 .4	1	20	891	910	UCCGUUGUCGUCCAGC GGUU	AACCGAUGGAUGUCAA UGGA	Cleava ge	1
trf_7_1 41	Bra000 529	3	11 .2	1	20	985	1004	UCCGUUGUCGUCCAGC GGUU	AACCGGAGGACGAUAA UGGA	Cleava ge	1
trf_7_1 42	Bra013 584	3	16 .4	1	25	886	910	UCCGUUGUCGUCCAGC GGUUAGGAU	AUGAUAACCGAUGGAU GUCAAUGGA	Cleava ge	1
trf_7_1 42	Bra000 529	3	11 .2	1	21	984	1004	UCCGUUGUCGUCCAGC GGUUA	UAACCGGAGGACGAUA AUGGA	Cleava ge	1
trf_7_1 43	Bra013 584	3	16 .4	1	26	885	910	UCCGUUGUCGUCCAGC GGUUAGGAUA	UAUGAUAACCGAUGGA UGUCAAUGGA	Cleava ge	1
trf_7_1 43	Bra000 529	3	11 .2	1	21	984	1004	UCCGUUGUCGUCCAGC GGUUA	UAACCGGAGGACGAUA AUGGA	Cleava ge	1
trf_7_1 44	Bra013 584	3	16 .4	1	21	890	910	UCCGUUGUCGUCCAGC GGUUA	UAACCGAUGGAUGUCA AUGGA	Cleava ge	1
trf_7_1 44	Bra000 529	3	11 .2	1	21	984	1004	UCCGUUGUCGUCCAGC GGUUA	UAACCGGAGGACGAUA AUGGA	Cleava ge	1
trf_7_1 45	Bra013 584	3	16 .4	1	21	890	910	UCCGUUGUCGUCCAGC GGUUA	UAACCGAUGGAUGUCA AUGGA	Cleava ge	1
trf_7_1 45	Bra000 529	3	11 .2	1	21	984	1004	UCCGUUGUCGUCCAGC GGUUA	UAACCGGAGGACGAUA AUGGA	Cleava ge	1
trf_7_1 46	Bra014 507	3	17 .0	1	23	541	563	GGGGAUGUAGCUCAUA UGGUAGA	UGUAUUAUAUUAGCU AUAUCUCC	Cleava ge	1
trf_7_1 47	Bra016 601	3	20 .8	1	24	415	438	UCCGUCGUAGUCUAGC UGGUUAGG	CUGAAGAAGCUAGGCU GCGACGGA	Cleava ge	1
trf_7_1 48	Bra016 601	3	20 .8	1	24	415	438	UCCGUCGUAGUCUAGC UGGUUAGG	CUGAAGAAGCUAGGCU GCGACGGA	Cleava ge	1
trf_7_1 50	Bra016 601	3	20 .8	1	24	415	438	UCCGUCGUAGUCUAGC UGGUUAGG	CUGAAGAAGCUAGGCU GCGACGGA	Cleava ge	1
trf_7_1 51	Bra005 511	2.5	12 .8	1	22	1113	1134	GUGGCUGUAGUUUAG UGGUGAG	CUCACCGCUGAACUAU GGCUAC	Cleava ge	1
trf_7_1 51	Bra035 454	3	17 .9	1	24	1241	1264	GUGGCUGUAGUUUAG UGGUGAGAA	UAUUCAUCUCUAGGCU ACAGCCAU	Cleava ge	1

trf_7_1	Bra009		12					GUGGCUGUAGUUUAG	CAACAUUGAGCUACAG	Cleava	
51	047	3	.1	1	20	243	262	UGGUG	CCAU	ge	1
trf_7_1	Bra009		14					GGGGAUGUAGCUCAGA	UUAUCAUCUGGGCUUC	Cleava	
52	876	3	.9	1	22	16	37	UGGUAG	GUCCUC	ge	1
trf_7_1	Bra016		10					GGGGAUGUAGCUCAGA	UUAUCAUCUGAGCUUC	Cleava	
52	141	3	.4	1	22	3031	3052	UGGUAG	GUUCUC	ge	1
trf_7_1	Bra036		20					GGGGAUGUAGCUCAGA	UCGACUGGCUGAGCUA	Cleava	
52	771	3	.2	1	23	5375	5397	UGGUAGA	CAUUCCU	ge	1
trf_7_1	Bra018		8.					GGGGAUGUAGCUCAGA	GCCACCUCAGCUACAUC	Cleava	
52	620	3	3	1	20	7	26	UGGU	CUC	ge	1
trf_7_1	Bra009		14					GGGGAUGUAGCUCAGA	UUAUCAUCUGGGCUUC	Cleava	
55	876	3	.9	1	22	16	37	UGGUAG	GUCCUC	ge	1
trf_7_1	Bra016		10					GGGGAUGUAGCUCAGA	UUAUCAUCUGAGCUUC	Cleava	
55	141	3	.4	1	22	3031	3052	UGGUAG	GUUCUC	ge	1
trf_7_1	Bra036		20					GGGGAUGUAGCUCAGA	UCGACUGGCUGAGCUA	Cleava	
55	771	3	.2	1	23	5375	5397	UGGUAGA	CAUUCCU	ge	1
trf_7_1	Bra018		8.					GGGGAUGUAGCUCAGA	GCCACCUCAGCUACAUC	Cleava	
55	620	3	3	1	20	7	26	UGGU	CUC	ge	1
trf_7_1	Bra009		14					GGGGAUGUAGCUCAGA	UUAUCAUCUGGGCUUC	Cleava	
56	876	3	.9	1	22	16	37	UGGUAG	GUCCUC	ge	1
trf_7_1	Bra016		10					GGGGAUGUAGCUCAGA	UUAUCAUCUGAGCUUC	Cleava	
56	141	3	.4	1	22	3031	3052	UGGUAG	GUUCUC	ge	1
trf_7_1	Bra036		20					GGGGAUGUAGCUCAGA	UCGACUGGCUGAGCUA	Cleava	
56	771	3	.2	1	23	5375	5397	UGGUAGA	CAUUCCU	ge	1
trf_7_1	Bra018		8.					GGGGAUGUAGCUCAGA	GCCACCUCAGCUACAUC	Cleava	
56	620	3	3	1	20	7	26	UGGU	CUC	ge	1
trf_7_1	Bra009		14					GGGGAUGUAGCUCAGA	UUAUCAUCUGGGCUUC	Cleava	
57	876	3	.9	1	22	16	37	UGGUAG	GUCCUC	ge	1
trf_7_1	Bra016		10					GGGGAUGUAGCUCAGA	UUAUCAUCUGAGCUUC	Cleava	
57	141	3	.4	1	22	3031	3052	UGGUAG	GUUCUC	ge	1
trf_7_1	Bra036		20					GGGGAUGUAGCUCAGA	UCGACUGGCUGAGCUA	Cleava	
57	771	3	.2	1	23	5375	5397	UGGUAGA	CAUUCCU	ge	1
trf_7_1	Bra018		8.					GGGGAUGUAGCUCAGA	GCCACCUCAGCUACAUC	Cleava	
57	620	3	3	1	20	7	26	UGGU	CUC	ge	1

trf_7_160	Bra009876	3	14.9	1	22	16	37	GGGGAUGUAGCUCAGAUGGUAG	UUAUCAUCUGGGCUUCGUCCUC	Cleavage	1
trf_7_160	Bra016141	3	10.4	1	22	3031	3052	GGGGAUGUAGCUCAGAUGGUAG	UUAUCAUCUGAGCUUCGUUCUC	Cleavage	1
trf_7_160	Bra036771	3	20.2	1	23	5375	5397	GGGGAUGUAGCUCAGAUGGUAGA	UCGACUGGCUGAGCUACAUUCCU	Cleavage	1
trf_7_160	Bra018620	3	8.3	1	20	7	26	GGGGAUGUAGCUCAGAUGGU	GCCACCUCAGCUACAUCCUC	Cleavage	1
trf_7_162	Bra009876	3	14.9	1	22	16	37	GGGGAUGUAGCUCAGAUGGUAG	UUAUCAUCUGGGCUUCGUCCUC	Cleavage	1
trf_7_162	Bra016141	3	10.4	1	22	3031	3052	GGGGAUGUAGCUCAGAUGGUAG	UUAUCAUCUGAGCUUCGUUCUC	Cleavage	1
trf_7_162	Bra036771	3	20.2	1	23	5375	5397	GGGGAUGUAGCUCAGAUGGUAGA	UCGACUGGCUGAGCUACAUUCCU	Cleavage	1
trf_7_162	Bra018620	3	8.3	1	20	7	26	GGGGAUGUAGCUCAGAUGGU	GCCACCUCAGCUACAUCCUC	Cleavage	1
trf_7_163	Bra024638	3	7.5	1	23	203	225	GGGGAUGUAGCUCAAAUGGUAGA	UCCAACAUUUCGGUUACAUCCCC	Cleavage	1
trf_7_163	Bra025904	3	17.2	1	20	344	363	GGGGAUGUAGCUCAAAUGGU	ACAAUUUGAGCUUCAUCUUC	Cleavage	1
trf_7_164	Bra009876	3	14.9	1	22	16	37	GGGGAUGUAGCUCAGAUGGUAG	UUAUCAUCUGGGCUUCGUCCUC	Cleavage	1
trf_7_164	Bra016141	3	10.4	1	22	3031	3052	GGGGAUGUAGCUCAGAUGGUAG	UUAUCAUCUGAGCUUCGUUCUC	Cleavage	1
trf_7_164	Bra036771	3	20.2	1	23	5375	5397	GGGGAUGUAGCUCAGAUGGUAGA	UCGACUGGCUGAGCUACAUUCCU	Cleavage	1
trf_7_164	Bra018620	3	8.3	1	20	7	26	GGGGAUGUAGCUCAGAUGGU	GCCACCUCAGCUACAUCCUC	Cleavage	1
trf_7_170	Bra009876	3	14.9	1	22	16	37	GGGGAUGUAGCUCAGAUGGUAG	UUAUCAUCUGGGCUUCGUCCUC	Cleavage	1
trf_7_170	Bra016141	3	10.4	1	22	3031	3052	GGGGAUGUAGCUCAGAUGGUAG	UUAUCAUCUGAGCUUCGUUCUC	Cleavage	1
trf_7_170	Bra036771	3	20.2	1	23	5375	5397	GGGGAUGUAGCUCAGAUGGUAGA	UCGACUGGCUGAGCUACAUUCCU	Cleavage	1

trf_7_170	Bra018620	3	8.3	1	20	7	26	GGGGAUGUAGCUCAGAUGGU	GCCACCUCAGCUACAUCCUC	Cleavage	1
trf_7_172	Bra009876	3	14.9	1	22	16	37	GGGGAUGUAGCUCAGAUGGUAG	UUAUCAUCUGGGCUUCGUCCUC	Cleavage	1
trf_7_172	Bra016141	3	10.4	1	22	3031	3052	GGGGAUGUAGCUCAGAUGGUAG	UUAUCAUCUGAGCUUCGUUCUC	Cleavage	1
trf_7_172	Bra036771	3	20.2	1	23	5375	5397	GGGGAUGUAGCUCAGAUGGUAGA	UCGACUGGCUGAGCUACAUUCCU	Cleavage	1
trf_7_172	Bra018620	3	8.3	1	20	7	26	GGGGAUGUAGCUCAGAUGGU	GCCACCUCAGCUACAUCCUC	Cleavage	1
trf_7_173	Bra036771	3	20.2	1	24	5374	5397	GGGGAUGUAGCUCAGAUGGUAGAG	CUCGACUGGCUGAGCUACAUUCCU	Cleavage	1
trf_7_173	Bra009876	3	14.9	1	24	14	37	GGGGAUGUAGCUCAGAUGGUAGAG	CGUUAUCAUCUGGGCUUCGUCCUC	Cleavage	1
trf_7_173	Bra016141	3	10.4	1	24	3029	3052	GGGGAUGUAGCUCAGAUGGUAGAG	CAUUAUCAUCUGAGCUUCGUUCUC	Cleavage	1
trf_7_173	Bra018620	3	8.3	1	20	7	26	GGGGAUGUAGCUCAGAUGGU	GCCACCUCAGCUACAUCCUC	Cleavage	1
trf_7_174	Bra024638	3	7.5	1	23	203	225	GGGGAUGUAGCUCAAAUGGUAGA	UCCAACAUUUCGGUUACAUCCCC	Cleavage	1
trf_7_174	Bra025904	3	17.2	1	20	344	363	GGGGAUGUAGCUCAAAUGGU	ACAAUUUGAGCUUCAUCUUC	Cleavage	1
trf_7_175	Bra009876	3	14.9	1	22	16	37	GGGGAUGUAGCUCAGAUGGUAG	UUAUCAUCUGGGCUUCGUCCUC	Cleavage	1
trf_7_175	Bra016141	3	10.4	1	22	3031	3052	GGGGAUGUAGCUCAGAUGGUAG	UUAUCAUCUGAGCUUCGUUCUC	Cleavage	1
trf_7_175	Bra036771	3	20.2	1	23	5375	5397	GGGGAUGUAGCUCAGAUGGUAGA	UCGACUGGCUGAGCUACAUUCCU	Cleavage	1
trf_7_175	Bra018620	3	8.3	1	20	7	26	GGGGAUGUAGCUCAGAUGGU	GCCACCUCAGCUACAUCCUC	Cleavage	1
trf_7_176	Bra009876	3	14.9	1	22	16	37	GGGGAUGUAGCUCAGAUGGUAG	UUAUCAUCUGGGCUUCGUCCUC	Cleavage	1
trf_7_176	Bra016141	3	10.4	1	22	3031	3052	GGGGAUGUAGCUCAGAUGGUAG	UUAUCAUCUGAGCUUCGUUCUC	Cleavage	1

trf_7_176	Bra036771	3	20.2	1	23	5375	5397	GGGGAUGUAGCUCAGAUGGUAGA	UCGACUGGCUGAGCUACAUUCCU	Cleavage	1
trf_7_176	Bra018620	3	8.3	1	20	7	26	GGGGAUGUAGCUCAGAUGGU	GCCACCUCAGCUACAUCCUC	Cleavage	1
trf_7_177	Bra014507	3	17.0	1	23	541	563	GGGGAUGUAGCUCAUAUGGUAGA	UGUAUUAUAUUAGCUAUAUCUCC	Cleavage	1
trf_7_178	Bra009876	3	14.9	1	22	16	37	GGGGAUGUAGCUCAGAUGGUAG	UUAUCAUCUGGGCUUCGUCCUC	Cleavage	1
trf_7_178	Bra016141	3	10.4	1	22	3031	3052	GGGGAUGUAGCUCAGAUGGUAG	UUAUCAUCUGAGCUUCGUUCUC	Cleavage	1
trf_7_178	Bra036771	3	20.2	1	23	5375	5397	GGGGAUGUAGCUCAGAUGGUAGA	UCGACUGGCUGAGCUACAUUCCU	Cleavage	1
trf_7_178	Bra018620	3	8.3	1	20	7	26	GGGGAUGUAGCUCAGAUGGU	GCCACCUCAGCUACAUCCUC	Cleavage	1
trf_7_179	Bra009876	3	14.9	1	22	16	37	GGGGAUGUAGCUCAGAUGGUAG	UUAUCAUCUGGGCUUCGUCCUC	Cleavage	1
trf_7_179	Bra016141	3	10.4	1	22	3031	3052	GGGGAUGUAGCUCAGAUGGUAG	UUAUCAUCUGAGCUUCGUUCUC	Cleavage	1
trf_7_179	Bra036771	3	20.2	1	23	5375	5397	GGGGAUGUAGCUCAGAUGGUAGA	UCGACUGGCUGAGCUACAUUCCU	Cleavage	1
trf_7_179	Bra018620	3	8.3	1	20	7	26	GGGGAUGUAGCUCAGAUGGU	GCCACCUCAGCUACAUCCUC	Cleavage	1
trf_7_180	Bra009876	3	14.9	1	22	16	37	GGGGAUGUAGCUCAGAUGGUAG	UUAUCAUCUGGGCUUCGUCCUC	Cleavage	1
trf_7_180	Bra016141	3	10.4	1	22	3031	3052	GGGGAUGUAGCUCAGAUGGUAG	UUAUCAUCUGAGCUUCGUUCUC	Cleavage	1
trf_7_180	Bra036771	3	20.2	1	23	5375	5397	GGGGAUGUAGCUCAGAUGGUAGA	UCGACUGGCUGAGCUACAUUCCU	Cleavage	1
trf_7_180	Bra018620	3	8.3	1	20	7	26	GGGGAUGUAGCUCAGAUGGU	GCCACCUCAGCUACAUCCUC	Cleavage	1
trf_7_181	Bra003753	1.5	16.2	1	20	284	303	GGGAUUGUAGUUCAAUUGGU	ACCAGUUGAAUUACAAUCCU	Cleavage	1
trf_7_181	Bra013336	3	19.5	1	20	267	286	GGGAUUGUAGUUCAAUUGGU	ACUAAUUGAACUAAGAUCUC	Cleavage	1

trf_7_1<br>81	Bra021<br>314	3	23<br>.2	1	20	234	253	GGGAUUGUAGUUCAA<br>UUGGU	ACCAACUGAACUGCAA<br>UCUG	Cleava<br>ge	1
trf_7_1<br>82	Bra003<br>753	2.5	16<br>.2	1	20	284	303	GGGAUUGUAGUUCAA<br>UUGGA	ACCAGUUGAAUUACAA<br>UCCU	Cleava<br>ge	1
trf_7_1<br>83	Bra003<br>753	1.5	16<br>.2	1	20	284	303	GGGAUUGUAGUUCAA<br>UUGGU	ACCAGUUGAAUUACAA<br>UCCU	Cleava<br>ge	1
trf_7_1<br>83	Bra013<br>336	3	19<br>.5	1	20	267	286	GGGAUUGUAGUUCAA<br>UUGGU	ACUAAUUGAACUAAGA<br>UCUC	Cleava<br>ge	1
trf_7_1<br>83	Bra021<br>314	3	23<br>.2	1	20	234	253	GGGAUUGUAGUUCAA<br>UUGGU	ACCAACUGAACUGCAA<br>UCUG	Cleava<br>ge	1
trf_7_1<br>84	Bra003<br>753	1.5	16<br>.2	1	20	284	303	GGGAUUGUAGUUCAA<br>UUGGU	ACCAGUUGAAUUACAA<br>UCCU	Cleava<br>ge	1
trf_7_1<br>84	Bra013<br>336	3	19<br>.5	1	20	267	286	GGGAUUGUAGUUCAA<br>UUGGU	ACUAAUUGAACUAAGA<br>UCUC	Cleava<br>ge	1
trf_7_1<br>84	Bra021<br>314	3	23<br>.2	1	20	234	253	GGGAUUGUAGUUCAA<br>UUGGU	ACCAACUGAACUGCAA<br>UCUG	Cleava<br>ge	1
trf_7_1<br>85	Bra003<br>753	2.5	16<br>.2	1	20	284	303	GGGAUUGUAGUUCAA<br>UCGGU	ACCAGUUGAAUUACAA<br>UCCU	Cleava<br>ge	1
trf_7_1<br>87	Bra003<br>753	2.5	16<br>.2	1	20	284	303	GGGAUUGUAGUUCAA<br>UUGGA	ACCAGUUGAAUUACAA<br>UCCU	Cleava<br>ge	1
trf_7_1<br>88	Bra003<br>753	2.5	16<br>.2	1	20	284	303	GGGAUUGUAGUUCAA<br>UCGGU	ACCAGUUGAAUUACAA<br>UCCU	Cleava<br>ge	1
trf_7_1<br>89	Bra003<br>753	1.5	16<br>.2	1	20	284	303	GGGAUUGUAGUUCAA<br>UUGGU	ACCAGUUGAAUUACAA<br>UCCU	Cleava<br>ge	1
trf_7_1<br>89	Bra013<br>336	3	19<br>.5	1	20	267	286	GGGAUUGUAGUUCAA<br>UUGGU	ACUAAUUGAACUAAGA<br>UCUC	Cleava<br>ge	1
trf_7_1<br>89	Bra021<br>314	3	23<br>.2	1	20	234	253	GGGAUUGUAGUUCAA<br>UUGGU	ACCAACUGAACUGCAA<br>UCUG	Cleava<br>ge	1
trf_7_1<br>90	Bra003<br>753	1.5	16<br>.2	1	20	284	303	GGGAUUGUAGUUCAA<br>UUGGU	ACCAGUUGAAUUACAA<br>UCCU	Cleava<br>ge	1
trf_7_1<br>90	Bra013<br>336	3	19<br>.5	1	20	267	286	GGGAUUGUAGUUCAA<br>UUGGU	ACUAAUUGAACUAAGA<br>UCUC	Cleava<br>ge	1
trf_7_1<br>90	Bra021<br>314	3	23<br>.2	1	20	234	253	GGGAUUGUAGUUCAA<br>UUGGU	ACCAACUGAACUGCAA<br>UCUG	Cleava<br>ge	1

trf_7_1	Bra003		16					GGGAUUGUAGUUCAA	ACCAGUUGAAUUACAA	Cleava	
91	753	1.5	.2	1	20	284	303	UUGGU	UCCU	ge	1
trf_7_1	Bra013		19					GGGAUUGUAGUUCAA	ACUAAUUGAACUAAGA	Cleava	
91	336	3	.5	1	20	267	286	UUGGU	UCUC	ge	1
trf_7_1	Bra021		23					GGGAUUGUAGUUCAA	ACCAACUGAACUGCAA	Cleava	
91	314	3	.2	1	20	234	253	UUGGU	UCUG	ge	1
trf_7_1	Bra003		16					GGGAUUGUAGUUCAA	ACCAGUUGAAUUACAA	Cleava	
92	753	1.5	.2	1	20	284	303	UUGGU	UCCU	ge	1
trf_7_1	Bra013		19					GGGAUUGUAGUUCAA	ACUAAUUGAACUAAGA	Cleava	
92	336	3	.5	1	20	267	286	UUGGU	UCUC	ge	1
trf_7_1	Bra021		23					GGGAUUGUAGUUCAA	ACCAACUGAACUGCAA	Cleava	
92	314	3	.2	1	20	234	253	UUGGU	UCUG	ge	1
trf_7_1	Bra003		16					GGGAUUGUAGUUCAA	ACCAGUUGAAUUACAA	Cleava	
93	753	2.5	.2	1	20	284	303	UUGGA	UCCU	ge	1
trf_7_1	Bra003		16					NGGAUUGUAGUUCAA	ACCAGUUGAAUUACAA	Cleava	
94	753	2	.2	1	20	284	303	UUGGU	UCCU	ge	1
trf_7_1	Bra021		23					NGGAUUGUAGUUCAA	ACCAACUGAACUGCAA	Cleava	
94	314	3	.2	1	20	234	253	UUGGU	UCUG	ge	1
trf_7_1	Bra003		16					GGGAUUGUAGUUCAA	AACCAGUUGAAUUACA	Cleava	
95	753	2.5	.2	1	21	283	303	UUGGAU	AUCCU	ge	1
trf_7_1	Bra003		16					GGGAUUGUAGUUCAA	ACCAGUUGAAUUACAA	Cleava	
96	753	1.5	.2	1	20	284	303	UUGGU	UCCU	ge	1
trf_7_1	Bra013		19					GGGAUUGUAGUUCAA	ACUAAUUGAACUAAGA	Cleava	
96	336	3	.5	1	20	267	286	UUGGU	UCUC	ge	1
trf_7_1	Bra021		23					GGGAUUGUAGUUCAA	ACCAACUGAACUGCAA	Cleava	
96	314	3	.2	1	20	234	253	UUGGU	UCUG	ge	1
trf_7_2	Bra014		17					GGGGAUGUAGCUCAUA	UGUAUUAUAUUAGCU	Cleava	
00	507	3	.0	1	23	541	563	UGGUAGA	AUAUCUCC	ge	1
trf_7_2	Bra014		17					GGGGAUGUAGCUCAUA	UUGUAUUAUAUUAGC	Cleava	
01	507	3	.0	1	24	540	563	UGGUAGAG	UAUAUCUCC	ge	1
trf_7_2	Bra009		14					GGGGAUGUAGCUCAGA	UUAUCAUCUGGGCUUC	Cleava	
02	876	3	.9	1	22	16	37	UGGUAG	GUCCUC	ge	1
trf_7_2	Bra016		10					GGGGAUGUAGCUCAGA	UUAUCAUCUGAGCUUC	Cleava	
02	141	3	.4	1	22	3031	3052	UGGUAG	GUUCUC	ge	1

trf_7_202	Bra036771	3	20.2	1	23	5375	5397	GGGGAUGUAGCUCAGAUGGUAGA	UCGACUGGCUGAGCUACAUUCCU	Cleavage	1
trf_7_202	Bra018620	3	8.3	1	20	7	26	GGGGAUGUAGCUCAGAUGGU	GCCACCUCAGCUACAUCCUC	Cleavage	1
trf_7_203	Bra009876	3	14.9	1	22	16	37	GGGGAUGUAGCUCAGAUGGUAG	UUAUCAUCUGGGCUUCGUCCUC	Cleavage	1
trf_7_203	Bra016141	3	10.4	1	22	3031	3052	GGGGAUGUAGCUCAGAUGGUAG	UUAUCAUCUGAGCUUCGUUCUC	Cleavage	1
trf_7_203	Bra036771	3	20.2	1	23	5375	5397	GGGGAUGUAGCUCAGAUGGUAGA	UCGACUGGCUGAGCUACAUUCCU	Cleavage	1
trf_7_203	Bra018620	3	8.3	1	20	7	26	GGGGAUGUAGCUCAGAUGGU	GCCACCUCAGCUACAUCCUC	Cleavage	1
trf_7_204	Bra014507	3	17.0	1	23	541	563	GGGGAUGUAGCUCAUAUGGUAGA	UGUAUUAUAUUAGCUAUAUCUCC	Cleavage	1
trf_7_205	Bra014507	3	17.0	1	24	540	563	GGGGAUGUAGCUCAUAUGGUAGAG	UUGUAUUAUAUUAGCUAUAUCUCC	Cleavage	1
trf_7_206	Bra009876	3	14.9	1	22	16	37	GGGGAUGUAGCUCAGAUGGUAG	UUAUCAUCUGGGCUUCGUCCUC	Cleavage	1
trf_7_206	Bra016141	3	10.4	1	22	3031	3052	GGGGAUGUAGCUCAGAUGGUAG	UUAUCAUCUGAGCUUCGUUCUC	Cleavage	1
trf_7_206	Bra036771	3	20.2	1	23	5375	5397	GGGGAUGUAGCUCAGAUGGUAGA	UCGACUGGCUGAGCUACAUUCCU	Cleavage	1
trf_7_206	Bra018620	3	8.3	1	20	7	26	GGGGAUGUAGCUCAGAUGGU	GCCACCUCAGCUACAUCCUC	Cleavage	1
trf_7_211	Bra013584	3	16.4	1	25	886	910	UCCGUUGUCGUCCAGCGGUUAGGAU	AUGAUAACCGAUGGAUGUCAAUGGA	Cleavage	1
trf_7_211	Bra000529	3	11.2	1	21	984	1004	UCCGUUGUCGUCCAGCGGUUA	UAACCGGAGGACGAUAAUGGA	Cleavage	1
trf_7_212	Bra013584	3	16.4	1	26	885	910	UCCGUUGUCGUCCAGCGGUUAGGAUA	UAUGAUAACCGAUGGAUGUCAAUGGA	Cleavage	1
trf_7_212	Bra000529	3	11.2	1	21	984	1004	UCCGUUGUCGUCCAGCGGUUA	UAACCGGAGGACGAUAAUGGA	Cleavage	1
trf_7_214	Bra013584	3	16.4	1	20	891	910	UCCGUUGUCGUCCAGCGGUU	AACCGAUGGAUGUCAAUGGA	Cleavage	1

trf_7_214	Bra000529	3	11.2	1	20	985	1004	UCCGUUGUCGUCCAGCGGUU	AACCGGAGGACGAUAAUGGA	Cleavage	1
trf_7_216	Bra013584	3	16.4	1	21	890	910	UCCGUUGUCGUCCAGCGGUUA	UAACCGAUGGAUGUCAAUGGA	Cleavage	1
trf_7_216	Bra000529	3	11.2	1	21	984	1004	UCCGUUGUCGUCCAGCGGUUA	UAACCGGAGGACGAUAAUGGA	Cleavage	1
trf_7_217	Bra013584	3	16.4	1	26	885	910	UCCGUUGUCGUCCAGCGGUUAGGAUA	UAUGAUAACCGAUGGAUGUCAAUGGA	Cleavage	1
trf_7_217	Bra000529	3	11.2	1	21	984	1004	UCCGUUGUCGUCCAGCGGUUA	UAACCGGAGGACGAUAAUGGA	Cleavage	1
trf_7_219	Bra016601	3	20.8	1	24	415	438	UCCGUCGUAGUCUAGCUGGUUAGG	CUGAAGAAGCUAGGCUGCGACGGA	Cleavage	1
trf_7_220	Bra016601	3	20.8	1	24	415	438	UCCGUCGUAGUCUAGCUGGUUAGG	CUGAAGAAGCUAGGCUGCGACGGA	Cleavage	1
trf_7_221	Bra024638	3	7.5	1	23	203	225	GGGGAUGUAGCUCAAAUGGUAGA	UCCAACAUUUCGGUUACAUCCCC	Cleavage	1
trf_7_221	Bra025904	3	17.2	1	20	344	363	GGGGAUGUAGCUCAAAUGGU	ACAAUUUGAGCUUCAUCUUC	Cleavage	1
trf_7_222	Bra016601	3	20.8	1	24	415	438	UCCGUCGUAGUCUAGCUGGUUAGG	CUGAAGAAGCUAGGCUGCGACGGA	Cleavage	1
trf_7_224	Bra016601	3	20.8	1	21	418	438	UCCGUCGUAGUCUAGCUGGUU	AAGAAGCUAGGCUGCGACGGA	Cleavage	1
trf_7_225	Bra016601	3	20.8	1	24	415	438	UCCGUCGUAGUCUAGCUGGUUAGG	CUGAAGAAGCUAGGCUGCGACGGA	Cleavage	1
trf_7_243	Bra024638	3	7.5	1	23	203	225	GGGGAUGUAGCUCAAAUGGUAGA	UCCAACAUUUCGGUUACAUCCCC	Cleavage	1
trf_7_243	Bra025904	3	17.2	1	20	344	363	GGGGAUGUAGCUCAAAUGGU	ACAAUUUGAGCUUCAUCUUC	Cleavage	1
trf_7_1	Bra033370	3	21.6	1	20	4750	4769	GGGGAUGUAGCUCAAAUGGU	ACCCUUUGGGUAACAUCCCC	Translation	1
trf_7_2	Bra033370	3	21.6	1	20	4750	4769	GGGGAUGUAGCUCAAAUGGU	ACCCUUUGGGUAACAUCCCC	Translation	1
trf_7_3	Bra037748	2.5	22.8	1	25	561	585	UCCGUUAUCGUCCAGCGGUUAGGAU	GUUGAAACCGCUGUCUGAUAACGGA	Translation	1

trf_7_4	Bra037748	2.5	22.8	1	20	566	585	UCCGUUAUCGUCCAGCGGUU	AACCGCUGUCUGAUAACGGA	Translation	1
trf_7_5	Bra037748	2.5	22.8	1	25	561	585	UCCGUUAUCGUCCAGCGGUUAGGAU	GUUGAAACCGCUGUCUGAUAACGGA	Translation	1
trf_7_6	Bra037748	2.5	22.8	1	20	566	585	UCCGUUAUCGUCCAGCGGUU	AACCGCUGUCUGAUAACGGA	Translation	1
trf_7_7	Bra037748	2.5	22.8	1	25	561	585	UCCGUUAUCGUCCAGCGGUUAGGAU	GUUGAAACCGCUGUCUGAUAACGGA	Translation	1
trf_7_8	Bra037748	2.5	22.8	1	20	566	585	UCCGUUAUCGUCCAGCGGUU	AACCGCUGUCUGAUAACGGA	Translation	1
trf_7_9	Bra037748	2.5	22.8	1	20	566	585	UCCGUUAUCGUCCAGCGGUU	AACCGCUGUCUGAUAACGGA	Translation	1
trf_7_12	Bra037499	3	14.7	1	22	174	195	UCCGUUGUAGUCUAGCUGGUUA	UGACUGGUUAGGCCACAACGGA	Translation	1
trf_7_13	Bra033370	3	21.6	1	20	4750	4769	GGGGAUGUAGCUCAAAUGGU	ACCCUUUGGGUAACAUCCCC	Translation	1
trf_7_18	Bra033370	3	21.6	1	20	4750	4769	GGGGAUGUAGCUCAAAUGGU	ACCCUUUGGGUAACAUCCCC	Translation	1
trf_7_25	Bra036328	3	13.8	1	20	708	727	GUGGCUGUAGUUUAGUGGUG	CAACACUAAAGUGCAGCUAC	Translation	1
trf_7_25	Bra003191	3	7.6	1	20	27	45	GUGGCUGUAGUUUAGUGGUG	UACCAUUAA-CUACAGCCAC	Translation	1
trf_7_37	Bra033370	3	21.6	1	20	4750	4769	GGGGAUGUAGCUCAAAUGGU	ACCCUUUGGGUAACAUCCCC	Translation	1
trf_7_48	Bra020153	2.5	15.4	1	22	1953	1974	UCCGUUGUCGUCCAGCGGUUAG	CCAACUGCUGGAAGACAAUGGG	Translation	1
trf_7_48	Bra012143	2.5	18.5	1	20	392	411	UCCGUUGUCGUCCAGCGGUU	AAUCGCUGAACCACAACGGA	Translation	1
trf_7_48	Bra037748	3	22.8	1	20	566	585	UCCGUUGUCGUCCAGCGGUU	AACCGCUGUCUGAUAACGGA	Translation	1
trf_7_49	Bra020153	2.5	15.4	1	22	1953	1974	UCCGUUGUCGUCCAGCGGUUAG	CCAACUGCUGGAAGACAAUGGG	Translation	1
trf_7_49	Bra012143	2.5	18.5	1	20	392	411	UCCGUUGUCGUCCAGCGGUU	AAUCGCUGAACCACAACGGA	Translation	1

trf_7_49	Bra037748	3	22.8	1	25	561	585	UCCGUUGUCGUCCAGCGGUUAGGAU	GUUGAAACCGCUGUCUGAUAACGGA	Translation	1
trf_7_50	Bra020153	2.5	15.4	1	22	1953	1974	UCCGUUGUCGUCCAGCGGUUAG	CCAACUGCUGGAAGACAAUGGG	Translation	1
trf_7_50	Bra012143	2.5	18.5	1	20	392	411	UCCGUUGUCGUCCAGCGGUU	AAUCGCUGAACCACAACGGA	Translation	1
trf_7_50	Bra037748	3	22.8	1	25	561	585	UCCGUUGUCGUCCAGCGGUUAGGAU	GUUGAAACCGCUGUCUGAUAACGGA	Translation	1
trf_7_51	Bra020153	2.5	15.4	1	22	1953	1974	UCCGUUGUCGUCCAGCGGUUAG	CCAACUGCUGGAAGACAAUGGG	Translation	1
trf_7_51	Bra012143	2.5	18.5	1	20	392	411	UCCGUUGUCGUCCAGCGGUU	AAUCGCUGAACCACAACGGA	Translation	1
trf_7_51	Bra037748	3	22.8	1	25	561	585	UCCGUUGUCGUCCAGCGGUUAGGAU	GUUGAAACCGCUGUCUGAUAACGGA	Translation	1
trf_7_52	Bra020153	3	15.4	1	22	1953	1974	NCCGUUGUCGUCCAGCGGUUAG	CCAACUGCUGGAAGACAAUGGG	Translation	1
trf_7_53	Bra020153	2.5	15.4	1	20	1955	1974	UCCGUUGUCGUCCAGCGGUU	AACUGCUGGAAGACAAUGGG	Translation	1
trf_7_53	Bra012143	2.5	18.5	1	20	392	411	UCCGUUGUCGUCCAGCGGUU	AAUCGCUGAACCACAACGGA	Translation	1
trf_7_53	Bra037748	3	22.8	1	20	566	585	UCCGUUGUCGUCCAGCGGUU	AACCGCUGUCUGAUAACGGA	Translation	1
trf_7_58	Bra036328	3	13.8	1	20	708	727	GUGGCUGUAGUUUAGUGGUG	CAACACUAAAGUGCAGCUAC	Translation	1
trf_7_58	Bra003191	3	7.6	1	20	27	45	GUGGCUGUAGUUUAGUGGUG	UACCAUUAA-CUACAGCCAC	Translation	1
trf_7_69	Bra033370	3	21.6	1	20	4750	4769	GGGGAUGUAGCUCAAAUGGU	ACCCUUUGGGUAACAUCCCC	Translation	1
trf_7_71	Bra033370	3	21.6	1	24	4746	4769	GGGGAUGUAGCUCAAAUGGUAGAG	CUGGACCCUUUGGGUAACAUCCCC	Translation	1
trf_7_72	Bra033370	3	21.6	1	20	4750	4769	GGGGAUGUAGCUCAAAUGGU	ACCCUUUGGGUAACAUCCCC	Translation	1
trf_7_75	Bra033370	3	21.6	1	20	4750	4769	GGGGAUGUAGCUCAAAUGGU	ACCCUUUGGGUAACAUCCCC	Translation	1

trf_7_82	Bra036328	3	13.8	1	20	708	727	GUGGCUGUAGUUUAGUGGUG	CAACACUAAAGUGCAGCUAC	Translation	1
trf_7_82	Bra003191	3	7.6	1	20	27	45	GUGGCUGUAGUUUAGUGGUG	UACCAUUAA-CUACAGCCAC	Translation	1
trf_7_107	Bra037748	2.5	22.8	1	25	561	585	UCCGUUAUCGUCCAGCGGUUAGGAU	GUUGAAACCGCUGUCUGAUAACGGA	Translation	1
trf_7_108	Bra037748	2.5	22.8	1	25	561	585	UCCGUUAUCGUCCAGCGGUUAGGAU	GUUGAAACCGCUGUCUGAUAACGGA	Translation	1
trf_7_109	Bra037748	2.5	22.8	1	20	566	585	UCCGUUAUCGUCCAGCGGUU	AACCGCUGUCUGAUAACGGA	Translation	1
trf_7_110	Bra037748	2.5	22.8	1	20	566	585	UCCGUUAUCGUCCAGCGGUU	AACCGCUGUCUGAUAACGGA	Translation	1
trf_7_111	Bra037748	2.5	22.8	1	25	561	585	UCCGUUAUCGUCCAGCGGUUAGGAU	GUUGAAACCGCUGUCUGAUAACGGA	Translation	1
trf_7_113	Bra037748	2.5	22.8	1	20	566	585	UCCGUUAUCGUCCAGCGGUU	AACCGCUGUCUGAUAACGGA	Translation	1
trf_7_115	Bra037748	2.5	22.8	1	20	566	585	UCCGUUAUCGUCCAGCGGUU	AACCGCUGUCUGAUAACGGA	Translation	1
trf_7_131	Bra020153	2.5	15.4	1	22	1953	1974	UCCGUUGUCGUCCAGCGGUUAG	CCAACUGCUGGAAGACAAUGGG	Translation	1
trf_7_131	Bra012143	2.5	18.5	1	20	392	411	UCCGUUGUCGUCCAGCGGUU	AAUCGCUGAACCACAACGGA	Translation	1
trf_7_131	Bra037748	3	22.8	1	20	566	585	UCCGUUGUCGUCCAGCGGUU	AACCGCUGUCUGAUAACGGA	Translation	1
trf_7_132	Bra020153	2.5	15.4	1	22	1953	1974	UCCGUUGUCGUCCAGCGGUUAG	CCAACUGCUGGAAGACAAUGGG	Translation	1
trf_7_132	Bra012143	2.5	18.5	1	20	392	411	UCCGUUGUCGUCCAGCGGUU	AAUCGCUGAACCACAACGGA	Translation	1
trf_7_132	Bra037748	3	22.8	1	25	561	585	UCCGUUGUCGUCCAGCGGUUAGGAU	GUUGAAACCGCUGUCUGAUAACGGA	Translation	1
trf_7_133	Bra020153	2.5	15.4	1	22	1953	1974	UCCGUUGUCGUCCAGCGGUUAG	CCAACUGCUGGAAGACAAUGGG	Translation	1
trf_7_133	Bra012143	2.5	18.5	1	20	392	411	UCCGUUGUCGUCCAGCGGUU	AAUCGCUGAACCACAACGGA	Translation	1

trf_7_1	Bra037		22					UCCGUUGUCGUCCAGC	GUUGAAACCGCUGUCU	Transl	
33	748	3	.8	1	25	561	585	GGUUAGGAU	GAUAACGGA	ation	1
trf_7_1	Bra020		15					UCCGUUGUCGUCCAGC	AACUGCUGGAAGACAA	Transl	
34	153	2.5	.4	1	20	1955	1974	GGUU	UGGG	ation	1
trf_7_1	Bra012		18					UCCGUUGUCGUCCAGC	AAUCGCUGAACCACAAC	Transl	
34	143	2.5	.5	1	20	392	411	GGUU	GGA	ation	1
trf_7_1	Bra037		22					UCCGUUGUCGUCCAGC	AACCGCUGUCUGAUAA	Transl	
34	748	3	.8	1	20	566	585	GGUU	CGGA	ation	1
trf_7_1	Bra020		15					UCCGUUGUCGUCCAGC	CCAACUGCUGGAAGAC	Transl	
35	153	2.5	.4	1	22	1953	1974	GGUUAG	AAUGGG	ation	1
trf_7_1	Bra012		18					UCCGUUGUCGUCCAGC	AAUCGCUGAACCACAAC	Transl	
35	143	2.5	.5	1	20	392	411	GGUU	GGA	ation	1
trf_7_1	Bra037		22					UCCGUUGUCGUCCAGC	GUUGAAACCGCUGUCU	Transl	
35	748	3	.8	1	25	561	585	GGUUAGGAU	GAUAACGGA	ation	1
trf_7_1	Bra020		15					UCCGUUGUCGUCCAGC	CCAACUGCUGGAAGAC	Transl	
37	153	2.5	.4	1	22	1953	1974	GGUUAG	AAUGGG	ation	1
trf_7_1	Bra012		18					UCCGUUGUCGUCCAGC	AAUCGCUGAACCACAAC	Transl	
37	143	2.5	.5	1	20	392	411	GGUU	GGA	ation	1
trf_7_1	Bra037		22					UCCGUUGUCGUCCAGC	AACCGCUGUCUGAUAA	Transl	
37	748	3	.8	1	20	566	585	GGUU	CGGA	ation	1
trf_7_1	Bra020		15					UCCGUUGUCGUCCAGC	CCAACUGCUGGAAGAC	Transl	
40	153	2.5	.4	1	22	1953	1974	GGUUAG	AAUGGG	ation	1
trf_7_1	Bra012		18					UCCGUUGUCGUCCAGC	AAUCGCUGAACCACAAC	Transl	
40	143	2.5	.5	1	20	392	411	GGUU	GGA	ation	1
trf_7_1	Bra037		22					UCCGUUGUCGUCCAGC	AACCGCUGUCUGAUAA	Transl	
40	748	3	.8	1	20	566	585	GGUU	CGGA	ation	1
trf_7_1	Bra020		15					UCCGUUGUCGUCCAGC	AACUGCUGGAAGACAA	Transl	
41	153	2.5	.4	1	20	1955	1974	GGUU	UGGG	ation	1
trf_7_1	Bra012		18					UCCGUUGUCGUCCAGC	AAUCGCUGAACCACAAC	Transl	
41	143	2.5	.5	1	20	392	411	GGUU	GGA	ation	1
trf_7_1	Bra037		22					UCCGUUGUCGUCCAGC	AACCGCUGUCUGAUAA	Transl	
41	748	3	.8	1	20	566	585	GGUU	CGGA	ation	1
trf_7_1	Bra020		15					UCCGUUGUCGUCCAGC	CCAACUGCUGGAAGAC	Transl	
42	153	2.5	.4	1	22	1953	1974	GGUUAG	AAUGGG	ation	1

trf_7_1	Bra012		18					UCCGUUGUCGUCCAGC	AAUCGCUGAACCACAAC	Transl	
42	143	2.5	.5	1	20	392	411	GGUU	GGA	ation	1
trf_7_1	Bra037		22					UCCGUUGUCGUCCAGC	GUUGAAACCGCUGUCU	Transl	
42	748	3	.8	1	25	561	585	GGUUAGGAU	GAUAACGGA	ation	1
trf_7_1	Bra020		15					UCCGUUGUCGUCCAGC	CCAACUGCUGGAAGAC	Transl	
43	153	2.5	.4	1	22	1953	1974	GGUUAG	AAUGGG	ation	1
trf_7_1	Bra012		18					UCCGUUGUCGUCCAGC	AAUCGCUGAACCACAAC	Transl	
43	143	2.5	.5	1	20	392	411	GGUU	GGA	ation	1
trf_7_1	Bra037		22					UCCGUUGUCGUCCAGC	GUUGAAACCGCUGUCU	Transl	
43	748	3	.8	1	25	561	585	GGUUAGGAU	GAUAACGGA	ation	1
trf_7_1	Bra020		15					UCCGUUGUCGUCCAGC	CCAACUGCUGGAAGAC	Transl	
44	153	2.5	.4	1	22	1953	1974	GGUUAG	AAUGGG	ation	1
trf_7_1	Bra012		18					UCCGUUGUCGUCCAGC	AAUCGCUGAACCACAAC	Transl	
44	143	2.5	.5	1	20	392	411	GGUU	GGA	ation	1
trf_7_1	Bra037		22					UCCGUUGUCGUCCAGC	AACCGCUGUCUGAUAA	Transl	
44	748	3	.8	1	20	566	585	GGUU	CGGA	ation	1
trf_7_1	Bra020		15					UCCGUUGUCGUCCAGC	CCAACUGCUGGAAGAC	Transl	
45	153	2.5	.4	1	22	1953	1974	GGUUAG	AAUGGG	ation	1
trf_7_1	Bra012		18					UCCGUUGUCGUCCAGC	AAUCGCUGAACCACAAC	Transl	
45	143	2.5	.5	1	20	392	411	GGUU	GGA	ation	1
trf_7_1	Bra037		22					UCCGUUGUCGUCCAGC	AACCGCUGUCUGAUAA	Transl	
45	748	3	.8	1	20	566	585	GGUU	CGGA	ation	1
trf_7_1	Bra036		13					GUGGCUGUAGUUUAG	CAACACUAAAGUGCAG	Transl	
51	328	3	.8	1	20	708	727	UGGUG	CUAC	ation	1
trf_7_1	Bra033		21					GGGGAUGUAGCUCAAA	ACCCUUUGGGUAACAU	Transl	
63	370	3	.6	1	20	4750	4769	UGGU	CCCC	ation	1
trf_7_1	Bra033		21					GGGGAUGUAGCUCAAA	ACCCUUUGGGUAACAU	Transl	
74	370	3	.6	1	20	4750	4769	UGGU	CCCC	ation	1
trf_7_1	Bra034		11					GGGAUUGUAGUUCAA	UCCAAAUGAGCAGCAA	Transl	
82	753	3	.6	1	20	443	462	UUGGA	UCCC	ation	1
trf_7_1	Bra003		13					GGGAUUGUAGUUCAA	UCCAGUUAAUCUACAA	Transl	
82	597	3	.1	1	20	1508	1527	UUGGA	UUCC	ation	1
trf_7_1	Bra023		12					GGGAUUGUAGUUCAA	UCCAAGUGAACAUCAA	Transl	
82	172	3	.4	1	20	614	633	UUGGA	UCCC	ation	1

trf_7_1	Bra033		10					GGGAUUGUAGUUCAA	ACCGAUUGAUCAGCAA	Transl	
85	536	3	.5	1	20	426	445	UCGGU	UUCC	ation	1
trf_7_1	Bra023		12					GGGAUUGUAGUUCAA	UUCCAAGUGAACAUCA	Transl	
87	172	3	.4	1	21	613	633	UUGGAA	AUCCC	ation	1
trf_7_1	Bra034		11					GGGAUUGUAGUUCAA	UCCAAAUGAGCAGCAA	Transl	
87	753	3	.6	1	20	443	462	UUGGA	UCCC	ation	1
trf_7_1	Bra003		13					GGGAUUGUAGUUCAA	UCCAGUUAAUCUACAA	Transl	
87	597	3	.1	1	20	1508	1527	UUGGA	UUCC	ation	1
trf_7_1	Bra033		10					GGGAUUGUAGUUCAA	ACCGAUUGAUCAGCAA	Transl	
88	536	3	.5	1	20	426	445	UCGGU	UUCC	ation	1
trf_7_1	Bra034		11					GGGAUUGUAGUUCAA	UCCAAAUGAGCAGCAA	Transl	
93	753	3	.6	1	20	443	462	UUGGA	UCCC	ation	1
trf_7_1	Bra003		13					GGGAUUGUAGUUCAA	UCCAGUUAAUCUACAA	Transl	
93	597	3	.1	1	20	1508	1527	UUGGA	UUCC	ation	1
trf_7_1	Bra023		12					GGGAUUGUAGUUCAA	UCCAAGUGAACAUCAA	Transl	
93	172	3	.4	1	20	614	633	UUGGA	UCCC	ation	1
trf_7_1	Bra034		11					GGGAUUGUAGUUCAA	AUCCAAAUGAGCAGCA	Transl	
95	753	3	.6	1	21	442	462	UUGGAU	AUCCC	ation	1
trf_7_1	Bra003		13					GGGAUUGUAGUUCAA	AUCCAGUUAAUCUACA	Transl	
95	597	3	.1	1	21	1507	1527	UUGGAU	AUUCC	ation	1
trf_7_1	Bra023		12					GGGAUUGUAGUUCAA	UCCAAGUGAACAUCAA	Transl	
95	172	3	.4	1	20	614	633	UUGGA	UCCC	ation	1
trf_7_2	Bra020		15					UCCGUUGUCGUCCAGC	CCAACUGCUGGAAGAC	Transl	
11	153	2.5	.4	1	22	1953	1974	GGUUAG	AAUGGG	ation	1
trf_7_2	Bra012		18					UCCGUUGUCGUCCAGC	AAUCGCUGAACCACAAC	Transl	
11	143	2.5	.5	1	20	392	411	GGUU	GGA	ation	1
trf_7_2	Bra037		22					UCCGUUGUCGUCCAGC	GUUGAAACCGCUGUCU	Transl	
11	748	3	.8	1	25	561	585	GGUUAGGAU	GAUAACGGA	ation	1
trf_7_2	Bra020		15					UCCGUUGUCGUCCAGC	CCAACUGCUGGAAGAC	Transl	
12	153	2.5	.4	1	22	1953	1974	GGUUAG	AAUGGG	ation	1
trf_7_2	Bra012		18					UCCGUUGUCGUCCAGC	AAUCGCUGAACCACAAC	Transl	
12	143	2.5	.5	1	20	392	411	GGUU	GGA	ation	1
trf_7_2	Bra037		22					UCCGUUGUCGUCCAGC	GUUGAAACCGCUGUCU	Transl	
12	748	3	.8	1	25	561	585	GGUUAGGAU	GAUAACGGA	ation	1

trf_7_2	Bra020		15					UCCGUUGUCGUCCAGC	AACUGCUGGAAGACAA	Transl	
14	153	2.5	.4	1	20	1955	1974	GGUU	UGGG	ation	1
trf_7_2	Bra012		18					UCCGUUGUCGUCCAGC	AAUCGCUGAACCACAAC	Transl	
14	143	2.5	.5	1	20	392	411	GGUU	GGA	ation	1
trf_7_2	Bra037		22					UCCGUUGUCGUCCAGC	AACCGCUGUCUGAUAA	Transl	
14	748	3	.8	1	20	566	585	GGUU	CGGA	ation	1
trf_7_2	Bra020		15					NCCGUUGUCGUCCAGC	CCAACUGCUGGAAGAC	Transl	
15	153	3	.4	1	22	1953	1974	GGUUAG	AAUGGG	ation	1
trf_7_2	Bra020		15					UCCGUUGUCGUCCAGC	CCAACUGCUGGAAGAC	Transl	
16	153	2.5	.4	1	22	1953	1974	GGUUAG	AAUGGG	ation	1
trf_7_2	Bra012		18					UCCGUUGUCGUCCAGC	AAUCGCUGAACCACAAC	Transl	
16	143	2.5	.5	1	20	392	411	GGUU	GGA	ation	1
trf_7_2	Bra037		22					UCCGUUGUCGUCCAGC	AACCGCUGUCUGAUAA	Transl	
16	748	3	.8	1	20	566	585	GGUU	CGGA	ation	1
trf_7_2	Bra020		15					UCCGUUGUCGUCCAGC	CCAACUGCUGGAAGAC	Transl	
17	153	2.5	.4	1	22	1953	1974	GGUUAG	AAUGGG	ation	1
trf_7_2	Bra012		18					UCCGUUGUCGUCCAGC	AAUCGCUGAACCACAAC	Transl	
17	143	2.5	.5	1	20	392	411	GGUU	GGA	ation	1
trf_7_2	Bra037		22					UCCGUUGUCGUCCAGC	GUUGAAACCGCUGUCU	Transl	
17	748	3	.8	1	25	561	585	GGUUAGGAU	GAUAACGGA	ation	1
trf_7_2	Bra033		21					GGGGAUGUAGCUCAAA	ACCCUUUGGGUAACAU	Transl	
21	370	3	.6	1	20	4750	4769	UGGU	CCCC	ation	1
trf_7_2	Bra033		21					GGGGAUGUAGCUCAAA	ACCCUUUGGGUAACAU	Transl	
43	370	3	.6	1	20	4750	4769	UGGU	CCCC	ation	1

rRF Leaves

ncRNA_Acc.	Target_Acc.	Expectation	UPE	ncRNA_start	ncRNA_end	Target_start	Target_end	ncRNA_aligned_fragment	Target_aligned_fragment	Inhibition	Multiplicity
rrf_1_1	Bra030994	2.5	18.8	1	24	1861	1884	CGAUCCACUGAGAUUCAGCCCUUU	AAAAGGCUGAAUCUUACUGGAUCU	Cleavage	1
rrf_1_1	Bra034079	3	19.1	1	20	2024	2043	CGAUCCACUGAGAUUCAGCC	GGCUUGAUCUUAGUGGAUCC	Cleavage	1
rrf_1_2	Bra030994	2.5	18.8	1	24	1861	1884	CGAUCCACUGAGAUUCAGCCCUUU	AAAAGGCUGAAUCUUACUGGAUCU	Cleavage	1
rrf_1_2	Bra034079	3	19.1	1	20	2024	2043	CGAUCCACUGAGAUUCAGCC	GGCUUGAUCUUAGUGGAUCC	Cleavage	1
rrf_1_4	Bra030994	2.5	18.8	1	24	1861	1884	CGAUCCACUGAGAUUCAGCCCUUU	AAAAGGCUGAAUCUUACUGGAUCU	Cleavage	1
rrf_1_4	Bra034079	3	19.1	1	20	2024	2043	CGAUCCACUGAGAUUCAGCC	GGCUUGAUCUUAGUGGAUCC	Cleavage	1
rrf_1_5	Bra030994	2.5	18.8	1	24	1861	1884	CGAUCCACUGAGAUUCAGCCCUUU	AAAAGGCUGAAUCUUACUGGAUCU	Cleavage	1
rrf_1_5	Bra034079	3	19.1	1	20	2024	2043	CGAUCCACUGAGAUUCAGCC	GGCUUGAUCUUAGUGGAUCC	Cleavage	1
rrf_1_7	Bra030994	2.5	18.8	1	24	1861	1884	CGAUCCACUGAGAUUCAGCCCUUU	AAAAGGCUGAAUCUUACUGGAUCU	Cleavage	1
rrf_1_7	Bra034079	3	19.1	1	20	2024	2043	CGAUCCACUGAGAUUCAGCC	GGCUUGAUCUUAGUGGAUCC	Cleavage	1
rrf_1_1	Bra013685	3	23.3	1	22	10	32	CGAUCCAC-UGAGAUUCAGCCCU	GGAGCUGAAUCUGAUGUGGAUCG	Translation	1
rrf_1_2	Bra013685	3	23.3	1	22	10	32	CGAUCCAC-UGAGAUUCAGCCCU	GGAGCUGAAUCUGAUGUGGAUCG	Translation	1
rrf_1_4	Bra013685	3	23.3	1	22	10	32	CGAUCCAC-UGAGAUUCAGCCCU	GGAGCUGAAUCUGAUGUGGAUCG	Translation	1
rrf_1_5	Bra013685	3	23.3	1	22	10	32	CGAUCCAC-UGAGAUUCAGCCCU	GGAGCUGAAUCUGAUGUGGAUCG	Translation	1
rrf_1_7	Bra013685	3	23.3	1	22	10	32	CGAUCCAC-UGAGAUUCAGCCCU	GGAGCUGAAUCUGAUGUGGAUCG	Translation	1

rRF Apic meristem

ncRNA_Acc.	Target_Acc.	Expectation	UPE	ncRNA_start	ncRNA_end	Target_start	Target_end	ncRNA_aligned_fragment	Target_aligned_fragment	Inhibition	Multiplicity
rrf_2_5	Bra030994	2.5	18.8	1	24	1861	1884	CGAUCCACUGAGAUUCAGCCCUUU	AAAAGGCUGAAUCUUACUGGAUCU	Cleavage	1
rrf_2_5	Bra034079	3	19.1	1	20	2024	2043	CGAUCCACUGAGAUUCAGCC	GGCUUGAUCUUAGUGGAUCC	Cleavage	1
rrf_2_10	Bra030994	2.5	18.8	1	24	1861	1884	CGAUCCACUGAGAUUCAGCCCUUU	AAAAGGCUGAAUCUUACUGGAUCU	Cleavage	1
rrf_2_10	Bra034079	3	19.1	1	20	2024	2043	CGAUCCACUGAGAUUCAGCC	GGCUUGAUCUUAGUGGAUCC	Cleavage	1
rrf_2_11	Bra001816	2	19.2	1	24	220	243	AGUUGCAUUUUCCUUGACACUUUU	AAAAGCGUCAAGGAGGAUGCAACU	Cleavage	1
rrf_2_11	Bra028470	3	10.8	1	24	205	228	AGUUGCAUUUUCCUUGACACUUUU	AAGAGUGUCAUGGAAAAUUCAGCU	Cleavage	1
rrf_2_11	Bra028748	3	18.0	1	24	446	468	AGUUGCAUUUUCCUUGACACUUUU	AGGAG-GUCAAGGAGAAUGCGACU	Cleavage	1
rrf_2_11	Bra021522	3	20.7	1	24	1018	1041	AGUUGCAUUUUCCUUGACACUUUU	AAAUGCGGCAAUGAAAAUGCAACU	Cleavage	1
rrf_2_11	Bra033125	3	18.6	1	21	16	36	AGUUGCAUUUUCCUUGACACU	ACUGCCAAGGAAAAUGUAGCU	Cleavage	1
rrf_2_11	Bra013166	3	13.5	1	20	293	312	AGUUGCAUUUUCCUUGACAC	AUGUCAAAGAGAAUGCAAUU	Cleavage	1
rrf_2_11	Bra032998	3	12.8	1	24	319	343	AGUUG-CAUUUUCCUUGACACUUUU	GAACCUGUCAAGGAAAAUGACGACU	Cleavage	1
rrf_2_11	Bra030472	3	19.2	1	24	1803	1826	AGUUGCAUUUUCCUUGACACUUUU	GAGCAUGUCAAAGAAACUGCAACU	Cleavage	1
rrf_2_12	Bra030994	2.5	18.8	1	24	1861	1884	CGAUCCACUGAGAUUCAGCCCUUU	AAAAGGCUGAAUCUUACUGGAUCU	Cleavage	1
rrf_2_12	Bra034079	3	19.1	1	20	2024	2043	CGAUCCACUGAGAUUCAGCC	GGCUUGAUCUUAGUGGAUCC	Cleavage	1

rrf_2_15	Bra030994	2.5	18.8	1	24	1861	1884	CGAUCCACUGAGAUUCAGCCCUUU	AAAAGGCUGAAUCUUACUGGAUCU	Cleavage	1
rrf_2_15	Bra034079	3	19.1	1	20	2024	2043	CGAUCCACUGAGAUUCAGCC	GGCUUGAUCUUAGUGGAUCC	Cleavage	1
rrf_2_5	Bra013685	3	23.3	1	22	10	32	CGAUCCAC-UGAGAUUCAGCCCU	GGAGCUGAAUCUGAUGUGGAUCG	Translation	1
rrf_2_10	Bra013685	3	23.3	1	22	10	32	CGAUCCAC-UGAGAUUCAGCCCU	GGAGCUGAAUCUGAUGUGGAUCG	Translation	1
rrf_2_12	Bra013685	3	23.3	1	22	10	32	CGAUCCAC-UGAGAUUCAGCCCU	GGAGCUGAAUCUGAUGUGGAUCG	Translation	1
rrf_2_15	Bra013685	3	23.3	1	22	10	32	CGAUCCAC-UGAGAUUCAGCCCU	GGAGCUGAAUCUGAUGUGGAUCG	Translation	1

rRF Unpollinated Ovules

ncRNA_Acc.	Target_Acc.	Expectation	UPE	ncRNA_start	ncRNA_end	Target_start	Target_end	ncRNA_aligned_fragment	Target_aligned_fragment	Inhibition	Multiplicity
rrf_4_3	Bra013685	3	23.3	1	22	10	32	CGAUCCAC-UGAGAUUCAGCCCU	GGAGCUGAAUCUGAUGUGGAUCG	Translation	1
rrf_4_3	Bra030994	2.5	18.8	1	24	1861	1884	CGAUCCACUGAGAUUCAGCCCUUU	AAAAGGCUGAAUCUUACUGGAUCU	Cleavage	1
rrf_4_3	Bra034079	3	19.1	1	20	2024	2043	CGAUCCACUGAGAUUCAGCC	GGCUUGAUCUUAGUGGAUCC	Cleavage	1

rRF Pollinated Ovules

ncRNA_Acc.	Target_Acc.	Expectation	UPE	ncRNA_start	ncRNA_end	Target_start	Target_end	ncRNA_aligned_fragment	Target_aligned_fragment	Inhibition	Multiplicity
rrf_5_4	Bra013685	3	23.3	1	22	10	32	CGAUCCAC-UGAGAUUCAGCCCU	GGAGCUGAAUCUGAUGUGGAUCG	Translation	1
rrf_5_4	Bra030994	2.5	18.8	1	24	1861	1884	CGAUCCACUGAGAUUCAGCCCUUU	AAAAGGCUGAAUCUUACUGGAUCU	Cleavage	1
rrf_5_4	Bra034	3	19	1	20	2024	2043	CGAUCCACUGAGAUU	GGCUUGAUCUUAGUG	Cleava	1

	079		.1					CAGCC	GAUCC	ge	
rrf_5_1 2	Bra021 135	3	14 .3	1	21	67	87	UAGGUGAACCUGCGG AAGGAU	AUCCUUCCGCGGCUU CACUUU	Transla tion	1
rrf_5_1 2	Bra026 243	3	12 .3	1	20	1656	1675	UAGGUGAACCUGCGG AAGGA	UCCUUUCCCACGUUC ACCUG	Transla tion	1

rRF Embryo

ncRNA _Acc.	Target _Acc.	Expect ation	U PE	ncRNA_ start	ncRNA _end	Target_ start	Target _end	ncRNA_aligned_fragm ent	Target_aligned_fragm ent	Inhibiti on	Multipl icity
rrf_6_1	Bra013 685	3	23 .3	1	22	10	32	CGAUCCAC- UGAGAUUCAGCCCU	GGAGCUGAAUCUGAU GUGGAUCG	Transla tion	1
rrf_6_1	Bra030 994	2.5	18 .8	1	24	1861	1884	CGAUCCACUGAGAUU CAGCCCUUU	AAAAGGCUGAAUCUU ACUGGAUCU	Cleava ge	1
rrf_6_1	Bra034 079	3	19 .1	1	20	2024	2043	CGAUCCACUGAGAUU CAGCC	GGCUUGAUCUUAGUG GAUCC	Cleava ge	1

rRF Endosperm

ncRNA _Acc.	Target _Acc.	Expect ation	U PE	ncRNA_ start	ncRNA _end	Target_ start	Target _end	ncRNA_aligned_fragm ent	Target_aligned_fragm ent	Inhibiti on	Multipl icity
rrf_7_1	Bra013 685	3	23 .3	1	22	10	32	CGAUCCAC- UGAGAUUCAGCCCU	GGAGCUGAAUCUGAU GUGGAUCG	Transla tion	1
rrf_7_1	Bra030 994	2.5	18 .8	1	24	1861	1884	CGAUCCACUGAGAUU CAGCCCUUU	AAAAGGCUGAAUCUU ACUGGAUCU	Cleava ge	1
rrf_7_1	Bra034 079	3	19 .1	1	20	2024	2043	CGAUCCACUGAGAUU CAGCC	GGCUUGAUCUUAGUG GAUCC	Cleava ge	1

snRF Leaves

ncRNA_	Target_	Expecta	UP	ncRNA_	ncRNA_	Target_	Target_	ncRNA_aligned_frag	Target_aligned_frag	Inhibiti	Multipl

Acc.	Acc.	tion	E	start	end	start	end	ment	ment	on	icity
snrf_1_2	Bra015318	3	18.7	1	20	12	31	GGAAGGGCUCCCUUCGGGGU	UUCCCGGAGGGAGUCCUUCU	Cleavage	1
snrf_1_2	Bra017901	3	22.0	1	20	74	93	GGAAGGGCUCCCUUCGGGGU	AUCCCGAGGAGAGCUCUUCU	Translation	1
snrf_1_3	Bra027237	3	22.6	1	20	829	848	GGAAGGGCUCCUUUCGGGGU	AACCUGAAACGAGCUCUUCC	Translation	1
snrf_1_3	Bra039942	2.5	20.0	1	21	923	943	GGAAGGGCUCCUUUCGGGGUA	UAUUCCGCAAGGAGCCCUUCU	Cleavage	1

snRF Apical meristem

ncRNA_Acc.	Target_Acc.	Expectation	UPE	ncRNA_start	ncRNA_end	Target_start	Target_end	ncRNA_aligned_fragment	Target_aligned_fragment	Inhibition	Multiplicity
snrf_2_2	Bra018742	3	13.6	1	20	1705	1724	AUUGCACGGACUGGCUCAAC	UUUGAGCCAGACGGUGCAAU	Translation	1
snrf_2_9	Bra011122	3	17.1	1	21	303	323	ACUGCACGGGCCGGCUCAACC	GGCUGAGCUGGUCAGUGCAGU	Cleavage	1
snrf_2_36	Bra007036	3	24.1	1	20	730	749	UUGCACGGGCUUGGCUCAAC	UUUGAGCCAAGCCCGUUCAG	Cleavage	1
snrf_2_7	Bra003006	3	12.8	1	20	396	415	ACUGCACGGGCUGGCUCAAC	UUUGAGACAGCUUGUGCAGU	Cleavage	1
snrf_2_27	Bra010978	3	16.3	1	20	466	485	ACGGCAUGGACCUGGCUUAA	UUAAGACGGGUCCAUGCUGC	Cleavage	1
snrf_2_2	Bra011353	3	15.4	1	20	493	512	AUUGCACGGACUGGCUCAAC	GCUGAUCCAGUUUGUGCAAU	Cleavage	1
snrf_2_17	Bra019990	3	24.0	1	22	1797	1818	UAUUGCAUGGCCUGGCUCAACC	GUUUGAGCCAGGUCAUGCUAUC	Cleavage	1
snrf_2_20	Bra027119	3	21.1	1	22	562	583	CUACUGCAUUGGCUGGCUCAAC	GCUGGGCCAGGCGAUGCGGUGG	Cleavage	1
snrf_2_1	Bra032383	3	23.9	1	21	1509	1529	GCACGGACUGGCUCAACCCGC	GUGGGUGAAGCCGGUCCGUGC	Cleavage	1
snrf_2_	Bra000	3	18	1	22	1425	1446	AUUGCACGGGCUUG	GCUUGAGCCUAGUCC	Cleava	1

38	399		.0					GCUCAACC	GAGCAAU	ge	
snrf_2_	Bra001		16					UAUUGCACGGAUCU	UAGAGUUGGAUCUGU	Cleava	
31	161	3	.4	1	21	2087	2107	GGCUCAA	GCAAUA	ge	1
snrf_2_	Bra002		17					UUGCACGGAUCUGGC	GUUGAGCCAGAUGCG	Cleava	
30	908	3	.9	1	20	286	305	UCAAC	UGUGU	ge	1
snrf_2_	Bra005		22					CUGCACGGGCUGGCU	GGAGGAGCUAGCCCG	Cleava	
24	157	2.5	.6	1	20	409	428	CAUCC	UGCAU	ge	1
snrf_2_	Bra006		18					CUGCAUGGACCUGGC	GUACGUUGAGCCAGG	Cleava	
26	208	2.5	.9	1	24	2898	2921	UCAACCCAC	UCCAGGUGG	ge	1
snrf_2_	Bra008		17					UAUUGCACGGAUCU	GCUGAUCCGGAUCCG	Cleava	
31	483	3	.3	1	22	100	121	GGCUCAAC	UGUGAUG	ge	1
snrf_2_	Bra011		22					UUGCACGGAUCUGGC	GGUGAGCUGGAUCUG	Cleava	
30	732	3	.6	1	20	780	799	UCAAC	UGCAG	ge	1
snrf_2_	Bra012		16					CUACUGCAUUGGCUG	GCUGAGUCAGCUGAU	Cleava	
20	866	3	.5	1	22	3075	3096	GCUCAAC	GCAGAAG	ge	1
snrf_2_	Bra015		20					CUGCACGGGCUGGCU	GGUUGGGCCAGCACU	Cleava	
14	754	3	.5	1	20	2082	2101	CAACC	UGCAG	ge	1
snrf_2_	Bra017		19					UGCACGGGCUUGGC	UGUGUUGCAGCCAAG	Cleava	
37	342	3	.4	1	22	1440	1462	U-CAACCCA	CUCGUGUA	ge	1
snrf_2_	Bra027		16					CGGCAUGGACCUGGC	GGAUGAGACAGGUUC	Cleava	
28	318	3	.2	1	21	587	607	UUAACC	AUGCCG	ge	1
snrf_2_	Bra030		18					UGCACGGACUGGC-	GGUUGAAGCUAGUCU	Cleava	
10	327	3	.5	1	19	6629	6648	UCAACC	GUGCA	ge	1
snrf_2_	Bra031		18					UAUUGCAUGGCCUG	GCUUGAGACAGGCUU	Cleava	
17	747	2.5	.9	1	22	1020	1041	GCUCAACC	UGCAAUA	ge	1
snrf_2_	Bra032		15					UUGCACGGAUCUGGC	GUUGAGCCAGAAGUG	Transla	
30	048	2.5	.7	1	20	207	226	UCAAC	UGCAA	tion	1
snrf_2_	Bra034		17					GCACGGACUGGCUCA	CGAGUUGAGCCAGUC	Cleava	
1	244	3	.4	1	20	702	721	ACCCG	UGAGC	ge	1
snrf_2_	Bra036		16					CUACUGCAUUGGCUG	GCUGAGUCAGCUGAU	Cleava	
20	834	3	.6	1	22	2724	2745	GCUCAAC	GCAGAAG	ge	1
snrf_2_	Bra041		20					UUGCAUGGCCUGGC	GUUGUUUGAGCUAGG	Cleava	
18	092	3	.1	1	23	990	1012	UCAACCCAC	UUAUGCAG	ge	1

snRF Pollen

ncRNA_Acc.	Target_Acc.	Expectation	UPE	ncRNA_start	ncRNA_end	Target_start	Target_end	ncRNA_aligned_fragment	Target_aligned_fragment	Inhibition	Multiplicity
snrf_3_11	Bra017342	3	19.4	1	22	1440	1462	UGCACGGGCUUGGCU-CAACCCA	UGUGUUGCAGCCAAGCUCGUGUA	Cleavage	1
snrf_3_5	Bra011353	3	15.4	1	20	493	512	AUUGCACGGACUGGCUCAAC	GCUGAUCCAGUUUGUGCAAU	Cleavage	1
snrf_3_1	Bra032383	3	23.9	1	21	1509	1529	GCACGGACUGGCUCAACCCGC	GUGGGUGAAGCCGGUCCGUGC	Cleavage	1
snrf_3_4	Bra003006	3	12.8	1	20	396	415	ACUGCACGGGCUGGCUCAAC	UUUGAGACAGCUUGUGCAGU	Cleavage	1
snrf_3_3	Bra011122	3	17.1	1	21	303	323	ACUGCACGGGCCGGCUCAACC	GGCUGAGCUGGUCAGUGCAGU	Cleavage	1
snrf_3_2	Bra030327	3	18.5	1	19	6629	6648	UGCACGGACUGGC-UCAACC	GGUUGAAGCUAGUCUGUGCA	Cleavage	1
snrf_3_1	Bra034244	3	17.4	1	20	702	721	GCACGGACUGGCUCAACCCG	CGAGUUGAGCCAGUCUGAGC	Cleavage	1

snRF Unpollinated Ovules

ncRNA_Acc.	Target_Acc.	Expectation	UPE	ncRNA_start	ncRNA_end	Target_start	Target_end	ncRNA_aligned_fragment	Target_aligned_fragment	Inhibition	Multiplicity
snrf_4_10	Bra015318	3	18.7	1	20	12	31	GGAAGGGCUCCCUUCGGGGU	UUCCCGGAGGGAGUCCUUCU	Cleavage	1
snrf_4_10	Bra017901	3	22.0	1	20	74	93	GGAAGGGCUCCCUUCGGGGU	AUCCCGAGGAGAGCUCUUCU	Translation	1
snrf_4_3	Bra018742	3	14.0	1	21	1706	1726	CUAUUGCACGGACUGGCUCAA	UUGAGCCAGACGGUGCAAUAU	Translation	1
snrf_4_1	Bra032383	3	23.9	1	21	1509	1529	GCACGGACUGGCUCAACCCGC	GUGGGUGAAGCCGGUCCGUGC	Cleavage	1
snrf_4_	Bra007	3	24	1	20	730	749	UUGCACGGGCUUGG	UUUGAGCCAAGCCCG	Cleava	1

18	036		.1					CUCAAC	UUCAG	ge	
snrf_4_5	Bra011122	3	17.5	1	23	303	325	CUACUGCACGGCCGGCUCAACC	GGCUGAGCUGGUCAGUGCAGUAC	Translation	1
snrf_4_9	Bra027119	3	21.1	1	22	562	583	CUAUUGCAUGGCCUGGCUCAAC	GCUGGGCCAGGCGAUGCGGUGG	Translation	1
snrf_4_17	Bra027237	3	22.6	1	20	829	848	GGAAGGGCUCCUUUCGGGGU	AACCUGAAACGAGCUCUUCC	Translation	1
snrf_4_2	Bra030327	3	18.5	1	19	6629	6648	UGCACGGACUGGC-UCAACC	GGUUGAAGCUAGUCUGUGCA	Cleavage	1
snrf_4_1	Bra034244	3	17.4	1	20	702	721	GCACGGACUGGCUCAACCCG	CGAGUUGAGCCAGUCUGAGC	Cleavage	1
snrf_4_17	Bra039942	2.5	20.0	1	21	923	943	GGAAGGGCUCCUUUCGGGGUA	UAUUCCGCAAGGAGCCCUUCU	Cleavage	1

snRF Pollinated Ovules

ncRNA_Acc.	Target_Acc.	Expectation	UPE	ncRNA_start	ncRNA_end	Target_start	Target_end	ncRNA_aligned_fragment	Target_aligned_fragment	Inhibition	Multiplicity
snrf_5_5	Bra018742	3	14.016	1	21	1706	1726	CUAUUGCACGGACUGGCUCAA	UUGAGCCAGACGGUGCAAUAU	Translation	1
snrf_5_1	Bra032383	3	23.896	1	22	1509	1530	UGCACGGACUGGCUCAACCCGC	GUGGGUGAAGCCGGUCCGUGCG	Cleavage	1
snrf_5_9	Bra011353	3	15.438	1	20	493	512	AUUGCACGGACUGGCUCAAC	GCUGAUCCAGUUUGUGCAAU	Cleavage	1
snrf_5_15	Bra006208	2.5	18.876	1	24	2898	2921	CUGCAUGGACCUGGCUCAACCCAC	GUACGUUGAGCCAGGUCCAGGUGG	Cleavage	1
snrf_5_20	Bra007036	3	24.114	1	20	730	749	UUGCACGGGCUUGGCUCAAC	UUUGAGCCAAGCCCGUUCAG	Cleavage	1
snrf_5_14	Bra010978	3	16.331	1	20	466	485	ACGGCAUGGACCUGGCUUAA	UUAAGACGGGUCCAUGCUGC	Cleavage	1
snrf_5_2	Bra011122	3	17.457	1	23	303	325	CUACUGCACGGGCCGGCUCAACC	GGCUGAGCUGGUCAGUGCAGUAC	Translation	1

snrf_5_1	Bra030327	3	18.523	1	19	6629	6648	UGCACGGACUGGC-UCAACC	GGUUGAAGCUAGUCUGUGCA	Cleavage	1
snrf_5_7	Bra034244	3	17.379	1	20	702	721	GCACGGACUGGCUCAACCCG	CGAGUUGAGCCAGUCUGAGC	Cleavage	1

snRF Embryo

ncRNA_Acc.	Target_Acc.	Expectation	UPE	ncRNA_start	ncRNA_end	Target_start	Target_end	ncRNA_aligned_fragment	Target_aligned_fragment	Inhibition	Multiplicity
snrf_6_1	Bra018742	3	14.016	1	21	1706	1726	CUAUUGCACGGACUGGCUCAA	UUGAGCCAGACGGUGCAAUAU	Translation	1
snrf_6_10	Bra015318	3	18.697	1	20	12	31	GGAAGGGCUCCCUUCGGGGU	UUCCCGGAGGGAGUCCUUCU	Cleavage	1
snrf_6_10	Bra017901	3	22.003	1	20	74	93	GGAAGGGCUCCCUUCGGGGU	AUCCCGAGGAGAGCUCUUCU	Translation	1
snrf_6_9	Bra027237	3	22.615	1	20	829	848	GGAAGGGCUCCUUUCGGGGU	AACCUGAAACGAGCUCUUCC	Translation	1
snrf_6_9	Bra039942	2.5	20.004	1	21	923	943	GGAAGGGCUCCUUUCGGGGUA	UAUUCCGCAAGGAGCCCUUCU	Cleavage	1
snrf_6_6	Bra011353	3	15.438	1	20	493	512	AUUGCACGGACUGGCUCAAC	GCUGAUCCAGUUUGUGCAAU	Cleavage	1
snrf_6_4	Bra032383	3	23.918	1	21	1509	1529	GCACGGACUGGCUCAACCCGC	GUGGGUGAAGCCGGUCCGUGC	Cleavage	1
snrf_6_23	Bra001161	3	16.408	1	21	2087	2107	UAUUGCACGGAUCUGGCUCAA	UAGAGUUGGAUCUGUGCAAUA	Cleavage	1
snrf_6_23	Bra008483	3	17.259	1	22	100	121	UAUUGCACGGAUCUGGCUCAAC	GCUGAUCCGGAUCCGUGUGAUG	Cleavage	1
snrf_6_21	Bra017746	2.5	21.525	1	22	1213	1234	UUGCAAGGGCCCGGCUCAUCCC	GAGAUGAGGCAGGCCCUUGCGA	Cleavage	1
snrf_6_7	Bra019990	3	23.993	1	22	1797	1818	UAUUGCAUGGCCUGGCUCAACC	GUUUGAGCCAGGUCAUGCUAUC	Cleavage	1
snrf_6_	Bra022	3	19.2	1	21	405	425	UUGCAAGGGCCCGG	GGAUGAACCGGGCAC	Cleava	1

21	087		37					CUCAUCC	UUGUGA	ge	
snrf_6_21	Bra022088	3	21.225	1	21	387	407	UUGCAAGGGCCCGGCUCAUCC	GGAUGAACCGGGCACUUGUGA	Cleavage	1
snrf_6_21	Bra024487	2.5	23.637	1	23	1332	1354	UUGCAAGGGCCCGGCUCAUCCCG	CGAGAUGAGGCAGGCUCUUGCAA	Cleavage	1
snrf_6_8	Bra027119	3	21.072	1	22	562	583	CUAUUGCAUGGCCUGGCUCAAC	GCUGGGCCAGGCGAUGCGGUGG	Translation	1
snrf_6_7	Bra031747	2.5	18.92	1	22	1020	1041	UAUUGCAUGGCCUGGCUCAACC	GCUUGAGACAGGCUUUGCAAUA	Cleavage	1
snrf_6_4	Bra034244	3	17.379	1	20	702	721	GCACGGACUGGCUCAACCCG	CGAGUUGAGCCAGUCUGAGC	Cleavage	1

snRF Endosperm

ncRNA_Acc.	Target_Acc.	Expectation	UPE	ncRNA_start	ncRNA_end	Target_start	Target_end	ncRNA_aligned_fragment	Target_aligned_fragment	Inhibition	Multiplicity
snrf_7_2	Bra018742	3	13.6	1	20	1705	1724	AUUGCACGGACUGGCUCAAC	UUUGAGCCAGACGGUGCAAU	Translation	1
snrf_7_2	Bra011353	3	15.4	1	20	493	512	AUUGCACGGACUGGCUCAAC	GCUGAUCCAGUUUGUGCAAU	Cleavage	1
snrf_7_1	Bra032383	3	23.9	1	22	1509	1530	UGCACGGACUGGCUCAACCCGC	GUGGGUGAAGCCGGUCCGUGCG	Cleavage	1
snrf_7_14	Bra001161	3	16.4	1	21	2087	2107	UAUUGCACGGAUCUGGCUCAA	UAGAGUUGGAUCUGUGCAAUA	Cleavage	1
snrf_7_15	Bra002908	3	17.9	1	20	286	305	UUGCACGGAUCUGGCUCAAC	GUUGAGCCAGAUGCGUGUGU	Cleavage	1
snrf_7_12	Bra005157	2.5	22.6	1	20	409	428	CUGCACGGGCUGGCUCAUCC	GGAGGAGCUAGCCCGUGCAU	Cleavage	1
snrf_7_20	Bra007036	3	24.1	1	20	730	749	UUGCACGGGCUUGGCUCAAC	UUUGAGCCAAGCCCGUUCAG	Cleavage	1
snrf_7_14	Bra008483	3	17.3	1	22	100	121	UAUUGCACGGAUCUGGCUCAAC	GCUGAUCCGGAUCCGUGUGAUG	Cleavage	1

snrf_7_7	Bra011122	3	17.5	1	23	303	325	CUACUGCACGGGCCGGCUCAACC	GGCUGAGCUGGUCAGUGCAGUAC	Translation	1
snrf_7_15	Bra011732	3	22.6	1	20	780	799	UUGCACGGAUCUGGCUCAAC	GGUGAGCUGGAUCUGUGCAG	Cleavage	1
snrf_7_13	Bra017746	2.5	21.5	1	22	1213	1234	UUGCAAGGGCCCGGCUCAUCCC	GAGAUGAGGCAGGCCCUUGCGA	Cleavage	1
snrf_7_13	Bra022087	3	19.2	1	21	405	425	UUGCAAGGGCCCGGCUCAUCC	GGAUGAACCGGGCACUUGUGA	Cleavage	1
snrf_7_13	Bra022088	3	21.2	1	21	387	407	UUGCAAGGGCCCGGCUCAUCC	GGAUGAACCGGGCACUUGUGA	Cleavage	1
snrf_7_13	Bra024487	2.5	23.6	1	23	1332	1354	UUGCAAGGGCCCGGCUCAUCCCG	CGAGAUGAGGCAGGCUCUUGCAA	Cleavage	1
snrf_7_1	Bra030327	3	18.5	1	19	6629	6648	UGCACGGACUGGC-UCAACC	GGUUGAAGCUAGUCUGUGCA	Cleavage	1
snrf_7_15	Bra032048	2.5	15.7	1	20	207	226	UUGCACGGAUCUGGCUCAAC	GUUGAGCCAGAAGUGUGCAA	Translation	1
snrf_7_6	Bra034244	3	17.4	1	20	702	721	GCACGGACUGGCUCAACCCG	CGAGUUGAGCCAGUCUGAGC	Cleavage	1

snoRF Leaves

ncRNA_Acc.	Target_Acc.	Expectation	UPE	ncRNA_start	ncRNA_end	Target_start	Target_end	ncRNA_aligned_fragment	Target_aligned_fragment	Inhibition	Multiplicity
snorf_1_1	Bra031281	3	14.1	1	24	1013	1036	UAUAGUGAUGAAUAUAAUCACAAU	AUGGUGACUAUAGUUAUCACUAUG	Cleavage	1
snorf_1_1	Bra010407	3	18.1	1	25	115	139	UAUAGUGAUGAAUAUAAUCACAAUU	AGUGAUUAUUGUGUUCAUCACUGUG	Cleavage	1
snorf_1_2	Bra033534	3	12.5	1	23	1050	1072	UGGCAGUGAUGAUUGAGAAUGUC	GACAAUCUCAAACAUCACUGCUG	Cleavage	1
snorf_1_2	Bra012741	3	15.1	1	23	531	553	UGGCAGUGAUGAUUGAGAAUGUC	GACAAUCUCAAACAUCACUGCUG	Cleavage	1
snorf_1_2	Bra010085	3	4.5	1	22	531	552	UGGCAGUGAUGAUUGAGAAUGU	ACUUUCUCCAUCAUCAUUGUCA	Cleavage	1

snorf_1_2	Bra026106	3	13.2	1	20	2324	2343	UGGCAGUGAUGAUUGAGAAU	CUGCUCAAUCAUCGCUGCUA	Cleavage	1
snorf_1_4	Bra030023	2.5	20.7	1	21	1214	1234	AAAGAUGAUGAAUAUGCACUG	CAGUGUUUAUUCAUCAUCUUG	Cleavage	1
snorf_1_4	Bra036097	2.5	20.1	1	21	1397	1417	AAAGAUGAUGAAUAUGCACUG	CAGUGUUUAUUCAUCAUCUUG	Cleavage	1
snorf_1_4	Bra036690	3	15.2	1	23	1126	1148	AAAGAUGAUGAAUAUGCACUGGG	CCAAAUGCAUCAUCAUCAUCUUU	Cleavage	1
snorf_1_5	Bra036097	2.5	20.1	1	20	1398	1417	AAAGAUGAUGAAUAUGCACU	AGUGUUUAUUCAUCAUCUUG	Cleavage	1
snorf_1_5	Bra030023	2.5	20.7	1	20	1215	1234	AAAGAUGAUGAAUAUGCACU	AGUGUUUAUUCAUCAUCUUG	Cleavage	1
snorf_1_5	Bra036690	3	15.2	1	20	1129	1148	AAAGAUGAUGAAUAUGCACU	AAUGCAUCAUCAUCAUCUUU	Cleavage	1
snorf_1_6	Bra022523	3	16.4	1	20	288	307	GAGCUGUGAUGAUAUUAGGC	CCCUAAUGUUGUCACAGUUC	Cleavage	1
snorf_1_6	Bra035613	2.5	20.7	1	20	153	172	GAGCUGUGAUGAUAUUAGGC	GCCUAGUCUCGUCACGGCUC	Cleavage	1
snorf_1_6	Bra000855	3	12.5	1	20	1892	1911	GAGCUGUGAUGAUAUUAGGC	GUUUAAUAAUAUCACAGCUU	Cleavage	1
snorf_1_6	Bra032852	3	18.5	1	20	857	876	GAGCUGUGAUGAUAUUAGGC	GUCCGAUAUCAUUACAGCUU	Cleavage	1
snorf_1_6	Bra026759	3	18.1	1	20	203	222	GAGCUGUGAUGAUAUUAGGC	GUCUAAUCACAUCACAGUUC	Cleavage	1
snorf_1_6	Bra004297	3	19.0	1	20	621	640	GAGCUGUGAUGAUAUUAGGC	GCCUGAUCUCAUGACAGUUC	Cleavage	1
snorf_1_7	Bra000733	3	11.7	1	26	817	841	GAAAGUGAUGAUAAGGAAUUGUCGCC	GGCGA-AGUUCCUUGUCAUCCUUUUC	Cleavage	1
snorf_1_7	Bra001673	3	4.3	1	21	396	416	GAAAGUGAUGAUAAGGAAUUG	CAAUUCUUUCUCAUCUCUUUC	Cleavage	1
snorf_1_7	Bra019926	3	24.4	1	20	284	303	GAAAGUGAUGAUAAGGAAUU	CAUUCGUUGUCGUCACUUUC	Cleavage	1
snorf_	Bra001	3	4.	1	21	396	416	GAAAGUGAUGAUAAGG	CAAUUCUUUCUCAUCU	Cleava	1

1_8	673		3					AAUUG	CUUUC	ge	
snorf_	Bra000		11					GAAAGUGAUGAUAAGG	AGUUCCUUGUCAUCCU	Cleava	
1_8	733	3	.7	1	20	822	841	AAUU	UUUC	ge	1
snorf_	Bra019		24					GAAAGUGAUGAUAAGG	CAUUCGUUGUCGUCAC	Cleava	
1_8	926	3	.4	1	20	284	303	AAUU	UUUC	ge	1
snorf_	Bra026		14					AUACAGUGAUGAGAUA	UUCUUGUCACGUCACU	Cleava	
1_9	860	3	.7	1	20	488	507	AGCA	GUAU	ge	1
snorf_	Bra039		15					CUAAAUGGGAAUCUCU	UCAGGGAGAUUCUCAU	Cleava	
1_10	448	1	.3	1	20	820	839	CUGA	UUAG	ge	1
snorf_	Bra034		14					CUAAAUGGGAAUCUCU	CCAGAGAGAUUCUUAU	Cleava	
1_10	045	2	.4	1	20	135	154	CUGA	UUAG	ge	1
snorf_	Bra029		14					CUAAAUGGGAAUCUCU	CCAGAGAAAUUCUUAU	Cleava	
1_10	738	3	.8	1	20	135	154	CUGA	UUAG	ge	1
snorf_	Bra039		16					AAAUGGGAAUCUCUCU	ACUCAGGGAGAUUCUC	Cleava	
1_11	448	3	.6	1	20	818	837	GAUG	AUUU	ge	1
snorf_	Bra003		16					AAAUGGGAAUCUCUCU	CACCGGAGAGAUUCCU	Cleava	
1_11	227	2.5	.1	1	20	888	907	GAUG	GUUU	ge	1
snorf_	Bra035		21					AAAUGGGAAUCUCUCU	CAUGAGAGAGGUUUCC	Cleava	
1_11	134	3	.8	1	20	2055	2074	GAUG	AUUG	ge	1
snorf_	Bra007		10					AAAUGGGAAUCUCUCU	CACCAGGUAGAUUCCC	Cleava	
1_11	474	2.5	.5	1	20	14	33	GAUG	AUUU	ge	1
snorf_	Bra036		18					UCAAAGCAAUGUCUAG	AGAUCUAGACAUUGCC	Cleava	
1_13	154	2	.5	1	20	1803	1822	AUUU	UUGA	ge	1
snorf_	Bra038		18					UCAAAGCAAUGUCUAG	AAAUCGAGACGUUGCU	Cleava	
1_13	386	2	.9	1	20	102	121	AUUU	UUGG	ge	1
snorf_	Bra022		18					UCAAAGCAAUGUCUAG	AGAUUUAGACAUUGUC	Cleava	
1_13	435	3	.2	1	20	289	308	AUUU	UUGA	ge	1
snorf_	Bra007		18					UCAAAGCAAUGUCUAG	GAAUCUACACAUUGCG	Cleava	
1_13	634	3	.3	1	20	717	736	AUUU	UUGA	ge	1
snorf_	Bra005		20					UCAAAGCAAUGUCUAG	AAAUCUACACAUUGCA	Cleava	
1_13	506	3	.3	1	20	233	252	AUUU	UUGG	ge	1
snorf_	Bra035		17					UCAAAGCAAUGUCUAG	AAAUCAAGGUAUAGCU	Cleava	
1_13	465	3	.4	1	20	709	728	AUUU	UUGA	ge	1
snorf_	Bra036	3	24	1	20	2009	2029	UCAAAGCAAUGUC-	GAAUCAAAGACAUUGC	Cleava	1

1_13	313		.2					UAGAUUU	UUUGA	ge	
snorf_	Bra039		15					CUAAAUGGGAAUCUCU	UCAGGGAGAUUCUCAU	Cleava	
1_14	448	1	.3	1	20	820	839	CUGA	UUAG	ge	1
snorf_	Bra034		14					CUAAAUGGGAAUCUCU	CCAGAGAGAUUCUUAU	Cleava	
1_14	045	2	.4	1	20	135	154	CUGA	UUAG	ge	1
snorf_	Bra029		14					CUAAAUGGGAAUCUCU	CCAGAGAAAUUCUUAU	Cleava	
1_14	738	3	.8	1	20	135	154	CUGA	UUAG	ge	1
snorf_	Bra024		20					CUUCCUUGGAUGUC-	GGUUUCGUGACAUUCA	Cleava	
1_16	547	3	.3	1	21	151	172	UGAAACC	AGGAAG	ge	1
snorf_	Bra020		10					CUUCCUUGGAUGUCUG	GUUUUGGACAUUAGA	Cleava	
1_16	090	3	.7	1	20	69	88	AAAC	GGAAG	ge	1
snorf_	Bra015		22					CUUCCUUGGAUGUCUG	GUUCCAGACGUCAAGG	Cleava	
1_16	499	3	.8	1	20	1186	1205	AAAC	GAAG	ge	1
snorf_	Bra030		20					CUUCCUUGGAUGUCUG	GUUCCAGACGUCAAGG	Cleava	
1_16	652	3	.0	1	20	1195	1214	AAAC	GAAG	ge	1
snorf_	Bra012		15					CUUCCUUGGAUG-	UUUCAGAUCAUUCGAG	Cleava	
1_16	536	3	.3	1	19	555	574	UCUGAAA	GAAG	ge	1
snorf_	Bra011		22					CUUCCUUGGAUGUCUG	GUUUGAAACAUCAAAG	Cleava	
1_16	271	3	.0	1	20	1170	1189	AAAC	GAAG	ge	1
snorf_	Bra006		19					AACGAGGCAUUUGUCU	UCUCCGACAAAUGCCU	Cleava	
1_17	972	3	.7	1	20	383	402	GAGA	UUUU	ge	1
snorf_	Bra006		13					AACGAGGCAUUUGUCU	UCUCCGACAAAUGCCU	Cleava	
1_17	971	3	.6	1	20	383	402	GAGA	UUUU	ge	1
snorf_	Bra007		16					AACGAGGCAUUUGUCU	CUCUCAGACAGAU-	Cleava	
1_17	284	3	.8	1	21	1664	1683	GAGAG	CUUCGUU	ge	1
snorf_	Bra010		19					UCUACCCAUAUUAUAU	UCAGAUAUAAUGUGGG	Cleava	
1_18	959	2	.5	1	20	565	584	CUGA	CAGA	ge	1
snorf_	Bra010		19					CUACCCAUAUUAUAUC	AUCAGAUAUAAUGUGG	Cleava	
1_20	959	3	.9	1	20	564	583	UGAG	GCAG	ge	1
snorf_	Bra013		16					UGAUUAAAAACUAUCA	UCGGAGUUGA-	Cleava	
1_25	275	2.5	.1	1	23	1784	1805	GCUCUGA	AGUUUUUAAUCA	ge	1
snorf_	Bra019		14					UGAUUAAAAACU-	AGAGCUGAUGAGUUU	Cleava	
1_25	178	2.5	.9	1	21	1696	1717	AUCAGCUCU	UUGAUCA	ge	1
snorf_	Bra013	3	11	1	20	163	182	UGAUUAAAAACUAUCA	GGGUUGGAAGUUUUU	Cleava	1

1_25	867		.2					GCUC	GAUCA	ge	
snorf_	Bra003		17					UAUGGAGACCUGAACU	CUCUCACUCAGUGCAG	Cleava	
1_26	395	3	.1	1	25	942	966	GAGUGGAGG	GUCAUCAUA	ge	1
snorf_	Bra014		14					UAUGGAGACCUGAACU	CUCUCACUCAGUACAG	Cleava	
1_26	523	3	.6	1	25	942	966	GAGUGGAGG	GUCAUCAUA	ge	1
snorf_	Bra007		10					UAUGGAGACCUGAACU	CUCUCACUCAGUACAG	Cleava	
1_26	493	3	.1	1	25	942	966	GAGUGGAGG	GUCAUCAUA	ge	1
snorf_	Bra011		21					CGAAUAA-	UUCUUCAGAAUUUGAG	Cleava	
1_27	396	2.5	.4	1	22	1182	1204	UCAAAUUCUGAGGGA	UUAUUCG	ge	1
snorf_	Bra035		18					CGAAUAAUCAAAUUCU	UCUCAGGAUUUGGUUA	Cleava	
1_27	606	3	.6	1	20	487	506	GAGG	UUGG	ge	1
snorf_	Bra026		19					CGAAUAAUCAAAUUCU	CAUUGAGGAUUUGAU	Cleava	
1_27	200	3	.1	1	21	561	581	GAGGG	UAUUCG	ge	1
snorf_	Bra032		15					AUGUAUUAGCUCUAUC	GCAUUGGAUGGAGCUG	Cleava	
1_29	239	2	.5	1	22	391	412	UGAUCC	AUACAU	ge	1
snorf_	Bra014		17					AUGUAUUAGCUCUAUC	UUUGGAUGGAGCUGA	Cleava	
1_29	099	3	.9	1	20	393	412	UGAU	UACAU	ge	1
snorf_	Bra009		16					AUGUAUUAGCUCUAUC	AUCAGAUAGAGCUAAA	Cleava	
1_29	459	3	.0	1	20	801	820	UGAU	GCAG	ge	1
snorf_	Bra019		18					AUGUAUUAGCUCUAUC	GUCACAUAGGGCUAAU	Cleava	
1_29	803	2.5	.7	1	20	255	274	UGAU	GCAU	ge	1
snorf_	Bra032		15					AUGUAUUAGCUCUAUC	CUGCAUUGGAUGGAGC	Cleava	
1_30	239	2	.5	1	24	389	412	UGAUCCUG	UGAUACAU	ge	1
snorf_	Bra014		17					AUGUAUUAGCUCUAUC	UUUGGAUGGAGCUGA	Cleava	
1_30	099	3	.9	1	20	393	412	UGAU	UACAU	ge	1
snorf_	Bra009		16					AUGUAUUAGCUCUAUC	AUCAGAUAGAGCUAAA	Cleava	
1_30	459	3	.0	1	20	801	820	UGAU	GCAG	ge	1
snorf_	Bra019		18					AUGUAUUAGCUCUAUC	GUCACAUAGGGCUAAU	Cleava	
1_30	803	2.5	.7	1	20	255	274	UGAU	GCAU	ge	1
snorf_	Bra039		18					UGUUACACACACUGAG	UAUCAUCUGUGUGUG	Cleava	
1_31	706	3	.2	1	20	732	751	GCUA	UAACA	ge	1
snorf_	Bra014		24					UCAAGUUUGGCUAUUC	UGCAAAAGAAUGGCCA	Cleava	
1_35	985	3	.0	1	23	303	325	UGUUACA	AGCUUGC	ge	1
snorf_	Bra027	2.5	13	1	20	781	800	UCAAGUUUGGCUAUUC	GACACAAUAGCUAAGC	Cleava	1

1_35	883		.4					UGUU	UUGA	ge	
snorf_	Bra026		15					UCAAGUUUGGCUAUUC	UAUCAGAAUUGUCAAG	Cleava	
1_35	682	3	.4	1	21	1787	1807	UGUUA	CUUGA	ge	1
snorf_	Bra026		14					UCAAGUUUGGCUAUUC	UAUCAGAAUUGUCAAG	Cleava	
1_35	977	3	.1	1	21	1886	1906	UGUUA	CUUGA	ge	1
snorf_	Bra025		18					ACUGUUUUACUCGGUG	CUGCAGCCAAGUAGAA	Cleava	
1_36	882	3	.7	1	20	1332	1351	GCAG	CAGU	ge	1
snorf_	Bra015		22					CUAGAAAGCUUAAUCU	GAUCAGAUUAGGGUU	Cleava	
1_39	786	3	.5	1	20	331	350	GAUU	UUUGG	ge	1
snorf_	Bra027		19					CUAGAAAGCUUAAUCU	AAGAAGGUUAAGCUUU	Cleava	
1_39	369	2.5	.2	1	20	505	524	GAUU	CUAG	ge	1
snorf_	Bra028		18					CUAGAAAGCUUAAUCU	AUUCAGAUUAGGGUU	Cleava	
1_39	595	3	.7	1	20	645	664	GAUU	UCUGG	ge	1
snorf_	Bra030		11					AUUUUAUUUCUCUGA	GAUCAUGAGAGGAAUG	Cleava	
1_40	997	2.5	.5	1	20	772	791	UGAUC	AGAU	ge	1
snorf_	Bra030		16					AUUUUAUUUCUCUGA	GAUCAUGAGAGGAAUG	Cleava	
1_40	998	2.5	.2	1	20	3109	3128	UGAUC	AGAU	ge	1
snorf_	Bra038		15					AUUUUAUUUCUCUGA	GAUCUUAAGAGAAAUA	Cleava	
1_40	921	2.5	.8	1	20	232	251	UGAUC	AGAU	ge	1
snorf_	Bra024		15					AUUUUAUUUCUCUGA	GAUCAUCAAAGAAGUG	Cleava	
1_40	202	3	.1	1	20	1338	1357	UGAUC	GAGU	ge	1
snorf_	Bra015		15					AUUUUAUUUCUCUGA	GGUUAUCAAGGAAAUG	Cleava	
1_40	229	3	.5	1	20	855	874	UGAUC	AAAU	ge	1
snorf_	Bra025		10					AUUUUAUUUCUCUGA	GAUCAAGAGAGAAAUA	Cleava	
1_40	087	3	.9	1	20	1056	1075	UGAUC	AAAG	ge	1
snorf_	Bra038		8.					UGAUUGACUAUUUUCG	GAUCCAGAGGAAAAUG	Cleava	
1_41	991	1.5	0	1	24	149	172	UCUGAUUC	GUCAAUCA	ge	1
snorf_	Bra028		16					UGAUUGACUAUUUUCG	UCAGAUGAAGAUAUUC	Cleava	
1_41	512	2	.1	1	21	754	774	UCUGA	AAUCA	ge	1
snorf_	Bra011		19					UGAUUGACUAUUUUCG	UUGGACGAUAAUAAUC	Cleava	
1_41	790	3	.9	1	21	697	717	UCUGA	AAUCA	ge	1
snorf_	Bra025		20					UGAUUGACUAUUUUCG	UCUGAUGAAGAUAUUC	Cleava	
1_41	458	3	.7	1	21	844	864	UCUGA	AAUCA	ge	1
snorf_	Bra016	3	16	1	20	632	651	UGAUUGACUAUUUUCG	CAUAUGCAAAUGGUCA	Cleava	1

1_41	942		.5					UCUG	AUCA	ge	
snorf_	Bra028		14					AUUGACUAUUUUCGUC	GGUCAGAGGAAAAGAG	Cleava	
1_42	945	2.5	.7	1	21	1924	1944	UGAUU	UCAAU	ge	1
snorf_	Bra034		13					AUUGACUAUUUUCGUC	ACUCAGACUAAGAUGG	Cleava	
1_42	795	3	.9	1	21	1072	1092	UGAUU	UCAAU	ge	1
snorf_	Bra024		20					AUUGACUAUUUUCGUC	GAUAAGACGAAAAGAG	Cleava	
1_42	432	3	.2	1	21	424	444	UGAUU	UCAAG	ge	1
snorf_	Bra038		7.					GAUUGACUAUUUUCGU	GAUCCAGAGGAAAAUG	Cleava	
1_43	991	2.5	5	1	23	149	171	CUGAUUC	GUCAAUC	ge	1
snorf_	Bra028		16					GAUUGACUAUUUUCGU	UCAGAUGAAGAUAUUC	Cleava	
1_43	512	2.5	.0	1	20	754	773	CUGA	AAUC	ge	1
snorf_	Bra034		13					GAUUGACUAUUUUCGU	ACUCAGACUAAGAUGG	Cleava	
1_43	795	3	.3	1	22	1072	1093	CUGAUU	UCAAUG	ge	1
snorf_	Bra038		8.					GUGAUUGACUAUUUUC	GAUCCAGAGGAAAAUG	Cleava	
1_44	991	2	7	1	25	149	173	GUCUGAUUC	GUCAAUCAU	ge	1
snorf_	Bra026		17					GUGAUUGACUAUUUUC	AGAUGAAAGUAGCCAG	Cleava	
1_44	885	3	.6	1	20	1124	1143	GUCU	UUAC	ge	1
snorf_	Bra039		15					CUAAAUGGGAAUCUCU	UCAGGGAGAUUCUCAU	Cleava	
1_48	448	1	.3	1	20	820	839	CUGA	UUAG	ge	1
snorf_	Bra034		14					CUAAAUGGGAAUCUCU	CCAGAGAGAUUCUUAU	Cleava	
1_48	045	2	.4	1	20	135	154	CUGA	UUAG	ge	1
snorf_	Bra029		14					CUAAAUGGGAAUCUCU	CCAGAGAAAUUCUUAU	Cleava	
1_48	738	3	.8	1	20	135	154	CUGA	UUAG	ge	1
snorf_	Bra039		16					AAAUGGGAAUCUCUCU	ACUCAGGGAGAUUCUC	Cleava	
1_49	448	3	.6	1	20	818	837	GAUG	AUUU	ge	1
snorf_	Bra003		16					AAAUGGGAAUCUCUCU	CACCGGAGAGAUUCCU	Cleava	
1_49	227	2.5	.1	1	20	888	907	GAUG	GUUU	ge	1
snorf_	Bra035		21					AAAUGGGAAUCUCUCU	CAUGAGAGAGGUUUCC	Cleava	
1_49	134	3	.8	1	20	2055	2074	GAUG	AUUG	ge	1
snorf_	Bra007		10					AAAUGGGAAUCUCUCU	CACCAGGUAGAUUCCC	Cleava	
1_49	474	2.5	.5	1	20	14	33	GAUG	AUUU	ge	1
snorf_	Bra004		20					AUCCUUCCUUGGAUGU	UAUCAGACGUUCAAGG	Cleava	
1_50	473	2	.8	1	22	647	668	CUGAAA	AGGGGU	ge	1
snorf_	Bra029	1.5	20	1	20	1518	1537	AUCCUUCCUUGGAUGU	UCAGACGACCAAGGAA	Cleava	1

1_50	073		.1					CUGA	GGAU	ge	
snorf_	Bra019		17					AUCCUUCCUUGGAUGU	UCCAGGCAUCCGGGGA	Cleava	
1_50	832	2.5	.3	1	21	493	513	CUGAA	AGGAU	ge	1
snorf_	Bra001		23					AUCCUUCCUUGGAUGU	AUUUUCAGAGAUCCGA	Cleava	
1_50	797	3	.7	1	24	158	181	CUGAAACU	AGGAGGAU	ge	1
snorf_	Bra009		18					AUCCUUCCUUGGAUGU	UCAGGCUUCCAGGGAA	Cleava	
1_50	240	2	.3	1	20	685	704	CUGA	GGAU	ge	1
snorf_	Bra003		19					AUCCUUCCUUGGAUGU	UCGGACGACCAAGGAG	Cleava	
1_50	063	2.5	.4	1	20	1602	1621	CUGA	GGAU	ge	1
snorf_	Bra006		22					AUCCUUCCUUGGAUGU	UCAGGUGUCCGAUGAA	Cleava	
1_50	952	3	.5	1	20	968	987	CUGA	GGAU	ge	1
snorf_	Bra035		14					AUCCUUCCUUGGAUGU	UCAGACCUCCAAAGAA	Cleava	
1_50	233	3	.9	1	20	317	336	CUGA	GGAG	ge	1
snorf_	Bra023		18					UGGCAGUGAUGAUUG	UGUUCUCAAUGAUGAU	Transl	
1_2	739	3	.4	1	21	879	899	AGAAUG	UGCCA	ation	1
snorf_	Bra040		17					GAGCUGUGA-	UCUGAUAUCAAUCACA	Transl	
1_6	986	3	.9	1	19	3393	3412	UGAUAUUAGG	GCUC	ation	1
snorf_	Bra000		16					GAAAGUGAUGAUAAGG	CAAUUCCUUUUGAUCA	Transl	
1_7	657	2.5	.6	1	21	37	57	AAUUG	UUUUC	ation	1
snorf_	Bra013		5.					GAAAGUGAUGAUAAGG	CUAAAAUUUCUUAUCC	Transl	
1_7	119	3	8	1	24	853	876	AAUUGUCG	UCUCUUUC	ation	1
snorf_	Bra039		11					GAAAGUGAUGAUAAGG	UAAUGUCCUUAACAUC	Transl	
1_7	389	3	.8	1	21	1248	1269	A-AUUG	ACUUUC	ation	1
snorf_	Bra000		16					GAAAGUGAUGAUAAGG	CAAUUCCUUUUGAUCA	Transl	
1_8	657	2.5	.6	1	21	37	57	AAUUG	UUUUC	ation	1
snorf_	Bra013		5.					GAAAGUGAUGAUAAGG	AAAAUUUCUUAUCCUC	Transl	
1_8	119	3	8	1	22	855	876	AAUUGU	UCUUUC	ation	1
snorf_	Bra039		11					GAAAGUGAUGAUAAGG	UAAUGUCCUUAACAUC	Transl	
1_8	389	3	.8	1	21	1248	1269	A-AUUG	ACUUUC	ation	1
snorf_	Bra016		17					AUACAGUGAUGAGAUA	UUUGUUUAUCUGAUU	Transl	
1_9	643	2.5	.3	1	22	82	103	AGCAGA	ACUGUGU	ation	1
snorf_	Bra012		15					AUACAGUGAUGAGAUA	CUGCUUGUCUUCUUGC	Transl	
1_9	204	3	.6	1	21	377	397	AGCAG	UGUAU	ation	1
snorf_	Bra030	2.5	17	1	20	432	451	UCAAAGCAAUGUCUAG	AGGUCUAGAGGUUGCU	Transl	1

1_13	700		.6					AUUU	UUGA	ation	
snorf_	Bra005		21					UCAAAGCAAUGUCUAG	GGAGCUAGAGAUUGCU	Transl	
1_13	206	3	.1	1	20	2649	2668	AUUU	UUGA	ation	1
snorf_	Bra013		19					UCAAAGCAAUGUCUAG	AAGUUUAGAC-	Transl	
1_13	921	3	.1	1	20	592	610	AUUU	UUGCUUUGA	ation	1
snorf_	Bra001		17					CUUCCUUGGAUGUCUG	GUUUCAGGUAGCCAAG	Transl	
1_16	502	3	.6	1	20	520	539	AAAC	GAAA	ation	1
snorf_	Bra003		16					CUUCCUUGGAUGUCUG	GUUUCAGACAAUCAAC	Transl	
1_16	592	3	.0	1	20	282	301	AAAC	GAAG	ation	1
snorf_	Bra035		15					CUUCCUUGGAUGUCUG	GUUUCAGACAAUCAAC	Transl	
1_16	143	3	.7	1	20	282	301	AAAC	GAAG	ation	1
snorf_	Bra002		13					AACGAGGCAUUUGUCU	UUCUCUUAGACAAAAA	Transl	
1_17	768	3	.9	1	23	1583	1605	GAGAGAA	CUUCGUU	ation	1
snorf_	Bra009		18					AACGAGGC-	UUUGCUCAGAUAAAUA	Transl	
1_17	103	3	.4	1	23	1009	1032	AUUUGUCUGAGAGAA	GCUUCGUU	ation	1
snorf_	Bra039		17					UCUACCCAUAUUAUAU	UCAGAUAUAAAGUGGG	Transl	
1_18	525	3	.0	1	20	2797	2816	CUGA	AAGA	ation	1
snorf_	Bra037		23					UGAUUAAAAACUAUCA	UCUGAAGAGCUGAUGU	Transl	
1_25	647	3	.4	1	26	1570	1595	GCUCUGAGGG	CUUUUGAUCA	ation	1
snorf_	Bra001		15					UAUGGAGACCUGAACU	UUCAUUCAGUUUAGAU	Transl	
1_26	667	3	.3	1	23	189	211	GAGUGGA	UUCCAUG	ation	1
snorf_	Bra024		23					UAUGGAGACCUGAACU	ACUCAGUUCGAGUCUC	Transl	
1_26	602	3	.3	1	20	593	612	GAGU	CAAA	ation	1
snorf_	Bra016		17					UAUGGAGACCUGAACU	ACUCAGUUCGAGUCUC	Transl	
1_26	345	3	.3	1	20	671	690	GAGU	CAAA	ation	1
snorf_	Bra003		22					UGUUACACACACUGAG	UAGUCUCGGCGCGUGU	Transl	
1_31	960	3	.6	1	20	873	892	GCUA	AACA	ation	1
snorf_	Bra040		17					UCAAGUUUGGCUAUUC	GCAUCUCACAGAAUAG	Transl	
1_35	779	3	.4	1	26	871	896	UGUUACAUUC	CAGAAUUUGA	ation	1
snorf_	Bra002		15					UCAAGUUUGGCUAUUC	AAUGGAAUAGCAAAAC	Transl	
1_35	352	2	.8	1	20	841	860	UGUU	UUGA	ation	1
snorf_	Bra035		10					UCAAGUUUGGCUAUUC	UCACAGAAUAGCAGAA	Transl	
1_35	539	2.5	.0	1	21	1092	1112	UGUUA	CUUGA	ation	1
snorf_	Bra011	2	9.	1	20	499	518	UCAAGUUUGGCUAUUC	AACAAAAUAGGCAAAC	Transl	1

1_35	260		2					UGUU	UUGA	ation	
snorf_	Bra007		17					UCAAGUUUGGCUAUUC	AACAGAAUAGCAAAAC	Transl	
1_35	263	2.5	.3	1	20	1363	1382	UGUU	AUGA	ation	1
snorf_	Bra032		17					ACUGUUUUACUCGGUG	UCUCUUCCACCGCGUC	Transl	
1_36	960	3	.8	1	23	521	543	GCAGAAA	AAACAGU	ation	1
snorf_	Bra027		19					ACUGUUUUACUCGGUG	CUGCCACUGAGGUAAA	Transl	
1_36	641	2.5	.4	1	20	2087	2106	GCAG	CAGU	ation	1
snorf_	Bra034		21					CUAGAAAGCUUAAUCU	AAGCAGAUUAUGUUUU	Transl	
1_39	403	3	.9	1	20	482	501	GAUU	CUGG	ation	1
snorf_	Bra002		15					AUUUUAUUUCUCUGA	UAUCAUCAGUGAAAUA	Transl	
1_40	333	3	.8	1	20	2337	2356	UGAUC	AAAC	ation	1
snorf_	Bra024		13					AUUUUAUUUCUCUGA	GAUCAUCAGAGCAAUA	Transl	
1_40	670	3	.0	1	20	504	523	UGAUC	GGAG	ation	1
snorf_	Bra014		20					AUUUUAUUUCUCUGA	GAUCAUCAGAUAGAGA	Transl	
1_40	869	3	.1	1	20	166	185	UGAUC	AAAU	ation	1
snorf_	Bra028		18					AUUUUAUUUCUCUGA	GAUCAUGAGAAAGAUG	Transl	
1_40	506	3	.3	1	20	55	74	UGAUC	AAAU	ation	1
snorf_	Bra011		17					UGAUUGACUAUUUUCG	AUCAGAGGAAAUUAGU	Transl	
1_41	753	3	.5	1	22	96	117	UCUGAU	CAAUCU	ation	1
snorf_	Bra005		15					UGAUUGACUAUUUUCG	GGUCAGAGGAAAAAAG	Transl	
1_41	646	3	.8	1	23	318	340	UCUGAUU	UCGGUCA	ation	1
snorf_	Bra011		15					AUUGACUAUUUUCGUC	AUCAGAGGAAAUUAGU	Transl	
1_42	753	2	.2	1	20	96	115	UGAU	CAAU	ation	1
snorf_	Bra011		16					GAUUGACUAUUUUCGU	AUCAGAGGAAAUUAGU	Transl	
1_43	753	2	.9	1	21	96	116	CUGAU	CAAUC	ation	1
snorf_	Bra028		16					GAUUGACUAUUUUCGU	GGUCAGAGGAAAAGAG	Transl	
1_43	945	3	.0	1	22	1924	1945	CUGAUU	UCAAUG	ation	1
snorf_	Bra005		15					GAUUGACUAUUUUCGU	GGUCAGAGGAAAAAAG	Transl	
1_43	646	3	.3	1	22	318	339	CUGAUU	UCGGUC	ation	1
snorf_	Bra039		24					GAUUGACUAUUUUCGU	UCAGAGGAGAGAAGUC	Transl	
1_43	494	3	.5	1	20	1051	1070	CUGA	AAUC	ation	1
snorf_	Bra028		16					GUGAUUGACUAUUUUC	UCAGAUGAAGAUAUUC	Transl	
1_44	512	3	.1	1	22	754	775	GUCUGA	AAUCAG	ation	1
snorf_	Bra011	3	22	1	22	697	718	GUGAUUGACUAUUUUC	UUGGACGAUAAUAAUC	Transl	1

1_44	790		.5					GUCUGA	AAUCAU	ation	
snorf_1_50	Bra029373	3	24.9	1	22	94	115	AUCCUUCCUUGGAUGUCUGAAA	UCUCAGAUAGCCACGGGAGGAU	Translation	1

snoRF Apical meristem

ncRNA_Acc.	Target_Acc.	Expectation	UPE	ncRNA_start	ncRNA_end	Target_start	Target_end	ncRNA_aligned_fragment	Target_aligned_fragment	Inhibition	Multiplicity
snorf_2_1	Bra005579	3	17.4	1	22	993	1014	AGCCGAUGAGGACAUCAGAUUA	UGGUCUCAUGUCUUUAUUGGCU	Cleavage	1
snorf_2_1	Bra030320	3	23.9	1	20	42	61	AGCCGAUGAGGACAUCAGAU	GUCUAGUGUCUUCAUUGGCU	Cleavage	1
snorf_2_2	Bra015439	3	16.8	1	22	292	313	AGGCAGUGAUGAUUGAGAAUGU	ACAUUCUCGAUGGUCAUUGCUU	Translation	1
snorf_2_2	Bra000424	2.5	24.3	1	23	903	925	AGGCAGUGAUGAUUGAGAAUGUC	GAAAUUCUCAAGAAUCAUUGCCU	Translation	1
snorf_2_2	Bra040348	2.5	22.4	1	23	435	457	AGGCAGUGAUGAUUGAGAAUGUC	GAAAUUCUCAAGAAUCAUUGCCU	Translation	1
snorf_2_2	Bra019100	3	9.4	1	22	322	343	AGGCAGUGAUGAUUGAGAAUGU	GUAUUCUUCAUCUUCAUUGCCU	Translation	1
snorf_2_2	Bra026423	3	11.8	1	22	304	325	AGGCAGUGAUGAUUGAGAAUGU	GUAUUCUUCAUCUUCAUUGCCU	Translation	1
snorf_2_2	Bra023810	3	11.8	1	22	290	311	AGGCAGUGAUGAUUGAGAAUGU	AAAUCCUCAAUAAUGACUGCCU	Translation	1
snorf_2_3	Bra033534	3	12.5	1	23	1050	1072	UGGCAGUGAUGAUUGAGAAUGUC	GACAAUCUCAAACAUCACUGCUG	Cleavage	1
snorf_2_3	Bra012741	3	15.1	1	23	531	553	UGGCAGUGAUGAUUGAGAAUGUC	GACAAUCUCAAACAUCACUGCUG	Cleavage	1
snorf_2_3	Bra010085	3	4.5	1	22	531	552	UGGCAGUGAUGAUUGAGAAUGU	ACUUUCUCCAUCAUCAUUGUCA	Cleavage	1
snorf_2_3	Bra026106	3	13.2	1	20	2324	2343	UGGCAGUGAUGAUUGAGAAU	CUGCUCAAUCAUCGCUGCUA	Cleavage	1
snorf_	Bra023	3	18	1	21	879	899	UGGCAGUGAUGAUUG	UGUUCUCAAUGAUGA	Transl	1

2_3	739		.4					AGAAUG	UUGCCA	ation	
snorf_	Bra030		15					GCUGUGAAGAUAAGAA	AAUUGUUCCUAUCUU	Cleava	
2_4	271	2.5	.7	1	21	328	348	CAAUU	CAUGGU	ge	1
snorf_	Bra014		10					GCUGUGAAGAUAAGAA	CUUGUUCUCAUCUUCA	Cleava	
2_4	329	3	.3	1	20	517	536	CAAU	UGGC	ge	1
snorf_	Bra014		17					GCUGUGAAGAUAAGAA	AUUGUUCUUCUCUUU	Transl	
2_4	071	3	.3	1	20	469	488	CAAU	ACAAC	ation	1
snorf_	Bra007		20					GCUGUGAAGAUAAGAA	UCAGUGUUCUUAUGU	Transl	
2_4	474	3	.5	1	22	3388	3409	CAAUUA	UUACAGU	ation	1
snorf_	Bra013		20					GCUGUGAAGAUAAGAA	AUUGUUCUGACCUUC	Transl	
2_4	538	3	.2	1	20	536	555	CAAU	ACAGA	ation	1
snorf_	Bra036		20					AAAGAUGAUGAAUAU	AGUGUUUAUUCAUCA	Cleava	
2_6	097	2.5	.1	1	20	1398	1417	GCACU	UCUUG	ge	1
snorf_	Bra030		20					AAAGAUGAUGAAUAU	AGUGUUUAUUCAUCA	Cleava	
2_6	023	2.5	.7	1	20	1215	1234	GCACU	UCUUG	ge	1
snorf_	Bra036		15					AAAGAUGAUGAAUAU	AAUGCAUCAUCAUCAU	Cleava	
2_6	690	3	.2	1	20	1129	1148	GCACU	CUUU	ge	1
snorf_	Bra022		16					GAGCUGUGAUGAUAU	CCCUAAUGUUGUCACA	Cleava	
2_7	523	3	.4	1	20	288	307	UAGGC	GUUC	ge	1
snorf_	Bra035		20					GAGCUGUGAUGAUAU	GCCUAGUCUCGUCACG	Cleava	
2_7	613	2.5	.7	1	20	153	172	UAGGC	GCUC	ge	1
snorf_	Bra000		12					GAGCUGUGAUGAUAU	GUUUAAUAAUAUCACA	Cleava	
2_7	855	3	.5	1	20	1892	1911	UAGGC	GCUU	ge	1
snorf_	Bra032		18					GAGCUGUGAUGAUAU	GUCCGAUAUCAUUACA	Cleava	
2_7	852	3	.5	1	20	857	876	UAGGC	GCUU	ge	1
snorf_	Bra026		18					GAGCUGUGAUGAUAU	GGUCUAAUCACAUCAC	Cleava	
2_7	759	3	.1	1	21	202	222	UAGGCU	AGUUC	ge	1
snorf_	Bra004		19					GAGCUGUGAUGAUAU	GCCUGAUCUCAUGACA	Cleava	
2_7	297	3	.0	1	20	621	640	UAGGC	GUUC	ge	1
snorf_	Bra040		17					GAGCUGUGA-	UCUGAUAUCAAUCACA	Transl	
2_7	986	3	.9	1	19	3393	3412	UGAUAUUAGG	GCUC	ation	1
snorf_	Bra022		16					GAGCUGUGAUGAUAU	CCCUAAUGUUGUCACA	Cleava	
2_9	523	3	.4	1	20	288	307	UAGGC	GUUC	ge	1
snorf_	Bra035	2.5	20	1	20	153	172	GAGCUGUGAUGAUAU	GCCUAGUCUCGUCACG	Cleava	1

2_9	613		.7					UAGGC	GCUC	ge	
snorf_	Bra000		12					GAGCUGUGAUGAUAU	GUUUAAUAAUAUCACA	Cleava	
2_9	855	3	.5	1	20	1892	1911	UAGGC	GCUU	ge	1
snorf_	Bra032		18					GAGCUGUGAUGAUAU	GUCCGAUAUCAUUACA	Cleava	
2_9	852	3	.5	1	20	857	876	UAGGC	GCUU	ge	1
snorf_	Bra026		18					GAGCUGUGAUGAUAU	GUCUAAUCACAUCACA	Cleava	
2_9	759	3	.1	1	20	203	222	UAGGC	GUUC	ge	1
snorf_	Bra004		19					GAGCUGUGAUGAUAU	GCCUGAUCUCAUGACA	Cleava	
2_9	297	3	.0	1	20	621	640	UAGGC	GUUC	ge	1
snorf_	Bra040		17					GAGCUGUGA-	UCUGAUAUCAAUCACA	Transl	
2_9	986	3	.9	1	19	3393	3412	UGAUAUUAGG	GCUC	ation	1
snorf_	Bra026		18					GAGCUGUGAUGAUAU	UAUGGUCUAAUCACAU	Cleava	
2_10	759	3	.1	1	24	199	222	UAGGCUUUA	CACAGUUC	ge	1
snorf_	Bra022		16					GAGCUGUGAUGAUAU	CCCUAAUGUUGUCACA	Cleava	
2_10	523	3	.4	1	20	288	307	UAGGC	GUUC	ge	1
snorf_	Bra035		20					GAGCUGUGAUGAUAU	GCCUAGUCUCGUCACG	Cleava	
2_10	613	2.5	.7	1	20	153	172	UAGGC	GCUC	ge	1
snorf_	Bra004		19					GAGCUGUGAUGAUAU	UUCAUGCCUGAUCUCA	Cleava	
2_10	297	3	.0	1	25	616	640	UAGGCUUUAG	UGACAGUUC	ge	1
snorf_	Bra000		12					GAGCUGUGAUGAUAU	AUGUUUAAUAAUAUC	Cleava	
2_10	855	3	.5	1	22	1890	1911	UAGGCUU	ACAGCUU	ge	1
snorf_	Bra032		18					GAGCUGUGAUGAUAU	GUCCGAUAUCAUUACA	Cleava	
2_10	852	3	.5	1	20	857	876	UAGGC	GCUU	ge	1
snorf_	Bra037		11					GAGCUGUGAUGAUAU	UCCAAAUCUCAUCAUA	Cleava	
2_11	392	3	.0	1	20	319	338	UUGGC	GCUU	ge	1
snorf_	Bra039		19					GAGCUGUGAUGAUAU	GUUGGAUCUCAUCACA	Cleava	
2_11	067	3	.5	1	20	409	428	UUGGC	GCUC	ge	1
snorf_	Bra008		17					GGUCUGUGAUGUUUG	AGUCUCAGACAUCAUA	Cleava	
2_13	901	2.5	.8	1	20	336	355	AGAUU	GGUC	ge	1
snorf_	Bra036		21					GGUCUGUGAUGUUUG	GAUCUCAAACAUCAUA	Cleava	
2_13	315	3	.7	1	20	1323	1342	AGAUU	GGUG	ge	1
snorf_	Bra030		24					GGUCUGUGAUGUUUG	GGUUUCGAACAUUACA	Cleava	
2_13	783	3	.4	1	20	915	934	AGAUU	GAUC	ge	1
snorf_	Bra005	3	13	1	20	198	217	GGUCUGUGAUGUUUG	GAUCUCGAGCUUCAUA	Transl	1

2_13	532		.5					AGAUU	GACC	ation	
snorf_	Bra032		21					GGUCUGUGAUGUUUG	GAUCUCAAGAGUUACA	Transl	
2_13	199	3	.4	1	20	1426	1445	AGAUU	GACC	ation	1
snorf_	Bra040		14					GGUCUGUGAUGUUUG	AAGAUCAAACAUCACA	Cleava	
2_13	723	2.5	.0	1	20	783	802	AGAUU	GAUC	ge	1
snorf_	Bra000		15					GGUCUGUGAUGUUUG	AGUCUCAAACAUGGUA	Cleava	
2_13	563	3	.2	1	20	2185	2204	AGAUU	GACU	ge	1
snorf_	Bra005		6.					GGUCUGUGAUGUUUG	ACUCUCAAACAUCUCA	Cleava	
2_13	952	3	3	1	20	1961	1980	AGAUU	GAUC	ge	1
snorf_	Bra030		14					GGUCUGUGAUGUUUG	AAGCUCAAAUAUCACA	Cleava	
2_13	576	3	.0	1	20	2982	3001	AGAUU	GAGC	ge	1
snorf_	Bra027		19					GGUCUGUGAUGU-	AAUUUCAAGAUAUCAC	Cleava	
2_13	982	3	.5	1	20	2302	2322	UUGAGAUU	AGACC	ge	1
snorf_	Bra038		17					GGUCUGUGAUGUUUG	AAUCU-	Cleava	
2_13	808	3	.3	1	20	610	628	AGAUU	AAACAUCACGGACU	ge	1
snorf_	Bra005		15					GGCCGAUGAUUUAAAU	UUGGAAUUACAUAAA	Cleava	
2_14	417	3	.6	1	24	63	86	AAUUUUGA	UCAUCGGUU	ge	1
snorf_	Bra040		20					AAUGGGAAUCUCUCUG	UGCAUUAGAGCGGUU	Transl	
2_15	463	2.5	.7	1	21	579	599	AUGCA	UCCAUU	ation	1
snorf_	Bra012		23					AAUGGGAAUCUCUCUG	GGCGUAUCAGAGAGA	Cleava	
2_15	699	3	.1	1	23	1021	1043	AUGCACU	UUCUUGUC	ge	1
snorf_	Bra033		20					AAUGGGAAUCUCUCUG	AAUGCCUCACAGAGAU	Cleava	
2_15	876	3	.7	1	23	447	469	AUGCACU	UUUCAUU	ge	1
snorf_	Bra035		21					AAUGGGAAUCUCUCUG	ACAUGAGAGAGGUUU	Cleava	
2_15	134	3	.1	1	20	2054	2073	AUGC	CCAUU	ge	1
snorf_	Bra012		22					AUGGGAAUCUCUCUGA	GGCGUAUCAGAGAGA	Cleava	
2_16	699	3	.5	1	22	1021	1042	UGCACU	UUCUUGU	ge	1
snorf_	Bra030		12					AUGGGAAUCUCUCUGA	AAUACAUCAGAGAGAU	Cleava	
2_16	518	2.5	.2	1	22	155	176	UGCACU	UCUCAG	ge	1
snorf_	Bra040		20					AUGGGAAUCUCUCUGA	UGCAUUAGAGCGGUU	Transl	
2_16	463	2.5	.4	1	20	579	598	UGCA	UCCAU	ation	1
snorf_	Bra040		18					AUGGGAAUCUCUCUGA	UGCAUUAGAGCGGUU	Transl	
2_16	497	2.5	.1	1	20	657	676	UGCA	UCCAU	ation	1
snorf_	Bra002	3	16	1	21	782	802	AUGGGAAUCUCUCUGA	GUGCAAUAGGAAGAU	Transl	1

2_16	806		.3					UGCAC	UCCCAU	ation	
snorf_	Bra033		20					AUGGGAAUCUCUCUGA	AAUGCCUCACAGAGAU	Cleava	
2_16	876	3	.9	1	22	447	468	UGCACU	UUUCAU	ge	1
snorf_	Bra013		19					AUGGGAAUCUCUCUGA	UUCAUCGGAAAGGUU	Transl	
2_16	550	3	.9	1	20	463	482	UGCA	CCCAU	ation	1
snorf_	Bra020		23					AUGGGAAUCUCUCUGA	UUCAUCGGAAAGGUU	Transl	
2_16	911	3	.5	1	20	475	494	UGCA	CCCAU	ation	1
snorf_	Bra039		15					CUAAAUGGGAAUCUCU	UCAGGGAGAUUCUCA	Cleava	
2_17	448	1	.3	1	20	820	839	CUGA	UUUAG	ge	1
snorf_	Bra034		14					CUAAAUGGGAAUCUCU	CCAGAGAGAUUCUUAU	Cleava	
2_17	045	2	.4	1	20	135	154	CUGA	UUAG	ge	1
snorf_	Bra029		14					CUAAAUGGGAAUCUCU	CCAGAGAAAUUCUUAU	Cleava	
2_17	738	3	.8	1	20	135	154	CUGA	UUAG	ge	1
snorf_	Bra012		22					AUGGGAAUCUCUCUGA	GGCGUAUCAGAGAGA	Cleava	
2_18	699	3	.5	1	22	1021	1042	UGCACU	UUCUUGU	ge	1
snorf_	Bra030		12					AUGGGAAUCUCUCUGA	AAUACAUCAGAGAGAU	Cleava	
2_18	518	2.5	.2	1	22	155	176	UGCACU	UCUCAG	ge	1
snorf_	Bra040		20					AUGGGAAUCUCUCUGA	UGCAUUAGAGCGGUU	Transl	
2_18	463	2.5	.4	1	20	579	598	UGCA	UCCAU	ation	1
snorf_	Bra040		18					AUGGGAAUCUCUCUGA	UGCAUUAGAGCGGUU	Transl	
2_18	497	2.5	.1	1	20	657	676	UGCA	UCCAU	ation	1
snorf_	Bra002		16					AUGGGAAUCUCUCUGA	GUGCAAUAGGAAGAU	Transl	
2_18	806	3	.3	1	21	782	802	UGCAC	UCCCAU	ation	1
snorf_	Bra033		20					AUGGGAAUCUCUCUGA	AAUGCCUCACAGAGAU	Cleava	
2_18	876	3	.9	1	22	447	468	UGCACU	UUUCAU	ge	1
snorf_	Bra013		19					AUGGGAAUCUCUCUGA	UUCAUCGGAAAGGUU	Transl	
2_18	550	3	.9	1	20	463	482	UGCA	CCCAU	ation	1
snorf_	Bra020		23					AUGGGAAUCUCUCUGA	UUCAUCGGAAAGGUU	Transl	
2_18	911	3	.5	1	20	475	494	UGCA	CCCAU	ation	1
snorf_	Bra039		15					CUAAAUGGGAAUCUCU	UCAGGGAGAUUCUCA	Cleava	
2_19	448	1	.3	1	20	820	839	CUGA	UUUAG	ge	1
snorf_	Bra034		14					CUAAAUGGGAAUCUCU	CCAGAGAGAUUCUUAU	Cleava	
2_19	045	2	.4	1	20	135	154	CUGA	UUAG	ge	1
snorf_	Bra029	3	14	1	20	135	154	CUAAAUGGGAAUCUCU	CCAGAGAAAUUCUUAU	Cleava	1

2_19	738		.8					CUGA	UUAG	ge	
snorf_	Bra029		22					CCCAUAUUAUAUCUGA	AGGCCCAGAUCUAAUA	Transl	
2_23	293	3	.4	1	20	655	674	GCCU	UGGC	ation	1
snorf_	Bra009		20					AAUUGGAGACCUGAAC	AGCUGUUAAGUUCAG	Cleava	
2_28	567	2.5	.1	1	25	906	930	UGAAGAGUU	GUGUCCGAUU	ge	1
snorf_	Bra017		14					AAUUGGAGACCUGAAC	AACAGUUCAGGUCUCC	Cleava	
2_28	838	3	.7	1	20	546	565	UGAA	AAUG	ge	1
snorf_	Bra028		14					AUGGAGACCUGAACUG	AAAGCUUUAAUUCGG	Cleava	
2_30	460	3	.5	1	23	4537	4559	AAGAGUU	GUCUCCAU	ge	1
snorf_	Bra016		20					AUGGAGACCUGAACUG	GGUUCUUCAGUU-	Transl	
2_30	932	3	.3	1	23	9	30	AAGAGUU	AGGUCUCCAG	ation	1
snorf_	Bra022		17					AUGGAGACCUGAACUG	UUCUUCACUUCAAGUC	Transl	
2_30	483	3	.9	1	21	61	81	AAGAG	UCCAG	ation	1
snorf_	Bra015		22					AUGGAGACCUGAACUG	AGUUCUUGAGU-	Cleava	
2_30	521	3	.9	1	23	344	365	AAGAGUU	CAGGUCUCCAU	ge	1
snorf_	Bra030		18					AUGGAGACCUGAACUG	UCUUCAAUUCAGAUU	Cleava	
2_30	840	3	.3	1	20	994	1013	AAGA	UCUAU	ge	1
snorf_	Bra006		22					UUGGAGACCUGAACUG	ACUCUUCAGUUCUGG	Transl	
2_32	754	2.5	.1	1	22	177	198	AAGAGU	ACUCCAA	ation	2
snorf_	Bra006		23					UUGGAGACCUGAACUG	ACUCUUCAGUUCUGG	Transl	
2_32	754	2.5	.5	1	22	924	945	AAGAGU	ACUCCAA	ation	2
snorf_	Bra020		21					UUGGAGACCUGAACUG	AGAUCUCCGGUUCAGG	Cleava	
2_32	436	2.5	.4	1	23	158	180	AAGAGUU	UUUUCAA	ge	1
snorf_	Bra038		19					UUGGAGACCUGAACUG	GGUUCUUCAGUUUGG	Cleava	
2_32	148	3	.3	1	23	902	924	AAGAGUU	CUCUCCGG	ge	1
snorf_	Bra002		14					UUGGAGACCUGAACUG	AGAUCUCUGGUUCAG	Cleava	
2_32	723	3	.0	1	23	167	189	AAGAGUU	GUUUUCAA	ge	1
snorf_	Bra032		18					UUGGAGACCUGAACUG	AAGUCUUCAGAUCGU	Transl	
2_32	144	3	.8	1	23	934	956	AAGAGUU	GUUUCCAA	ation	1
snorf_	Bra038		23					UUGGAGACCUGAACUG	GGUUCUUUGGACCAG	Cleava	
2_32	611	3	.3	1	23	1122	1144	AAGAGUU	GUCUCCAA	ge	1
snorf_	Bra016		20					UUGGAGACCUGAACUG	GGUUCUUCAGUU-	Transl	
2_32	932	2.5	.3	1	23	9	30	AAGAGUU	AGGUCUCCAG	ation	1
snorf_	Bra022	2.5	17	1	21	61	81	UUGGAGACCUGAACUG	UUCUUCACUUCAAGUC	Transl	1

2_32	483		.9					AAGAG	UCCAG	ation	
snorf_	Bra026		10					UUGGAGACCUGAACUG	CUUUUCACUUCACGUC	Transl	
2_32	846	3	.6	1	21	49	69	AAGAG	UUCAA	ation	1
snorf_	Bra000		20					UUGGAGACCUGAACUG	GUUCUUCAGUUCAAU	Transl	
2_32	658	3	.5	1	22	1025	1046	AAGAGU	UCUCUAG	ation	1
snorf_	Bra025		16					UUGGAGACCUGAACUG	UUCUUCAGUUCAUCU	Transl	
2_32	935	3	.6	1	21	239	259	AAGAG	UUCUAA	ation	1
snorf_	Bra002		14					UUGGAGACCUGAACUG	AAUCUUCACUUGAAGU	Transl	
2_32	246	3	.7	1	22	1049	1070	AAGAGU	CUCCAA	ation	1
snorf_	Bra006		22					AUUGGAGACCUGAACU	ACUCUUCAGUUCUGG	Transl	
2_34	754	3	.3	1	23	177	199	GAAGAGU	ACUCCAAG	ation	2
snorf_	Bra006		23					AUUGGAGACCUGAACU	ACUCUUCAGUUCUGG	Transl	
2_34	754	3	.4	1	23	924	946	GAAGAGU	ACUCCAAG	ation	2
snorf_	Bra010		18					AUUGGAGACCUGAACU	AAUCCUCCGGUUCAGG	Cleava	
2_34	291	2.5	.8	1	24	173	196	GAAGAGUU	UUUUCAAU	ge	1
snorf_	Bra020		21					AUUGGAGACCUGAACU	AGAUCUCCGGUUCAGG	Cleava	
2_34	436	2.5	.2	1	24	158	181	GAAGAGUU	UUUUCAAU	ge	1
snorf_	Bra038		20					AUUGGAGACCUGAACU	GGUUCUUCAGUUUGG	Transl	
2_34	148	3	.6	1	24	902	925	GAAGAGUU	CUCUCCGGU	ation	1
snorf_	Bra002		14					AUUGGAGACCUGAACU	AGAUCUCUGGUUCAG	Cleava	
2_34	723	3	.2	1	24	167	190	GAAGAGUU	GUUUUCAAU	ge	1
snorf_	Bra017		14					AUUGGAGACCUGAACU	AAACAGUUCAGGUCUC	Cleava	
2_34	838	3	.8	1	20	545	564	GAAG	CAAU	ge	1
snorf_	Bra023		20					AUUGGAGACCUGAACU	CCUCGGUUCAAGUUUC	Transl	
2_34	921	3	.1	1	20	837	856	GAAG	CAAU	ation	1
snorf_	Bra015		19					AUUGGAGACCUGAACU	CUUCAGUUCGCGGUU	Transl	
2_34	683	3	.1	1	20	244	263	GAAG	CCAAU	ation	1
snorf_	Bra028		14					UAAUGGAGACCUGAAC	AAAGCUUUAAUUCGG	Cleava	
2_35	460	2.5	.6	1	25	4537	4561	UGAAGAGUU	GUCUCCAUUG	ge	1
snorf_	Bra029		14					AGAAACAAAUCUGAGG	UGUCUCUUAGAUUUG	Cleava	
2_37	006	2.5	.0	1	20	1304	1323	CAUA	UUUCU	ge	1
snorf_	Bra030		21					AGAAACAAAUCUGAGG	UAUACGUCGGGUUUG	Cleava	
2_37	358	3	.3	1	20	383	402	CAUA	UUUCU	ge	1
snorf_	Bra020	3	21	1	20	383	402	AGAAACAAAUCUGAGG	UAUACGUCGGGUUUG	Cleava	1

2_37	560		.3					CAUA	UUUCU	ge	
snorf_	Bra034		18					AGAAACAAAUCUGAGG	UUUGCGUUGGAUUUG	Cleava	
2_37	174	3	.5	1	20	1171	1190	CAUA	UUUCU	ge	1
snorf_	Bra015		21					AGAAACAA-	AUGCAUCAGAUCUUG	Transl	
2_37	990	3	.5	1	19	1151	1170	AUCUGAGGCAU	UUUCU	ation	1
snorf_	Bra028		21					AAAAAGAAACAAAUCU	UGACUUUGAUUUGUU	Cleava	
2_38	371	2.5	.0	1	22	578	599	GAGGCA	UCUUUUU	ge	1
snorf_	Bra040		9.					AAAAAGAAACAAAUCU	UCUCAGUUUUGUUUC	Cleava	
2_38	587	3	6	1	20	23	42	GAGG	UAUUU	ge	1
snorf_	Bra017		20					AAGAAACAAAUCUGAG	CUGCUUCAGAGUUGU	Transl	
2_40	434	2.5	.2	1	20	1571	1590	GCAU	UUCUU	ation	1
snorf_	Bra020		21					AAGAAACAAAUCUGAG	UAUACGUCGGGUUUG	Cleava	
2_40	560	3	.1	1	21	383	403	GCAUA	UUUCUU	ge	1
snorf_	Bra030		21					AAGAAACAAAUCUGAG	UAUACGUCGGGUUUG	Cleava	
2_40	358	3	.1	1	21	383	403	GCAUA	UUUCUU	ge	1
snorf_	Bra040		19					AAGAAACAAAUCUGAG	AUGGCGCAGAAUUGU	Transl	
2_40	779	3	.4	1	20	176	195	GCAU	UUCUU	ation	1
snorf_	Bra038		19					AAGAAACAAAUCUGAG	AUGCCUCAGAAU-	Transl	
2_40	776	3	.3	1	20	1873	1891	GCAU	GUUUCUU	ation	1
snorf_	Bra017		20					AAAGAAACAAAUCUGA	UGCUUCAGAGUUGUU	Transl	
2_42	434	2.5	.9	1	20	1572	1591	GGCA	UCUUG	ation	1
snorf_	Bra028		22					AAAGAAACAAAUCUGA	AUUGACUUUGAUUUG	Cleava	
2_42	371	2.5	.8	1	22	576	597	GGCAUU	UUUCUUU	ge	1
snorf_	Bra017		20					AAGAAACAAAUCUGAG	CUGCUUCAGAGUUGU	Transl	
2_45	434	2.5	.2	1	20	1571	1590	GCAU	UUCUU	ation	1
snorf_	Bra020		21					AAGAAACAAAUCUGAG	AUACGUCGGGUUUGU	Cleava	
2_45	560	3	.1	1	20	384	403	GCAU	UUCUU	ge	1
snorf_	Bra030		21					AAGAAACAAAUCUGAG	AUACGUCGGGUUUGU	Cleava	
2_45	358	3	.1	1	20	384	403	GCAU	UUCUU	ge	1
snorf_	Bra040		19					AAGAAACAAAUCUGAG	GAUGGCGCAGAAUUG	Transl	
2_45	779	3	.4	1	21	175	195	GCAUU	UUUCUU	ation	1
snorf_	Bra038		19					AAGAAACAAAUCUGAG	GAUGCCUCAGAAU-	Transl	
2_45	776	3	.3	1	21	1872	1891	GCAUU	GUUUCUU	ation	1
snorf_	Bra001	2	22	1	20	600	619	AGCAUUUAUACUCUGA	AUCUUCAGGGUGUGA	Cleava	1

2_48	724		.1					GGAU	AUGCU	ge	
snorf_	Bra026		20					AGCAUUUAUACUCUGA	AUCCCCAAAGUGUAAA	Cleava	
2_48	537	2.5	.1	1	20	306	325	GGAU	UGCU	ge	1
snorf_	Bra027		20					AGCAUUUAUACUCUGA	AUCCCCAAAGUGUAAA	Cleava	
2_48	085	2.5	.1	1	20	795	814	GGAU	UGCU	ge	1
snorf_	Bra019		19					AGCAUUUAUACUCUGA	AACCUGAGAGUAUGAA	Cleava	
2_48	598	2.5	.4	1	20	536	555	GGAU	UGCU	ge	1
snorf_	Bra010		23					AGCAUUUAUACUCUGA	AUUCUCGGCGAAUAAA	Transl	
2_48	998	3	.3	1	20	1059	1078	GGAU	UGCU	ation	1
snorf_	Bra021		22					AGCAUUUAUACUCUGA	AGCUUCAGAGGAUGA	Transl	
2_48	774	3	.1	1	20	527	546	GGAU	AUGCU	ation	1
snorf_	Bra015		17					AGCAUUUAUACUCUGA	AUUGUCAGAGUCUGA	Transl	
2_48	729	3	.3	1	20	2408	2427	GGAU	AUGCU	ation	1
snorf_	Bra028		16					AGCAUUUAUACUCUGA	AUCUCCAGAGAAUAAA	Transl	
2_48	876	3	.0	1	20	3635	3654	GGAU	UGUU	ation	1
snorf_	Bra004		22					AGCAUUUUUACUCUGA	UUCAUCAGAGUGAAG	Cleava	
2_49	232	3	.6	1	20	587	606	GGAU	AUGCU	ge	1
snorf_	Bra001		22					AGCAUUUUUACUCUGA	AUCUUCAGGGUGUGA	Cleava	
2_49	724	3	.1	1	20	600	619	GGAU	AUGCU	ge	1
snorf_	Bra021		12					AGCAUUUUUACUCUGA	AUCUUGAGAGUAAGA	Cleava	
2_49	294	3	.3	1	20	782	801	GGAU	GUGUU	ge	1
snorf_	Bra016		17					AGCAUUUUUACUCUGA	AUCUUGAGAGUAAGA	Cleava	
2_49	570	3	.6	1	20	785	804	GGAU	GUGUU	ge	1
snorf_	Bra040		11					AGCAUUUUUACUCUGA	AUUCUCAUAGUAAUAA	Cleava	
2_49	585	3	.3	1	20	1112	1131	GGAU	UGCU	ge	1
snorf_	Bra025		9.					AGCAUUUUUACUCUGA	AUUCUCACAGUAACAA	Cleava	
2_49	408	3	2	1	20	1073	1092	GGAU	UGCU	ge	1
snorf_	Bra027		17					AGCAUUUUUACUCUGA	AUCUUGAGAGGAAAA	Transl	
2_49	290	2.5	.2	1	20	1226	1245	GGAU	AUGCU	ation	1
snorf_	Bra006		13					AGCAUUUUUACUCUGA	AUCCUCAAAGGGAAAA	Transl	
2_49	809	3	.2	1	20	740	759	GGAU	UGUU	ation	1
snorf_	Bra002		16					AUGGGAAUCUCUCUGA	UGUGCAAUAGGAAGA	Transl	
2_51	806	3	.3	1	22	781	802	UGCACA	UUCCCAU	ation	1
snorf_	Bra012	3	22	1	21	1022	1042	AUGGGAAUCUCUCUGA	GCGUAUCAGAGAGAU	Cleava	1

2_51	699		.5					UGCAC	UCUUGU	ge	
snorf_	Bra040		20					AUGGGAAUCUCUCUGA	UUUGCAUUAGAGCGG	Transl	
2_51	463	2.5	.4	1	22	577	598	UGCACA	UUUCCAU	ation	1
snorf_	Bra030		12					AUGGGAAUCUCUCUGA	UACAUCAGAGAGAUUC	Cleava	
2_51	518	2.5	.2	1	20	157	176	UGCA	UCAG	ge	1
snorf_	Bra040		18					AUGGGAAUCUCUCUGA	UUUGCAUUAGAGCGG	Transl	
2_51	497	2.5	.1	1	22	655	676	UGCACA	UUUCCAU	ation	1
snorf_	Bra013		19					AUGGGAAUCUCUCUGA	UUUUCAUCGGAAAGG	Transl	
2_51	550	3	.9	1	22	461	482	UGCACA	UUCCCAU	ation	1
snorf_	Bra020		23					AUGGGAAUCUCUCUGA	UUUUCAUCGGAAAGG	Transl	
2_51	911	3	.5	1	22	473	494	UGCACA	UUCCCAU	ation	1
snorf_	Bra033		20					AUGGGAAUCUCUCUGA	UGCCUCACAGAGAUUU	Cleava	
2_51	876	3	.9	1	20	449	468	UGCA	UCAU	ge	1
snorf_	Bra030		12					UGGGAAUCUCUCUGA	AUACAUCAGAGAGAUU	Cleava	
2_52	518	2.5	.6	1	20	156	175	UGCAC	CUCA	ge	1
snorf_	Bra002		18					UGGGAAUCUCUCUGA	UGUGCAAUAGGAAGA	Transl	
2_52	806	3	.1	1	21	781	801	UGCACA	UUCCCA	ation	1
snorf_	Bra012		22					UGGGAAUCUCUCUGA	GCGUAUCAGAGAGAU	Cleava	
2_52	699	3	.5	1	20	1022	1041	UGCAC	UCUUG	ge	1
snorf_	Bra032		16					UGGGAAUCUCUCUGA	GUACAUAAGAGAGAU	Cleava	
2_52	561	3	.2	1	20	156	175	UGCAC	UCUUA	ge	1
snorf_	Bra037		16					UGGGAAUC-	UGUAUCAGAGAAGAU	Transl	
2_52	660	3	.3	1	19	42	61	UCUCUGAUGCA	UCUCA	ation	1
snorf_	Bra007		19					UGGGAAUCUCUCUGA	GUGCAUCAG-	Transl	
2_52	594	3	.3	1	20	2224	2242	UGCAC	GAGAUUCUUA	ation	1
snorf_	Bra039		15					CUAAAUGGGAAUCUCU	UCAGGGAGAUUCUCA	Cleava	
2_53	448	1	.3	1	20	820	839	CUGA	UUUAG	ge	1
snorf_	Bra034		14					CUAAAUGGGAAUCUCU	CCAGAGAGAUUCUUAU	Cleava	
2_53	045	2	.4	1	20	135	154	CUGA	UUAG	ge	1
snorf_	Bra029		14					CUAAAUGGGAAUCUCU	CCAGAGAAAUUCUUAU	Cleava	
2_53	738	3	.8	1	20	135	154	CUGA	UUAG	ge	1

snoRF Unpollinated ovules

ncRNA_Acc.	Target_Acc.	Expectation	UPE	ncRNA_start	ncRNA_end	Target_start	Target_end	ncRNA_aligned_fragment	Target_aligned_fragment	Inhibition	Multiplicity
snorf_4_2	Bra022523	3	16.4	1	20	288	307	GAGCUGUGAUGAUAUUAGGC	CCCUAAUGUUGUCACAGUUC	Cleavage	1
snorf_4_2	Bra035613	2.5	20.7	1	20	153	172	GAGCUGUGAUGAUAUUAGGC	GCCUAGUCUCGUCACGGCUC	Cleavage	1
snorf_4_2	Bra000855	3	12.5	1	20	1892	1911	GAGCUGUGAUGAUAUUAGGC	GUUUAAUAAUAUCACAGCUU	Cleavage	1
snorf_4_2	Bra032852	3	18.5	1	20	857	876	GAGCUGUGAUGAUAUUAGGC	GUCCGAUAUCAUUACAGCUU	Cleavage	1
snorf_4_2	Bra026759	3	18.1	1	20	203	222	GAGCUGUGAUGAUAUUAGGC	GUCUAAUCACAUCACAGUUC	Cleavage	1
snorf_4_2	Bra004297	3	19.0	1	20	621	640	GAGCUGUGAUGAUAUUAGGC	GCCUGAUCUCAUGACAGUUC	Cleavage	1
snorf_4_2	Bra040986	3	17.9	1	19	3393	3412	GAGCUGUGA-UGAUAUUAGG	UCUGAUAUCAAUCACAGCUC	Translation	1
snorf_4_3	Bra037392	3	11.0	1	20	319	338	GAGCUGUGAUGAUAUUUGGC	UCCAAAUCUCAUCAUAGCUU	Cleavage	1
snorf_4_3	Bra039067	3	19.5	1	20	409	428	GAGCUGUGAUGAUAUUUGGC	GUUGGAUCUCAUCACAGCUC	Cleavage	1
snorf_4_4	Bra005417	3	15.6	1	24	63	86	GGCCGAUGAUUUAAAUAAUUUUGA	UUGGAAUUACAUAAAUCAUCGGUU	Cleavage	1
snorf_4_5	Bra039448	1	15.3	1	20	820	839	CUAAAUGGGAAUCUCUCUGA	UCAGGGAGAUUCUCAUUUAG	Cleavage	1
snorf_4_5	Bra034045	2	14.4	1	20	135	154	CUAAAUGGGAAUCUCUCUGA	CCAGAGAGAUUCUUAUUUAG	Cleavage	1
snorf_4_5	Bra029738	3	14.8	1	20	135	154	CUAAAUGGGAAUCUCUCUGA	CCAGAGAAAUUCUUAUUUAG	Cleavage	1
snorf_4_7	Bra039448	1	15.3	1	20	820	839	CUAAAUGGGAAUCUCUCUGA	UCAGGGAGAUUCUCAUUUAG	Cleavage	1
snorf_4_7	Bra034045	2	14.4	1	20	135	154	CUAAAUGGGAAUCUCUCUGA	CCAGAGAGAUUCUUAUUUAG	Cleavage	1
snorf_4_7	Bra029738	3	14.8	1	20	135	154	CUAAAUGGGAAUCUCUCUGA	CCAGAGAAAUUCUUAUUUAG	Cleavage	1

snorf_4_8	Bra028460	3	14.5	1	23	4537	4559	AUGGAGACCUGAACUGAAGAGUU	AAAGCUUUAAUUCGGGUCUCCAU	Cleavage	1
snorf_4_8	Bra016932	3	20.3	1	23	9	30	AUGGAGACCUGAACUGAAGAGUU	GGUUCUUCAGUU-AGGUCUCCAG	Translation	1
snorf_4_8	Bra022483	3	17.9	1	21	61	81	AUGGAGACCUGAACUGAAGAG	UUCUUCACUUCAAGUCUCCAG	Translation	1
snorf_4_8	Bra015521	3	22.9	1	23	344	365	AUGGAGACCUGAACUGAAGAGUU	AGUUCUUGAGU-CAGGUCUCCAU	Cleavage	1
snorf_4_8	Bra030840	3	18.3	1	20	994	1013	AUGGAGACCUGAACUGAAGA	UCUUCAAUUCAGAUUUCUAU	Cleavage	1
snorf_4_10	Bra001667	3	15.3	1	23	189	211	UAUGGAGACCUGAACUGAGUGGA	UUCAUUCAGUUUAGAUUUCCAUG	Translation	1
snorf_4_10	Bra003395	3	17.1	1	25	942	966	UAUGGAGACCUGAACUGAGUGGAGG	CUCUCACUCAGUGCAGGUCAUCAUA	Cleavage	1
snorf_4_10	Bra014523	3	14.6	1	25	942	966	UAUGGAGACCUGAACUGAGUGGAGG	CUCUCACUCAGUACAGGUCAUCAUA	Cleavage	1
snorf_4_10	Bra007493	3	10.1	1	25	942	966	UAUGGAGACCUGAACUGAGUGGAGG	CUCUCACUCAGUACAGGUCAUCAUA	Cleavage	1
snorf_4_10	Bra024602	3	23.3	1	20	593	612	UAUGGAGACCUGAACUGAGU	ACUCAGUUCGAGUCUCCAAA	Translation	1
snorf_4_10	Bra016345	3	17.3	1	20	671	690	UAUGGAGACCUGAACUGAGU	ACUCAGUUCGAGUCUCCAAA	Translation	1
snorf_4_11	Bra037378	2.5	23.6	1	23	1412	1434	AAUCAAGCCAAUGUUCUGAUCCA	UGGCUCAGAAUAUUGGCUUGAUG	Cleavage	1
snorf_4_11	Bra028461	2.5	15.9	1	24	379	402	AAUCAAGCCAAUGUUCUGAUCCAU	AUAGAUCAGAACAUUAGUUUGAUC	Translation	1
snorf_4_11	Bra000949	3	14.8	1	23	1454	1476	AAUCAAGCCAAUGUUCUGAUCCA	UGGCUCGGAAUAUUGGCUUGAUG	Cleavage	1
snorf_4_13	Bra032960	3	17.8	1	23	521	543	ACUGUUUUACUCGGUGGCAGAAA	UCUCUUCCACCGCGUCAAACAGU	Translation	1
snorf_4_13	Bra027641	2.5	19.4	1	20	2087	2106	ACUGUUUUACUCGGUGGCAG	CUGCCACUGAGGUAAACAGU	Translation	1
snorf_4_13	Bra025882	3	18.7	1	20	1332	1351	ACUGUUUUACUCGGUGGCAG	CUGCAGCCAAGUAGAACAGU	Cleavage	1

snorf_4_15	Bra004232	3	22.6	1	20	587	606	AGCAUUUUUACUCUGAGGAU	UUCAUCAGAGUGAAGAUGCU	Cleavage	1
snorf_4_15	Bra001724	3	22.1	1	20	600	619	AGCAUUUUUACUCUGAGGAU	AUCUUCAGGGUGUGAAUGCU	Cleavage	1
snorf_4_15	Bra021294	3	12.3	1	20	782	801	AGCAUUUUUACUCUGAGGAU	AUCUUGAGAGUAAGAGUGUU	Cleavage	1
snorf_4_15	Bra016570	3	17.6	1	20	785	804	AGCAUUUUUACUCUGAGGAU	AUCUUGAGAGUAAGAGUGUU	Cleavage	1
snorf_4_15	Bra040585	3	11.3	1	20	1112	1131	AGCAUUUUUACUCUGAGGAU	AUUCUCAUAGUAAUAAUGCU	Cleavage	1
snorf_4_15	Bra025408	3	9.2	1	20	1073	1092	AGCAUUUUUACUCUGAGGAU	AUUCUCACAGUAACAAUGCU	Cleavage	1
snorf_4_15	Bra027290	2.5	17.2	1	20	1226	1245	AGCAUUUUUACUCUGAGGAU	AUCUUGAGAGGAAAAAUGCU	Translation	1
snorf_4_15	Bra006809	3	13.2	1	20	740	759	AGCAUUUUUACUCUGAGGAU	AUCCUCAAAGGGAAAAUGUU	Translation	1
snorf_4_17	Bra030997	2.5	11.5	1	20	772	791	AUUUUAUUUCUCUGAUGAUC	GAUCAUGAGAGGAAUGAGAU	Cleavage	1
snorf_4_17	Bra030998	2.5	16.2	1	20	3109	3128	AUUUUAUUUCUCUGAUGAUC	GAUCAUGAGAGGAAUGAGAU	Cleavage	1
snorf_4_17	Bra002333	3	15.8	1	20	2337	2356	AUUUUAUUUCUCUGAUGAUC	UAUCAUCAGUGAAAUAAAAC	Translation	1
snorf_4_17	Bra024670	3	13.0	1	20	504	523	AUUUUAUUUCUCUGAUGAUC	GAUCAUCAGAGCAAUAGGAG	Translation	1
snorf_4_17	Bra038921	2.5	15.8	1	20	232	251	AUUUUAUUUCUCUGAUGAUC	GAUCUUAAGAGAAAUAAGAU	Cleavage	1
snorf_4_17	Bra024202	3	15.1	1	20	1338	1357	AUUUUAUUUCUCUGAUGAUC	GAUCAUCAAAGAAGUGGAGU	Cleavage	1
snorf_4_17	Bra015229	3	15.5	1	20	855	874	AUUUUAUUUCUCUGAUGAUC	GGUUAUCAAGGAAAUGAAAU	Cleavage	1
snorf_4_17	Bra014869	3	20.1	1	20	166	185	AUUUUAUUUCUCUGAUGAUC	GAUCAUCAGAUAGAGAAAAU	Translation	1
snorf_4_17	Bra025087	3	10.9	1	20	1056	1075	AUUUUAUUUCUCUGAUGAUC	GAUCAAGAGAGAAAUAAAAG	Cleavage	1

snorf_4_17	Bra028506	3	18.3	1	20	55	74	AUUUUAUUUCUCUGAUGAUC	GAUCAUGAGAAAGAUGAAAU	Translation	1

snoRF Pollinated ovules

ncRNA_Acc.	Target_Acc.	Expectation	UPE	ncRNA_start	ncRNA_end	Target_start	Target_end	ncRNA_aligned_fragment	Target_aligned_fragment	Inhibition	Multiplicity
snorf_5_1	Bra005417	3	15.6	1	24	63	86	GGCCGAUGAUUUAAAUAAUUUUGA	UUGGAAUUACAUAAAUCAUCGGUU	Cleavage	1
snorf_5_2	Bra039448	1	15.3	1	20	820	839	CUAAAUGGGAAUCUCUCUGA	UCAGGGAGAUUCUCAUUUAG	Cleavage	1
snorf_5_2	Bra034045	2	14.4	1	20	135	154	CUAAAUGGGAAUCUCUCUGA	CCAGAGAGAUUCUUAUUUAG	Cleavage	1
snorf_5_2	Bra029738	3	14.8	1	20	135	154	CUAAAUGGGAAUCUCUCUGA	CCAGAGAAAUUCUUAUUUAG	Cleavage	1
snorf_5_4	Bra039448	1	15.3	1	20	820	839	CUAAAUGGGAAUCUCUCUGA	UCAGGGAGAUUCUCAUUUAG	Cleavage	1
snorf_5_4	Bra034045	2	14.4	1	20	135	154	CUAAAUGGGAAUCUCUCUGA	CCAGAGAGAUUCUUAUUUAG	Cleavage	1
snorf_5_4	Bra029738	3	14.8	1	20	135	154	CUAAAUGGGAAUCUCUCUGA	CCAGAGAAAUUCUUAUUUAG	Cleavage	1
snorf_5_7	Bra028460	3	14.5	1	23	4537	4559	AUGGAGACCUGAACUGAAGAGUU	AAAGCUUUAAUUCGGGUCUCCAU	Cleavage	1
snorf_5_7	Bra016932	3	20.3	1	23	9	30	AUGGAGACCUGAACUGAAGAGUU	GGUUCUUCAGUU-AGGUCUCCAG	Translation	1
snorf_5_7	Bra022483	3	17.9	1	21	61	81	AUGGAGACCUGAACUGAAGAG	UUCUUCACUUCAAGUCUCCAG	Translation	1
snorf_5_7	Bra015521	3	22.9	1	23	344	365	AUGGAGACCUGAACUGAAGAGUU	AGUUCUUGAGU-CAGGUCUCCAU	Cleavage	1
snorf_5_7	Bra030840	3	18.3	1	20	994	1013	AUGGAGACCUGAACUGAAGA	UCUUCAAUUCAGAUUUCUAU	Cleavage	1
snorf_5_8	Bra028460	2	14.6	1	24	4537	4560	AAUGGAGACCUGAACUGAAGAGUU	AAAGCUUUAAUUCGGGUCUCCAUU	Cleavage	1

snorf_5<br>_9	Bra028<br>460	2.5	14<br>.6	1	25	4537	4561	UAAUGGAGACCUGAAC<br>UGAAGAGUU	AAAGCUUUAAUUCGG<br>GUCUCCAUUG	Cleava<br>ge	1
snorf_5<br>_10	Bra006<br>754	3	22<br>.3	1	23	177	199	AUUGGAGACCUGAAC<br>UGAAGAGU	ACUCUUCAGUUCUGG<br>ACUCCAAG	Transl<br>ation	2
snorf_5<br>_10	Bra006<br>754	3	23<br>.4	1	23	924	946	AUUGGAGACCUGAAC<br>UGAAGAGU	ACUCUUCAGUUCUGG<br>ACUCCAAG	Transl<br>ation	2
snorf_5<br>_10	Bra010<br>291	2.5	18<br>.8	1	24	173	196	AUUGGAGACCUGAAC<br>UGAAGAGUU	AAUCCUCCGGUUCAGG<br>UUUUCAAU	Cleava<br>ge	1
snorf_5<br>_10	Bra020<br>436	2.5	21<br>.2	1	24	158	181	AUUGGAGACCUGAAC<br>UGAAGAGUU	AGAUCUCCGGUUCAG<br>GUUUUCAAU	Cleava<br>ge	1
snorf_5<br>_10	Bra038<br>148	3	20<br>.6	1	24	902	925	AUUGGAGACCUGAAC<br>UGAAGAGUU	GGUUCUUCAGUUUGG<br>CUCUCCGGU	Transl<br>ation	1
snorf_5<br>_10	Bra002<br>723	3	14<br>.2	1	24	167	190	AUUGGAGACCUGAAC<br>UGAAGAGUU	AGAUCUCUGGUUCAG<br>GUUUUCAAU	Cleava<br>ge	1
snorf_5<br>_10	Bra017<br>838	3	14<br>.8	1	20	545	564	AUUGGAGACCUGAAC<br>UGAAG	AAACAGUUCAGGUCUC<br>CAAU	Cleava<br>ge	1
snorf_5<br>_10	Bra023<br>921	3	20<br>.1	1	20	837	856	AUUGGAGACCUGAAC<br>UGAAG	CCUCGGUUCAAGUUUC<br>CAAU	Transl<br>ation	1
snorf_5<br>_10	Bra015<br>683	3	19<br>.1	1	20	244	263	AUUGGAGACCUGAAC<br>UGAAG	CUUCAGUUCGCGGUU<br>CCAAU	Transl<br>ation	1
snorf_5<br>_12	Bra006<br>754	2.5	22<br>.1	1	22	177	198	UUGGAGACCUGAACU<br>GAAGAGU	ACUCUUCAGUUCUGG<br>ACUCCAA	Transl<br>ation	2
snorf_5<br>_12	Bra006<br>754	2.5	23<br>.5	1	22	924	945	UUGGAGACCUGAACU<br>GAAGAGU	ACUCUUCAGUUCUGG<br>ACUCCAA	Transl<br>ation	2
snorf_5<br>_12	Bra020<br>436	2.5	21<br>.4	1	23	158	180	UUGGAGACCUGAACU<br>GAAGAGUU	AGAUCUCCGGUUCAG<br>GUUUUCAA	Cleava<br>ge	1
snorf_5<br>_12	Bra038<br>148	3	19<br>.3	1	23	902	924	UUGGAGACCUGAACU<br>GAAGAGUU	GGUUCUUCAGUUUGG<br>CUCUCCGG	Cleava<br>ge	1
snorf_5<br>_12	Bra002<br>723	3	14<br>.0	1	23	167	189	UUGGAGACCUGAACU<br>GAAGAGUU	AGAUCUCUGGUUCAG<br>GUUUUCAA	Cleava<br>ge	1
snorf_5<br>_12	Bra032<br>144	3	18<br>.8	1	23	934	956	UUGGAGACCUGAACU<br>GAAGAGUU	AAGUCUUCAGAUCGU<br>GUUUCCAA	Transl<br>ation	1
snorf_5<br>_12	Bra038<br>611	3	23<br>.3	1	23	1122	1144	UUGGAGACCUGAACU<br>GAAGAGUU	GGUUCUUUGGACCAG<br>GUCUCCAA	Cleava<br>ge	1

snorf_5_12	Bra016932	2.5	20.3	1	23	9	30	UUGGAGACCUGAACUGAAGAGUU	GGUUCUUCAGUU-AGGUCUCCAG	Translation	1
snorf_5_12	Bra022483	2.5	17.9	1	21	61	81	UUGGAGACCUGAACUGAAGAG	UUCUUCACUUCAAGUCUCCAG	Translation	1
snorf_5_12	Bra026846	3	10.6	1	21	49	69	UUGGAGACCUGAACUGAAGAG	CUUUUCACUUCACGUCUUCAA	Translation	1
snorf_5_12	Bra000658	3	20.5	1	22	1025	1046	UUGGAGACCUGAACUGAAGAGU	GUUCUUCAGUUCAAUUCUCUAG	Translation	1
snorf_5_12	Bra025935	3	16.6	1	21	239	259	UUGGAGACCUGAACUGAAGAG	UUCUUCAGUUCAUCUUUCUAA	Translation	1
snorf_5_12	Bra002246	3	14.7	1	22	1049	1070	UUGGAGACCUGAACUGAAGAGU	AAUCUUCACUUGAAGUCUCCAA	Translation	1
snorf_5_18	Bra037378	2.5	23.6	1	23	1412	1434	AAUCAAGCCAAUGUUCUGAUCCA	UGGCUCAGAAUAUUGGCUUGAUG	Cleavage	1
snorf_5_18	Bra028461	2.5	15.9	1	24	379	402	AAUCAAGCCAAUGUUCUGAUCCAU	AUAGAUCAGAACAUUAGUUUGAUC	Translation	1
snorf_5_18	Bra000949	3	14.8	1	23	1454	1476	AAUCAAGCCAAUGUUCUGAUCCA	UGGCUCGGAAUAUUGGCUUGAUG	Cleavage	1
snorf_5_20	Bra004232	3	22.6	1	20	587	606	AGCAUUUUUACUCUGAGGAU	UUCAUCAGAGUGAAGAUGCU	Cleavage	1
snorf_5_20	Bra001724	3	22.1	1	20	600	619	AGCAUUUUUACUCUGAGGAU	AUCUUCAGGGUGUGAAUGCU	Cleavage	1
snorf_5_20	Bra021294	3	12.3	1	20	782	801	AGCAUUUUUACUCUGAGGAU	AUCUUGAGAGUAAGAGUGUU	Cleavage	1
snorf_5_20	Bra016570	3	17.6	1	20	785	804	AGCAUUUUUACUCUGAGGAU	AUCUUGAGAGUAAGAGUGUU	Cleavage	1
snorf_5_20	Bra040585	3	11.3	1	20	1112	1131	AGCAUUUUUACUCUGAGGAU	AUUCUCAUAGUAAUAAUGCU	Cleavage	1
snorf_5_20	Bra025408	3	9.2	1	20	1073	1092	AGCAUUUUUACUCUGAGGAU	AUUCUCACAGUAACAAUGCU	Cleavage	1
snorf_5_20	Bra027290	2.5	17.2	1	20	1226	1245	AGCAUUUUUACUCUGAGGAU	AUCUUGAGAGGAAAAAUGCU	Translation	1
snorf_5_20	Bra006809	3	13.2	1	20	740	759	AGCAUUUUUACUCUGAGGAU	AUCCUCAAAGGGAAAAUGUU	Translation	1

snoRF Embryo

ncRNA_Acc.	Target_Acc.	Expectation	UPE	ncRNA_start	ncRNA_end	Target_start	Target_end	ncRNA_aligned_fragment	Target_aligned_fragment	Inhibition	Multiplicity
snorf_6_3	Bra029738	3	14.8	1	20	135	154	CUAAAUGGGAAUCUCUCUGA	CCAGAGAAAUUCUUAUUUAG	Cleavage	1
snorf_6_3	Bra034045	2	14.4	1	20	135	154	CUAAAUGGGAAUCUCUCUGA	CCAGAGAGAUUCUUAUUUAG	Cleavage	1
snorf_6_3	Bra039448	1	15.3	1	20	820	839	CUAAAUGGGAAUCUCUCUGA	UCAGGGAGAUUCUCAUUUAG	Cleavage	1
snorf_6_1	Bra000855	3	12.5	1	20	1892	1911	GAGCUGUGAUGAUAUUAGGC	GUUUAAUAAUAUCACAGCUU	Cleavage	1
snorf_6_8	Bra001667	3	15.3	1	23	189	211	UAUGGAGACCUGAACUGAGUGGA	UUCAUUCAGUUUAGAUUUCCAUG	Translation	1
snorf_6_9	Bra002352	2	15.8	1	20	841	860	UCAAGUUUGGCUAUUCUGUU	AAUGGAAUAGCAAAACUUGA	Translation	1
snorf_6_8	Bra003395	3	17.1	1	25	942	966	UAUGGAGACCUGAACUGAGUGGAGG	CUCUCACUCAGUGCAGGUCAUCAUA	Cleavage	1
snorf_6_4	Bra003828	3	17.2	1	20	600	619	GAUGAUUCUUCUGCAAUUCU	AGAAUUGUACAAGAAACAUC	Translation	1
snorf_6_1	Bra004297	3	19.0	1	20	621	640	GAGCUGUGAUGAUAUUAGGC	GCCUGAUCUCAUGACAGUUC	Cleavage	1
snorf_6_2	Bra005417	3	15.6	1	24	63	86	GGCCGAUGAUUUAAAUAAUUUUGA	UUGGAAUUACAUAAAUCAUCGGUU	Cleavage	1
snorf_6_6	Bra006739	2.5	16.8	1	20	1231	1250	CUACCCAUAUUAUUUCUGAG	CUCAGAAACAAUAUGGUUAG	Cleavage	1
snorf_6_9	Bra007263	2.5	17.3	1	20	1363	1382	UCAAGUUUGGCUAUUCUGUU	AACAGAAUAGCAAAACAUGA	Translation	1
snorf_6_8	Bra007493	3	10.1	1	25	942	966	UAUGGAGACCUGAACUGAGUGGAGG	CUCUCACUCAGUACAGGUCAUCAUA	Cleavage	1
snorf_6_9	Bra011260	2	9.2	1	20	499	518	UCAAGUUUGGCUAUUCUGUU	AACAAAAUAGGCAAACUUGA	Translation	1
snorf_6_7	Bra013275	2.5	16.1	1	23	1784	1805	UGAUUAAAAACUAUCAGCUCUGA	UCGGAGUUGA-AGUUUUUAAUCA	Cleavage	1

snorf_6_7	Bra013867	3	11.2	1	20	163	182	UGAUUAAAAACUAUCAGCUC	GGGUUGGAAGUUUUUGAUCA	Cleavage	1
snorf_6_8	Bra014523	3	14.6	1	25	942	966	UAUGGAGACCUGAACUGAGUGGAGG	CUCUCACUCAGUACAGGUCAUCAUA	Cleavage	1
snorf_6_9	Bra014985	3	24.0	1	23	303	325	UCAAGUUUGGCUAUUCUGUUACA	UGCAAAAGAAUGGCCAAGCUUGC	Cleavage	1
snorf_6_8	Bra016345	3	17.3	1	20	671	690	UAUGGAGACCUGAACUGAGU	ACUCAGUUCGAGUCUCCAAA	Translation	1
snorf_6_7	Bra019178	2.5	14.9	1	21	1696	1717	UGAUUAAAAACU-AUCAGCUCU	AGAGCUGAUGAGUUUUUGAUCA	Cleavage	1
snorf_6_1	Bra022523	3	16.4	1	20	288	307	GAGCUGUGAUGAUAUUAGGC	CCCUAAUGUUGUCACAGUUC	Cleavage	1
snorf_6_8	Bra024602	3	23.3	1	20	593	612	UAUGGAGACCUGAACUGAGU	ACUCAGUUCGAGUCUCCAAA	Translation	1
snorf_6_4	Bra025126	3	20.4	1	21	668	688	GAUGAUUCUUCUGCAAUUCUG	UAGCAUUGCAGAUGAGUCAUU	Translation	1
snorf_6_4	Bra025654	3	24.5	1	26	464	489	GAUGAUUCUUCUGCAAUUCUGAGGCU	ACCUUGAGAACUGCAGGAGAAUGAUC	Cleavage	1
snorf_6_10	Bra025882	3	18.7	1	20	1332	1351	ACUGUUUUACUCGGUGGCAG	CUGCAGCCAAGUAGAACAGU	Cleavage	1
snorf_6_4	Bra026543	3	18.5	1	21	704	724	GAUGAUUCUUCUGCAAUUCUG	UAGCAUUGCAGAUGAGUCAUU	Translation	1
snorf_6_9	Bra026682	3	15.4	1	21	1787	1807	UCAAGUUUGGCUAUUCUGUUA	UAUCAGAAUUGUCAAGCUUGA	Cleavage	1
snorf_6_1	Bra026759	3	18.1	1	20	203	222	GAGCUGUGAUGAUAUUAGGC	GUCUAAUCACAUCACAGUUC	Cleavage	1
snorf_6_9	Bra026977	3	14.1	1	21	1886	1906	UCAAGUUUGGCUAUUCUGUUA	UAUCAGAAUUGUCAAGCUUGA	Cleavage	1
snorf_6_10	Bra027641	2.5	19.4	1	20	2087	2106	ACUGUUUUACUCGGUGGCAG	CUGCCACUGAGGUAAACAGU	Translation	1
snorf_6_9	Bra027883	2.5	13.4	1	20	781	800	UCAAGUUUGGCUAUUCUGUU	GACACAAUAGCUAAGCUUGA	Cleavage	1
snorf_6_4	Bra028438	2.5	20.7	1	26	488	513	GAUGAUUCUUCUGCAAUUCUGAGGCU	ACCUUGAGAACUGCAGAAGAAUGAUC	Cleavage	1

snorf_<br>6_4	Bra029<br>440	2.5	20<br>.8	1	23	742	764	GAUGAUUCUUCUGCAA<br>UUCUGAG	CAUGGAGUUGCUGAAG<br>GAUCAUC	Cleava<br>ge	1
snorf_<br>6_1	Bra032<br>852	3	18<br>.5	1	20	857	876	GAGCUGUGAUGAUAU<br>UAGGC	GUCCGAUAUCAUUACA<br>GCUU	Cleava<br>ge	1
snorf_<br>6_10	Bra032<br>960	3	17<br>.8	1	23	521	543	ACUGUUUUACUCGGUG<br>GCAGAAA	UCUCUUCCACCGCGUC<br>AAACAGU	Transl<br>ation	1
snorf_<br>6_4	Bra034<br>014	3	19<br>.6	1	21	416	437	GAUGAU-<br>UCUUCUGCAAUUCUG	UGGGAUUGCAGAAGAC<br>AUCAUC	Cleava<br>ge	1
snorf_<br>6_4	Bra034<br>260	2.5	18<br>.7	1	21	957	977	GAUGAUUCUUCUGCAA<br>UUCUG	UGGGACUGCAGAAGAA<br>UCGUC	Cleava<br>ge	1
snorf_<br>6_9	Bra035<br>539	2.5	10<br>.0	1	21	1092	1112	UCAAGUUUGGCUAUUC<br>UGUUA	UCACAGAAUAGCAGAA<br>CUUGA	Transl<br>ation	1
snorf_<br>6_1	Bra035<br>613	2.5	20<br>.7	1	20	153	172	GAGCUGUGAUGAUAU<br>UAGGC	GCCUAGUCUCGUCACG<br>GCUC	Cleava<br>ge	1
snorf_<br>6_7	Bra037<br>647	3	23<br>.4	1	26	1570	1595	UGAUUAAAAACUAUCA<br>GCUCUGAGGG	UCUGAAGAGCUGAUGU<br>CUUUUGAUCA	Transl<br>ation	1
snorf_<br>6_4	Bra039<br>659	3	20<br>.2	1	22	1160	1181	GAUGAUUCUUCUGCAA<br>UUCUGA	UCAACGUUGCAGAAGA<br>GUCAUC	Cleava<br>ge	1
snorf_<br>6_9	Bra040<br>779	3	17<br>.4	1	26	871	896	UCAAGUUUGGCUAUUC<br>UGUUACAUUC	GCAUCUCACAGAAUAG<br>CAGAAUUUGA	Transl<br>ation	1
snorf_<br>6_1	Bra040<br>986	3	17<br>.9	1	19	3393	3412	GAGCUGUGA-<br>UGAUAUUAGG	UCUGAUAUCAAUCACA<br>GCUC	Transl<br>ation	1
