# Supplementary material for: Computational Characterization of ncRNA Fragments in Various Tissues of the Brassica rapa Plant
Source: Noncoding RNA. 2017 Mar 24;3(2):17. doi: 10.3390/ncrna3020017 (PMC5831936; doi:10.3390/ncrna3020017)
Supplement: Supplementary file 1 [file ncrna-03-00017-s001.zip › Supplementary tables.pdf]

Table S1. Frequency of occurrence of various tRNAs in different tissues of *Brassica rapa*

	**Leaves**	**ApicMer**	**Pollen**	**UnpolOv**	**PollOv**	**Embryo**	**Endosperm**
**Ala**	1.10	0.85	0.16	0.98	0.64	0.96	0.69
**Arg**	**6.89***	0.65	0.22	0.76	0.43	0.28	0.10
**Asn**	0.05	0.05	0.03	0.08	0.14	0.03	
**Asp**	**6.73***	0.61	0.24	1.55	0.63	3.36	1.31
**Cys**	0.09	0.11	0.09	0.29		0.08	0.04
**Gln**	0.99	1.08	1.12	1.71	1.77	1.22	1.11
**Glu**	58.23	62.63	78.40	64.99	59.27	76.23	92.19
**Gly**	8.18	23.75	5.56	24.36	31.18	14.04	3.16
**His**	0.12	0.68	0.10	0.37		0.59	1.23
**Ile**	0.02	0.14			0.10	0.05	
**Leu**	0.16	0.33	12.30	0.18	0.56	0.09	
**Lys**	0.19	0.16	0.05	0.83	0.74	0.15	
**Met**	0.25	0.27	0.06	0.35	0.22	0.24	
**Phe**	0.25		1.12				
**Pro**	0.52			0.12		1.29	0.05
**SeC**	0.01		0.00	0.02		0.00	0.01
**Ser**	0.12			0.19		0.05	
**Thr**	1.11			0.29		0.16	
**Trp**							
**Tyr**	3.28	7.96		2.93	3.91	1.15	
**Val**	0.13	0.09	0.19			0.04	
**Pseudo**	**11.59***	0.65	0.35	0.32	0.41	0.27	0.11

Leaves – leaf tissue, ApicMer – apical meristem, Pollen – pollen, UnpolOv – unpollinated ovule, PollOv – pollinated ovule, Embryo – embryo tissues, Endosperm – endosperm tissues. Asterisks indicate difference from all other tissues ($p<0.05$).

Table S2. Frequency of occurrence of various tRFs in different tissues of *Brassica rapa*

	Leaves	ApicMer	Pollen	UnpolOv	PollOv	Embryo	Endosperm
**Ala**	8.06	6.54	0.76	0.11	0.24	4.70	17.09
**Arg**	2.02	0.87	0.90	***13.96**	0.96	4.17	1.50
**Asn**	0.02	0.31	0.11	0.88	0.08	0.13	
**Asp**	***75.40**	14.51	2.16	13.87	8.76	23.15	12.44
**Cys**	0.07	0.16	0.53			0.21	
**Gln**	0.07	0.67	***8.25**	0.65	1.63		
**Glu**	3.38	25.78	29.88	20.80	35.50	11.67	21.65
**Gly**	***7.77**	43.85	56.67	42.15	47.44	51.80	44.20
**His**		1.36	0.25			0.15	0.50
**Ile**	0.01	0.18			0.08	0.21	
**Leu**	0.14	0.59	0.17	3.55	1.37	0.33	
**Lys**	0.07	0.18	0.17	0.74	0.17	0.14	
**Met**	0.06	2.43	0.06	0.47	1.36	2.63	
**Phe**	0.18		0.03				
**Pro**						0.11	
**SeC**			0.03			0.00	
**Ser**	0.03					0.03	
**Thr**	0.08					0.15	
**Trp**							
**Tyr**	1.34	0.07			0.08	0.16	
**Val**	0.10	0.14	0.03			0.45	
**Pseudo**	1.27	2.36	3.15	2.81	2.35	2.71	2.62

Leaves – leaf tissue, ApicMer – apical meristem, Pollen – pollen, UnpolOv – unpollinated ovule, PollOv – pollinated ovule, Embryo – embryo tissues, Endosperm – endosperm tissues. Asterisks indicate difference from all other tissues ($p<0.05$).

Table S3. tRF to tRNA ratio for different tissues

tRnaType	Leaves	ApMer	Pollen	UnpolOvule	PolOvule	Embryo	Endosperm
**Asp_GUC5'**	178.359	2.247	0.848	20.547	2.146	51.914	39.834
**Pseudo_UCC**	25.667	5.091	2.230	12.667	3.895	48.141	25.263
**Gly_UCC**	24.498	7.619	2.403	13.555	6.122	37.944	26.330
**Gln_UUG**	7.666	0.066	2.932	1.721	0.028	2.011	2.112
**Pro_CGG**	5.929			2.143		5.626	0.818
**Ala_AGC**	4.573	0.796	0.160		0.030	0.868	1.827
**Met_CAU**	3.402	0.848	0.096	0.259	0.978	1.255	
**Arg_CCU**	2.127	0.152	0.384	6.356	0.688	4.652	1.375
**Arg_UCU5'**	1.951	0.128			0.094	0.116	
**Gly_GCC**	1.637	0.032	0.047	0.169		1.006	2.616
**Ala_UGC**	1.483	0.739	0.218	0.221	0.108		1.315
**Cys_GCA**	1.457	0.145	0.579	2.976		1.925	3.885
**Ala_CGC**	1.453	0.464	0.045	0.163		0.355	0.692
**SeC_UCA**	1.250		0.833	2.222		6.000	2.250
**His_GUG**	1.042	0.192	0.231	2.026		7.068	4.735
**Glu_CUC**	1.027	0.345	0.776	6.886	0.177	4.582	1.522
**Gly_CCC**	0.982	0.011	0.052	0.684	0.013	1.226	4.743
**Gln_CUG5'**	0.755	0.056		3.702	0.179	1.907	0.667
**Leu_UAG**	0.585	0.167	0.001	2.079	0.381	0.544	
**Val_GAC**	0.500						
**Pro_UGG**	0.317					0.339	
**Phe_GAA**	0.313		0.003				
**Val_CAC**	0.304	0.143	0.016			1.200	
**Ile_UAU**	0.294	0.124			0.114	0.455	
**Leu_UAA**	0.283					3.000	
**Pro_AGG**	0.263					0.500	
**Asn_GUU**	0.200	0.575	0.321	1.343	0.085	0.462	
**Glu_UUC5'**	0.195	0.030	0.032	0.174	0.088	0.243	0.454
**Tyr_GUA5'**	0.181	0.001		0.009	0.003		

**Ser_UGA**	0.171						
**Lys_UUU**	0.160	0.107	0.308	0.095	0.035	0.144	
**Thr_UGU**	0.066						
**Arg_UCG**	0.034	0.094		0.803			
**Pseudo_GGC**	0.032	0.011	0.019	0.152	0.044	0.036	
**Arg_UCU3'**	0.016	0.064		0.450			0.792
**Tyr_GUA3'**	0.004					0.025	
**Asp_GUC3'**	0.002						
**Glu_UUC3'**	0.001					0.0002	
**Gln_CUG3'**		0.031	0.048				
**Thr_CGU**				3.000		3.959	5.497
**Ser_CGA**				0.524		2.041	5.107
**Thr_AGU**				0.361		1.127	
**Ala_AGC**				0.067		0.789	
**Pseudo_UCU3'**						0.005	

The ratio was calculated by dividing the number of tRF reads (shorter or equal to 27 nt) by the number of tRNA reads (longer than 29 nt).

Table S4. Common targets for various ncRFs

tRF	Leaves	ApicMer	Pollen	UnpolOv	PolOv	Embryo	Endosperm	
Bra003753	53	23	15	17	17	13	15	BEL1-like homeodomain 3
Bra021314	31	10	5	8	8	8	9	No annotation
Bra013336	25	10	5	8	8	8	8	Leucine-rich repeat protein kinase
Bra000529	14	42	42	19	25	33	36	proteosome family protein
Bra037748	14	42	30	18	25	33	36	cyclin-dependent kinase E1
Bra037499	10	10	7	3	5	13	1	B-box type zinc finger protein with CCT domain
Bra013528	10	10	7	3	5	13	1	PapD-like superfamily protein
Bra013584	9	28	25	10	13	25	22	WRKY DNA binding protein 31
Bra020153	9	28	25	10	13	26	24	LA RNA-binding protein
Bra012143	9	33	25	10	13	25	21	Unknown
Bra016601	8	21	19	14	16	17	10	alpha/beta-Hydrolases superfamily protein
Bra034753	7	2	2	3	1	2	4	Unknown
Bra003597	7	2	2	3	1	2	4	nudix hydrolase homolog 3, phosphohydrolase
Bra023172	7	2	2	3	1	2	4	Argonaute family protein - AGO2
Bra032111	5	14	15	9	12	8	14	LJRHL1-like 1
Bra033536	4	5	8	2	8	3	2	associated molecule with the SH3 domain of STAM 3

rRF	Leaves	ApicMer	Pollen	UnpolOv	PolOv	Embryo	Endosperm	
Bra030994	5	4		5	5	5	5	Disease resistance protein (TIR-NBS-LRR class) family
Bra034079	5	4		5	5	5	5	Disease resistance protein (TIR-NBS-LRR class) family
Bra013685	5	4		5	5	5	5	unknown

snRF	Leaves	ApicMer	Pollen	UnpolOv	PolOv	Embryo	Endosperm
Bra032383	0	2	2	2	3	2	2

Bra034244	0	1	1	1	1	1	1	
Bra018742	0	8	1	3	6	8	7	Tetratricopeptide repeat (TPR)-like superfamily protein
Bra030327	0	1	1	1	1	1	1	

**snoRF**	**Leaves**	**ApicMer**	**Pollen**	**UnpolOv**	**PolOv**	**Embryo**	**Endosperm**	
Bra039448	5	3	0	2	2	3	0	unknown function
Bra034045	3	3	0	2	2	3	0	Peptidase S24/S26A/S26B/S26C family protein
Bra029738	3	3	0	2	2	3	0	Peptidase S24/S26A/S26B/S26C family protein

Numbers show the number of unique ncRFs targeting specific genes.

Table S5. Frequency of predicted binding of tRFs to various regions of their potential targets in different tissues

Each mRNA target was divided into three sections (5' third, central third and 3' third). The number of tRFs binding to specific regions is expressed in percentage of total.

**tRF**		**Cleavage**			**Translation inhibition**		
**tissue**		**5' third**	**central third**	**3' third**	**5' third**	**central third**	**3' third**
1	leaves	48%	50%	2%	65%	34%	2%
2	ap_meri	68%	31%	2%	23%	75%	2%
3	pollen	66%	34%	0%	15%	85%	0%
4	unpol_ovule	75%	24%	1%	26%	73%	1%
5	pol_ovule	74%	26%	1%	25%	74%	1%
6	embryo	41%	58%	1%	24%	75%	1%
7	endosperm	64%	36%	0%	13%	87%	0%
	**Average**	**63%**	**37%**	**1%**	**26%**	**74%**	**1%**
