# Supplementary figures and images for: Computational Characterization of ncRNA Fragments in Various Tissues of the Brassica rapa Plant
Source: Noncoding RNA. 2017 Mar 24;3(2):17. doi: 10.3390/ncrna3020017 (PMC5831936; doi:10.3390/ncrna3020017)

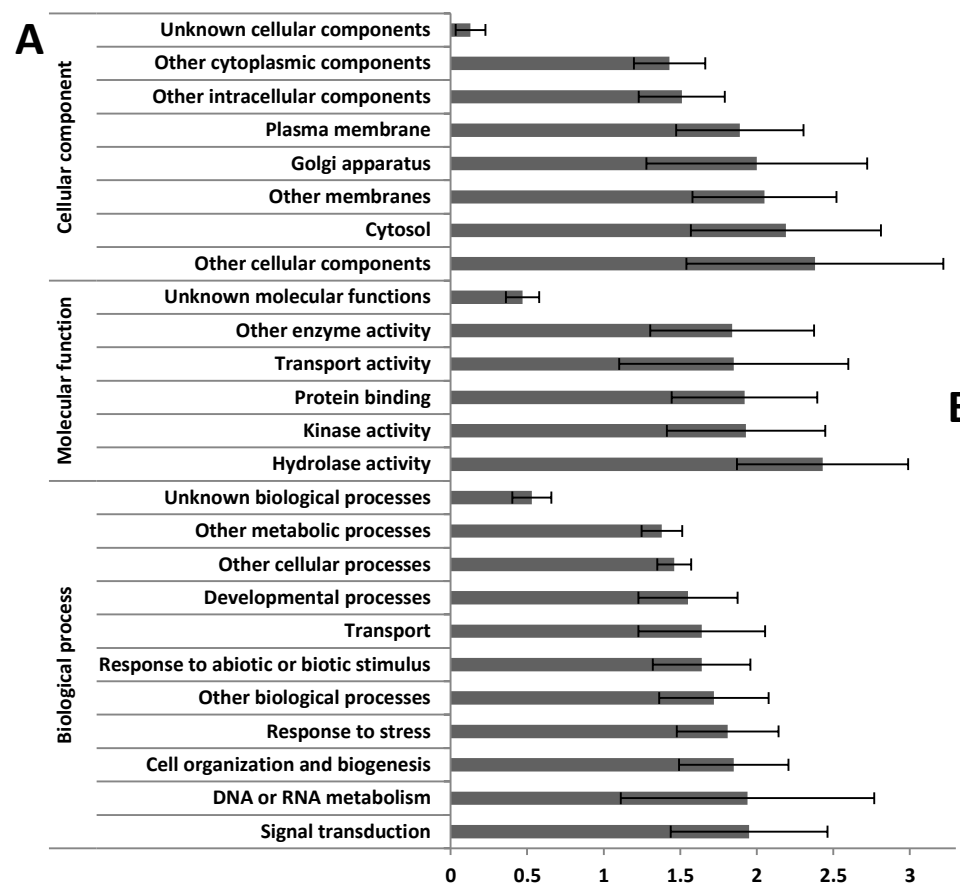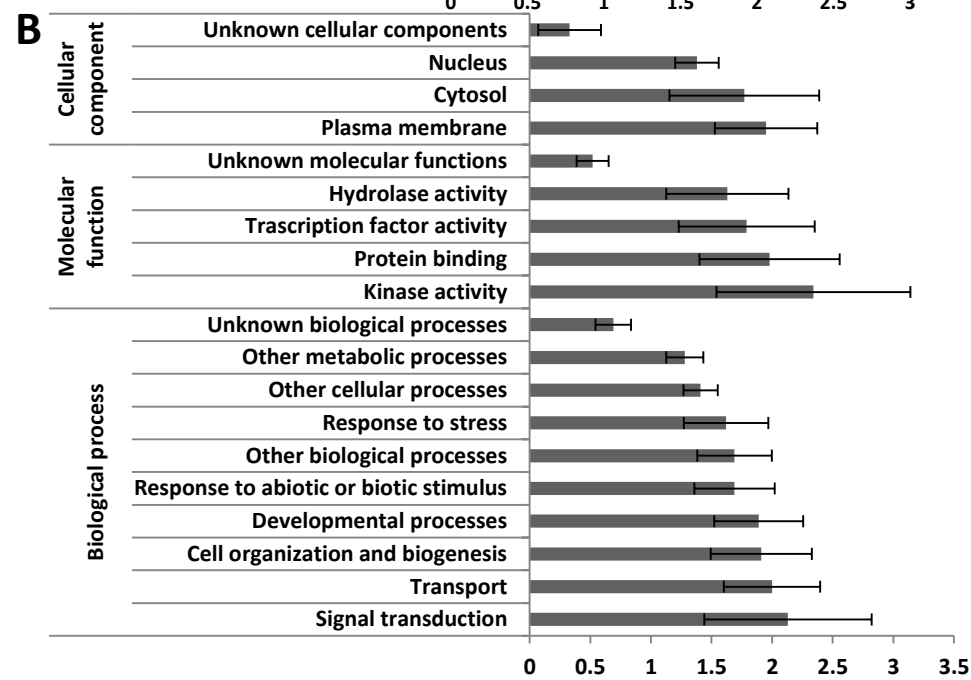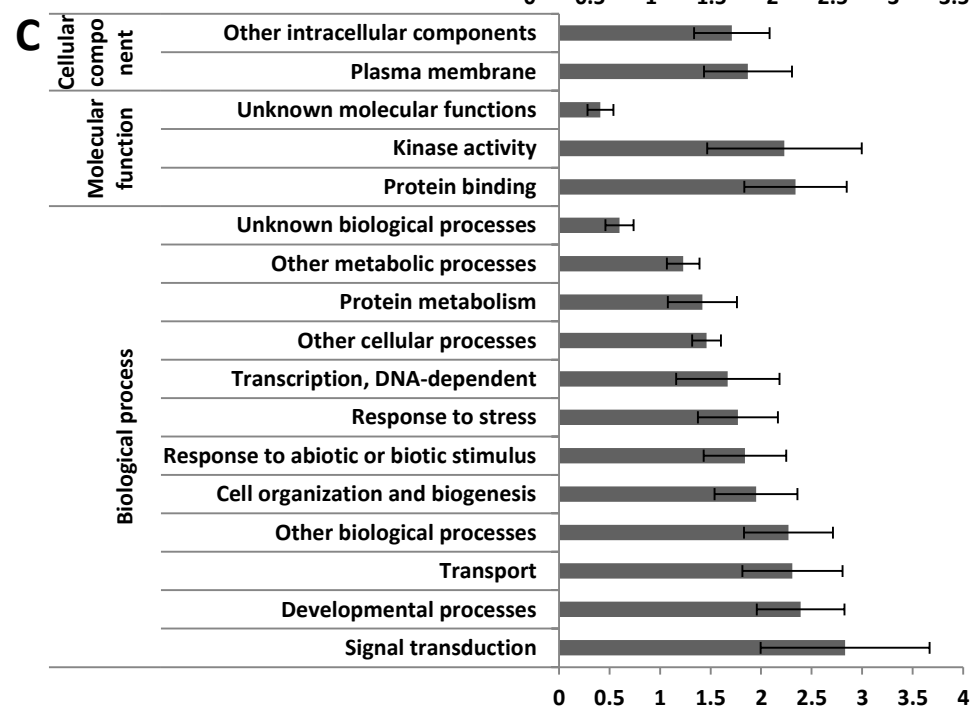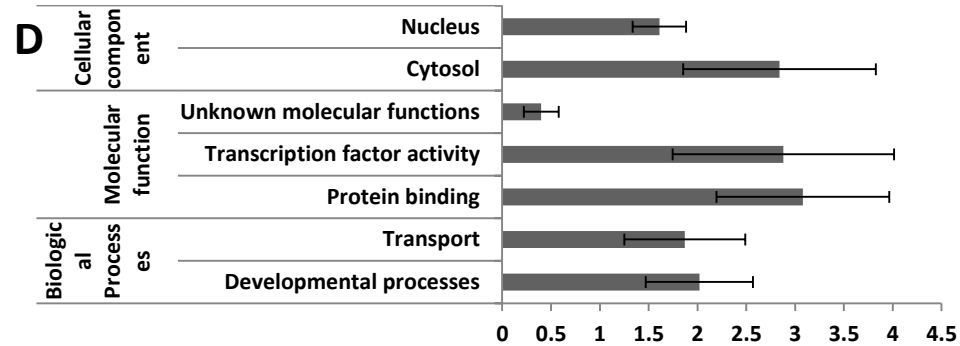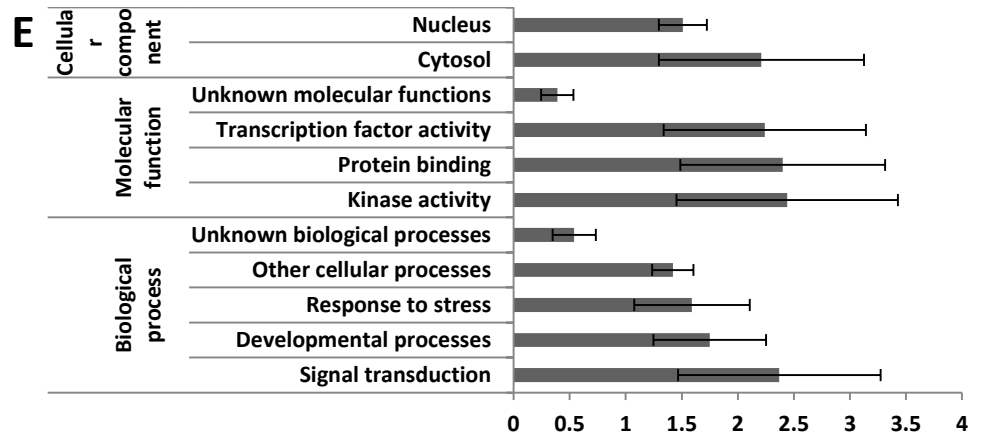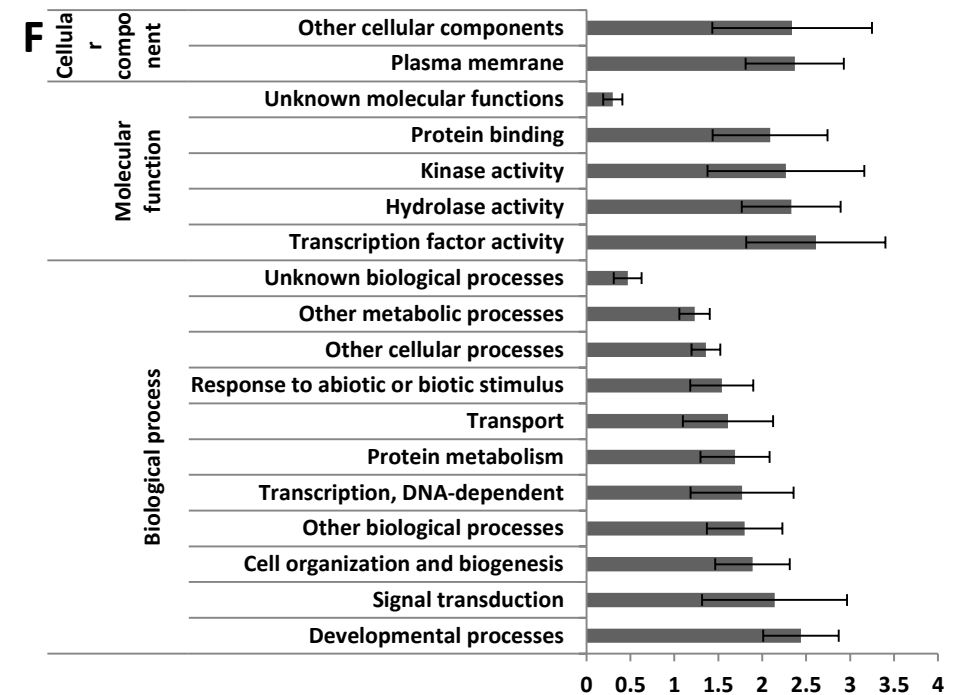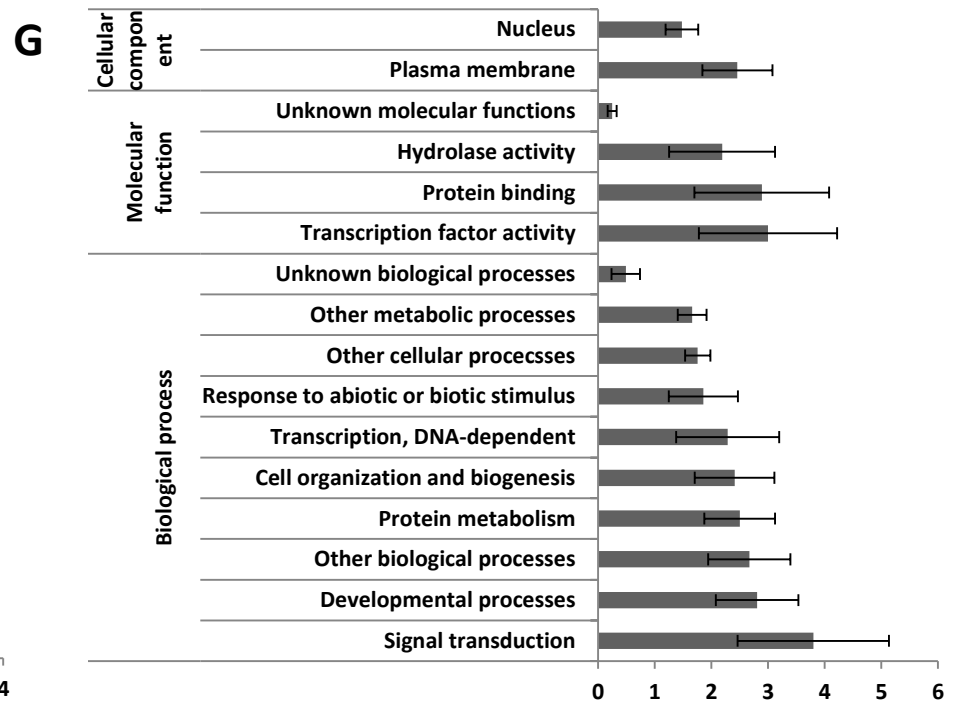

Supplement: Supplementary file 1 [file ncrna-03-00017-s001.zip › Figure S6_SuperViewer tRF.pdf]

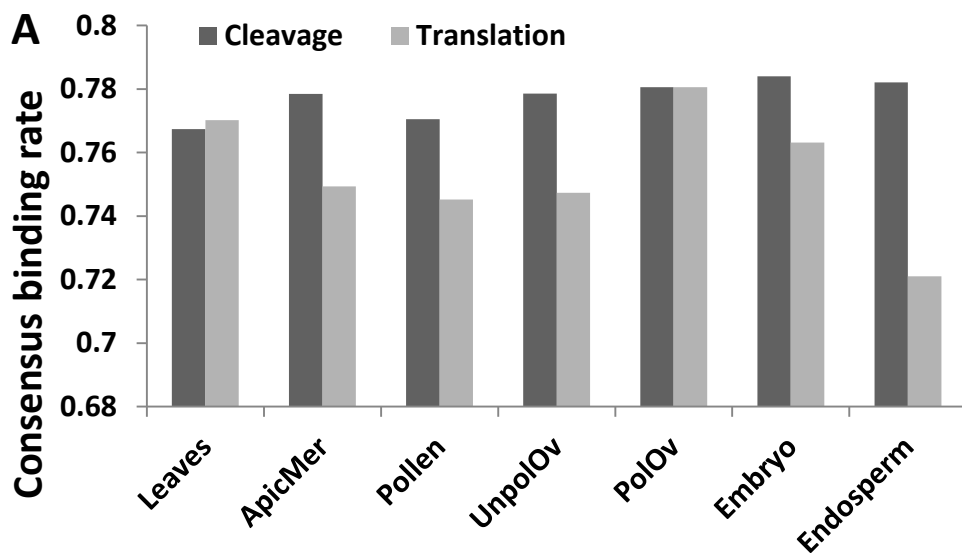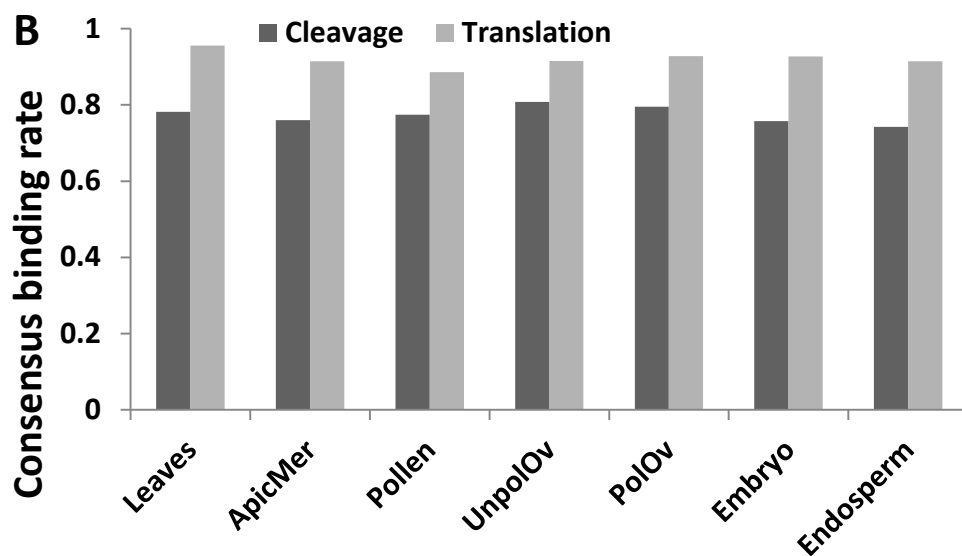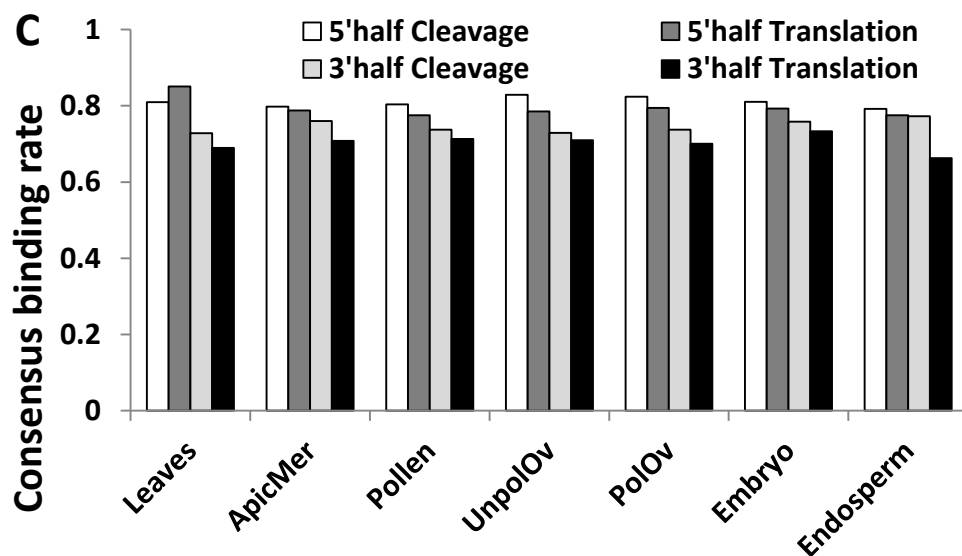

Supplement: Supplementary file 1 [file ncrna-03-00017-s001.zip › Figure S7.pdf]

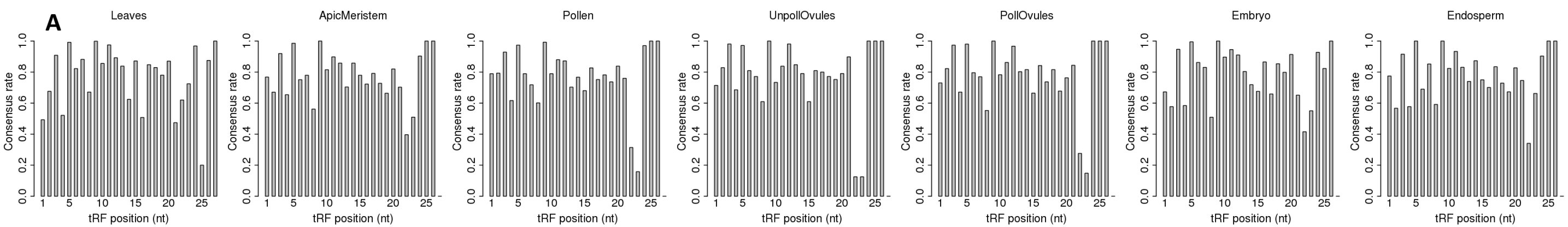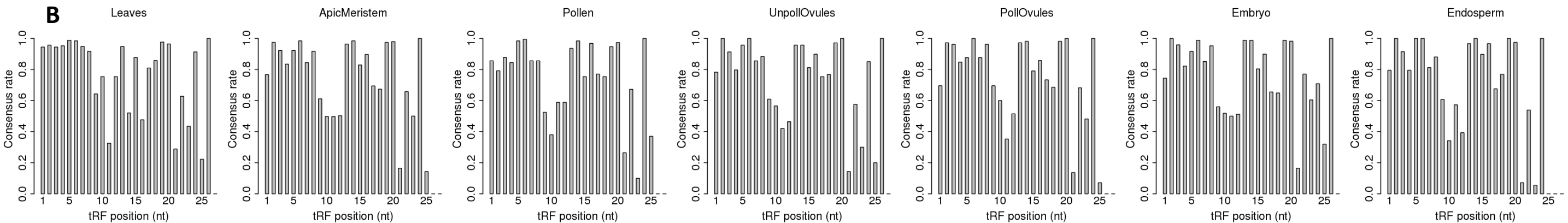

Supplement: Supplementary file 1 [file ncrna-03-00017-s001.zip › Figure S8 new.pdf]
